# Tracing Species Boundaries Through Genomic Baits: Diagnostic SNP Panels for Genotyping Orchid Pollinaria

**DOI:** 10.1111/eva.70315

**Published:** 2026-08-24

**Authors:** Teresa Rosa Galise, Paulo Milet‐Pinheiro, Farida Tara Bandesha, Manfred Ayasse, Donata Cafasso, Salvatore Cozzolino

**Affiliations:** ^1^ Department of Biology University of Naples Federico II Naples Italy; ^2^ Laboratório de Interações Ecológicas e Semioquímicos University of Pernambuco Petrolina Brazil; ^3^ Institute of Evolutionary Ecology and Conservation Genomics, Ulm University Ulm Germany

**Keywords:** 20KOrchidBaits, baits, *Catasetum*, *Orchidaceae*, SNP resampling, species‐specific SNPs, target capture sequencing

## Abstract

*Orchidaceae* is one of the largest angiosperm families, with extensive floral morphological diversity, complex pollination systems, and frequent hybridization, which complicate species delimitation. Target capture offers a scalable alternative to whole‐genome sequencing for phylogenomic and ecological applications, but operational thresholds for reliable species identification, particularly from reduced or degraded single‐nucleotide polymorphism (SNP) datasets, remain poorly quantified. We developed the 20KOrchidbaits, a kit of 20,000 nuclear and 400 plastid baits, specifically designed for *Orchidaceae* by using already available (*Phalaenopsis*) and recently sequenced (*Ophrys*) orchid genomes. We then tested the kit for assessing phylogenetic relationships and pollinaria assignment in *Catasetum*, a young and fast‐evolving neotropical orchid genus. In silico tests of the 20KOrchidbaits kit showed high recovery efficiency for nuclear baits, particularly in the subfamilies Apostasioideae, Orchidoideae, and Epidendroideae. The kit also showed robust plastid performance (85.9% average mapping). Applying 20KOrchidbaits to *Catasetum* members confirmed its high recovery efficiency and showed a strong ability to disclose phylogenetic relationships and identify species‐specific SNPs. Using the 20KOrchidbaits kit for *Catasetum* pollinaria genotyping, the pollinaria were accurately assigned to their source orchid species, both with the full dataset of species‐specific SNPs and with subsets of it. The 20KOrchidbaits provide a versatile toolkit for gathering species resolution even in rapidly radiating orchid clades. This tool also represents the first reliable species‐level identification of orchid pollinarium with target capture, surpassing traditional barcode markers.

## 1 Introduction

*Orchidaceae* is the second most diverse angiosperm family, affiliated with five subfamilies (Apostasioideae, Cypripedioideae, Epidendroideae, Orchidoideae, and Vanilloideae) encompassing about 25,000–30,000 species according to different, often debated taxonomic treatments and exceeding 100,000 hybrids and cultivars (Chase 2005; Dressler 2005). Widely distributed globally, these plants thrive in diverse environments, ranging from tropical rainforests to arctic tundra (Kimsey 1980), resulting in a wide range of morphological, physiological, and ecological adaptations (Dressler 1981; Cozzolino and Widmer 2005; Pillon and Chase 2007).

Orchids exhibit extraordinary floral morphological diversity, with significant variations in forms, sizes, and structures among different species. This diversity is accentuated by specialized adaptations for reproduction, often linked to complex interactions with pollinating insects (Ollerton 2017; Widmer et al. 2000). Several orchid lineages underwent extensive adaptive radiation, diversifying into various ecological niches and adapting to diverse environments. Coupling this with the ability of orchids to hybridize readily and their specialization for specific microhabitats, such as epiphytic and terrestrial environments, adds further challenges to species definition (Dressler 1981; Phillips et al. 2020). Taxonomists use morphological, molecular, and ecological data to navigate these complexities. Phylogenetic studies have revealed complex evolutionary relationships within the *Orchidaceae*, and understanding these relationships is crucial for accurate taxonomy, albeit complicated by convergent similarities and evolutionary adaptations (Chase 2005; Chase et al. 2015). DNA analysis has been pivotal in clarifying relationships and identifying hidden diversity. Nevertheless, the taxonomy of several orchid clades remains dynamic and sometimes controversial, with ongoing revisions and discoveries as researchers strive to comprehend the intricate relationships among the most recently radiated lineages (Breitkopf et al. 2015; Bateman et al. 2018; Givnish et al. 2015).

Orchids employ diverse reproductive strategies, relying on intricate pollination mechanisms to ensure successful reproduction. As a result, orchids have been long and extensively studied for their interactions with pollinators (Dodson and Frymire 1961). Pollinator identification has often been considered pivotal for defining the degree of orchid pollination specificity and the occurrence of ecological reproductive isolation, in accordance with the biological species concept (Vereecken et al. 2010, 2013). However, studies on orchid pollination have traditionally suffered from low visitation rates to orchid flowers, especially in species with deceptive pollination systems (Ayasse et al. 2000; Dodson and Frymire 1961). Further, the low population densities commonly observed in epiphytic orchids make direct observations exceedingly time‐consuming and challenging (Silva et al. 2025), especially in the absence of pollinators (Tremblay et al. 2005). Over time, genetic markers have been used to disclose these relationships (Cozzolino and Widmer 2005; Indsto et al. 2005; Widmer et al. 2000), sometimes only with partial conclusions due to the limited resolution power of the employed molecular markers for discrimination at the species level (Mauad et al. 2022).

Whole genome sequencing offers a powerful tool for addressing phylogenetic and ecological challenges in orchids, but it is still time‐consuming and expensive. Orchids possess complex and large genomes which necessitate high costs for sequencing and heavy effort for bioinformatic analyses (Leitch et al. 2009; Cai et al. 2015). Alternatively, reduced‐representation sequencing methods allow cost‐effective examination of specific genome regions (Soltis 2013). Such methods, as the double‐digest restriction site‐associated DNA sequencing (ddRADseq) or genotype by sequencing (GBS), are a practical approach for non‐model organisms with complex genomes, offering cost‐effective marker development (Davey and Blaxter 2010). However, these approaches have an intrinsic limitation in determining homologous sites when comparing diverse datasets and species. Further, incorrect assembly, large amounts of missing data, and overestimation of heterozygosity may occur, necessitating careful consideration of filtering strategies (Bateman et al. 2018; Cozzolino et al. 2020; Gargiulo et al. 2021). Target capture sequencing represents a promising alternative. By employing synthetic DNA or RNA probes (baits), target capture selectively enriches specific genomic regions, and its applications span diagnostics, metagenomics, phylogenetics, museomics, and ecological studies (Andermann et al. 2020; Gasc et al. 2016).

The Angiosperms353 probe set, a universal target capture sequencing kit for any flowering plant, offers ample applications for systematic studies at deep and shallow phylogenetic levels (Johnson et al. 2019). In that case, bait designs are based on highly conserved loci from different species, potentially applicable to many flowering plants (McLay et al. 2021; Siniscalchi et al. 2021). While universal kits offer easy establishment and comparable data sets, taxon‐specific baits are often preferred for improving the phylogenetic resolution of the plant group of interest. The universal Angiosperms353 probe set has recently been evaluated on orchids, showing both advantages and limitations for resolving species‐level relationships (Anthoons et al. 2025). while otherstudies already advanced the field by developing orchid‐specific bait sets, notably Orchidaceae963 and Orchidinae‐205. The Orchidaceae963 set targets 963 single‐copy nuclear genes and has shown excellent performance, especially within Epidendroideae, but its design, only based on *Phalaenopsis equestris* reference genome, limits its broader applicability (Eserman et al. 2021). Similarly, the Orchidinae‐205 kit, which is optimized for terrestrial orchids of the subtribe Orchidinae, performs well within its narrow focus yet does not extend to the full evolutionary diversity of the family (Veltman et al. 2024).

In this work, we develop a new orchid bait set, the 20KOrchidBaits, specifically designed to capture genomic regions across all five orchid subfamilies. By combining nuclear and plastid loci, the kit integrates biparentally and uniparentally inherited markers, enhancing phylogenetic resolution and enabling the detection of hybridization and plastome capture events (Cafasso et al. 2005; Chang et al. 2000). Once designed, the bait set was validated both in silico and empirically, testing its performance in phylogenetic and ecological applications (i.e., pollinaria barcoding) in the genus *Catasetum* (Epidendroideae), a fast‐radiating neotropical orchid clade (Ramírez et al. 2011; Pérez‐Escobar et al. 2016, 2024) famous for being pollinated by male euglossine bees (Hills et al. 1972; Milet‐Pinheiro and Gerlach 2017). By genotyping both plants and their pollinaria, we evaluated the reliability and traceability of pollinaria identification by using species‐specific single‐nucleotide polymorphisms (SNPs) for species assignments. By implementing replicate subsampling and tree reconstruction, we empirically determined the minimum number of SNPs required to achieve stable phylogenetic topologies and accurate pollinarium‐to‐species assignments. This approach provides practical thresholds for assessing genomic traceability and contributes a robust, reproducible framework for applying target capture data to ecological and evolutionary studies in orchids.

## 2 Materials and Methods

### 2.1 General Workflow for Selection of Orchid Baits

We adopted two complementary and parallel strategies for bait design: one focused on capturing nuclear genomic regions and the other tailored for plastid genome targets, each optimized for high efficiency and broad coverage. Nuclear and plastid baits were designed independently using a sliding window approach, with filtering based on guanine–cytosine (GC) content, alignment identity, and absence of paralogous or repetitive elements. These two sets of baits were then merged into a single comprehensive dataset, which is made publicly available via GitHub (https://github.com/terryr92/20KOrchidbaits.git).

We utilized the genomes and nuclear transcripts of *Ophrys sphegodes* (*v2.4* submitted genome in Russo et al. 2024; *GHXJ01000001*; Orchidoideae) and *Phalaenopsis equestris* (GCA_001263595.1; GDHE01000001; Epidendroideae), which represented the available orchid nuclear genomes at the time we started designing the nuclear baits. These latter were tiled within exonic and intronic regions of single‐copy genes in these two annotated reference genomes/transcripts with the mrbait software (Chafin et al. 2018). A subset of single‐copy genes was retained after homology and copy‐number filtering, so that each target locus corresponds to an individual single‐copy gene represented by one or more 100‐bp baits. Target regions were selected based on sliding window regions between 500 and 1000 bp, with a GC composition between 20% and 50% and without gaps. Passing target regions were parsed to design a putative set of baits, both as designed and reverse complement, with a length of 100 bp and a maximum allowable variant count of 5. The resulting loci sequences and bait sequences were stored in an SQL3 database. The baits that tile within the same target region belong, by design, to the same nuclear locus. We did not impose an explicit minimum inter‐locus distance, as the gene‐based, genome‐wide distribution of the targets already minimises linkage. Since the nuclear genomes were only available in an unassembled state at the chromosomal level, specific baits were designed for each genome. To compare the designed baits, we used BLASTn to align them to the genome and transcriptome of the other orchid species (Camacho et al. 2009). We established strict criteria to increase single‐copy loci by only keeping baits with a 90%–95% identity and mapping only once on each reference genome.

We chose this dual‐filter approach to avoid targeting highly conserved or overly variable regions and to exclude baits mapping to multiple locations within repeated regions. The resulting baits were then locally mapped onto the genomes (*O. sphegodes* and *P. equestris* ) using Bowtie2 (Langmead and Salzberg 2013) and statistically evaluated using SAMtools software (Li et al. 2009). For the design of plastid baits, we conducted a multiple alignment of plastome genomes, including one representative for each of the five orchid subfamilies (*O. sphegodes*, *P. equestris* , *Vanilla planifolia* , *Apostasia odorata*, *Cypripedium calceolus* ).

The plastid baits, like nuclear baits, were generated using the mrbaits software (Chafin et al. 2018), selecting 500 bp sliding windows with a CG content between 20% and 50%, a length of 100 bp, and a 50% overlap in the same target region.

The plastid baits were mapped onto the reference plastomes using BLASTn (Camacho et al. 2009) with a 90%–95% identity threshold. We then evaluated whether any baits fell within the duplicated inverted repeat regions (IRa and IRb), and all plastid baits mapping to these regions were removed. The final bait set, nuclear and plastidial (hereafter 20KOrchidbaits), was synthesized by Daicel Arbor Biosciences (Ann Arbor, MI, USA).

### 2.2 In Silico Evaluation of 20KOrchidbaits

To comprehensively evaluate the performance of 20KOrchidbaits, we conducted an in silico assessment of the selected bait set. The software CapSim (Cao et al. 2018) was used to estimate the efficiency of the bait set for recovering loci among members of the orchid family. Nuclear bait performance was evaluated in silico using 38 orchid nuclear genomes (2 Apostasioideae, 1 Cypripedioideae, 15 Orchidoideae, 19 Epidendroideae, 1 Vanilloideae), whereas plastid performance was assessed using 634 complete plastomes (476 Epidendroideae, 41 Orchidoideae, 106 Cypripedioideae, 7 Vanilloideae, 4 Apostasioideae). All genomes and plastomes were downloaded from NCBI (September 2024), and they were reported in the (Tables S1 and S2).

Before running CapSim, the baits were aligned to the genomes or transcriptomes using Bowtie2, as recommended in CapSim guidelines (https://github.com/mdcao/capsim). The mapped reads were sorted and indexed into BAM files using SAMtools v1.9 (Li et al. 2009) and used as input for CapSim. We set the median fragment size at shearing (‐‐fmedian) to 600 bp, simulated Illumina Miseq (‐‐miseq), and the read length (‐‐illen) to 300 bp, generating simulated reads that were saved as raw fastq paired‐end (PE) reads.

HybPiper v2 pipeline (Johnson et al. 2016) mapped in silico reads to reference target sequences and bait sets using BWA (Li and Durbin 2010). The specificity (i.e., the proportion of reads in baits) and the efficiency (i.e., the proportion of baits covered by at least three reads) were calculated for each sample using the TEQC R package (Hummel et al. 2011).

To identify the core set of baits shared within and among the five orchid subfamilies, the lists of recovered baits from all in silico samples were imported into R v4.5 (R Core Team 2025). These lists were matched using exact string comparisons, and shared targets were identified by computing the intersection of bait sets across subfamilies (intersect). Baits recovered exclusively within or outside specific lineages were quantified using set difference operations (setdiff). This procedure enabled us to determine the conserved bait fraction consistently captured across all subfamilies and to define the universal core of the 20KOrchidbaits.

### 2.3 Experimental Evaluation of 20KOrchidbaits

The 20KOrchidbaits kit was experimentally validated by Milet‐Pinheiro et al. (in prep.), on a broad range of wild‐collected *Catasetum* species, by performing hybridization with the baits following the manufacturer's protocol (v4.0; http://www.arborbiosci.com/mybaits‐manual) and sequencing the libraries on an Illumina MiSeq platform (2 × 250 bp PE reads). From the Milet‐Pinheiro et al. (in prep.) dataset, we selected 22 *Catasetum* species with available pollinaria for genotyping (see below) (Tables S3 and S4).

To evaluate how efficiently the 20KOrchidbaits capture genomic regions across these 22 species, we used the trimmed reads of the samples (Tables S3 and S4) and mapped them to the reference target sequences using the HybPiper v2 pipeline and the TEQC R package as described above. The reads were partitioned into nuclear and plastid datasets based on their mapping to the corresponding bait sets. The nuclear and plastid datasets were processed independently in the subsequent steps. Then, to assess how many baits recovered in *Catasetum* belong to the conserved target set predicted for Epidendroideae, the two lists of recovered baits were imported into R v4.5 (R core team 2025) and matched using exact string comparisons (base functions intersect, setdiff) as described above.

### 2.4 Construction of the SNPs Panel for *Catasetum*

To generate a panel of diagnostic species‐specific SNPs for the 22 *Catasetum* species, the reads mapping to the baited regions were extracted directly from the BAM files generated by HybPiper v2 by using SAMtools v1.9 (Li et al. 2009) and converted back to FASTQ format for downstream reprocessing. Then all reads were re‐aligned with Bowtie2 v2.4.5 under end‐to‐end alignment mode to minimize false SNP calls and improve genotype reliability (Langmead and Salzberg 2013), and variant calling was performed with BCFtools v1.17 (Li et al. 2009). SNPs were subsequently filtered with VCFtools v0.1.13 (Danecek et al. 2011) using the following thresholds: minimum coverage ≥ 8×, Phred quality ≥ 100, exclusion of indels and all heterozygous sites, and ≤ 5% missing data. These filtered VCFs were imported into R v4.5 using the vcfR package (Knaus and Grünwald 2017).

Genotypes were extracted using the *extract.gt* function in the vcfR R package to retain only SNPs that satisfied the criteria for species‐specificity. In detail, SNPs were considered species‐specific when they were (i) exclusive to a single species, (ii) present in a homozygous state relative to the reference.

The missing data were filtered in a taxon‐aware way: we kept only homozygous SNPs in the VCF files that were unique to a species and present in all other species with the alternative allele. Any SNPs with missing data in any species were removed.

We estimated the number of nuclear and plastid species‐specific SNPs in each species and tested whether species with higher numbers of species‐specific nuclear SNPs also possessed higher numbers of species‐specific plastid SNPs using Pearson correlation analyses, implemented in the base R v4.5 function cor.test() (R Core Team 2025).

The resultant species‐specific VCF files (nuclear and plastidial) were used to quantify genetic divergence among the 22 *Catasetum* species using pairwise p‐distances. These were computed using a genotype‐aware metric in which homozygous differences represent complete allelic substitutions (0/0 vs. 1/1 = 1), using the rdist R package (Brown 2024).

To estimate intraspecific genetic variation, the species‐specific SNP panel was additionally evaluated across a subset of species from which multiple conspecific accessions were available in Milet Pinheiro et al. (in prep.), namely *C. denticulatum* , *n* = 7; *C. pulchrum* , *n* = 4; *C. lanciferum*, *n* = 3; *C. gardneri* , *C. hookeri* , and *C. tigrinum* , *n* = 2.

For each of these species, genotypes were extracted at the species‐specific nuclear and plastid SNP positions from the combined multi‐sample VCF, and pairwise distances among conspecific individuals were computed with the same genotype‐aware pdistance metric described above (rdist R package; Brown 2024). For each species, the number of polymorphic sites among conspecific individuals was tallied separately for the nuclear and plastid sets and averaged across species to obtain a mean estimate of intraspecific variation, which was compared with the pollinarium‐to‐reference distances (Table S5).

### 2.5 Pollinarium Genotyping

We assessed the efficiency of the 20KOrchidbaits in genotyping and assigning pollinaria9 to the corresponding reference *Catasetum* species. While the plant material of the 22 *Catasetum* species was collected in the wild, 56 pollinaria (one from each plant) were manually collected from flowering plants of the same 22 *Catasetum* species cultivated in three plant nurseries in the municipality of Rolim de Moura, Rondônia, North Brazil. The *Catasetum* plants of these nurseries were originally collected in the surrounding forests of Rondônia and cultivated in pots.

Total genomic DNA was extracted from the single pollinarium using the CTAB method (Doyle and Doyle 1987). DNA concentration was measured with a Qubit BR assay (Thermo Fisher Scientific), and DNA quality was assessed using a NanoDrop spectrophotometer (Thermo Fisher Scientific) and agarose gel electrophoresis. Fully constructed libraries were hybridized to 100 bp baits, and bait–DNA hybrids were captured on streptavidin‐coated magnetic beads using the 20KOrchidBaits, by performing hybridization with the baits following the manufacturer's protocol (v4.0; http://www.arborbiosci.com/mybaits‐manual). The resulting pollinaria DNA libraries were pooled equimolarly and sequenced on the Illumina NovaSeq X platform (2 × 150 bp PE reads). Raw reads were filtered with Trimmomatic (Bolger et al. 2014) to remove bases with Phred scores < 30 and to discard reads shorter than 60 bp. Overlapping read pairs were merged with FLASH (Magoč and Salzberg 2011) using a minimum overlap of 10 bp and a maximum mismatch ratio of 0.1, producing longer sequences. Non‐overlapping read pairs and singletons were retained for downstream analyses. To generate the pollinaria VCF file, the reads were mapped to reference target sequences by using the same procedure and settings as described above for the reference species.

To integrate pollinaria collected from the nurseries (and sequenced in this study) and wild‐collected reference species (sequenced by Milet‐Pinheiro et al. in prep.) within a single analytical framework, pollinaria VCF and species‐specific VCF were merged using BCFtools merge (Li et al. 2009), producing a multi‐sample combined VCF. This combined VCF was then filtered to retain only the species‐specific SNP as defined in the species‐specific VCF, using the vcf_subset() function of the vcfR R package (Knaus and Grünwald 2017). This approach enabled the identification of the most likely species assignment for each pollinarium based solely on the fixed set of species‐specific diagnostic SNPs. Pairwise nuclear p‐distances among all samples, including reference species and pollinaria, were calculated using the same genotype‐aware metric described above. The most likely species assignment for each pollinarium was inferred from a maximum‐likelihood (ML) tree built from the full set of nuclear species‐specific SNPs in IQ‐TREE2 v2.2 (Minh et al. 2020) under the GTR + F + ASC model—the same framework used in the subsampling analysis—with nodal support estimated from 1000 ultrafast bootstrap (UFBoot) and 1000 SH‐aLRT replicates. This allowed a direct assessment of each pollinarium's placement relative to its reference species, with branch support reported for every species cluster.

### 2.6 Evaluation of Assignment Accuracy Under Progressive SNPs Reduction

To evaluate how the reduction in the number of SNP loci affects pollinarium assignment and phylogenetic signal, we implemented a SNP subsampling strategy following Koch et al. (2021). The full SNP dataset (100%) was defined as the species‐specific VCF file. From this full SNP dataset, we randomly sampled decreasing fractions of SNPs (75%, 50%, 25%, and 10% of the species‐specific VCF file), generating 500 independent SNP subsets for each subsampling level (i.e., 500 SNP subsets in total). Custom Python and R scripts were developed to automate the SNP subsampling (available at GitHub: https://github.com/terryr92/20KOrchidbaits.git).

Each SNP subset was then used to infer a phylogenetic representation in IQ‐TREE2 v2.2 (Minh et al. 2020) under the GTR + F + ASC model, with nodal support estimated using 1000 ultrafast bootstrap (UFBoot) and 1000 SH‐aLRT replicates. For each tree, we quantified assignment accuracy as the proportion of pollinarium clustering with their respective reference species (range 0–1). From the same trees, we also extracted treeness (t), saturation (R^2^), and median bootstrap support (boot) to assess the stability of phylogenomic signal under progressive SNP depletion.

Finally, to evaluate how SNP subsampling influenced the recovery and stability of the underlying genetic cluster (pollinaria and reference species), we estimated the optimal number of genetic clusters (K) using the snmf algorithm implemented in the LEA package (Frichot and François 2015). These analyses were performed on both the full SNP dataset and on all subsampled SNP matrices, scanning values of *K* = 20–60 and adopting the settings recommended by Janes et al. (2017) for assessing clustering robustness under reduced genomic representation. This approach allowed us to quantify how decreasing SNP density affects the resolution and consistency of inferred genetic clusters.

The minimum number of species‐specific SNPs required for reliable assignment was estimated by modelling assignment accuracy as a function of SNP density with a logistic function, acc(*x*) = 1/(1 + exp.(−*k* (*x* – *x*_0_))), where *x* is the number of species‐specific SNPs, and *k* and *x*_0_ are the steepness and inflection‐point parameters. The model was fitted by nonlinear least‐squares (curve_fit, scipy.optimize; Virtanen et al. 2020) to all 500 individual replicate observations, each contributing the SNP number of its subset and the assignment accuracy of the corresponding IQ‐TREE2 tree, under a binomial error structure (56 pollinaria per replicate). The SNP density yielding ≥ 95% assignment accuracy was derived from the fitted curve, and its 95% confidence interval was obtained by non‐parametric bootstrap (1000 resamples) of the 500 replicate observations.

All calculations and curve‐fitting procedures were conducted in Python 3.9 using the SciPy and NumPy libraries, and the resulting logistic function was plotted using Matplotlib v3.7.

## 3 Results

### 3.1 Baits Selection for the 20KOrchidbaits

The nuclear bait set was initially designed from 1,680,457 candidate loci, comprising 769,809 *Ophrys* and 910,648 *Phalaenopsis* loci derived from their genomes and transcriptomes. All candidate bait sequences were initially evaluated through homology‐ and copy‐number filtering, yielding a first subset of 25,801 nuclear loci suitable for downstream validation. A further mapping‐based screening refined this selection, ultimately confirming 23,212 single‐copy nuclear regions, consistently recovered across both reference genomes and transcriptome data. Of these, the first 20,000, based on their in silico capture performance and the breadth and consistency of their recovery across the 38 orchid genomes, were selected for the nuclear bait set.

The plastid baits were designed from 27,103 candidate target regions generated from the multiple plastome alignment. Following identity filtering and stringent removal of all sequences mapping within the duplicated inverted repeat regions (IRa/IRb) using BLASTn and SAMtools, 400 plastid baits were retained as high‐confidence markers.

The finalized bait set was combined by a total of 20,000 nuclear and 400 plastid baits, constituting the 20KOrchidbaits set, now commercially available through Arbor Biosciences (Ann Arbor, MI, USA). Bait and target sequences were available in the project repository: https://github.com/terryr92/20KOrchidbaits.git.

### 3.2 In Silico Evaluation of 20KOrchidbaits

**FIGURE 1:**
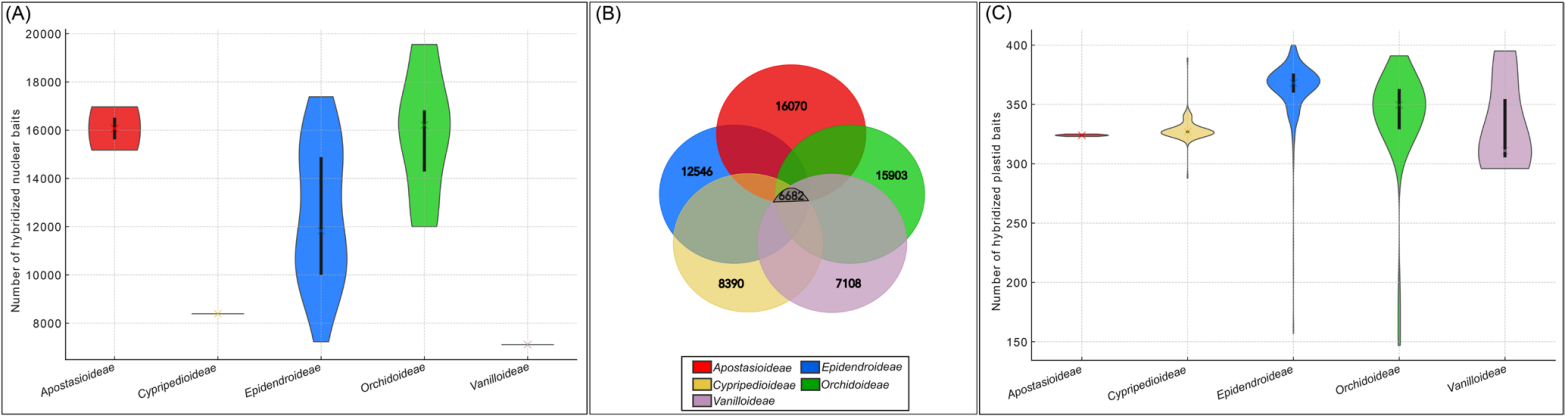
In silico hybridization efficiency of the 20KOrchidbaits probe set among orchid subfamilies. (A) Distribution of the number of nuclear loci recovered per species in each subfamily. (B) The core set of 6682 nuclear baits was shared among orchid subfamilies, representing the universal fraction of the bait set. (C) Distribution of the number of plastid loci recovered per subfamily. For panels A and C, the black vertical bars indicate the interquartile range (IQR), and the dots correspond to median values, following the colour palette shown in panel B.

When tested with the nuclear genomes of 38 orchid species, the 20KOrchidbaits set recovered an average of 14,325 nuclear loci per genome (median = 14,832; IQR = 11,767–16,328; range = 7108–19,555; Table S1, and Figure 1A), with, on average, 81.49% of the in silico reads mapped among the species. The set generally retained more nuclear loci from the Apostasioideae, Epidendroideae, and Orchidoideae subfamilies (see Table S1 and Figure 1A). A core set of 6682 nuclear loci was shared across the five orchid subfamilies, based on in silico recovery simulation at coverage ≥ 3, representing the universal fraction of our bait set (Figure 1B; Table S3; https://github.com/terryr92/20KOrchidbaits.git). Because the bait set was designed from genes and transcripts rather than from chromosome‐level assemblies (not yet available), this core set of nuclear loci is defined at the level of individual genes rather than by chromosomal coordinates. However, as each target locus is an independent single‐copy gene, physical linkage among loci is expected to be low.

We selected all available (634) orchid plastomes to evaluate the efficiency of the plastid baits in silico hybridization (see Table S2, and Figure 1C). The plastid bait set showed good performance across all orchid subfamilies, with an average of 356 plastid baits (85.91% of reads mapped) recovered per species (median = 364; IQR = 340–373; range = 147–400; Table S2; Figure 1C). Among subfamilies, the Epidendroideae exhibited the highest average recovery, with 365 plastid loci (Table S2; Figure 1C). A plastid core set of 300 loci (as determined from the in silico screening of the 634 orchid plastomes) was shared across all five orchid clades (dataset available at https://github.com/terryr92/20KOrchidbaits.git).

A few species showed substantially reduced nuclear and plastid bait recovery (for instance, *Vanilla planifolia* (Vanilloideae) with 7108 nuclear loci and *Cypripedium japonicum* (Cypripedioideae) with 157 plastid loci; see Tables S1 and S2 for details).

### 3.3 Experimental Evaluation of 20KOrchidbaits

Concordantly with the in silico predictions, the experimental approach applied to the 22 *Catasetum* species recovered a total of 13,500 nuclear baits with coverage ≥ 1, corresponding to an average of 10,979 nuclear baits per species at coverage ≥ 3, with 71.14% of reads mapped (Table S4). Similarly, the plastid bait set recovered a total of 377 loci for *Catasetum* species, with an average of 333 plastid baits per species at coverage ≥ 3, and 5.37% of reads mapped (Table S4).

Overall, the captured loci in *Catasetum* overlapped extensively with the conserved bait subset identified in the in silico screening of orchid subfamilies (dataset available at https://github.com/terryr92/20KOrchidbaits.git). In particular, the *Catasetum* loci corresponded closely to those identified in silico (77.65% for nuclear and 94.2% for plastid baits) for Epidendroideae (dataset available at https://github.com/terryr92/20KOrchidbaits.git). Similarly, for the 56 *Catasetum* pollinaria, the total number of nuclear baits recovered was 12,836 (Table S4) with coverage ≥ 1, corresponding to an average of 10,497 nuclear baits at coverage ≥ 3. The captured loci in the pollinaria dataset corresponded closely to those captured in the *Catasetum* species (95.08%).

### 3.4 Construction of the Species‐Diagnostic SNP Matrix

Across the 22 *Catasetum* species, variant calling and filtering yielded 10,844 nuclear SNPs and 2619 plastid SNPs. Of these, 5219 nuclear SNPs and 1043 plastid SNPs met all the criteria to be defined as species‐specific and were retained in the species VCF file (Table S4).

**FIGURE 2:**
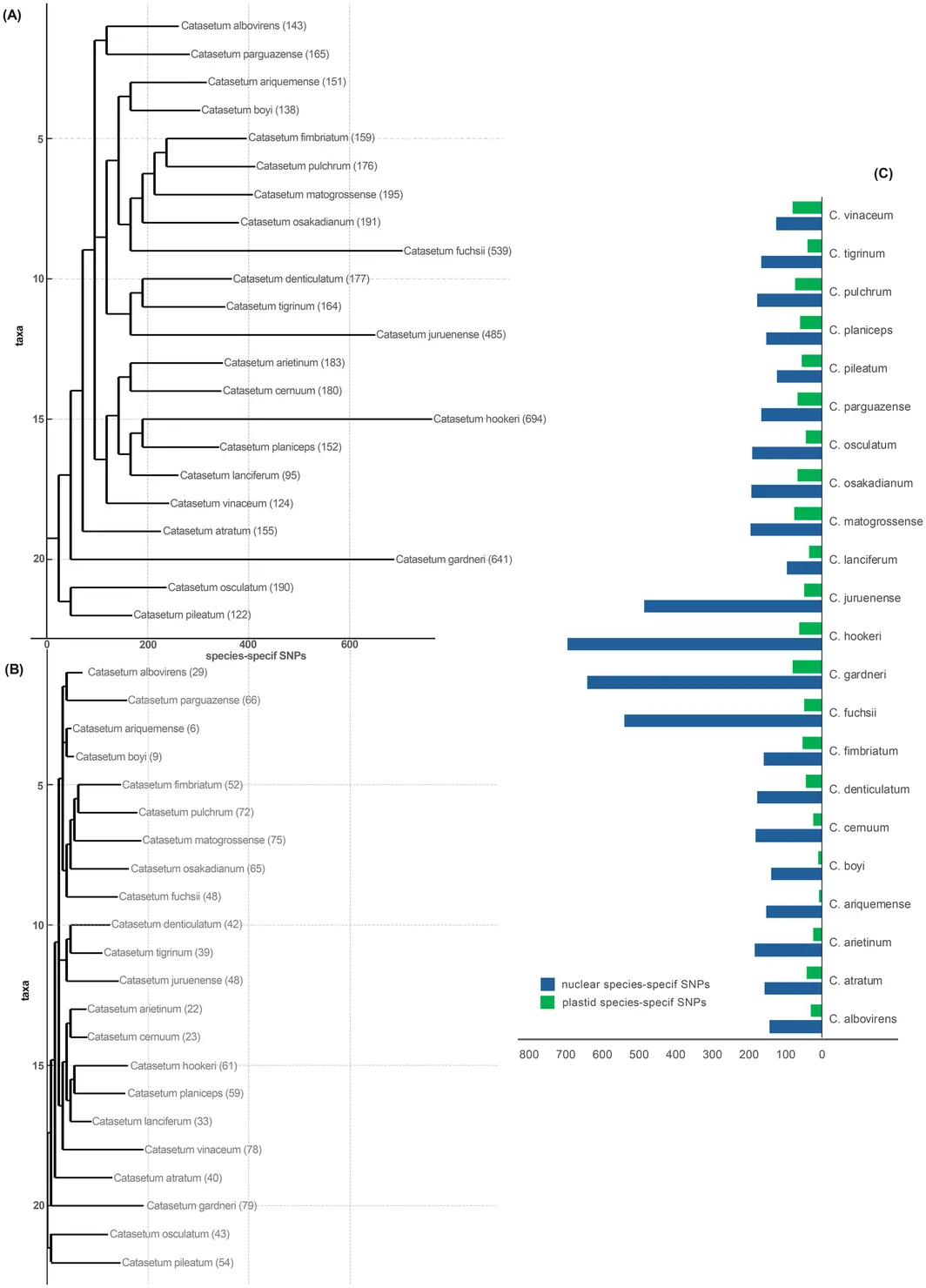
(A) Neighbour‐Joining (NJ) tree inferred from nuclear‐specific SNPs. (B) NJ tree based on plastid‐specific SNPs. (C) Number of nuclear and plastid species‐diagnostic SNPs recovered for each of the 22 *Catasetum* species.

The number of species‐specific nuclear SNPs varied widely across taxa, ranging from 95 in *C. lanciferum* to 694 in *C. hookeri* (Table S4; Figure 2C). *C. hookeri* , *C. gardneri* , and *C. fuchsii* represented the species with the largest number of diagnostic nuclear SNPs (≥ 500).

Plastid species‐specific SNPs showed a similar, though shallower, pattern of variation, ranging from 6 in *C. ariquemense* to 79 in *C. gardneri* (Table S4; Figure 2C).

**FIGURE 3:**
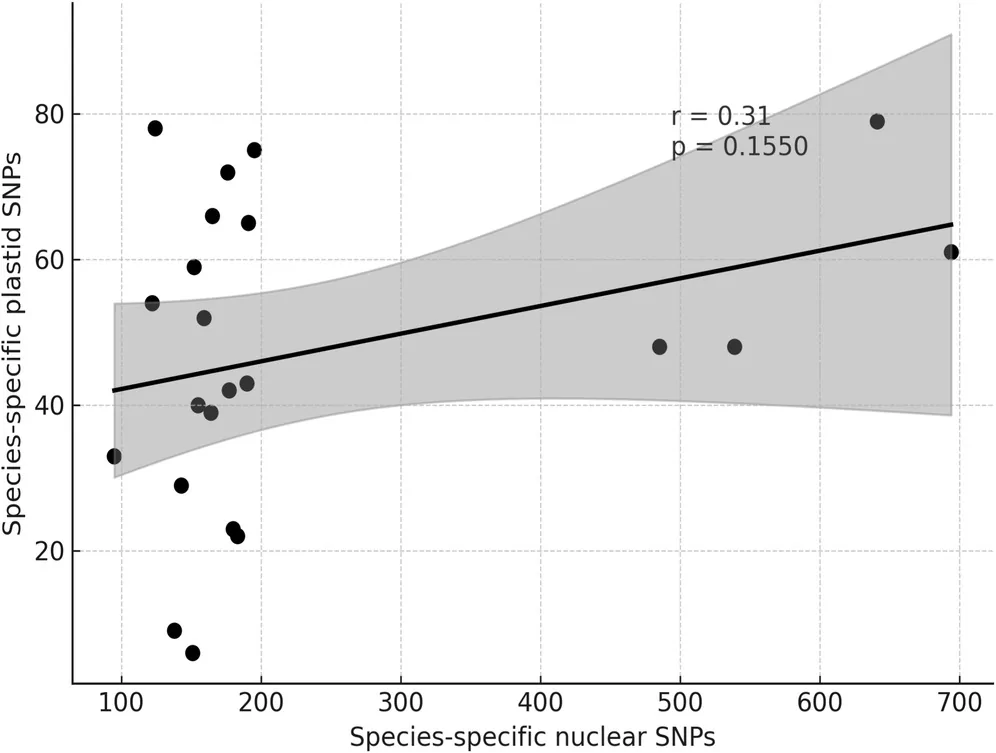
Correlation between nuclear and plastid species‐specific SNPs across the 22 *Catasetum* species.

Two species displayed a concordant number of species‐diagnostic SNPs across nuclear and plastid SNPs: *C. gardneri* showed high divergence with 542 nuclear and 79 plastid species‐diagnostic SNPs, while at the opposite end, *C. lanciferum* exhibited consistently low divergence, with only 95 nuclear and 14 plastid species‐diagnostic SNPs. In contrast, *C. ariquemense* showed moderate nuclear variation (241 species‐diagnostic SNPs) but extremely small plastid differentiation (only 6 species‐diagnostic SNPs) (Table S4; Figure 2C). However, for the full dataset, the number of species‐specific nuclear SNPs did not correlate with the number of species‐specific plastid SNPs per species (*r* = 0.31, *p* = 0.155; Figure 3).

The nuclear NJ tree (Figure 2A) reveals a well‐structured and highly resolved pattern of divergence among the 22 *Catasetum* species. Species characterized by large numbers of diagnostic SNPs, such as *C. hookeri, C. gardneri*, and *C. fuchsii* (≥ 500 fixed variants), display long and distinctive terminal branches, reflecting deep and unambiguous species separation. Conversely, species with fewer diagnostic SNPs, most notably *C. lanciferum*, occupy much shorter terminal branches. Despite this heterogeneity, all species form non‐overlapping clusters, with no evidence of ambiguous affinities or near‐collapsing nodes. The overall topology depicts a clear set of well‐separated lineages, each corresponding to a single *Catasetum* species.

The plastid NJ tree (Figure 2B) also recovers an equivalent set of 22 species‐level lineages, but with much shorter branches than the nuclear tree, resulting in a markedly compressed topology with shallow divergence.

The evaluation of the species‐specific VCF file across multiple accessions of the same species confirmed that conspecific individuals consistently clustered together (result not shown), yielding an average intraspecific divergence of 15 SNPs for the nuclear set and 2 SNPs for the plastid set (Appendix S1: Table S5). The highest intraspecific values were observed in *C. pulchrum* and *C. denticulatum* , whereas no polymorphic sites were detected between the two available accessions of *C. gardneri* . On average, this intraspecific genetic distance among conspecific individuals was comparable to that observed among conspecific pollinaria, which differed from their reference by 22.9 SNPs on average (range 6–60; Table S5).

### 3.5 Pollinarium Genotyping

From the 56 genotyped pollinaria, we recovered 8643 high‐quality nuclear SNPs.

**FIGURE 4:**
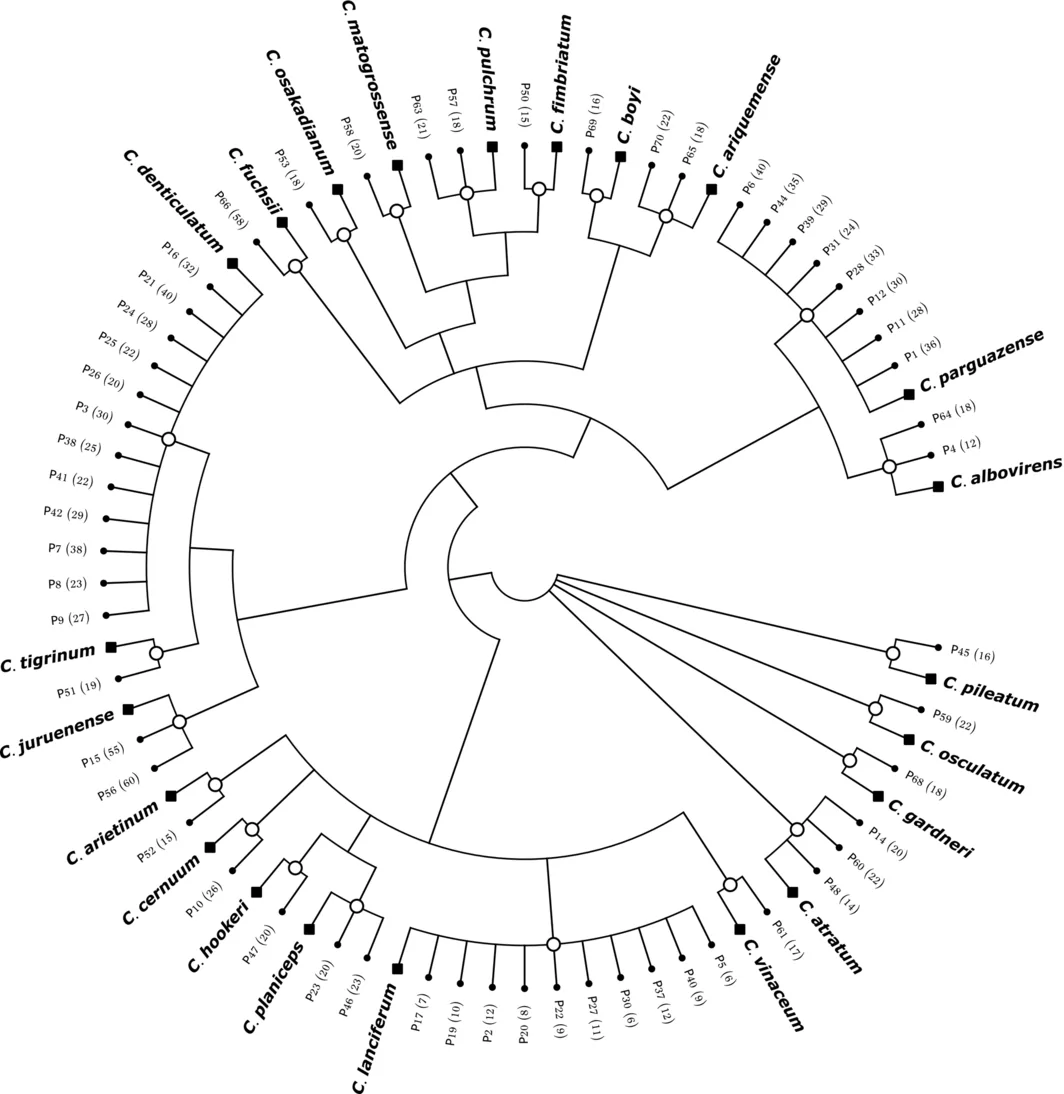
Maximum‐likelihood (ML) tree inferred in IQ‐TREE2 from the species‐specific nuclear SNPs under the GTR + F + ASC model, showing the genetic placement of the 56 pollinaria relative to their reference species in *Catasetum*. Branch support was assessed with two measures, each based on 1000 replicates: the ultrafast bootstrap approximation (UFBoot; Hoang et al. 2018) and the SH‐aLRT test; values are reported at the species clusters (open circles) as SH‐aLRT/UFBoot, with thresholds of SH‐aLRT ≥ 80% and UFBoot ≥ 95%. Reference species are indicated by their names (bold, filled squares) and the individual pollinaria by their sample IDs (P1–P70, dots); the number in parentheses next to each pollinarium indicates the number of SNPs by which it differs from its reference species (Appendix S1: Table S4).

Of these, the 5219 SNPs that matched the diagnostic positions defined in the species‐specific VCF file were retained in the combined multi‐sample VCF (Table S4). In the resulting ML tree, species form distinct clusters, and each pollinarium cluster with its reference species, confirming correct genetic assignment (Figure 4). Although all pollinaria clustered with their expected reference species, the metric tree highlighted variation in terminal branch lengths, reflecting some degrees of genomic divergence within species (Figure 4). The pollinarium–reference species distances were values that ranged from 6 to 12 SNPs in species with low intraspecific divergence such as *C. lanciferum* to 20–45 SNPs in more variable taxa (e.g., *C. denticulatum* , *C. parguazense*). A small subset of pollinaria showed substantially higher divergence: P66 ( *C. fuchsii* ), P15, and P56 (*C. juruenense*) differed from their reference (58, 55, and 60 SNPs), consistent with their longer terminal branches in Figure 4.

### 3.6 Evaluation of Assignment Accuracy Under Progressive SNPs Reduction

**FIGURE 5:**
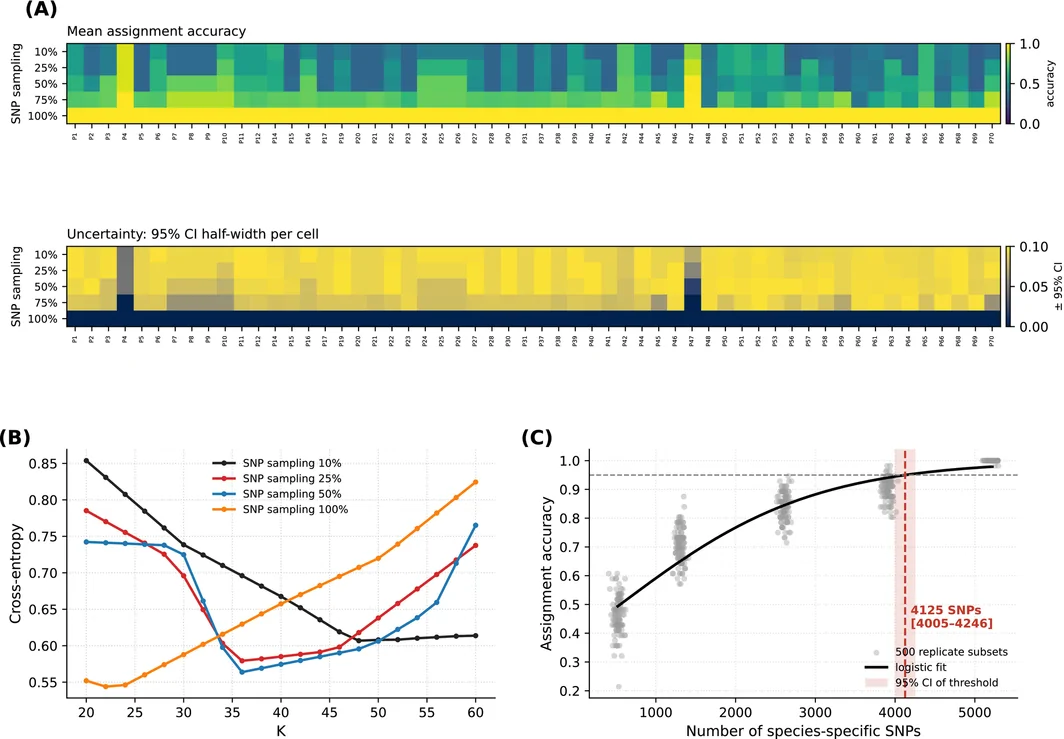
Effect of SNP resampling on pollinaria‐to‐species assignment. (A) *Top*: Heatmap of the mean assignment accuracy of each pollinarium (columns, P1–P70) across the five SNP‐resampling levels (rows: 100%, 75%, 50%, 25%, 10%), computed as the proportion of the replicate subsets per level in which the pollinarium was correctly assigned to its reference species (viridis scale, 0–1). *Bottom*: Uncertainty of each estimate, shown as the half‐width of the 95% confidence interval of the per‐cell mean (±1.96·√(p(1 − *p*)/100); cividis scale, 0–0.10). (B) Cross‐entropy profiles obtained with LEA across *K* = 20–60 for each SNP‐resampling level; lower values indicate the best‐supported number of genetic clusters (*K*). (C) Logistic regression of species‐assignment accuracy on SNP density, fitted to all individual replicate subsets; grey points are the individual subsets, the black curve is the fitted logistic model, the horizontal dashed line marks the 95% accuracy threshold, and the vertical dashed line with shaded band the minimum number of species‐specific SNPs required to reach it (4125 SNPs; 95% CI 4005–4246).

The resulting 500 ML trees gathered from the series of reduced SNP datasets (5219; 3914; 2610; 1305; and 522 SNPs, respectively) showed a progressive decline in all phylogenomic metrics. In particular, the assignment accuracy dropped at low SNP densities (Figure 5A), decreasing from 0.90 at 75% (3914 SNPs) to 0.84 at 50% (2610 SNPs), 0.70 at 25% (1305 SNPs), and reaching 0.45 in the 10% dataset (522 SNPs) where misassignment exceeded 50%. These trends were paralleled by corresponding reductions in treeness (from 0.84 to 0.42), saturation (from 0.92 to 0.62), and median bootstrap support (from 95 to 39) (Figure S1).

The SNP subsampling analysis revealed a strong dependency between assignment accuracy and genomic representation (Figure 5A). At high SNP densities (75–100%), all pollinaria were correctly assigned to their species, with accuracy, treeness, and bootstrap support values comparable to the full dataset (Figure S1). Assignment remained robust also at 50% SNP density, although a few samples, particularly from species with fewer diagnostic SNPs, displayed slightly reduced accuracy. At 25% SNP density, assignment performance declined more noticeably.

Pollinaria belonging to *C. lanciferum*, ithat is, the species characterised by the lowest number of diagnostic SNPs, showed the steepest loss of assignment accuracy across resampling replicates (Figure 5A; Figure S2). Additional pollinaria exhibiting early accuracy decay included P52 ( *C. arietinum* ), P69 (*C. boyi*), P66 ( *C. fuchsii* ), P63 ( *C. pulchrum* ), P64 ( *C. albovirens* ), P8 ( *C. denticulatum* ), and P39 (*C. parguazense*). These samples showed a progressive reduction in discriminatory signal when SNPs were removed, especially at 25% and 10% densities.

Conversely, a few pollinaria, most notably P4 ( *C. albovirens* ) and P47 ( *C. hookeri* ), maintained high assignment accuracy even at 10% SNP representation, reflecting the large number of highly distinctive SNPs available for their species (Figure 5A).

LEA cross‐entropy profiles confirmed these general patterns (Figure 5B). The full dataset showed a sharp minimum at *K* = 22, corresponding to the 22 analyzed species. In contrast, the 25% and 10% SNP matrices exhibited flattened entropy curves and inflated or unstable *K* values (*K* = 40–52), indicating fragmentation of genetic structure in low‐density datasets. The pollinaria responsible for this instability were the same that showed early assignment decay in Figure S2, first and foremost those of *C. lanciferum*, followed by the outliers listed above.

Finally, logistic regression between SNP number and assignment performance (Figure **5**C) estimated a threshold of 4125 species‐specific SNPs for ≥ 95% assignment accuracy, with a 95% confidence interval (CI) of 4005–4246 SNPs resulting from bootstrap resampling of replicates across the 22 *Catasetum* species.

## 4 Discussion

Our results demonstrate that the custom 20KOrchidbaits kit performs robustly across in silico, experimental, and ecological applications, underscoring its broad utility for Orchidaceae.

While earlier kits (e.g., Orchidinae‐205; Veltman et al. 2024) were restricted to nuclear markers, our design integrated nuclear and plastid baits, providing a unique opportunity to explore both genomic compartments by enabling the investigation of biparental and uniparental markers within a single sequencing run. This comprehensive approach allows for capturing both sides of the orchid genome, which was critical for detecting cytonuclear discordances arising from processes such as hybridization, introgression, or incomplete lineage sorting (ILS) (Duan et al. 2023; Rose et al. 2021; Lu 2025) (Figure 1; Tables S1 and S2). In a recent phylogenomic study of *Bromeliaceae*, leveraging both nuclear loci and plastome sequences in a target‐capture approach yielded a well‐resolved backbone phylogeny with only minor topological incongruences between genomes, highlighting the benefit of analyzing nuclear and organellar sequences (Bratzel et al. 2023).

The results from both in silico and experimental analysis confirm the efficiency and broad applicability of the 20KOrchidbaits set. The in silico evaluation revealed high capture breadth across all orchid subfamilies, with a few exceptions, such as *Vanilla planifolia* (7108) (Figure 1A; Table S1). The reduced efficiency observed in a few taxa is unlikely to be explained solely by phylogenetic distance, as the most basal orchid lineage (i.e., Apostasioideae) did not show poor recovery performance in our in silico tests (15,173–16,967 nuclear loci; 68%–93% of reads mapped). Instead, this sporadic low efficiency may reflect lineage‐specific genomic features, such as repetitive content and assembly completeness, which are known to influence in silico target‐capture performance independently of genomic divergence (Hale et al. 2020; Pérez‐Escobar et al. 2024). In particular, the *V. planifolia* genome is subject to partial endoreplication, which produces highly unbalanced DNA content and uneven sequencing depth, so that ~67% of the genes are concentrated in ~30% of the genome while the remaining ~33% lie in low‐coverage, repeat‐rich regions (Piet et al. 2022). Indeed, even after several attempts, the anchored published assembly of *V. planifolia* represents only ~83% of the genome (Piet et al. 2022).

In silico screenings of orchid plastomes revealed overall high recovery across most lineages included in our bait design. However, *C. japonicum* showed markedly reduced coverage (Figure 1C; Table S2) and represented a clear outlier. This result was consistent with previous studies reporting extensive plastome rearrangements and accelerated sequence evolution in the genus. Indeed, the *Cypripedium* plastome is markedly expanded (up to ~212 kb, the largest in orchids) through proliferation of AT‐biased repeats, with record‐low GC content (~28%–30%) and low gene density (0.62–0.75) (Guo et al. 2025; Lu 2025). Because our plastid baits were selected within a 20%–50% GC window from more typical GC‐richer orchid plastomes, they mapped less efficiently to this AT‐rich, repeat‐expanded plastome, lowering in silico recovery for this species. The observed signal drop for *C. japonicum* thus reflects a genuine biological divergence rather than a technical artefact and highlights the limitations of universal bait design when applied to highly derived plastome lineages.

The in silico evaluation revealed a universal core of 6682 nuclear and 300 plastid loci common to all subfamilies (dataset available at https://github.com/terryr92/20KOrchidbaits.git), despite the genomic heterogeneity of Orchidaceae. Considering a 50% tiling overlap, these 300 plastid loci cover ~15–30 kb, that is, ~10%–20% of an average ~150 kb orchid plastome.

The experimental application of the kit consistently recovered high numbers of nuclear and plastid loci in *Catasetum*, closely matching the in silico analysis and further confirming the predictive reliability of the bait‐design filtering pipeline (Kadlec et al. 2017). In particular, the proportion (5.37%) of *Catasetum* plastid reads we recovered from the total output of a dual‐compartment capture from whole‐genomic DNA (nuclear and plastidial) aligns with the expected theoretical outcome. This proportion was constrained by our bait design: only 400 of the 20,400 baits (1.96%) target the plastome. Despite the small plastid read fraction, we recovered 377 of the 400 plastid baits (94.2%) in *Catasetum*, closely matching the in silico prediction (85.9%, 365 loci).

Almost the same recovered baits in plant DNAs of *Catasetum* were also recovered in the pollinaria hybridization experiment. In our specific case study, the pollinaria DNAs yielded even higher nuclear locus recovery than leaf‐derived DNAs (Table S4). In hybrid‐capture applications, incomplete recovery of predicted loci was expected and reflected the combined influence of DNA quality, technical conditions, and stochastic variation inherent to competition among probes during hybridization, library preparation, and sequencing (Staats et al. 2013; Jones and Good 2016; Bi et al. 2012). However, in our case, we interpret the difference in yield as due to the sequencing platforms used to generate the datasets (Illumina NovaSeq for pollinaria and MiSeq for leaf samples), which differ in capture breadth and coverage depth (Han et al. 2024).

The comparative analysis of nuclear and plastid SNP datasets reveals marked differences in their ability to resolve species boundaries in *Catasetum*, as well as complementary insights into the evolutionary processes shaping the group (Figure 3). Despite the overall reduction in signal, the plastid NJ tree does not contradict the nuclear NJ tree reconstruction: species remain distinct, and their relative positions remain broadly consistent. However, the plastid tree exhibits substantially shorter terminal branches and a markedly compressed topology, reflecting the reduced number of plastid SNPs recovered for most species (Figure 2B).

The comparison between nuclear and plastid SNP divergence (Figure 2A,B) indicates that species with higher numbers of species‐specific nuclear SNPs do not always also exhibit higher plastid variation. Indeed, the correlation between nuclear and plastid species‐specific SNPs was weak and non‐significant (Figure 3), *which may suggest some decoupling between nuclear and plastid divergence, although this is not statistically supported and should be interpreted with caution*. Species with many nuclear diagnostic SNPs did not necessarily show high plastid divergence, and vice versa. This pattern was consistent with the different evolutionary dynamics of nuclear and plastid genomes and highlighted why integrating nuclear and plastid SNPs provided a more complete representation of species differentiation and divergence time. Contrasting patterns of nuclear and plastid divergence may be consistent with past introgression or plastid capture events that mask the true extent of genomic divergence (Pérez‐Escobar et al. 2024). This evidence reinforces the utility of dual‐marker kits like 20KOrchidbaits for capturing complementary evolutionary signals in orchid lineages undergoing hybridization or lineage sorting (Duan et al. 2023; Rose et al. 2021).

The 20KOrchidbaits kit proved highly effective for resolving relationships among closely related *Catasetum* species, even after excluding the heterozygous sites that can potentially introduce background noise into the species delimitation. Indeed, by validating the panel performance on genetically diverse field individuals (both plants and pollinaria), we confirmed that non‐polymorphic (species‐specific) variation was sufficient to delimit *Catasetum* species.

The large number of informative nuclear SNPs recovered indicates that the panel can successfully discriminate species even when genomic divergence was shallow, providing a reliable framework for sample identification and assignment. Such capacity is particularly valuable in contexts requiring authentication, biodiversity monitoring, and commercial traceability. For instance, Anderson et al. (2023) recently developed a SNP genotyping panel to distinguish economically important V*anilla* species (orchids used for vanilla flavoring) and their hybrids from small tissue samples, including processed vanilla beans.

The application of nuclear baits proved especially valuable for species identification in ecological settings, particularly within plant–pollinator interaction studies. Direct observations of orchid pollination events were notoriously difficult, especially of tropical orchids. This lack of direct observations greatly limits our understanding of orchid–pollinator relationships and particularly the extent of pollinator‐specificity, making it very important to increase indirect observations of orchid pollination through pollinarium genotyping from bees collected in the wild or from museum collections (Widmer et al. 2000).

Unlike traditional barcodes for pollen identification, such as Internal Transcribed Spacer (ITS), which often lack sufficient interspecific variability in rapidly radiating orchid groups (Widmer et al. 2000), the genome‐wide nuclear SNP signal obtained with the 20KOrchidbaits kit reliably assigned pollinaria to their reference species, including closely related taxa (Figure 4). This capacity is essential for directly linking the genomic distinctiveness of each species (Figure 2) to documented ecological interactions, as revealed by pollinarium genotyping.

In our dataset, the individuals used as references were not the same plants from which the pollinaria were collected. The strong discriminatory power of nuclear SNPs was directly reflected in the clustering patterns observed in the NJ tree, where each pollinarium consistently grouped with the correct species, despite measurable intraspecific divergence (Figure 4). Most pollinaria showed only limited divergence from their respective reference individuals, while a few exhibited higher values. These cases correspond to the longer terminal branches observed in the NJ tree and are likely to reflect the high number of species‐specific SNPs typical of each species (Figure 4). Nevertheless, the number of SNPs differentiating each pollinarium from its reference species (Table S4) remains well below the number of diagnostic SNPs defined in the species‐specific matrix (Morin et al. 2004; Ellegren 2014) (Table S4). These variants, therefore, represent normal intraspecific polymorphisms that happened to be captured in the pollinium but were absent in the specific reference individuals sequenced by Milet‐Pinheiro et al. (in prep.) and reflect the natural genetic variability expected within species. These findings underscore the reliability of nuclear SNP profiles in maintaining species‐level resolution even when applied to non‐identical individuals, a key requirement for ecological and forensic applications where samples often come from unknown or non‐reference tissues (Andermann et al. 2020; Anderson et al. 2023).

Our SNP subsampling procedure demonstrated that assignment reliability was, as expected, tightly linked to genomic representation (Figure 5; Figure S1). Matrices retaining a high number of species‐diagnostic SNPs, that is, high‐density SNP matrices, consistently preserved clear species boundaries, whereas assignment success declined sharply as SNP number decreased. Concordantly, assignment accuracy, treeness, and bootstrap support all declined smoothly and proportionally with decreasing SNP number (Figure 5A; Figure S1).

A few pollinaria, most clearly P4 (*C. albovirans*) and P47 (*C. hookeri*), maintained high assignment accuracy even under the most severe SNP resampling (10%). These pollinaria belong to species with highly distinctive diagnostic SNPs: even when only a small fraction of sites was retained in the resampling routine, the probability of capturing at least some of these diagnostic markers remains high. In contrast, for species with a smaller number of diagnostic markers, such as *C. lanciferum* (only 95 species‐specific SNPs), the assignment accuracy of pollinaria drops rapidly when SNPs are removed (Figure 5A; Figure S2). Accordingly, in the resampling tests, the correspondence between pollinaria and *C. lanciferum* declined more quickly than for any other species. Thus, an adequate SNP representation is important for ensuring both species delimitation and intraspecific traceability, particularly in ecological or forensic applications where ambiguous matches must be avoided even between closely related species. Finally, pollinaria that were genetically closer to the reference individuals remain stable under progressive SNP depletion, whereas those carrying more private variants lose support earlier (Figure 5A).

The behaviour of clustering algorithms under increasingly sparse data further reinforces this conclusion. LEA outputs showed inflation and instability of K when SNP density was reduced, an expected outcome when noise exceeds the biological signal (Figure 5B). This artefactual partitioning fits within broader population‐genomic theory: datasets with few loci tend to overestimate population subdivision and create false genetic clusters due to stochastic sampling of alleles (Janes et al. 2017; O'Leary et al. 2018).

Phylogenetically, the contrast between high‐ and low‐density matrices emphasizes that deep and stable topologies require broad genome sampling (Figure S1). Trees inferred from full datasets were reproducible across replicates and retained internal resolution, whereas trees inferred from reduced datasets compressed branch lengths and eroded bootstrap support. Our results empirically defined a minimum threshold of 4125 SNPs (95% CI 4005–4246) required to maintain ≥ 95% assignment accuracy among the 22 *Catasetum* species (Figure 5C). These diagnostic SNPs are spread across the nuclear bait set at an average of 1 SNP per bait captured in *Catasetum* (13,500 baits recovered at coverage ≥ 1 across the 22 species). This corresponds to fewer than one diagnostic variant per 100‐bp bait, indicating that the diagnostic signal is distributed genome‐wide across the captured loci rather than concentrated in a few baits.

In conclusion, our findings underscore that genomic markers are not only a powerful tool for species identification but also provide empirical benchmarks to guide future applications that would be relevant for uncovering the high diversity of ecological interactions in the neotropics.

The application of the 20KOrchidbaits to pollinaria identification represents a proof of concept that SNP panels can potentially be used to identify unknown orchid species from their pollinaria, even if further work is needed to make the SNP panel independent from the addition/removal of reference species (for instance, by including AI‐based species attribution).

For example, through the analysis of pollinaria preserved on insect specimens housed in museum collections, we can bridge critical knowledge gaps concerning neotropical orchid pollinators, whose information is often scarce owing to their epiphytic lifestyle, sparse distribution, and presence in challenging, remote localities (Pessoa et al. 2025). Resolving the critical paucity of orchid pollinator data is paramount to elucidating macroevolutionary questions, specifically how pollinators act as selective agents driving the evolution of floral traits, such as morphology, color, and scent (van der Niet 2021).

The broad phylogenetic versatility and high discriminatory power of the 20KOrchidbaits panel make it a scalable and field‐ready tool for ecological and evolutionary studies across *Orchidaceae*. As plant ecology moves deeper into the genomic era, such optimized resources can help bridge the gap between lab‐based genomic advances and real‐world biodiversity challenges.

## Funding

This work was supported by the Deutsche Forschungsgemeinschaft (DFG: AY 12/12‐1; AY 12/12‐3), the Fundaçao de Apoio ao Desenvolvimento da Universidade Federal de Pernambuco (FADE‐UFPE), and the Conselho Nacional de Desenvolvimento Científico e Tecnológico (CNPq/Universal Proc.n. 422647/2021‐7 to P.M.P). Grants were provided by the Conselho Nacional de Desenvolvimento Científico e Tecnolóogico to P.M.P (CNPq Proc.n. 313948/2021‐6). S.C. was funded by the National Recovery and Resilience Plan (NRRP), Mission 4, Component 2, Investment 1.1, Call for tender No. 104 published on 2.2.2022 by the Italian Ministry of University and Research (MUR), funded by the European Union—NextGenerationEU—PRIN 2022 (project number 2022W52K49). D.C. was funded by project FRA‐HYSENSING, financially supported by the University of Naples Federico II.

## Conflicts of Interest

The authors declare no conflicts of interest.

## Supporting information


**Figure S1:** Effect of SNP subsampling on phylogenomic metrics. (A) Median ultrafast bootstrap support, (B) Treeness (t) values, and (C) Saturation index (*R* ^2^). Black bars = IQR; orange dots = median.
**Figure S2:** The clustering assignment profiles for C. lanciferum under SNP resampling. Ancestry proportions for each pollinarium (P2, P5, P17, P19, P20, P22, P27, P30, P37, P40) per C. lanciferum and OTHER across SNP resampling (100%, 75%, 50%, 25%, 10%).
**Table S1:** In silico evaluation of nuclear bait recovery across Orchidaceae. For each species, we reported taxonomic affiliation, availability of genomic or transcriptomic resources, NCBI accession numbers, numbers of simulated paired‐end reads, mapping efficiency, and the number of nuclear baits. The nuclear bait recovery was summarized as mean, median, interquartile range (IQR), and range. Mean nuclear mapping percentages were reported for each subfamily. A, Apostasioideae; C, Cypripedioideae; E, Epidendroideae; O, Orchidoideae; V, Vanilloideae.
**Table S2:** In silico evaluation of plastid bait recovery across Orchidaceae. For each species, we reported taxonomic affiliation, NCBI accession numbers, numbers of simulated paired‐end reads, mapping efficiency, and the number of plastid baits. The plastid baits recovery was summarized as mean, median, interquartile range (IQR), and range. Mean plastid mapping percentages were reported for each subfamily. A, Apostasioideae; C, Cypripedioideae; E, Epidendroideae; O, Orchidoideae; V, Vanilloideae.
**Table S3:** Distribution of the 20,000 nuclear baits across the five Orchidaceae subfamilies. For each bait (rows), 1/0 indicates recovery or absence in Apostasioideae (A; *n* = 16,070), Cypripedioideae (C; *n* = 8390), Epidendroideae (E; *n* = 12,546), Orchidoideae (O; *n* = 15,903) and Vanilloideae (V; *n* = 7108); the N_groups column reports the number of subfamilies in which the bait was recovered. The 6682 baits with N_groups = 5 represent the set shared across all major clades.
**Table S4:** Sequencing statistics, SNP recovery, and pollinarium–reference divergence for *Catasetum*. The table reported sequencing and mapping statistics for both reference species and 56 pollinaria samples. For each pollinarium, we provided raw and trimmed read counts, mapping efficiency, nuclear bait recovery, total nuclear SNPs, the subset of species‐specific diagnostic SNPs used for assignment, and the number of SNPs differing from the corresponding reference species. Reference‐level capture statistics (nuclear and plastid mapped reads and baits recovered) were reported for each species and were repeated across conspecific pollinarium to facilitate direct comparison between intraspecific divergence and species‐level genomic representation. The species‐specific nuclear and plastid SNPs were defined from the reference dataset. In contrast, pollinarium‐specific SNP differences represented private or low‐frequency variants that were not present in the sequenced reference individuals.
**Table S5:** Intraspecific polymorphism of species‐specific diagnostic SNPs.

## Data Availability

The list of recovered loci and the universal core dataset are available at GitHub (https://github.com/terryr92/20KOrchidbaits.git). All custom scripts used in this study are publicly available at GitHub (https://github.com/terryr92/20KOrchidbaits.git). Raw sequencing data supporting the findings of this study have been deposited in a public repository (NCBI SRA, BioProject ID: PRJNA150482) and will be available upon acceptance of the manuscript.
