# Supplementary material for: Tracing Species Boundaries Through Genomic Baits: Diagnostic SNP Panels for Genotyping Orchid Pollinaria
Source: Evol Appl. 2026 Aug 24;19(8):e70315. doi: 10.1111/eva.70315 (PMC13501508; doi:10.1111/eva.70315)
Supplement: Supplementary file 1 [file EVA-19-e70315-s001.docx]

**Appendix S1**

**Tracing species boundaries through genomic baits: Diagnostic SNP panels for genotyping orchid pollinaria**

**Supplementary Figures**

**Fig. S1.** Effect of SNP subsampling on phylogenomic metrics. (A) Median ultrafast bootstrap support, (B) Treeness (t) values, and (C) Saturation index (R²). Black bars = IQR; orange dots = median.

**Fig. S2.** The clustering assignment profiles for C. lanciferum under SNP resampling. Ancestry proportions for each pollinarium (P2, P5, P17, P19, P20, P22, P27, P30, P37, P40) per C. lanciferum and OTHER across SNP resampling (100%, 75%, 50%, 25%, 10%).

**Supplementary Tables**

| **Species** | **Subfamily** | **Available genome or transcriptome** | **NCBI accession number** | ***In silico* number raw reads (PE)** | ***In silico* number mapped reads (PE)** | **% *in silico* mapped reads** | **Number of nuclear baits with coverage >3** | **Number of nuclear baits per subfamily (mean; median; IQR; range)** | **% in silico mapped reads per subfamily** |  |
| --- | --- | --- | --- | --- | --- | --- | --- | --- | --- | --- |
|  |  |  |  |  |  |  |  |  |  |  |
| *Apostasia ramifera* | A | only genome | GCA_019297695.1 | 26584529 | 24817601 | 93.35 | 15173 | 16070 ( 16070; 15621.5–16518.5; 15173.0–16967.0) | 80.71 |  |
| *Apostasia shenzhenica* | A | only genome | GCA_002786265.1 | 38258670 | 26043885 | 68.07 | 16967 |  |  |  |
| *Cypripedium macranthos var. rebunense* | C | only transcriptome | ICTD00000000.1 | 4630708 | 4288344 | 92.61 | 8390 | 8390 ( 8390; 8390.0–8390.0; 8390.0–8390.0) | 92.61 |  |
| *Bletilla sinensis* | E | only genome | GCA_022495875.1 | 39049111 | 26431281 | 67.69 | 10273 | 12546.26 (11832; 10001.5–14884.0; 7227.0–17385.0) | 69.01 |  |
| *Bletilla striata* | E | only transcriptome | GGUU00000000.1 | 46556780 | 29167184 | 62.65 | 17385 |  |  |  |
| *Calanthe flava* | E | only transcriptome | GJQF00000000.1 | 3285160 | 2810332 | 85.55 | 14832 |  |  |  |
| *Cymbidium faberi* | E | only transcriptome | GDHD01000001 | 24857742 | 19987029 | 80.41 | 16446 |  |  |  |
| *Cymbidium sinense* | E | only genome | GCA_021442155.1 | 40727468 | 26874159 | 65.99 | 11832 |  |  |  |
| *Dendrobium catenatum* | E | both | GCA001605985.2 | 20489840 | 11469010 | 55.97 | 14936 |  |  |  |
|  | E |  | GEZV01000001 | 12788678 | 8411055 | 65.77 | 14778 |  |  |  |
| *Dendrobium chrysotoxum* | E | only genome | GCA_019925795.1 | 8556168 | 6628854 | 77.47 | 12017 |  |  |  |
| *Dendrobium huoshanense* | E | only genome | GCA_016618105.1 | 30431703 | 15985449 | 52.53 | 11735 |  |  |  |
| *Dendrobium nobile* | E | only genome | GCA_022539455.1 | 20281600 | 15433695 | 76.10 | 11799 |  |  |  |
| *Epipogium aphyllum* | E | only transcriptome | GFWK00000000.1 | 25717335 | 24515099 | 95.33 | 9636 |  |  |  |
| *Epipogium roseum* | E | only transcriptome | GFWL00000000.1 | 14719242 | 10690689 | 72.63 | 9879 |  |  |  |
| *Gastrodia elata* | E | only genome | GCA_016760335.1 | 18030288 | 12271559 | 68.06 | 9937 |  |  |  |
| *Oberonia myosurus* | E | only transcriptome | GISE00000000.1 | 37291527 | 19520134 | 52.34 | 9769 |  |  |  |
| *Oncidium hybrid cultivar* | E | only transcriptome | GIQW00000000.1 | 46054178 | 28681326 | 62.28 | 16096 |  |  |  |
| *Papilionanthe hookeriana x Papilionanthe teres* | E | only genome | GCA_022702705.1 | 40334882 | 32429047 | 80.40 | 10066 |  |  |  |
| *Phalaenopsis aphrodite* | E | both | GCA_003013225.1 | 26246560 | 14096828 | 53.71 | 14714 |  |  |  |
|  | E |  | GENW00000000.1 | 21009001 | 13848514 | 65.92 | 15022 |  |  |  |
| *Vanda hybrid cultivar Mimi Palmer* | E | only transcriptome | GGYD00000000.1 | 46891129 | 33047647 | 70.48 | 7227 |  |  |  |
| *Anoectochilus roxburghii* | O | only transcriptome | GETU00000000.1 | 29891752 | 21162080 | 70.80 | 12462 | 15902.67 (16209; 14289.5–16824.0; 11999.0–19555.0) | 74.4 |  |
| *Chiloglottis trapeziformis* | O | only transcriptome | GFPK00000000.1 | 39407771 | 22662919 | 57.51 | 15040 |  |  |  |
| *Dactylorhiza fuchsii* | O | only transcriptome | GFHX00000000.1 | 18013446 | 13581016 | 75.39 | 15964 |  |  |  |
| *Dactylorhiza incarnata* | O | both | GCA_033032865.1 | 29009224 | 21211583 | 73.12 | 12290 |  |  |  |
|  |  |  | GFJV00000000.1 | 13910049 | 6998609 | 50.31 | 15002 |  |  |  |
| *Glossodia major* | O | only transcriptome | GKRN00000000.1 | 9130144 | 7622285 | 83.48 | 11999 |  |  |  |
| *Gymnadenia conopsea* | O | only transcriptome | GHXG00000000.1 | 6545583 | 5812239 | 88.80 | 16209 |  |  |  |
| *Gymnadenia odoratissima* | O | only transcriptome | GHXF00000000.1 | 43542436 | 38657990 | 88.78 | 16674 |  |  |  |
| *Gymnadenia rhellicani* | O | only transcriptome | GHXH00000000.1 | 48626914 | 42666230 | 87.74 | 16839 |  |  |  |
| *Gymnadenia x densiflora* | O | only transcriptome | GHXE00000000.1 | 41070421 | 35179205 | 85.66 | 16729 |  |  |  |
| *Ophrys insectifera subsp. Aymoninii* | O | only transcriptome | GHWW00000000.1 | 16881728 | 16747325 | 99.20 | 19555 |  |  |  |
| *Ophrys insectifera* | O | only transcriptome | GHWX00000000.1 | 36045666 | 20031854 | 55.57 | 19488 |  |  |  |
| *Ophrys iricolor* | O | only transcriptome | GHXI00000000.1 | 45891300 | 24147132 | 52.62 | 19430 |  |  |  |
| *Orchis italica* | O | only transcriptome | - | 14722094 | 7903382 | 53.68 | 16809 |  |  |  |
| *Platanthera zijinensis* | O | only genome | - | 45464141 | 34554655 | 76.00 | 13539 |  |  |  |
| *Platanthera guangdongensis* | O | only genome | - | 24178434 | 16359287 | 67.66 | 15513 |  |  |  |
| *Vanilla planifolia* | V | only genome | GCA_023846275.1 | 48321408 | 47638299 | 98.59 | 7108 | 7108 ( 7108; 7108.0–7108.0; 7108.0–7108.0) | 98.59 |  |

**Suppl. Table 1.** In silico evaluation of nuclear bait recovery across Orchidaceae. For each species, we reported taxonomic affiliation, availability of genomic or transcriptomic resources, NCBI accession numbers, numbers of simulated paired-end reads, mapping efficiency, and the number of nuclear baits. The nuclear bait recovery was summarized as mean, median, interquartile range (IQR), and range. Mean nuclear mapping percentages were reported for each subfamily. A=Apostasioideae; C=Cypripedioideae; E=Epidendroideae; O=Orchidoideae;V=Vanilloideae.

| **Species** | **Subfamily** | **NCBI accession number** | ***In silico* number raw reads (PE)** | ***In silico* number mapped reads (PE)** | **% *in silico* mapped reads** | **Number of plastid baits with coverage >3** | **Number of plastid baits per subfamily (mean; median; IQR; range)** | **% *in silico* mapped reads per subfamily** |  |
| --- | --- | --- | --- | --- | --- | --- | --- | --- | --- |
|  |  |  |  |  |  |  |  |  |  |
| *Apostasia odorata* | A | KM244734.1 | 12227 | 9819 | 80.31 | 325 | 324 (324; 323.75–324.25; 323.0–325.0) | 83.02 |  |
| *Apostasia ramifera* | A | MT864006.1 | 25014 | 17203 | 68.77 | 324 |  |  |  |
| *Apostasia shenzhenica* | A | MG772639.1 | 15441 | 14479 | 93.77 | 324 |  |  |  |
| *Apostasia wallichii* | A | LC199394.1 | 28934 | 25816 | 89.22 | 323 |  |  |  |
| *Cypripedium calceolus* | C | MN602053.1 | 33256 | 29714 | 89.35 | 323 | 328.42 (327; 326.0–328.0; 288.0–389.0) | 90.17 |  |
| *Cypripedium debile* | C | MW924111.1 | 95433 | 90938 | 95.29 | 341 |  |  |  |
| *Cypripedium farreri* | C | OM066273.1 | 50021 | 44954 | 89.87 | 342 |  |  |  |
| *Cypripedium fasciolatum* | C | OM066274.1 | 27917 | 21678 | 77.65 | 338 |  |  |  |
| *Cypripedium flavum* | C | OM066275.1 | 27763 | 25711 | 92.61 | 341 |  |  |  |
| *Cypripedium formosanum* | C | KJ501998.1 | 79629 | 60207 | 75.61 | 340 |  |  |  |
| *Cypripedium franchetii* | C | OM066277.1 | 79825 | 63237 | 79.22 | 342 |  |  |  |
| *Cypripedium henryi* | C | OM066279.1 | 62524 | 47031 | 75.22 | 341 |  |  |  |
| *Cypripedium japonicum* | C | OM066280.1 | 62514 | 51612 | 82.56 | 288 |  |  |  |
| *Cypripedium macranthos* | C | KF925434.1 | 19689 | 17606 | 89.42 | 340 |  |  |  |
| *Cypripedium maoxianense* | C | OM066283.1 | 97085 | 76348 | 78.64 | 339 |  |  |  |
| *Cypripedium tibeticum* | C | MN561380.1 | 26321 | 20996 | 79.77 | 340 |  |  |  |
| *Cypripedium x ventricosum* | C | OM066286.1 | 42637 | 38216 | 89.63 | 340 |  |  |  |
| *Cypripedium yunnanense* | C | MW489465.1 | 78306 | 60938 | 77.82 | 341 |  |  |  |
| *Mexipedium xerophyticum* | C | NC_069868.1 | 46911 | 42731 | 91.09 | 323 |  |  |  |
| *Paphiopedilum acmodontum* | C | NC_069650.1 | 49983 | 42406 | 84.84 | 325 |  |  |  |
| *Paphiopedilum agusii* | C | NC_069954.1 | 49862 | 47279 | 94.82 | 326 |  |  |  |
| *Paphiopedilum appletonianum* | C | MZ751073.1 | 49692 | 42159 | 84.84 | 325 |  |  |  |
| *Paphiopedilum argus* | C | NC_069956.1 | 49828 | 43749 | 87.80 | 330 |  |  |  |
| *Paphiopedilum armeniacum* | C | KJ566307.1 | 50227 | 42613 | 84.84 | 325 |  |  |  |
| *Paphiopedilum barbatum* | C | NC_069957.1 | 50616 | 43904 | 86.74 | 326 |  |  |  |
| *Paphiopedilum barbigerum* | C | MN153814.1 | 50491 | 44331 | 87.80 | 324 |  |  |  |
| *Paphiopedilum bellatulum* | C | MN315107.1 | 50779 | 46945 | 92.45 | 326 |  |  |  |
| *Paphiopedilum braemii* | C | NC_069958.1 | 50261 | 48386 | 96.27 | 327 |  |  |  |
| *Paphiopedilum bullenianum* | C | NC_069959.1 | 50139 | 48269 | 96.27 | 327 |  |  |  |
| *Paphiopedilum callosum* | C | NC_069960.1 | 50379 | 48500 | 96.27 | 324 |  |  |  |
| *Paphiopedilum canhii* | C | NC_069961.1 | 22900 | 22609 | 98.73 | 326 |  |  |  |
| *Paphiopedilum charlesworthii* | C | NC_060650.1 | 50512 | 42854 | 84.84 | 326 |  |  |  |
| *Paphiopedilum ciliolare* | C | NC_069962.1 | 50643 | 46819 | 92.45 | 327 |  |  |  |
| *Paphiopedilum coccineum* | C | NC_069963.1 | 50365 | 48486 | 96.27 | 330 |  |  |  |
| *Paphiopedilum concolor* | C | OQ981989.1 | 50692 | 49115 | 96.89 | 325 |  |  |  |
| *Paphiopedilum dayanum* | C | NC_069965.1 | 50662 | 44481 | 87.80 | 330 |  |  |  |
| *Paphiopedilum delenatii* | C | MK463585.1 | 50551 | 49065 | 97.06 | 328 |  |  |  |
| *Paphiopedilum dianthum* | C | MF983795.1 | 50243 | 43581 | 86.74 | 328 |  |  |  |
| *Paphiopedilum druryi* | C | NC_069966.1 | 50316 | 43644 | 86.74 | 327 |  |  |  |
| *Paphiopedilum emersonii* | C | MT648789.1 | 35042 | 30045 | 85.74 | 327 |  |  |  |
| *Paphiopedilum exul* | C | NC_069967.1 | 50043 | 48176 | 96.27 | 327 |  |  |  |
| *Paphiopedilum fairrieanum* | C | NC_069968.1 | 49939 | 42368 | 84.84 | 327 |  |  |  |
| *Paphiopedilum fowliei* | C | NC_069970.1 | 49946 | 43323 | 86.74 | 326 |  |  |  |
| *Paphiopedilum gigantifolium* | C | NC_069971.1 | 49666 | 43080 | 86.74 | 325 |  |  |  |
| *Paphiopedilum glanduliferum* | C | NC_069972.1 | 49309 | 43293 | 87.80 | 328 |  |  |  |
| *Paphiopedilum glaucophyllum* | C | NC_069973.1 | 49319 | 42779 | 86.74 | 328 |  |  |  |
| *Paphiopedilum godefroyae* | C | NC_069974.1 | 49691 | 47838 | 96.27 | 326 |  |  |  |
| *Paphiopedilum gratrixianum* | C | MW284890.1 | 49698 | 47645 | 95.87 | 327 |  |  |  |
| *Paphiopedilum hangianum* | C | NC_069869.1 | 49921 | 43301 | 86.74 | 328 |  |  |  |
| *Paphiopedilum haynaldianum* | C | NC_069870.1 | 49923 | 48061 | 96.27 | 326 |  |  |  |
| *Paphiopedilum helenae* | C | NC_064145.1 | 50087 | 43445 | 86.74 | 325 |  |  |  |
| *Paphiopedilum hennisianum* | C | NC_069871.1 | 49809 | 43732 | 87.80 | 326 |  |  |  |
| *Paphiopedilum henryanum* | C | MN315108.1 | 49913 | 43295 | 86.74 | 322 |  |  |  |
| *Paphiopedilum hirsutissimum* | C | MN153815.1 | 50076 | 43436 | 86.74 | 326 |  |  |  |
| *Paphiopedilum hookerae* | C | NC_069873.1 | 50148 | 48277 | 96.27 | 327 |  |  |  |
| *Paphiopedilum insigne* | C | NC_069874.1 | 29317 | 28455 | 97.06 | 326 |  |  |  |
| *Paphiopedilum intaniae* | C | NC_069875.1 | 13024 | 12538 | 96.27 | 326 |  |  |  |
| *Paphiopedilum jackii* | C | NC_069882.1 | 46279 | 40142 | 86.74 | 325 |  |  |  |
| *Paphiopedilum javanicum* | C | NC_069876.1 | 46286 | 40148 | 86.74 | 327 |  |  |  |
| *Paphiopedilum kolopakingii* | C | MW794132.1 | 25006 | 24271 | 97.06 | 325 |  |  |  |
| *Paphiopedilum lawrenceanum* | C | NC_069877.1 | 50224 | 48150 | 95.87 | 328 |  |  |  |
| *Paphiopedilum leucochilum* | C | NC_069878.1 | 49669 | 45919 | 92.45 | 327 |  |  |  |
| *Paphiopedilum liemianum* | C | NC_069879.1 | 42979 | 36463 | 84.84 | 327 |  |  |  |
| *Paphiopedilum lowii* | C | NC_069880.1 | 49718 | 47665 | 95.87 | 325 |  |  |  |
| *Paphiopedilum malipoense* | C | NC_069881.1 | 49717 | 43125 | 86.74 | 327 |  |  |  |
| *Paphiopedilum mastersianum* | C | NC_069883.1 | 50073 | 43433 | 86.74 | 329 |  |  |  |
| *Paphiopedilum micranthum* | C | MN535014.1 | 49998 | 43898 | 87.80 | 327 |  |  |  |
| *Paphiopedilum moquetteanum* | C | NC_069884.1 | 50083 | 43442 | 86.74 | 326 |  |  |  |
| *Paphiopedilum nataschae* | C | NC_069885.1 | 50047 | 48576 | 97.06 | 327 |  |  |  |
| *Paphiopedilum niveum* | C | KJ524105.1 | 50009 | 47944 | 95.87 | 327 |  |  |  |
| *Paphiopedilum papuanum* | C | NC_069886.1 | 40998 | 39469 | 96.27 | 327 |  |  |  |
| *Paphiopedilum parishii* | C | MW528213.1 | 49529 | 45790 | 92.45 | 326 |  |  |  |
| *Paphiopedilum parnatanum* | C | NC_069887.1 | 50025 | 43392 | 86.74 | 325 |  |  |  |
| *Paphiopedilum philippinense* | C | MW794131.1 | 38371 | 34484 | 89.87 | 328 |  |  |  |
| *Paphiopedilum primulinum* | C | OK120860.1 | 38346 | 37153 | 96.89 | 326 |  |  |  |
| *Paphiopedilum purpuratum* | C | MN535015.1 | 49964 | 43868 | 87.80 | 324 |  |  |  |
| *Paphiopedilum randsii* | C | NC_069889.1 | 50082 | 43441 | 86.74 | 325 |  |  |  |
| *Paphiopedilum rothschildianum* | C | NC_069890.1 | 49975 | 43878 | 87.80 | 326 |  |  |  |
| *Paphiopedilum rungsuriyanum* | C | NC_069891.1 | 49761 | 47706 | 95.87 | 323 |  |  |  |
| *Paphiopedilum sanderianum* | C | NC_069892.1 | 50115 | 47519 | 94.82 | 327 |  |  |  |
| *Paphiopedilum sangii* | C | NC_069893.1 | 49092 | 47649 | 97.06 | 326 |  |  |  |
| *Paphiopedilum schoseri* | C | NC_069894.1 | 50157 | 43135 | 86.00 | 326 |  |  |  |
| *Paphiopedilum spicerianum* | C | OM066324.1 | 50181 | 48109 | 95.87 | 327 |  |  |  |
| *Paphiopedilum stonei* | C | NC_069895.1 | 49686 | 42154 | 84.84 | 326 |  |  |  |
| *Paphiopedilum sugiyamanum* | C | NC_069896.1 | 49908 | 43290 | 86.74 | 323 |  |  |  |
| *Paphiopedilum sukhakulii* | C | NC_069897.1 | 50101 | 43989 | 87.80 | 327 |  |  |  |
| *Paphiopedilum supardii* | C | NC_069898.1 | 50187 | 43532 | 86.74 | 326 |  |  |  |
| *Paphiopedilum superbiens* | C | NC_069899.1 | 50079 | 43439 | 86.74 | 323 |  |  |  |
| *Paphiopedilum tigrinum* | C | NC_062049.1 | 49987 | 47398 | 94.82 | 325 |  |  |  |
| *Paphiopedilum tonsum* | C | NC_069900.1 | 50166 | 43514 | 86.74 | 326 |  |  |  |
| *Paphiopedilum tranlienianum* | C | MW794129.1 | 50319 | 48241 | 95.87 | 326 |  |  |  |
| *Paphiopedilum urbanianum* | C | NC_069901.1 | 14991 | 14432 | 96.27 | 327 |  |  |  |
| *Paphiopedilum vejvarutianum* | C | NC_069902.1 | 49791 | 43189 | 86.74 | 323 |  |  |  |
| *Paphiopedilum venustum* | C | MZ150831.1 | 60072 | 52106 | 86.74 | 326 |  |  |  |
| *Paphiopedilum victoria-mariae* | C | MW794133.1 | 59531 | 57072 | 95.87 | 329 |  |  |  |
| *Paphiopedilum vietnamense* | C | NC_069905.1 | 22089 | 20121 | 91.09 | 327 |  |  |  |
| *Paphiopedilum villosum var* | C | OL875115.1 | 59654 | 50610 | 84.84 | 326 |  |  |  |
| *Paphiopedilum violascens* | C | MW794134.1 | 27333 | 26751 | 97.87 | 324 |  |  |  |
| *Paphiopedilum wardii* | C | NC_069907.1 | 11241 | 10102 | 89.87 | 330 |  |  |  |
| *Paphiopedilum wenshanense* | C | MZ150830.1 | 38966 | 37357 | 95.87 | 322 |  |  |  |
| *Paphiopedilum wilhelminae* | C | NC_069909.1 | 24966 | 21656 | 86.74 | 324 |  |  |  |
| *Paphiopedilum x areeanum* | C | NC_069955.1 | 23549 | 20426 | 86.74 | 329 |  |  |  |
| *Paphiopedilum x fanaticum* | C | NC_069969.1 | 26891 | 26318 | 97.87 | 327 |  |  |  |
| *Paphiopedilum x herrmannii* | C | NC_069872.1 | 93398 | 90652 | 97.06 | 389 |  |  |  |
| *Phragmipedium besseae* | C | NC_050285.1 | 55453 | 48100 | 86.74 | 335 |  |  |  |
| *Phragmipedium boissierianum* | C | MW794127.1 | 34842 | 34400 | 98.73 | 331 |  |  |  |
| *Phragmipedium hirtzii* | C | NC_069911.1 | 18262 | 17725 | 97.06 | 334 |  |  |  |
| *Phragmipedium kovachii* | C | NC_069912.1 | 18933 | 16623 | 87.80 | 334 |  |  |  |
| *Phragmipedium lindenii* | C | MW794128.1 | 23208 | 22913 | 98.73 | 333 |  |  |  |
| *Phragmipedium longifolium* | C | NC_069910.1 | 15906 | 15704 | 98.73 | 334 |  |  |  |
| *Acriopsis javanica* | E | NC_067758.1 | 19429 | 13584 | 69.92 | 352 | 364.2 (368; 360.0–376.0; 157.0–400.0) | 84.97 |  |
| *Anathallis microphyta* | E | MW375124.1 | 48351 | 40680 | 84.13 | 359 |  |  |  |
| *Anathallis obovata* | E | MH979332.1 | 29713 | 21216 | 71.40 | 362 |  |  |  |
| *Anoectochilus burmannicus* | E | NC_066958.1 | 39638 | 34260 | 86.43 | 341 |  |  |  |
| *Anoectochilus chapaensis* | E | MW589500.1 | 48188 | 43677 | 90.64 | 341 |  |  |  |
| *Anoectochilus emeiensis* | E | LC057212.1 | 48063 | 39046 | 81.24 | 342 |  |  |  |
| *Anoectochilus formosanus* | E | MN880624.1 | 18212 | 16400 | 90.05 | 341 |  |  |  |
| *Anoectochilus hainanensis* | E | MW589501.1 | 38815 | 24857 | 64.04 | 342 |  |  |  |
| *Anoectochilus roxburghii* | E | MN880626.1 | 28863 | 21021 | 72.83 | 343 |  |  |  |
| *Anoectochilus zhejiangensis* | E | MW173020.1 | 19637 | 15894 | 80.94 | 343 |  |  |  |
| *Bletilla formosana* | E | MN526744.1 | 16012 | 14611 | 91.25 | 378 |  |  |  |
| *Bletilla ochracea* | E | KT695602.1 | 42012 | 34521 | 82.17 | 374 |  |  |  |
| *Bletilla sinensis* | E | MT806143.1 | 48226 | 35647 | 73.92 | 372 |  |  |  |
| *Bletilla striata* | E | KT588924.1 | 48561 | 32914 | 67.78 | 372 |  |  |  |
| *Bulbophyllum affine* | E | LC556091.1 | 47468 | 33243 | 70.03 | 367 |  |  |  |
| *Bulbophyllum albociliatum* | E | NC_070215.1 | 15963 | 10378 | 65.01 | 367 |  |  |  |
| *Bulbophyllum andersonii* | E | LC703293.1 | 29725 | 18867 | 63.47 | 363 |  |  |  |
| *Bulbophyllum disciflorum* | E | LC498826.1 | 24823 | 18215 | 73.38 | 366 |  |  |  |
| *Bulbophyllum epiphytum* | E | MN737573.1 | 33069 | 28666 | 86.69 | 361 |  |  |  |
| *Bulbophyllum exaltatum* | E | MN604054.1 | 48145 | 37021 | 76.89 | 359 |  |  |  |
| *Bulbophyllum flammuliferum* | E | NC_084215.1 | 48018 | 36712 | 76.45 | 369 |  |  |  |
| *Bulbophyllum forrestii* | E | LC642719.1 | 48236 | 37735 | 78.23 | 365 |  |  |  |
| *Bulbophyllum funingense* | E | LC556093.1 | 48249 | 42093 | 87.24 | 364 |  |  |  |
| *Bulbophyllum gedangense* | E | MW161053.1 | 42114 | 32870 | 78.05 | 367 |  |  |  |
| *Bulbophyllum granulosum* | E | MN604055.1 | 96124 | 91086 | 94.76 | 369 |  |  |  |
| *Bulbophyllum hirtum* | E | LC642724.1 | 48114 | 39883 | 82.89 | 360 |  |  |  |
| *Bulbophyllum hirundinis* | E | LC642721.1 | 47755 | 43762 | 91.64 | 361 |  |  |  |
| *Bulbophyllum inconspicuum* | E | MN200377.1 | 49829 | 34385 | 69.01 | 369 |  |  |  |
| *Bulbophyllum kwangtungense* | E | LC642722.1 | 49936 | 37133 | 74.36 | 360 |  |  |  |
| *Bulbophyllum leopardinum* | E | LC642723.1 | 49989 | 36785 | 73.59 | 364 |  |  |  |
| *Bulbophyllum lingii* | E | MW161052.1 | 39988 | 33436 | 83.62 | 360 |  |  |  |
| *Bulbophyllum menghaiense* | E | MW161050.1 | 44837 | 33155 | 73.95 | 359 |  |  |  |
| *Bulbophyllum mentosum* | E | MN604056.1 | 48783 | 42787 | 87.71 | 364 |  |  |  |
| *Bulbophyllum ningboense* | E | MW683325.1 | 48208 | 42767 | 88.71 | 365 |  |  |  |
| *Bulbophyllum odoratissimum* | E | LC642720.1 | 96297 | 84033 | 87.26 | 360 |  |  |  |
| *Bulbophyllum orientale* | E | LC642725.1 | 28262 | 27449 | 97.12 | 362 |  |  |  |
| *Bulbophyllum pectinatum* | E | LC556092.1 | 97124 | 88235 | 90.85 | 364 |  |  |  |
| *Bulbophyllum pentaneurum* | E | MW161051.1 | 13240 | 11253 | 84.99 | 358 |  |  |  |
| *Bulbophyllum pingnanense* | E | MW822749.1 | 48747 | 38688 | 79.36 | 365 |  |  |  |
| *Bulbophyllum plumosum* | E | MN580547.1 | 24858 | 22085 | 88.84 | 357 |  |  |  |
| *Bulbophyllum regnellii* | E | MN604057.1 | 50047 | 43265 | 86.45 | 369 |  |  |  |
| *Bulbophyllum reptans* | E | MW800884.1 | 38346 | 33707 | 87.90 | 380 |  |  |  |
| *Bulbophyllum steyermarkii* | E | MN604058.1 | 49946 | 43282 | 86.66 | 364 |  |  |  |
| *Bulbophyllum tianguii* | E | MZ983368.1 | 49939 | 45749 | 91.61 | 367 |  |  |  |
| *Bulbophyllum weddellii* | E | MN604059.1 | 33012 | 30510 | 92.42 | 362 |  |  |  |
| *Bulleyia yunnanensis* | E | MN822195.1 | 47605 | 45453 | 95.48 | 375 |  |  |  |
| *Calanthe alismifolia* | E | NC_084137.1 | 47951 | 42703 | 89.06 | 375 |  |  |  |
| *Calanthe alleizettei* | E | NC_084138.1 | 29264 | 28915 | 98.81 | 378 |  |  |  |
| *Calanthe alpina* | E | NC_067584.1 | 96363 | 84243 | 87.42 | 373 |  |  |  |
| *Calanthe angustifolia* | E | NC_084139.1 | 55762 | 49388 | 88.57 | 377 |  |  |  |
| *Calanthe arcuata* | E | OK075084.1 | 96648 | 86852 | 89.86 | 378 |  |  |  |
| *Calanthe arcuata var* | E | OK180390.1 | 54998 | 41582 | 75.61 | 378 |  |  |  |
| *Calanthe argenteostriata* | E | NC_084140.1 | 96518 | 95203 | 98.64 | 372 |  |  |  |
| *Calanthe arisanensis* | E | NC_084141.1 | 48158 | 42068 | 87.35 | 376 |  |  |  |
| *Calanthe aristulifera* | E | MN200378.1 | 48699 | 34470 | 70.78 | 377 |  |  |  |
| *Calanthe bicolor* | E | MN200379.1 | 97425 | 78533 | 80.61 | 377 |  |  |  |
| *Calanthe brevicornu* | E | NC_067586.1 | 48171 | 39252 | 81.48 | 378 |  |  |  |
| *Calanthe cardioglossa* | E | NC_084142.1 | 28299 | 25069 | 88.59 | 378 |  |  |  |
| *Calanthe clavata* | E | OK180395.1 | 48301 | 45742 | 94.70 | 378 |  |  |  |
| *Calanthe davidii* | E | MG925365.1 | 48062 | 46388 | 96.52 | 373 |  |  |  |
| *Calanthe delavayi* | E | MK388860.1 | 46333 | 40835 | 88.13 | 369 |  |  |  |
| *Calanthe densiflora* | E | NC_084143.1 | 29473 | 24408 | 82.81 | 378 |  |  |  |
| *Calanthe discolor* | E | NC_068094.1 | 48071 | 42338 | 88.07 | 378 |  |  |  |
| *Calanthe ecarinata* | E | NC_067587.1 | 92787 | 54333 | 58.56 | 378 |  |  |  |
| *Calanthe flava* | E | OR159226.1 | 49666 | 19442 | 39.15 | 379 |  |  |  |
| *Calanthe graciliflora* | E | NC_072910.1 | 22753 | 21685 | 95.31 | 378 |  |  |  |
| *Calanthe griffithii* | E | MZ474966.1 | 50038 | 47187 | 94.30 | 380 |  |  |  |
| *Calanthe hancockii* | E | MW683326.1 | 47422 | 40851 | 86.14 | 378 |  |  |  |
| *Calanthe henryi* | E | MT385870.1 | 95967 | 34436 | 35.88 | 377 |  |  |  |
| *Calanthe herbacea* | E | NC_062706.1 | 96332 | 74136 | 76.96 | 375 |  |  |  |
| *Calanthe lamellosa* | E | NC_084145.1 | 28428 | 22319 | 78.51 | 377 |  |  |  |
| *Calanthe lechangensis* | E | NC_084146.1 | 47945 | 41812 | 87.21 | 375 |  |  |  |
| *Calanthe lyroglossa* | E | MN708350.1 | 58942 | 50725 | 86.06 | 377 |  |  |  |
| *Calanthe mannii* | E | NC_084147.1 | 48778 | 43884 | 89.97 | 378 |  |  |  |
| *Calanthe mcgregorii* | E | NC_084148.1 | 23968 | 19010 | 79.31 | 375 |  |  |  |
| *Calanthe metoensis* | E | NC_084149.1 | 24834 | 19331 | 77.84 | 378 |  |  |  |
| *Calanthe nipponica* | E | NC_067588.1 | 48017 | 45058 | 93.84 | 378 |  |  |  |
| *Calanthe reflexa* | E | NC_062707.1 | 49529 | 42797 | 86.41 | 377 |  |  |  |
| *Calanthe rubens* | E | MN708352.1 | 24991 | 19772 | 79.12 | 373 |  |  |  |
| *Calanthe sinica* | E | NC_084150.1 | 49718 | 42754 | 85.99 | 374 |  |  |  |
| *Calanthe sp* | E | OK180383.1 | 49998 | 47228 | 94.46 | 373 |  |  |  |
| *Calanthe striata var* | E | OK180408.1 | 50259 | 44289 | 88.12 | 378 |  |  |  |
| *Calanthe sylvatica* | E | MK736029.1 | 34979 | 25984 | 74.28 | 376 |  |  |  |
| *Calanthe taibaishanensis* | E | NC_067589.1 | 48185 | 34483 | 71.56 | 379 |  |  |  |
| *Calanthe tricarinata* | E | NC_067590.1 | 33962 | 31934 | 94.03 | 378 |  |  |  |
| *Calanthe trifida* | E | NC_084151.1 | 48108 | 34567 | 71.85 | 378 |  |  |  |
| *Calanthe triplicata* | E | KF753635.1 | 48268 | 42411 | 87.87 | 375 |  |  |  |
| *Calanthe tsoongiana* | E | NC_080272.1 | 54843 | 50104 | 91.36 | 378 |  |  |  |
| *Calanthe yaoshanensis* | E | NC_084152.1 | 48198 | 47634 | 98.83 | 378 |  |  |  |
| *Cattleya crispata* | E | NC_084153.1 | 47351 | 34342 | 72.53 | 378 |  |  |  |
| *Cattleya liliputana* | E | KP168671.1 | 48283 | 40062 | 82.97 | 380 |  |  |  |
| *Cephalanthera damasonium* | E | KP202881.1 | 48631 | 40339 | 82.95 | 380 |  |  |  |
| *Cephalanthera falcata* | E | MH590345.1 | 96875 | 93536 | 96.55 | 365 |  |  |  |
| *Cephalanthera longibracteata* | E | NC_082061.1 | 55827 | 50269 | 90.04 | 364 |  |  |  |
| *Cephalanthera longifolia* | E | MH590346.1 | 31794 | 29209 | 91.87 | 366 |  |  |  |
| *Cephalanthera nanchuanica* | E | KU551263.1 | 48428 | 40839 | 84.33 | 365 |  |  |  |
| *Cephalanthera rubra* | E | OR947462.1 | 29845 | 27985 | 93.77 | 365 |  |  |  |
| *Cephalantheropsis halconensis* | E | MH590347.1 | 94269 | 92210 | 97.82 | 376 |  |  |  |
| *Cephalantheropsis obcordata* | E | NC_084154.1 | 13518 | 11261 | 83.30 | 376 |  |  |  |
| *Chamaegastrodia inverta* | E | MN708351.1 | 48717 | 45268 | 92.92 | 210 |  |  |  |
| *Chamaegastrodia shikokiana* | E | MW589502.1 | 50043 | 41878 | 83.68 | 233 |  |  |  |
| *Changnienia amoena* | E | MN200367.1 | 49921 | 44900 | 89.94 | 376 |  |  |  |
| *Cheirostylis chinensis* | E | MN047293.1 | 49983 | 45286 | 90.60 | 330 |  |  |  |
| *Cheirostylis moniliformis* | E | MN641483.1 | 21317 | 18632 | 87.40 | 332 |  |  |  |
| *Cheirostylis montana* | E | MW589503.1 | 96289 | 90234 | 93.71 | 331 |  |  |  |
| *Cheirostylis thailandica* | E | MW589504.1 | 53918 | 46901 | 86.99 | 327 |  |  |  |
| *Chiloschista exuperei* | E | MK848867.1 | 47937 | 45743 | 95.42 | 377 |  |  |  |
| *Chiloschista parishii* | E | NC_072256.1 | 48618 | 44309 | 91.14 | 377 |  |  |  |
| *Chiloschista yunnanensis* | E | NC_071783.1 | 96274 | 86089 | 89.42 | 378 |  |  |  |
| *Chrysoglossum ornatum* | E | NC_067616.1 | 46323 | 43546 | 94.01 | 371 |  |  |  |
| *Claderia viridiflora* | E | NC_082281.1 | 48691 | 39889 | 81.92 | 376 |  |  |  |
| *Coelogyne articulata* | E | NC_084191.1 | 96419 | 61565 | 63.85 | 374 |  |  |  |
| *Coelogyne barbata* | E | MT712149.1 | 48546 | 34700 | 71.48 | 378 |  |  |  |
| *Coelogyne bulleyia* | E | MT627604.1 | 29143 | 27916 | 95.79 | 373 |  |  |  |
| *Coelogyne cantonensis* | E | MT610368.1 | 29579 | 23975 | 81.05 | 374 |  |  |  |
| *Coelogyne chen-tsii* | E | NC_067981.1 | 24432 | 17337 | 70.96 | 377 |  |  |  |
| *Coelogyne chinensis* | E | MT704835.1 | 41492 | 37003 | 89.18 | 376 |  |  |  |
| *Coelogyne corymbosa* | E | NC_067982.1 | 47973 | 42984 | 89.60 | 374 |  |  |  |
| *Coelogyne fimbriata* | E | MT712148.1 | 25009 | 19004 | 75.99 | 377 |  |  |  |
| *Coelogyne flaccida* | E | MK946948.1 | 96565 | 89139 | 92.31 | 373 |  |  |  |
| *Coelogyne imbricata* | E | NC_063910.1 | 53814 | 41394 | 76.92 | 378 |  |  |  |
| *Coelogyne kouytcheensis* | E | MN398392.1 | 35353 | 29651 | 83.87 | 373 |  |  |  |
| *Coelogyne leveilleana* | E | MT610370.1 | 48116 | 40321 | 83.80 | 374 |  |  |  |
| *Coelogyne missionariorum* | E | NC_067983.1 | 34856 | 27707 | 79.49 | 372 |  |  |  |
| *Coelogyne niana* | E | NC_067984.1 | 47531 | 37150 | 78.16 | 375 |  |  |  |
| *Coelogyne ovalis* | E | NC_067985.1 | 48202 | 44606 | 92.54 | 378 |  |  |  |
| *Coelogyne pallida* | E | MK946949.1 | 48433 | 38867 | 80.25 | 377 |  |  |  |
| *Coelogyne protracta* | E | NC_067986.1 | 32094 | 29453 | 91.77 | 375 |  |  |  |
| *Coelogyne uniflora* | E | NC_067987.1 | 25006 | 19400 | 77.58 | 373 |  |  |  |
| *Coelogyne ventricosa* | E | MT610371.1 | 31933 | 29679 | 92.94 | 377 |  |  |  |
| *Coelogyne wenshanica* | E | NC_067988.1 | 73185 | 57875 | 79.08 | 375 |  |  |  |
| *Collabium chinense* | E | NC_067989.1 | 94257 | 71051 | 75.38 | 377 |  |  |  |
| *Collabium formosanum* | E | NC_084155.1 | 50073 | 43073 | 86.02 | 377 |  |  |  |
| *Corallorhiza maculata* | E | MZ618356.1 | 50083 | 46247 | 92.34 | 319 |  |  |  |
| *Corallorhiza sinensis* | E | MN200380.1 | 49717 | 44089 | 88.68 | 347 |  |  |  |
| *Corallorhiza striata var* | E | MW191791.1 | 50025 | 40350 | 80.66 | 274 |  |  |  |
| *Crepidium elegans* | E | MN200381.1 | 96441 | 89247 | 92.54 | 361 |  |  |  |
| *Crepidium purpureum* | E | NC_084118.1 | 96674 | 79186 | 81.91 | 364 |  |  |  |
| *Cryptochilus roseus* | E | NC_084377.1 | 92573 | 83686 | 90.40 | 378 |  |  |  |
| *Cyclopogon comosus* | E | NC_067941.1 | 48252 | 47519 | 98.48 | 321 |  |  |  |
| *Cyclopogon longibracteatus* | E | MW589506.1 | 95435 | 90081 | 94.39 | 321 |  |  |  |
| *Cymbidium aestivum* | E | MN597436.1 | 96521 | 84060 | 87.09 | 369 |  |  |  |
| *Cymbidium aloifolium* | E | MK848033.1 | 19345 | 15987 | 82.64 | 363 |  |  |  |
| *Cymbidium aloifolium* | E | MK848046.1 | 73052 | 61006 | 83.51 | 366 |  |  |  |
| *Cymbidium atropurpureum* | E | MK848053.1 | 49747 | 41842 | 84.11 | 366 |  |  |  |
| *Cymbidium banaense* | E | MK848060.1 | 50224 | 37768 | 75.20 | 362 |  |  |  |
| *Cymbidium bicolor* | E | MN654912.1 | 85755 | 74401 | 86.76 | 366 |  |  |  |
| *Cymbidium canaliculatum* | E | MK848061.1 | 19754 | 16313 | 82.58 | 369 |  |  |  |
| *Cymbidium caulescens* | E | MK848059.1 | 21974 | 20398 | 92.83 | 371 |  |  |  |
| *Cymbidium changningense* | E | MK848044.1 | 95855 | 91254 | 95.20 | 373 |  |  |  |
| *Cymbidium cochleare* | E | MK848049.1 | 95951 | 88073 | 91.79 | 371 |  |  |  |
| *Cymbidium cyperifolium* | E | MK848050.1 | 48217 | 36288 | 75.26 | 371 |  |  |  |
| *Cymbidium daweishanense* | E | MK848040.1 | 40822 | 32351 | 79.25 | 366 |  |  |  |
| *Cymbidium dayanum* | E | MK848039.1 | 48294 | 38302 | 79.31 | 364 |  |  |  |
| *Cymbidium defoliatum* | E | MK848048.1 | 48138 | 42217 | 87.70 | 370 |  |  |  |
| *Cymbidium eburneum* | E | MK820374.1 | 47888 | 39776 | 83.06 | 371 |  |  |  |
| *Cymbidium elegans* | E | NC_067753.1 | 48671 | 41288 | 84.83 | 364 |  |  |  |
| *Cymbidium ensifolium* | E | KT722983.1 | 48619 | 40237 | 82.76 | 371 |  |  |  |
| *Cymbidium erythraeum* | E | MK820373.1 | 37902 | 29821 | 78.68 | 371 |  |  |  |
| *Cymbidium faberi* | E | KR919606.1 | 37886 | 32165 | 84.90 | 370 |  |  |  |
| *Cymbidium floribundum* | E | MW582685.1 | 27642 | 25284 | 91.47 | 365 |  |  |  |
| *Cymbidium gaoligongense* | E | MK848054.1 | 47361 | 35639 | 75.25 | 368 |  |  |  |
| *Cymbidium goeringii* | E | KT722982.1 | 47262 | 36992 | 78.27 | 372 |  |  |  |
| *Cymbidium haematodes* | E | MK848047.1 | 47318 | 40220 | 85.00 | 371 |  |  |  |
| *Cymbidium hookerianum* | E | MT800927.1 | 20072 | 15381 | 76.63 | 365 |  |  |  |
| *Cymbidium iridioides* | E | MZ044639.1 | 48377 | 39756 | 82.18 | 370 |  |  |  |
| *Cymbidium kanran* | E | KU179435.1 | 48244 | 36598 | 75.86 | 368 |  |  |  |
| *Cymbidium lancifolium* | E | KU179436.1 | 49857 | 45699 | 91.66 | 370 |  |  |  |
| *Cymbidium longibracteatum* | E | MT259022.1 | 48621 | 40005 | 82.28 | 371 |  |  |  |
| *Cymbidium lowianum* | E | MT576628.1 | 49669 | 40952 | 82.45 | 365 |  |  |  |
| *Cymbidium macrorhizon* | E | KU179437.1 | 29577 | 23446 | 79.27 | 368 |  |  |  |
| *Cymbidium maguanense* | E | MK848036.1 | 15502 | 11972 | 77.23 | 368 |  |  |  |
| *Cymbidium mannii* | E | MT576626.1 | 96625 | 73899 | 76.48 | 370 |  |  |  |
| *Cymbidium mastersii* | E | MT576627.1 | 47886 | 44232 | 92.37 | 369 |  |  |  |
| *Cymbidium nanulum* | E | MK820372.1 | 16739 | 13957 | 83.38 | 368 |  |  |  |
| *Cymbidium paucifolium* | E | MK848055.1 | 48041 | 42617 | 88.71 | 370 |  |  |  |
| *Cymbidium qiubeiense* | E | MK848035.1 | 24811 | 18908 | 76.21 | 371 |  |  |  |
| *Cymbidium serratum* | E | MT273089.1 | 16725 | 14302 | 85.51 | 370 |  |  |  |
| *Cymbidium sinense* | E | MK848064.1 | 85817 | 67409 | 78.55 | 370 |  |  |  |
| *Cymbidium tortisepalum* | E | MK848058.1 | 96354 | 81631 | 84.72 | 365 |  |  |  |
| *Cymbidium tracyanum* | E | MW582691.1 | 39727 | 30844 | 77.64 | 369 |  |  |  |
| *Cymbidium tracyanum x Cymbidium faberi* | E | MT675524.1 | 96727 | 76753 | 79.35 | 369 |  |  |  |
| *Cymbidium wenshanense* | E | MK848057.1 | 48173 | 44473 | 92.32 | 370 |  |  |  |
| *Cymbidium wilsonii* | E | MK848051.1 | 30275 | 28138 | 92.94 | 339 |  |  |  |
| *Dendrobium acinaciforme FYJ2* | E | LC636124.1 | 35353 | 29940 | 84.69 | 368 |  |  |  |
| *Dendrobium aduncum ZSY06* | E | LC348858.1 | 48116 | 47851 | 99.45 | 364 |  |  |  |
| *Dendrobium aphyllum* | E | LC192953.1 | 34856 | 27397 | 78.60 | 368 |  |  |  |
| *Dendrobium bensoniae* | E | NC_072269.1 | 47531 | 38148 | 80.26 | 368 |  |  |  |
| *Dendrobium brymerianum* | E | LC192954.1 | 48202 | 36624 | 75.98 | 369 |  |  |  |
| *Dendrobium candidum* | E | MZ129032.1 | 48433 | 47726 | 98.54 | 368 |  |  |  |
| *Dendrobium capillipes* | E | NC_072707.1 | 32094 | 26949 | 83.97 | 365 |  |  |  |
| *Dendrobium cariniferum* | E | NC_080348.1 | 25006 | 19482 | 77.91 | 369 |  |  |  |
| *Dendrobium catenatum cultivar zhong ke IV hao* | E | KX507360.1 | 31933 | 24595 | 77.02 | 371 |  |  |  |
| *Dendrobium christyanum* | E | MZ241112.1 | 73185 | 57772 | 78.94 | 369 |  |  |  |
| *Dendrobium chrysanthum* | E | LC193514.1 | 94257 | 74802 | 79.36 | 365 |  |  |  |
| *Dendrobium chrysocrepis* | E | MN915015.1 | 50073 | 38411 | 76.71 | 374 |  |  |  |
| *Dendrobium chrysotoxum* | E | KT633996.1 | 50083 | 38133 | 76.14 | 367 |  |  |  |
| *Dendrobium comatum* | E | MZ666386.1 | 49717 | 37631 | 75.69 | 365 |  |  |  |
| *Dendrobium crepidatum* | E | LC193509.1 | 50025 | 38759 | 77.48 | 366 |  |  |  |
| *Dendrobium crystallinum LLD* | E | LC490676.1 | 38371 | 32389 | 84.41 | 363 |  |  |  |
| *Dendrobium denneanum* | E | LC192955.1 | 13949 | 10060 | 72.12 | 369 |  |  |  |
| *Dendrobium densiflorum* | E | MW007719.1 | 23915 | 20148 | 84.25 | 365 |  |  |  |
| *Dendrobium devonianum* | E | LC192956.1 | 49791 | 38389 | 77.10 | 367 |  |  |  |
| *Dendrobium ellipsophyllum* | E | LC193519.1 | 48427 | 36732 | 75.85 | 361 |  |  |  |
| *Dendrobium exile* | E | LC193522.1 | 47342 | 39398 | 83.22 | 365 |  |  |  |
| *Dendrobium falconeri* | E | LC192957.1 | 44807 | 38238 | 85.34 | 367 |  |  |  |
| *Dendrobium fanjingshanense* | E | LC193523.1 | 46286 | 36506 | 78.87 | 365 |  |  |  |
| *Dendrobium fimbriatum* | E | LC193521.1 | 46279 | 39388 | 85.11 | 369 |  |  |  |
| *Dendrobium findlayanum* | E | MZ424316.1 | 34313 | 26991 | 78.66 | 366 |  |  |  |
| *Dendrobium flexicaule* | E | OQ360111.1 | 96441 | 80683 | 83.66 | 369 |  |  |  |
| *Dendrobium gratiosissimum* | E | LC192958.1 | 96674 | 76604 | 79.24 | 356 |  |  |  |
| *Dendrobium hainanense* | E | NC_067939.1 | 92573 | 77521 | 83.74 | 369 |  |  |  |
| *Dendrobium hancockii HZY* | E | LC500592.1 | 48252 | 40585 | 84.11 | 367 |  |  |  |
| *Dendrobium henryi* | E | LC193513.1 | 95435 | 90482 | 94.81 | 368 |  |  |  |
| *Dendrobium hercoglossum* | E | LC192959.1 | 96521 | 90903 | 94.18 | 367 |  |  |  |
| *Dendrobium heterocarpum* | E | NC_068067.1 | 19345 | 15058 | 77.84 | 366 |  |  |  |
| *Dendrobium huoshanense* | E | KT630834.1 | 73052 | 56323 | 77.10 | 367 |  |  |  |
| *Dendrobium jenkinsii* | E | LC193515.1 | 49747 | 41658 | 83.74 | 365 |  |  |  |
| *Dendrobium kingianum* | E | LC331062.1 | 50224 | 38346 | 76.35 | 365 |  |  |  |
| *Dendrobium lindleyi FYJ2* | E | LC636121.1 | 85755 | 72043 | 84.01 | 368 |  |  |  |
| *Dendrobium lituiflorum LLD23_2* | E | LC490680.1 | 19754 | 16631 | 84.19 | 349 |  |  |  |
| *Dendrobium loddigesii* | E | ON028638.1 | 21974 | 17203 | 78.29 | 367 |  |  |  |
| *Dendrobium lohohense* | E | LC193516.1 | 95855 | 92433 | 96.43 | 367 |  |  |  |
| *Dendrobium longicornu* | E | MN227146.1 | 95951 | 93341 | 97.28 | 368 |  |  |  |
| *Dendrobium moniliforme* | E | MN200384.1 | 48217 | 36823 | 76.37 | 368 |  |  |  |
| *Dendrobium moniliforme cultivar Royaldream* | E | MN200385.1 | 40822 | 30796 | 75.44 | 368 |  |  |  |
| *Dendrobium moniliforme cultivar Sangeum* | E | MN200383.1 | 48294 | 40210 | 83.26 | 367 |  |  |  |
| *Dendrobium moniliforme LLD04_* | E | LC490377.1 | 48138 | 40436 | 84.00 | 367 |  |  |  |
| *Dendrobium moniliforme LLD04_0* | E | LC490386.1 | 47888 | 36864 | 76.98 | 367 |  |  |  |
| *Dendrobium moniliforme LLD04_042* | E | LC490652.1 | 48671 | 41541 | 85.35 | 367 |  |  |  |
| *Dendrobium moniliforme LLD04_20* | E | LC490387.1 | 48619 | 41642 | 85.65 | 366 |  |  |  |
| *Dendrobium moschatum* | E | NC_061572.1 | 37902 | 29215 | 77.08 | 367 |  |  |  |
| *Dendrobium nobile* | E | KT591465.1 | 37886 | 29468 | 77.78 | 363 |  |  |  |
| *Dendrobium ochreatum* | E | MW665324.1 | 27642 | 24084 | 87.13 | 371 |  |  |  |
| *Dendrobium odiosum* | E | MZ905453.1 | 47361 | 36203 | 76.44 | 369 |  |  |  |
| *Dendrobium officinale* | E | KC771275.1 | 47262 | 35773 | 75.69 | 359 |  |  |  |
| *Dendrobium parciflorum* | E | LC193512.1 | 47318 | 39809 | 84.13 | 370 |  |  |  |
| *Dendrobium parishii* | E | LC193518.1 | 20072 | 15769 | 78.56 | 365 |  |  |  |
| *Dendrobium pendulum* | E | KT695604.1 | 48377 | 38561 | 79.71 | 366 |  |  |  |
| *Dendrobium primulinum* | E | LC192810.1 | 48244 | 38465 | 79.73 | 363 |  |  |  |
| *Dendrobium pseudotenellum* | E | MN915016.1 | 49857 | 41511 | 83.26 | 366 |  |  |  |
| *Dendrobium salaccense* | E | LC193510.1 | 48621 | 38012 | 78.18 | 367 |  |  |  |
| *Dendrobium scoriarum ZSY02* | E | LC348850.1 | 49669 | 39129 | 78.78 | 369 |  |  |  |
| *Dendrobium shixingense ZSY0900* | E | LC348860.1 | 29577 | 25022 | 84.60 | 372 |  |  |  |
| *Dendrobium sinense* | E | NC_067940.1 | 15502 | 12357 | 79.71 | 359 |  |  |  |
| *Dendrobium sinominutiflorum* | E | NC_061573.1 | 15502 | 12357 | 79.71 | 362 |  |  |  |
| *Dendrobium spatella* | E | LC193511.1 | 96625 | 81841 | 84.70 | 371 |  |  |  |
| *Dendrobium strongylanthum* | E | KR673323.1 | 47886 | 40334 | 84.23 | 365 |  |  |  |
| *Dendrobium stuposum LLD202206_00* | E | LC715249.1 | 16739 | 13883 | 82.94 | 369 |  |  |  |
| *Dendrobium sulcatum* | E | NC_084375.1 | 48041 | 38553 | 80.25 | 356 |  |  |  |
| *Dendrobium terminale FYJ2* | E | LC635346.1 | 24811 | 20578 | 82.94 | 369 |  |  |  |
| *Dendrobium thyrsiflorum* | E | MN306203.1 | 16725 | 14584 | 87.20 | 157 |  |  |  |
| *Dendrobium trigonopus* | E | NC_068068.1 | 85817 | 68576 | 79.91 | 370 |  |  |  |
| *Dendrobium wangliangii* | E | MT798588.1 | 96354 | 91392 | 94.85 | 368 |  |  |  |
| *Dendrobium wattii* | E | MN913340.1 | 39727 | 32691 | 82.29 | 366 |  |  |  |
| *Dendrobium williamsonii* | E | OK173601.1 | 96727 | 84559 | 87.42 | 368 |  |  |  |
| *Dendrobium wilsonii* | E | LC193508.1 | 48173 | 38360 | 79.63 | 368 |  |  |  |
| *Dendrobium xichouense* | E | LC193520.1 | 30275 | 27665 | 91.38 | 362 |  |  |  |
| *Dendrobium zhenghuoense* | E | MN164627.1 | 33256 | 28790 | 86.57 | 163 |  |  |  |
| *Diplandrorchis sinica* | E | MZ014629.1 | 95433 | 85537 | 89.63 | 357 |  |  |  |
| *Dipodium roseum* | E | MN200386.1 | 50021 | 43723 | 87.41 | 337 |  |  |  |
| *Dracula erythrochaete* | E | OR713738.1 | 27763 | 23726 | 85.46 | 363 |  |  |  |
| *Dracula mendozae* | E | OR713737.1 | 79629 | 69683 | 87.51 | 364 |  |  |  |
| *Dryadella lilliputiana* | E | MW375126.1 | 79825 | 65065 | 81.51 | 373 |  |  |  |
| *Elleanthus sodiroi* | E | KR260986.1 | 62524 | 54790 | 87.63 | 376 |  |  |  |
| *Epidendrum avicula* | E | OR460026.1 | 62514 | 54062 | 86.48 | 375 |  |  |  |
| *Epidendrum ciliare* | E | OR460022.1 | 19689 | 13987 | 71.04 | 370 |  |  |  |
| *Epidendrum diffusum* | E | OR460023.1 | 97085 | 56669 | 58.37 | 374 |  |  |  |
| *Epidendrum eburneum* | E | OR460025.1 | 26321 | 22891 | 86.97 | 369 |  |  |  |
| *Epidendrum porpax* | E | OR460024.1 | 42637 | 35176 | 82.50 | 354 |  |  |  |
| *Epipactis albensis* | E | MH590348.1 | 78306 | 69959 | 89.34 | 356 |  |  |  |
| *Epipactis atrorubens* | E | MH590349.1 | 78192 | 65861 | 84.23 | 358 |  |  |  |
| *Epipactis gigantea* | E | MH590350.1 | 94965 | 85032 | 89.54 | 354 |  |  |  |
| *Epipactis helleborine* | E | MH590351.1 | 29521 | 23705 | 80.30 | 358 |  |  |  |
| *Epipactis mairei* | E | KU551264.1 | 43177 | 36903 | 85.47 | 357 |  |  |  |
| *Epipactis microphylla* | E | MH590352.1 | 43042 | 37218 | 86.47 | 357 |  |  |  |
| *Epipactis palustris* | E | MH590353.1 | 52687 | 45901 | 87.12 | 354 |  |  |  |
| *Epipactis purpurata* | E | MH590354.1 | 52653 | 46708 | 88.71 | 359 |  |  |  |
| *Epipactis thunbergii* | E | MN200387.1 | 29109 | 24527 | 84.26 | 357 |  |  |  |
| *Epipactis veratrifolia* | E | KU551267.1 | 29042 | 24924 | 85.82 | 373 |  |  |  |
| *Eria corneri* | E | MN477202.1 | 21682 | 17317 | 79.87 | 376 |  |  |  |
| *Eria lasiopetala* | E | MZ424201.1 | 21693 | 18756 | 86.46 | 331 |  |  |  |
| *Erycina pusilla* | E | JF746994.1 | 13288 | 10567 | 79.52 | 346 |  |  |  |
| *Erythrodes blumei* | E | MW589509.1 | 13378 | 10819 | 80.87 | 348 |  |  |  |
| *Erythrodes hirsuta* | E | MW589510.1 | 20136 | 16969 | 84.27 | 348 |  |  |  |
| *Eucosia viridiflora* | E | MW589521.1 | 20065 | 17792 | 88.67 | 359 |  |  |  |
| *Eulophia flava* | E | MK855051.1 | 94192 | 81655 | 86.69 | 361 |  |  |  |
| *Eulophia guineensis* | E | NC_081004.1 | 94104 | 74088 | 78.73 | 349 |  |  |  |
| *Eulophia zollingeri* | E | MG181954.1 | 95332 | 84884 | 89.04 | 311 |  |  |  |
| *Eurystyles cotyledon* | E | MN597435.1 | 95633 | 81125 | 84.83 | 356 |  |  |  |
| *Gastrochilus calceolaris* | E | MH719016.1 | 95664 | 84548 | 88.38 | 386 |  |  |  |
| *Gastrochilus fuscopunctatus* | E | KX871233.1 | 95909 | 74262 | 77.43 | 393 |  |  |  |
| *Gastrochilus japonicus* | E | KX871236.1 | 75626 | 53725 | 71.04 | 393 |  |  |  |
| *Gastrochilus sinensis* | E | NC_080211.1 | 75537 | 66563 | 88.12 | 357 |  |  |  |
| *Geodorum attenuatum* | E | NC_072631.1 | 18526 | 14723 | 79.47 | 357 |  |  |  |
| *Geodorum densiflorum* | E | NC_072632.1 | 31854 | 25505 | 80.07 | 359 |  |  |  |
| *Geodorum eulophioides* | E | OP820525.1 | 22655 | 18165 | 80.18 | 357 |  |  |  |
| *Geodorum recurvum* | E | NC_072527.1 | 22755 | 19421 | 85.35 | 363 |  |  |  |
| *Gomesa flexuosa* | E | NC_062132.1 | 46915 | 40492 | 86.31 | 348 |  |  |  |
| *Goodyera biflora* | E | NC_064114.1 | 46979 | 37001 | 78.76 | 346 |  |  |  |
| *Goodyera foliosa* | E | MW589512.1 | 75163 | 64445 | 85.74 | 352 |  |  |  |
| *Goodyera fumata* | E | KJ501999.1 | 74803 | 64495 | 86.22 | 348 |  |  |  |
| *Goodyera henryi* | E | NC_064115.1 | 42104 | 33207 | 78.87 | 345 |  |  |  |
| *Goodyera malipoensis* | E | MW589514.1 | 42099 | 36399 | 86.46 | 344 |  |  |  |
| *Goodyera marginata* | E | MW589515.1 | 48151 | 38126 | 79.18 | 343 |  |  |  |
| *Goodyera nankoensis* | E | MW589516.1 | 48054 | 37967 | 79.01 | 342 |  |  |  |
| *Goodyera procera* | E | KT886429.1 | 31794 | 26774 | 84.21 | 347 |  |  |  |
| *Goodyera pubescens* | E | NC_064116.1 | 31933 | 26935 | 84.35 | 343 |  |  |  |
| *Goodyera repens* | E | MW589518.1 | 31933 | 31294 | 98.00 | 338 |  |  |  |
| *Goodyera rosulacea* | E | MN200390.1 | 48118 | 41112 | 85.44 | 342 |  |  |  |
| *Goodyera schlechtendaliana* | E | KT886431.1 | 48582 | 42159 | 86.78 | 349 |  |  |  |
| *Goodyera seikomontana* | E | MW589520.1 | 48116 | 45340 | 94.23 | 339 |  |  |  |
| *Goodyera striata* | E | NC_064117.1 | 48196 | 46148 | 95.75 | 338 |  |  |  |
| *Goodyera velutina* | E | KT886432.1 | 48428 | 43484 | 89.79 | 344 |  |  |  |
| *Goodyera yangmeishanensis* | E | MW589522.1 | 48370 | 43872 | 90.70 | 344 |  |  |  |
| *Goodyera yunnanensis* | E | NC_081512.1 | 35353 | 34646 | 98.00 | 365 |  |  |  |
| *Grammatophyllum scriptum* | E | NC_067759.1 | 23518 | 22918 | 97.45 | 357 |  |  |  |
| *Haraella retrocalla* | E | NC_072971.1 | 48466 | 44773 | 92.38 | 348 |  |  |  |
| *Hemipilia yajiangensis* | E | NC_067080.1 | 48158 | 39490 | 82.00 | 343 |  |  |  |
| *Herpysma longicaulis* | E | MW589523.1 | 48063 | 42425 | 88.27 | 341 |  |  |  |
| *Hetaeria anomala* | E | MW589524.1 | 48185 | 42446 | 88.09 | 341 |  |  |  |
| *Hetaeria oblongifolia* | E | MW589525.1 | 48188 | 44347 | 92.03 | 340 |  |  |  |
| *Hetaeria youngsayei* | E | MW589526.1 | 48226 | 44691 | 92.67 | 390 |  |  |  |
| *Holcoglossum omeiense* | E | NC_064528.1 | 40807 | 33462 | 82.00 | 386 |  |  |  |
| *Holcoglossum tsii* | E | MK836106.1 | 42114 | 41272 | 98.00 | 314 |  |  |  |
| *Lankesterella ceracifolia* | E | MN597434.1 | 42012 | 35500 | 84.50 | 363 |  |  |  |
| *Liparis auriculata* | E | MN200365.1 | 28428 | 26418 | 92.93 | 371 |  |  |  |
| *Liparis bootanensis* | E | MN627759.1 | 48822 | 44472 | 91.09 | 361 |  |  |  |
| *Liparis ferruginea* | E | NC_072702.1 | 48546 | 42109 | 86.74 | 361 |  |  |  |
| *Liparis japonica* | E | MK886513.1 | 48618 | 37791 | 77.73 | 364 |  |  |  |
| *Liparis kumokiri* | E | MN687902.1 | 14834 | 14398 | 97.06 | 365 |  |  |  |
| *Liparis loeselii* | E | MF374688.1 | 14920 | 14602 | 97.87 | 370 |  |  |  |
| *Liparis luteola* | E | MW683327.1 | 37902 | 37095 | 97.87 | 363 |  |  |  |
| *Liparis makinoana* | E | MN200368.1 | 37886 | 37405 | 98.73 | 362 |  |  |  |
| *Liparis nervosa* | E | MN641753.1 | 12753 | 12591 | 98.73 | 361 |  |  |  |
| *Liparis pauliana* | E | MN686022.1 | 27642 | 26829 | 97.06 | 368 |  |  |  |
| *Liparis pingtaoi* | E | MN627758.1 | 47361 | 46352 | 97.87 | 364 |  |  |  |
| *Liparis rostrata* | E | MW683328.1 | 47262 | 40995 | 86.74 | 371 |  |  |  |
| *Liparis viridiflora* | E | MT968779.1 | 48202 | 46211 | 95.87 | 363 |  |  |  |
| *Liparis yongnoana* | E | MK801140.1 | 47422 | 46976 | 99.06 | 352 |  |  |  |
| *Listera fugongensis* | E | NC_030711.1 | 47318 | 37727 | 79.73 | 340 |  |  |  |
| *Ludisia discolor* | E | KU578274.1 | 47254 | 37203 | 78.73 | 389 |  |  |  |
| *Luisia hancockii* | E | NC_080893.1 | 47345 | 41067 | 86.74 | 339 |  |  |  |
| *Macodes petola* | E | MW589527.1 | 43805 | 35806 | 81.74 | 341 |  |  |  |
| *Macodes sanderiana* | E | MW589528.1 | 43834 | 36527 | 83.33 | 354 |  |  |  |
| *Malaxis monophyllos* | E | NC_083997.1 | 46968 | 41238 | 87.80 | 363 |  |  |  |
| *Masdevallia coccinea* | E | KP205432.1 | 46718 | 38930 | 83.33 | 362 |  |  |  |
| *Masdevallia picturata* | E | KJ566305.1 | 47353 | 45587 | 96.27 | 379 |  |  |  |
| *Mengzia foliosa* | E | NC_082055.1 | 47248 | 39372 | 83.33 | 320 |  |  |  |
| *Myoxanthus exasperatus* | E | MW375127.1 | 16739 | 15248 | 91.09 | 386 |  |  |  |
| *Neofinetia falcata cultivar CheongSan* | E | NC_036372.1 | 16725 | 15235 | 91.09 | 386 |  |  |  |
| *Neofinetia richardsiana* | E | KT726908.1 | 48208 | 41816 | 86.74 | 337 |  |  |  |
| *Neottia cordata* | E | MH590356.1 | 48433 | 44776 | 92.45 | 352 |  |  |  |
| *Neottia fugongensis* | E | KU551270.1 | 20940 | 17954 | 85.74 | 353 |  |  |  |
| *Neottia japonica* | E | MH321183.1 | 32072 | 31665 | 98.73 | 361 |  |  |  |
| *Neottia ovata* | E | KU551271.1 | 63500 | 52915 | 83.33 | 361 |  |  |  |
| *Neottia pinetorum* | E | KU551269.1 | 19345 | 18776 | 97.06 | 352 |  |  |  |
| *Neottia suzukii* | E | MH321185.1 | 48377 | 41043 | 84.84 | 337 |  |  |  |
| *Nervilia aragoana* | E | NC_066070.1 | 48244 | 46444 | 96.27 | 337 |  |  |  |
| *Nervilia fordii* | E | NC_066071.1 | 48654 | 38792 | 79.73 | 324 |  |  |  |
| *Neuwiedia singapureana* | E | KM244735.1 | 48437 | 42528 | 87.80 | 324 |  |  |  |
| *Neuwiedia zollingeri var* | E | LC199503.1 | 29713 | 29336 | 98.73 | 392 |  |  |  |
| *Nothodoritis zhejiangensis* | E | MW646088.1 | 29638 | 29007 | 97.87 | 357 |  |  |  |
| *Oberonia integerrima* | E | NC_084378.1 | 49829 | 42275 | 84.84 | 342 |  |  |  |
| *Oberonia japonica* | E | KX871235.1 | 49747 | 43151 | 86.74 | 349 |  |  |  |
| *Oberonia seidenfadenii* | E | MN414241.1 | 50146 | 45066 | 89.87 | 358 |  |  |  |
| *Oberonioides microtatantha* | E | MT559316.1 | 50067 | 47474 | 94.82 | 367 |  |  |  |
| *Octomeria grandiflora* | E | MW375128.1 | 50297 | 43628 | 86.74 | 345 |  |  |  |
| *Odontochilus elwesii* | E | MN880629.1 | 50395 | 45290 | 89.87 | 343 |  |  |  |
| *Odontochilus tortus* | E | MW589530.1 | 49936 | 48073 | 96.27 | 341 |  |  |  |
| *Odontochilus yakushimensis* | E | MW683330.1 | 45014 | 39045 | 86.74 | 361 |  |  |  |
| *Oncidium Gower Ramsey* | E | GQ324949.1 | 50259 | 48696 | 96.89 | 355 |  |  |  |
| *Ornithochilus yingjiangensis* | E | NC_067617.1 | 50038 | 43403 | 86.74 | 372 |  |  |  |
| *Oxystophyllum changjiangense WMT2000* | E | LC579430.1 | 49917 | 47855 | 95.87 | 362 |  |  |  |
| *Pabstiella mirabilis* | E | MW375130.1 | 49958 | 42384 | 84.84 | 364 |  |  |  |
| *Paraphalaenopsis denevei* | E | NC_082047.1 | 52504 | 46099 | 87.80 | 389 |  |  |  |
| *Paraphalaenopsis labukensis* | E | NC_082046.1 | 30422 | 29287 | 96.27 | 391 |  |  |  |
| *Paraphalaenopsis laycockii cultivar Semi-alba* | E | NC_082048.1 | 18109 | 17577 | 97.06 | 347 |  |  |  |
| *Pecteilis hawkesiana* | E | NC_082102.1 | 22589 | 21925 | 97.06 | 350 |  |  |  |
| *Pecteilis susannae* | E | NC_082101.1 | 44699 | 43747 | 97.87 | 395 |  |  |  |
| *Pelatantheria scolopendrifolia* | E | MG752972.1 | 51213 | 44422 | 86.74 | 382 |  |  |  |
| *Pennilabium yunnanense* | E | NC_067615.1 | 35754 | 33055 | 92.45 | 379 |  |  |  |
| *Phaius antoninae* | E | NC_084156.1 | 37883 | 32860 | 86.74 | 379 |  |  |  |
| *Phaius columnaris* | E | NC_084157.1 | 29262 | 28053 | 95.87 | 378 |  |  |  |
| *Phaius flavus* | E | NC_067591.1 | 17631 | 15845 | 89.87 | 381 |  |  |  |
| *Phaius hainanensis* | E | MW463050.1 | 33024 | 28018 | 84.84 | 379 |  |  |  |
| *Phaius mishmensis* | E | NC_084158.1 | 26043 | 21702 | 83.33 | 379 |  |  |  |
| *Phaius philippinensis* | E | NC_084159.1 | 69865 | 66980 | 95.87 | 378 |  |  |  |
| *Phaius takeoi* | E | NC_084160.1 | 15170 | 14977 | 98.73 | 378 |  |  |  |
| *Phaius tancarvilleae* | E | MN708349.1 | 17056 | 16896 | 99.06 | 378 |  |  |  |
| *Phalaenopsis braceana* | E | NC_084161.1 | 16712 | 14496 | 86.74 | 398 |  |  |  |
| *Phalaenopsis deliciosa* | E | NC_072257.1 | 19736 | 17328 | 87.80 | 393 |  |  |  |
| *Phalaenopsis hybrid cultivar cultivar Tiny Star* | E | NC_067938.1 | 23798 | 21678 | 91.09 | 400 |  |  |  |
| *Phalaenopsis japonica* | E | KJ944326.1 | 50544 | 43842 | 86.74 | 391 |  |  |  |
| *Phalaenopsis lobbii* | E | MN200373.1 | 15454 | 15000 | 97.06 | 386 |  |  |  |
| *Phalaenopsis lowii* | E | MT830847.1 | 27945 | 23960 | 85.74 | 399 |  |  |  |
| *Phalaenopsis malipoensis* | E | MN385684.1 | 19108 | 18865 | 98.73 | 386 |  |  |  |
| *Phalaenopsis mannii* | E | NC_060875.1 | 67457 | 57231 | 84.84 | 391 |  |  |  |
| *Phalaenopsis pulcherrima* | E | MT822270.1 | 73469 | 62331 | 84.84 | 399 |  |  |  |
| *Phalaenopsis stobartiana* | E | MG459020.1 | 13714 | 13422 | 97.87 | 398 |  |  |  |
| *Phalaenopsis subparishii* | E | MW531729.1 | 10808 | 10362 | 95.87 | 390 |  |  |  |
| *Phalaenopsis wilsonii* | E | OR918892.1 | 77257 | 67013 | 86.74 | 397 |  |  |  |
| *Phalaenopsis zhejiangensis* | E | OP235489.1 | 34151 | 29623 | 86.74 | 392 |  |  |  |
| *Phloeophila pelecaniceps* | E | MZ326749.1 | 11800 | 11453 | 97.06 | 373 |  |  |  |
| *Pholidota articulata* | E | OR909688.1 | 22298 | 19341 | 86.74 | 374 |  |  |  |
| *Pholidota imbricata* | E | NC_050859.1 | 60452 | 51287 | 84.84 | 378 |  |  |  |
| *Pholidota longipes* | E | NC_048955.1 | 22645 | 21710 | 95.87 | 377 |  |  |  |
| *Pholidota yunnanensis* | E | NC_057974.1 | 30688 | 29786 | 97.06 | 373 |  |  |  |
| *Pleione albiflora* | E | MN686021.1 | 11567 | 11458 | 99.06 | 376 |  |  |  |
| *Pleione arunachalensis* | E | MT952838.1 | 33017 | 29365 | 88.94 | 376 |  |  |  |
| *Pleione aurita* | E | MT954962.1 | 25126 | 24807 | 98.73 | 377 |  |  |  |
| *Pleione bulbocodioides* | E | MW014291.1 | 10385 | 9008 | 86.74 | 377 |  |  |  |
| *Pleione coronaria* | E | KY849819.1 | 24408 | 23690 | 97.06 | 376 |  |  |  |
| *Pleione dilamellata* | E | MT954963.1 | 67404 | 60994 | 90.49 | 377 |  |  |  |
| *Pleione formosana* | E | MW007167.1 | 18917 | 18677 | 98.73 | 376 |  |  |  |
| *Pleione grandiflora* | E | MK361027.1 | 23456 | 20346 | 86.74 | 377 |  |  |  |
| *Pleione hookeriana* | E | MW007161.1 | 23925 | 23621 | 98.73 | 375 |  |  |  |
| *Pleione humilis* | E | MW007162.1 | 68181 | 58458 | 85.74 | 374 |  |  |  |
| *Pleione jinhuana* | E | MT954964.1 | 99652 | 86438 | 86.74 | 376 |  |  |  |
| *Pleione limprichtii* | E | MW007163.1 | 39122 | 37506 | 95.87 | 376 |  |  |  |
| *Pleione maculata* | E | MT954965.1 | 94982 | 82387 | 86.74 | 374 |  |  |  |
| *Pleione microphylla* | E | MT954966.1 | 35520 | 32838 | 92.45 | 377 |  |  |  |
| *Pleione praecox* | E | MW007168.1 | 54500 | 46238 | 84.84 | 372 |  |  |  |
| *Pleione saxicola* | E | MW007164.1 | 26810 | 23845 | 88.94 | 374 |  |  |  |
| *Pleione scopulorum* | E | MT954967.1 | 34285 | 32509 | 94.82 | 374 |  |  |  |
| *Pleione vietnamensis* | E | MT954968.1 | 43785 | 38443 | 87.80 | 374 |  |  |  |
| *Pleione x taliensis* | E | MW007165.1 | 15349 | 15205 | 99.06 | 378 |  |  |  |
| *Pleione yunnanensis* | E | MZ958822.2 | 13637 | 13464 | 98.73 | 377 |  |  |  |
| *Pleurothallis matudana* | E | MW007166.1 | 98174 | 85156 | 86.74 | 372 |  |  |  |
| *Pogonia japonica* | E | OR909687.1 | 18566 | 18171 | 97.87 | 303 |  |  |  |
| *Pogonia minor* | E | MN200371.1 | 17828 | 15640 | 87.73 | 303 |  |  |  |
| *Polystachya adansoniae* | E | MN200372.1 | 11354 | 10737 | 94.57 | 363 |  |  |  |
| *Polystachya bennettiana* | E | NC_066146.1 | 25454 | 23483 | 92.26 | 362 |  |  |  |
| *Polystachya concreta* | E | NC_066148.1 | 49967 | 45701 | 91.46 | 362 |  |  |  |
| *Polystachya dendrobiiflora* | E | NC_066150.1 | 76738 | 65058 | 84.78 | 364 |  |  |  |
| *Polystachya modesta* | E | NC_066149.1 | 40676 | 37515 | 92.23 | 357 |  |  |  |
| *Polystachya tenuissima* | E | NC_066147.1 | 29141 | 27141 | 93.14 | 348 |  |  |  |
| *Ponerorchis gracilis* | E | MN200376.1 | 30262 | 26696 | 88.22 | 328 |  |  |  |
| *Prescottia stachyodes* | E | MN597433.1 | 19417 | 17900 | 92.19 | 385 |  |  |  |
| *Renanthera citrina* | E | OP131935.1 | 39417 | 35429 | 89.88 | 393 |  |  |  |
| *Renanthera coccinea* | E | OP131934.1 | 26367 | 17553 | 66.57 | 366 |  |  |  |
| *Restrepia trichoglossa* | E | OR713739.1 | 13909 | 10240 | 73.62 | 339 |  |  |  |
| *Rhomboda fanjingensis* | E | MW683331.1 | 68979 | 53727 | 77.89 | 339 |  |  |  |
| *Rhomboda lanceolata* | E | MW589531.1 | 21321 | 13969 | 65.52 | 340 |  |  |  |
| *Rhomboda moulmeinensis* | E | MN880625.1 | 41189 | 38681 | 93.91 | 359 |  |  |  |
| *Satyrium nepalense var* | E | MN497244.1 | 13317 | 10685 | 80.24 | 369 |  |  |  |
| *Scaphosepalum antenniferum* | E | OR713741.1 | 82774 | 57360 | 69.30 | 375 |  |  |  |
| *Sobralia callosa* | E | KM032623.1 | 51271 | 42621 | 83.13 | 371 |  |  |  |
| *Spathoglottis aurea* | E | NC_079577.1 | 30184 | 22453 | 74.39 | 365 |  |  |  |
| *Specklinia grobyi* | E | OR713740.1 | 12018 | 10672 | 88.80 | 323 |  |  |  |
| *Stelis grandiflora* | E | MW375129.1 | 30855 | 29844 | 96.72 | 367 |  |  |  |
| *Stelis kefersteiniana* | E | OR909686.1 | 54740 | 13400 | 24.48 | 367 |  |  |  |
| *Stelis montserratii* | E | MW375125.1 | 14919 | 13831 | 92.71 | 363 |  |  |  |
| *Stichorkis gibbosa* | E | NC_063087.1 | 75423 | 65763 | 87.19 | 371 |  |  |  |
| *Tainia acuminata* | E | NC_060558.1 | 21271 | 19875 | 93.44 | 374 |  |  |  |
| *Tainia cordifolia* | E | MN577470.1 | 52336 | 15809 | 30.21 | 372 |  |  |  |
| *Tainia dunnii* | E | MN641754.1 | 19913 | 10000 | 50.22 | 360 |  |  |  |
| *Thecopus maingayi* | E | NC_067760.1 | 19666 | 17247 | 87.70 | 388 |  |  |  |
| *Thrixspermum amplexicaule* | E | MW574621.1 | 24028 | 22395 | 93.20 | 382 |  |  |  |
| *Thrixspermum japonicum* | E | NC_035831.2 | 30883 | 29228 | 94.64 | 378 |  |  |  |
| *Thunia alba* | E | MN606292.1 | 39813 | 25024 | 62.85 | 381 |  |  |  |
| *Thuniopsis cleistogama* | E | OL809660.1 | 76527 | 59553 | 77.82 | 392 |  |  |  |
| *Trichoglottis cirrhifera* | E | NC_083135.1 | 60937 | 54342 | 89.18 | 392 |  |  |  |
| *Trichoglottis ionosma* | E | NC_083136.1 | 12476 | 12247 | 98.16 | 392 |  |  |  |
| *Trichoglottis lanceolaria* | E | NC_083137.1 | 80144 | 52998 | 66.13 | 392 |  |  |  |
| *Trichoglottis orchidea* | E | NC_083138.1 | 91740 | 34662 | 37.78 | 392 |  |  |  |
| *Trichoglottis subviolacea* | E | NC_083139.1 | 90630 | 85670 | 94.53 | 379 |  |  |  |
| *Tridactyle tridactylites* | E | MW760855.1 | 24156 | 17228 | 71.32 | 397 |  |  |  |
| *Vanda coerulea* | E | MN711649.1 | 52138 | 46726 | 89.62 | 396 |  |  |  |
| *Vanda coerulescens* | E | MN711650.1 | 16373 | 12824 | 78.32 | 395 |  |  |  |
| *Vanda concolor* | E | MK836105.1 | 26122 | 16866 | 64.57 | 386 |  |  |  |
| *Vanda falcata cultivar CheongSan* | E | KT726907.1 | 61579 | 42239 | 68.59 | 386 |  |  |  |
| *Vanda falcata* | E | KT726909.1 | 97482 | 94173 | 96.61 | 302 |  |  |  |
| *Vrydagzynea nuda* | E | MW589534.1 | 45859 | 36206 | 78.95 | 337 |  |  |  |
| *Vrydagzynea tristriata* | E | MW589535.1 | 15267 | 11788 | 77.21 | 341 |  |  |  |
| *Aspidogyne longicornu* | O | MN597437.1 | 48466 | 41569 | 85.77 | 341 | 340.98 (349.5; 329.0–363.0; 147.0–391.0) | 86.55 |  |
| *Chiloglottis cornuta* | O | MW589505.1 | 12086 | 10304 | 85.26 | 323 |  |  |  |
| *Corybas cheesemanii* | O | JX087681.1 | 38371 | 34292 | 89.37 | 328 |  |  |  |
| *Corybas diemenicus* | O | MK876367.1 | 13949 | 11389 | 81.65 | 322 |  |  |  |
| *Corybas iridescens* | O | MK867776.1 | 23915 | 18472 | 77.24 | 323 |  |  |  |
| *Corybas macranthus* | O | MK867777.1 | 49791 | 44583 | 89.54 | 324 |  |  |  |
| *Corybas obscurus* | O | MK876368.1 | 48427 | 37153 | 76.72 | 327 |  |  |  |
| *Corybas taliensis* | O | MK876365.1 | 47342 | 37268 | 78.72 | 323 |  |  |  |
| *Corybas vitreus* | O | MN783368.1 | 44807 | 34891 | 77.87 | 327 |  |  |  |
| *Cremastra aphylla* | O | MK848868.1 | 46286 | 35015 | 75.65 | 367 |  |  |  |
| *Cremastra appendiculata* | O | MW683324.1 | 46279 | 40536 | 87.59 | 367 |  |  |  |
| *Cremastra unguiculata* | O | MG925366.1 | 34313 | 33716 | 98.26 | 369 |  |  |  |
| *Cystorchis variegata* | O | NC_084190.1 | 41492 | 34857 | 84.01 | 357 |  |  |  |
| *Dactylorhiza majalis* | O | MK984209.1 | 47973 | 41842 | 87.22 | 357 |  |  |  |
| *Dactylorhiza viridis* | O | MN824430.1 | 25009 | 23421 | 93.65 | 332 |  |  |  |
| *Danhatchia australis* | O | MN251184.1 | 96565 | 82515 | 85.45 | 187 |  |  |  |
| *Danxiaorchis yangii* | O | OQ540481.2 | 53814 | 40737 | 75.70 | 363 |  |  |  |
| *Dossinia marmorata* | O | MW589508.1 | 27917 | 22833 | 81.79 | 363 |  |  |  |
| *Galearis cyclochila* | O | MN200388.1 | 96591 | 86739 | 89.80 | 147 |  |  |  |
| *Gymnadenia conopsea* | O | MN200391.1 | 48044 | 47083 | 98.00 | 357 |  |  |  |
| *Gymnadenia crassinervis* | O | MW322684.1 | 48171 | 44674 | 92.74 | 358 |  |  |  |
| *Habenaria aitchisonii* | O | MW316693.1 | 18934 | 18555 | 98.00 | 346 |  |  |  |
| *Habenaria chejuensis* | O | MN200392.1 | 28942 | 25947 | 89.65 | 349 |  |  |  |
| *Habenaria ciliolaris* | O | MN495954.1 | 48717 | 44956 | 92.28 | 349 |  |  |  |
| *Habenaria dentata* | O | OK012095.1 | 48778 | 39998 | 82.00 | 345 |  |  |  |
| *Habenaria flagellifera* | O | MN200366.1 | 48283 | 44420 | 92.00 | 348 |  |  |  |
| *Habenaria pantlingiana* | O | KJ524104.1 | 48351 | 37878 | 78.34 | 350 |  |  |  |
| *Habenaria radiata* | O | KX871237.1 | 48108 | 47146 | 98.00 | 376 |  |  |  |
| *Hancockia uniflora* | O | OK012601.1 | 48114 | 43144 | 89.67 | 387 |  |  |  |
| *Microtis unifolia* | O | MK796239.1 | 46976 | 41245 | 87.80 | 368 |  |  |  |
| *Orchis militaris* | O | NC_084283.1 | 49989 | 43890 | 87.80 | 367 |  |  |  |
| *Oreorchis patens* | O | MN200369.1 | 49857 | 42299 | 84.84 | 391 |  |  |  |
| *Panisea uniflora* | O | NC_052740.1 | 50173 | 43520 | 86.74 | 327 |  |  |  |
| *Platanthera chlorantha* | O | KM032625.1 | 56659 | 49146 | 86.74 | 353 |  |  |  |
| *Platanthera japonica* | O | MK937914.1 | 90411 | 76705 | 84.84 | 353 |  |  |  |
| *Platanthera mandarinorum* | O | MG925368.2 | 14545 | 14408 | 99.06 | 353 |  |  |  |
| *Platanthera ussuriensis* | O | MN200370.1 | 16056 | 15393 | 95.87 | 354 |  |  |  |
| *Spiranthes sinensis* | O | MK936427.1 | 13089 | 10864 | 83.00 | 366 |  |  |  |
| *Zeuxine affinis* | O | MW589536.1 | 72672 | 65452 | 90.06 | 342 |  |  |  |
| *Zeuxine flava* | O | MW589537.1 | 23528 | 19592 | 83.27 | 336 |  |  |  |
| *Zeuxine nervosa* | O | MW589538.1 | 27860 | 23762 | 85.29 | 336 |  |  |  |
| *Galeola lindleyana* | V | MW528436.1 | 96886 | 86742 | 89.53 | 395 | 331.71 (311; 305.5–354.5; 296.0–395.0) | 83.34 |  |
| *Palmorchis pabstii* | V | MH590357.1 | 50211 | 42599 | 84.84 | 373 |  |  |  |
| *Vanilla aphylla* | V | LC085348.1 | 30374 | 26350 | 86.75 | 296 |  |  |  |
| *Vanilla madagascariensis* | V | MN200374.1 | 20800 | 15380 | 73.94 | 311 |  |  |  |
| *Vanilla planifolia* | V | KJ566306.1 | 20090 | 17296 | 86.09 | 309 |  |  |  |
| *Vanilla pompona* | V | MF197310.1 | 27077 | 25844 | 95.45 | 302 |  |  |  |
| *Vanilla somae* | V | NC_079955.1 | 46752 | 31224 | 66.79 | 336 |  |  |  |

**Suppl. Table 2.** In silico evaluation of plastid bait recovery across Orchidaceae. For each species, we reported taxonomic affiliation, NCBI accession numbers, numbers of simulated paired-end reads, mapping efficiency, and the number of plastid baits. The plastid baits recovery was summarized as mean, median, interquartile range (IQR), and range. Mean plastid mapping percentages were reported for each subfamily. A=Apostasioideae; C=Cypripedioideae; E=Epidendroideae; O=Orchidoideae;V=Vanilloideae.

**Bait	A	C	E	O	V	N**

```
Ophrys-gene4954                       1    1    1    1    1   5
Ophrys-gene4955                       1    1    1    1    1   5
Ophrys-gene4944                       1    1    1    1    1   5
Ophrys-gene4945                       1    1    1    1    1   5
Ophrys-gene4952                       1    1    1    1    1   5
Ophrys-gene5234                       0    0    1    1    0   2
Ophrys-gene6637                       1    1    1    1    1   5
Ophrys-gene5233                       1    1    0    0    0   2
Ophrys-gene7662                       1    0    0    0    0   1
Ophrys-gene8322                       1    1    1    1    1   5
Ophrys-gene8005                       1    0    0    0    0   1
Ophrys-gene8583                       1    1    1    1    1   5
Ophrys-gene8917                       1    1    1    1    1   5
Ophrys-gene8901                       1    1    1    1    1   5
Ophrys-gene8975                       1    0    0    0    0   1
Ophrys-gene8976                       1    0    0    1    1   3
Ophrys-gene9302                       1    0    0    1    0   2
Ophrys-gene9779                       1    1    1    1    1   5
Ophrys-gene10910                      1    1    1    1    1   5
Ophrys-gene11406                      1    1    1    1    1   5
Ophrys-gene11522                      1    0    0    0    0   1
Ophrys-gene11767                      1    0    0    1    1   3
Ophrys-gene11771                      0    0    1    1    0   2
Ophrys-gene12115                      1    1    1    1    1   5
Ophrys-gene20341                      1    0    0    1    0   2
Ophrys-gene20626                      1    1    1    1    1   5
Ophrys-gene21735                      1    1    1    1    1   5
Ophrys-gene22667                      1    0    0    1    0   2
Ophrys-gene23058                      1    1    1    1    1   5
Ophrys-gene23221                      1    0    1    1    0   3
Ophrys-gene24273                      1    1    1    1    1   5
Ophrys-gene25726                      1    0    0    1    0   2
Ophrys-gene25727                      0    0    0    0    0   0
Ophrys-gene25766                      1    0    1    1    0   3
Ophrys-gene25767                      1    0    0    1    0   2
Ophrys-gene36129                      1    0    1    1    0   3
Ophrys-gene38177                      0    0    1    1    0   2
Ophrys-gene39281                      1    1    1    1    0   4
Ophrys-gene40733                      1    0    0    1    0   2
Ophrys-gene45678                      1    1    0    0    0   2
Ophrys-gene46706                      1    0    0    1    0   2
Ophrys-gene46813                      0    0    1    1    0   2
Ophrys-gene48221                      0    0    0    1    0   1
Ophrys-gene49935                      1    0    1    1    0   3
Ophrys-gene50732                      1    1    0    1    0   3
Ophrys-gene51277                      1    1    1    1    1   5
Ophrys-gene52379                      1    1    1    1    1   5
Ophrys-gene53507                      0    0    1    1    0   2
Ophrys-gene53983                      1    1    1    1    0   4
Ophrys-gene54812                      1    0    1    0    0   2
Ophrys-gene59939                      1    1    1    1    1   5
Ophrys-gene62146                      1    1    1    1    1   5
Ophrys-gene62981                      0    1    1    1    0   3
Ophrys-gene65863                      0    0    0    1    0   1
Ophrys-gene67099                      1    1    1    1    1   5
Ophrys-gene68627                      1    1    1    1    1   5
Ophrys-gene68702                      1    1    1    1    1   5
Ophrys-gene70981                      1    1    1    1    1   5
Ophrys-gene70988                      1    1    1    1    1   5
Ophrys-gene71243                      1    0    0    0    0   1
Ophrys-gene71636                      1    0    1    1    0   3
Ophrys-gene71690                      0    0    1    1    0   2
Ophrys-gene71699                      1    0    0    0    0   1
Ophrys-gene72694                      0    0    0    1    0   1
Ophrys-gene73970                      1    0    1    0    0   2
Ophrys-gene74982                      1    1    1    1    1   5
Ophrys-gene76255                      1    0    1    1    1   4
Ophrys-gene76891                      0    0    1    0    0   1
Ophrys-gene76915                      1    1    1    1    1   5
Ophrys-gene77829                      1    0    0    1    0   2
Ophrys-gene79456                      1    1    1    1    1   5
Ophrys-gene79457                      1    1    1    1    1   5
Ophrys-gene79464                      1    0    0    1    0   2
Ophrys-gene81681                      1    1    1    1    1   5
Ophrys-gene81682                      1    1    1    1    1   5
Ophrys-gene81937                      1    0    0    0    1   2
Ophrys-gene84021                      1    1    1    1    1   5
Ophrys-gene86266                      1    1    1    0    0   3
Ophrys-gene86850                      1    0    1    1    0   3
Ophrys-gene86851                      1    1    1    1    1   5
Ophrys-gene88647                      1    1    1    1    1   5
Ophrys-gene91042                      1    0    0    1    0   2
Ophrys-gene91043                      1    0    1    1    0   3
Ophrys-gene91044                      1    1    1    1    1   5
Ophrys-gene91054                      1    0    0    1    0   2
Ophrys-gene93862                      1    1    0    1    1   4
Ophrys-gene93863                      1    1    1    1    1   5
Ophrys-gene93906                      1    1    1    1    1   5
Ophrys-gene93907                      0    0    0    1    0   1
Ophrys-gene93995                      1    1    0    1    0   3
Ophrys-gene96410                      0    0    0    1    0   1
Ophrys-gene96411                      1    1    1    1    1   5
Ophrys-gene96432                      0    0    0    1    0   1
Ophrys-gene96454                      1    1    0    0    0   2
Ophrys-gene96473                      1    0    1    1    0   3
Ophrys-gene96474                      0    1    0    1    0   2
Ophrys-gene96727                      1    0    0    0    0   1
Ophrys-gene97673                      1    0    0    1    0   2
Ophrys-gene100623                     1    0    1    1    0   3
Ophrys-gene100620                     1    1    1    1    1   5
Ophrys-gene100858                     0    0    0    1    0   1
Ophrys-gene102227                     1    0    0    1    0   2
Ophrys-gene102228                     0    0    0    0    0   0
Ophrys-gene102640                     1    0    0    1    0   2
Ophrys-gene105584                     1    1    1    1    1   5
Ophrys-gene105587                     1    0    1    1    1   4
Ophrys-gene106580                     1    1    1    1    1   5
Ophrys-gene108162                     1    1    1    1    1   5
Ophrys-gene111994                     0    0    1    1    0   2
Ophrys-gene112879                     1    1    1    1    0   4
Ophrys-gene115854                     1    1    1    1    1   5
Ophrys-gene116207                     1    0    1    1    0   3
Ophrys-gene118628                     1    0    0    1    0   2
Ophrys-gene118629                     1    0    0    0    0   1
Ophrys-gene118630                     0    0    0    0    0   0
Ophrys-gene120144                     1    0    0    1    0   2
Ophrys-gene126480                     1    0    1    0    0   2
Ophrys-gene127057                     1    1    1    1    1   5
Ophrys-gene131325                     1    1    0    1    0   3
Ophrys-gene131886                     1    0    0    1    0   2
Ophrys-gene134879                     0    0    1    1    0   2
Ophrys-gene137144                     1    0    1    1    0   3
Ophrys-gene140679                     1    0    1    1    0   3
Ophrys-gene143301                     0    0    0    0    0   0
Ophrys-gene143302                     1    0    1    1    0   3
Ophrys-gene144746                     1    0    0    0    0   1
Ophrys-gene145226                     1    1    0    1    0   3
Ophrys-gene145227                     1    1    1    1    1   5
Ophrys-gene146518                     1    1    1    1    1   5
Ophrys-gene146642                     1    0    0    1    0   2
Ophrys-gene150246                     1    0    0    1    0   2
Ophrys-gene154928                     1    0    1    1    0   3
Ophrys-gene154956                     1    1    1    1    1   5
Ophrys-gene159118                     1    1    1    1    1   5
Ophrys-gene160736                     1    0    0    1    0   2
Ophrys-gene168066                     1    0    1    1    0   3
Ophrys-gene168515                     0    0    1    0    0   1
Ophrys-gene173577                     1    1    1    1    1   5
Ophrys-gene173619                     1    0    0    1    0   2
Ophrys-gene173614                     1    1    0    1    0   3
Ophrys-gene173821                     1    0    0    0    0   1
Ophrys-gene176714                     1    1    1    0    0   3
Ophrys-gene178519                     1    1    1    1    1   5
Ophrys-gene178520                     1    0    0    0    0   1
Ophrys-gene178624                     1    1    0    1    0   3
Ophrys-gene178670                     1    0    0    1    0   2
Ophrys-gene178715                     1    0    0    1    0   2
Ophrys-gene178829                     1    0    0    1    0   2
Ophrys-gene180202                     1    1    1    1    1   5
Ophrys-gene182426                     1    1    1    1    1   5
Ophrys-gene183092                     1    0    1    1    0   3
Ophrys-gene190710                     0    1    1    1    0   3
Ophrys-gene190711                     1    1    1    1    0   4
Ophrys-gene190758                     1    1    1    1    1   5
Ophrys-gene194061                     1    1    1    1    1   5
Ophrys-gene195213                     1    0    0    1    0   2
Ophrys-gene197601                     1    1    1    1    1   5
Ophrys-gene197602                     1    1    1    1    1   5
Ophrys-gene197603                     1    1    1    1    1   5
Ophrys-gene197604                     1    1    1    1    1   5
Ophrys-gene199064                     0    0    0    1    0   1
Ophrys-gene203611                     1    1    1    1    1   5
Ophrys-gene203633                     1    0    0    1    0   2
Ophrys-gene203694                     0    0    1    0    0   1
Ophrys-gene203777                     1    1    1    0    0   3
Ophrys-gene206123                     1    0    1    1    0   3
Ophrys-gene207971                     1    1    1    1    1   5
Ophrys-gene207972                     1    1    1    1    1   5
Ophrys-gene208042                     0    0    1    0    1   2
Ophrys-gene208043                     1    1    1    1    1   5
Ophrys-gene210369                     1    1    1    1    1   5
Ophrys-gene210661                     0    0    0    1    0   1
Ophrys-gene210662                     1    1    1    1    1   5
Ophrys-gene210735                     1    0    0    1    0   2
Ophrys-gene210852                     0    0    0    0    0   0
Ophrys-gene210891                     1    0    0    1    0   2
Ophrys-gene213733                     0    0    1    1    0   2
Ophrys-gene215393                     1    0    0    1    0   2
Ophrys-gene215606                     1    1    1    1    1   5
Ophrys-gene216057                     1    0    1    1    0   3
Ophrys-gene217608                     1    1    1    1    1   5
Ophrys-gene217609                     1    1    1    1    1   5
Ophrys-gene218111                     1    0    0    1    0   2
Ophrys-gene222206                     1    1    1    1    1   5
Ophrys-gene222216                     1    1    1    1    1   5
Ophrys-gene222217                     1    1    1    1    1   5
Ophrys-gene223102                     1    0    1    1    0   3
Ophrys-gene223169                     1    0    1    1    0   3
Ophrys-gene224208                     1    1    1    1    1   5
Ophrys-gene231691                     1    0    1    1    0   3
Ophrys-gene234797                     1    1    1    1    1   5
Ophrys-gene235648                     1    1    1    1    1   5
Ophrys-gene239511                     0    0    0    1    0   1
Ophrys-gene239609                     1    1    1    1    1   5
Ophrys-gene253006                     1    1    1    1    1   5
Ophrys-gene255853                     1    0    0    0    0   1
Ophrys-gene255849                     1    1    1    1    1   5
Ophrys-gene257652                     1    0    0    0    0   1
Ophrys-gene259025                     1    1    1    1    1   5
Ophrys-gene259475                     0    0    0    1    0   1
Ophrys-gene265349                     1    1    1    1    1   5
Ophrys-gene265610                     1    1    1    1    1   5
Ophrys-gene271077                     1    0    1    1    0   3
Ophrys-gene271078                     1    1    1    1    0   4
Ophrys-gene271121                     1    0    1    1    0   3
Ophrys-gene271112                     1    0    0    0    0   1
Ophrys-gene271478                     1    1    1    1    1   5
Ophrys-gene271848                     0    0    0    1    0   1
Ophrys-gene272051                     1    1    1    1    1   5
Ophrys-gene272179                     1    1    1    1    1   5
Ophrys-gene273851                     1    1    1    1    1   5
Ophrys-gene277964                     0    0    0    0    0   0
Ophrys-gene278051                     1    0    1    1    0   3
Ophrys-gene280506                     1    1    1    1    1   5
Ophrys-gene280455                     1    1    1    1    1   5
Ophrys-gene280577                     1    0    0    1    0   2
Ophrys-gene280604                     1    0    0    1    1   3
Ophrys-gene280630                     1    0    0    1    1   3
Ophrys-gene280680                     1    0    0    0    0   1
Ophrys-gene280681                     1    0    1    0    0   2
Ophrys-gene286714                     0    0    1    0    1   2
Ophrys-gene286810                     1    1    1    0    0   3
Ophrys-gene287290                     1    1    0    0    0   2
Ophrys-gene287291                     1    1    1    1    1   5
Ophrys-gene288735                     0    0    1    1    0   2
Ophrys-gene288717                     1    0    0    1    0   2
Ophrys-gene288725                     1    1    1    1    1   5
Ophrys-gene289126                     1    1    1    1    1   5
Ophrys-gene290620                     1    0    1    1    0   3
Ophrys-gene290621                     0    0    0    1    0   1
Ophrys-gene291931                     1    0    0    0    0   1
Ophrys-gene292666                     1    0    0    1    0   2
Ophrys-gene293197                     0    1    1    1    0   3
Ophrys-gene296651                     1    1    1    1    1   5
Ophrys-gene298055                     1    0    0    0    0   1
Ophrys-gene298734                     1    1    1    1    1   5
Ophrys-gene299721                     0    0    0    1    0   1
Ophrys-gene304049                     1    1    1    1    1   5
Ophrys-gene304322                     1    0    0    1    0   2
Ophrys-gene304348                     0    0    0    0    0   0
Ophrys-gene304561                     1    1    1    1    1   5
Ophrys-gene306776                     1    1    1    1    1   5
Ophrys-gene316520                     1    1    0    1    0   3
Ophrys-gene324756                     1    0    0    1    0   2
Ophrys-gene337426                     1    1    1    1    1   5
Ophrys-gene341960                     1    0    0    1    0   2
Ophrys-gene344520                     1    0    0    1    0   2
Ophrys-gene346392                     1    1    1    1    1   5
Ophrys-gene346416                     0    0    0    1    0   1
Ophrys-gene347944                     1    1    1    1    1   5
Ophrys-gene348904                     1    1    1    1    1   5
Ophrys-gene351798                     0    0    0    1    0   1
Ophrys-gene359784                     1    0    1    1    0   3
Ophrys-gene364234                     1    0    0    0    0   1
Ophrys-gene369738                     1    0    1    1    0   3
Ophrys-gene369992                     0    0    1    0    0   1
Ophrys-gene370892                     1    1    1    1    1   5
Ophrys-gene375555                     1    1    1    1    1   5
Ophrys-gene382144                     1    0    1    1    0   3
Ophrys-gene382229                     1    0    0    1    0   2
Ophrys-gene383208                     1    1    1    1    1   5
Ophrys-gene385068                     1    0    0    1    0   2
Ophrys-gene386356                     1    0    0    1    0   2
Ophrys-gene386404                     1    0    0    1    0   2
Ophrys-gene388290                     1    0    1    1    0   3
Ophrys-gene388292                     1    0    1    1    0   3
Ophrys-gene390985                     1    0    0    1    0   2
Ophrys-gene392881                     1    1    1    1    1   5
Ophrys-gene392882                     1    0    0    1    0   2
Ophrys-gene395782                     1    0    1    1    0   3
Ophrys-gene396872                     1    0    0    1    0   2
Ophrys-gene398680                     1    0    1    1    0   3
Ophrys-gene398960                     1    0    1    1    0   3
Ophrys-gene399433                     1    0    0    0    0   1
Ophrys-gene399434                     1    1    1    1    1   5
Ophrys-gene399968                     0    0    0    1    0   1
Ophrys-gene401534                     0    0    0    1    0   1
Ophrys-gene401634                     1    0    1    1    0   3
Ophrys-gene401635                     0    0    0    1    0   1
Ophrys-gene405020                     1    1    1    1    1   5
Ophrys-gene409488                     1    0    0    1    0   2
Ophrys-gene409834                     1    1    0    1    0   3
Ophrys-gene410566                     1    1    1    1    1   5
Ophrys-gene412253                     1    0    0    1    0   2
Ophrys-gene412858                     1    0    0    0    0   1
Ophrys-gene413972                     1    1    1    1    1   5
Ophrys-gene413959                     0    0    1    0    0   1
Ophrys-gene420652                     1    0    0    1    0   2
Ophrys-gene421017                     1    0    0    0    0   1
Ophrys-gene422979                     1    1    1    1    1   5
Ophrys-gene422978                     1    1    1    1    1   5
Ophrys-gene425610                     1    0    0    1    0   2
Ophrys-gene425686                     1    0    1    1    0   3
Ophrys-gene425687                     1    0    1    1    0   3
Ophrys-gene427277                     1    0    1    1    0   3
Ophrys-gene427709                     1    1    1    1    1   5
Ophrys-gene428739                     1    1    1    1    1   5
Ophrys-gene428741                     1    1    1    1    1   5
Ophrys-gene428742                     1    1    1    1    1   5
Ophrys-gene428743                     1    1    1    1    1   5
Ophrys-gene428744                     1    1    1    1    1   5
Ophrys-gene429081                     1    0    1    1    0   3
Ophrys-gene429455                     1    1    1    1    1   5
Ophrys-gene429436                     1    1    1    1    1   5
Ophrys-gene430763                     1    0    0    0    0   1
Ophrys-gene431769                     1    1    1    1    0   4
Ophrys-gene434723                     1    0    0    1    0   2
Ophrys-gene435884                     1    1    1    1    1   5
Ophrys-gene438755                     1    1    1    1    0   4
Ophrys-gene440282                     1    1    1    1    1   5
Ophrys-gene440803                     1    1    1    1    1   5
Ophrys-gene441001                     1    0    0    1    0   2
Ophrys-gene444566                     1    0    0    0    0   1
Ophrys-gene444674                     1    0    0    1    0   2
Ophrys-gene444675                     1    0    0    1    0   2
Ophrys-gene444727                     0    0    0    1    0   1
Ophrys-gene444780                     1    0    0    1    0   2
Ophrys-gene444936                     1    1    1    1    1   5
Ophrys-gene444937                     1    0    0    0    0   1
Ophrys-gene444989                     1    0    0    1    0   2
Ophrys-gene445038                     1    0    0    1    0   2
Ophrys-gene445088                     1    0    0    1    0   2
Ophrys-gene445189                     1    1    0    0    0   2
Ophrys-gene445237                     1    0    0    1    0   2
Ophrys-gene445283                     1    0    0    1    0   2
Ophrys-gene445385                     1    1    1    1    0   4
Ophrys-gene445500                     1    1    1    1    1   5
Ophrys-gene445572                     1    1    1    1    1   5
Ophrys-gene445607                     0    1    0    0    0   1
Ophrys-gene445640                     1    0    1    0    0   2
Ophrys-gene446247                     0    0    1    1    0   2
Ophrys-gene446681                     1    0    0    0    0   1
Ophrys-gene447269                     1    0    0    0    0   1
Ophrys-gene448729                     1    1    1    1    1   5
Ophrys-gene449230                     1    1    1    1    1   5
Ophrys-gene451819                     1    0    0    0    0   1
Ophrys-gene451856                     1    1    1    1    1   5
Ophrys-gene452117                     0    1    1    1    0   3
Ophrys-gene453192                     0    0    1    1    0   2
Ophrys-gene454547                     0    0    0    1    0   1
Ophrys-gene455365                     0    0    0    0    0   0
Ophrys-gene457361                     1    0    0    0    0   1
Ophrys-gene458230                     1    1    1    1    1   5
Ophrys-gene466286                     1    1    1    1    1   5
Ophrys-gene466287                     1    1    1    1    1   5
Ophrys-gene469317                     1    0    0    1    0   2
Ophrys-gene469741                     1    1    1    1    1   5
Ophrys-gene470178                     1    0    0    0    0   1
Ophrys-gene471415                     1    0    1    0    0   2
Ophrys-gene477589                     1    1    1    1    1   5
Ophrys-gene483464                     0    0    1    1    0   2
Ophrys-gene485147                     0    0    1    1    0   2
Ophrys-gene487190                     1    0    1    0    0   2
Ophrys-gene487775                     1    1    1    1    1   5
Ophrys-gene488618                     1    0    1    1    0   3
Ophrys-gene488645                     1    1    1    1    1   5
Ophrys-gene488601                     1    1    1    0    0   3
Ophrys-gene488876                     1    0    0    0    0   1
Ophrys-gene492446                     1    1    0    1    0   3
Ophrys-gene493065                     1    0    0    0    0   1
Ophrys-gene493066                     1    0    0    1    0   2
Ophrys-gene493067                     1    0    1    1    0   3
Ophrys-gene493102                     1    0    0    1    0   2
Ophrys-gene493103                     1    1    1    1    1   5
Ophrys-gene493755                     1    1    1    1    1   5
Ophrys-gene494698                     0    0    1    1    0   2
Ophrys-gene495249                     1    1    1    0    0   3
Ophrys-gene495312                     1    0    1    1    0   3
Ophrys-gene495374                     1    0    1    1    0   3
Ophrys-gene495444                     1    0    1    1    0   3
Ophrys-gene495503                     1    1    1    1    1   5
Ophrys-gene495609                     0    0    0    1    0   1
Ophrys-gene495674                     1    0    1    0    0   2
Ophrys-gene495733                     0    0    0    0    0   0
Ophrys-gene495791                     1    1    1    0    0   3
Ophrys-gene495898                     1    0    0    1    0   2
Ophrys-gene495944                     1    1    1    1    1   5
Ophrys-gene495945                     1    1    1    1    0   4
Ophrys-gene496054                     0    0    0    1    0   1
Ophrys-gene496149                     1    0    1    1    0   3
Ophrys-gene496203                     1    0    1    1    0   3
Ophrys-gene496251                     0    0    0    1    0   1
Ophrys-gene496430                     0    1    1    1    0   3
Ophrys-gene499064                     1    1    1    1    1   5
Ophrys-gene506611                     1    0    0    1    0   2
Ophrys-gene509052                     1    1    1    1    1   5
Ophrys-gene509042                     1    1    1    1    1   5
Ophrys-gene510246                     1    1    1    1    1   5
Ophrys-gene511156                     1    1    1    1    1   5
Ophrys-gene511200                     0    0    1    1    0   2
Ophrys-gene511201                     1    1    1    1    1   5
Ophrys-gene511986                     1    1    1    1    1   5
Ophrys-gene512108                     1    1    1    1    1   5
Ophrys-gene512329                     1    0    1    1    0   3
Ophrys-gene512330                     0    0    1    0    0   1
Ophrys-gene518809                     0    0    0    0    0   0
Ophrys-gene525816                     1    1    1    1    1   5
Ophrys-gene525928                     1    1    0    1    0   3
Ophrys-gene526019                     1    0    1    0    0   2
Ophrys-gene528188                     1    0    0    0    0   1
Ophrys-gene531294                     1    0    0    1    0   2
Ophrys-gene531999                     1    1    0    1    0   3
Ophrys-gene538311                     1    1    1    1    1   5
Ophrys-gene546762                     1    1    1    1    1   5
Ophrys-gene546823                     1    1    1    1    1   5
Ophrys-gene546875                     1    0    0    1    0   2
Ophrys-gene551595                     0    0    1    0    0   1
Ophrys-gene552147                     1    0    0    1    0   2
Ophrys-gene553949                     1    1    1    1    1   5
Ophrys-gene553950                     0    0    0    1    0   1
Ophrys-gene554479                     1    0    1    1    0   3
Ophrys-gene554483                     1    1    1    1    1   5
Ophrys-gene554495                     1    0    0    1    0   2
Ophrys-gene554500                     1    0    0    0    0   1
Ophrys-gene555137                     1    0    0    1    0   2
Ophrys-gene555138                     1    0    1    0    0   2
Ophrys-gene555139                     1    1    1    1    1   5
Ophrys-gene555165                     1    1    1    1    0   4
Ophrys-gene555166                     1    1    1    1    1   5
Ophrys-gene556001                     1    0    1    0    0   2
Ophrys-gene559758                     1    1    1    1    1   5
Ophrys-gene560147                     1    0    0    1    0   2
Ophrys-gene561804                     1    0    1    0    0   2
Ophrys-gene561805                     1    0    1    1    0   3
Ophrys-gene561829                     1    0    0    1    0   2
Ophrys-gene561830                     0    1    1    1    0   3
Ophrys-gene563072                     1    0    0    1    0   2
Ophrys-gene564558                     0    1    0    1    0   2
Ophrys-gene564559                     0    0    0    1    0   1
Ophrys-gene565168                     1    1    1    1    1   5
Ophrys-gene565167                     1    1    1    1    1   5
Ophrys-gene566330                     1    0    0    1    0   2
Ophrys-gene566441                     1    1    1    1    1   5
Ophrys-gene568671                     1    1    1    1    1   5
Ophrys-gene571420                     1    0    1    1    0   3
Ophrys-gene575719                     0    0    0    1    0   1
Ophrys-gene575718                     1    1    1    1    1   5
Ophrys-gene581699                     1    1    1    1    1   5
Ophrys-gene581753                     1    1    1    1    1   5
Ophrys-gene586927                     0    0    0    1    0   1
Ophrys-gene588622                     1    0    0    1    0   2
Ophrys-gene588675                     1    1    0    1    0   3
Ophrys-gene596240                     1    1    1    1    1   5
Ophrys-gene597615                     1    0    0    1    0   2
Ophrys-gene597800                     1    1    1    1    1   5
Ophrys-gene597925                     1    0    0    0    0   1
Ophrys-gene603309                     1    0    0    1    0   2
Ophrys-gene605829                     1    1    1    1    1   5
Ophrys-gene607165                     1    1    1    1    1   5
Ophrys-gene609103                     1    0    0    1    1   3
Ophrys-gene609784                     1    1    1    1    1   5
Ophrys-gene609914                     1    1    1    1    1   5
Ophrys-gene610099                     1    0    0    1    0   2
Ophrys-gene610274                     0    0    0    1    0   1
Ophrys-gene615031                     1    1    1    1    1   5
Ophrys-gene615438                     1    0    0    0    0   1
Ophrys-gene616217                     1    0    0    1    0   2
Ophrys-gene622675                     1    1    1    1    1   5
Ophrys-gene628392                     1    1    1    1    1   5
Ophrys-gene628440                     1    1    1    1    1   5
Ophrys-gene629893                     1    0    1    1    0   3
Ophrys-gene637459                     1    1    1    1    1   5
Ophrys-gene639494                     0    1    0    1    0   2
Ophrys-gene640073                     1    0    1    0    0   2
Ophrys-gene640540                     1    1    1    1    1   5
Ophrys-gene644135                     0    0    0    0    0   0
Ophrys-gene645253                     0    0    1    0    0   1
Ophrys-gene648782                     1    1    1    1    1   5
Ophrys-gene649357                     0    1    0    1    0   2
Ophrys-gene649394                     1    1    0    1    0   3
Ophrys-gene649691                     1    1    1    1    1   5
Ophrys-gene650956                     0    0    0    0    0   0
Ophrys-gene650957                     1    1    1    1    1   5
Ophrys-gene654878                     1    1    1    1    1   5
Ophrys-gene660427                     1    1    0    1    0   3
Ophrys-gene662242                     1    0    1    0    0   2
Ophrys-gene671118                     1    1    1    1    1   5
Ophrys-gene671153                     1    1    1    1    1   5
Ophrys-gene676624                     1    0    0    1    0   2
Ophrys-gene676642                     1    1    1    1    1   5
Ophrys-gene681262                     1    0    0    0    0   1
Ophrys-gene682237                     1    0    1    1    0   3
Ophrys-gene683058                     1    0    0    1    0   2
Ophrys-gene685343                     1    0    0    1    0   2
Ophrys-gene689773                     1    1    1    1    1   5
Ophrys-gene690315                     1    0    0    1    0   2
Ophrys-gene690347                     1    1    1    1    1   5
Ophrys-gene691914                     1    1    1    1    1   5
Ophrys-gene692908                     0    0    0    1    0   1
Ophrys-gene696143                     1    1    1    1    1   5
Ophrys-gene698635                     0    0    1    1    0   2
Ophrys-gene698640                     1    0    1    1    0   3
Ophrys-gene699518                     1    1    1    1    1   5
Ophrys-gene699519                     1    1    1    1    1   5
Ophrys-gene700919                     1    0    0    1    0   2
Ophrys-gene702668                     1    0    1    1    0   3
Ophrys-gene707991                     1    1    1    1    1   5
Ophrys-gene708431                     1    0    0    0    0   1
Ophrys-gene708645                     1    1    1    1    1   5
Ophrys-gene709213                     1    0    1    0    0   2
Ophrys-gene709682                     1    1    1    1    0   4
Ophrys-gene709683                     1    1    1    1    1   5
Ophrys-gene709693                     1    1    1    1    1   5
Ophrys-gene711535                     1    0    1    1    0   3
Ophrys-gene712147                     1    1    1    1    1   5
Ophrys-gene714329                     1    1    1    1    1   5
Ophrys-gene714330                     1    1    1    1    1   5
Ophrys-gene715261                     1    0    0    1    0   2
Ophrys-gene715389                     0    0    0    1    0   1
Ophrys-gene718530                     1    0    0    1    0   2
Ophrys-gene718512                     0    0    0    0    0   0
Ophrys-gene720180                     1    1    1    1    1   5
Ophrys-gene720207                     1    0    0    1    0   2
Ophrys-gene720751                     1    1    1    1    1   5
Ophrys-gene722361                     0    1    0    1    0   2
Ophrys-gene722496                     0    0    0    1    0   1
Ophrys-gene731617                     1    0    1    1    0   3
Ophrys-gene739306                     1    1    1    1    1   5
Ophrys-gene742054                     0    0    0    1    0   1
Ophrys-gene753427                     1    0    0    1    0   2
Ophrys-gene753624                     1    0    0    1    0   2
Ophrys-gene757443                     0    0    1    1    0   2
Ophrys-gene757503                     1    0    1    1    0   3
Ophrys-gene757563                     1    1    1    1    1   5
Ophrys-gene757622                     1    0    1    1    0   3
Ophrys-gene757738                     0    0    0    0    0   0
Ophrys-gene758222                     1    0    1    1    0   3
Ophrys-gene758419                     1    0    0    1    0   2
Ophrys-gene759890                     0    0    1    0    0   1
Ophrys-gene759922                     1    1    1    1    1   5
Ophrys-gene759923                     1    1    1    1    1   5
Ophrys-gene762873                     1    1    1    0    0   3
Ophrys-gene762994                     1    1    1    1    1   5
Ophrys-gene762995                     1    0    0    1    0   2
Ophrys-gene765627                     0    0    0    0    0   0
Ophrys-gene190720                     0    1    1    0    0   2
Ophrys-gene200932                     0    1    0    1    0   2
Ophrys-gene207605                     1    1    1    1    1   5
Ophrys-gene252079                     1    0    0    0    0   1
Ophrys-gene511151                     1    1    1    1    1   5
Ophrys-gene654986                     1    1    1    1    1   5
Ophrys-gene658435                     1    1    1    1    1   5
Ophrys-gene710814                     1    0    0    1    0   2
Ophrys-gene239521                     0    0    0    1    0   1
Ophrys-gene595640                     1    0    0    1    0   2
Ophrys-gene639419                     0    0    0    1    0   1
Ophrys-gene764378                     0    0    0    1    0   1
Ophrys-gene62105                      1    1    1    1    1   5
Ophrys-gene210738                     1    1    1    1    1   5
Ophrys-gene8006                       1    0    0    1    0   2
Ophrys-gene21100                      1    0    0    1    0   2
Ophrys-gene402518                     0    0    1    1    0   2
Ophrys-gene444622                     0    0    0    0    0   0
Ophrys-gene445331                     1    0    0    1    0   2
Ophrys-gene455399                     1    1    1    1    1   5
Ophrys-gene521878                     1    1    0    0    0   2
Ophrys-gene183701                     0    1    0    1    0   2
Ophrys-gene297029                     1    1    1    1    1   5
Ophrys-gene386138                     1    1    1    0    0   3
Ophrys-gene386173                     1    0    0    1    0   2
Ophrys-gene700914                     1    1    1    1    1   5
Ophrys-gene81281                      1    1    1    1    0   4
Ophrys-gene166775                     1    1    1    1    1   5
Ophrys-gene265053                     1    0    0    1    0   2
Ophrys-gene746823                     1    1    1    1    1   5
Ophrys-gene96453                      1    1    1    1    1   5
Ophrys-gene167701                     1    0    1    1    0   3
Ophrys-gene546141                     1    0    1    1    0   3
Ophrys-gene171650                     1    1    1    1    1   5
Ophrys-gene425665                     0    0    0    1    0   1
Ophrys-gene560305                     1    1    1    1    1   5
Ophrys-gene729078                     1    1    1    1    1   5
Ophrys-gene7753                       1    0    1    1    0   3
Ophrys-gene102641                     1    0    0    1    0   2
Ophrys-gene577065                     1    0    0    0    0   1
Ophrys-gene4709                       1    1    1    1    1   5
Ophrys-gene10130                      1    1    1    1    1   5
Ophrys-gene10476                      1    1    1    1    1   5
Ophrys-gene12241                      1    1    1    1    1   5
Ophrys-gene20066                      1    1    1    1    1   5
Ophrys-gene20192                      1    1    1    1    1   5
Ophrys-gene20218                      1    0    1    1    0   3
Ophrys-gene20403                      1    1    1    1    1   5
Ophrys-gene21677                      1    0    0    0    0   1
Ophrys-gene21743                      1    1    1    1    1   5
Ophrys-gene22666                      1    1    1    1    1   5
Ophrys-gene23838                      1    1    1    1    1   5
Ophrys-gene48000                      0    0    1    0    0   1
Ophrys-gene50790                      0    0    1    0    0   1
Ophrys-gene51463                      1    1    1    1    1   5
Ophrys-gene52663                      1    0    0    1    0   2
Ophrys-gene60400                      1    1    1    1    1   5
Ophrys-gene66904                      1    1    1    1    1   5
Ophrys-gene69439                      1    1    1    1    1   5
Ophrys-gene71645                      0    1    1    0    0   2
Ophrys-gene76283                      1    1    1    1    1   5
Ophrys-gene76839                      1    1    1    1    1   5
Ophrys-gene76895                      1    0    0    1    0   2
Ophrys-gene76823                      0    0    1    1    1   3
Ophrys-gene79465                      1    0    0    1    0   2
Ophrys-gene84020                      1    1    1    1    1   5
Ophrys-gene88405                      0    0    0    1    0   1
Ophrys-gene91053                      1    0    0    0    0   1
Ophrys-gene96433                      1    1    1    1    1   5
Ophrys-gene100631                     0    0    1    1    0   2
Ophrys-gene112878                     1    0    0    1    0   2
Ophrys-gene114865                     1    0    1    1    1   4
Ophrys-gene118631                     1    1    0    0    0   2
Ophrys-gene123709                     1    0    0    0    0   1
Ophrys-gene126479                     1    0    0    1    0   2
Ophrys-gene134804                     1    1    1    1    1   5
Ophrys-gene137136                     1    1    1    1    0   4
Ophrys-gene141576                     1    1    1    1    1   5
Ophrys-gene141678                     0    0    1    1    0   2
Ophrys-gene144449                     1    0    1    1    0   3
Ophrys-gene144758                     0    0    1    1    0   2
Ophrys-gene162298                     1    1    1    1    1   5
Ophrys-gene174190                     1    0    1    0    0   2
Ophrys-gene180334                     1    1    1    1    1   5
Ophrys-gene183182                     1    0    1    1    0   3
Ophrys-gene187155                     1    0    1    1    0   3
Ophrys-gene190757                     1    1    1    1    1   5
Ophrys-gene190753                     0    0    1    1    0   2
Ophrys-gene206013                     1    0    1    0    0   2
Ophrys-gene214111                     1    1    1    1    1   5
Ophrys-gene214155                     1    0    1    1    0   3
Ophrys-gene219178                     0    0    0    1    0   1
Ophrys-gene220650                     1    1    1    1    1   5
Ophrys-gene222220                     1    1    1    1    1   5
Ophrys-gene222205                     1    0    1    0    0   2
Ophrys-gene225215                     1    0    0    1    0   2
Ophrys-gene225216                     1    1    1    1    1   5
Ophrys-gene231692                     1    1    1    1    1   5
Ophrys-gene258661                     1    0    0    0    0   1
Ophrys-gene258762                     1    0    0    1    1   3
Ophrys-gene263165                     1    1    1    1    1   5
Ophrys-gene263285                     1    1    1    1    1   5
Ophrys-gene265763                     1    1    1    1    1   5
Ophrys-gene271069                     1    0    0    0    0   1
Ophrys-gene271886                     1    0    1    0    0   2
Ophrys-gene281289                     1    1    1    1    1   5
Ophrys-gene285185                     1    0    1    1    0   3
Ophrys-gene286153                     1    0    0    1    0   2
Ophrys-gene287289                     1    0    1    1    0   3
Ophrys-gene287292                     1    1    1    1    1   5
Ophrys-gene288736                     0    1    1    0    0   2
Ophrys-gene288723                     1    0    0    1    0   2
Ophrys-gene288724                     1    0    1    1    0   3
Ophrys-gene289802                     1    1    1    1    1   5
Ophrys-gene291987                     1    1    1    1    1   5
Ophrys-gene295182                     0    0    1    1    0   2
Ophrys-gene297208                     1    0    0    1    0   2
Ophrys-gene298776                     1    1    1    1    1   5
Ophrys-gene304050                     1    0    1    0    0   2
Ophrys-gene340535                     1    0    1    1    0   3
Ophrys-gene342465                     1    1    1    1    1   5
Ophrys-gene342457                     0    0    0    1    0   1
Ophrys-gene344518                     0    0    0    0    0   0
Ophrys-gene346434                     0    0    1    1    0   2
Ophrys-gene348905                     1    1    1    1    1   5
Ophrys-gene349575                     1    0    0    0    0   1
Ophrys-gene351525                     0    0    1    1    0   2
Ophrys-gene352595                     1    0    0    1    0   2
Ophrys-gene377612                     1    1    1    1    1   5
Ophrys-gene382224                     1    0    0    1    0   2
Ophrys-gene382309                     0    0    0    1    0   1
Ophrys-gene385862                     1    0    1    0    1   3
Ophrys-gene385866                     1    1    1    1    1   5
Ophrys-gene385867                     1    1    1    1    1   5
Ophrys-gene386403                     1    0    1    0    0   2
Ophrys-gene388291                     1    1    1    1    1   5
Ophrys-gene390540                     1    0    1    1    0   3
Ophrys-gene395694                     0    1    1    0    0   2
Ophrys-gene396937                     1    1    1    1    1   5
Ophrys-gene400015                     1    0    0    1    1   3
Ophrys-gene403934                     1    0    0    0    0   1
Ophrys-gene404507                     1    0    0    1    0   2
Ophrys-gene410567                     1    0    0    0    0   1
Ophrys-gene435139                     0    1    0    1    0   2
Ophrys-gene444567                     0    0    0    1    1   2
Ophrys-gene444728                     1    1    0    1    0   3
Ophrys-gene444843                     1    0    1    1    0   3
Ophrys-gene445039                     1    0    0    1    0   2
Ophrys-gene445089                     0    0    0    1    0   1
Ophrys-gene445148                     1    1    1    1    1   5
Ophrys-gene445284                     0    0    0    0    0   0
Ophrys-gene446315                     1    1    1    1    1   5
Ophrys-gene446731                     1    0    0    0    0   1
Ophrys-gene447268                     1    0    0    1    0   2
Ophrys-gene448040                     1    0    0    1    0   2
Ophrys-gene451807                     1    1    0    0    0   2
Ophrys-gene452253                     1    0    0    0    0   1
Ophrys-gene462293                     1    1    0    1    0   3
Ophrys-gene467393                     0    0    0    1    0   1
Ophrys-gene480781                     1    0    1    1    0   3
Ophrys-gene484566                     1    1    1    1    1   5
Ophrys-gene484567                     0    0    0    0    0   0
Ophrys-gene493101                     1    0    0    1    0   2
Ophrys-gene506610                     1    1    1    1    1   5
Ophrys-gene507982                     1    1    1    1    1   5
Ophrys-gene509369                     1    1    0    1    0   3
Ophrys-gene510605                     1    0    0    0    0   1
Ophrys-gene511157                     1    0    1    0    0   2
Ophrys-gene515823                     0    0    1    1    0   2
Ophrys-gene516164                     1    0    1    0    0   2
Ophrys-gene525918                     1    1    1    1    1   5
Ophrys-gene526221                     1    1    1    1    1   5
Ophrys-gene536628                     1    0    1    0    0   2
Ophrys-gene543926                     0    0    0    1    0   1
Ophrys-gene545810                     1    1    0    1    0   3
Ophrys-gene546140                     0    0    1    1    0   2
Ophrys-gene551665                     0    0    0    1    0   1
Ophrys-gene554484                     1    0    0    0    0   1
Ophrys-gene566424                     1    1    1    1    1   5
Ophrys-gene579288                     1    1    1    1    1   5
Ophrys-gene583072                     1    0    0    1    0   2
Ophrys-gene586417                     1    1    1    1    1   5
Ophrys-gene586444                     1    0    0    0    0   1
Ophrys-gene586783                     0    0    0    1    0   1
Ophrys-gene586833                     0    0    0    0    0   0
Ophrys-gene586926                     1    1    1    1    1   5
Ophrys-gene586972                     1    1    0    1    0   3
Ophrys-gene597924                     1    0    0    1    0   2
Ophrys-gene601283                     1    1    1    1    1   5
Ophrys-gene608265                     1    1    0    1    0   3
Ophrys-gene613459                     1    0    1    0    0   2
Ophrys-gene613552                     0    0    0    1    0   1
Ophrys-gene613826                     1    0    0    0    0   1
Ophrys-gene613877                     0    0    0    1    0   1
Ophrys-gene615437                     0    0    1    1    0   2
Ophrys-gene616636                     1    1    1    1    1   5
Ophrys-gene626187                     1    0    0    1    0   2
Ophrys-gene628439                     1    0    0    1    0   2
Ophrys-gene639906                     0    0    1    1    0   2
Ophrys-gene642587                     1    0    0    1    0   2
Ophrys-gene642609                     0    0    1    0    0   1
Ophrys-gene650915                     1    0    0    1    0   2
Ophrys-gene661658                     1    1    0    1    0   3
Ophrys-gene661640                     1    0    1    0    0   2
Ophrys-gene662245                     1    1    1    1    1   5
Ophrys-gene668155                     1    1    1    1    1   5
Ophrys-gene668156                     1    1    1    1    1   5
Ophrys-gene683103                     1    1    1    1    1   5
Ophrys-gene691412                     1    0    1    1    0   3
Ophrys-gene696055                     0    0    1    1    0   2
Ophrys-gene700926                     1    1    1    1    1   5
Ophrys-gene709058                     1    1    1    1    1   5
Ophrys-gene709314                     1    0    0    0    0   1
Ophrys-gene710969                     1    1    1    1    1   5
Ophrys-gene712485                     1    1    1    1    1   5
Ophrys-gene712785                     1    0    0    1    1   3
Ophrys-gene731741                     0    0    1    1    0   2
Ophrys-gene731866                     1    0    1    1    0   3
Ophrys-gene739305                     1    0    1    1    0   3
Ophrys-gene755152                     1    1    1    1    1   5
Ophrys-gene755153                     1    1    1    1    1   5
Ophrys-gene758069                     1    1    1    1    0   4
Ophrys-gene758221                     0    1    1    1    0   3
Ophrys-gene758418                     1    0    1    0    0   2
Ophrys-gene769418                     1    1    1    1    1   5
Phalaenopsis-gene100592               0    0    0    1    0   1
Phalaenopsis-gene107060               1    1    1    1    1   5
Phalaenopsis-gene107061               1    1    1    1    1   5
Phalaenopsis-gene108041               1    1    1    1    1   5
Phalaenopsis-gene110708               1    1    1    1    1   5
Phalaenopsis-gene110709               0    0    1    0    0   1
Phalaenopsis-gene110710               1    1    1    1    1   5
Phalaenopsis-gene111682               1    1    1    1    1   5
Phalaenopsis-gene111683               1    1    1    1    1   5
Phalaenopsis-gene111684               1    1    1    1    1   5
Phalaenopsis-gene111685               1    1    1    1    1   5
Phalaenopsis-gene114497               1    0    0    1    0   2
Phalaenopsis-gene115562               1    1    1    1    1   5
Phalaenopsis-gene120226               1    0    1    1    0   3
Phalaenopsis-gene120552               1    1    1    1    0   4
Phalaenopsis-gene120553               1    1    1    1    0   4
Phalaenopsis-gene123359               0    0    1    1    0   2
Phalaenopsis-gene124871               1    0    1    0    0   2
Phalaenopsis-gene125030               0    0    1    1    0   2
Phalaenopsis-gene125343               1    0    0    1    0   2
Phalaenopsis-gene128419               1    0    0    1    0   2
Phalaenopsis-gene129808               1    0    1    1    0   3
Phalaenopsis-gene129809               1    1    1    1    1   5
Phalaenopsis-gene130044               1    0    1    0    0   2
Phalaenopsis-gene130306               1    0    1    1    0   3
Phalaenopsis-gene130390               1    0    1    1    0   3
Phalaenopsis-gene130419               0    0    1    1    0   2
Phalaenopsis-gene133035               1    0    0    1    0   2
Phalaenopsis-gene135373               1    1    1    1    1   5
Phalaenopsis-gene137648               1    1    1    1    1   5
Phalaenopsis-gene137692               1    0    1    1    0   3
Phalaenopsis-gene138452               1    0    1    1    0   3
Phalaenopsis-gene138875               1    1    1    1    1   5
Phalaenopsis-gene138876               0    0    1    1    0   2
Phalaenopsis-gene149970               1    1    0    1    0   3
Phalaenopsis-gene150079               1    0    1    0    0   2
Phalaenopsis-gene150228               1    1    1    0    1   4
Phalaenopsis-gene150313               1    0    0    1    0   2
Phalaenopsis-gene151090               1    0    1    1    0   3
Phalaenopsis-gene151233               0    0    1    1    0   2
Phalaenopsis-gene151286               1    0    1    1    0   3
Phalaenopsis-gene152445               1    1    1    1    1   5
Phalaenopsis-gene152912               1    1    1    1    1   5
Phalaenopsis-gene152953               1    0    0    1    1   3
Phalaenopsis-gene152979               1    1    1    1    1   5
Phalaenopsis-gene156047               1    1    1    1    1   5
Phalaenopsis-gene156057               1    1    1    1    1   5
Phalaenopsis-gene156137               0    0    1    1    0   2
Phalaenopsis-gene156160               1    0    0    1    0   2
Phalaenopsis-gene156977               1    0    1    1    0   3
Phalaenopsis-gene15833                1    1    1    1    1   5
Phalaenopsis-gene15834                1    1    1    1    1   5
Phalaenopsis-gene163312               0    0    0    1    0   1
Phalaenopsis-gene163313               1    0    1    1    0   3
Phalaenopsis-gene163541               1    0    0    1    0   2
Phalaenopsis-gene163563               1    0    0    0    0   1
Phalaenopsis-gene163585               0    0    0    0    0   0
Phalaenopsis-gene163733               1    1    1    1    0   4
Phalaenopsis-gene167424               1    0    1    1    1   4
Phalaenopsis-gene168148               0    0    1    1    1   3
Phalaenopsis-gene169624               1    0    0    1    0   2
Phalaenopsis-gene169649               1    0    0    1    0   2
Phalaenopsis-gene169677               0    0    0    1    0   1
Phalaenopsis-gene169706               1    0    0    1    0   2
Phalaenopsis-gene169729               1    0    0    1    0   2
Phalaenopsis-gene169755               1    0    0    1    0   2
Phalaenopsis-gene169786               1    0    0    1    0   2
Phalaenopsis-gene170756               1    0    1    0    0   2
Phalaenopsis-gene170902               1    0    0    1    0   2
Phalaenopsis-gene171943               1    0    1    1    0   3
Phalaenopsis-gene172039               1    1    0    0    0   2
Phalaenopsis-gene172209               1    0    1    1    0   3
Phalaenopsis-gene173913               1    1    0    1    0   3
Phalaenopsis-gene173914               1    0    1    0    0   2
Phalaenopsis-gene173915               0    0    0    0    0   0
Phalaenopsis-gene174059               1    1    0    0    0   2
Phalaenopsis-gene174060               0    1    1    1    0   3
Phalaenopsis-gene174061               0    0    1    1    0   2
Phalaenopsis-gene174112               1    0    0    1    0   2
Phalaenopsis-gene174113               0    0    1    1    0   2
Phalaenopsis-gene178900               1    1    1    1    0   4
Phalaenopsis-gene178937               1    1    1    1    1   5
Phalaenopsis-gene181766               1    1    1    1    1   5
Phalaenopsis-gene181767               1    1    1    1    1   5
Phalaenopsis-gene181768               1    1    0    1    0   3
Phalaenopsis-gene181769               1    1    1    1    1   5
Phalaenopsis-gene181772               1    1    1    1    1   5
Phalaenopsis-gene18220                1    0    1    1    0   3
Phalaenopsis-gene183072               0    0    1    1    0   2
Phalaenopsis-gene183187               1    0    0    0    0   1
Phalaenopsis-gene183198               1    0    0    1    0   2
Phalaenopsis-gene184327               0    0    0    1    0   1
Phalaenopsis-gene184488               0    0    0    1    0   1
Phalaenopsis-gene184661               1    0    0    1    0   2
Phalaenopsis-gene18706                1    1    1    1    0   4
Phalaenopsis-gene188617               1    0    1    1    0   3
Phalaenopsis-gene188751               1    1    1    1    1   5
Phalaenopsis-gene189013               1    0    1    1    0   3
Phalaenopsis-gene192999               1    0    1    0    0   2
Phalaenopsis-gene194765               1    1    1    1    1   5
Phalaenopsis-gene194822               1    1    1    1    1   5
Phalaenopsis-gene196458               1    0    1    1    0   3
Phalaenopsis-gene197967               0    0    0    1    0   1
Phalaenopsis-gene199474               1    1    1    0    0   3
Phalaenopsis-gene202829               1    0    1    1    0   3
Phalaenopsis-gene203075               0    0    1    0    0   1
Phalaenopsis-gene203840               1    0    1    1    0   3
Phalaenopsis-gene204501               1    0    1    1    0   3
Phalaenopsis-gene209179               1    0    0    1    0   2
Phalaenopsis-gene209217               0    0    0    1    0   1
Phalaenopsis-gene209255               1    0    0    1    0   2
Phalaenopsis-gene209269               1    0    0    1    0   2
Phalaenopsis-gene211555               1    0    0    0    0   1
Phalaenopsis-gene214953               0    1    0    1    0   2
Phalaenopsis-gene217979               1    0    0    0    0   1
Phalaenopsis-gene218937               0    0    1    0    0   1
Phalaenopsis-gene222337               1    1    1    1    1   5
Phalaenopsis-gene222527               1    0    0    1    0   2
Phalaenopsis-gene223199               0    0    1    0    0   1
Phalaenopsis-gene22710                1    1    1    1    1   5
Phalaenopsis-gene22714                1    1    1    1    1   5
Phalaenopsis-gene22715                1    1    1    1    1   5
Phalaenopsis-gene22716                1    1    1    1    1   5
Phalaenopsis-gene22717                1    1    1    1    1   5
Phalaenopsis-gene22719                1    1    1    1    1   5
Phalaenopsis-gene22720                1    1    1    1    1   5
Phalaenopsis-gene22721                1    1    1    1    1   5
Phalaenopsis-gene228738               1    0    1    1    0   3
Phalaenopsis-gene22919                1    1    1    1    1   5
Phalaenopsis-gene229627               1    0    1    0    0   2
Phalaenopsis-gene23026                1    1    0    0    0   2
Phalaenopsis-gene231804               1    0    0    0    0   1
Phalaenopsis-gene234927               1    1    0    1    1   4
Phalaenopsis-gene23920                1    0    1    1    0   3
Phalaenopsis-gene240942               1    1    1    1    1   5
Phalaenopsis-gene241669               1    1    1    1    1   5
Phalaenopsis-gene243616               1    1    1    1    0   4
Phalaenopsis-gene244686               0    0    0    1    0   1
Phalaenopsis-gene244797               1    1    1    1    1   5
Phalaenopsis-gene247182               0    0    1    0    0   1
Phalaenopsis-gene247183               1    0    0    1    0   2
Phalaenopsis-gene247190               1    1    1    1    1   5
Phalaenopsis-gene250178               1    0    0    0    0   1
Phalaenopsis-gene254646               1    0    0    0    0   1
Phalaenopsis-gene254647               1    0    1    0    0   2
Phalaenopsis-gene254657               1    1    0    1    0   3
Phalaenopsis-gene254672               0    0    0    0    0   0
Phalaenopsis-gene254673               1    0    1    1    0   3
Phalaenopsis-gene254683               1    1    1    1    1   5
Phalaenopsis-gene254684               1    0    1    1    0   3
Phalaenopsis-gene254699               1    0    0    1    0   2
Phalaenopsis-gene254700               1    1    0    1    0   3
Phalaenopsis-gene254710               1    0    0    1    0   2
Phalaenopsis-gene254711               1    0    0    1    0   2
Phalaenopsis-gene25479                1    1    1    1    1   5
Phalaenopsis-gene254813               1    0    0    1    0   2
Phalaenopsis-gene255167               1    1    1    1    1   5
Phalaenopsis-gene255255               1    1    1    1    1   5
Phalaenopsis-gene255726               1    0    1    1    1   4
Phalaenopsis-gene255727               1    0    1    0    0   2
Phalaenopsis-gene25804                0    0    0    1    0   1
Phalaenopsis-gene258290               1    0    1    1    0   3
Phalaenopsis-gene258425               1    0    0    1    0   2
Phalaenopsis-gene259775               0    0    1    1    0   2
Phalaenopsis-gene259944               1    0    1    1    0   3
Phalaenopsis-gene267406               1    1    1    1    1   5
Phalaenopsis-gene27332                1    0    1    0    0   2
Phalaenopsis-gene27341                1    1    1    1    1   5
Phalaenopsis-gene273427               1    0    0    1    0   2
Phalaenopsis-gene273428               1    1    1    1    1   5
Phalaenopsis-gene27662                0    1    1    1    0   3
Phalaenopsis-gene276802               1    1    1    1    0   4
Phalaenopsis-gene276852               1    1    1    1    1   5
Phalaenopsis-gene278407               1    0    0    1    0   2
Phalaenopsis-gene280703               1    1    1    1    1   5
Phalaenopsis-gene280704               1    1    1    1    1   5
Phalaenopsis-gene281356               1    1    0    1    0   3
Phalaenopsis-gene282157               1    0    1    0    0   2
Phalaenopsis-gene282221               0    0    1    1    0   2
Phalaenopsis-gene282236               1    1    1    0    0   3
Phalaenopsis-gene28417                1    1    1    1    1   5
Phalaenopsis-gene285471               0    1    1    1    0   3
Phalaenopsis-gene285520               1    0    0    0    0   1
Phalaenopsis-gene285572               1    0    0    0    0   1
Phalaenopsis-gene285822               1    0    1    0    0   2
Phalaenopsis-gene285856               1    1    1    1    1   5
Phalaenopsis-gene286543               0    0    1    1    0   2
Phalaenopsis-gene286601               1    0    1    1    0   3
Phalaenopsis-gene28680                1    1    1    1    1   5
Phalaenopsis-gene287220               1    0    0    1    0   2
Phalaenopsis-gene287285               1    0    1    0    0   2
Phalaenopsis-gene289076               1    1    1    1    1   5
Phalaenopsis-gene289316               1    1    1    1    1   5
Phalaenopsis-gene289317               1    0    1    0    0   2
Phalaenopsis-gene289734               1    0    1    1    0   3
Phalaenopsis-gene289735               0    0    1    1    0   2
Phalaenopsis-gene291283               1    1    1    1    1   5
Phalaenopsis-gene291286               1    1    1    1    1   5
Phalaenopsis-gene291313               1    1    1    1    1   5
Phalaenopsis-gene292413               1    1    1    1    1   5
Phalaenopsis-gene292414               1    1    1    1    1   5
Phalaenopsis-gene294987               1    0    1    1    0   3
Phalaenopsis-gene296606               1    1    1    1    1   5
Phalaenopsis-gene296607               0    0    0    0    0   0
Phalaenopsis-gene297285               1    1    1    1    1   5
Phalaenopsis-gene299208               1    0    0    1    0   2
Phalaenopsis-gene300846               0    0    0    0    0   0
Phalaenopsis-gene300847               1    0    0    0    0   1
Phalaenopsis-gene300848               1    0    0    1    0   2
Phalaenopsis-gene300849               1    1    0    1    0   3
Phalaenopsis-gene300872               1    0    1    1    0   3
Phalaenopsis-gene300873               0    0    1    1    0   2
Phalaenopsis-gene300874               0    0    0    1    0   1
Phalaenopsis-gene306641               1    0    0    1    0   2
Phalaenopsis-gene306642               1    0    1    1    0   3
Phalaenopsis-gene306971               1    1    1    1    1   5
Phalaenopsis-gene307607               1    1    1    1    0   4
Phalaenopsis-gene307622               1    0    1    1    1   4
Phalaenopsis-gene307746               0    0    0    1    0   1
Phalaenopsis-gene307870               1    0    0    1    0   2
Phalaenopsis-gene310814               0    0    0    1    0   1
Phalaenopsis-gene311669               1    1    1    1    1   5
Phalaenopsis-gene312040               1    1    1    1    1   5
Phalaenopsis-gene312592               0    0    0    1    0   1
Phalaenopsis-gene314278               0    1    0    1    0   2
Phalaenopsis-gene31861                1    1    1    1    1   5
Phalaenopsis-gene318822               1    0    1    1    0   3
Phalaenopsis-gene319735               1    0    0    1    0   2
Phalaenopsis-gene320071               1    0    1    1    0   3
Phalaenopsis-gene320076               1    0    1    0    0   2
Phalaenopsis-gene320154               0    0    1    0    0   1
Phalaenopsis-gene320159               1    0    1    1    0   3
Phalaenopsis-gene321495               1    1    1    1    1   5
Phalaenopsis-gene324523               1    0    0    1    0   2
Phalaenopsis-gene324546               0    0    0    0    0   0
Phalaenopsis-gene324559               0    0    0    0    0   0
Phalaenopsis-gene324579               0    0    1    0    0   1
Phalaenopsis-gene324584               1    1    1    1    0   4
Phalaenopsis-gene326405               1    1    1    1    1   5
Phalaenopsis-gene326451               1    0    1    1    0   3
Phalaenopsis-gene326726               1    0    0    1    0   2
Phalaenopsis-gene327244               1    1    1    1    0   4
Phalaenopsis-gene328790               0    0    0    1    0   1
Phalaenopsis-gene331604               1    1    1    1    1   5
Phalaenopsis-gene331605               0    0    1    1    0   2
Phalaenopsis-gene332070               1    1    1    1    1   5
Phalaenopsis-gene333279               1    1    1    1    1   5
Phalaenopsis-gene333280               1    0    0    0    0   1
Phalaenopsis-gene333376               1    0    0    1    0   2
Phalaenopsis-gene334346               1    1    1    1    1   5
Phalaenopsis-gene334823               0    0    0    1    0   1
Phalaenopsis-gene334941               1    0    0    1    0   2
Phalaenopsis-gene335060               1    1    0    1    0   3
Phalaenopsis-gene335173               1    1    1    1    1   5
Phalaenopsis-gene337915               1    0    1    0    0   2
Phalaenopsis-gene337922               1    1    1    1    1   5
Phalaenopsis-gene337923               1    0    0    0    0   1
Phalaenopsis-gene339297               1    1    1    1    1   5
Phalaenopsis-gene340066               0    0    0    1    0   1
Phalaenopsis-gene34295                1    1    1    1    1   5
Phalaenopsis-gene34296                1    1    1    1    1   5
Phalaenopsis-gene34299                0    0    0    1    0   1
Phalaenopsis-gene345039               1    0    1    1    0   3
Phalaenopsis-gene347339               0    1    1    1    0   3
Phalaenopsis-gene348559               1    0    0    0    0   1
Phalaenopsis-gene348776               1    0    0    1    0   2
Phalaenopsis-gene348802               1    1    1    1    1   5
Phalaenopsis-gene349020               1    1    1    1    1   5
Phalaenopsis-gene349078               1    1    1    1    1   5
Phalaenopsis-gene349082               1    0    1    0    0   2
Phalaenopsis-gene349230               1    1    1    1    1   5
Phalaenopsis-gene349316               0    0    0    1    0   1
Phalaenopsis-gene349317               1    1    1    1    1   5
Phalaenopsis-gene349381               1    1    1    1    1   5
Phalaenopsis-gene350781               1    0    1    1    0   3
Phalaenopsis-gene350814               1    1    1    1    1   5
Phalaenopsis-gene351256               0    0    0    1    0   1
Phalaenopsis-gene351329               1    0    0    1    0   2
Phalaenopsis-gene351404               1    0    0    1    0   2
Phalaenopsis-gene351410               1    0    1    0    0   2
Phalaenopsis-gene351463               1    1    0    1    0   3
Phalaenopsis-gene351863               1    0    1    1    0   3
Phalaenopsis-gene351876               1    0    1    1    0   3
Phalaenopsis-gene353043               1    1    1    1    1   5
Phalaenopsis-gene355363               1    0    1    0    0   2
Phalaenopsis-gene356209               1    1    1    1    1   5
Phalaenopsis-gene356210               0    0    1    0    0   1
Phalaenopsis-gene357911               0    0    1    1    0   2
Phalaenopsis-gene363554               0    1    0    0    0   1
Phalaenopsis-gene363555               1    0    0    1    0   2
Phalaenopsis-gene36571                1    0    0    1    0   2
Phalaenopsis-gene36641                1    0    1    1    0   3
Phalaenopsis-gene36645                1    0    0    0    0   1
Phalaenopsis-gene36714                1    0    0    1    0   2
Phalaenopsis-gene36718                1    0    0    1    0   2
Phalaenopsis-gene36787                0    0    1    1    1   3
Phalaenopsis-gene36862                1    0    0    0    0   1
Phalaenopsis-gene36866                1    0    0    0    0   1
Phalaenopsis-gene369414               1    1    1    1    1   5
Phalaenopsis-gene369794               1    1    1    1    1   5
Phalaenopsis-gene370616               1    0    1    1    0   3
Phalaenopsis-gene370890               1    0    1    1    0   3
Phalaenopsis-gene370891               0    0    1    1    0   2
Phalaenopsis-gene370962               1    1    1    1    1   5
Phalaenopsis-gene370963               1    0    1    1    0   3
Phalaenopsis-gene371034               1    0    1    1    1   4
Phalaenopsis-gene371035               0    0    1    1    0   2
Phalaenopsis-gene372169               1    0    1    1    0   3
Phalaenopsis-gene372199               0    0    0    1    0   1
Phalaenopsis-gene37271                1    1    1    1    1   5
Phalaenopsis-gene373559               1    0    1    0    0   2
Phalaenopsis-gene373560               1    1    1    1    1   5
Phalaenopsis-gene374980               1    1    1    1    1   5
Phalaenopsis-gene375443               1    0    0    1    0   2
Phalaenopsis-gene377196               1    1    1    1    1   5
Phalaenopsis-gene377865               0    0    0    1    0   1
Phalaenopsis-gene377899               1    0    1    1    0   3
Phalaenopsis-gene377900               0    0    0    1    0   1
Phalaenopsis-gene378841               1    1    1    0    0   3
Phalaenopsis-gene378880               1    1    1    1    1   5
Phalaenopsis-gene384266               1    1    1    1    1   5
Phalaenopsis-gene384539               1    1    1    1    1   5
Phalaenopsis-gene384541               1    1    1    1    1   5
Phalaenopsis-gene385246               1    1    1    1    1   5
Phalaenopsis-gene387292               1    1    0    0    0   2
Phalaenopsis-gene387335               1    1    1    1    1   5
Phalaenopsis-gene387340               1    0    0    0    0   1
Phalaenopsis-gene38759                1    1    1    1    1   5
Phalaenopsis-gene388027               0    0    1    1    0   2
Phalaenopsis-gene391034               0    0    1    1    0   2
Phalaenopsis-gene391243               1    1    1    1    1   5
Phalaenopsis-gene391244               1    1    1    1    0   4
Phalaenopsis-gene393760               1    0    1    1    0   3
Phalaenopsis-gene393854               1    0    1    1    0   3
Phalaenopsis-gene398824               1    0    1    1    0   3
Phalaenopsis-gene403482               1    1    1    1    1   5
Phalaenopsis-gene405064               0    0    1    1    0   2
Phalaenopsis-gene405068               1    0    1    0    0   2
Phalaenopsis-gene407679               1    1    0    1    0   3
Phalaenopsis-gene410767               1    0    1    0    0   2
Phalaenopsis-gene412714               1    0    1    1    0   3
Phalaenopsis-gene412818               1    1    0    1    0   3
Phalaenopsis-gene413168               0    0    0    0    0   0
Phalaenopsis-gene419166               0    0    1    1    0   2
Phalaenopsis-gene421978               1    1    1    1    1   5
Phalaenopsis-gene424276               1    1    1    1    1   5
Phalaenopsis-gene42541                1    0    0    1    0   2
Phalaenopsis-gene425518               1    0    0    1    1   3
Phalaenopsis-gene425654               1    0    0    0    0   1
Phalaenopsis-gene426569               1    0    1    1    0   3
Phalaenopsis-gene426588               1    1    1    1    1   5
Phalaenopsis-gene429896               1    1    1    1    1   5
Phalaenopsis-gene429898               1    0    1    1    0   3
Phalaenopsis-gene431951               1    0    1    1    0   3
Phalaenopsis-gene433409               1    1    1    1    1   5
Phalaenopsis-gene434132               1    1    1    1    1   5
Phalaenopsis-gene434177               1    0    1    1    0   3
Phalaenopsis-gene434178               1    0    1    1    0   3
Phalaenopsis-gene434197               0    0    1    1    0   2
Phalaenopsis-gene434218               1    1    1    1    1   5
Phalaenopsis-gene43638                1    1    1    1    1   5
Phalaenopsis-gene43639                1    1    1    1    1   5
Phalaenopsis-gene43664                1    1    1    1    1   5
Phalaenopsis-gene437336               0    0    0    1    0   1
Phalaenopsis-gene437573               1    0    0    1    0   2
Phalaenopsis-gene438620               1    1    1    1    1   5
Phalaenopsis-gene44208                1    0    1    0    0   2
Phalaenopsis-gene44210                1    1    1    1    1   5
Phalaenopsis-gene44219                1    0    1    1    1   4
Phalaenopsis-gene44220                1    1    1    1    1   5
Phalaenopsis-gene442560               1    1    1    1    1   5
Phalaenopsis-gene443794               1    0    0    0    0   1
Phalaenopsis-gene443868               1    0    1    0    0   2
Phalaenopsis-gene444155               0    0    1    1    0   2
Phalaenopsis-gene444176               0    1    0    1    1   3
Phalaenopsis-gene444181               1    1    0    1    0   3
Phalaenopsis-gene444694               1    1    1    1    1   5
Phalaenopsis-gene445856               1    0    0    1    0   2
Phalaenopsis-gene447137               1    1    1    1    1   5
Phalaenopsis-gene447271               1    0    1    0    0   2
Phalaenopsis-gene448490               1    1    1    1    1   5
Phalaenopsis-gene448491               0    0    1    1    0   2
Phalaenopsis-gene44940                1    1    1    1    1   5
Phalaenopsis-gene44941                1    0    1    0    0   2
Phalaenopsis-gene450750               1    1    1    1    1   5
Phalaenopsis-gene451678               1    1    1    1    1   5
Phalaenopsis-gene451679               1    0    1    1    0   3
Phalaenopsis-gene451706               1    0    1    1    0   3
Phalaenopsis-gene451707               1    0    0    1    0   2
Phalaenopsis-gene452677               1    0    1    1    0   3
Phalaenopsis-gene454128               1    0    0    0    0   1
Phalaenopsis-gene455607               0    0    0    0    0   0
Phalaenopsis-gene455635               1    1    1    1    1   5
Phalaenopsis-gene456250               1    1    1    1    1   5
Phalaenopsis-gene456303               1    1    1    1    1   5
Phalaenopsis-gene459232               1    0    0    1    0   2
Phalaenopsis-gene460472               1    0    0    0    0   1
Phalaenopsis-gene460473               1    0    0    0    0   1
Phalaenopsis-gene460484               1    1    1    1    1   5
Phalaenopsis-gene460934               1    1    1    1    1   5
Phalaenopsis-gene460958               1    1    1    1    1   5
Phalaenopsis-gene461826               1    1    1    1    1   5
Phalaenopsis-gene461895               1    1    1    1    1   5
Phalaenopsis-gene461931               1    1    1    1    1   5
Phalaenopsis-gene461966               1    0    1    0    0   2
Phalaenopsis-gene463580               1    1    1    1    1   5
Phalaenopsis-gene465956               1    1    1    1    0   4
Phalaenopsis-gene467781               1    0    1    1    0   3
Phalaenopsis-gene46796                0    1    1    1    1   4
Phalaenopsis-gene469209               1    1    1    1    1   5
Phalaenopsis-gene469218               0    0    1    1    0   2
Phalaenopsis-gene469657               1    1    0    1    0   3
Phalaenopsis-gene469658               1    0    0    1    0   2
Phalaenopsis-gene470450               1    0    1    0    0   2
Phalaenopsis-gene471898               1    0    1    1    0   3
Phalaenopsis-gene471940               1    0    1    0    0   2
Phalaenopsis-gene471982               1    1    1    1    0   4
Phalaenopsis-gene4726                 0    0    1    1    0   2
Phalaenopsis-gene475223               1    0    1    1    0   3
Phalaenopsis-gene476529               0    0    1    1    0   2
Phalaenopsis-gene476530               1    1    1    1    1   5
Phalaenopsis-gene477536               1    0    0    1    0   2
Phalaenopsis-gene477892               0    1    1    0    1   3
Phalaenopsis-gene477965               1    1    1    1    1   5
Phalaenopsis-gene479687               1    1    1    1    1   5
Phalaenopsis-gene480641               1    1    1    1    1   5
Phalaenopsis-gene482161               1    0    1    0    0   2
Phalaenopsis-gene48487                1    0    0    1    0   2
Phalaenopsis-gene485457               0    0    1    0    0   1
Phalaenopsis-gene485621               1    0    0    1    0   2
Phalaenopsis-gene485872               1    0    1    0    0   2
Phalaenopsis-gene485873               1    0    0    1    0   2
Phalaenopsis-gene486197               1    0    1    0    0   2
Phalaenopsis-gene486198               1    0    0    1    0   2
Phalaenopsis-gene486282               0    1    0    1    0   2
Phalaenopsis-gene486771               1    0    0    1    0   2
Phalaenopsis-gene486809               0    0    0    1    0   1
Phalaenopsis-gene486848               1    1    1    1    1   5
Phalaenopsis-gene487527               1    1    1    1    1   5
Phalaenopsis-gene487528               0    0    0    1    0   1
Phalaenopsis-gene487548               1    1    1    1    1   5
Phalaenopsis-gene490466               0    0    0    1    0   1
Phalaenopsis-gene49052                1    0    1    1    0   3
Phalaenopsis-gene490542               1    0    0    1    0   2
Phalaenopsis-gene490543               1    0    0    1    0   2
Phalaenopsis-gene490790               0    0    1    0    0   1
Phalaenopsis-gene499355               1    1    1    1    1   5
Phalaenopsis-gene502899               1    0    1    1    0   3
Phalaenopsis-gene504401               1    0    1    0    0   2
Phalaenopsis-gene504423               0    0    1    1    0   2
Phalaenopsis-gene504543               0    0    0    1    0   1
Phalaenopsis-gene504567               1    0    0    1    0   2
Phalaenopsis-gene50492                1    1    1    1    1   5
Phalaenopsis-gene505090               1    1    1    1    1   5
Phalaenopsis-gene505462               1    1    1    1    1   5
Phalaenopsis-gene506686               0    0    1    0    0   1
Phalaenopsis-gene508006               1    1    1    1    0   4
Phalaenopsis-gene508017               0    0    0    0    0   0
Phalaenopsis-gene508022               1    1    1    0    0   3
Phalaenopsis-gene510586               1    1    1    1    1   5
Phalaenopsis-gene511876               1    1    1    1    1   5
Phalaenopsis-gene51336                1    1    1    1    1   5
Phalaenopsis-gene51337                1    1    1    1    1   5
Phalaenopsis-gene51353                1    0    1    0    0   2
Phalaenopsis-gene51354                1    0    0    1    0   2
Phalaenopsis-gene513643               1    1    1    1    1   5
Phalaenopsis-gene513734               1    0    1    1    0   3
Phalaenopsis-gene513779               0    0    1    0    0   1
Phalaenopsis-gene513825               0    0    0    1    0   1
Phalaenopsis-gene513830               0    0    1    0    0   1
Phalaenopsis-gene514728               1    0    1    0    0   2
Phalaenopsis-gene515738               0    0    1    1    0   2
Phalaenopsis-gene516335               0    0    0    1    0   1
Phalaenopsis-gene516358               1    0    0    1    0   2
Phalaenopsis-gene517992               1    0    0    1    0   2
Phalaenopsis-gene519173               1    0    0    1    0   2
Phalaenopsis-gene520079               1    0    1    1    0   3
Phalaenopsis-gene520235               0    1    0    0    0   1
Phalaenopsis-gene520711               1    0    1    1    0   3
Phalaenopsis-gene522410               0    0    1    0    0   1
Phalaenopsis-gene523443               1    0    1    1    0   3
Phalaenopsis-gene523494               1    0    0    1    0   2
Phalaenopsis-gene528055               0    0    1    0    0   1
Phalaenopsis-gene53270                1    0    0    1    0   2
Phalaenopsis-gene53298                1    0    0    1    0   2
Phalaenopsis-gene534060               1    1    1    1    1   5
Phalaenopsis-gene536710               1    1    0    1    0   3
Phalaenopsis-gene538059               1    0    1    1    0   3
Phalaenopsis-gene538063               1    1    1    1    1   5
Phalaenopsis-gene539562               1    1    1    1    1   5
Phalaenopsis-gene54074                1    0    0    0    0   1
Phalaenopsis-gene54167                1    0    0    1    0   2
Phalaenopsis-gene54197                0    0    1    1    0   2
Phalaenopsis-gene543776               1    0    1    0    0   2
Phalaenopsis-gene545604               1    1    0    1    0   3
Phalaenopsis-gene546578               1    0    1    0    0   2
Phalaenopsis-gene548136               1    0    0    0    0   1
Phalaenopsis-gene548166               0    0    1    1    0   2
Phalaenopsis-gene548228               1    1    1    1    1   5
Phalaenopsis-gene548278               1    1    1    1    0   4
Phalaenopsis-gene549120               1    0    1    1    0   3
Phalaenopsis-gene549240               1    0    1    0    0   2
Phalaenopsis-gene549409               1    0    0    1    0   2
Phalaenopsis-gene549410               1    0    1    1    0   3
Phalaenopsis-gene551032               0    1    0    1    0   2
Phalaenopsis-gene551502               1    1    1    1    1   5
Phalaenopsis-gene551720               1    0    1    1    0   3
Phalaenopsis-gene556712               0    0    1    1    0   2
Phalaenopsis-gene556715               1    0    1    1    0   3
Phalaenopsis-gene559407               1    1    1    1    1   5
Phalaenopsis-gene560460               1    1    1    1    1   5
Phalaenopsis-gene561558               0    1    1    1    0   3
Phalaenopsis-gene561938               1    1    1    1    1   5
Phalaenopsis-gene562521               1    0    0    0    1   2
Phalaenopsis-gene564701               1    0    0    1    0   2
Phalaenopsis-gene564718               1    1    1    1    1   5
Phalaenopsis-gene564719               0    0    1    1    0   2
Phalaenopsis-gene567694               1    0    0    1    0   2
Phalaenopsis-gene568300               1    1    1    1    1   5
Phalaenopsis-gene570201               1    0    1    0    0   2
Phalaenopsis-gene571720               1    1    1    1    1   5
Phalaenopsis-gene572967               0    1    1    0    0   2
Phalaenopsis-gene573008               1    1    1    1    1   5
Phalaenopsis-gene573051               1    0    0    1    0   2
Phalaenopsis-gene57412                1    0    1    0    0   2
Phalaenopsis-gene574465               1    1    1    1    1   5
Phalaenopsis-gene574470               1    0    0    0    0   1
Phalaenopsis-gene574471               1    0    1    0    0   2
Phalaenopsis-gene576164               1    0    1    1    0   3
Phalaenopsis-gene578763               1    1    1    1    1   5
Phalaenopsis-gene578796               1    0    0    1    0   2
Phalaenopsis-gene579131               1    1    1    1    1   5
Phalaenopsis-gene579138               1    0    1    1    0   3
Phalaenopsis-gene57917                1    0    0    1    0   2
Phalaenopsis-gene579484               1    0    1    1    0   3
Phalaenopsis-gene580890               1    0    1    1    0   3
Phalaenopsis-gene588146               1    0    1    1    0   3
Phalaenopsis-gene588147               1    0    1    0    0   2
Phalaenopsis-gene588960               1    1    1    1    1   5
Phalaenopsis-gene588981               1    0    0    1    0   2
Phalaenopsis-gene588986               1    0    1    0    0   2
Phalaenopsis-gene58993                1    0    1    0    0   2
Phalaenopsis-gene58994                1    1    1    1    1   5
Phalaenopsis-gene590804               1    1    1    1    1   5
Phalaenopsis-gene590811               0    0    1    1    0   2
Phalaenopsis-gene591701               1    0    1    0    0   2
Phalaenopsis-gene592751               1    1    0    1    0   3
Phalaenopsis-gene592797               1    0    0    1    0   2
Phalaenopsis-gene593291               1    1    1    1    1   5
Phalaenopsis-gene59336                1    0    1    1    0   3
Phalaenopsis-gene594962               1    1    0    1    0   3
Phalaenopsis-gene595161               0    0    0    0    0   0
Phalaenopsis-gene595224               0    0    0    0    0   0
Phalaenopsis-gene595742               1    0    1    1    0   3
Phalaenopsis-gene596064               1    0    0    1    0   2
Phalaenopsis-gene60905                1    0    0    1    0   2
Phalaenopsis-gene609344               1    1    1    1    1   5
Phalaenopsis-gene609345               1    0    1    1    0   3
Phalaenopsis-gene610695               1    0    1    1    1   4
Phalaenopsis-gene610696               0    0    1    1    0   2
Phalaenopsis-gene611214               1    1    1    1    1   5
Phalaenopsis-gene611307               1    0    0    0    0   1
Phalaenopsis-gene611493               1    1    1    1    1   5
Phalaenopsis-gene611494               1    1    1    1    1   5
Phalaenopsis-gene61305                1    0    0    1    0   2
Phalaenopsis-gene614424               1    0    0    1    0   2
Phalaenopsis-gene615902               1    0    0    0    0   1
Phalaenopsis-gene615903               0    0    0    0    0   0
Phalaenopsis-gene615904               1    0    1    1    0   3
Phalaenopsis-gene615968               1    0    0    1    0   2
Phalaenopsis-gene615969               0    0    1    0    1   2
Phalaenopsis-gene616025               1    0    0    1    0   2
Phalaenopsis-gene616026               0    0    0    0    0   0
Phalaenopsis-gene620413               1    0    1    1    0   3
Phalaenopsis-gene620920               1    1    1    1    1   5
Phalaenopsis-gene620921               0    0    1    1    0   2
Phalaenopsis-gene620930               1    0    1    1    1   4
Phalaenopsis-gene621495               1    0    1    1    0   3
Phalaenopsis-gene621609               1    1    1    1    1   5
Phalaenopsis-gene621672               1    1    1    1    1   5
Phalaenopsis-gene621706               0    0    1    0    0   1
Phalaenopsis-gene621709               1    1    1    1    1   5
Phalaenopsis-gene62333                1    1    0    0    0   2
Phalaenopsis-gene623353               1    0    1    1    0   3
Phalaenopsis-gene623606               0    0    1    0    0   1
Phalaenopsis-gene623655               1    0    1    1    0   3
Phalaenopsis-gene623792               1    0    0    0    0   1
Phalaenopsis-gene627019               1    0    0    1    0   2
Phalaenopsis-gene627444               1    1    1    1    1   5
Phalaenopsis-gene627445               1    0    1    1    0   3
Phalaenopsis-gene627919               1    1    1    1    1   5
Phalaenopsis-gene632034               0    0    0    1    0   1
Phalaenopsis-gene632991               1    1    1    1    1   5
Phalaenopsis-gene63386                1    0    0    1    0   2
Phalaenopsis-gene644319               1    1    0    0    0   2
Phalaenopsis-gene644660               1    0    0    1    0   2
Phalaenopsis-gene644704               1    0    1    1    0   3
Phalaenopsis-gene644705               0    0    0    1    0   1
Phalaenopsis-gene644740               0    0    1    1    0   2
Phalaenopsis-gene644741               1    0    0    0    0   1
Phalaenopsis-gene644782               1    0    1    1    0   3
Phalaenopsis-gene644783               1    1    0    1    0   3
Phalaenopsis-gene645274               1    0    0    1    0   2
Phalaenopsis-gene645280               1    1    1    1    0   4
Phalaenopsis-gene645697               1    1    1    1    1   5
Phalaenopsis-gene647231               1    1    1    1    1   5
Phalaenopsis-gene647270               1    1    1    0    0   3
Phalaenopsis-gene647299               1    0    0    1    0   2
Phalaenopsis-gene657008               1    0    1    1    0   3
Phalaenopsis-gene657769               1    0    0    1    0   2
Phalaenopsis-gene657836               1    1    0    1    0   3
Phalaenopsis-gene659397               0    0    0    1    0   1
Phalaenopsis-gene66010                0    0    1    1    0   2
Phalaenopsis-gene662855               1    0    1    1    0   3
Phalaenopsis-gene662898               1    0    1    1    0   3
Phalaenopsis-gene66319                1    0    1    1    0   3
Phalaenopsis-gene664814               1    0    1    1    0   3
Phalaenopsis-gene664949               0    0    1    1    0   2
Phalaenopsis-gene664950               1    0    1    0    0   2
Phalaenopsis-gene667416               1    1    1    1    1   5
Phalaenopsis-gene668365               0    1    0    1    0   2
Phalaenopsis-gene668424               1    1    0    0    0   2
Phalaenopsis-gene668716               1    0    1    0    0   2
Phalaenopsis-gene670095               1    0    1    1    1   4
Phalaenopsis-gene670138               0    0    1    0    0   1
Phalaenopsis-gene676914               0    0    0    1    0   1
Phalaenopsis-gene676915               0    0    1    0    0   1
Phalaenopsis-gene676948               1    0    0    1    0   2
Phalaenopsis-gene676949               1    0    1    0    0   2
Phalaenopsis-gene677026               0    0    0    1    0   1
Phalaenopsis-gene677061               1    0    1    1    0   3
Phalaenopsis-gene677106               1    0    0    0    0   1
Phalaenopsis-gene677150               1    0    0    0    0   1
Phalaenopsis-gene677329               0    0    1    1    0   2
Phalaenopsis-gene677396               1    0    0    0    0   1
Phalaenopsis-gene677404               1    1    1    1    1   5
Phalaenopsis-gene677405               0    1    1    1    1   4
Phalaenopsis-gene679201               1    1    1    1    1   5
Phalaenopsis-gene679278               1    1    1    1    1   5
Phalaenopsis-gene679839               1    1    1    1    1   5
Phalaenopsis-gene679840               0    1    1    0    0   2
Phalaenopsis-gene679846               1    1    1    1    1   5
Phalaenopsis-gene679847               1    0    0    1    0   2
Phalaenopsis-gene679848               0    0    1    1    0   2
Phalaenopsis-gene681097               1    1    1    1    1   5
Phalaenopsis-gene682137               1    1    1    1    1   5
Phalaenopsis-gene685304               0    0    1    1    0   2
Phalaenopsis-gene685305               1    1    1    1    1   5
Phalaenopsis-gene685473               1    0    0    1    0   2
Phalaenopsis-gene685474               1    1    1    1    1   5
Phalaenopsis-gene685745               1    1    1    1    1   5
Phalaenopsis-gene685751               1    0    1    1    0   3
Phalaenopsis-gene685770               1    0    1    1    0   3
Phalaenopsis-gene685771               0    0    0    1    0   1
Phalaenopsis-gene685796               1    1    1    1    0   4
Phalaenopsis-gene685797               1    1    1    1    1   5
Phalaenopsis-gene686850               1    1    1    1    1   5
Phalaenopsis-gene68972                1    1    1    1    1   5
Phalaenopsis-gene689892               1    1    1    1    1   5
Phalaenopsis-gene693166               1    0    0    1    0   2
Phalaenopsis-gene693212               1    0    0    0    0   1
Phalaenopsis-gene694125               1    0    1    1    0   3
Phalaenopsis-gene694134               1    0    0    1    0   2
Phalaenopsis-gene694166               1    1    1    1    1   5
Phalaenopsis-gene694216               1    0    0    1    0   2
Phalaenopsis-gene696571               1    1    1    1    0   4
Phalaenopsis-gene697312               1    1    0    0    0   2
Phalaenopsis-gene697313               1    0    1    1    0   3
Phalaenopsis-gene697363               1    0    1    0    0   2
Phalaenopsis-gene697364               1    0    0    1    0   2
Phalaenopsis-gene697719               1    1    1    1    0   4
Phalaenopsis-gene697891               1    1    1    1    1   5
Phalaenopsis-gene697974               0    0    1    0    0   1
Phalaenopsis-gene698042               1    0    1    0    0   2
Phalaenopsis-gene698111               0    0    1    1    0   2
Phalaenopsis-gene698179               1    1    1    1    1   5
Phalaenopsis-gene698586               0    0    0    1    0   1
Phalaenopsis-gene698617               0    0    0    1    0   1
Phalaenopsis-gene698986               0    0    0    1    0   1
Phalaenopsis-gene701492               1    0    1    1    0   3
Phalaenopsis-gene704589               1    1    1    1    0   4
Phalaenopsis-gene706700               1    0    1    1    0   3
Phalaenopsis-gene709615               1    1    1    1    1   5
Phalaenopsis-gene710162               1    1    1    1    1   5
Phalaenopsis-gene710163               1    1    1    1    1   5
Phalaenopsis-gene712397               0    0    0    1    0   1
Phalaenopsis-gene713925               1    1    1    1    1   5
Phalaenopsis-gene714274               1    1    1    1    1   5
Phalaenopsis-gene716258               1    0    0    1    0   2
Phalaenopsis-gene71657                1    1    1    1    1   5
Phalaenopsis-gene71660                1    1    1    1    1   5
Phalaenopsis-gene718790               0    0    1    0    0   1
Phalaenopsis-gene718823               1    0    1    0    0   2
Phalaenopsis-gene718824               0    0    0    0    0   0
Phalaenopsis-gene72440                1    1    0    1    0   3
Phalaenopsis-gene725474               0    1    0    0    0   1
Phalaenopsis-gene726144               1    1    1    0    0   3
Phalaenopsis-gene726174               1    1    1    1    1   5
Phalaenopsis-gene727633               0    0    1    1    0   2
Phalaenopsis-gene727634               1    0    1    0    0   2
Phalaenopsis-gene727662               1    1    1    1    1   5
Phalaenopsis-gene729471               0    0    1    0    0   1
Phalaenopsis-gene731540               1    0    0    1    1   3
Phalaenopsis-gene733039               0    0    0    1    0   1
Phalaenopsis-gene733044               1    0    1    0    0   2
Phalaenopsis-gene733603               1    1    1    1    1   5
Phalaenopsis-gene734949               1    1    1    1    1   5
Phalaenopsis-gene735125               1    1    1    1    1   5
Phalaenopsis-gene737075               1    1    1    1    1   5
Phalaenopsis-gene741589               1    0    0    1    0   2
Phalaenopsis-gene741610               1    1    0    1    0   3
Phalaenopsis-gene742432               0    0    1    1    0   2
Phalaenopsis-gene744083               1    1    1    1    1   5
Phalaenopsis-gene744091               1    0    1    1    0   3
Phalaenopsis-gene745976               1    1    1    1    1   5
Phalaenopsis-gene745977               1    1    1    0    0   3
Phalaenopsis-gene746004               1    0    1    1    0   3
Phalaenopsis-gene746005               1    1    1    1    1   5
Phalaenopsis-gene746006               1    0    1    0    0   2
Phalaenopsis-gene747856               1    1    1    1    1   5
Phalaenopsis-gene747863               1    1    1    1    1   5
Phalaenopsis-gene750326               1    1    1    1    1   5
Phalaenopsis-gene750327               1    1    1    1    1   5
Phalaenopsis-gene75248                1    1    1    1    0   4
Phalaenopsis-gene753815               1    0    0    1    0   2
Phalaenopsis-gene753946               0    0    0    0    0   0
Phalaenopsis-gene754070               1    0    1    1    0   3
Phalaenopsis-gene754071               1    1    1    1    1   5
Phalaenopsis-gene754192               0    0    0    1    0   1
Phalaenopsis-gene754643               1    0    0    1    0   2
Phalaenopsis-gene754786               1    0    1    0    0   2
Phalaenopsis-gene755140               1    0    1    0    0   2
Phalaenopsis-gene755141               1    0    1    1    0   3
Phalaenopsis-gene757853               1    0    1    1    0   3
Phalaenopsis-gene762704               1    0    1    1    0   3
Phalaenopsis-gene763175               0    0    1    1    0   2
Phalaenopsis-gene763217               1    1    1    1    1   5
Phalaenopsis-gene763260               0    0    1    1    0   2
Phalaenopsis-gene763261               0    0    1    0    0   1
Phalaenopsis-gene764035               0    0    0    0    0   0
Phalaenopsis-gene764113               1    0    0    1    0   2
Phalaenopsis-gene764194               1    1    1    1    1   5
Phalaenopsis-gene766030               1    0    0    1    0   2
Phalaenopsis-gene766593               1    1    1    1    1   5
Phalaenopsis-gene767197               1    1    1    1    1   5
Phalaenopsis-gene772647               0    0    0    0    0   0
Phalaenopsis-gene772746               1    0    1    1    0   3
Phalaenopsis-gene773092               1    1    1    1    1   5
Phalaenopsis-gene773880               1    0    0    1    0   2
Phalaenopsis-gene773890               0    0    0    1    0   1
Phalaenopsis-gene773912               1    0    1    1    0   3
Phalaenopsis-gene773923               1    0    0    1    0   2
Phalaenopsis-gene774104               1    0    1    1    0   3
Phalaenopsis-gene774314               0    1    1    1    0   3
Phalaenopsis-gene774315               1    0    1    1    0   3
Phalaenopsis-gene774368               0    0    1    1    0   2
Phalaenopsis-gene774369               1    0    1    1    0   3
Phalaenopsis-gene776189               1    0    0    1    0   2
Phalaenopsis-gene776197               1    1    1    1    1   5
Phalaenopsis-gene783384               1    0    0    0    0   1
Phalaenopsis-gene78464                1    0    1    0    0   2
Phalaenopsis-gene78468                1    1    1    1    1   5
Phalaenopsis-gene78469                1    0    0    1    0   2
Phalaenopsis-gene786207               1    1    1    1    1   5
Phalaenopsis-gene790764               1    0    0    1    0   2
Phalaenopsis-gene790943               1    0    0    1    0   2
Phalaenopsis-gene792505               0    0    0    1    0   1
Phalaenopsis-gene792621               1    0    0    1    0   2
Phalaenopsis-gene792655               1    0    0    1    0   2
Phalaenopsis-gene806407               1    1    1    1    1   5
Phalaenopsis-gene806408               0    0    1    1    0   2
Phalaenopsis-gene806409               1    1    1    1    1   5
Phalaenopsis-gene806410               0    0    1    0    0   1
Phalaenopsis-gene806411               1    1    1    1    1   5
Phalaenopsis-gene806425               1    1    1    1    1   5
Phalaenopsis-gene81194                1    1    1    1    1   5
Phalaenopsis-gene812145               1    0    1    1    0   3
Phalaenopsis-gene812197               0    0    1    0    0   1
Phalaenopsis-gene812702               1    1    1    1    1   5
Phalaenopsis-gene814277               1    1    1    1    1   5
Phalaenopsis-gene814569               1    0    1    1    0   3
Phalaenopsis-gene814570               1    1    1    1    1   5
Phalaenopsis-gene814613               1    0    1    1    0   3
Phalaenopsis-gene817571               1    0    1    0    0   2
Phalaenopsis-gene817642               0    0    0    0    0   0
Phalaenopsis-gene818100               1    0    0    0    0   1
Phalaenopsis-gene819559               1    1    1    1    1   5
Phalaenopsis-gene819629               1    0    0    0    0   1
Phalaenopsis-gene819679               1    0    0    0    0   1
Phalaenopsis-gene820051               0    0    0    1    0   1
Phalaenopsis-gene820106               1    0    0    1    0   2
Phalaenopsis-gene822783               0    0    0    1    0   1
Phalaenopsis-gene822784               0    0    0    1    0   1
Phalaenopsis-gene82416                0    0    0    1    0   1
Phalaenopsis-gene824988               0    0    1    0    0   1
Phalaenopsis-gene825725               1    1    1    1    1   5
Phalaenopsis-gene825951               1    0    1    1    0   3
Phalaenopsis-gene830511               1    1    1    1    1   5
Phalaenopsis-gene831919               1    0    1    1    0   3
Phalaenopsis-gene835965               0    0    0    1    0   1
Phalaenopsis-gene836679               1    0    1    1    0   3
Phalaenopsis-gene836712               1    0    1    0    0   2
Phalaenopsis-gene841833               1    0    1    0    0   2
Phalaenopsis-gene84238                1    0    1    1    0   3
Phalaenopsis-gene842831               0    0    1    0    0   1
Phalaenopsis-gene84819                1    1    1    1    1   5
Phalaenopsis-gene84824                1    1    1    1    1   5
Phalaenopsis-gene851452               1    1    1    1    1   5
Phalaenopsis-gene851757               0    0    0    0    0   0
Phalaenopsis-gene851790               1    0    1    1    1   4
Phalaenopsis-gene851792               1    1    1    1    0   4
Phalaenopsis-gene851819               1    0    0    0    0   1
Phalaenopsis-gene851849               0    0    1    1    0   2
Phalaenopsis-gene851877               1    0    1    1    0   3
Phalaenopsis-gene852528               1    0    1    1    0   3
Phalaenopsis-gene852559               1    1    1    1    1   5
Phalaenopsis-gene8531                 1    1    1    1    1   5
Phalaenopsis-gene854742               1    1    1    1    1   5
Phalaenopsis-gene857240               1    0    0    1    0   2
Phalaenopsis-gene857609               1    1    1    1    1   5
Phalaenopsis-gene857636               1    1    0    0    0   2
Phalaenopsis-gene857637               1    1    1    1    0   4
Phalaenopsis-gene857652               1    0    0    1    0   2
Phalaenopsis-gene857696               1    0    0    1    0   2
Phalaenopsis-gene857697               1    0    1    0    0   2
Phalaenopsis-gene857712               1    0    0    1    0   2
Phalaenopsis-gene857812               1    0    0    1    0   2
Phalaenopsis-gene857813               1    1    0    0    0   2
Phalaenopsis-gene857828               1    0    1    0    0   2
Phalaenopsis-gene867489               1    0    0    1    0   2
Phalaenopsis-gene871579               1    1    1    1    1   5
Phalaenopsis-gene874848               1    1    1    1    0   4
Phalaenopsis-gene880853               1    1    1    1    0   4
Phalaenopsis-gene881694               1    0    1    1    0   3
Phalaenopsis-gene881712               1    1    1    1    1   5
Phalaenopsis-gene881777               1    0    0    1    0   2
Phalaenopsis-gene882146               1    1    1    1    1   5
Phalaenopsis-gene882581               0    0    1    0    0   1
Phalaenopsis-gene882582               1    1    1    1    1   5
Phalaenopsis-gene887241               0    0    0    0    0   0
Phalaenopsis-gene887288               0    0    0    1    0   1
Phalaenopsis-gene888950               1    1    1    1    1   5
Phalaenopsis-gene892038               1    0    0    1    0   2
Phalaenopsis-gene892039               0    0    1    1    0   2
Phalaenopsis-gene89274                1    1    1    1    1   5
Phalaenopsis-gene892957               1    0    1    0    0   2
Phalaenopsis-gene893989               1    0    0    0    0   1
Phalaenopsis-gene895193               1    0    1    1    0   3
Phalaenopsis-gene895201               0    0    1    1    0   2
Phalaenopsis-gene896648               1    1    1    1    1   5
Phalaenopsis-gene897676               1    1    0    1    0   3
Phalaenopsis-gene897729               1    0    1    1    0   3
Phalaenopsis-gene898918               1    1    1    1    1   5
Phalaenopsis-gene899796               1    1    0    1    0   3
Phalaenopsis-gene901947               1    0    1    1    1   4
Phalaenopsis-gene903077               1    0    0    1    1   3
Phalaenopsis-gene904182               0    0    1    1    0   2
Phalaenopsis-gene904229               1    0    1    1    0   3
Phalaenopsis-gene904595               0    0    0    0    0   0
Phalaenopsis-gene906989               1    1    1    1    1   5
Phalaenopsis-gene908070               1    0    0    1    0   2
Phalaenopsis-gene94004                1    0    0    0    0   1
Phalaenopsis-gene95957                1    0    1    1    0   3
Phalaenopsis-gene95982                1    0    1    1    0   3
Phalaenopsis-gene96062                1    1    1    1    1   5
Phalaenopsis-gene97148                1    0    1    1    0   3
Ophrys-gene5282                       1    0    0    1    0   2
Ophrys-gene20228                      1    0    0    1    0   2
Ophrys-gene21543                      0    1    0    0    0   1
Ophrys-gene23537                      1    1    1    0    0   3
Ophrys-gene24977                      1    1    1    1    1   5
Ophrys-gene46806                      0    0    0    1    0   1
Ophrys-gene55261                      1    1    1    1    1   5
Ophrys-gene67443                      0    0    0    1    0   1
Ophrys-gene68625                      1    0    0    1    0   2
Ophrys-gene68700                      1    1    1    1    1   5
Ophrys-gene99965                      1    1    1    1    1   5
Ophrys-gene114626                     1    1    1    1    1   5
Ophrys-gene133875                     1    1    1    1    1   5
Ophrys-gene138184                     1    1    1    1    0   4
Ophrys-gene140678                     1    0    1    0    0   2
Ophrys-gene167697                     1    1    1    1    1   5
Ophrys-gene168566                     1    1    1    1    1   5
Ophrys-gene173495                     1    1    1    1    1   5
Ophrys-gene203696                     1    0    0    1    0   2
Ophrys-gene203717                     1    1    1    1    1   5
Ophrys-gene214613                     1    1    1    1    1   5
Ophrys-gene215355                     1    0    0    1    0   2
Ophrys-gene258769                     1    0    1    0    0   2
Ophrys-gene263284                     1    1    1    1    1   5
Ophrys-gene268035                     1    1    1    1    1   5
Ophrys-gene287013                     0    0    0    0    0   0
Ophrys-gene342492                     1    1    1    1    1   5
Ophrys-gene429080                     1    1    1    1    1   5
Ophrys-gene434946                     1    1    1    1    1   5
Ophrys-gene438751                     1    0    0    1    0   2
Ophrys-gene525701                     1    1    1    1    1   5
Ophrys-gene554494                     0    0    0    1    0   1
Ophrys-gene565169                     1    1    0    1    0   3
Ophrys-gene565172                     1    1    1    1    1   5
Ophrys-gene566347                     1    0    1    1    0   3
Ophrys-gene586883                     1    0    0    1    0   2
Ophrys-gene608262                     1    1    1    1    1   5
Ophrys-gene677093                     1    0    0    1    0   2
Ophrys-gene757562                     1    1    0    1    0   3
Ophrys-gene758888                     1    0    0    1    0   2
Ophrys-gene6009                       0    0    0    1    0   1
Ophrys-gene102176                     1    0    0    0    0   1
Ophrys-gene663409                     1    1    1    1    1   5
Ophrys-gene721829                     1    1    1    1    1   5
Ophrys-gene2022                       1    1    1    1    1   5
Ophrys-gene4714                       1    1    1    1    1   5
Ophrys-gene52665                      0    0    1    0    0   1
Ophrys-gene71646                      1    0    0    1    0   2
Ophrys-gene71835                      1    0    0    1    0   2
Ophrys-gene75180                      1    0    1    1    1   4
Ophrys-gene76937                      1    0    0    1    0   2
Ophrys-gene80983                      1    0    1    1    0   3
Ophrys-gene81683                      1    1    1    1    1   5
Ophrys-gene96317                      1    1    1    1    1   5
Ophrys-gene120864                     1    1    1    1    1   5
Ophrys-gene134883                     1    1    1    1    1   5
Ophrys-gene168065                     1    1    0    1    0   3
Ophrys-gene173582                     1    0    1    0    0   2
Ophrys-gene180212                     1    1    1    1    1   5
Ophrys-gene182852                     1    0    0    1    0   2
Ophrys-gene190715                     0    1    0    1    0   2
Ophrys-gene190752                     1    1    1    1    1   5
Ophrys-gene205895                     0    0    0    1    0   1
Ophrys-gene209350                     0    0    0    1    0   1
Ophrys-gene211080                     1    1    1    1    1   5
Ophrys-gene211135                     1    0    1    1    1   4
Ophrys-gene213725                     1    1    1    1    1   5
Ophrys-gene219243                     0    1    0    1    0   2
Ophrys-gene226902                     1    0    1    0    0   2
Ophrys-gene239092                     0    0    0    1    0   1
Ophrys-gene249758                     1    1    1    1    1   5
Ophrys-gene268036                     1    1    1    1    1   5
Ophrys-gene270092                     1    0    1    1    0   3
Ophrys-gene274531                     1    1    1    1    1   5
Ophrys-gene383106                     1    0    0    0    1   2
Ophrys-gene383406                     0    1    0    1    1   3
Ophrys-gene392569                     1    0    0    1    0   2
Ophrys-gene399316                     0    0    1    1    0   2
Ophrys-gene412942                     1    0    1    1    0   3
Ophrys-gene421167                     1    0    0    0    0   1
Ophrys-gene428740                     1    1    1    1    1   5
Ophrys-gene444781                     1    1    0    1    0   3
Ophrys-gene469276                     1    0    0    1    0   2
Ophrys-gene477585                     1    1    0    0    0   2
Ophrys-gene492447                     1    0    1    1    0   3
Ophrys-gene495563                     1    0    0    0    0   1
Ophrys-gene499783                     1    0    0    1    0   2
Ophrys-gene510247                     1    1    1    1    1   5
Ophrys-gene512379                     1    1    1    1    1   5
Ophrys-gene518272                     1    1    1    1    1   5
Ophrys-gene519093                     1    0    0    1    0   2
Ophrys-gene525700                     1    0    1    0    0   2
Ophrys-gene526009                     1    1    1    1    1   5
Ophrys-gene568024                     1    0    0    0    0   1
Ophrys-gene571461                     0    0    0    1    0   1
Ophrys-gene586732                     0    0    0    0    0   0
Ophrys-gene635319                     1    0    1    1    0   3
Ophrys-gene645075                     1    1    1    1    1   5
Ophrys-gene650958                     1    1    1    1    1   5
Ophrys-gene684265                     1    0    1    1    0   3
Ophrys-gene700216                     1    0    1    1    0   3
Ophrys-gene757382                     1    0    1    1    0   3
Ophrys-gene181543                     0    0    0    0    0   0
Ophrys-gene288267                     1    1    1    1    1   5
Ophrys-gene395164                     1    0    0    1    0   2
Ophrys-gene395165                     0    0    0    1    0   1
Ophrys-gene710844                     1    1    1    1    1   5
Ophrys-gene2421                       1    1    1    1    1   5
Ophrys-gene45666                      1    1    1    0    0   3
Ophrys-gene72077                      1    0    0    1    0   2
Ophrys-gene134819                     1    0    0    1    0   2
Ophrys-gene134898                     1    1    1    1    1   5
Ophrys-gene141670                     1    1    1    1    1   5
Ophrys-gene180201                     1    0    1    1    0   3
Ophrys-gene190748                     1    1    1    1    1   5
Ophrys-gene199469                     1    1    1    1    1   5
Ophrys-gene205935                     1    1    0    1    0   3
Ophrys-gene258501                     1    0    0    1    0   2
Ophrys-gene271120                     0    1    0    1    0   2
Ophrys-gene288718                     1    0    0    1    0   2
Ophrys-gene291007                     0    0    1    1    0   2
Ophrys-gene348909                     1    0    1    0    0   2
Ophrys-gene375172                     1    1    1    1    1   5
Ophrys-gene444895                     1    1    1    0    0   3
Ophrys-gene473589                     0    0    1    0    0   1
Ophrys-gene480784                     1    1    1    1    1   5
Ophrys-gene511147                     0    1    1    0    0   2
Ophrys-gene543925                     1    1    1    1    1   5
Ophrys-gene555940                     1    1    1    1    1   5
Ophrys-gene579503                     1    0    0    1    0   2
Ophrys-gene606513                     1    1    1    1    1   5
Ophrys-gene640148                     1    1    1    0    0   3
Ophrys-gene692709                     0    0    1    1    0   2
Ophrys-gene693244                     1    1    1    1    1   5
Ophrys-gene703150                     1    0    0    0    0   1
Ophrys-gene708642                     1    1    1    1    1   5
Ophrys-gene739896                     1    0    0    1    1   3
Ophrys-gene596332                     1    0    0    0    0   1
Ophrys-gene60913                      0    0    1    0    0   1
Ophrys-gene183140                     1    1    1    1    1   5
Ophrys-gene245752                     1    1    1    1    1   5
Ophrys-gene681269                     1    0    0    1    0   2
Ophrys-gene2345                       1    1    1    1    1   5
Ophrys-gene112865                     1    1    1    1    1   5
Ophrys-gene126228                     1    1    1    1    1   5
Ophrys-gene159101                     1    0    1    0    0   2
Ophrys-gene187062                     0    0    1    1    0   2
Ophrys-gene194120                     1    0    0    1    0   2
Ophrys-gene212077                     1    1    1    1    1   5
Ophrys-gene213249                     1    0    1    1    0   3
Ophrys-gene240125                     1    0    1    0    0   2
Ophrys-gene304742                     0    0    1    0    0   1
Ophrys-gene375074                     1    0    1    1    0   3
Ophrys-gene386184                     1    0    1    1    0   3
Ophrys-gene386349                     1    1    1    1    1   5
Ophrys-gene511192                     1    1    1    1    1   5
Ophrys-gene555928                     1    1    1    1    1   5
Ophrys-gene586881                     1    0    0    1    0   2
Ophrys-gene698652                     1    0    1    1    0   3
Ophrys-gene731485                     0    1    0    1    0   2
Ophrys-gene731984                     1    0    1    1    0   3
Ophrys-gene6780                       1    0    0    1    0   2
Ophrys-gene11520                      1    0    1    0    0   2
Ophrys-gene20068                      1    1    1    1    1   5
Ophrys-gene23069                      1    1    1    1    1   5
Ophrys-gene56710                      0    0    0    0    0   0
Ophrys-gene71676                      1    0    1    1    1   4
Ophrys-gene86838                      1    1    1    1    0   4
Ophrys-gene99885                      1    0    0    0    0   1
Ophrys-gene167040                     1    1    0    1    0   3
Ophrys-gene173260                     0    0    0    1    0   1
Ophrys-gene184023                     1    0    0    0    0   1
Ophrys-gene190717                     1    1    1    1    1   5
Ophrys-gene194966                     1    1    1    1    1   5
Ophrys-gene210370                     0    0    0    0    0   0
Ophrys-gene258323                     1    0    0    1    0   2
Ophrys-gene289125                     1    1    1    1    1   5
Ophrys-gene296430                     1    0    0    0    0   1
Ophrys-gene296691                     0    0    1    1    0   2
Ophrys-gene342485                     1    0    1    1    0   3
Ophrys-gene391073                     0    0    1    0    0   1
Ophrys-gene412146                     1    1    1    1    1   5
Ophrys-gene413908                     1    0    0    1    0   2
Ophrys-gene432593                     1    1    1    1    0   4
Ophrys-gene495596                     0    0    0    1    0   1
Ophrys-gene495986                     1    0    0    1    0   2
Ophrys-gene543928                     1    1    1    1    1   5
Ophrys-gene544273                     1    1    1    1    1   5
Ophrys-gene561831                     1    0    1    1    0   3
Ophrys-gene709099                     1    1    1    1    1   5
Ophrys-gene739686                     1    0    1    0    0   2
Ophrys-gene1985                       1    0    0    0    0   1
Ophrys-gene67492                      0    0    0    0    1   1
Ophrys-gene203627                     0    0    1    1    0   2
Ophrys-gene219176                     1    0    0    1    0   2
Ophrys-gene254181                     1    0    0    1    0   2
Ophrys-gene254816                     1    1    1    1    1   5
Ophrys-gene286517                     1    0    0    1    0   2
Ophrys-gene298986                     1    0    0    1    0   2
Ophrys-gene361928                     1    1    1    1    1   5
Ophrys-gene582791                     0    0    1    1    0   2
Ophrys-gene689685                     0    0    0    0    0   0
Ophrys-gene698651                     0    0    0    0    0   0
Ophrys-gene700205                     1    1    1    1    1   5
Ophrys-gene173574                     1    1    1    1    1   5
Ophrys-gene178188                     0    0    0    1    0   1
Ophrys-gene446299                     1    1    1    1    1   5
Ophrys-gene595841                     1    0    0    0    0   1
Ophrys-gene644268                     1    0    0    1    0   2
Ophrys-gene153769                     1    0    0    1    0   2
Ophrys-gene184886                     1    0    0    1    0   2
Ophrys-gene184920                     1    1    1    1    1   5
Ophrys-gene185048                     0    0    1    1    0   2
Ophrys-gene185078                     1    0    1    1    0   3
Ophrys-gene185107                     0    0    1    1    0   2
Ophrys-gene619124                     1    1    1    1    1   5
Ophrys-gene5528                       0    1    0    1    0   2
Ophrys-gene58698                      1    0    0    1    0   2
Ophrys-gene69885                      1    1    1    1    1   5
Ophrys-gene84007                      1    0    1    0    1   3
Ophrys-gene154579                     1    0    1    1    0   3
Ophrys-gene213729                     1    0    0    1    0   2
Ophrys-gene318880                     1    0    1    0    0   2
Ophrys-gene397410                     1    0    0    0    0   1
Ophrys-gene496282                     1    0    0    1    0   2
Ophrys-gene710958                     1    1    1    1    1   5
Ophrys-gene5378                       1    0    0    0    0   1
Ophrys-gene48688                      1    1    1    1    1   5
Ophrys-gene131908                     0    0    0    0    0   0
Ophrys-gene509214                     1    1    1    1    1   5
Ophrys-gene766544                     1    0    0    0    0   1
Ophrys-gene10723                      1    0    1    1    0   3
Ophrys-gene65738                      1    0    1    0    0   2
Ophrys-gene71725                      1    1    1    0    0   3
Ophrys-gene77860                      1    0    0    1    0   2
Ophrys-gene369010                     1    0    0    1    0   2
Ophrys-gene404765                     1    1    1    1    1   5
Ophrys-gene422274                     1    1    1    1    1   5
Ophrys-gene496133                     1    1    1    1    0   4
Ophrys-gene677077                     1    1    1    1    1   5
Ophrys-gene708976                     1    1    1    1    1   5
Ophrys-gene297042                     1    0    0    1    0   2
Ophrys-gene452394                     0    0    0    1    0   1
Ophrys-gene545809                     1    1    1    1    1   5
Ophrys-gene595774                     1    0    0    1    0   2
Ophrys-gene709239                     1    0    0    1    0   2
Ophrys-gene3520                       1    1    0    0    0   2
Ophrys-gene383401                     1    0    0    1    0   2
Ophrys-gene644324                     1    0    0    1    0   2
Ophrys-gene21385                      1    0    1    1    0   3
Ophrys-gene162740                     1    1    1    1    1   5
Ophrys-gene178871                     1    0    0    1    0   2
Ophrys-gene195126                     1    0    0    1    1   3
Ophrys-gene207962                     0    0    1    1    0   2
Ophrys-gene301995                     1    0    0    1    0   2
Ophrys-gene304064                     1    1    1    1    1   5
Ophrys-gene441815                     1    0    0    1    0   2
Ophrys-gene526308                     1    1    1    1    1   5
Ophrys-gene651223                     1    0    1    0    0   2
Ophrys-gene11741                      1    0    1    0    0   2
Ophrys-gene22541                      1    1    1    1    1   5
Ophrys-gene54161                      1    0    1    1    0   3
Ophrys-gene76328                      1    1    1    1    1   5
Ophrys-gene183178                     1    1    1    1    1   5
Ophrys-gene217195                     1    1    1    1    1   5
Ophrys-gene258494                     0    0    0    0    0   0
Ophrys-gene287274                     1    1    1    1    1   5
Ophrys-gene390951                     0    0    0    1    0   1
Ophrys-gene392558                     1    0    0    1    0   2
Ophrys-gene401533                     1    1    1    1    1   5
Ophrys-gene425609                     0    1    0    1    0   2
Ophrys-gene427699                     1    1    1    1    1   5
Ophrys-gene528783                     0    0    0    0    0   0
Ophrys-gene709281                     1    0    0    1    0   2
Ophrys-gene715209                     1    1    1    1    1   5
Ophrys-gene715329                     0    0    0    1    0   1
Ophrys-gene69911                      1    1    1    1    1   5
Ophrys-gene95167                      1    1    1    1    1   5
Ophrys-gene139720                     1    0    1    1    0   3
Ophrys-gene149973                     1    1    1    1    1   5
Ophrys-gene178478                     1    0    0    0    0   1
Ophrys-gene352183                     0    0    1    1    0   2
Ophrys-gene496340                     0    0    0    1    0   1
Ophrys-gene510329                     1    1    1    1    1   5
Ophrys-gene542207                     1    1    1    1    1   5
Ophrys-gene601284                     1    0    0    1    0   2
Ophrys-gene635318                     0    0    1    1    0   2
Ophrys-gene683100                     0    0    1    1    0   2
Ophrys-gene709217                     0    0    0    0    1   1
Ophrys-gene295607                     1    0    1    1    0   3
Ophrys-gene304006                     1    0    0    1    0   2
Ophrys-gene419058                     1    1    1    1    1   5
Ophrys-gene436059                     1    0    0    1    0   2
Ophrys-gene445424                     1    1    1    1    1   5
Ophrys-gene445536                     1    1    1    1    1   5
Ophrys-gene488874                     1    1    1    1    1   5
Ophrys-gene596376                     0    0    0    1    0   1
Ophrys-gene99882                      0    0    0    1    0   1
Ophrys-gene99962                      1    0    0    0    0   1
Ophrys-gene605282                     1    0    0    0    0   1
Ophrys-gene167714                     1    1    1    1    1   5
Ophrys-gene187150                     1    1    1    1    1   5
Ophrys-gene305043                     0    0    0    0    0   0
Ophrys-gene345449                     1    0    1    1    0   3
Ophrys-gene710588                     1    0    1    1    0   3
Ophrys-gene283148                     1    1    1    1    1   5
Ophrys-gene582790                     0    0    1    1    0   2
Ophrys-gene349639                     1    0    0    1    0   2
Ophrys-gene498154                     1    0    1    1    0   3
Ophrys-gene549038                     1    0    1    1    0   3
Ophrys-gene21061                      1    1    1    1    1   5
Ophrys-gene76318                      1    0    0    1    0   2
Ophrys-gene252638                     1    0    1    0    0   2
Ophrys-gene597497                     0    0    0    0    0   0
Ophrys-gene616237                     1    0    1    0    0   2
Ophrys-gene102123                     0    0    1    1    0   2
Ophrys-gene102621                     1    1    1    1    1   5
Ophrys-gene155311                     1    1    1    1    1   5
Ophrys-gene295798                     1    1    1    1    0   4
Ophrys-gene431789                     1    0    1    0    0   2
Ophrys-gene444834                     1    0    0    1    0   2
Ophrys-gene445139                     1    0    0    1    0   2
Ophrys-gene538859                     1    0    1    0    0   2
Ophrys-gene543989                     1    1    1    1    1   5
Ophrys-gene6065                       1    1    1    1    1   5
Ophrys-gene113104                     1    1    1    1    1   5
Ophrys-gene366799                     1    1    1    1    1   5
Ophrys-gene436310                     1    1    0    0    0   2
Ophrys-gene436398                     1    1    0    1    0   3
Ophrys-gene495599                     1    0    0    1    0   2
Ophrys-gene717279                     1    1    1    1    1   5
Ophrys-gene140803                     0    0    0    0    0   0
Ophrys-gene169453                     1    1    1    1    1   5
Ophrys-gene268120                     0    0    0    0    0   0
Ophrys-gene284595                     1    0    1    1    0   3
Ophrys-gene301731                     1    0    0    1    0   2
Ophrys-gene405060                     1    1    0    1    0   3
Ophrys-gene425004                     1    1    1    1    1   5
Ophrys-gene425574                     1    0    0    1    0   2
Ophrys-gene445462                     1    0    1    1    0   3
Ophrys-gene764379                     1    0    0    1    1   3
Ophrys-gene274533                     0    0    1    1    0   2
Ophrys-gene431753                     1    1    1    1    1   5
Ophrys-gene489222                     1    1    1    1    1   5
Ophrys-gene601276                     0    0    0    0    0   0
Ophrys-gene2864                       1    1    1    1    1   5
Ophrys-gene27                         0    0    1    1    0   2
Ophrys-gene919                        0    0    1    0    0   1
Ophrys-gene922                        1    1    1    1    1   5
Ophrys-gene1180                       1    1    1    1    1   5
Ophrys-gene2176                       1    0    1    1    1   4
Ophrys-gene4223                       1    1    1    1    1   5
Ophrys-gene4695                       1    1    1    1    1   5
Ophrys-gene4891                       1    0    0    0    0   1
Ophrys-gene6078                       0    0    0    1    0   1
Ophrys-gene7876                       1    0    1    0    0   2
Ophrys-gene7877                       1    0    1    1    0   3
Ophrys-gene8636                       1    0    0    1    0   2
Ophrys-gene9230                       1    0    1    1    0   3
Ophrys-gene9774                       1    1    1    1    1   5
Ophrys-gene10103                      1    1    1    1    1   5
Ophrys-gene10477                      0    1    1    1    0   3
Ophrys-gene10911                      0    1    0    1    0   2
Ophrys-gene11768                      1    1    0    1    0   3
Ophrys-gene11859                      1    0    0    0    1   2
Ophrys-gene12114                      1    1    1    1    1   5
Ophrys-gene23106                      1    1    1    1    1   5
Ophrys-gene23220                      0    0    0    0    0   0
Ophrys-gene24978                      1    0    0    1    0   2
Ophrys-gene47746                      1    1    1    1    1   5
Ophrys-gene47991                      1    0    0    1    0   2
Ophrys-gene49936                      1    0    0    1    0   2
Ophrys-gene50733                      1    0    0    1    0   2
Ophrys-gene50720                      1    0    0    1    0   2
Ophrys-gene50838                      1    0    1    1    0   3
Ophrys-gene52664                      0    0    0    1    0   1
Ophrys-gene54883                      1    0    0    0    0   1
Ophrys-gene56613                      1    1    1    1    1   5
Ophrys-gene56621                      1    0    1    1    0   3
Ophrys-gene61418                      1    0    1    1    0   3
Ophrys-gene64023                      1    0    0    1    0   2
Ophrys-gene67098                      1    1    1    1    1   5
Ophrys-gene67496                      1    0    0    1    0   2
Ophrys-gene68624                      1    1    1    1    1   5
Ophrys-gene68699                      1    1    1    1    1   5
Ophrys-gene70006                      0    0    0    1    0   1
Ophrys-gene71700                      1    0    0    1    0   2
Ophrys-gene71816                      1    1    1    1    1   5
Ophrys-gene72076                      0    0    0    1    0   1
Ophrys-gene72122                      1    0    0    1    0   2
Ophrys-gene73969                      1    0    1    1    0   3
Ophrys-gene76256                      1    0    1    1    0   3
Ophrys-gene78240                      1    0    1    1    0   3
Ophrys-gene78241                      1    1    1    1    1   5
Ophrys-gene81482                      1    1    1    1    1   5
Ophrys-gene82087                      1    1    1    1    1   5
Ophrys-gene84009                      0    0    0    1    0   1
Ophrys-gene84010                      0    0    1    1    0   2
Ophrys-gene84001                      1    0    0    0    0   1
Ophrys-gene85595                      1    0    0    1    0   2
Ophrys-gene97674                      1    0    1    0    0   2
Ophrys-gene100622                     1    1    1    1    1   5
Ophrys-gene100857                     1    1    1    1    1   5
Ophrys-gene103997                     0    0    1    0    0   1
Ophrys-gene112874                     1    1    1    1    1   5
Ophrys-gene112866                     1    1    1    1    1   5
Ophrys-gene118634                     0    1    0    1    0   2
Ophrys-gene120868                     1    1    1    1    1   5
Ophrys-gene123204                     1    0    1    1    0   3
Ophrys-gene124306                     1    1    0    1    0   3
Ophrys-gene127056                     1    1    1    1    1   5
Ophrys-gene132030                     1    1    1    1    1   5
Ophrys-gene132961                     0    0    0    1    0   1
Ophrys-gene133880                     1    1    1    1    1   5
Ophrys-gene137142                     1    0    0    1    0   2
Ophrys-gene138457                     1    0    0    1    0   2
Ophrys-gene139730                     1    1    1    1    1   5
Ophrys-gene140666                     1    0    0    0    0   1
Ophrys-gene140667                     1    0    1    0    0   2
Ophrys-gene141904                     1    1    1    1    1   5
Ophrys-gene144745                     1    1    1    1    1   5
Ophrys-gene145416                     1    0    0    0    0   1
Ophrys-gene151921                     1    0    0    1    0   2
Ophrys-gene154957                     1    0    1    0    0   2
Ophrys-gene155335                     1    1    1    1    1   5
Ophrys-gene155953                     1    0    1    1    0   3
Ophrys-gene157586                     1    0    0    1    0   2
Ophrys-gene160752                     1    1    1    1    1   5
Ophrys-gene160753                     1    1    1    1    1   5
Ophrys-gene160769                     1    0    0    1    0   2
Ophrys-gene160809                     1    0    0    1    0   2
Ophrys-gene160825                     1    0    0    1    0   2
Ophrys-gene167041                     0    0    0    1    0   1
Ophrys-gene167698                     1    0    1    1    0   3
Ophrys-gene167702                     1    1    1    1    1   5
Ophrys-gene170939                     1    1    1    1    1   5
Ophrys-gene173578                     1    0    0    1    0   2
Ophrys-gene174021                     0    0    0    1    0   1
Ophrys-gene176996                     1    0    0    1    0   2
Ophrys-gene177776                     1    0    1    0    0   2
Ophrys-gene177785                     1    1    1    1    1   5
Ophrys-gene178476                     1    1    1    1    1   5
Ophrys-gene180206                     0    0    1    0    0   1
Ophrys-gene180208                     1    0    0    1    0   2
Ophrys-gene180238                     1    1    1    1    1   5
Ophrys-gene182817                     1    0    0    1    0   2
Ophrys-gene182821                     1    0    0    1    0   2
Ophrys-gene182816                     1    0    0    0    0   1
Ophrys-gene182856                     1    1    1    1    1   5
Ophrys-gene182853                     1    1    1    1    1   5
Ophrys-gene183186                     1    0    0    1    0   2
Ophrys-gene183993                     1    1    1    1    1   5
Ophrys-gene184178                     0    0    0    1    0   1
Ophrys-gene184185                     1    0    0    1    0   2
Ophrys-gene186972                     1    1    1    1    0   4
Ophrys-gene190716                     1    1    0    1    0   3
Ophrys-gene199063                     1    1    1    1    1   5
Ophrys-gene203715                     1    0    0    1    0   2
Ophrys-gene203716                     1    1    1    1    1   5
Ophrys-gene203799                     0    0    0    1    0   1
Ophrys-gene203800                     1    0    0    0    0   1
Ophrys-gene208618                     0    0    1    1    0   2
Ophrys-gene209719                     1    1    1    1    1   5
Ophrys-gene210886                     1    0    1    0    0   2
Ophrys-gene211127                     0    0    0    1    0   1
Ophrys-gene215389                     1    1    1    1    1   5
Ophrys-gene216056                     1    1    1    1    1   5
Ophrys-gene217612                     1    0    1    1    0   3
Ophrys-gene219246                     1    0    0    1    0   2
Ophrys-gene223042                     1    0    1    1    0   3
Ophrys-gene223133                     1    0    1    1    0   3
Ophrys-gene224232                     1    0    1    0    0   2
Ophrys-gene226858                     1    1    1    1    1   5
Ophrys-gene236066                     0    0    1    1    0   2
Ophrys-gene240120                     0    0    1    0    0   1
Ophrys-gene240127                     1    0    0    1    0   2
Ophrys-gene250068                     1    1    1    1    1   5
Ophrys-gene258621                     1    0    0    1    0   2
Ophrys-gene259013                     1    1    1    1    1   5
Ophrys-gene263242                     1    1    1    1    1   5
Ophrys-gene263286                     1    1    1    1    1   5
Ophrys-gene263860                     1    0    1    1    0   3
Ophrys-gene263861                     0    0    0    0    0   0
Ophrys-gene263866                     1    1    1    1    1   5
Ophrys-gene265616                     0    0    1    0    0   1
Ophrys-gene265617                     1    0    1    0    0   2
Ophrys-gene265606                     1    1    1    1    1   5
Ophrys-gene270113                     1    0    1    1    0   3
Ophrys-gene271082                     1    1    1    1    1   5
Ophrys-gene278351                     1    1    1    1    1   5
Ophrys-gene278482                     1    0    0    0    0   1
Ophrys-gene280456                     0    1    1    0    0   2
Ophrys-gene280682                     1    0    0    1    0   2
Ophrys-gene281287                     1    1    1    1    1   5
Ophrys-gene284315                     1    0    0    1    0   2
Ophrys-gene284417                     1    0    1    0    0   2
Ophrys-gene284458                     0    0    0    1    0   1
Ophrys-gene284557                     1    0    1    1    1   4
Ophrys-gene284527                     0    1    0    1    0   2
Ophrys-gene284687                     1    0    1    1    0   3
Ophrys-gene286715                     1    0    1    1    0   3
Ophrys-gene287025                     1    1    1    1    1   5
Ophrys-gene287288                     1    0    1    1    0   3
Ophrys-gene288726                     1    1    1    1    0   4
Ophrys-gene289121                     1    1    1    1    1   5
Ophrys-gene291707                     1    0    1    0    0   2
Ophrys-gene293141                     1    0    0    1    0   2
Ophrys-gene293142                     0    0    0    1    0   1
Ophrys-gene293770                     0    0    0    0    0   0
Ophrys-gene293801                     0    0    1    1    0   2
Ophrys-gene295392                     1    0    1    0    0   2
Ophrys-gene295569                     0    0    0    1    0   1
Ophrys-gene296681                     1    0    0    1    1   3
Ophrys-gene298884                     1    1    1    1    1   5
Ophrys-gene303796                     1    1    1    1    1   5
Ophrys-gene306843                     1    1    1    1    1   5
Ophrys-gene316519                     1    0    0    1    0   2
Ophrys-gene338175                     0    0    0    1    0   1
Ophrys-gene338176                     0    0    1    1    0   2
Ophrys-gene341959                     1    0    1    0    0   2
Ophrys-gene342495                     1    1    1    1    1   5
Ophrys-gene342486                     1    1    0    0    0   2
Ophrys-gene347928                     1    1    1    1    1   5
Ophrys-gene347929                     1    0    1    1    0   3
Ophrys-gene355233                     1    1    1    1    1   5
Ophrys-gene375332                     1    1    1    1    0   4
Ophrys-gene375375                     0    0    0    1    0   1
Ophrys-gene375815                     1    0    1    1    0   3
Ophrys-gene376420                     1    0    1    1    0   3
Ophrys-gene377853                     1    0    0    1    0   2
Ophrys-gene378777                     1    1    1    1    1   5
Ophrys-gene378772                     1    1    1    1    1   5
Ophrys-gene385865                     1    1    1    1    1   5
Ophrys-gene385868                     1    1    1    1    1   5
Ophrys-gene386357                     1    0    0    1    0   2
Ophrys-gene390537                     1    0    0    1    0   2
Ophrys-gene392596                     1    1    0    0    0   2
Ophrys-gene395600                     1    0    1    0    0   2
Ophrys-gene398679                     1    0    1    0    0   2
Ophrys-gene403935                     1    0    0    1    0   2
Ophrys-gene403954                     0    0    0    0    0   0
Ophrys-gene410497                     1    1    1    1    1   5
Ophrys-gene421043                     1    0    0    1    0   2
Ophrys-gene421496                     1    0    0    0    0   1
Ophrys-gene422980                     1    0    0    0    0   1
Ophrys-gene425693                     1    1    1    1    1   5
Ophrys-gene427710                     1    1    1    1    1   5
Ophrys-gene429451                     1    1    0    1    0   3
Ophrys-gene429437                     1    1    1    1    1   5
Ophrys-gene431754                     1    0    1    1    0   3
Ophrys-gene438756                     1    0    0    1    0   2
Ophrys-gene444631                     1    1    1    1    1   5
Ophrys-gene445340                     1    1    1    1    0   4
Ophrys-gene446312                     1    0    0    0    0   1
Ophrys-gene446316                     1    0    1    0    0   2
Ophrys-gene446732                     1    1    1    1    1   5
Ophrys-gene460039                     1    0    1    0    0   2
Ophrys-gene467271                     1    0    0    1    0   2
Ophrys-gene470316                     1    1    1    1    1   5
Ophrys-gene479108                     1    0    0    1    0   2
Ophrys-gene479109                     1    0    0    1    0   2
Ophrys-gene483714                     1    1    1    1    1   5
Ophrys-gene485148                     1    1    1    1    1   5
Ophrys-gene487079                     0    0    0    1    0   1
Ophrys-gene490151                     1    1    1    1    1   5
Ophrys-gene491267                     0    0    1    1    0   2
Ophrys-gene491268                     1    1    1    1    1   5
Ophrys-gene493754                     1    0    1    1    0   3
Ophrys-gene495170                     1    1    1    1    1   5
Ophrys-gene495361                     1    0    0    0    0   1
Ophrys-gene495490                     1    1    1    1    1   5
Ophrys-gene495550                     1    1    0    1    0   3
Ophrys-gene495551                     1    1    0    0    0   2
Ophrys-gene495726                     1    1    1    1    1   5
Ophrys-gene495835                     0    1    0    1    0   2
Ophrys-gene495999                     1    0    1    1    0   3
Ophrys-gene496136                     1    0    1    0    0   2
Ophrys-gene496281                     1    0    0    1    0   2
Ophrys-gene496393                     1    1    1    1    1   5
Ophrys-gene496470                     1    1    0    1    0   3
Ophrys-gene496471                     0    0    0    1    0   1
Ophrys-gene496476                     0    0    0    1    0   1
Ophrys-gene496510                     1    0    1    1    0   3
Ophrys-gene496511                     1    0    0    1    0   2
Ophrys-gene496553                     1    1    1    1    1   5
Ophrys-gene496580                     1    0    0    0    0   1
Ophrys-gene496581                     1    0    0    1    0   2
Ophrys-gene496586                     1    1    1    1    1   5
Ophrys-gene505096                     1    1    1    1    0   4
Ophrys-gene507981                     1    0    1    1    0   3
Ophrys-gene508293                     1    1    1    1    1   5
Ophrys-gene508474                     1    1    1    1    1   5
Ophrys-gene509337                     0    0    0    0    0   0
Ophrys-gene510606                     1    1    1    1    1   5
Ophrys-gene515342                     1    1    1    1    1   5
Ophrys-gene515409                     1    0    0    0    0   1
Ophrys-gene516163                     1    0    0    1    0   2
Ophrys-gene520847                     0    0    0    1    0   1
Ophrys-gene523088                     0    0    0    0    0   0
Ophrys-gene525815                     1    0    1    1    0   3
Ophrys-gene525917                     1    1    1    1    1   5
Ophrys-gene525978                     1    1    1    1    1   5
Ophrys-gene526319                     0    0    0    0    0   0
Ophrys-gene531866                     1    1    1    1    0   4
Ophrys-gene536734                     0    0    0    1    0   1
Ophrys-gene536884                     1    0    0    0    0   1
Ophrys-gene542206                     1    1    1    0    0   3
Ophrys-gene543934                     0    0    1    0    0   1
Ophrys-gene554493                     1    1    1    1    1   5
Ophrys-gene559763                     1    0    0    1    0   2
Ophrys-gene561806                     1    1    0    1    0   3
Ophrys-gene563256                     1    1    1    1    1   5
Ophrys-gene564564                     1    1    1    1    1   5
Ophrys-gene564907                     1    0    0    1    0   2
Ophrys-gene566206                     1    0    0    0    0   1
Ophrys-gene566339                     1    1    0    1    0   3
Ophrys-gene566430                     1    0    0    1    0   2
Ophrys-gene570046                     1    1    1    1    1   5
Ophrys-gene570986                     1    1    1    1    1   5
Ophrys-gene571416                     1    1    1    1    0   4
Ophrys-gene579494                     1    0    1    1    0   3
Ophrys-gene579493                     1    0    1    1    0   3
Ophrys-gene579946                     1    0    0    1    0   2
Ophrys-gene585726                     1    1    0    0    0   2
Ophrys-gene586731                     1    1    1    1    1   5
Ophrys-gene586782                     1    0    0    1    0   2
Ophrys-gene586832                     1    1    1    1    1   5
Ophrys-gene586971                     1    0    0    1    0   2
Ophrys-gene597869                     1    1    1    1    1   5
Ophrys-gene601272                     0    0    0    0    0   0
Ophrys-gene603340                     1    1    1    1    1   5
Ophrys-gene607164                     1    0    1    1    0   3
Ophrys-gene613475                     1    0    0    0    0   1
Ophrys-gene613827                     1    1    1    1    1   5
Ophrys-gene613852                     0    0    0    1    0   1
Ophrys-gene613876                     0    0    0    1    0   1
Ophrys-gene615030                     1    1    1    1    1   5
Ophrys-gene616675                     1    1    1    1    1   5
Ophrys-gene617118                     1    0    0    1    0   2
Ophrys-gene617211                     1    0    0    1    0   2
Ophrys-gene617285                     0    0    1    0    0   1
Ophrys-gene636289                     1    0    0    1    0   2
Ophrys-gene641779                     1    0    0    1    0   2
Ophrys-gene642588                     1    1    1    1    1   5
Ophrys-gene644543                     1    0    0    1    0   2
Ophrys-gene649690                     1    0    0    1    0   2
Ophrys-gene654877                     1    1    1    1    0   4
Ophrys-gene655002                     1    1    1    1    1   5
Ophrys-gene658628                     1    0    0    1    0   2
Ophrys-gene660349                     1    0    0    0    0   1
Ophrys-gene660369                     1    0    1    1    0   3
Ophrys-gene661543                     1    0    0    0    0   1
Ophrys-gene661544                     1    1    1    1    1   5
Ophrys-gene661561                     1    1    1    1    1   5
Ophrys-gene662241                     1    1    1    1    1   5
Ophrys-gene663854                     1    1    1    1    1   5
Ophrys-gene683104                     1    0    0    1    0   2
Ophrys-gene696458                     1    1    0    1    0   3
Ophrys-gene700567                     1    0    1    0    0   2
Ophrys-gene700930                     1    0    0    1    0   2
Ophrys-gene701012                     1    1    1    1    1   5
Ophrys-gene701014                     1    1    1    1    1   5
Ophrys-gene702656                     1    1    1    1    1   5
Ophrys-gene702669                     0    0    0    1    0   1
Ophrys-gene709318                     0    0    1    1    0   2
Ophrys-gene711622                     1    0    1    1    0   3
Ophrys-gene712486                     1    0    1    1    0   3
Ophrys-gene715378                     1    1    1    1    1   5
Ophrys-gene731306                     0    0    1    0    0   1
Ophrys-gene731542                     0    0    1    1    0   2
Ophrys-gene731659                     1    1    1    1    1   5
Ophrys-gene732062                     1    1    1    1    1   5
Ophrys-gene735546                     1    1    1    1    1   5
Ophrys-gene746822                     1    1    1    1    1   5
Ophrys-gene747982                     1    1    1    1    1   5
Ophrys-gene747976                     1    0    1    1    0   3
Ophrys-gene753426                     1    0    0    1    0   2
Ophrys-gene757442                     0    0    1    0    0   1
Ophrys-gene757449                     0    0    0    1    0   1
Ophrys-gene757509                     1    0    0    0    0   1
Ophrys-gene757621                     1    1    1    1    0   4
Ophrys-gene757737                     1    0    1    1    0   3
Ophrys-gene757744                     1    0    0    0    0   1
Ophrys-gene762872                     1    1    1    1    1   5
Phalaenopsis-gene125355               1    0    1    1    0   3
Phalaenopsis-gene136147               1    0    0    1    0   2
Phalaenopsis-gene150164               1    0    1    0    0   2
Phalaenopsis-gene156081               1    1    1    1    1   5
Phalaenopsis-gene247250               1    0    0    0    0   1
Phalaenopsis-gene247252               1    1    1    1    1   5
Phalaenopsis-gene267050               1    0    1    0    0   2
Phalaenopsis-gene324588               0    0    1    1    0   2
Phalaenopsis-gene348729               0    0    1    1    0   2
Phalaenopsis-gene371045               1    1    1    1    1   5
Phalaenopsis-gene377238               0    0    0    1    0   1
Phalaenopsis-gene393882               1    0    1    1    0   3
Phalaenopsis-gene410157               1    1    1    1    1   5
Phalaenopsis-gene412741               1    0    0    1    0   2
Phalaenopsis-gene425652               1    0    1    0    0   2
Phalaenopsis-gene437335               1    1    1    1    1   5
Phalaenopsis-gene461860               0    0    1    1    0   2
Phalaenopsis-gene461894               1    0    1    1    0   3
Phalaenopsis-gene461930               1    1    1    1    1   5
Phalaenopsis-gene479683               1    1    0    1    0   3
Phalaenopsis-gene486036               1    0    0    1    0   2
Phalaenopsis-gene550132               1    0    1    0    0   2
Phalaenopsis-gene572972               1    0    1    1    0   3
Phalaenopsis-gene57423                0    0    1    1    0   2
Phalaenopsis-gene574373               1    1    1    1    1   5
Phalaenopsis-gene616018               1    0    0    1    0   2
Phalaenopsis-gene616314               1    0    0    1    0   2
Phalaenopsis-gene651154               0    0    1    0    0   1
Phalaenopsis-gene652190               1    1    1    1    1   5
Phalaenopsis-gene724512               1    0    1    1    0   3
Phalaenopsis-gene729562               0    0    1    1    0   2
Phalaenopsis-gene74697                1    0    1    0    0   2
Phalaenopsis-gene774361               1    0    0    1    0   2
Phalaenopsis-gene786424               1    1    1    1    1   5
Phalaenopsis-gene807101               1    1    1    1    1   5
Phalaenopsis-gene851758               1    0    0    1    0   2
Phalaenopsis-gene857610               1    0    1    1    1   4
Phalaenopsis-gene857807               1    1    1    1    1   5
Phalaenopsis-gene857823               1    0    1    1    0   3
Phalaenopsis-gene882600               1    1    1    1    1   5
Phalaenopsis-gene898913               1    0    1    1    0   3
Phalaenopsis-gene184632               0    0    0    1    0   1
Phalaenopsis-gene772101               1    0    1    1    0   3
Phalaenopsis-gene900923               1    0    0    0    0   1
Phalaenopsis-gene130334               1    0    0    1    0   2
Phalaenopsis-gene291370               1    1    1    1    1   5
Phalaenopsis-gene351263               1    0    1    1    1   4
Phalaenopsis-gene351336               1    0    1    0    0   2
Phalaenopsis-gene623387               1    1    1    1    1   5
Phalaenopsis-gene156004               1    0    1    1    0   3
Phalaenopsis-gene156060               1    1    0    1    0   3
Phalaenopsis-gene167980               1    1    1    1    1   5
Phalaenopsis-gene18714                1    1    1    1    1   5
Phalaenopsis-gene189020               0    0    1    1    0   2
Phalaenopsis-gene201792               0    0    0    1    0   1
Phalaenopsis-gene214664               1    1    0    1    0   3
Phalaenopsis-gene222259               1    0    1    1    0   3
Phalaenopsis-gene23018                0    0    1    1    0   2
Phalaenopsis-gene251239               0    0    0    1    0   1
Phalaenopsis-gene251249               1    1    1    1    1   5
Phalaenopsis-gene25419                1    0    1    0    0   2
Phalaenopsis-gene282141               0    1    1    1    0   3
Phalaenopsis-gene307233               1    0    0    0    0   1
Phalaenopsis-gene310227               1    1    1    1    1   5
Phalaenopsis-gene312175               1    1    1    1    1   5
Phalaenopsis-gene31862                0    0    0    0    0   0
Phalaenopsis-gene324449               0    0    1    1    0   2
Phalaenopsis-gene393788               1    0    1    1    0   3
Phalaenopsis-gene429911               1    0    0    1    0   2
Phalaenopsis-gene434196               0    0    0    0    0   0
Phalaenopsis-gene437331               0    0    1    1    0   2
Phalaenopsis-gene437727               0    0    1    0    0   1
Phalaenopsis-gene447228               1    0    1    1    0   3
Phalaenopsis-gene461965               1    0    1    1    0   3
Phalaenopsis-gene481105               1    0    1    1    0   3
Phalaenopsis-gene485707               1    0    1    1    0   3
Phalaenopsis-gene485708               0    0    1    1    0   2
Phalaenopsis-gene485955               1    0    1    1    0   3
Phalaenopsis-gene511873               1    0    1    1    0   3
Phalaenopsis-gene567804               0    0    0    1    1   2
Phalaenopsis-gene677330               1    0    1    0    0   2
Phalaenopsis-gene686742               1    1    1    1    1   5
Phalaenopsis-gene694174               1    1    1    1    1   5
Phalaenopsis-gene694175               0    0    0    1    0   1
Phalaenopsis-gene721125               1    1    1    1    1   5
Phalaenopsis-gene774307               1    0    1    1    0   3
Phalaenopsis-gene851820               1    0    0    1    0   2
Phalaenopsis-gene851876               1    0    0    1    0   2
Phalaenopsis-gene857631               1    1    1    1    1   5
Phalaenopsis-gene857647               1    0    1    0    0   2
Phalaenopsis-gene857691               0    0    1    1    0   2
Phalaenopsis-gene868262               1    0    1    1    0   3
Phalaenopsis-gene893490               1    0    0    1    0   2
Phalaenopsis-gene905672               1    1    1    1    1   5
Phalaenopsis-gene96807                0    0    0    1    0   1
Phalaenopsis-gene218936               1    0    1    1    0   3
Phalaenopsis-gene286487               0    0    0    1    0   1
Phalaenopsis-gene383906               1    1    1    1    1   5
Phalaenopsis-gene465693               1    1    1    1    1   5
Phalaenopsis-gene611900               1    0    0    1    0   2
Phalaenopsis-gene698936               0    1    0    1    0   2
Phalaenopsis-gene138717               1    1    1    1    1   5
Phalaenopsis-gene138752               1    1    1    1    1   5
Phalaenopsis-gene171942               0    0    1    0    0   1
Phalaenopsis-gene234928               1    1    1    1    1   5
Phalaenopsis-gene254682               1    0    0    0    0   1
Phalaenopsis-gene307482               1    1    1    0    0   3
Phalaenopsis-gene348692               1    0    1    1    0   3
Phalaenopsis-gene354955               1    1    1    1    1   5
Phalaenopsis-gene393761               1    0    1    1    0   3
Phalaenopsis-gene393855               1    0    1    0    0   2
Phalaenopsis-gene405063               0    0    1    1    0   2
Phalaenopsis-gene415078               1    0    1    0    0   2
Phalaenopsis-gene425538               1    0    0    1    0   2
Phalaenopsis-gene429891               1    1    1    1    1   5
Phalaenopsis-gene442764               1    0    1    1    0   3
Phalaenopsis-gene451677               0    1    1    0    0   2
Phalaenopsis-gene482601               1    1    1    1    1   5
Phalaenopsis-gene566028               1    1    1    1    1   5
Phalaenopsis-gene574428               1    0    0    1    0   2
Phalaenopsis-gene592634               1    0    0    1    0   2
Phalaenopsis-gene629705               1    1    0    0    0   2
Phalaenopsis-gene664851               1    0    1    0    0   2
Phalaenopsis-gene676912               1    0    1    0    0   2
Phalaenopsis-gene689777               1    1    1    1    1   5
Phalaenopsis-gene721178               1    0    1    0    0   2
Phalaenopsis-gene758297               0    0    1    0    0   1
Phalaenopsis-gene905236               1    0    0    0    0   1
Phalaenopsis-gene352053               1    1    1    1    1   5
Phalaenopsis-gene152976               1    1    1    1    1   5
Phalaenopsis-gene303433               1    0    1    1    0   3
Phalaenopsis-gene309604               1    1    0    1    0   3
Phalaenopsis-gene388642               0    0    1    1    0   2
Phalaenopsis-gene538075               1    0    0    1    0   2
Phalaenopsis-gene222489               1    0    1    0    0   2
Phalaenopsis-gene306754               1    0    1    1    0   3
Phalaenopsis-gene324609               1    0    0    1    1   3
Phalaenopsis-gene348656               1    0    1    1    1   4
Phalaenopsis-gene375284               1    0    1    1    0   3
Phalaenopsis-gene375520               1    0    1    1    0   3
Phalaenopsis-gene471022               1    1    1    1    1   5
Phalaenopsis-gene494236               1    1    1    1    1   5
Phalaenopsis-gene504360               1    0    0    0    0   1
Phalaenopsis-gene514044               1    1    1    1    1   5
Phalaenopsis-gene548684               1    0    1    1    0   3
Phalaenopsis-gene627447               1    1    1    1    1   5
Phalaenopsis-gene678671               1    0    1    0    0   2
Phalaenopsis-gene788735               1    1    1    1    1   5
Phalaenopsis-gene89413                0    0    1    0    0   1
Phalaenopsis-gene103250               1    0    0    1    1   3
Phalaenopsis-gene108047               0    0    1    1    0   2
Phalaenopsis-gene129591               1    1    1    1    1   5
Phalaenopsis-gene135531               1    1    1    1    1   5
Phalaenopsis-gene138425               1    1    1    1    1   5
Phalaenopsis-gene18227                1    0    1    0    0   2
Phalaenopsis-gene194764               1    0    1    1    0   3
Phalaenopsis-gene209174               0    0    1    0    0   1
Phalaenopsis-gene214753               1    0    0    0    0   1
Phalaenopsis-gene22122                0    0    0    1    0   1
Phalaenopsis-gene234967               1    1    1    1    1   5
Phalaenopsis-gene244798               1    1    1    1    1   5
Phalaenopsis-gene25337                1    0    1    1    0   3
Phalaenopsis-gene300878               1    0    0    1    0   2
Phalaenopsis-gene326915               0    0    1    0    0   1
Phalaenopsis-gene344824               1    1    1    1    1   5
Phalaenopsis-gene355126               0    0    1    0    0   1
Phalaenopsis-gene361650               1    0    0    1    0   2
Phalaenopsis-gene361768               1    0    0    0    0   1
Phalaenopsis-gene387344               1    0    0    1    0   2
Phalaenopsis-gene422003               1    1    1    1    1   5
Phalaenopsis-gene457327               0    0    1    1    0   2
Phalaenopsis-gene463509               1    1    1    1    0   4
Phalaenopsis-gene495343               0    1    1    1    1   4
Phalaenopsis-gene534065               1    0    1    1    0   3
Phalaenopsis-gene543773               1    0    1    1    0   3
Phalaenopsis-gene547236               1    1    1    1    1   5
Phalaenopsis-gene565255               1    1    1    1    1   5
Phalaenopsis-gene593009               1    1    1    1    1   5
Phalaenopsis-gene59340                1    1    1    1    1   5
Phalaenopsis-gene594160               1    0    0    1    0   2
Phalaenopsis-gene60770                1    1    1    1    1   5
Phalaenopsis-gene611686               1    0    1    1    0   3
Phalaenopsis-gene614510               0    0    1    1    0   2
Phalaenopsis-gene621629               1    1    1    1    1   5
Phalaenopsis-gene651097               1    1    1    1    1   5
Phalaenopsis-gene664926               1    1    1    1    1   5
Phalaenopsis-gene693081               1    0    0    1    0   2
Phalaenopsis-gene698619               0    0    1    0    0   1
Phalaenopsis-gene774867               1    0    0    0    0   1
Phalaenopsis-gene780767               0    0    1    0    0   1
Phalaenopsis-gene784867               1    0    0    0    0   1
Phalaenopsis-gene792421               1    0    0    1    0   2
Phalaenopsis-gene818101               1    1    1    1    1   5
Phalaenopsis-gene135764               0    1    0    1    0   2
Phalaenopsis-gene145885               1    1    1    1    1   5
Phalaenopsis-gene178889               1    1    1    1    1   5
Phalaenopsis-gene209212               1    0    0    0    0   1
Phalaenopsis-gene252139               1    0    1    1    0   3
Phalaenopsis-gene274108               1    1    1    1    1   5
Phalaenopsis-gene332133               0    0    1    0    0   1
Phalaenopsis-gene349315               1    1    1    1    1   5
Phalaenopsis-gene425248               1    1    1    1    1   5
Phalaenopsis-gene444150               1    0    0    1    0   2
Phalaenopsis-gene490317               1    0    1    1    0   3
Phalaenopsis-gene513695               1    0    1    1    0   3
Phalaenopsis-gene549110               0    0    1    1    0   2
Phalaenopsis-gene65133                1    0    0    1    0   2
Phalaenopsis-gene698588               1    0    1    1    0   3
Phalaenopsis-gene735829               1    0    1    1    0   3
Phalaenopsis-gene774866               1    1    1    1    1   5
Phalaenopsis-gene831076               0    0    0    1    0   1
Phalaenopsis-gene143583               1    0    0    1    0   2
Phalaenopsis-gene367937               1    0    1    1    0   3
Phalaenopsis-gene506687               1    0    1    1    0   3
Phalaenopsis-gene685581               1    1    1    1    1   5
Phalaenopsis-gene722676               1    0    1    0    0   2
Phalaenopsis-gene31884                1    0    0    1    0   2
Phalaenopsis-gene491999               1    0    1    0    0   2
Phalaenopsis-gene499833               1    1    1    1    1   5
Phalaenopsis-gene526696               1    1    1    1    1   5
Phalaenopsis-gene22445                1    1    1    1    1   5
Phalaenopsis-gene431521               1    1    0    1    0   3
Phalaenopsis-gene478126               1    0    1    1    0   3
Phalaenopsis-gene528185               1    1    1    1    1   5
Phalaenopsis-gene626057               1    0    1    0    1   3
Phalaenopsis-gene290154               1    0    1    1    0   3
Phalaenopsis-gene302341               0    0    1    0    0   1
Phalaenopsis-gene322283               1    0    1    1    0   3
Phalaenopsis-gene322325               1    1    1    1    1   5
Phalaenopsis-gene370858               1    0    0    1    0   2
Phalaenopsis-gene370925               1    0    1    0    0   2
Phalaenopsis-gene454520               1    1    1    1    1   5
Phalaenopsis-gene465776               1    1    1    0    0   3
Phalaenopsis-gene557759               1    1    1    1    1   5
Phalaenopsis-gene640996               0    0    1    1    0   2
Phalaenopsis-gene65156                1    0    0    0    0   1
Phalaenopsis-gene78465                1    1    1    1    1   5
Phalaenopsis-gene819554               1    1    1    1    1   5
Phalaenopsis-gene133120               0    1    0    1    0   2
Phalaenopsis-gene133157               1    0    0    1    0   2
Phalaenopsis-gene374397               1    1    1    1    1   5
Phalaenopsis-gene875008               1    0    0    1    0   2
Phalaenopsis-gene888967               1    1    1    1    1   5
Phalaenopsis-gene145222               1    0    1    1    0   3
Phalaenopsis-gene156069               0    0    1    0    0   1
Phalaenopsis-gene184665               0    1    0    0    0   1
Phalaenopsis-gene199475               1    0    0    1    0   2
Phalaenopsis-gene213347               0    0    1    1    0   2
Phalaenopsis-gene274161               0    0    1    1    0   2
Phalaenopsis-gene289302               1    0    0    0    0   1
Phalaenopsis-gene332102               0    0    1    1    0   2
Phalaenopsis-gene549249               1    1    1    1    1   5
Phalaenopsis-gene849364               1    0    0    1    0   2
Phalaenopsis-gene209610               1    0    1    1    0   3
Phalaenopsis-gene21086                1    0    1    1    0   3
Phalaenopsis-gene218947               1    0    1    1    0   3
Phalaenopsis-gene231803               1    1    1    1    1   5
Phalaenopsis-gene28416                1    1    1    1    1   5
Phalaenopsis-gene309678               1    0    0    0    1   2
Phalaenopsis-gene317825               1    0    1    1    0   3
Phalaenopsis-gene350545               1    0    1    1    0   3
Phalaenopsis-gene350663               1    0    0    1    0   2
Phalaenopsis-gene38809                0    0    1    0    0   1
Phalaenopsis-gene517953               1    1    1    1    1   5
Phalaenopsis-gene565351               0    1    1    1    0   3
Phalaenopsis-gene630983               1    1    0    1    0   3
Phalaenopsis-gene642195               1    0    1    0    0   2
Phalaenopsis-gene647298               1    1    1    1    1   5
Phalaenopsis-gene668337               1    0    1    1    0   3
Phalaenopsis-gene668364               1    0    0    1    0   2
Phalaenopsis-gene678709               0    0    0    1    0   1
Phalaenopsis-gene678738               0    0    0    0    0   0
Phalaenopsis-gene686980               1    0    1    1    0   3
Phalaenopsis-gene96095                0    0    1    0    0   1
Phalaenopsis-gene184558               0    0    0    1    0   1
Phalaenopsis-gene259967               1    1    1    1    1   5
Phalaenopsis-gene266949               1    0    0    0    0   1
Phalaenopsis-gene322870               1    1    1    1    1   5
Phalaenopsis-gene36942                1    1    1    1    1   5
Phalaenopsis-gene443869               1    0    1    1    0   3
Phalaenopsis-gene446762               1    0    0    0    0   1
Phalaenopsis-gene455608               1    0    1    0    0   2
Phalaenopsis-gene501632               1    1    1    1    1   5
Phalaenopsis-gene558245               1    0    1    0    0   2
Phalaenopsis-gene734996               1    0    1    1    0   3
Phalaenopsis-gene207674               1    1    1    1    1   5
Phalaenopsis-gene21031                1    0    1    0    0   2
Phalaenopsis-gene328849               0    0    0    1    0   1
Phalaenopsis-gene445890               1    1    1    1    0   4
Phalaenopsis-gene47713                1    1    1    1    1   5
Phalaenopsis-gene177262               1    1    0    1    0   3
Phalaenopsis-gene197963               1    1    1    1    1   5
Phalaenopsis-gene551733               1    0    1    0    0   2
Phalaenopsis-gene576472               1    0    1    1    0   3
Phalaenopsis-gene44215                1    1    1    1    1   5
Phalaenopsis-gene557683               0    0    1    0    0   1
Phalaenopsis-gene628696               1    1    1    1    0   4
Phalaenopsis-gene374232               1    1    1    1    1   5
Phalaenopsis-gene560940               1    0    0    1    0   2
Phalaenopsis-gene892890               1    1    1    1    1   5
Phalaenopsis-gene150165               1    1    1    1    1   5
Phalaenopsis-gene501672               1    0    1    0    0   2
Phalaenopsis-gene614348               0    0    0    1    0   1
Phalaenopsis-gene664806               1    0    1    1    0   3
Phalaenopsis-gene664843               1    0    0    1    0   2
Phalaenopsis-gene685631               0    0    1    1    0   2
Phalaenopsis-gene708130               1    0    1    1    0   3
Phalaenopsis-gene774236               1    0    1    0    0   2
Phalaenopsis-gene832738               1    1    1    1    1   5
Phalaenopsis-gene317543               1    1    0    0    0   2
Phalaenopsis-gene379196               1    1    1    1    1   5
Phalaenopsis-gene735751               1    0    1    0    0   2
Phalaenopsis-gene374335               1    1    1    1    1   5
Phalaenopsis-gene439952               1    0    1    0    0   2
Phalaenopsis-gene502209               0    0    0    1    0   1
Phalaenopsis-gene700251               1    1    1    1    1   5
Phalaenopsis-gene700404               1    0    0    1    0   2
Phalaenopsis-gene792506               1    0    0    0    0   1
Phalaenopsis-gene890982               1    0    0    1    0   2
Phalaenopsis-gene260034               1    0    0    1    0   2
Phalaenopsis-gene316594               1    0    1    1    0   3
Phalaenopsis-gene351437               1    0    0    0    0   1
Phalaenopsis-gene456682               0    0    0    1    0   1
Phalaenopsis-gene51508                1    0    1    1    0   3
Phalaenopsis-gene614423               1    0    0    0    0   1
Phalaenopsis-gene616318               0    0    1    0    0   1
Phalaenopsis-gene735599               1    1    1    1    1   5
Phalaenopsis-gene189070               1    0    1    1    0   3
Phalaenopsis-gene267055               1    0    0    1    0   2
Phalaenopsis-gene274267               1    0    0    1    0   2
Phalaenopsis-gene31307                1    1    1    1    0   4
Phalaenopsis-gene319795               1    1    1    1    1   5
Phalaenopsis-gene355371               0    0    0    0    0   0
Phalaenopsis-gene432904               1    0    0    0    0   1
Phalaenopsis-gene592774               0    1    0    1    0   2
Phalaenopsis-gene59499                1    1    1    1    1   5
Phalaenopsis-gene623420               1    0    1    1    0   3
Phalaenopsis-gene649337               0    0    1    1    0   2
Phalaenopsis-gene715124               1    1    1    1    1   5
Phalaenopsis-gene785914               1    0    0    1    0   2
Phalaenopsis-gene792605               0    0    1    1    0   2
Phalaenopsis-gene321469               1    1    1    1    1   5
Phalaenopsis-gene429899               1    1    1    1    1   5
Phalaenopsis-gene471941               1    0    1    1    0   3
Phalaenopsis-gene471983               1    0    1    0    0   2
Phalaenopsis-gene487960               1    1    1    1    0   4
Phalaenopsis-gene636687               1    0    1    0    1   3
Phalaenopsis-gene140339               1    0    1    0    0   2
Phalaenopsis-gene426570               1    1    1    1    1   5
Phalaenopsis-gene448076               1    1    1    1    1   5
Phalaenopsis-gene644772               0    0    0    1    0   1
Phalaenopsis-gene228739               1    1    1    1    1   5
Phalaenopsis-gene475979               1    0    0    1    0   2
Phalaenopsis-gene550978               1    1    1    1    1   5
Phalaenopsis-gene110707               0    0    1    1    0   2
Phalaenopsis-gene116772               0    0    1    1    0   2
Phalaenopsis-gene129538               1    0    1    1    0   3
Phalaenopsis-gene130582               1    0    1    0    0   2
Phalaenopsis-gene137223               1    0    1    0    0   2
Phalaenopsis-gene138437               1    0    0    1    0   2
Phalaenopsis-gene138859               1    0    1    1    0   3
Phalaenopsis-gene148785               0    0    0    1    0   1
Phalaenopsis-gene148797               1    1    0    1    0   3
Phalaenopsis-gene160585               1    1    1    1    0   4
Phalaenopsis-gene163421               0    0    1    1    0   2
Phalaenopsis-gene163422               1    0    1    1    0   3
Phalaenopsis-gene167981               1    1    1    1    1   5
Phalaenopsis-gene168008               1    0    0    1    0   2
Phalaenopsis-gene171945               1    0    1    1    0   3
Phalaenopsis-gene172177               0    1    1    1    0   3
Phalaenopsis-gene172213               1    0    1    1    0   3
Phalaenopsis-gene18221                1    1    1    1    1   5
Phalaenopsis-gene183199               0    1    1    1    0   3
Phalaenopsis-gene194629               1    1    1    1    1   5
Phalaenopsis-gene201113               1    0    1    0    0   2
Phalaenopsis-gene202324               1    0    0    1    0   2
Phalaenopsis-gene209250               1    1    1    1    1   5
Phalaenopsis-gene209443               1    1    1    1    1   5
Phalaenopsis-gene209444               1    0    1    1    0   3
Phalaenopsis-gene209611               0    0    1    1    0   2
Phalaenopsis-gene211554               1    0    0    0    0   1
Phalaenopsis-gene214708               1    0    1    1    0   3
Phalaenopsis-gene217975               1    1    1    1    1   5
Phalaenopsis-gene219131               1    1    1    1    1   5
Phalaenopsis-gene222061               1    1    1    1    1   5
Phalaenopsis-gene228735               1    1    1    1    1   5
Phalaenopsis-gene23017                1    1    1    1    1   5
Phalaenopsis-gene244712               1    0    0    1    0   2
Phalaenopsis-gene244738               1    0    0    1    0   2
Phalaenopsis-gene247184               1    1    0    0    0   2
Phalaenopsis-gene250751               1    1    0    0    0   2
Phalaenopsis-gene251209               1    0    1    0    0   2
Phalaenopsis-gene251220               1    0    1    1    0   3
Phalaenopsis-gene253790               1    1    1    1    1   5
Phalaenopsis-gene253799               1    1    1    1    1   5
Phalaenopsis-gene254642               1    1    1    1    1   5
Phalaenopsis-gene254643               1    0    1    1    0   3
Phalaenopsis-gene254656               1    0    1    1    0   3
Phalaenopsis-gene254668               1    0    1    1    0   3
Phalaenopsis-gene256093               1    0    1    1    0   3
Phalaenopsis-gene266945               1    0    1    1    0   3
Phalaenopsis-gene269458               1    0    0    1    0   2
Phalaenopsis-gene27342                0    0    0    1    0   1
Phalaenopsis-gene279611               0    0    0    1    0   1
Phalaenopsis-gene281449               0    0    1    1    0   2
Phalaenopsis-gene281469               1    1    1    1    1   5
Phalaenopsis-gene281613               1    1    1    1    1   5
Phalaenopsis-gene28485                1    0    1    1    0   3
Phalaenopsis-gene28554                1    0    0    1    0   2
Phalaenopsis-gene28647                1    0    0    1    0   2
Phalaenopsis-gene286558               1    1    1    1    1   5
Phalaenopsis-gene297294               1    1    1    1    1   5
Phalaenopsis-gene300855               1    0    0    1    0   2
Phalaenopsis-gene300871               1    0    0    1    0   2
Phalaenopsis-gene303210               1    0    1    1    0   3
Phalaenopsis-gene306753               1    1    1    1    1   5
Phalaenopsis-gene308831               1    0    1    1    0   3
Phalaenopsis-gene309948               1    0    1    1    0   3
Phalaenopsis-gene310133               1    0    1    1    1   4
Phalaenopsis-gene312174               1    1    1    1    0   4
Phalaenopsis-gene312176               1    1    1    1    1   5
Phalaenopsis-gene31306                1    0    0    1    0   2
Phalaenopsis-gene320066               1    1    1    1    1   5
Phalaenopsis-gene320149               0    0    0    1    0   1
Phalaenopsis-gene321470               0    0    0    0    0   0
Phalaenopsis-gene322843               1    0    0    1    0   2
Phalaenopsis-gene322917               1    0    1    1    0   3
Phalaenopsis-gene324325               1    1    1    1    1   5
Phalaenopsis-gene324445               1    1    1    1    1   5
Phalaenopsis-gene324446               1    0    0    1    0   2
Phalaenopsis-gene324551               0    1    1    1    0   3
Phalaenopsis-gene324552               0    1    1    1    0   3
Phalaenopsis-gene324578               1    1    1    1    1   5
Phalaenopsis-gene324585               1    1    1    1    1   5
Phalaenopsis-gene325341               1    1    1    1    1   5
Phalaenopsis-gene331277               1    1    1    1    1   5
Phalaenopsis-gene334824               1    1    1    1    1   5
Phalaenopsis-gene335939               1    0    0    1    0   2
Phalaenopsis-gene337738               1    1    1    1    0   4
Phalaenopsis-gene341637               0    0    1    1    0   2
Phalaenopsis-gene341638               1    1    1    1    1   5
Phalaenopsis-gene343528               0    0    0    0    0   0
Phalaenopsis-gene343529               1    0    1    1    0   3
Phalaenopsis-gene343830               1    1    1    1    1   5
Phalaenopsis-gene343868               1    0    1    0    0   2
Phalaenopsis-gene345040               1    1    1    1    1   5
Phalaenopsis-gene345059               1    0    1    0    0   2
Phalaenopsis-gene34977                1    1    1    1    1   5
Phalaenopsis-gene353042               1    1    1    1    1   5
Phalaenopsis-gene361603               1    1    0    0    0   2
Phalaenopsis-gene364347               1    1    1    1    1   5
Phalaenopsis-gene36570                1    0    1    0    0   2
Phalaenopsis-gene370901               1    1    1    1    1   5
Phalaenopsis-gene370973               1    0    1    1    0   3
Phalaenopsis-gene372200               1    1    1    1    1   5
Phalaenopsis-gene372203               1    1    1    1    1   5
Phalaenopsis-gene373558               1    1    1    1    1   5
Phalaenopsis-gene375290               1    1    1    0    0   3
Phalaenopsis-gene375526               0    0    1    1    0   2
Phalaenopsis-gene384306               1    0    1    1    0   3
Phalaenopsis-gene384555               1    0    1    0    0   2
Phalaenopsis-gene391035               1    1    1    1    1   5
Phalaenopsis-gene403308               1    0    0    1    0   2
Phalaenopsis-gene412713               1    0    0    1    0   2
Phalaenopsis-gene412743               1    0    0    1    0   2
Phalaenopsis-gene413458               0    0    1    0    0   1
Phalaenopsis-gene413492               1    0    1    0    0   2
Phalaenopsis-gene419332               1    0    1    0    0   2
Phalaenopsis-gene429824               0    0    0    1    0   1
Phalaenopsis-gene429825               0    0    0    0    0   0
Phalaenopsis-gene434217               1    0    0    1    0   2
Phalaenopsis-gene43492                1    1    1    1    1   5
Phalaenopsis-gene437724               1    1    1    1    1   5
Phalaenopsis-gene438619               1    0    1    0    0   2
Phalaenopsis-gene442985               1    1    0    1    0   3
Phalaenopsis-gene442986               1    1    1    1    1   5
Phalaenopsis-gene44304                1    1    1    1    1   5
Phalaenopsis-gene443159               1    1    1    1    1   5
Phalaenopsis-gene448078               1    1    1    1    1   5
Phalaenopsis-gene451705               1    0    0    1    0   2
Phalaenopsis-gene455760               1    1    1    1    1   5
Phalaenopsis-gene456249               1    0    1    1    0   3
Phalaenopsis-gene459233               1    1    1    1    1   5
Phalaenopsis-gene461825               1    0    1    1    0   3
Phalaenopsis-gene461861               0    0    1    1    0   2
Ophrys-gene9118                       1    1    1    1    1   5
Ophrys-gene21039                      1    0    1    0    0   2
Phalaenopsis-gene47562                1    1    1    1    1   5
Phalaenopsis-gene482166               1    1    1    1    1   5
Phalaenopsis-gene485456               0    0    1    1    0   2
Phalaenopsis-gene485620               1    0    1    0    0   2
Phalaenopsis-gene485954               0    1    1    1    0   3
Phalaenopsis-gene486281               1    0    0    0    0   1
Phalaenopsis-gene488013               1    0    0    0    0   1
Phalaenopsis-gene490789               1    0    0    0    0   1
Phalaenopsis-gene502900               1    1    1    1    0   4
Phalaenopsis-gene51349                1    0    1    0    0   2
Phalaenopsis-gene514729               1    0    0    0    0   1
Phalaenopsis-gene514733               1    0    1    1    0   3
Phalaenopsis-gene516161               1    0    1    1    0   3
Phalaenopsis-gene516162               1    1    1    1    0   4
Phalaenopsis-gene519172               1    0    0    0    0   1
Phalaenopsis-gene523436               0    0    0    1    0   1
Phalaenopsis-gene523487               1    0    0    1    0   2
Phalaenopsis-gene526659               1    0    1    1    0   3
Phalaenopsis-gene531067               1    0    0    1    0   2
Phalaenopsis-gene531072               1    0    1    0    0   2
Phalaenopsis-gene533980               1    0    1    1    0   3
Phalaenopsis-gene538062               1    1    1    1    1   5
Phalaenopsis-gene547701               1    1    1    1    1   5
Phalaenopsis-gene547841               1    0    1    1    0   3
Phalaenopsis-gene548135               1    0    1    1    0   3
Phalaenopsis-gene548162               1    1    0    1    0   3
Phalaenopsis-gene548233               1    0    1    1    0   3
Phalaenopsis-gene548274               1    0    0    1    0   2
Phalaenopsis-gene551715               1    1    1    1    1   5
Phalaenopsis-gene565270               1    1    1    1    1   5
Phalaenopsis-gene568301               1    0    1    0    0   2
Phalaenopsis-gene570202               1    1    1    1    1   5
Phalaenopsis-gene572955               1    1    0    0    0   2
Phalaenopsis-gene574265               1    1    1    1    1   5
Phalaenopsis-gene574461               0    0    1    1    0   2
Phalaenopsis-gene578788               1    0    0    1    0   2
Phalaenopsis-gene579133               1    1    1    1    1   5
Phalaenopsis-gene579134               1    0    0    1    0   2
Phalaenopsis-gene579482               0    0    1    1    0   2
Phalaenopsis-gene579485               0    1    1    0    0   2
Phalaenopsis-gene579492               1    1    1    1    1   5
Phalaenopsis-gene588959               1    0    0    1    0   2
Phalaenopsis-gene590531               1    1    1    1    1   5
Phalaenopsis-gene592207               1    0    0    0    0   1
Phalaenopsis-gene592208               1    1    1    1    1   5
Phalaenopsis-gene592249               1    0    0    1    0   2
Phalaenopsis-gene592871               1    1    1    1    1   5
Phalaenopsis-gene593320               0    0    0    1    1   2
Phalaenopsis-gene59498                1    1    1    1    1   5
Phalaenopsis-gene61113                0    0    1    1    1   3
Phalaenopsis-gene615885               1    0    1    1    0   3
Phalaenopsis-gene621634               1    1    1    1    1   5
Phalaenopsis-gene629706               1    0    1    1    0   3
Phalaenopsis-gene630178               0    0    1    0    0   1
Phalaenopsis-gene640990               1    0    0    1    0   2
Phalaenopsis-gene644310               1    1    1    1    1   5
Phalaenopsis-gene649384               1    0    1    0    0   2
Phalaenopsis-gene650823               1    1    1    1    1   5
Phalaenopsis-gene651040               1    1    1    1    1   5
Phalaenopsis-gene662848               1    0    1    1    0   3
Phalaenopsis-gene66320                0    0    0    1    0   1
Phalaenopsis-gene664802               1    1    1    1    1   5
Phalaenopsis-gene664839               1    0    1    0    0   2
Phalaenopsis-gene664850               1    0    1    1    0   3
Phalaenopsis-gene667417               1    1    1    1    1   5
Phalaenopsis-gene668704               1    0    1    0    0   2
Phalaenopsis-gene668717               0    0    0    1    0   1
Phalaenopsis-gene670054               1    1    1    1    1   5
Phalaenopsis-gene676913               0    0    0    1    0   1
Phalaenopsis-gene677328               1    1    1    1    1   5
Phalaenopsis-gene681589               1    1    0    0    0   2
Phalaenopsis-gene686979               1    1    1    1    1   5
Phalaenopsis-gene692028               1    1    1    1    1   5
Phalaenopsis-gene709614               1    1    1    1    1   5
Phalaenopsis-gene714301               1    0    1    1    0   3
Phalaenopsis-gene716032               0    0    1    1    0   2
Phalaenopsis-gene724511               0    0    1    0    0   1
Phalaenopsis-gene724614               1    1    1    1    1   5
Phalaenopsis-gene725110               1    1    1    1    1   5
Phalaenopsis-gene731510               1    1    1    1    1   5
Phalaenopsis-gene733037               1    0    0    0    0   1
Phalaenopsis-gene733038               0    0    0    0    0   0
Phalaenopsis-gene734958               1    0    1    0    0   2
Phalaenopsis-gene734985               0    0    0    0    0   0
Phalaenopsis-gene741167               1    1    1    1    1   5
Phalaenopsis-gene741695               1    0    0    1    0   2
Phalaenopsis-gene745941               1    1    1    1    1   5
Phalaenopsis-gene74698                0    0    1    1    0   2
Phalaenopsis-gene747886               1    1    1    1    1   5
Phalaenopsis-gene750434               1    1    1    1    1   5
Phalaenopsis-gene751366               1    0    0    0    0   1
Phalaenopsis-gene754656               1    1    1    0    1   4
Phalaenopsis-gene754659               1    1    1    1    1   5
Phalaenopsis-gene755042               1    1    1    1    1   5
Phalaenopsis-gene75769                1    1    1    1    1   5
Phalaenopsis-gene758338               1    0    1    0    0   2
Phalaenopsis-gene773922               1    0    1    1    0   3
Phalaenopsis-gene774319               1    1    1    1    1   5
Phalaenopsis-gene774373               0    0    1    1    0   2
Phalaenopsis-gene778949               1    0    0    0    0   1
Phalaenopsis-gene784198               1    1    1    1    1   5
Phalaenopsis-gene786147               1    0    1    1    0   3
Phalaenopsis-gene786152               1    0    1    1    0   3
Phalaenopsis-gene788736               1    0    0    0    0   1
Phalaenopsis-gene789565               1    1    1    1    1   5
Phalaenopsis-gene789592               1    1    1    1    1   5
Phalaenopsis-gene794274               1    0    1    0    0   2
Phalaenopsis-gene806421               0    0    1    1    0   2
Phalaenopsis-gene807102               1    1    1    1    1   5
Phalaenopsis-gene807119               1    1    1    1    1   5
Phalaenopsis-gene814278               1    0    0    0    0   1
Phalaenopsis-gene814285               1    0    1    1    0   3
Phalaenopsis-gene814602               1    0    1    0    0   2
Phalaenopsis-gene819675               1    0    0    1    0   2
Phalaenopsis-gene822785               1    0    0    1    0   2
Phalaenopsis-gene830987               1    0    1    1    0   3
Phalaenopsis-gene835964               1    1    1    1    1   5
Phalaenopsis-gene84227                1    1    1    1    1   5
Phalaenopsis-gene846674               1    0    0    1    0   2
Phalaenopsis-gene84826                1    1    1    1    1   5
Phalaenopsis-gene85147                1    0    1    0    0   2
Phalaenopsis-gene851759               1    0    1    0    0   2
Phalaenopsis-gene851760               1    0    0    1    0   2
Phalaenopsis-gene851793               1    0    0    0    0   1
Phalaenopsis-gene851821               1    1    1    1    1   5
Phalaenopsis-gene851822               0    0    0    1    0   1
Phalaenopsis-gene851851               0    0    1    1    0   2
Phalaenopsis-gene852618               1    1    1    1    1   5
Phalaenopsis-gene854738               1    1    1    1    1   5
Phalaenopsis-gene855374               1    0    0    0    0   1
Phalaenopsis-gene855393               1    0    1    1    0   3
Phalaenopsis-gene855412               1    1    1    1    1   5
Phalaenopsis-gene857611               0    0    1    1    0   2
Phalaenopsis-gene857707               1    0    1    0    0   2
Phalaenopsis-gene859316               1    0    1    1    0   3
Phalaenopsis-gene859401               1    1    1    1    1   5
Phalaenopsis-gene865840               1    0    1    1    0   3
Phalaenopsis-gene874703               1    1    1    1    1   5
Phalaenopsis-gene876534               1    0    0    1    0   2
Phalaenopsis-gene876700               1    0    1    1    0   3
Phalaenopsis-gene888202               1    1    1    1    1   5
Phalaenopsis-gene888213               1    1    1    1    1   5
Phalaenopsis-gene888225               1    0    1    0    0   2
Phalaenopsis-gene888255               1    0    1    0    0   2
Phalaenopsis-gene888309               1    1    1    1    1   5
Phalaenopsis-gene892997               1    0    0    0    0   1
Phalaenopsis-gene89313                1    1    1    1    1   5
Phalaenopsis-gene896650               1    1    1    1    1   5
Phalaenopsis-gene897241               1    0    0    1    1   3
Phalaenopsis-gene90143                0    0    0    1    0   1
Phalaenopsis-gene90386                1    0    1    0    0   2
Phalaenopsis-gene904226               1    1    1    1    1   5
Phalaenopsis-gene9355                 1    0    1    1    0   3
Phalaenopsis-gene96303                1    1    1    1    1   5
Phalaenopsis-gene98824                1    1    1    1    1   5
Phalaenopsis-gene99440                1    1    1    1    1   5
Ophrys-gene6942                       1    1    1    1    1   5
Ophrys-gene114917                     1    1    1    1    1   5
Ophrys-gene137824                     1    0    0    0    0   1
Ophrys-gene436006                     0    1    0    1    0   2
Ophrys-gene366                        1    1    1    1    1   5
Ophrys-gene2422                       1    0    1    1    0   3
Ophrys-gene4886                       0    1    0    1    0   2
Ophrys-gene6012                       1    1    0    1    0   3
Ophrys-gene7897                       1    1    1    1    1   5
Ophrys-gene8333                       1    0    0    0    0   1
Ophrys-gene9298                       1    0    0    1    0   2
Ophrys-gene21337                      0    0    1    1    0   2
Ophrys-gene49955                      1    0    1    0    0   2
Ophrys-gene50243                      1    1    1    1    1   5
Ophrys-gene77911                      1    1    0    1    0   3
Ophrys-gene141551                     1    1    1    1    1   5
Ophrys-gene141905                     1    1    1    1    1   5
Ophrys-gene143303                     1    0    0    1    0   2
Ophrys-gene160808                     1    0    1    1    0   3
Ophrys-gene182806                     0    0    0    0    0   0
Ophrys-gene183873                     1    0    0    1    0   2
Ophrys-gene184360                     1    1    1    1    1   5
Ophrys-gene190754                     1    0    1    0    0   2
Ophrys-gene199062                     1    0    1    1    0   3
Ophrys-gene207289                     1    0    0    0    0   1
Ophrys-gene207970                     1    1    1    1    1   5
Ophrys-gene210660                     1    1    1    1    1   5
Ophrys-gene210785                     1    0    0    1    0   2
Ophrys-gene226847                     1    0    0    1    0   2
Ophrys-gene258565                     1    0    0    1    0   2
Ophrys-gene265307                     1    0    0    1    0   2
Ophrys-gene280654                     1    1    1    1    1   5
Ophrys-gene284387                     0    0    1    1    0   2
Ophrys-gene284596                     1    0    0    0    0   1
Ophrys-gene294675                     1    1    1    1    1   5
Ophrys-gene294943                     1    1    1    1    1   5
Ophrys-gene296692                     1    1    1    1    1   5
Ophrys-gene297028                     0    0    1    1    0   2
Ophrys-gene297192                     1    1    1    1    1   5
Ophrys-gene302239                     1    1    1    1    1   5
Ophrys-gene324715                     1    1    1    1    1   5
Ophrys-gene342461                     1    1    1    1    1   5
Ophrys-gene342489                     0    0    1    1    0   2
Ophrys-gene369337                     1    1    1    1    1   5
Ophrys-gene373285                     1    0    1    1    0   3
Ophrys-gene391087                     1    0    1    1    0   3
Ophrys-gene399441                     0    0    0    1    0   1
Ophrys-gene412826                     1    1    1    1    1   5
Ophrys-gene416885                     1    0    1    0    0   2
Ophrys-gene431786                     0    0    0    1    0   1
Ophrys-gene434830                     0    0    0    1    0   1
Ophrys-gene444886                     1    0    0    1    0   2
Ophrys-gene445198                     1    0    1    1    0   3
Ophrys-gene458142                     0    0    0    0    0   0
Ophrys-gene491439                     1    1    1    1    1   5
Ophrys-gene495932                     1    1    1    0    0   3
Ophrys-gene525780                     1    0    0    1    0   2
Ophrys-gene554227                     0    0    0    1    0   1
Ophrys-gene569472                     1    0    1    1    1   4
Ophrys-gene575720                     1    1    1    1    1   5
Ophrys-gene602871                     1    1    1    1    1   5
Ophrys-gene615043                     1    0    1    1    0   3
Ophrys-gene617144                     1    0    0    1    0   2
Ophrys-gene629892                     1    0    0    1    0   2
Ophrys-gene637235                     1    1    1    1    1   5
Ophrys-gene644544                     1    1    1    1    1   5
Ophrys-gene646077                     1    1    1    1    1   5
Ophrys-gene661644                     0    0    0    1    0   1
Ophrys-gene662234                     1    1    1    1    1   5
Ophrys-gene694840                     1    0    1    1    0   3
Ophrys-gene707987                     1    1    0    1    0   3
Ophrys-gene710459                     1    1    1    1    1   5
Ophrys-gene710959                     0    0    0    1    0   1
Ophrys-gene731424                     1    0    0    0    0   1
Ophrys-gene731823                     1    0    1    1    0   3
Ophrys-gene603356                     1    1    1    1    1   5
Ophrys-gene1181                       1    0    1    1    0   3
Ophrys-gene3118                       1    1    1    1    1   5
Ophrys-gene4698                       1    1    1    1    1   5
Ophrys-gene5266                       1    0    0    1    0   2
Ophrys-gene6097                       1    0    1    1    0   3
Ophrys-gene8160                       1    1    1    1    1   5
Ophrys-gene11517                      1    1    1    1    1   5
Ophrys-gene12232                      0    0    1    1    0   2
Ophrys-gene12371                      1    0    1    0    0   2
Ophrys-gene21472                      1    1    1    1    1   5
Ophrys-gene22661                      1    1    1    1    1   5
Ophrys-gene23070                      1    1    1    1    1   5
Ophrys-gene23222                      1    0    0    1    0   2
Ophrys-gene24469                      1    1    1    1    1   5
Ophrys-gene24973                      1    0    1    1    0   3
Ophrys-gene46814                      1    0    0    1    0   2
Ophrys-gene50721                      1    1    1    1    1   5
Ophrys-gene58601                      1    1    1    1    0   4
Ophrys-gene59583                      1    1    1    1    1   5
Ophrys-gene76842                      0    0    1    1    0   2
Ophrys-gene77910                      1    1    1    1    1   5
Ophrys-gene88438                      1    1    1    1    1   5
Ophrys-gene118637                     0    0    1    1    0   2
Ophrys-gene127051                     1    0    1    1    0   3
Ophrys-gene141543                     1    0    0    1    0   2
Ophrys-gene143304                     1    0    0    1    0   2
Ophrys-gene154927                     1    1    1    1    1   5
Ophrys-gene155849                     0    0    1    1    0   2
Ophrys-gene167688                     1    1    1    1    1   5
Ophrys-gene180054                     0    0    0    1    0   1
Ophrys-gene180216                     1    1    1    1    1   5
Ophrys-gene183904                     1    0    0    0    0   1
Ophrys-gene186787                     1    1    1    1    1   5
Ophrys-gene195127                     1    0    1    1    0   3
Ophrys-gene199095                     1    1    0    0    0   2
Ophrys-gene199468                     1    1    1    1    1   5
Ophrys-gene203710                     1    1    1    1    1   5
Ophrys-gene203779                     1    1    1    1    1   5
Ophrys-gene205142                     1    1    1    1    1   5
Ophrys-gene210664                     1    1    1    1    1   5
Ophrys-gene211126                     1    0    1    1    0   3
Ophrys-gene213724                     1    1    1    1    1   5
Ophrys-gene215380                     1    1    1    1    1   5
Ophrys-gene215608                     1    0    0    1    0   2
Ophrys-gene236047                     0    0    1    1    0   2
Ophrys-gene239099                     1    0    1    1    0   3
Ophrys-gene239548                     1    1    1    1    1   5
Ophrys-gene246903                     1    0    0    0    0   1
Ophrys-gene252088                     0    0    0    1    0   1
Ophrys-gene253004                     1    1    1    1    1   5
Ophrys-gene265609                     1    1    1    1    1   5
Ophrys-gene277388                     1    1    1    1    1   5
Ophrys-gene278352                     1    0    0    0    0   1
Ophrys-gene284625                     1    1    1    1    1   5
Ophrys-gene303748                     1    1    1    1    1   5
Ophrys-gene304231                     0    0    0    1    0   1
Ophrys-gene305022                     1    1    1    1    1   5
Ophrys-gene305998                     1    1    0    1    0   3
Ophrys-gene324686                     1    0    1    0    0   2
Ophrys-gene342456                     1    0    1    1    0   3
Ophrys-gene342460                     1    1    1    1    1   5
Ophrys-gene343312                     1    1    1    1    1   5
Ophrys-gene344526                     1    0    1    1    0   3
Ophrys-gene373320                     1    1    0    0    0   2
Ophrys-gene378148                     1    1    1    1    1   5
Ophrys-gene384539                     1    1    1    1    1   5
Ophrys-gene386407                     1    1    1    1    1   5
Ophrys-gene389367                     1    1    1    1    1   5
Ophrys-gene392656                     1    1    1    1    1   5
Ophrys-gene397456                     1    0    1    1    0   3
Ophrys-gene402532                     1    0    0    1    0   2
Ophrys-gene412210                     1    1    0    1    0   3
Ophrys-gene427713                     1    1    1    1    1   5
Ophrys-gene444577                     0    0    0    1    0   1
Ophrys-gene444738                     1    0    0    0    0   1
Ophrys-gene458157                     1    0    0    1    1   3
Ophrys-gene477483                     1    1    1    1    1   5
Ophrys-gene480796                     0    0    0    0    0   0
Ophrys-gene487268                     1    0    0    1    0   2
Ophrys-gene495237                     1    0    1    1    0   3
Ophrys-gene495300                     1    0    1    1    0   3
Ophrys-gene495432                     1    0    1    0    0   2
Ophrys-gene495556                     1    0    0    0    0   1
Ophrys-gene496294                     0    0    1    0    0   1
Ophrys-gene496517                     0    0    0    1    0   1
Ophrys-gene510328                     1    1    1    1    1   5
Ophrys-gene511155                     1    1    1    1    1   5
Ophrys-gene519090                     1    0    1    1    0   3
Ophrys-gene526216                     1    1    1    1    1   5
Ophrys-gene546824                     1    0    1    0    0   2
Ophrys-gene570069                     1    1    1    1    1   5
Ophrys-gene585658                     1    1    1    1    1   5
Ophrys-gene590711                     1    1    1    1    1   5
Ophrys-gene591108                     0    0    0    0    0   0
Ophrys-gene603353                     1    0    1    1    0   3
Ophrys-gene613536                     1    0    0    1    0   2
Ophrys-gene616682                     1    0    1    1    0   3
Ophrys-gene617145                     0    0    1    1    1   3
Ophrys-gene617306                     1    0    0    0    0   1
Ophrys-gene642631                     1    0    1    1    0   3
Ophrys-gene689885                     0    0    0    1    0   1
Ophrys-gene689917                     1    1    1    1    1   5
Ophrys-gene701017                     1    0    0    1    0   2
Ophrys-gene702667                     0    0    0    0    0   0
Ophrys-gene709769                     1    0    0    0    0   1
Ophrys-gene710458                     0    0    1    1    0   2
Ophrys-gene722356                     1    0    1    1    0   3
Ophrys-gene731576                     0    0    0    1    0   1
Ophrys-gene735365                     0    0    1    1    0   2
Ophrys-gene757502                     1    0    1    1    0   3
Ophrys-gene141593                     1    0    1    0    0   2
Ophrys-gene320746                     1    0    0    1    1   3
Ophrys-gene546851                     1    1    0    1    0   3
Ophrys-gene753296                     1    1    1    1    1   5
Ophrys-gene756280                     1    0    1    1    0   3
Ophrys-gene756310                     1    0    0    1    0   2
Ophrys-gene137683                     1    0    0    1    0   2
Ophrys-gene144475                     1    1    1    1    1   5
Ophrys-gene168529                     1    1    1    1    1   5
Ophrys-gene722564                     1    1    1    1    1   5
Ophrys-gene5027                       1    1    1    1    1   5
Ophrys-gene6266                       1    1    1    1    1   5
Ophrys-gene8974                       1    1    0    1    0   3
Ophrys-gene9778                       1    0    1    1    0   3
Ophrys-gene12231                      1    1    1    1    1   5
Ophrys-gene21544                      0    0    0    0    0   0
Ophrys-gene21676                      0    0    0    1    0   1
Ophrys-gene24537                      1    0    1    1    0   3
Ophrys-gene48001                      1    0    1    0    0   2
Ophrys-gene53545                      1    1    1    1    1   5
Ophrys-gene60412                      1    1    1    1    0   4
Ophrys-gene126355                     1    1    0    0    0   2
Ophrys-gene136712                     1    0    0    1    0   2
Ophrys-gene141883                     1    0    1    0    0   2
Ophrys-gene149148                     0    0    0    1    0   1
Ophrys-gene158430                     1    0    1    0    0   2
Ophrys-gene167042                     1    1    1    1    1   5
Ophrys-gene215607                     1    0    0    1    0   2
Ophrys-gene243834                     1    1    1    1    1   5
Ophrys-gene249759                     0    0    1    0    0   1
Ophrys-gene268088                     1    0    1    0    0   2
Ophrys-gene271081                     1    1    1    1    1   5
Ophrys-gene278517                     0    0    0    1    0   1
Ophrys-gene295466                     0    0    0    1    0   1
Ophrys-gene341059                     1    1    1    1    1   5
Ophrys-gene355303                     1    0    0    1    0   2
Ophrys-gene357610                     1    1    1    1    1   5
Ophrys-gene383160                     1    0    0    1    0   2
Ophrys-gene385878                     1    1    1    1    1   5
Ophrys-gene399452                     0    0    0    0    0   0
Ophrys-gene412211                     1    0    0    1    0   2
Ophrys-gene441857                     1    1    1    1    1   5
Ophrys-gene445294                     1    0    0    1    0   2
Ophrys-gene481275                     1    1    1    1    1   5
Ophrys-gene488617                     1    1    1    1    1   5
Ophrys-gene510602                     1    1    1    1    1   5
Ophrys-gene515104                     1    0    0    0    0   1
Ophrys-gene586882                     1    1    1    1    1   5
Ophrys-gene588951                     1    0    1    1    0   3
Ophrys-gene597731                     0    0    1    1    0   2
Ophrys-gene615026                     0    0    1    0    0   1
Ophrys-gene617298                     0    1    0    1    0   2
Ophrys-gene638847                     1    1    0    0    0   2
Ophrys-gene676955                     1    1    1    1    1   5
Ophrys-gene690490                     1    1    1    1    1   5
Ophrys-gene701031                     1    0    0    1    0   2
Ophrys-gene8941                       1    1    1    1    1   5
Ophrys-gene71691                      1    0    0    0    0   1
Ophrys-gene75339                      1    1    1    1    1   5
Ophrys-gene84210                      0    0    1    0    0   1
Ophrys-gene91049                      1    0    0    1    0   2
Ophrys-gene134802                     1    0    0    0    0   1
Ophrys-gene154929                     0    1    1    0    1   3
Ophrys-gene194067                     1    0    1    1    0   3
Ophrys-gene267852                     1    1    1    1    1   5
Ophrys-gene293131                     1    1    1    1    1   5
Ophrys-gene298095                     0    0    1    0    0   1
Ophrys-gene298971                     1    1    1    1    1   5
Ophrys-gene316518                     1    1    1    1    1   5
Ophrys-gene339112                     1    0    1    1    0   3
Ophrys-gene344508                     1    1    1    1    1   5
Ophrys-gene375123                     1    1    1    1    1   5
Ophrys-gene392861                     1    1    0    1    0   3
Ophrys-gene455367                     1    0    1    1    0   3
Ophrys-gene489505                     1    1    1    1    1   5
Ophrys-gene495662                     1    0    1    0    0   2
Ophrys-gene509225                     1    0    1    1    0   3
Ophrys-gene538310                     1    1    1    1    1   5
Ophrys-gene544270                     0    0    0    1    0   1
Ophrys-gene568680                     1    1    1    1    1   5
Ophrys-gene608284                     0    0    1    1    0   2
Ophrys-gene617358                     1    1    1    1    1   5
Ophrys-gene661551                     1    0    0    0    0   1
Ophrys-gene720208                     1    0    0    1    0   2
Ophrys-gene722408                     1    1    1    0    0   3
Ophrys-gene722452                     0    0    0    1    0   1
Ophrys-gene722598                     1    0    1    1    0   3
Ophrys-gene739652                     1    0    1    1    0   3
Ophrys-gene5532                       1    1    1    1    1   5
Ophrys-gene5599                       1    0    0    1    0   2
Ophrys-gene21353                      1    1    1    1    1   5
Ophrys-gene77253                      1    0    0    1    0   2
Ophrys-gene98993                      1    1    0    1    0   3
Ophrys-gene134881                     1    0    0    1    0   2
Ophrys-gene136053                     0    0    0    1    0   1
Ophrys-gene173970                     1    0    0    0    0   1
Ophrys-gene177775                     1    1    1    1    1   5
Ophrys-gene194066                     1    0    0    0    0   1
Ophrys-gene201108                     1    1    1    1    1   5
Ophrys-gene208033                     1    1    1    1    1   5
Ophrys-gene214157                     1    0    0    1    0   2
Ophrys-gene219244                     1    0    0    1    0   2
Ophrys-gene264787                     1    0    1    1    0   3
Ophrys-gene295664                     1    0    0    1    0   2
Ophrys-gene342493                     0    0    1    1    0   2
Ophrys-gene370048                     1    1    1    1    1   5
Ophrys-gene400019                     1    1    1    0    0   3
Ophrys-gene410496                     1    0    0    0    0   1
Ophrys-gene487189                     1    1    1    1    1   5
Ophrys-gene488614                     1    1    1    1    1   5
Ophrys-gene495487                     1    0    1    1    0   3
Ophrys-gene496579                     0    0    0    1    0   1
Ophrys-gene510589                     1    0    1    1    0   3
Ophrys-gene546876                     1    0    0    0    0   1
Ophrys-gene616638                     1    0    1    1    0   3
Ophrys-gene617219                     1    0    0    1    0   2
Ophrys-gene670540                     1    0    0    1    0   2
Ophrys-gene683717                     1    1    1    1    1   5
Ophrys-gene700983                     1    0    0    1    0   2
Ophrys-gene709763                     1    0    0    1    0   2
Ophrys-gene385869                     1    1    1    1    1   5
Ophrys-gene546763                     1    0    0    1    0   2
Ophrys-gene258374                     1    1    0    1    0   3
Ophrys-gene258435                     1    0    0    1    0   2
Ophrys-gene271576                     1    0    0    0    0   1
Ophrys-gene3730                       1    1    1    1    1   5
Ophrys-gene5281                       1    0    0    1    0   2
Ophrys-gene75724                      1    1    1    1    1   5
Ophrys-gene75771                      0    0    1    1    0   2
Ophrys-gene118874                     1    0    1    1    0   3
Ophrys-gene118878                     1    1    1    1    1   5
Ophrys-gene132960                     0    0    1    1    0   2
Ophrys-gene132962                     1    1    1    1    1   5
Ophrys-gene141884                     1    1    1    1    1   5
Ophrys-gene167036                     1    1    1    1    1   5
Ophrys-gene211081                     1    0    1    1    0   3
Ophrys-gene215356                     1    0    1    1    0   3
Ophrys-gene277970                     0    0    0    1    0   1
Ophrys-gene286706                     1    0    0    0    0   1
Ophrys-gene295787                     1    0    0    1    0   2
Ophrys-gene346409                     1    1    1    1    1   5
Ophrys-gene458081                     0    0    1    1    0   2
Ophrys-gene480785                     1    1    1    1    1   5
Ophrys-gene496547                     1    0    0    1    0   2
Ophrys-gene532000                     0    1    0    0    0   1
Ophrys-gene562311                     1    0    0    0    0   1
Ophrys-gene609850                     1    0    0    1    0   2
Ophrys-gene610392                     0    0    0    1    0   1
Ophrys-gene617136                     1    0    0    1    0   2
Ophrys-gene617202                     0    0    0    0    0   0
Ophrys-gene618155                     0    0    1    1    0   2
Ophrys-gene628366                     1    0    1    1    0   3
Ophrys-gene635297                     0    0    0    1    0   1
Ophrys-gene700217                     1    1    1    1    1   5
Ophrys-gene110805                     1    1    1    1    1   5
Ophrys-gene110790                     1    0    0    0    0   1
Ophrys-gene190759                     1    0    1    0    0   2
Ophrys-gene5244                       0    0    0    0    0   0
Ophrys-gene8643                       1    1    1    1    0   4
Ophrys-gene11098                      1    1    0    1    0   3
Ophrys-gene53508                      1    1    1    1    1   5
Ophrys-gene100632                     0    0    0    0    0   0
Ophrys-gene109089                     0    0    1    1    0   2
Ophrys-gene239210                     0    0    0    1    0   1
Ophrys-gene306249                     1    0    1    1    0   3
Ophrys-gene306286                     0    0    1    1    0   2
Ophrys-gene337461                     0    0    0    1    0   1
Ophrys-gene435313                     1    1    1    1    1   5
Ophrys-gene444121                     1    1    1    1    0   4
Ophrys-gene447642                     1    1    1    1    1   5
Ophrys-gene495664                     0    0    0    1    0   1
Ophrys-gene515341                     1    1    1    1    1   5
Ophrys-gene541302                     1    1    1    1    1   5
Ophrys-gene546795                     1    0    0    0    0   1
Ophrys-gene567004                     1    0    1    1    0   3
Ophrys-gene633363                     1    1    1    1    0   4
Ophrys-gene701000                     0    1    0    1    0   2
Ophrys-gene702663                     1    1    1    1    1   5
Ophrys-gene88401                      1    1    1    1    1   5
Ophrys-gene453176                     1    0    1    1    0   3
Ophrys-gene536838                     0    0    0    0    0   0
Ophrys-gene715260                     1    1    1    1    1   5
Ophrys-gene794                        1    0    0    1    0   2
Ophrys-gene4986                       1    1    1    1    1   5
Ophrys-gene6077                       1    0    1    0    0   2
Ophrys-gene21026                      1    0    0    1    0   2
Ophrys-gene117748                     1    0    1    0    0   2
Ophrys-gene133881                     1    0    1    1    0   3
Ophrys-gene136060                     1    0    1    0    0   2
Ophrys-gene173255                     1    1    1    1    1   5
Ophrys-gene210733                     1    1    1    1    1   5
Ophrys-gene271671                     1    1    0    1    0   3
Ophrys-gene271716                     0    0    0    1    0   1
Ophrys-gene293769                     1    1    1    1    1   5
Ophrys-gene313005                     1    0    1    1    0   3
Ophrys-gene357591                     1    0    0    1    0   2
Ophrys-gene374519                     1    0    0    1    0   2
Ophrys-gene375213                     1    1    1    1    1   5
Ophrys-gene378778                     0    0    1    1    0   2
Ophrys-gene390643                     1    1    1    1    1   5
Ophrys-gene441462                     0    0    0    0    0   0
Ophrys-gene444839                     1    0    1    0    0   2
Ophrys-gene445144                     0    0    1    1    0   2
Ophrys-gene484500                     1    1    1    1    1   5
Ophrys-gene582231                     1    1    1    1    1   5
Ophrys-gene595297                     1    1    1    1    1   5
Ophrys-gene637460                     1    0    0    0    0   1
Ophrys-gene658631                     1    1    1    1    1   5
Ophrys-gene695870                     1    1    1    1    1   5
Ophrys-gene715339                     1    0    0    1    0   2
Ophrys-gene727849                     1    0    0    1    0   2
Ophrys-gene69251                      1    0    1    1    0   3
Ophrys-gene231608                     1    0    1    1    0   3
Ophrys-gene231678                     0    0    1    1    0   2
Ophrys-gene414694                     1    0    0    1    0   2
Ophrys-gene282220                     1    1    1    1    0   4
Ophrys-gene436162                     1    0    0    1    0   2
Ophrys-gene3135                       1    0    0    1    0   2
Ophrys-gene238904                     1    1    1    1    1   5
Ophrys-gene268062                     1    0    0    1    0   2
Ophrys-gene291936                     1    0    0    1    0   2
Ophrys-gene306135                     0    0    1    1    0   2
Ophrys-gene441392                     1    1    1    1    1   5
Ophrys-gene683057                     1    1    1    1    1   5
Ophrys-gene8918                       1    1    1    1    1   5
Ophrys-gene113714                     1    1    1    1    1   5
Ophrys-gene122065                     1    1    0    1    0   3
Ophrys-gene137656                     1    1    1    1    1   5
Ophrys-gene211070                     1    0    0    1    0   2
Ophrys-gene236599                     1    1    1    1    1   5
Ophrys-gene291681                     1    1    1    1    1   5
Ophrys-gene361923                     1    1    1    1    1   5
Ophrys-gene436356                     1    0    0    0    0   1
Ophrys-gene462504                     1    1    1    1    1   5
Ophrys-gene538677                     0    0    1    1    0   2
Ophrys-gene609115                     1    1    1    1    1   5
Ophrys-gene632854                     1    0    0    1    0   2
Ophrys-gene661647                     1    0    0    1    0   2
Ophrys-gene670578                     1    0    0    0    0   1
Ophrys-gene670579                     0    0    0    1    0   1
Ophrys-gene672413                     1    0    0    0    0   1
Ophrys-gene695796                     1    0    0    0    0   1
Ophrys-gene740741                     1    1    1    1    1   5
Ophrys-gene5185                       1    1    1    1    1   5
Ophrys-gene45672                      0    0    0    1    0   1
Ophrys-gene144448                     1    1    1    1    1   5
Ophrys-gene164409                     0    0    1    1    0   2
Ophrys-gene170937                     1    0    1    1    0   3
Ophrys-gene271725                     0    0    0    0    0   0
Ophrys-gene282259                     1    0    0    1    0   2
Ophrys-gene295540                     0    0    1    1    0   2
Ophrys-gene338298                     1    1    1    1    1   5
Ophrys-gene399436                     1    1    1    1    1   5
Ophrys-gene412166                     1    1    1    1    1   5
Ophrys-gene425190                     0    0    0    1    0   1
Ophrys-gene564561                     1    1    1    1    1   5
Ophrys-gene570767                     1    0    0    0    0   1
Ophrys-gene615019                     0    0    0    0    0   0
Ophrys-gene618473                     1    0    0    0    1   2
Ophrys-gene632850                     0    0    1    0    0   1
Ophrys-gene696459                     1    0    0    1    0   2
Ophrys-gene298872                     0    0    1    0    0   1
Ophrys-gene258713                     0    0    0    1    0   1
Ophrys-gene92062                      1    0    0    1    0   2
Ophrys-gene249204                     1    1    1    1    1   5
Ophrys-gene298756                     1    1    1    1    1   5
Ophrys-gene459575                     1    1    1    1    1   5
Ophrys-gene487080                     1    0    0    1    0   2
Ophrys-gene525779                     1    0    1    1    0   3
Ophrys-gene647330                     1    1    1    1    1   5
Ophrys-gene701677                     1    1    1    1    1   5
Ophrys-gene8063                       1    0    0    1    0   2
Ophrys-gene133892                     1    1    1    1    1   5
Ophrys-gene536788                     1    0    0    1    0   2
Ophrys-gene574036                     1    1    1    1    1   5
Ophrys-gene1179                       1    0    1    0    0   2
Ophrys-gene50785                      1    1    1    1    1   5
Ophrys-gene50833                      1    1    1    1    1   5
Ophrys-gene137378                     1    1    1    1    1   5
Ophrys-gene203797                     0    1    0    1    0   2
Ophrys-gene249115                     1    0    0    1    0   2
Ophrys-gene280603                     0    1    1    0    0   2
Ophrys-gene380365                     0    1    1    1    0   3
Ophrys-gene442717                     1    1    0    1    0   3
Ophrys-gene452395                     1    1    0    0    0   2
Ophrys-gene496733                     1    0    1    1    0   3
Ophrys-gene563071                     1    1    1    1    1   5
Ophrys-gene690320                     1    0    1    1    0   3
Ophrys-gene692714                     0    0    1    1    0   2
Ophrys-gene113692                     1    1    1    1    1   5
Ophrys-gene255850                     1    1    1    1    1   5
Ophrys-gene321693                     0    0    1    0    0   1
Ophrys-gene518106                     1    0    0    1    0   2
Ophrys-gene566442                     1    0    0    1    0   2
Ophrys-gene578284                     1    0    0    1    0   2
Ophrys-gene597807                     1    0    0    1    0   2
Ophrys-gene597931                     1    0    0    1    0   2
Ophrys-gene153215                     1    1    1    1    1   5
Ophrys-gene183998                     0    0    0    1    0   1
Ophrys-gene444549                     0    0    0    1    0   1
Ophrys-gene444713                     1    1    1    1    1   5
Ophrys-gene487972                     0    0    1    0    0   1
Ophrys-gene687648                     1    1    1    1    1   5
Ophrys-gene20402                      1    1    1    1    1   5
Ophrys-gene295193                     1    1    1    1    1   5
Ophrys-gene296417                     1    1    1    1    1   5
Ophrys-gene490041                     1    1    1    1    1   5
Ophrys-gene670749                     0    1    0    0    0   1
Ophrys-gene701676                     1    1    1    1    1   5
Ophrys-gene365                        1    1    1    1    1   5
Ophrys-gene933                        0    1    1    1    0   3
Ophrys-gene1087                       1    0    0    1    0   2
Ophrys-gene1100                       1    1    1    1    1   5
Ophrys-gene2093                       0    0    0    1    0   1
Ophrys-gene4661                       1    1    1    1    1   5
Ophrys-gene4946                       0    0    1    1    0   2
Ophrys-gene5080                       1    1    0    1    0   3
Ophrys-gene5103                       1    1    1    1    1   5
Ophrys-gene7712                       1    1    1    1    0   4
Ophrys-gene7896                       0    0    0    1    0   1
Ophrys-gene8004                       0    0    0    1    0   1
Ophrys-gene8610                       0    0    1    1    0   2
Ophrys-gene8630                       1    0    1    0    0   2
Ophrys-gene9717                       0    0    1    1    0   2
Ophrys-gene9773                       1    1    1    1    1   5
Ophrys-gene11590                      1    1    1    1    1   5
Ophrys-gene20221                      0    0    1    1    0   2
Ophrys-gene20215                      1    1    1    1    1   5
Ophrys-gene20217                      1    0    1    0    0   2
Ophrys-gene21029                      1    0    1    1    0   3
Ophrys-gene24763                      1    1    1    1    1   5
Ophrys-gene38167                      1    0    1    0    0   2
Ophrys-gene39280                      0    0    0    0    0   0
Ophrys-gene40745                      0    1    1    1    0   3
Ophrys-gene45677                      1    1    1    1    1   5
Ophrys-gene46245                      0    0    1    1    0   2
Ophrys-gene46810                      1    1    1    1    1   5
Ophrys-gene47997                      1    0    0    1    0   2
Ophrys-gene48159                      1    1    1    0    0   3
Ophrys-gene50797                      1    1    0    1    0   3
Ophrys-gene52662                      1    1    1    1    1   5
Ophrys-gene54132                      1    1    1    1    1   5
Ophrys-gene54133                      1    1    1    1    1   5
Ophrys-gene54884                      1    0    1    1    0   3
Ophrys-gene55264                      1    1    1    1    1   5
Ophrys-gene59603                      0    0    1    0    0   1
Ophrys-gene59940                      1    0    1    1    0   3
Ophrys-gene60542                      1    1    1    1    1   5
Ophrys-gene60733                      1    0    1    0    0   2
Ophrys-gene62956                      1    1    1    1    1   5
Ophrys-gene65739                      1    1    0    1    0   3
Ophrys-gene67442                      1    0    0    1    0   2
Ophrys-gene68626                      1    1    1    1    1   5
Ophrys-gene68701                      1    0    0    0    0   1
Ophrys-gene69438                      1    1    1    1    0   4
Ophrys-gene69970                      1    0    0    1    0   2
Ophrys-gene71247                      1    1    1    1    1   5
Ophrys-gene76324                      1    1    1    1    1   5
Ophrys-gene77025                      0    0    0    1    0   1
Ophrys-gene76892                      1    1    1    1    1   5
Ophrys-gene76894                      1    1    1    1    1   5
Ophrys-gene76994                      0    0    0    1    0   1
Ophrys-gene77221                      1    1    1    1    1   5
Ophrys-gene81481                      1    1    1    1    1   5
Ophrys-gene84202                      1    1    1    1    1   5
Ophrys-gene84211                      1    0    0    1    0   2
Ophrys-gene86417                      1    0    0    0    0   1
Ophrys-gene88139                      1    1    1    1    1   5
Ophrys-gene88143                      1    1    1    1    1   5
Ophrys-gene90413                      1    1    1    1    1   5
Ophrys-gene91047                      1    1    0    1    0   3
Ophrys-gene94165                      1    0    0    0    0   1
Ophrys-gene97675                      1    1    1    1    1   5
Ophrys-gene100058                     1    1    1    1    1   5
Ophrys-gene101222                     1    1    1    1    1   5
Ophrys-gene102175                     1    1    0    1    0   3
Ophrys-gene103687                     1    1    1    1    1   5
Ophrys-gene104678                     1    0    1    1    0   3
Ophrys-gene111888                     1    1    1    1    1   5
Ophrys-gene112873                     1    0    0    0    0   1
Ophrys-gene113711                     1    1    1    1    1   5
Ophrys-gene113716                     1    1    1    1    1   5
Ophrys-gene113696                     0    0    1    0    0   1
Ophrys-gene116905                     1    1    1    1    0   4
Ophrys-gene118875                     1    0    1    1    0   3
Ophrys-gene120460                     1    0    1    1    0   3
Ophrys-gene120866                     1    1    1    1    1   5
Ophrys-gene120926                     0    0    1    1    0   2
Ophrys-gene122093                     0    0    0    1    0   1
Ophrys-gene124307                     1    1    0    1    0   3
Ophrys-gene126354                     1    1    1    1    1   5
Ophrys-gene132870                     1    0    0    1    0   2
Ophrys-gene132989                     1    1    1    1    1   5
Ophrys-gene133885                     1    0    0    0    0   1
Ophrys-gene134118                     1    0    1    0    0   2
Ophrys-gene136265                     1    1    1    1    1   5
Ophrys-gene138016                     1    0    1    1    0   3
Ophrys-gene138183                     1    1    1    1    1   5
Ophrys-gene141577                     0    0    1    0    0   1
Ophrys-gene149149                     0    0    1    1    0   2
Ophrys-gene149198                     1    0    0    0    0   1
Ophrys-gene149250                     1    0    0    1    0   2
Ophrys-gene151920                     0    0    0    1    0   1
Ophrys-gene152270                     1    1    1    1    1   5
Ophrys-gene152674                     1    1    1    1    1   5
Ophrys-gene155961                     1    1    1    1    1   5
Ophrys-gene165846                     1    0    1    1    0   3
Ophrys-gene167037                     1    1    1    1    1   5
Ophrys-gene167260                     1    0    0    0    0   1
Ophrys-gene167703                     1    0    1    1    0   3
Ophrys-gene168565                     1    0    0    1    0   2
Ophrys-gene169609                     1    0    1    0    0   2
Ophrys-gene169602                     1    1    1    1    1   5
Ophrys-gene170259                     1    1    1    1    1   5
Ophrys-gene170467                     1    0    1    0    0   2
Ophrys-gene171649                     0    0    1    1    0   2
Ophrys-gene171671                     0    0    0    0    0   0
Ophrys-gene173615                     1    1    1    1    1   5
Ophrys-gene173779                     1    1    1    1    1   5
Ophrys-gene174197                     1    1    1    1    1   5
Ophrys-gene177786                     1    1    1    1    1   5
Ophrys-gene178521                     1    1    1    1    1   5
Ophrys-gene178758                     1    1    1    1    1   5
Ophrys-gene178794                     1    1    1    1    1   5
Ophrys-gene180239                     1    1    1    1    1   5
Ophrys-gene181165                     0    0    1    1    0   2
Ophrys-gene181531                     0    0    0    1    0   1
Ophrys-gene181742                     1    0    1    1    0   3
Ophrys-gene182815                     1    1    1    1    1   5
Ophrys-gene182857                     0    0    0    1    0   1
Ophrys-gene182851                     1    0    0    0    0   1
Ophrys-gene183174                     0    0    0    0    0   0
Ophrys-gene186258                     1    1    1    1    1   5
Ophrys-gene186370                     0    0    0    1    0   1
Ophrys-gene189232                     0    0    0    0    0   0
Ophrys-gene190721                     1    0    1    1    0   3
Ophrys-gene190714                     1    1    1    1    1   5
Ophrys-gene194060                     1    1    1    1    1   5
Ophrys-gene194062                     0    0    0    1    0   1
Ophrys-gene194125                     1    0    0    1    0   2
Ophrys-gene194967                     1    0    1    0    0   2
Ophrys-gene197374                     1    1    1    1    1   5
Ophrys-gene199065                     1    0    0    1    0   2
Ophrys-gene203632                     0    0    0    1    0   1
Ophrys-gene203798                     1    0    0    1    0   2
Ophrys-gene205684                     1    0    1    1    0   3
Ophrys-gene207288                     1    1    1    1    1   5
Ophrys-gene207961                     0    0    0    0    0   0
Ophrys-gene208041                     1    0    1    1    0   3
Ophrys-gene208400                     0    0    0    0    0   0
Ophrys-gene210754                     1    1    1    1    1   5
Ophrys-gene210734                     0    0    0    0    0   0
Ophrys-gene210783                     1    0    0    1    0   2
Ophrys-gene210784                     1    0    0    0    0   1
Ophrys-gene210844                     1    0    0    1    0   2
Ophrys-gene213732                     1    1    1    1    1   5
Ophrys-gene215354                     1    0    1    1    0   3
Ophrys-gene215357                     1    0    1    1    0   3
Ophrys-gene215605                     1    0    0    0    0   1
Ophrys-gene218725                     1    0    0    0    0   1
Ophrys-gene218726                     1    1    1    1    1   5
Ophrys-gene219179                     1    1    1    1    1   5
Ophrys-gene219245                     1    1    1    1    1   5
Ophrys-gene222209                     0    0    0    1    0   1
Ophrys-gene222213                     1    0    1    1    0   3
Ophrys-gene235600                     1    1    1    1    1   5
Ophrys-gene250151                     1    0    1    1    0   3
Ophrys-gene251421                     0    0    1    1    0   2
Ophrys-gene252464                     1    1    1    1    1   5
Ophrys-gene254726                     1    1    1    1    1   5
Ophrys-gene256428                     0    0    1    1    0   2
Ophrys-gene258725                     1    0    0    1    0   2
Ophrys-gene258775                     0    0    0    1    1   2
Ophrys-gene259333                     0    0    0    1    0   1
Ophrys-gene259381                     1    0    0    1    0   2
Ophrys-gene262854                     1    1    1    1    1   5
Ophrys-gene265657                     0    0    1    0    0   1
Ophrys-gene265649                     1    1    1    1    1   5
Ophrys-gene265764                     1    1    1    1    1   5
Ophrys-gene268116                     1    0    0    0    0   1
Ophrys-gene271068                     0    0    1    0    0   1
Ophrys-gene271124                     1    0    0    1    0   2
Ophrys-gene277348                     0    0    1    1    0   2
Ophrys-gene277377                     1    1    1    1    1   5
Ophrys-gene278286                     1    1    1    1    1   5
Ophrys-gene278299                     1    0    0    0    0   1
Ophrys-gene278571                     1    1    1    1    1   5
Ophrys-gene280691                     1    1    1    1    1   5
Ophrys-gene281286                     1    0    1    0    0   2
Ophrys-gene282198                     1    1    1    1    1   5
Ophrys-gene284682                     0    0    0    0    0   0
Ophrys-gene285186                     1    1    1    1    1   5
Ophrys-gene286716                     1    1    1    1    1   5
Ophrys-gene288716                     1    1    1    0    0   3
Ophrys-gene291892                     1    1    0    1    0   3
Ophrys-gene291897                     1    0    0    0    0   1
Ophrys-gene293196                     1    0    0    1    0   2
Ophrys-gene295681                     1    0    1    1    0   3
Ophrys-gene298686                     0    0    0    1    0   1
Ophrys-gene301933                     0    0    0    0    0   0
Ophrys-gene301934                     0    1    0    1    0   2
Ophrys-gene302232                     1    1    1    1    1   5
Ophrys-gene304048                     1    1    1    1    1   5
Ophrys-gene305377                     1    0    0    1    0   2
Ophrys-gene318861                     1    1    1    1    1   5
Ophrys-gene324771                     1    0    0    1    0   2
Ophrys-gene331573                     1    1    1    1    1   5
Ophrys-gene339392                     1    0    0    0    0   1
Ophrys-gene340183                     0    0    0    0    0   0
Ophrys-gene341066                     1    1    1    1    1   5
Ophrys-gene341191                     1    1    1    1    1   5
Ophrys-gene342468                     1    0    1    1    0   3
Ophrys-gene342488                     0    1    1    1    0   3
Ophrys-gene343313                     0    0    1    1    0   2
Ophrys-gene344507                     1    1    1    1    1   5
Ophrys-gene348908                     1    1    0    1    0   3
Ophrys-gene361964                     1    1    1    1    1   5
Ophrys-gene369986                     0    0    0    1    0   1
Ophrys-gene369985                     0    0    0    1    0   1
Ophrys-gene373284                     1    0    0    1    0   2
Ophrys-gene375019                     1    1    1    1    1   5
Ophrys-gene376417                     1    1    1    1    1   5
Ophrys-gene386150                     1    1    1    1    1   5
Ophrys-gene386183                     1    0    0    1    0   2
Ophrys-gene386346                     1    0    1    1    0   3
Ophrys-gene390649                     0    0    0    1    0   1
Ophrys-gene390651                     0    0    0    1    0   1
Ophrys-gene390652                     1    0    0    1    0   2
Ophrys-gene392657                     1    0    0    1    0   2
Ophrys-gene392880                     1    1    1    1    1   5
Ophrys-gene394135                     1    0    0    0    0   1
Ophrys-gene395569                     1    0    1    1    0   3
Ophrys-gene395601                     1    1    1    1    1   5
Ophrys-gene395632                     0    0    0    0    0   0
Ophrys-gene395753                     1    0    0    1    0   2
Ophrys-gene395809                     1    0    0    1    0   2
Ophrys-gene395863                     1    0    0    1    0   2
Ophrys-gene399276                     1    1    1    1    1   5
Ophrys-gene399973                     0    0    0    0    0   0
Ophrys-gene400020                     1    0    0    1    0   2
Ophrys-gene403953                     1    0    1    0    0   2
Ophrys-gene406289                     1    1    1    1    1   5
Ophrys-gene412252                     1    0    0    1    0   2
Ophrys-gene413960                     0    0    0    0    0   0
Ophrys-gene427278                     1    0    1    1    0   3
Ophrys-gene427280                     1    0    0    1    0   2
Ophrys-gene429431                     1    1    1    1    1   5
Ophrys-gene429531                     1    1    1    1    1   5
Ophrys-gene431771                     1    1    1    1    1   5
Ophrys-gene432928                     1    0    0    1    0   2
Ophrys-gene435140                     1    1    1    1    1   5
Ophrys-gene435311                     1    1    1    1    1   5
Ophrys-gene444685                     1    0    0    1    0   2
Ophrys-gene444947                     1    0    0    1    0   2
Ophrys-gene446246                     1    1    0    1    0   3
Ophrys-gene448046                     1    1    1    1    1   5
Ophrys-gene451818                     1    1    1    1    1   5
Ophrys-gene453175                     1    1    1    1    1   5
Ophrys-gene464238                     0    0    1    1    0   2
Ophrys-gene467207                     1    0    0    1    0   2
Ophrys-gene472377                     1    1    1    1    1   5
Ophrys-gene473127                     1    1    1    1    1   5
Ophrys-gene477106                     1    1    1    1    1   5
Ophrys-gene477109                     1    1    1    1    1   5
Ophrys-gene477486                     1    1    1    1    1   5
Ophrys-gene483264                     1    1    0    1    0   3
Ophrys-gene483465                     1    0    1    0    0   2
Ophrys-gene484009                     1    1    1    1    1   5
Ophrys-gene484568                     1    1    0    1    0   3
Ophrys-gene484945                     1    0    0    1    0   2
Ophrys-gene488646                     1    1    1    1    1   5
Ophrys-gene488708                     1    0    1    1    1   4
Ophrys-gene490074                     1    0    1    1    0   3
Ophrys-gene492782                     1    1    1    1    1   5
Ophrys-gene492783                     1    0    1    1    0   3
Ophrys-gene495429                     1    1    1    1    1   5
Ophrys-gene495661                     1    0    0    1    0   2
Ophrys-gene495987                     0    0    0    1    0   1
Ophrys-gene496518                     1    0    0    1    0   2
Ophrys-gene506608                     1    1    1    1    1   5
Ophrys-gene508292                     1    0    0    1    0   2
Ophrys-gene509041                     1    1    1    1    1   5
Ophrys-gene513992                     1    1    0    1    0   3
Ophrys-gene518273                     1    1    1    1    1   5
Ophrys-gene519087                     0    0    1    0    0   1
Ophrys-gene523087                     1    1    1    1    1   5
Ophrys-gene525977                     1    1    1    1    1   5
Ophrys-gene526220                     1    1    1    1    1   5
Ophrys-gene528676                     1    0    0    1    0   2
Ophrys-gene528745                     1    0    1    1    0   3
Ophrys-gene532068                     1    0    0    1    0   2
Ophrys-gene541030                     1    0    1    1    0   3
Ophrys-gene542098                     1    1    1    1    1   5
Ophrys-gene542101                     1    1    1    1    1   5
Ophrys-gene543927                     1    1    1    1    1   5
Ophrys-gene551596                     1    1    1    1    1   5
Ophrys-gene551672                     1    1    0    1    0   3
Ophrys-gene554478                     1    0    1    1    0   3
Ophrys-gene554487                     1    1    0    1    0   3
Ophrys-gene555164                     0    0    0    1    0   1
Ophrys-gene555939                     1    1    0    1    0   3
Ophrys-gene559755                     0    0    1    1    0   2
Ophrys-gene561369                     0    0    1    1    0   2
Ophrys-gene564557                     1    0    1    1    0   3
Ophrys-gene565178                     1    0    0    1    0   2
Ophrys-gene565190                     0    0    0    0    0   0
Ophrys-gene565161                     1    1    1    1    0   4
Ophrys-gene565975                     1    0    0    0    0   1
Ophrys-gene568020                     0    0    0    1    0   1
Ophrys-gene579289                     1    0    1    0    0   2
Ophrys-gene579954                     1    0    1    1    0   3
Ophrys-gene581790                     1    1    1    1    1   5
Ophrys-gene585663                     1    1    1    1    1   5
Ophrys-gene586784                     1    0    1    1    0   3
Ophrys-gene587274                     0    0    1    0    0   1
Ophrys-gene587295                     1    1    1    1    1   5
Ophrys-gene587326                     0    0    0    0    0   0
Ophrys-gene591167                     1    0    1    0    0   2
Ophrys-gene595511                     1    1    1    1    1   5
Ophrys-gene598825                     1    0    1    1    0   3
Ophrys-gene603339                     1    1    1    0    0   3
Ophrys-gene603952                     1    0    0    0    0   1
Ophrys-gene606825                     0    0    0    0    0   0
Ophrys-gene607166                     1    1    1    1    1   5
Ophrys-gene610036                     1    1    1    0    0   3
Ophrys-gene610156                     1    0    0    0    0   1
Ophrys-gene613458                     1    1    0    0    0   2
Ophrys-gene615041                     0    0    0    0    0   0
Ophrys-gene616646                     0    0    0    1    0   1
Ophrys-gene616674                     1    0    1    1    0   3
Ophrys-gene617127                     0    0    0    1    0   1
Ophrys-gene617228                     1    1    1    1    1   5
Ophrys-gene617289                     0    0    1    0    0   1
Ophrys-gene617367                     1    0    1    1    0   3
Ophrys-gene617385                     1    1    1    1    1   5
Ophrys-gene619491                     1    1    1    1    1   5
Ophrys-gene625913                     1    1    1    1    1   5
Ophrys-gene625925                     1    0    1    1    0   3
Ophrys-gene628391                     0    0    0    1    0   1
Ophrys-gene636282                     1    0    1    1    0   3
Ophrys-gene639908                     1    0    1    1    0   3
Ophrys-gene641015                     1    1    1    1    1   5
Ophrys-gene641682                     1    0    1    1    0   3
Ophrys-gene642578                     1    0    1    1    0   3
Ophrys-gene649687                     1    0    0    1    0   2
Ophrys-gene654360                     0    1    1    1    0   3
Ophrys-gene656757                     1    0    0    1    0   2
Ophrys-gene661411                     0    0    1    0    0   1
Ophrys-gene661547                     1    1    1    1    1   5
Ophrys-gene662238                     1    0    0    1    0   2
Ophrys-gene663859                     1    1    1    1    1   5
Ophrys-gene669140                     1    0    0    0    0   1
Ophrys-gene670614                     1    0    0    0    0   1
Ophrys-gene675305                     1    1    1    1    1   5
Ophrys-gene676615                     0    0    0    1    0   1
Ophrys-gene676646                     0    1    0    0    0   1
Ophrys-gene679711                     1    0    1    0    0   2
Ophrys-gene687428                     1    1    1    1    1   5
Ophrys-gene689741                     0    0    1    1    0   2
Ophrys-gene690459                     1    0    0    1    0   2
Ophrys-gene692648                     0    1    1    1    0   3
Ophrys-gene693531                     1    0    1    0    1   3
Ophrys-gene695814                     1    1    0    0    0   2
Ophrys-gene696116                     1    1    1    1    0   4
Ophrys-gene698738                     1    1    1    1    1   5
Ophrys-gene699617                     0    0    0    1    0   1
Ophrys-gene702038                     0    0    1    1    0   2
Ophrys-gene707678                     1    0    0    1    0   2
Ophrys-gene709100                     1    0    1    1    0   3
Ophrys-gene709061                     0    0    0    0    0   0
Ophrys-gene709155                     0    0    1    1    0   2
Ophrys-gene709317                     1    1    1    1    1   5
Ophrys-gene709645                     0    0    0    1    0   1
Ophrys-gene712484                     1    0    1    0    0   2
Ophrys-gene717276                     1    1    1    0    0   3
Ophrys-gene720746                     1    1    1    1    1   5
Ophrys-gene722354                     1    1    1    1    1   5
Ophrys-gene726154                     1    1    1    1    1   5
Ophrys-gene726155                     0    0    0    0    0   0
Ophrys-gene726167                     0    0    1    1    0   2
Ophrys-gene731658                     1    0    0    1    0   2
Ophrys-gene731740                     1    0    0    1    0   2
Ophrys-gene732061                     1    0    0    1    0   2
Ophrys-gene739653                     1    0    0    0    0   1
Ophrys-gene747983                     1    0    1    0    0   2
Ophrys-gene757628                     0    0    0    0    0   0
Ophrys-gene765585                     1    0    0    1    0   2
Phalaenopsis-gene184378               0    0    0    1    0   1
Phalaenopsis-gene351864               1    1    1    1    1   5
Phalaenopsis-gene377337               1    1    1    1    1   5
Phalaenopsis-gene467782               0    0    1    0    0   1
Phalaenopsis-gene593292               1    1    1    0    0   3
Phalaenopsis-gene77037                0    0    1    1    0   2
Phalaenopsis-gene897240               0    0    1    1    0   2
Phalaenopsis-gene905674               1    1    1    1    1   5
Phalaenopsis-gene103249               0    0    1    0    0   1
Phalaenopsis-gene128030               0    0    1    1    0   2
Phalaenopsis-gene152832               1    0    1    0    0   2
Phalaenopsis-gene155737               1    1    1    1    1   5
Phalaenopsis-gene165988               1    1    1    1    1   5
Phalaenopsis-gene168545               1    1    1    1    0   4
Phalaenopsis-gene209167               1    0    0    0    0   1
Phalaenopsis-gene209205               1    0    0    0    0   1
Phalaenopsis-gene209243               1    1    1    1    1   5
Phalaenopsis-gene244765               1    1    0    1    0   3
Phalaenopsis-gene254669               1    1    1    1    1   5
Phalaenopsis-gene269492               1    0    0    1    0   2
Phalaenopsis-gene283678               1    1    1    1    1   5
Phalaenopsis-gene296608               1    1    1    1    1   5
Phalaenopsis-gene304727               1    1    1    1    1   5
Phalaenopsis-gene317362               1    0    0    1    0   2
Phalaenopsis-gene320121               1    1    1    1    1   5
Phalaenopsis-gene324440               1    0    1    1    0   3
Phalaenopsis-gene324555               1    0    1    1    0   3
Phalaenopsis-gene333247               1    0    0    1    0   2
Phalaenopsis-gene333263               1    1    1    1    1   5
Phalaenopsis-gene342256               1    0    1    1    0   3
Phalaenopsis-gene344323               1    1    1    1    1   5
Phalaenopsis-gene347872               1    1    1    1    1   5
Phalaenopsis-gene354770               1    0    1    1    0   3
Phalaenopsis-gene354851               0    0    1    0    0   1
Phalaenopsis-gene364322               0    0    1    1    0   2
Phalaenopsis-gene370037               1    0    1    1    0   3
Phalaenopsis-gene404873               1    1    1    1    1   5
Phalaenopsis-gene408054               1    1    1    1    1   5
Phalaenopsis-gene431937               1    1    1    1    1   5
Phalaenopsis-gene444697               1    1    0    1    0   3
Phalaenopsis-gene447182               1    0    1    1    0   3
Phalaenopsis-gene447187               1    0    1    0    0   2
Phalaenopsis-gene448082               1    0    1    1    0   3
Phalaenopsis-gene456302               1    1    1    1    1   5
Phalaenopsis-gene471899               1    1    1    1    1   5
Phalaenopsis-gene476528               1    0    1    1    0   3
Phalaenopsis-gene486037               1    0    0    0    0   1
Phalaenopsis-gene487934               1    1    0    1    0   3
Phalaenopsis-gene50487                1    1    1    1    1   5
Phalaenopsis-gene513875               1    0    1    1    0   3
Phalaenopsis-gene526665               1    0    1    0    0   2
Phalaenopsis-gene570203               1    1    1    1    1   5
Phalaenopsis-gene574469               1    0    1    1    0   3
Phalaenopsis-gene593071               1    0    0    1    0   2
Phalaenopsis-gene60464                1    1    1    1    1   5
Phalaenopsis-gene616007               1    1    1    1    1   5
Phalaenopsis-gene65112                1    1    1    1    1   5
Phalaenopsis-gene662859               1    0    1    1    0   3
Phalaenopsis-gene664813               1    0    1    1    0   3
Phalaenopsis-gene692233               1    0    1    1    0   3
Phalaenopsis-gene694161               0    0    1    1    0   2
Phalaenopsis-gene694165               1    0    0    0    0   1
Phalaenopsis-gene706498               1    0    1    0    0   2
Phalaenopsis-gene73657                1    0    0    1    0   2
Phalaenopsis-gene745886               1    0    0    1    0   2
Phalaenopsis-gene745915               1    1    1    1    1   5
Phalaenopsis-gene750324               1    1    1    1    1   5
Phalaenopsis-gene754213               1    1    1    1    1   5
Phalaenopsis-gene755076               0    0    1    1    0   2
Phalaenopsis-gene776193               1    0    1    1    0   3
Phalaenopsis-gene778915               1    0    0    1    0   2
Phalaenopsis-gene786441               1    0    0    1    0   2
Phalaenopsis-gene788723               0    0    1    1    0   2
Phalaenopsis-gene790759               1    1    1    1    1   5
Phalaenopsis-gene805644               0    1    1    1    0   3
Phalaenopsis-gene812144               1    1    1    1    1   5
Phalaenopsis-gene836317               1    1    1    1    1   5
Phalaenopsis-gene84395                1    0    1    0    0   2
Phalaenopsis-gene857604               0    0    1    1    0   2
Phalaenopsis-gene868324               1    1    1    1    1   5
Phalaenopsis-gene871580               1    0    0    1    0   2
Phalaenopsis-gene892912               1    1    1    1    1   5
Phalaenopsis-gene904179               1    1    1    1    1   5
Phalaenopsis-gene361593               1    0    0    1    0   2
Phalaenopsis-gene578789               1    0    1    1    0   3
Phalaenopsis-gene84145                1    1    1    1    1   5
Phalaenopsis-gene888951               1    1    1    1    1   5
Phalaenopsis-gene903043               1    0    1    1    1   4
Phalaenopsis-gene131304               1    1    1    1    1   5
Phalaenopsis-gene146730               1    0    1    1    0   3
Phalaenopsis-gene151241               1    0    0    0    0   1
Phalaenopsis-gene171944               1    0    1    1    0   3
Phalaenopsis-gene172185               1    0    0    0    0   1
Phalaenopsis-gene201553               1    1    1    0    0   3
Phalaenopsis-gene202278               1    1    1    1    1   5
Phalaenopsis-gene202321               1    0    0    1    0   2
Phalaenopsis-gene204602               1    1    1    1    1   5
Phalaenopsis-gene209204               1    1    1    1    0   4
Phalaenopsis-gene223202               1    1    1    1    1   5
Phalaenopsis-gene247677               1    0    1    0    0   2
Phalaenopsis-gene247702               1    0    1    1    0   3
Phalaenopsis-gene253791               1    1    1    1    1   5
Phalaenopsis-gene266944               1    0    0    1    0   2
Phalaenopsis-gene27077                1    1    1    1    1   5
Phalaenopsis-gene28515                1    0    1    1    0   3
Phalaenopsis-gene286542               1    1    1    1    1   5
Phalaenopsis-gene287412               1    0    1    1    1   4
Phalaenopsis-gene307497               1    0    0    1    0   2
Phalaenopsis-gene307731               1    0    1    1    0   3
Phalaenopsis-gene310208               1    1    1    1    1   5
Phalaenopsis-gene320038               1    0    1    1    0   3
Phalaenopsis-gene325299               1    1    1    1    1   5
Phalaenopsis-gene331603               0    0    1    1    0   2
Phalaenopsis-gene333264               1    1    1    1    1   5
Phalaenopsis-gene343854               1    1    1    1    1   5
Phalaenopsis-gene345103               1    0    1    1    1   4
Phalaenopsis-gene351866               1    1    1    1    1   5
Phalaenopsis-gene370221               1    0    0    1    0   2
Phalaenopsis-gene371404               1    0    0    0    0   1
Phalaenopsis-gene375166               1    0    1    0    0   2
Phalaenopsis-gene385247               1    1    1    1    1   5
Phalaenopsis-gene407699               1    0    0    1    0   2
Phalaenopsis-gene408666               0    0    0    1    0   1
Phalaenopsis-gene419362               1    0    1    0    0   2
Phalaenopsis-gene44315                1    1    1    1    1   5
Phalaenopsis-gene447186               0    0    1    0    0   1
Phalaenopsis-gene450173               1    1    1    1    1   5
Phalaenopsis-gene460468               1    0    1    1    0   3
Phalaenopsis-gene468336               1    0    0    0    0   1
Phalaenopsis-gene471984               0    0    1    0    0   1
Phalaenopsis-gene485962               1    1    1    1    1   5
Phalaenopsis-gene486040               1    0    1    0    1   3
Phalaenopsis-gene487903               0    1    1    1    0   3
Phalaenopsis-gene487937               1    1    1    1    0   4
Phalaenopsis-gene487980               0    0    1    0    0   1
Phalaenopsis-gene520234               1    0    0    0    0   1
Phalaenopsis-gene520247               1    1    1    1    1   5
Phalaenopsis-gene523469               1    0    1    0    0   2
Phalaenopsis-gene539540               1    0    1    1    0   3
Phalaenopsis-gene539544               1    1    1    1    1   5
Phalaenopsis-gene543761               1    1    1    1    1   5
Phalaenopsis-gene549408               1    1    1    1    1   5
Phalaenopsis-gene551026               1    1    1    1    1   5
Phalaenopsis-gene556717               1    1    1    0    0   3
Phalaenopsis-gene570087               0    0    1    1    0   2
Phalaenopsis-gene578054               1    0    0    1    1   3
Phalaenopsis-gene585578               1    0    1    1    0   3
Phalaenopsis-gene593028               0    0    0    1    0   1
Phalaenopsis-gene593072               1    0    0    1    0   2
Phalaenopsis-gene623130               1    0    1    1    0   3
Phalaenopsis-gene629302               1    1    1    1    1   5
Phalaenopsis-gene654298               1    0    1    0    0   2
Phalaenopsis-gene657770               1    0    0    0    0   1
Phalaenopsis-gene659570               1    0    0    1    0   2
Phalaenopsis-gene668705               0    0    1    1    0   2
Phalaenopsis-gene720876               1    1    1    1    1   5
Phalaenopsis-gene725111               1    0    1    1    0   3
Phalaenopsis-gene735675               1    1    1    1    1   5
Phalaenopsis-gene741665               1    0    1    0    0   2
Phalaenopsis-gene747885               1    1    1    1    1   5
Phalaenopsis-gene747908               1    0    1    0    0   2
Phalaenopsis-gene758141               1    0    1    1    0   3
Phalaenopsis-gene759077               1    0    1    1    0   3
Phalaenopsis-gene767932               1    1    1    1    1   5
Phalaenopsis-gene772646               0    0    0    0    0   0
Phalaenopsis-gene784048               1    1    1    1    1   5
Phalaenopsis-gene790651               1    1    1    1    1   5
Phalaenopsis-gene831776               1    0    1    1    0   3
Phalaenopsis-gene83818                1    0    0    1    0   2
Phalaenopsis-gene839820               1    0    1    0    0   2
Phalaenopsis-gene841517               1    1    0    1    0   3
Phalaenopsis-gene851848               1    0    0    1    0   2
Phalaenopsis-gene854741               1    1    1    1    1   5
Phalaenopsis-gene872157               1    0    0    1    0   2
Phalaenopsis-gene897283               1    0    0    1    0   2
Phalaenopsis-gene898906               0    1    0    1    0   2
Phalaenopsis-gene906866               1    1    1    1    1   5
Phalaenopsis-gene215675               1    1    1    1    1   5
Phalaenopsis-gene343279               1    0    1    0    0   2
Phalaenopsis-gene792380               0    1    0    1    0   2
Phalaenopsis-gene856713               1    1    1    1    1   5
Phalaenopsis-gene366985               1    1    1    1    0   4
Phalaenopsis-gene403478               0    0    0    0    0   0
Phalaenopsis-gene51439                1    1    1    1    1   5
Phalaenopsis-gene563906               1    1    1    1    1   5
Phalaenopsis-gene789554               0    0    0    1    1   2
Phalaenopsis-gene90103                0    0    0    0    0   0
Phalaenopsis-gene151091               1    0    1    1    0   3
Phalaenopsis-gene151234               1    0    1    0    0   2
Phalaenopsis-gene155992               0    1    0    0    0   1
Phalaenopsis-gene173929               1    0    0    1    0   2
Phalaenopsis-gene177214               1    1    0    1    0   3
Phalaenopsis-gene18710                1    1    1    1    1   5
Phalaenopsis-gene209166               1    0    1    1    0   3
Phalaenopsis-gene222225               0    0    1    1    0   2
Phalaenopsis-gene232077               1    1    1    1    1   5
Phalaenopsis-gene247724               1    0    1    0    0   2
Phalaenopsis-gene267002               1    0    0    0    0   1
Phalaenopsis-gene267051               1    0    0    1    1   3
Phalaenopsis-gene267108               1    0    0    1    0   2
Phalaenopsis-gene292412               1    1    1    1    1   5
Phalaenopsis-gene319688               1    1    1    1    1   5
Phalaenopsis-gene324450               1    1    1    1    1   5
Phalaenopsis-gene325340               1    0    0    1    0   2
Phalaenopsis-gene333377               1    1    1    1    1   5
Phalaenopsis-gene355476               1    1    1    1    1   5
Phalaenopsis-gene356213               1    1    1    0    0   3
Phalaenopsis-gene361662               1    1    1    1    1   5
Phalaenopsis-gene36713                0    0    0    0    0   0
Phalaenopsis-gene370879               1    1    1    0    0   3
Phalaenopsis-gene424868               1    0    1    1    0   3
Phalaenopsis-gene444177               1    0    1    1    0   3
Phalaenopsis-gene448125               0    0    1    1    0   2
Phalaenopsis-gene539559               1    1    1    1    1   5
Phalaenopsis-gene558820               1    1    1    1    1   5
Phalaenopsis-gene565259               0    0    0    1    0   1
Phalaenopsis-gene578096               0    0    1    0    0   1
Phalaenopsis-gene590810               1    1    0    1    0   3
Phalaenopsis-gene593029               0    0    0    1    0   1
Phalaenopsis-gene616310               1    1    1    1    1   5
Phalaenopsis-gene629325               1    0    0    0    0   1
Phalaenopsis-gene636639               0    0    0    0    0   0
Phalaenopsis-gene636688               1    0    0    1    0   2
Phalaenopsis-gene650701               1    0    1    1    0   3
Phalaenopsis-gene651153               1    0    0    1    0   2
Phalaenopsis-gene686851               1    1    1    1    1   5
Phalaenopsis-gene692160               0    0    1    1    0   2
Phalaenopsis-gene708029               1    0    1    1    1   4
Phalaenopsis-gene735124               0    0    0    1    0   1
Phalaenopsis-gene747383               0    0    1    1    1   3
Phalaenopsis-gene750323               1    0    0    1    0   2
Phalaenopsis-gene763220               1    0    1    1    0   3
Phalaenopsis-gene774316               1    1    1    0    0   3
Phalaenopsis-gene788722               1    1    1    1    1   5
Phalaenopsis-gene788724               1    0    0    1    0   2
Phalaenopsis-gene790975               0    0    0    1    0   1
Phalaenopsis-gene807118               1    0    1    1    0   3
Phalaenopsis-gene807133               1    0    0    1    0   2
Phalaenopsis-gene819463               0    0    0    1    0   1
Phalaenopsis-gene819625               1    1    1    1    1   5
Phalaenopsis-gene839534               1    0    0    1    0   2
Phalaenopsis-gene84226                1    0    0    0    0   1
Phalaenopsis-gene851879               1    1    0    1    0   3
Phalaenopsis-gene866567               1    1    1    1    1   5
Phalaenopsis-gene867427               0    0    0    1    0   1
Phalaenopsis-gene879633               1    1    1    1    1   5
Phalaenopsis-gene882587               0    0    1    1    0   2
Phalaenopsis-gene887190               0    1    1    1    0   3
Phalaenopsis-gene895172               1    0    1    0    0   2
Phalaenopsis-gene905224               1    0    0    1    0   2
Phalaenopsis-gene99824                1    0    1    1    0   3
Phalaenopsis-gene133163               1    1    1    1    1   5
Phalaenopsis-gene136115               1    1    1    1    1   5
Phalaenopsis-gene231762               1    0    0    0    0   1
Phalaenopsis-gene244819               1    0    1    1    0   3
Phalaenopsis-gene266896               0    0    0    1    0   1
Phalaenopsis-gene282235               1    0    0    1    0   2
Phalaenopsis-gene302342               1    0    0    0    0   1
Phalaenopsis-gene302343               1    1    1    1    1   5
Phalaenopsis-gene320039               1    1    1    1    1   5
Phalaenopsis-gene320122               1    1    1    1    1   5
Phalaenopsis-gene34308                1    0    1    1    0   3
Phalaenopsis-gene343829               0    0    1    1    0   2
Phalaenopsis-gene345087               1    1    1    1    0   4
Phalaenopsis-gene349420               1    1    1    1    1   5
Phalaenopsis-gene374398               1    1    1    1    1   5
Phalaenopsis-gene393908               0    1    1    0    0   2
Phalaenopsis-gene487540               1    1    1    1    1   5
Phalaenopsis-gene488038               1    0    0    1    0   2
Phalaenopsis-gene492062               0    1    1    0    0   2
Phalaenopsis-gene516334               1    0    1    0    0   2
Phalaenopsis-gene517958               1    0    1    1    0   3
Phalaenopsis-gene528721               1    1    1    1    1   5
Phalaenopsis-gene547421               1    1    1    1    1   5
Phalaenopsis-gene596119               1    1    1    1    0   4
Phalaenopsis-gene61092                1    0    1    0    0   2
Phalaenopsis-gene614750               1    1    1    1    1   5
Phalaenopsis-gene676909               0    0    1    1    0   2
Phalaenopsis-gene694664               1    0    0    1    0   2
Phalaenopsis-gene706535               1    0    1    0    0   2
Phalaenopsis-gene729567               0    0    1    0    0   1
Phalaenopsis-gene751323               1    1    1    1    1   5
Phalaenopsis-gene758100               1    0    0    0    0   1
Phalaenopsis-gene764195               1    1    1    1    1   5
Phalaenopsis-gene774335               1    1    1    1    1   5
Phalaenopsis-gene774370               1    0    1    1    0   3
Phalaenopsis-gene784029               1    1    1    1    1   5
Phalaenopsis-gene786502               1    0    0    0    0   1
Phalaenopsis-gene860404               1    1    0    1    0   3
Phalaenopsis-gene867464               1    1    1    1    1   5
Phalaenopsis-gene876802               1    1    1    1    1   5
Phalaenopsis-gene881778               1    0    1    1    0   3
Phalaenopsis-gene887151               1    1    0    0    0   2
Phalaenopsis-gene90346                1    0    1    1    0   3
Phalaenopsis-gene908066               1    0    1    0    0   2
Phalaenopsis-gene127757               1    0    0    1    0   2
Phalaenopsis-gene151758               1    0    1    1    0   3
Phalaenopsis-gene18217                1    0    1    0    0   2
Phalaenopsis-gene216672               0    0    0    1    0   1
Phalaenopsis-gene23868                1    0    1    1    1   4
Phalaenopsis-gene27333                0    0    0    1    0   1
Phalaenopsis-gene279610               1    0    0    1    1   3
Phalaenopsis-gene28617                1    0    1    1    0   3
Phalaenopsis-gene34297                1    1    1    1    1   5
Phalaenopsis-gene348468               1    1    0    1    0   3
Phalaenopsis-gene413673               0    0    1    0    0   1
Phalaenopsis-gene443218               1    1    1    1    1   5
Phalaenopsis-gene446774               0    0    0    1    0   1
Phalaenopsis-gene447232               1    0    1    1    0   3
Phalaenopsis-gene460195               1    1    1    1    1   5
Phalaenopsis-gene485715               1    1    1    1    0   4
Phalaenopsis-gene487881               1    0    0    0    0   1
Phalaenopsis-gene490345               1    0    0    0    0   1
Phalaenopsis-gene501529               1    0    1    0    0   2
Phalaenopsis-gene511872               1    0    0    1    0   2
Phalaenopsis-gene51516                1    1    1    1    1   5
Phalaenopsis-gene536709               1    1    1    1    1   5
Phalaenopsis-gene536741               0    0    1    1    1   3
Phalaenopsis-gene566068               1    1    1    1    1   5
Phalaenopsis-gene598713               1    1    1    1    1   5
Phalaenopsis-gene631481               1    0    1    1    0   3
Phalaenopsis-gene657837               1    0    0    1    0   2
Phalaenopsis-gene682285               1    0    0    0    0   1
Phalaenopsis-gene686743               1    0    1    1    0   3
Phalaenopsis-gene692155               1    1    1    1    1   5
Phalaenopsis-gene753967               0    0    0    0    0   0
Phalaenopsis-gene808549               1    1    1    1    1   5
Phalaenopsis-gene816824               1    1    1    1    1   5
Phalaenopsis-gene855394               1    0    0    0    0   1
Phalaenopsis-gene856827               1    0    1    0    0   2
Phalaenopsis-gene857818               0    0    0    0    0   0
Phalaenopsis-gene89034                1    0    0    1    0   2
Phalaenopsis-gene896647               1    1    1    1    0   4
Phalaenopsis-gene343519               1    0    1    1    1   4
Phalaenopsis-gene502172               1    0    1    1    0   3
Phalaenopsis-gene702901               1    1    1    0    0   3
Phalaenopsis-gene238971               1    0    1    1    0   3
Phalaenopsis-gene247313               1    0    0    0    0   1
Phalaenopsis-gene252382               1    0    1    0    0   2
Phalaenopsis-gene846645               1    0    0    1    0   2
Phalaenopsis-gene881695               1    0    1    0    0   2
Phalaenopsis-gene149897               1    1    1    0    0   3
Phalaenopsis-gene181764               1    1    1    1    1   5
Phalaenopsis-gene214645               1    1    0    1    0   3
Phalaenopsis-gene248134               1    0    0    0    0   1
Phalaenopsis-gene293041               1    0    1    1    0   3
Phalaenopsis-gene308895               1    1    1    1    1   5
Phalaenopsis-gene309586               1    0    1    0    0   2
Phalaenopsis-gene324316               1    1    1    1    1   5
Phalaenopsis-gene361634               0    0    0    1    0   1
Phalaenopsis-gene370038               1    0    1    1    0   3
Phalaenopsis-gene400627               0    0    1    1    0   2
Phalaenopsis-gene409771               1    0    1    1    0   3
Phalaenopsis-gene425517               1    0    1    1    0   3
Phalaenopsis-gene448083               1    1    1    1    1   5
Phalaenopsis-gene468478               1    1    1    1    1   5
Phalaenopsis-gene480606               1    1    1    1    0   4
Phalaenopsis-gene484968               1    0    1    1    0   3
Phalaenopsis-gene485023               1    1    1    1    1   5
Phalaenopsis-gene488559               1    1    1    1    1   5
Phalaenopsis-gene503872               1    0    1    0    0   2
Phalaenopsis-gene508005               0    0    0    1    0   1
Phalaenopsis-gene545914               1    0    0    1    0   2
Phalaenopsis-gene588982               0    0    1    0    0   1
Phalaenopsis-gene644730               1    0    0    1    0   2
Phalaenopsis-gene655815               1    1    1    1    1   5
Phalaenopsis-gene663234               1    1    1    1    1   5
Phalaenopsis-gene773913               0    0    0    1    0   1
Phalaenopsis-gene774233               1    0    1    1    0   3
Phalaenopsis-gene839710               1    1    1    1    1   5
Phalaenopsis-gene9344                 1    1    1    1    1   5
Phalaenopsis-gene201594               1    0    0    0    0   1
Phalaenopsis-gene21943                1    1    1    1    1   5
Phalaenopsis-gene353044               1    0    1    1    0   3
Phalaenopsis-gene430495               1    0    1    0    0   2
Phalaenopsis-gene430675               1    0    1    1    0   3
Phalaenopsis-gene431963               0    0    0    1    0   1
Phalaenopsis-gene555703               1    0    0    1    0   2
Phalaenopsis-gene623354               1    0    1    1    0   3
Phalaenopsis-gene734959               1    1    1    1    1   5
Phalaenopsis-gene128923               1    0    0    1    0   2
Phalaenopsis-gene130361               0    0    0    1    0   1
Phalaenopsis-gene152820               1    1    1    1    1   5
Phalaenopsis-gene211378               1    0    1    1    0   3
Phalaenopsis-gene253780               1    1    1    1    1   5
Phalaenopsis-gene302452               1    0    0    0    0   1
Phalaenopsis-gene333749               0    0    1    1    0   2
Phalaenopsis-gene335482               1    0    0    0    0   1
Phalaenopsis-gene361524               1    0    1    1    0   3
Phalaenopsis-gene424843               1    0    1    1    0   3
Phalaenopsis-gene433663               1    1    1    1    0   4
Phalaenopsis-gene486205               0    0    1    1    0   2
Phalaenopsis-gene499420               0    1    1    1    0   3
Phalaenopsis-gene511884               1    0    0    1    0   2
Phalaenopsis-gene523122               0    0    1    1    0   2
Phalaenopsis-gene557576               0    0    1    1    1   3
Phalaenopsis-gene558117               1    0    1    1    0   3
Phalaenopsis-gene585575               0    0    0    0    0   0
Phalaenopsis-gene588154               0    0    0    1    0   1
Phalaenopsis-gene714291               1    0    1    1    0   3
Phalaenopsis-gene723868               1    1    1    1    1   5
Phalaenopsis-gene739679               1    1    1    1    1   5
Phalaenopsis-gene741996               1    1    1    1    1   5
Phalaenopsis-gene854752               1    0    1    0    0   2
Phalaenopsis-gene879628               1    0    1    0    0   2
Phalaenopsis-gene90334                1    1    1    1    1   5
Phalaenopsis-gene522401               1    0    1    0    0   2
Phalaenopsis-gene545637               1    1    1    1    1   5
Phalaenopsis-gene545901               1    0    0    1    0   2
Phalaenopsis-gene560988               0    0    0    0    0   0
Phalaenopsis-gene764276               1    1    1    1    1   5
Phalaenopsis-gene817643               1    0    1    0    0   2
Phalaenopsis-gene106764               0    0    1    1    0   2
Phalaenopsis-gene115256               1    1    1    1    0   4
Phalaenopsis-gene200019               1    1    1    1    1   5
Phalaenopsis-gene222243               1    0    1    1    0   3
Phalaenopsis-gene292556               1    0    1    1    0   3
Phalaenopsis-gene319712               1    0    0    1    0   2
Phalaenopsis-gene370946               1    0    0    1    0   2
Phalaenopsis-gene374399               1    1    1    1    1   5
Phalaenopsis-gene485880               1    0    0    1    0   2
Phalaenopsis-gene503958               1    1    1    1    1   5
Phalaenopsis-gene570093               1    0    0    1    0   2
Phalaenopsis-gene635572               1    1    1    1    1   5
Phalaenopsis-gene673442               1    1    1    1    1   5
Phalaenopsis-gene74688                0    0    0    1    0   1
Phalaenopsis-gene762304               1    0    1    1    0   3
Phalaenopsis-gene787513               1    1    1    1    1   5
Phalaenopsis-gene812196               0    0    1    1    0   2
Phalaenopsis-gene83451                1    1    1    1    1   5
Phalaenopsis-gene84844                1    1    1    1    1   5
Phalaenopsis-gene865841               1    1    1    1    1   5
Phalaenopsis-gene90091                1    0    0    0    0   1
Phalaenopsis-gene593003               1    0    1    1    0   3
Phalaenopsis-gene786162               1    0    1    1    0   3
Phalaenopsis-gene592206               0    0    0    1    0   1
Phalaenopsis-gene723899               1    0    1    1    0   3
Phalaenopsis-gene772429               1    1    1    1    1   5
Phalaenopsis-gene351330               1    0    0    1    0   2
Phalaenopsis-gene444167               1    0    1    0    0   2
Phalaenopsis-gene635527               1    1    1    1    1   5
Phalaenopsis-gene64934                1    0    0    1    0   2
Phalaenopsis-gene716026               1    1    1    1    1   5
Phalaenopsis-gene723838               1    0    1    0    0   2
Phalaenopsis-gene79018                1    0    0    1    0   2
Phalaenopsis-gene860423               1    0    0    1    0   2
Phalaenopsis-gene141025               0    0    0    0    0   0
Phalaenopsis-gene149918               1    0    1    1    0   3
Phalaenopsis-gene1910                 1    1    0    0    0   2
Phalaenopsis-gene202320               1    1    1    1    1   5
Phalaenopsis-gene2057                 1    0    0    1    0   2
Phalaenopsis-gene209168               1    0    0    0    0   1
Phalaenopsis-gene209206               1    0    1    1    0   3
Phalaenopsis-gene245508               1    0    0    1    0   2
Phalaenopsis-gene256461               1    0    0    1    0   2
Phalaenopsis-gene312958               1    0    0    0    0   1
Phalaenopsis-gene347361               1    0    1    1    0   3
Phalaenopsis-gene355406               1    0    0    0    0   1
Phalaenopsis-gene360689               0    0    1    0    0   1
Phalaenopsis-gene36836                0    0    1    0    0   1
Phalaenopsis-gene36861                1    1    1    0    0   3
Phalaenopsis-gene402527               1    1    1    1    1   5
Phalaenopsis-gene408229               1    0    0    1    0   2
Phalaenopsis-gene426589               1    0    0    0    0   1
Phalaenopsis-gene441588               1    1    1    1    1   5
Phalaenopsis-gene491069               1    0    1    0    0   2
Phalaenopsis-gene538060               1    1    1    1    1   5
Phalaenopsis-gene540111               1    1    1    1    1   5
Phalaenopsis-gene543316               0    1    0    1    0   2
Phalaenopsis-gene57196                1    1    1    1    1   5
Phalaenopsis-gene57415                1    1    1    1    1   5
Phalaenopsis-gene627062               1    1    1    1    0   4
Phalaenopsis-gene692239               1    0    0    1    0   2
Phalaenopsis-gene722578               1    0    1    1    0   3
Phalaenopsis-gene752943               1    0    0    1    0   2
Phalaenopsis-gene794123               1    0    1    1    0   3
Phalaenopsis-gene808741               1    0    1    0    0   2
Phalaenopsis-gene408665               1    1    1    0    0   3
Phalaenopsis-gene133293               0    0    1    0    0   1
Phalaenopsis-gene150337               1    0    0    1    0   2
Phalaenopsis-gene196459               1    1    1    1    1   5
Phalaenopsis-gene216916               1    1    0    1    0   3
Phalaenopsis-gene351257               0    0    0    1    0   1
Phalaenopsis-gene439960               1    0    1    1    0   3
Phalaenopsis-gene456989               1    0    0    0    0   1
Phalaenopsis-gene46854                1    0    1    1    0   3
Phalaenopsis-gene56080                1    0    0    1    0   2
Phalaenopsis-gene634501               1    1    1    1    0   4
Phalaenopsis-gene647271               0    0    1    1    0   2
Phalaenopsis-gene649016               1    0    0    1    1   3
Phalaenopsis-gene825726               1    0    1    1    0   3
Phalaenopsis-gene893498               1    0    1    1    0   3
Phalaenopsis-gene28365                1    0    0    0    1   2
Phalaenopsis-gene348560               1    0    1    1    0   3
Phalaenopsis-gene556194               1    0    1    1    0   3
Phalaenopsis-gene742866               0    0    1    1    0   2
Phalaenopsis-gene764438               1    0    1    0    0   2
Phalaenopsis-gene201693               1    0    1    1    0   3
Phalaenopsis-gene635254               1    1    1    1    1   5
Phalaenopsis-gene654442               0    0    1    1    0   2
Phalaenopsis-gene766073               1    0    1    1    0   3
Phalaenopsis-gene786590               1    1    1    1    1   5
Phalaenopsis-gene786669               1    1    1    1    1   5
Phalaenopsis-gene152899               1    0    1    1    1   4
Phalaenopsis-gene152940               1    1    1    1    0   4
Phalaenopsis-gene113091               1    0    0    1    0   2
Phalaenopsis-gene136069               0    1    1    1    0   3
Phalaenopsis-gene168019               1    0    0    0    0   1
Phalaenopsis-gene25674                1    1    1    1    1   5
Phalaenopsis-gene258780               1    1    0    0    0   2
Phalaenopsis-gene273173               0    0    1    0    0   1
Phalaenopsis-gene365841               1    0    1    1    0   3
Phalaenopsis-gene596506               1    0    0    1    0   2
Phalaenopsis-gene97878                1    0    0    1    0   2
Phalaenopsis-gene312959               1    0    1    1    0   3
Phalaenopsis-gene432958               1    1    1    1    1   5
Phalaenopsis-gene722127               1    1    1    1    1   5
Phalaenopsis-gene842920               1    0    0    0    0   1
Phalaenopsis-gene733040               0    0    1    1    0   2
Phalaenopsis-gene110918               1    0    0    0    0   1
Phalaenopsis-gene132798               1    0    0    1    0   2
Phalaenopsis-gene136103               1    1    1    1    1   5
Phalaenopsis-gene138445               0    0    0    1    0   1
Phalaenopsis-gene164218               1    1    1    1    1   5
Phalaenopsis-gene178891               1    0    1    1    0   3
Phalaenopsis-gene21138                1    1    1    1    1   5
Phalaenopsis-gene250355               0    0    0    0    0   0
Phalaenopsis-gene286600               0    0    0    0    0   0
Phalaenopsis-gene286641               1    1    1    1    1   5
Phalaenopsis-gene351857               1    1    1    1    1   5
Phalaenopsis-gene361809               1    0    0    1    0   2
Phalaenopsis-gene450759               1    1    1    1    1   5
Phalaenopsis-gene455710               1    0    0    1    0   2
Phalaenopsis-gene546517               1    1    1    0    0   3
Phalaenopsis-gene585485               1    1    1    1    1   5
Phalaenopsis-gene585509               1    1    1    0    0   3
Phalaenopsis-gene60110                1    0    1    1    0   3
Phalaenopsis-gene771044               0    0    1    0    0   1
Phalaenopsis-gene792183               1    1    1    1    1   5
Phalaenopsis-gene808554               1    1    1    1    1   5
Phalaenopsis-gene907605               1    0    1    1    0   3
Phalaenopsis-gene349081               1    1    1    1    1   5
Phalaenopsis-gene375160               1    1    1    1    1   5
Phalaenopsis-gene388689               1    0    1    1    0   3
Phalaenopsis-gene448024               1    1    1    1    1   5
Phalaenopsis-gene488089               1    0    0    1    0   2
Phalaenopsis-gene543763               1    0    0    1    0   2
Phalaenopsis-gene550862               1    0    1    1    0   3
Phalaenopsis-gene626110               1    1    1    0    0   3
Phalaenopsis-gene660050               1    1    1    1    1   5
Phalaenopsis-gene698589               0    0    1    1    0   2
Phalaenopsis-gene698620               1    0    1    0    0   2
Phalaenopsis-gene735752               1    1    1    1    1   5
Phalaenopsis-gene897728               1    0    1    1    0   3
Phalaenopsis-gene9356                 1    0    1    1    0   3
Phalaenopsis-gene124830               1    0    0    1    0   2
Phalaenopsis-gene432792               1    1    0    0    0   2
Phalaenopsis-gene887146               1    0    0    1    0   2
Phalaenopsis-gene903721               0    1    1    1    0   3
Phalaenopsis-gene184329               0    0    1    0    0   1
Phalaenopsis-gene1911                 1    0    0    1    0   2
Phalaenopsis-gene385950               1    0    1    1    0   3
Phalaenopsis-gene516156               0    0    1    1    0   2
Phalaenopsis-gene548224               1    1    1    1    1   5
Phalaenopsis-gene592796               0    1    0    1    0   2
Phalaenopsis-gene595223               1    0    0    0    0   1
Phalaenopsis-gene635255               0    0    1    0    0   1
Phalaenopsis-gene694120               0    0    0    0    0   0
Phalaenopsis-gene704582               1    1    1    1    1   5
Phalaenopsis-gene747348               1    0    0    1    0   2
Phalaenopsis-gene764114               1    1    1    1    1   5
Phalaenopsis-gene767983               1    1    1    1    1   5
Phalaenopsis-gene806424               1    0    1    0    0   2
Phalaenopsis-gene882352               0    0    0    0    0   0
Phalaenopsis-gene113124               0    0    0    1    0   1
Phalaenopsis-gene116773               1    0    1    0    0   2
Phalaenopsis-gene125344               1    0    0    0    0   1
Phalaenopsis-gene1266                 1    0    0    1    0   2
Phalaenopsis-gene130040               0    0    0    1    0   1
Phalaenopsis-gene130555               1    0    1    0    0   2
Phalaenopsis-gene135530               1    1    1    1    1   5
Phalaenopsis-gene140340               1    1    1    1    1   5
Phalaenopsis-gene143517               1    1    1    1    1   5
Phalaenopsis-gene146731               1    0    1    0    0   2
Phalaenopsis-gene148784               1    1    1    1    1   5
Phalaenopsis-gene148788               1    1    1    1    1   5
Phalaenopsis-gene150094               0    0    0    1    0   1
Phalaenopsis-gene150095               1    1    1    1    1   5
Phalaenopsis-gene150244               0    0    1    0    0   1
Phalaenopsis-gene151098               1    1    1    1    0   4
Phalaenopsis-gene151293               1    0    1    1    0   3
Phalaenopsis-gene152900               1    0    1    0    0   2
Phalaenopsis-gene156008               1    1    1    1    0   4
Phalaenopsis-gene156189               1    0    1    1    0   3
Phalaenopsis-gene15963                1    0    1    0    0   2
Phalaenopsis-gene163754               0    0    0    0    0   0
Phalaenopsis-gene166991               0    0    1    1    0   2
Phalaenopsis-gene167056               0    0    0    1    0   1
Phalaenopsis-gene17139                1    1    1    1    1   5
Phalaenopsis-gene174128               1    0    0    1    0   2
Phalaenopsis-gene177256               1    0    1    1    0   3
Phalaenopsis-gene178899               1    1    0    1    0   3
Phalaenopsis-gene178926               0    0    0    1    0   1
Phalaenopsis-gene178936               1    0    1    1    0   3
Phalaenopsis-gene181773               1    1    1    1    1   5
Phalaenopsis-gene189021               1    0    1    1    0   3
Phalaenopsis-gene194821               1    0    1    0    0   2
Phalaenopsis-gene197962               1    1    1    1    1   5
Phalaenopsis-gene198540               0    0    1    1    0   2
Phalaenopsis-gene202393               0    0    0    0    0   0
Phalaenopsis-gene202409               1    0    1    1    0   3
Phalaenopsis-gene203742               1    0    0    1    0   2
Phalaenopsis-gene203780               1    0    1    0    0   2
Phalaenopsis-gene203781               1    0    0    1    0   2
Phalaenopsis-gene203835               1    0    1    1    0   3
Phalaenopsis-gene214952               1    1    1    1    1   5
Phalaenopsis-gene215655               1    1    1    1    1   5
Phalaenopsis-gene216866               1    0    0    0    0   1
Phalaenopsis-gene216914               1    0    1    1    0   3
Phalaenopsis-gene217419               1    0    1    1    0   3
Phalaenopsis-gene217971               1    0    1    1    0   3
Phalaenopsis-gene217978               1    0    0    1    1   3
Phalaenopsis-gene228728               0    0    1    1    0   2
Phalaenopsis-gene23021                1    1    1    1    1   5
Phalaenopsis-gene23022                1    0    1    1    0   3
Phalaenopsis-gene231763               1    0    1    0    0   2
Phalaenopsis-gene234929               1    0    1    0    0   2
Phalaenopsis-gene234968               1    0    0    1    0   2
Phalaenopsis-gene23784                1    0    0    1    0   2
Phalaenopsis-gene23921                1    0    1    1    0   3
Phalaenopsis-gene241668               0    0    1    1    0   2
Phalaenopsis-gene242898               1    1    1    1    1   5
Phalaenopsis-gene244685               1    1    1    1    0   4
Phalaenopsis-gene244711               1    1    1    1    1   5
Phalaenopsis-gene244735               0    0    0    1    0   1
Phalaenopsis-gene244737               0    0    1    1    1   3
Phalaenopsis-gene244762               0    0    0    0    0   0
Phalaenopsis-gene244764               0    0    0    1    0   1
Phalaenopsis-gene247187               1    0    0    1    0   2
Phalaenopsis-gene247249               1    0    0    1    0   2
Phalaenopsis-gene247258               1    0    1    0    0   2
Phalaenopsis-gene247766               1    0    0    1    0   2
Phalaenopsis-gene251250               1    1    1    1    1   5
Phalaenopsis-gene254649               1    0    0    1    0   2
Phalaenopsis-gene254675               1    0    0    0    0   1
Phalaenopsis-gene254703               0    0    0    1    0   1
Phalaenopsis-gene254707               1    1    1    1    1   5
Phalaenopsis-gene257401               1    1    1    1    1   5
Phalaenopsis-gene258427               1    1    1    1    1   5
Phalaenopsis-gene259965               1    1    1    1    1   5
Phalaenopsis-gene269495               0    0    1    1    0   2
Phalaenopsis-gene269526               1    1    1    1    0   4
Phalaenopsis-gene269529               1    0    1    1    0   3
Phalaenopsis-gene27073                0    0    1    0    0   1
Phalaenopsis-gene27074                1    1    1    1    1   5
Phalaenopsis-gene281419               1    0    1    0    0   2
Phalaenopsis-gene281612               1    0    0    1    0   2
Phalaenopsis-gene285855               1    1    1    1    1   5
Phalaenopsis-gene289318               1    1    1    1    1   5
Phalaenopsis-gene289340               1    1    1    1    0   4
Phalaenopsis-gene291304               1    0    0    0    0   1
Phalaenopsis-gene296617               0    0    1    0    0   1
Phalaenopsis-gene300397               1    1    1    1    1   5
Phalaenopsis-gene300398               1    0    0    1    0   2
Phalaenopsis-gene300875               1    0    0    1    0   2
Phalaenopsis-gene304731               1    1    1    1    1   5
Phalaenopsis-gene306970               1    1    1    1    1   5
Phalaenopsis-gene307855               1    0    1    1    0   3
Phalaenopsis-gene309603               1    1    1    1    1   5
Phalaenopsis-gene309677               0    0    0    0    0   0
Phalaenopsis-gene310206               0    0    1    1    0   2
Phalaenopsis-gene320053               1    1    1    0    0   3
Phalaenopsis-gene320069               1    0    0    1    0   2
Phalaenopsis-gene320070               0    0    1    1    0   2
Phalaenopsis-gene320072               0    0    0    0    0   0
Phalaenopsis-gene320136               1    0    1    0    0   2
Phalaenopsis-gene320153               0    0    1    1    0   2
Phalaenopsis-gene320155               1    0    1    1    0   3
Phalaenopsis-gene320754               1    1    1    1    1   5
Phalaenopsis-gene322504               1    0    0    1    0   2
Phalaenopsis-gene325288               1    0    0    1    0   2
Phalaenopsis-gene325309               1    1    1    1    1   5
Phalaenopsis-gene325565               1    1    1    1    1   5
Phalaenopsis-gene326395               0    0    1    1    0   2
Phalaenopsis-gene333375               0    0    1    0    0   1
Phalaenopsis-gene335898               1    1    1    1    1   5
Phalaenopsis-gene341639               1    1    1    1    1   5
Phalaenopsis-gene343553               1    0    1    1    0   3
Phalaenopsis-gene351242               1    1    1    1    1   5
Phalaenopsis-gene351861               1    0    1    0    0   2
Phalaenopsis-gene361562               0    1    0    1    0   2
Phalaenopsis-gene361780               1    1    1    1    0   4
Phalaenopsis-gene370129               0    0    1    1    0   2
Phalaenopsis-gene370130               0    1    1    1    0   3
Phalaenopsis-gene370222               1    1    1    1    0   4
Phalaenopsis-gene370223               1    0    0    1    0   2
Phalaenopsis-gene370997               1    0    1    0    0   2
Phalaenopsis-gene374915               1    0    1    1    0   3
Phalaenopsis-gene374981               0    0    1    1    0   2
Phalaenopsis-gene375032               1    0    1    1    0   3
Phalaenopsis-gene375161               1    1    1    1    0   4
Phalaenopsis-gene375462               1    0    0    1    0   2
Phalaenopsis-gene375468               1    1    1    1    1   5
Phalaenopsis-gene375658               1    0    0    1    0   2
Phalaenopsis-gene377247               1    1    1    1    1   5
Phalaenopsis-gene385245               1    0    1    1    0   3
Phalaenopsis-gene387278               1    0    0    1    0   2
Phalaenopsis-gene387291               0    1    0    1    0   2
Phalaenopsis-gene387339               1    0    1    1    0   3
Phalaenopsis-gene390123               1    1    1    1    1   5
Phalaenopsis-gene397739               1    1    1    1    1   5
Phalaenopsis-gene408057               1    0    0    1    0   2
Phalaenopsis-gene410975               1    0    1    0    0   2
Phalaenopsis-gene412740               1    1    1    1    0   4
Phalaenopsis-gene413459               1    1    1    1    1   5
Phalaenopsis-gene424275               1    0    1    0    0   2
Phalaenopsis-gene424844               1    0    1    1    0   3
Phalaenopsis-gene424869               1    0    1    1    0   3
Phalaenopsis-gene425245               1    0    0    1    0   2
Phalaenopsis-gene428073               1    0    1    1    0   3
Phalaenopsis-gene429890               1    0    0    0    0   1
Phalaenopsis-gene429904               1    1    1    1    1   5
Phalaenopsis-gene437631               0    0    0    1    0   1
Phalaenopsis-gene437889               1    0    1    1    0   3
Phalaenopsis-gene44213                1    1    1    1    1   5
Phalaenopsis-gene44214                1    1    1    1    1   5
Phalaenopsis-gene44295                1    1    1    1    1   5
Phalaenopsis-gene442998               1    0    0    1    0   2
Phalaenopsis-gene444182               0    1    1    0    1   3
Phalaenopsis-gene444693               1    0    1    1    0   3
Phalaenopsis-gene446794               1    1    0    1    1   4
Phalaenopsis-gene447136               1    1    1    1    1   5
Phalaenopsis-gene447233               1    1    1    1    1   5
Phalaenopsis-gene447270               1    1    1    1    1   5
Phalaenopsis-gene448120               1    1    1    1    1   5
Phalaenopsis-gene452678               1    1    1    1    1   5
Phalaenopsis-gene455610               1    0    1    1    0   3
Phalaenopsis-gene455638               1    1    1    1    1   5
Phalaenopsis-gene467824               1    1    1    1    1   5
Phalaenopsis-gene470831               1    0    0    1    0   2
Phalaenopsis-gene471942               1    0    0    0    0   1
Phalaenopsis-gene477894               1    1    1    1    1   5
Phalaenopsis-gene478575               1    0    1    1    0   3
Phalaenopsis-gene478611               1    0    1    0    0   2
Phalaenopsis-gene479684               0    0    1    1    0   2
Phalaenopsis-gene482061               1    1    1    1    1   5
Phalaenopsis-gene482160               0    1    1    1    0   3
Phalaenopsis-gene482165               1    1    1    1    1   5
Phalaenopsis-gene484338               1    1    1    1    1   5
Phalaenopsis-gene485714               1    0    1    0    0   2
Phalaenopsis-gene488031               1    1    1    1    1   5
Phalaenopsis-gene488057               1    0    1    1    0   3
Phalaenopsis-gene488064               1    0    0    1    0   2
Phalaenopsis-gene492386               1    0    1    1    0   3
Phalaenopsis-gene494235               1    0    0    0    0   1
Phalaenopsis-gene494563               0    0    1    0    0   1
Phalaenopsis-gene495438               1    1    1    1    1   5
Phalaenopsis-gene499415               1    1    1    1    0   4
Phalaenopsis-gene50493                1    1    1    1    1   5
Phalaenopsis-gene511662               1    0    1    1    0   3
Phalaenopsis-gene511663               1    0    1    1    0   3
Phalaenopsis-gene511883               1    1    1    1    1   5
Phalaenopsis-gene514734               1    1    1    1    1   5
Phalaenopsis-gene517044               1    0    1    1    0   3
Phalaenopsis-gene522409               1    0    1    1    0   3
Phalaenopsis-gene522545               1    1    1    1    1   5
Phalaenopsis-gene523071               1    1    1    1    1   5
Phalaenopsis-gene523470               1    1    0    0    0   2
Phalaenopsis-gene524292               1    0    1    1    0   3
Phalaenopsis-gene526666               1    1    1    1    1   5
Phalaenopsis-gene528197               1    0    1    0    0   2
Phalaenopsis-gene533264               1    1    1    1    1   5
Phalaenopsis-gene534061               1    0    1    1    0   3
Phalaenopsis-gene536713               1    0    1    1    0   3
Phalaenopsis-gene545647               1    0    1    1    0   3
Phalaenopsis-gene545911               1    0    1    1    0   3
Phalaenopsis-gene549254               1    1    1    1    1   5
Phalaenopsis-gene551716               1    1    1    1    1   5
Phalaenopsis-gene556716               1    0    0    1    0   2
Phalaenopsis-gene556793               1    0    1    0    0   2
Phalaenopsis-gene556813               1    1    1    1    1   5
Phalaenopsis-gene556815               1    1    1    1    1   5
Phalaenopsis-gene557613               1    1    1    0    0   3
Phalaenopsis-gene558787               0    0    1    1    0   2
Phalaenopsis-gene564727               1    1    1    1    1   5
Phalaenopsis-gene566573               1    1    1    1    1   5
Phalaenopsis-gene570111               1    0    0    0    0   1
Phalaenopsis-gene572966               1    1    1    1    1   5
Phalaenopsis-gene57414                0    0    0    0    0   0
Phalaenopsis-gene57424                1    0    1    0    0   2
Phalaenopsis-gene574266               1    0    0    0    0   1
Phalaenopsis-gene574374               1    0    0    1    0   2
Phalaenopsis-gene574427               1    0    1    1    0   3
Phalaenopsis-gene588730               1    0    1    1    0   3
Phalaenopsis-gene588955               1    0    0    1    0   2
Phalaenopsis-gene588987               1    0    0    1    0   2
Phalaenopsis-gene590537               1    0    1    0    0   2
Phalaenopsis-gene590803               1    1    0    0    0   2
Phalaenopsis-gene592250               1    0    1    1    0   3
Phalaenopsis-gene592875               1    1    1    1    1   5
Phalaenopsis-gene593112               1    0    0    0    0   1
Phalaenopsis-gene593290               0    1    1    0    1   3
Phalaenopsis-gene59343                1    1    1    1    1   5
Phalaenopsis-gene601851               1    0    1    1    0   3
Phalaenopsis-gene606555               1    1    1    1    1   5
Phalaenopsis-gene606557               1    1    1    1    1   5
Phalaenopsis-gene60771                0    0    1    1    0   2
Phalaenopsis-gene609350               1    0    0    0    0   1
Phalaenopsis-gene609352               1    0    1    1    0   3
Phalaenopsis-gene61112                1    1    1    1    0   4
Phalaenopsis-gene612746               1    0    1    1    0   3
Phalaenopsis-gene615950               1    0    0    1    1   3
Phalaenopsis-gene623706               1    0    1    1    0   3
Phalaenopsis-gene623758               1    0    1    0    0   2
Phalaenopsis-gene624173               1    0    1    1    0   3
Phalaenopsis-gene627446               0    0    0    1    0   1
Phalaenopsis-gene628581               1    1    1    1    1   5
Phalaenopsis-gene629637               1    0    0    0    0   1
Phalaenopsis-gene629659               1    0    0    1    0   2
Phalaenopsis-gene629686               1    1    1    1    1   5
Phalaenopsis-gene632992               1    0    1    1    0   3
Phalaenopsis-gene636667               0    0    1    1    0   2
Phalaenopsis-gene636937               1    1    1    1    1   5
Phalaenopsis-gene640184               1    1    1    1    1   5
Phalaenopsis-gene640989               1    0    1    0    0   2
Phalaenopsis-gene641207               1    0    1    1    0   3
Phalaenopsis-gene644309               0    0    1    1    0   2
Phalaenopsis-gene644651               1    0    0    1    0   2
Phalaenopsis-gene651165               1    1    1    1    1   5
Phalaenopsis-gene659569               1    1    1    1    1   5
Phalaenopsis-gene662905               1    1    1    1    1   5
Phalaenopsis-gene66309                1    0    0    1    0   2
Phalaenopsis-gene668423               0    0    0    1    0   1
Phalaenopsis-gene67293                1    0    1    1    0   3
Phalaenopsis-gene677406               1    1    1    1    1   5
Phalaenopsis-gene678670               1    1    1    1    1   5
Phalaenopsis-gene679845               1    1    1    1    1   5
Phalaenopsis-gene679851               1    1    1    1    1   5
Phalaenopsis-gene689687               1    0    0    1    0   2
Phalaenopsis-gene694133               1    1    1    1    1   5
Phalaenopsis-gene694674               1    1    1    1    1   5
Phalaenopsis-gene69653                1    1    1    1    1   5
Phalaenopsis-gene696570               1    1    1    1    1   5
Phalaenopsis-gene698110               1    1    1    1    1   5
Phalaenopsis-gene698616               1    1    1    1    1   5
Phalaenopsis-gene708084               0    0    1    1    0   2
Phalaenopsis-gene709606               1    1    1    1    1   5
Phalaenopsis-gene714255               1    0    1    0    0   2
Phalaenopsis-gene716116               1    1    1    1    1   5
Phalaenopsis-gene724615               1    0    0    1    0   2
Phalaenopsis-gene731569               1    1    1    1    1   5
Phalaenopsis-gene734950               1    0    1    1    0   3
Phalaenopsis-gene741995               0    1    1    1    0   3
Phalaenopsis-gene750328               1    0    1    0    0   2
Phalaenopsis-gene753412               0    0    1    1    0   2
Phalaenopsis-gene754658               1    1    0    1    0   3
Phalaenopsis-gene755041               1    0    0    0    0   1
Phalaenopsis-gene75763                1    1    1    1    1   5
Phalaenopsis-gene758258               1    0    1    0    0   2
Phalaenopsis-gene759071               1    1    1    1    1   5
Phalaenopsis-gene762151               1    1    1    1    1   5
Phalaenopsis-gene763218               0    0    1    1    0   2
Phalaenopsis-gene766025               1    1    1    1    1   5
Phalaenopsis-gene767833               0    0    0    0    0   0
Phalaenopsis-gene772321               0    0    0    1    0   1
Phalaenopsis-gene774390               0    0    1    1    0   2
Phalaenopsis-gene784028               1    0    1    1    0   3
Phalaenopsis-gene786148               1    1    1    1    1   5
Phalaenopsis-gene788725               1    0    1    1    0   3
Phalaenopsis-gene789582               1    1    1    1    1   5
Phalaenopsis-gene790758               1    0    1    0    0   2
Phalaenopsis-gene794052               0    0    0    1    0   1
Phalaenopsis-gene799780               1    1    1    1    1   5
Phalaenopsis-gene805643               1    1    1    1    1   5
Phalaenopsis-gene807100               1    0    1    0    0   2
Phalaenopsis-gene814601               1    1    1    1    1   5
Phalaenopsis-gene817506               1    1    1    1    1   5
Phalaenopsis-gene817582               0    0    0    1    0   1
Phalaenopsis-gene819553               0    0    0    1    0   1
Phalaenopsis-gene824987               1    0    1    1    0   3
Phalaenopsis-gene830988               1    0    0    1    0   2
Phalaenopsis-gene833659               1    1    1    1    1   5
Phalaenopsis-gene833660               1    1    1    1    1   5
Phalaenopsis-gene834144               1    0    1    1    0   3
Phalaenopsis-gene839594               1    0    1    0    0   2
Phalaenopsis-gene85150                1    0    0    1    0   2
Phalaenopsis-gene85176                0    0    0    0    0   0
Phalaenopsis-gene851791               0    1    0    1    0   2
Phalaenopsis-gene854617               0    1    1    1    0   3
Phalaenopsis-gene854624               1    0    1    0    0   2
Phalaenopsis-gene854625               1    0    0    1    0   2
Phalaenopsis-gene855413               1    0    0    1    0   2
Phalaenopsis-gene857617               1    0    1    1    0   3
Phalaenopsis-gene857638               1    0    1    1    0   3
Phalaenopsis-gene857698               1    0    1    1    0   3
Phalaenopsis-gene857814               0    0    1    1    0   2
Phalaenopsis-gene857837               1    1    1    1    1   5
Phalaenopsis-gene860422               1    0    0    0    0   1
Phalaenopsis-gene866797               1    1    1    1    1   5
Phalaenopsis-gene876681               1    1    0    1    0   3
Phalaenopsis-gene881805               1    1    1    1    1   5
Phalaenopsis-gene882068               1    0    1    1    0   3
Phalaenopsis-gene884728               1    0    1    1    0   3
Phalaenopsis-gene888203               1    1    1    1    1   5
Phalaenopsis-gene89065                1    0    0    1    0   2
Phalaenopsis-gene891511               1    1    0    1    0   3
Phalaenopsis-gene89312                0    0    0    0    0   0
Phalaenopsis-gene893456               0    0    0    0    0   0
Phalaenopsis-gene897331               1    0    1    0    0   2
Phalaenopsis-gene903714               0    0    0    1    0   1
Phalaenopsis-gene905102               1    0    0    1    0   2
Phalaenopsis-gene905114               1    1    1    1    1   5
Phalaenopsis-gene908042               1    0    0    1    0   2
Phalaenopsis-gene9338                 1    0    0    0    0   1
Phalaenopsis-gene96304                1    1    1    1    1   5
Phalaenopsis-gene96892                1    0    0    1    0   2
Phalaenopsis-gene97062                1    0    0    1    0   2
Phalaenopsis-gene98829                1    1    1    1    1   5
Ophrys-gene536887                     0    0    0    0    0   0
Ophrys-gene597590                     1    0    0    1    0   2
Ophrys-gene6801                       1    0    0    1    0   2
Ophrys-gene106575                     1    1    1    1    1   5
Ophrys-gene303756                     1    1    1    1    1   5
Ophrys-gene630255                     1    1    1    1    1   5
Ophrys-gene284388                     1    0    0    0    0   1
Ophrys-gene295255                     1    1    1    1    1   5
Ophrys-gene446682                     1    1    1    1    1   5
Ophrys-gene490080                     1    0    0    1    0   2
Ophrys-gene525688                     1    0    0    0    0   1
Ophrys-gene571423                     0    0    0    1    0   1
Ophrys-gene680114                     1    1    1    1    1   5
Ophrys-gene697948                     1    0    0    0    0   1
Ophrys-gene760435                     1    0    1    0    0   2
Ophrys-gene1839                       1    1    1    1    1   5
Ophrys-gene2099                       1    1    1    1    1   5
Ophrys-gene4697                       1    0    1    0    0   2
Ophrys-gene7606                       1    1    1    1    1   5
Ophrys-gene8991                       1    0    0    1    0   2
Ophrys-gene10936                      1    1    1    1    1   5
Ophrys-gene12116                      1    1    1    1    1   5
Ophrys-gene21739                      0    0    0    1    0   1
Ophrys-gene21742                      1    1    1    1    1   5
Ophrys-gene23840                      1    1    1    1    1   5
Ophrys-gene55266                      1    1    1    1    1   5
Ophrys-gene59602                      1    0    0    1    0   2
Ophrys-gene71254                      1    1    1    1    1   5
Ophrys-gene76280                      1    0    0    1    0   2
Ophrys-gene76327                      1    1    1    1    1   5
Ophrys-gene76916                      1    1    1    1    1   5
Ophrys-gene77218                      1    1    1    1    1   5
Ophrys-gene84206                      1    1    1    1    1   5
Ophrys-gene88437                      1    0    0    1    0   2
Ophrys-gene91052                      1    0    1    1    0   3
Ophrys-gene99886                      0    0    1    0    0   1
Ophrys-gene99943                      1    0    0    1    0   2
Ophrys-gene100022                     1    0    0    1    0   2
Ophrys-gene102624                     1    1    1    1    0   4
Ophrys-gene106557                     1    1    1    1    1   5
Ophrys-gene107287                     1    0    1    1    0   3
Ophrys-gene110493                     0    0    1    1    1   3
Ophrys-gene124096                     0    0    0    0    0   0
Ophrys-gene125881                     1    1    1    1    1   5
Ophrys-gene131887                     1    1    1    1    1   5
Ophrys-gene134799                     1    1    1    1    1   5
Ophrys-gene134803                     1    0    1    1    0   3
Ophrys-gene149036                     0    1    0    1    0   2
Ophrys-gene152269                     1    0    1    0    0   2
Ophrys-gene155373                     0    1    1    1    0   3
Ophrys-gene158726                     1    1    1    1    1   5
Ophrys-gene168528                     1    1    1    1    1   5
Ophrys-gene184147                     0    0    1    1    0   2
Ophrys-gene187063                     1    0    1    1    0   3
Ophrys-gene195077                     1    1    1    1    0   4
Ophrys-gene203612                     0    0    0    1    0   1
Ophrys-gene207969                     1    1    1    1    1   5
Ophrys-gene208617                     0    0    1    1    0   2
Ophrys-gene223286                     1    0    0    1    0   2
Ophrys-gene223287                     1    1    1    1    1   5
Ophrys-gene224231                     1    0    0    0    0   1
Ophrys-gene236042                     1    0    1    1    0   3
Ophrys-gene245753                     1    0    1    1    0   3
Ophrys-gene258507                     0    0    0    1    0   1
Ophrys-gene265751                     1    1    1    1    1   5
Ophrys-gene272614                     1    1    1    1    1   5
Ophrys-gene272822                     1    0    0    0    0   1
Ophrys-gene278483                     1    1    1    1    1   5
Ophrys-gene280690                     1    1    1    1    1   5
Ophrys-gene284345                     1    0    1    1    0   3
Ophrys-gene286225                     1    0    0    1    0   2
Ophrys-gene289541                     1    1    1    1    1   5
Ophrys-gene291934                     0    0    1    1    0   2
Ophrys-gene295271                     1    1    1    1    1   5
Ophrys-gene342467                     1    1    1    1    1   5
Ophrys-gene367982                     1    1    0    0    0   2
Ophrys-gene369022                     0    0    0    1    0   1
Ophrys-gene380372                     0    0    1    1    0   2
Ophrys-gene386411                     0    0    0    1    0   1
Ophrys-gene388851                     1    1    1    1    1   5
Ophrys-gene395724                     1    0    1    1    0   3
Ophrys-gene395864                     1    1    1    1    1   5
Ophrys-gene445049                     1    0    0    0    0   1
Ophrys-gene477588                     1    0    1    0    0   2
Ophrys-gene480774                     1    0    0    1    0   2
Ophrys-gene491455                     1    0    1    1    0   3
Ophrys-gene493076                     1    0    0    1    0   2
Ophrys-gene495929                     1    1    1    1    1   5
Ophrys-gene505091                     0    0    0    1    0   1
Ophrys-gene507983                     1    1    1    1    1   5
Ophrys-gene559756                     1    0    1    0    0   2
Ophrys-gene563255                     1    0    0    1    0   2
Ophrys-gene565184                     1    0    1    1    0   3
Ophrys-gene566329                     1    0    0    0    0   1
Ophrys-gene566338                     1    1    1    1    1   5
Ophrys-gene566373                     1    1    1    1    1   5
Ophrys-gene579502                     1    0    1    1    0   3
Ophrys-gene586928                     1    1    1    1    1   5
Ophrys-gene591400                     0    1    1    0    0   2
Ophrys-gene606876                     1    1    1    1    1   5
Ophrys-gene608263                     1    1    1    1    1   5
Ophrys-gene610158                     1    0    0    1    0   2
Ophrys-gene610359                     1    1    1    1    1   5
Ophrys-gene617220                     1    1    1    1    1   5
Ophrys-gene617384                     0    0    0    1    0   1
Ophrys-gene629889                     1    1    1    1    1   5
Ophrys-gene654879                     1    1    1    1    1   5
Ophrys-gene677076                     1    0    1    0    0   2
Ophrys-gene682238                     1    1    1    1    1   5
Ophrys-gene695926                     1    0    1    0    0   2
Ophrys-gene699618                     1    1    1    1    1   5
Ophrys-gene709657                     1    1    1    1    1   5
Ophrys-gene731865                     1    0    0    1    0   2
Ophrys-gene735340                     1    0    1    1    0   3
Ophrys-gene756287                     1    0    0    0    0   1
Ophrys-gene6364                       1    1    1    1    1   5
Ophrys-gene6758                       1    1    1    1    1   5
Ophrys-gene10551                      1    1    1    1    0   4
Ophrys-gene11115                      1    0    0    1    0   2
Ophrys-gene11405                      1    0    0    0    0   1
Ophrys-gene20220                      1    0    0    0    0   1
Ophrys-gene41697                      1    1    1    1    1   5
Ophrys-gene50241                      1    0    1    1    0   3
Ophrys-gene60397                      1    1    1    1    1   5
Ophrys-gene62118                      1    1    1    1    1   5
Ophrys-gene64039                      1    1    1    1    1   5
Ophrys-gene65919                      0    0    0    1    0   1
Ophrys-gene71637                      0    0    0    1    0   1
Ophrys-gene75166                      0    0    0    1    0   1
Ophrys-gene76980                      0    0    1    1    0   2
Ophrys-gene77217                      0    0    0    1    0   1
Ophrys-gene77905                      1    0    1    0    0   2
Ophrys-gene80993                      1    1    1    1    1   5
Ophrys-gene88297                      1    1    1    1    1   5
Ophrys-gene90423                      1    0    1    0    0   2
Ophrys-gene114625                     1    1    1    1    1   5
Ophrys-gene126483                     1    0    0    1    0   2
Ophrys-gene134878                     1    1    1    1    1   5
Ophrys-gene137137                     1    1    1    1    1   5
Ophrys-gene138455                     1    1    1    1    1   5
Ophrys-gene154546                     1    1    1    1    1   5
Ophrys-gene166463                     1    1    1    1    1   5
Ophrys-gene166642                     1    0    1    1    0   3
Ophrys-gene166931                     1    0    0    1    0   2
Ophrys-gene169610                     1    0    1    0    0   2
Ophrys-gene176526                     1    1    1    1    1   5
Ophrys-gene176715                     1    1    1    1    1   5
Ophrys-gene183493                     0    0    0    0    0   0
Ophrys-gene187135                     1    0    0    0    0   1
Ophrys-gene206048                     1    0    0    0    0   1
Ophrys-gene206050                     0    1    0    1    0   2
Ophrys-gene206084                     1    0    0    1    0   2
Ophrys-gene214166                     1    1    1    1    1   5
Ophrys-gene214655                     0    1    0    1    0   2
Ophrys-gene238841                     0    0    1    1    0   2
Ophrys-gene239199                     1    1    0    0    0   2
Ophrys-gene243829                     1    1    1    1    1   5
Ophrys-gene255023                     1    1    1    1    1   5
Ophrys-gene258447                     1    1    1    1    1   5
Ophrys-gene258616                     0    1    0    1    0   2
Ophrys-gene258622                     1    1    1    1    1   5
Ophrys-gene263847                     1    0    0    1    0   2
Ophrys-gene271477                     1    1    1    1    1   5
Ophrys-gene278518                     1    0    0    1    0   2
Ophrys-gene278519                     1    0    0    1    0   2
Ophrys-gene282446                     1    0    0    1    0   2
Ophrys-gene284488                     1    1    0    1    1   4
Ophrys-gene286161                     1    0    0    1    1   3
Ophrys-gene291898                     1    1    1    1    1   5
Ophrys-gene296422                     1    0    1    0    0   2
Ophrys-gene304558                     1    0    1    1    0   3
Ophrys-gene304562                     1    1    1    1    1   5
Ophrys-gene305094                     0    0    0    1    0   1
Ophrys-gene305376                     1    1    1    1    1   5
Ophrys-gene338397                     1    1    1    0    0   3
Ophrys-gene341069                     0    0    1    1    0   2
Ophrys-gene343350                     1    0    0    1    0   2
Ophrys-gene343464                     1    0    0    0    0   1
Ophrys-gene343575                     1    0    0    0    0   1
Ophrys-gene343687                     1    0    0    1    0   2
Ophrys-gene353949                     1    1    1    1    1   5
Ophrys-gene375064                     1    0    0    1    0   2
Ophrys-gene375740                     1    0    0    1    0   2
Ophrys-gene378089                     1    1    1    1    1   5
Ophrys-gene386355                     1    1    0    1    0   3
Ophrys-gene388293                     0    0    1    1    0   2
Ophrys-gene393164                     0    0    0    1    0   1
Ophrys-gene395602                     1    1    1    1    1   5
Ophrys-gene400251                     0    0    0    1    0   1
Ophrys-gene404508                     0    1    0    1    0   2
Ophrys-gene412254                     1    1    0    1    0   3
Ophrys-gene413958                     1    1    1    1    1   5
Ophrys-gene425095                     0    0    1    1    0   2
Ophrys-gene431770                     1    0    0    1    0   2
Ophrys-gene435883                     1    0    1    1    0   3
Ophrys-gene437205                     1    0    1    0    0   2
Ophrys-gene440178                     1    0    0    1    0   2
Ophrys-gene446245                     0    0    1    0    0   1
Ophrys-gene448811                     1    1    1    1    1   5
Ophrys-gene452943                     1    0    0    0    0   1
Ophrys-gene462292                     0    0    0    1    0   1
Ophrys-gene495658                     1    1    0    1    0   3
Ophrys-gene495829                     0    0    0    1    0   1
Ophrys-gene495992                     0    0    0    1    0   1
Ophrys-gene496469                     1    0    0    1    0   2
Ophrys-gene508472                     1    1    1    1    1   5
Ophrys-gene509226                     1    0    0    0    0   1
Ophrys-gene519092                     1    1    1    1    1   5
Ophrys-gene536128                     1    1    1    1    1   5
Ophrys-gene536784                     1    1    0    1    0   3
Ophrys-gene540416                     1    1    1    1    1   5
Ophrys-gene551603                     1    0    1    0    0   2
Ophrys-gene564563                     1    1    0    1    0   3
Ophrys-gene570768                     1    0    0    0    0   1
Ophrys-gene570928                     1    1    1    1    1   5
Ophrys-gene579276                     1    0    0    1    0   2
Ophrys-gene579955                     1    1    1    1    1   5
Ophrys-gene587277                     1    0    0    1    0   2
Ophrys-gene588621                     1    0    0    0    0   1
Ophrys-gene588786                     1    0    0    1    0   2
Ophrys-gene591107                     0    0    0    1    0   1
Ophrys-gene598180                     1    1    0    1    0   3
Ophrys-gene608074                     1    0    0    1    0   2
Ophrys-gene609975                     1    1    1    1    1   5
Ophrys-gene609977                     1    0    0    1    0   2
Ophrys-gene613851                     1    0    0    0    0   1
Ophrys-gene619490                     0    0    1    1    0   2
Ophrys-gene625912                     1    1    1    1    1   5
Ophrys-gene628220                     1    0    0    0    0   1
Ophrys-gene654361                     1    0    1    0    0   2
Ophrys-gene660479                     1    0    0    1    1   3
Ophrys-gene660480                     1    1    1    1    1   5
Ophrys-gene661672                     1    1    1    1    1   5
Ophrys-gene670660                     0    0    0    0    0   0
Ophrys-gene676227                     1    1    1    1    1   5
Ophrys-gene700936                     1    1    1    1    1   5
Ophrys-gene708977                     0    0    0    0    0   0
Ophrys-gene709280                     1    0    0    0    0   1
Ophrys-gene715340                     1    1    1    1    1   5
Ophrys-gene718513                     1    1    1    1    1   5
Ophrys-gene727846                     1    0    0    1    0   2
Ophrys-gene739687                     1    0    0    0    0   1
Ophrys-gene758068                     1    1    1    1    1   5
Ophrys-gene8584                       1    0    1    0    0   2
Ophrys-gene11100                      1    1    1    1    1   5
Ophrys-gene24380                      1    1    1    1    1   5
Ophrys-gene48182                      0    0    1    0    0   1
Ophrys-gene64486                      1    0    1    1    1   4
Ophrys-gene67495                      1    0    0    0    0   1
Ophrys-gene73889                      0    0    0    1    0   1
Ophrys-gene81938                      1    0    0    1    0   2
Ophrys-gene83762                      1    0    1    1    0   3
Ophrys-gene85394                      1    1    1    1    1   5
Ophrys-gene111861                     1    0    0    1    0   2
Ophrys-gene113710                     1    1    1    1    1   5
Ophrys-gene116201                     0    0    1    1    0   2
Ophrys-gene122165                     1    0    1    1    0   3
Ophrys-gene139681                     1    1    1    1    1   5
Ophrys-gene141906                     1    1    1    1    1   5
Ophrys-gene144370                     0    0    1    0    0   1
Ophrys-gene158432                     1    1    1    1    1   5
Ophrys-gene162782                     1    0    0    1    0   2
Ophrys-gene167261                     1    1    1    1    1   5
Ophrys-gene178473                     0    0    1    1    0   2
Ophrys-gene180240                     1    1    1    1    1   5
Ophrys-gene186259                     0    0    0    1    0   1
Ophrys-gene186788                     1    0    0    1    0   2
Ophrys-gene186973                     1    1    1    1    1   5
Ophrys-gene210805                     1    1    1    1    1   5
Ophrys-gene244773                     1    1    1    1    1   5
Ophrys-gene258386                     1    0    0    0    0   1
Ophrys-gene258566                     0    0    0    1    0   1
Ophrys-gene280655                     1    1    0    0    0   2
Ophrys-gene285269                     1    0    1    1    0   3
Ophrys-gene298042                     1    1    1    1    1   5
Ophrys-gene304232                     0    0    1    1    0   2
Ophrys-gene307487                     1    1    1    1    1   5
Ophrys-gene324710                     0    0    0    1    0   1
Ophrys-gene342462                     1    0    1    0    0   2
Ophrys-gene365197                     1    0    0    1    0   2
Ophrys-gene375162                     1    0    0    0    0   1
Ophrys-gene383358                     1    1    0    1    0   3
Ophrys-gene395754                     1    0    1    1    0   3
Ophrys-gene408686                     1    1    0    1    0   3
Ophrys-gene427283                     0    1    0    1    0   2
Ophrys-gene440798                     1    1    1    1    1   5
Ophrys-gene446256                     1    1    1    1    1   5
Ophrys-gene467272                     1    0    0    1    0   2
Ophrys-gene486012                     1    1    1    1    1   5
Ophrys-gene505101                     1    1    1    1    1   5
Ophrys-gene510360                     1    1    1    1    1   5
Ophrys-gene515822                     1    1    0    1    0   3
Ophrys-gene526212                     1    1    1    1    1   5
Ophrys-gene536789                     1    1    1    1    1   5
Ophrys-gene549850                     1    0    1    0    0   2
Ophrys-gene571419                     1    1    1    1    1   5
Ophrys-gene571433                     0    0    0    0    0   0
Ophrys-gene596241                     1    1    0    1    0   3
Ophrys-gene632851                     1    1    1    1    0   4
Ophrys-gene656769                     1    1    1    1    1   5
Ophrys-gene692027                     1    1    1    1    1   5
Ophrys-gene692740                     1    0    1    1    0   3
Ophrys-gene700998                     1    0    1    1    0   3
Ophrys-gene701024                     1    0    0    0    0   1
Ophrys-gene713617                     0    0    0    0    0   0
Ophrys-gene763265                     1    1    1    1    1   5
Ophrys-gene765747                     1    1    1    1    1   5
Ophrys-gene307357                     1    0    0    0    0   1
Ophrys-gene339410                     1    1    1    1    1   5
Ophrys-gene442547                     1    0    0    0    0   1
Ophrys-gene487205                     0    0    0    1    0   1
Ophrys-gene487340                     1    1    1    1    1   5
Ophrys-gene537474                     0    0    0    0    0   0
Ophrys-gene618333                     1    1    1    1    1   5
Ophrys-gene618427                     1    0    1    0    0   2
Ophrys-gene670836                     0    1    0    0    0   1
Ophrys-gene104647                     0    1    0    1    0   2
Ophrys-gene715746                     0    0    1    0    0   1
Ophrys-gene21099                      1    0    1    0    0   2
Ophrys-gene66907                      1    1    1    1    1   5
Ophrys-gene251357                     1    1    1    1    1   5
Ophrys-gene6779                       1    0    1    1    0   3
Ophrys-gene20062                      1    1    1    1    1   5
Ophrys-gene78253                      1    1    0    1    0   3
Ophrys-gene116904                     1    0    0    1    0   2
Ophrys-gene141885                     1    1    1    1    1   5
Ophrys-gene168067                     1    1    0    0    0   2
Ophrys-gene171648                     1    1    1    1    0   4
Ophrys-gene200237                     1    0    0    1    0   2
Ophrys-gene212131                     0    1    0    0    0   1
Ophrys-gene222519                     1    0    0    0    0   1
Ophrys-gene254936                     0    0    0    0    0   0
Ophrys-gene278478                     1    0    1    1    0   3
Ophrys-gene278484                     0    1    0    1    0   2
Ophrys-gene278516                     1    0    0    0    0   1
Ophrys-gene295484                     1    1    1    1    1   5
Ophrys-gene339384                     1    0    0    0    0   1
Ophrys-gene347931                     1    0    1    1    0   3
Ophrys-gene361866                     0    0    0    1    0   1
Ophrys-gene374926                     1    0    0    1    0   2
Ophrys-gene386371                     0    1    0    0    0   1
Ophrys-gene433164                     1    0    1    0    1   3
Ophrys-gene446327                     1    0    0    0    0   1
Ophrys-gene510601                     1    1    0    1    0   3
Ophrys-gene560146                     1    0    0    1    0   2
Ophrys-gene565914                     1    1    1    1    1   5
Ophrys-gene626190                     1    1    1    1    1   5
Ophrys-gene656756                     1    0    1    0    0   2
Ophrys-gene665461                     1    1    1    1    1   5
Ophrys-gene665959                     1    0    1    1    0   3
Ophrys-gene680820                     1    1    1    1    1   5
Ophrys-gene5348                       1    1    0    1    0   3
Ophrys-gene67444                      0    0    1    0    0   1
Ophrys-gene72068                      1    0    1    0    0   2
Ophrys-gene138018                     1    1    1    1    1   5
Ophrys-gene181815                     1    0    0    1    0   2
Ophrys-gene452007                     1    0    0    0    0   1
Ophrys-gene616218                     1    1    1    1    1   5
Ophrys-gene650961                     1    1    1    1    1   5
Ophrys-gene258662                     1    0    0    1    0   2
Ophrys-gene300262                     1    0    0    1    0   2
Ophrys-gene2122                       1    1    1    1    1   5
Ophrys-gene3369                       1    0    1    1    1   4
Ophrys-gene10712                      1    1    1    1    1   5
Ophrys-gene21810                      1    1    1    1    1   5
Ophrys-gene40744                      1    1    1    1    1   5
Ophrys-gene48222                      1    1    1    1    1   5
Ophrys-gene62123                      1    0    0    1    0   2
Ophrys-gene72116                      1    1    1    1    1   5
Ophrys-gene72133                      1    0    0    0    0   1
Ophrys-gene113677                     1    0    0    0    0   1
Ophrys-gene120867                     1    1    1    1    1   5
Ophrys-gene121387                     1    0    0    1    0   2
Ophrys-gene145215                     1    1    1    1    1   5
Ophrys-gene154923                     1    1    1    1    1   5
Ophrys-gene167038                     1    1    1    1    1   5
Ophrys-gene176515                     1    1    1    1    1   5
Ophrys-gene184112                     1    0    0    1    0   2
Ophrys-gene194045                     1    1    1    1    1   5
Ophrys-gene196230                     1    1    0    1    0   3
Ophrys-gene201109                     1    0    1    1    0   3
Ophrys-gene208619                     0    0    1    1    0   2
Ophrys-gene210364                     1    1    1    1    1   5
Ophrys-gene210812                     1    0    0    1    0   2
Ophrys-gene238928                     1    1    1    1    1   5
Ophrys-gene258674                     1    0    0    1    0   2
Ophrys-gene278912                     0    1    1    1    0   3
Ophrys-gene291680                     1    1    1    1    1   5
Ophrys-gene298976                     1    1    1    1    1   5
Ophrys-gene298984                     1    1    1    0    0   3
Ophrys-gene304876                     0    0    0    1    0   1
Ophrys-gene349615                     1    1    1    1    1   5
Ophrys-gene365144                     1    0    0    1    0   2
Ophrys-gene369339                     1    1    1    1    1   5
Ophrys-gene375209                     1    1    1    1    1   5
Ophrys-gene383214                     1    0    0    1    0   2
Ophrys-gene395810                     1    0    1    0    0   2
Ophrys-gene421495                     1    1    1    1    0   4
Ophrys-gene446274                     0    0    0    1    0   1
Ophrys-gene447284                     1    1    1    1    1   5
Ophrys-gene464231                     1    1    1    1    1   5
Ophrys-gene508808                     1    1    1    1    1   5
Ophrys-gene511879                     1    0    0    0    0   1
Ophrys-gene565173                     1    1    1    1    1   5
Ophrys-gene568023                     1    0    1    1    0   3
Ophrys-gene571434                     1    1    1    1    0   4
Ophrys-gene578287                     0    0    0    1    0   1
Ophrys-gene591246                     1    0    1    1    0   3
Ophrys-gene609429                     1    1    1    1    1   5
Ophrys-gene612307                     1    0    0    1    0   2
Ophrys-gene636280                     1    0    1    1    0   3
Ophrys-gene654375                     1    0    1    1    0   3
Ophrys-gene673008                     1    1    1    1    1   5
Ophrys-gene690173                     1    0    0    0    0   1
Ophrys-gene9600                       1    0    1    0    0   2
Ophrys-gene59971                      1    1    1    1    1   5
Ophrys-gene83731                      1    1    1    1    1   5
Ophrys-gene109291                     1    1    1    1    1   5
Ophrys-gene146641                     1    0    0    1    0   2
Ophrys-gene173275                     1    1    1    1    1   5
Ophrys-gene305095                     0    0    0    1    0   1
Ophrys-gene307224                     1    1    1    1    1   5
Ophrys-gene385430                     1    1    1    1    1   5
Ophrys-gene386761                     0    1    0    1    0   2
Ophrys-gene390986                     1    1    1    1    1   5
Ophrys-gene619524                     1    0    1    1    0   3
Ophrys-gene697260                     1    0    0    1    0   2
Ophrys-gene769394                     1    1    1    1    1   5
Ophrys-gene1498                       1    0    1    1    0   3
Ophrys-gene6177                       1    1    1    1    1   5
Ophrys-gene10142                      0    0    1    1    0   2
Ophrys-gene21972                      1    1    1    1    1   5
Ophrys-gene59943                      1    0    0    1    0   2
Ophrys-gene86269                      1    1    1    1    1   5
Ophrys-gene88235                      1    0    0    0    0   1
Ophrys-gene94906                      1    0    0    1    0   2
Ophrys-gene149292                     1    0    0    0    0   1
Ophrys-gene157987                     1    1    1    1    1   5
Ophrys-gene200921                     1    1    1    1    1   5
Ophrys-gene203714                     1    0    1    1    0   3
Ophrys-gene209738                     1    1    1    1    1   5
Ophrys-gene222221                     0    0    0    0    0   0
Ophrys-gene228847                     1    1    1    1    1   5
Ophrys-gene271680                     0    0    0    1    0   1
Ophrys-gene313055                     1    1    1    1    1   5
Ophrys-gene319007                     1    1    1    1    1   5
Ophrys-gene365024                     1    1    1    1    1   5
Ophrys-gene392586                     1    0    0    1    0   2
Ophrys-gene397315                     1    0    0    1    0   2
Ophrys-gene399275                     1    1    1    1    1   5
Ophrys-gene399972                     0    0    0    1    0   1
Ophrys-gene425174                     1    1    1    1    1   5
Ophrys-gene441693                     1    1    1    1    1   5
Ophrys-gene484472                     1    0    1    0    0   2
Ophrys-gene526004                     0    0    0    1    0   1
Ophrys-gene582812                     1    0    0    1    0   2
Ophrys-gene587314                     0    1    1    1    0   3
Ophrys-gene612410                     1    1    1    1    1   5
Ophrys-gene617122                     1    0    0    1    1   3
Ophrys-gene641668                     1    1    1    1    1   5
Ophrys-gene681952                     1    1    1    1    1   5
Ophrys-gene687432                     0    0    0    1    0   1
Ophrys-gene709232                     1    1    0    1    0   3
Ophrys-gene735552                     0    0    0    1    0   1
Ophrys-gene759919                     1    0    0    1    0   2
Ophrys-gene10137                      1    1    1    1    1   5
Ophrys-gene20647                      0    0    1    1    0   2
Ophrys-gene306909                     1    0    0    1    0   2
Ophrys-gene3784                       0    0    1    1    0   2
Ophrys-gene6066                       1    1    1    1    1   5
Ophrys-gene107748                     1    0    1    0    0   2
Ophrys-gene197868                     1    1    1    1    1   5
Ophrys-gene249137                     1    1    1    1    1   5
Ophrys-gene304244                     1    1    1    1    1   5
Ophrys-gene365570                     1    1    1    1    1   5
Ophrys-gene386706                     0    0    0    1    0   1
Ophrys-gene449960                     1    0    1    1    0   3
Ophrys-gene528779                     1    1    1    1    1   5
Ophrys-gene596289                     1    0    0    0    0   1
Ophrys-gene670959                     1    0    0    1    0   2
Ophrys-gene916                        1    0    1    0    0   2
Ophrys-gene58888                      1    0    1    1    0   3
Ophrys-gene61417                      1    0    1    0    0   2
Ophrys-gene68428                      1    1    1    1    1   5
Ophrys-gene74199                      1    0    0    1    0   2
Ophrys-gene103688                     1    0    1    1    0   3
Ophrys-gene178978                     1    0    0    1    0   2
Ophrys-gene238905                     1    1    1    1    1   5
Ophrys-gene278513                     1    0    1    0    0   2
Ophrys-gene324687                     1    1    1    1    1   5
Ophrys-gene349614                     1    1    1    1    1   5
Ophrys-gene395666                     0    0    1    1    0   2
Ophrys-gene436261                     1    0    0    1    0   2
Ophrys-gene493107                     1    1    1    1    1   5
Ophrys-gene526297                     1    0    0    1    0   2
Ophrys-gene536738                     1    0    0    1    0   2
Ophrys-gene570830                     0    1    1    0    0   2
Ophrys-gene696636                     1    1    1    1    1   5
Ophrys-gene64278                      1    1    1    1    1   5
Ophrys-gene127686                     1    1    1    1    1   5
Ophrys-gene335800                     1    1    1    1    1   5
Ophrys-gene352177                     1    0    0    1    0   2
Ophrys-gene467267                     0    0    0    0    0   0
Ophrys-gene542126                     1    0    0    1    0   2
Ophrys-gene8600                       1    0    0    1    0   2
Ophrys-gene306707                     0    0    0    0    0   0
Ophrys-gene452348                     1    1    1    1    1   5
Ophrys-gene298866                     1    0    0    0    0   1
Ophrys-gene395866                     1    0    1    0    0   2
Ophrys-gene434512                     1    1    1    0    0   3
Ophrys-gene510613                     0    0    0    1    0   1
Ophrys-gene10411                      1    1    1    1    1   5
Ophrys-gene22341                      1    0    0    1    0   2
Ophrys-gene38166                      1    1    1    1    1   5
Ophrys-gene41336                      1    1    1    1    1   5
Ophrys-gene45679                      1    1    1    1    1   5
Ophrys-gene48203                      1    1    1    1    1   5
Ophrys-gene67100                      1    1    1    1    1   5
Ophrys-gene73731                      1    1    1    1    1   5
Ophrys-gene76995                      1    0    0    1    0   2
Ophrys-gene92777                      0    0    0    1    0   1
Ophrys-gene122094                     1    0    1    0    0   2
Ophrys-gene206121                     1    1    1    1    1   5
Ophrys-gene209718                     1    1    1    1    1   5
Ophrys-gene240674                     1    1    0    0    0   2
Ophrys-gene295475                     1    0    1    1    0   3
Ophrys-gene324761                     1    1    0    1    0   3
Ophrys-gene340146                     1    0    1    1    0   3
Ophrys-gene393511                     1    1    1    1    1   5
Ophrys-gene399341                     0    0    0    0    0   0
Ophrys-gene405059                     1    0    1    0    0   2
Ophrys-gene408681                     1    0    0    1    0   2
Ophrys-gene440281                     1    0    1    1    1   4
Ophrys-gene442246                     1    0    0    1    0   2
Ophrys-gene628424                     1    0    0    1    0   2
Ophrys-gene639890                     0    1    1    0    0   2
Ophrys-gene669082                     1    1    1    1    1   5
Ophrys-gene676272                     1    1    1    1    1   5
Ophrys-gene676486                     0    0    0    1    0   1
Ophrys-gene699694                     1    1    1    1    1   5
Ophrys-gene1137                       1    1    1    1    1   5
Ophrys-gene22658                      1    1    1    1    1   5
Ophrys-gene41337                      1    0    0    1    0   2
Ophrys-gene65183                      1    1    0    1    0   3
Ophrys-gene73888                      0    0    1    1    0   2
Ophrys-gene146517                     1    0    0    1    0   2
Ophrys-gene159472                     0    0    1    1    0   2
Ophrys-gene168603                     0    0    0    1    0   1
Ophrys-gene172438                     0    0    0    1    0   1
Ophrys-gene183909                     0    0    0    1    0   1
Ophrys-gene184057                     1    0    1    1    0   3
Ophrys-gene225530                     1    0    0    1    0   2
Ophrys-gene228898                     1    0    0    1    0   2
Ophrys-gene230050                     1    1    1    1    1   5
Ophrys-gene240135                     1    1    1    1    1   5
Ophrys-gene258495                     0    0    1    1    0   2
Ophrys-gene258763                     1    0    1    1    0   3
Ophrys-gene324785                     0    0    1    1    0   2
Ophrys-gene335795                     1    0    1    1    0   3
Ophrys-gene390991                     1    0    0    0    0   1
Ophrys-gene395726                     0    0    1    1    0   2
Ophrys-gene429452                     1    1    1    1    1   5
Ophrys-gene436212                     1    0    0    1    0   2
Ophrys-gene448796                     1    1    1    1    1   5
Ophrys-gene448810                     1    1    1    1    1   5
Ophrys-gene454999                     1    1    1    1    1   5
Ophrys-gene457307                     1    1    1    1    1   5
Ophrys-gene511840                     1    0    0    0    0   1
Ophrys-gene511987                     1    0    0    1    0   2
Ophrys-gene526217                     1    0    1    1    0   3
Ophrys-gene528359                     1    1    1    1    1   5
Ophrys-gene571435                     1    1    1    1    1   5
Ophrys-gene577280                     1    1    1    1    1   5
Ophrys-gene591678                     1    1    1    1    1   5
Ophrys-gene646783                     0    0    1    1    0   2
Ophrys-gene696457                     1    1    1    1    1   5
Ophrys-gene36130                      1    1    1    1    1   5
Ophrys-gene94908                      0    0    1    1    0   2
Ophrys-gene109008                     1    1    1    1    1   5
Ophrys-gene134470                     1    0    0    1    0   2
Ophrys-gene155478                     1    1    1    1    1   5
Ophrys-gene160454                     1    1    1    1    1   5
Ophrys-gene213020                     1    0    0    0    0   1
Ophrys-gene213088                     0    0    0    1    0   1
Ophrys-gene247245                     1    0    0    1    0   2
Ophrys-gene255008                     1    1    1    1    1   5
Ophrys-gene306766                     1    1    1    1    1   5
Ophrys-gene424988                     0    0    0    0    0   0
Ophrys-gene429032                     1    1    1    1    0   4
Ophrys-gene430225                     1    0    0    1    0   2
Ophrys-gene435954                     1    1    1    1    1   5
Ophrys-gene446638                     1    1    1    1    1   5
Ophrys-gene499356                     1    0    0    1    0   2
Ophrys-gene528206                     0    0    0    0    0   0
Ophrys-gene546740                     1    0    0    0    1   2
Ophrys-gene548954                     1    1    1    1    1   5
Ophrys-gene595708                     1    0    0    0    0   1
Ophrys-gene618425                     0    0    0    1    0   1
Ophrys-gene710448                     1    1    1    1    1   5
Ophrys-gene118953                     0    1    0    1    0   2
Ophrys-gene146385                     0    0    0    0    0   0
Ophrys-gene304349                     1    0    0    1    0   2
Ophrys-gene702091                     1    0    1    0    0   2
Ophrys-gene8900                       0    0    0    1    0   1
Ophrys-gene104744                     1    1    0    0    0   2
Ophrys-gene251422                     1    1    1    1    1   5
Ophrys-gene526231                     1    0    0    1    0   2
Ophrys-gene3506                       0    0    0    0    0   0
Ophrys-gene109292                     1    1    1    1    1   5
Ophrys-gene467206                     1    0    0    1    0   2
Ophrys-gene635340                     1    1    1    1    1   5
Ophrys-gene69219                      1    1    1    1    1   5
Ophrys-gene132872                     1    0    1    1    0   3
Ophrys-gene140940                     1    1    1    1    1   5
Ophrys-gene215388                     0    0    0    1    0   1
Ophrys-gene280488                     1    0    0    1    0   2
Ophrys-gene291448                     1    0    0    1    0   2
Ophrys-gene291592                     1    0    0    0    0   1
Ophrys-gene298991                     1    0    1    1    0   3
Ophrys-gene375753                     0    0    0    0    0   0
Ophrys-gene386353                     1    0    0    1    0   2
Ophrys-gene386413                     1    1    1    1    1   5
Ophrys-gene387153                     1    1    1    1    1   5
Ophrys-gene391765                     1    1    1    1    1   5
Ophrys-gene394145                     1    0    1    1    0   3
Ophrys-gene395571                     1    0    1    1    0   3
Ophrys-gene597937                     1    0    0    1    0   2
Ophrys-gene607151                     0    1    0    1    0   2
Ophrys-gene618331                     0    0    0    0    0   0
Ophrys-gene697448                     1    1    1    1    1   5
Ophrys-gene697726                     0    0    0    1    0   1
Ophrys-gene697894                     1    1    1    1    1   5
Ophrys-gene700176                     1    1    1    1    1   5
Ophrys-gene721713                     1    1    1    1    1   5
Ophrys-gene731283                     1    1    1    1    1   5
Ophrys-gene731519                     1    1    1    1    1   5
Ophrys-gene390657                     0    1    0    1    0   2
Ophrys-gene935                        1    1    1    1    1   5
Ophrys-gene23376                      1    1    1    1    1   5
Ophrys-gene50845                      0    1    0    0    0   1
Ophrys-gene56620                      1    0    0    0    0   1
Ophrys-gene68709                      1    0    1    1    0   3
Ophrys-gene160737                     0    0    1    0    0   1
Ophrys-gene258380                     1    0    1    1    0   3
Ophrys-gene258441                     1    0    1    1    0   3
Ophrys-gene258560                     1    1    1    1    0   4
Ophrys-gene536888                     0    0    0    1    0   1
Ophrys-gene591290                     1    1    1    1    1   5
Ophrys-gene598828                     1    1    1    1    1   5
Ophrys-gene610038                     0    0    0    0    0   0
Ophrys-gene610216                     0    0    0    1    0   1
Ophrys-gene610330                     1    1    0    1    0   3
Ophrys-gene610391                     0    0    0    0    0   0
Ophrys-gene613462                     1    0    0    1    0   2
Ophrys-gene619610                     1    1    1    1    1   5
Ophrys-gene628365                     1    1    1    1    1   5
Ophrys-gene711650                     1    0    1    0    0   2
Ophrys-gene145214                     1    0    0    1    1   3
Ophrys-gene146452                     1    1    1    1    1   5
Ophrys-gene511758                     1    1    0    1    0   3
Ophrys-gene597472                     1    1    1    1    1   5
Ophrys-gene7175                       1    1    1    1    1   5
Ophrys-gene10011                      1    1    1    1    1   5
Ophrys-gene21779                      0    1    0    1    0   2
Ophrys-gene56127                      1    1    1    1    1   5
Ophrys-gene65485                      1    1    1    1    1   5
Ophrys-gene70987                      1    1    1    1    1   5
Ophrys-gene149200                     1    0    0    0    0   1
Ophrys-gene213015                     1    1    1    1    1   5
Ophrys-gene254921                     1    1    1    1    1   5
Ophrys-gene291638                     0    0    0    1    0   1
Ophrys-gene351656                     1    0    0    1    0   2
Ophrys-gene370261                     1    0    1    0    0   2
Ophrys-gene404734                     0    0    0    1    0   1
Ophrys-gene525629                     1    1    1    1    1   5
Ophrys-gene582813                     1    0    0    1    0   2
Ophrys-gene656961                     1    0    1    1    0   3
Ophrys-gene701667                     1    1    1    1    1   5
Phalaenopsis-gene178928               1    1    1    1    1   5
Phalaenopsis-gene209858               1    0    1    1    1   4
Phalaenopsis-gene385244               1    0    0    1    0   2
Phalaenopsis-gene663426               1    1    0    0    0   2
Phalaenopsis-gene726811               1    0    1    1    0   3
Phalaenopsis-gene882558               1    0    1    0    0   2
Phalaenopsis-gene432849               0    0    0    0    0   0
Phalaenopsis-gene504591               0    0    0    1    0   1
Phalaenopsis-gene658918               1    1    1    1    1   5
Phalaenopsis-gene310470               1    1    0    1    1   4
Phalaenopsis-gene317766               1    1    1    1    1   5
Phalaenopsis-gene355364               1    0    1    1    0   3
Phalaenopsis-gene361694               0    1    0    1    0   2
Phalaenopsis-gene688251               1    0    0    0    0   1
Phalaenopsis-gene697897               1    1    1    1    1   5
Phalaenopsis-gene110693               1    0    0    1    0   2
Phalaenopsis-gene137168               1    1    1    1    1   5
Phalaenopsis-gene150243               1    1    0    1    1   4
Phalaenopsis-gene152941               1    0    1    1    0   3
Phalaenopsis-gene170900               0    1    1    0    0   2
Phalaenopsis-gene188750               1    1    1    1    1   5
Phalaenopsis-gene197893               1    0    1    1    0   3
Phalaenopsis-gene203836               1    0    1    0    0   2
Phalaenopsis-gene21032                1    0    1    1    0   3
Phalaenopsis-gene21087                1    0    1    1    0   3
Phalaenopsis-gene223072               1    1    1    1    1   5
Phalaenopsis-gene23783                1    0    1    0    0   2
Phalaenopsis-gene244709               1    1    0    1    0   3
Phalaenopsis-gene247181               1    0    0    0    0   1
Phalaenopsis-gene247207               1    0    1    0    0   2
Phalaenopsis-gene259955               1    1    1    1    0   4
Phalaenopsis-gene274265               1    0    1    1    0   3
Phalaenopsis-gene291312               1    1    1    1    1   5
Phalaenopsis-gene309591               1    0    0    1    0   2
Phalaenopsis-gene320152               1    1    1    1    1   5
Phalaenopsis-gene343562               1    1    1    1    1   5
Phalaenopsis-gene344813               0    0    0    1    0   1
Phalaenopsis-gene347996               1    0    1    1    0   3
Phalaenopsis-gene356218               1    0    1    1    0   3
Phalaenopsis-gene370040               1    0    1    1    0   3
Phalaenopsis-gene371018               0    0    0    0    0   0
Phalaenopsis-gene375265               1    1    0    1    0   3
Phalaenopsis-gene375521               1    0    1    1    0   3
Phalaenopsis-gene376100               1    1    1    1    1   5
Phalaenopsis-gene387300               1    0    1    0    0   2
Phalaenopsis-gene398140               0    0    1    1    0   2
Phalaenopsis-gene414197               0    0    1    1    0   2
Phalaenopsis-gene421977               1    0    1    1    0   3
Phalaenopsis-gene425537               0    0    0    1    0   1
Phalaenopsis-gene429901               1    0    1    1    0   3
Phalaenopsis-gene437332               1    1    0    1    0   3
Phalaenopsis-gene443136               1    1    1    1    1   5
Phalaenopsis-gene443172               1    0    0    1    0   2
Phalaenopsis-gene447090               1    0    0    0    0   1
Phalaenopsis-gene450751               1    1    1    1    1   5
Phalaenopsis-gene456982               1    1    1    1    1   5
Phalaenopsis-gene470832               1    1    1    1    1   5
Phalaenopsis-gene471900               1    0    1    0    0   2
Phalaenopsis-gene486204               1    0    0    0    0   1
Phalaenopsis-gene487910               1    0    0    1    0   2
Phalaenopsis-gene503966               0    0    0    1    0   1
Phalaenopsis-gene504422               1    1    1    1    1   5
Phalaenopsis-gene514730               1    1    1    1    1   5
Phalaenopsis-gene522516               1    1    1    1    1   5
Phalaenopsis-gene533981               1    0    1    1    0   3
Phalaenopsis-gene547108               1    1    1    1    1   5
Phalaenopsis-gene548231               1    0    1    1    0   3
Phalaenopsis-gene548708               1    0    1    1    0   3
Phalaenopsis-gene570137               1    1    0    1    0   3
Phalaenopsis-gene579486               1    0    0    1    0   2
Phalaenopsis-gene590549               1    1    1    1    1   5
Phalaenopsis-gene60086                1    0    0    1    0   2
Phalaenopsis-gene606556               1    1    1    1    1   5
Phalaenopsis-gene623388               1    0    1    1    0   3
Phalaenopsis-gene623605               1    0    0    0    0   1
Phalaenopsis-gene623654               1    0    1    1    0   3
Phalaenopsis-gene630193               1    1    1    1    1   5
Phalaenopsis-gene630760               1    0    0    1    0   2
Phalaenopsis-gene636640               1    0    0    1    0   2
Phalaenopsis-gene636644               1    1    1    1    1   5
Phalaenopsis-gene647234               0    0    1    0    0   1
Phalaenopsis-gene64907                1    1    1    1    1   5
Phalaenopsis-gene662909               1    0    1    1    0   3
Phalaenopsis-gene690122               1    0    1    1    0   3
Phalaenopsis-gene709607               1    0    1    0    0   2
Phalaenopsis-gene751365               1    0    1    0    0   2
Phalaenopsis-gene754655               0    0    1    0    0   1
Phalaenopsis-gene758177               1    0    1    0    0   2
Phalaenopsis-gene766068               1    1    1    0    0   3
Phalaenopsis-gene774391               1    0    0    0    1   2
Phalaenopsis-gene78470                1    0    1    1    0   3
Phalaenopsis-gene78473                1    0    1    1    0   3
Phalaenopsis-gene790767               1    1    1    1    1   5
Phalaenopsis-gene807099               1    0    1    1    0   3
Phalaenopsis-gene807138               1    1    1    1    1   5
Phalaenopsis-gene815136               0    0    1    1    0   2
Phalaenopsis-gene819558               1    0    1    1    0   3
Phalaenopsis-gene819626               1    0    1    1    0   3
Phalaenopsis-gene838851               1    1    1    1    1   5
Phalaenopsis-gene841518               1    1    1    1    1   5
Phalaenopsis-gene856858               1    1    1    1    1   5
Phalaenopsis-gene857661               1    0    0    0    0   1
Phalaenopsis-gene871594               1    1    1    1    1   5
Phalaenopsis-gene872229               1    1    1    1    1   5
Phalaenopsis-gene872370               0    0    0    1    0   1
Phalaenopsis-gene872440               1    0    1    1    0   3
Phalaenopsis-gene892996               0    0    0    1    0   1
Phalaenopsis-gene89308                1    1    1    1    0   4
Phalaenopsis-gene896654               1    0    0    1    0   2
Phalaenopsis-gene901311               1    0    1    1    0   3
Phalaenopsis-gene9991                 1    1    1    1    1   5
Phalaenopsis-gene114496               0    1    1    1    0   3
Phalaenopsis-gene125022               1    0    0    0    0   1
Phalaenopsis-gene126415               1    0    1    0    0   2
Phalaenopsis-gene1267                 1    0    1    1    0   3
Phalaenopsis-gene129544               1    0    1    1    0   3
Phalaenopsis-gene140999               1    1    1    1    1   5
Phalaenopsis-gene143611               0    0    0    0    0   0
Phalaenopsis-gene146112               1    0    1    1    0   3
Phalaenopsis-gene150052               1    0    0    0    0   1
Phalaenopsis-gene152819               1    1    1    1    1   5
Phalaenopsis-gene156061               1    1    1    1    1   5
Phalaenopsis-gene156191               1    1    1    1    1   5
Phalaenopsis-gene160588               1    0    1    1    0   3
Phalaenopsis-gene170931               1    0    1    1    0   3
Phalaenopsis-gene189102               1    0    0    1    0   2
Phalaenopsis-gene198593               1    0    1    0    0   2
Phalaenopsis-gene202406               1    0    1    1    0   3
Phalaenopsis-gene21139                0    0    1    1    0   2
Phalaenopsis-gene213334               1    1    1    1    1   5
Phalaenopsis-gene216863               1    0    1    1    0   3
Phalaenopsis-gene216998               1    1    1    1    1   5
Phalaenopsis-gene222205               0    0    0    1    0   1
Phalaenopsis-gene228737               1    1    1    1    1   5
Phalaenopsis-gene22959                1    0    0    1    0   2
Phalaenopsis-gene229623               1    1    1    1    1   5
Phalaenopsis-gene249072               1    0    1    1    0   3
Phalaenopsis-gene262807               1    0    1    1    0   3
Phalaenopsis-gene266891               1    0    0    0    0   1
Phalaenopsis-gene267103               0    0    0    1    0   1
Phalaenopsis-gene270200               0    0    1    0    0   1
Phalaenopsis-gene281472               1    0    1    1    0   3
Phalaenopsis-gene28584                1    0    1    0    0   2
Phalaenopsis-gene293042               1    1    1    1    1   5
Phalaenopsis-gene307248               0    0    0    0    0   0
Phalaenopsis-gene310220               0    0    1    0    0   1
Phalaenopsis-gene319860               0    0    0    1    0   1
Phalaenopsis-gene320040               1    0    1    1    0   3
Phalaenopsis-gene322842               1    1    1    1    1   5
Phalaenopsis-gene324322               1    1    1    1    1   5
Phalaenopsis-gene324524               1    0    0    0    0   1
Phalaenopsis-gene324619               1    0    1    1    0   3
Phalaenopsis-gene325290               1    1    1    1    1   5
Phalaenopsis-gene332071               1    1    1    1    1   5
Phalaenopsis-gene339891               1    0    1    1    0   3
Phalaenopsis-gene350928               1    1    1    1    1   5
Phalaenopsis-gene355286               1    1    1    1    1   5
Phalaenopsis-gene355320               1    1    1    1    1   5
Phalaenopsis-gene36547                1    1    1    1    1   5
Phalaenopsis-gene3661                 1    0    0    1    0   2
Phalaenopsis-gene36615                1    1    1    1    1   5
Phalaenopsis-gene3662                 1    1    1    1    1   5
Phalaenopsis-gene371905               1    0    0    1    0   2
Phalaenopsis-gene372185               1    0    1    0    0   2
Phalaenopsis-gene374916               1    1    1    1    1   5
Phalaenopsis-gene387986               1    1    1    1    1   5
Phalaenopsis-gene388637               1    0    1    1    0   3
Phalaenopsis-gene404872               1    0    1    1    0   3
Phalaenopsis-gene430370               1    0    0    0    0   1
Phalaenopsis-gene434131               1    1    1    1    1   5
Phalaenopsis-gene438580               0    0    1    1    0   2
Phalaenopsis-gene444675               1    1    1    1    1   5
Phalaenopsis-gene480640               1    1    1    1    1   5
Phalaenopsis-gene484337               1    1    1    1    1   5
Phalaenopsis-gene485958               1    1    0    1    0   3
Phalaenopsis-gene487987               1    0    0    0    0   1
Phalaenopsis-gene489274               1    0    1    0    1   3
Phalaenopsis-gene504018               0    0    1    1    0   2
Phalaenopsis-gene50488                1    0    1    0    0   2
Phalaenopsis-gene508749               1    0    1    1    0   3
Phalaenopsis-gene515896               1    1    1    1    1   5
Phalaenopsis-gene545650               1    1    1    1    1   5
Phalaenopsis-gene548280               1    0    1    1    0   3
Phalaenopsis-gene548709               1    1    1    1    1   5
Phalaenopsis-gene551031               1    1    1    1    1   5
Phalaenopsis-gene568302               1    1    1    0    0   3
Phalaenopsis-gene572971               1    0    0    1    0   2
Phalaenopsis-gene611308               1    1    1    1    1   5
Phalaenopsis-gene61283                0    0    0    0    0   0
Phalaenopsis-gene615961               0    0    0    1    0   1
Phalaenopsis-gene615967               0    0    0    0    0   0
Phalaenopsis-gene621586               1    0    1    1    0   3
Phalaenopsis-gene628943               1    1    1    1    1   5
Phalaenopsis-gene630804               1    0    0    0    0   1
Phalaenopsis-gene635250               0    0    0    0    0   0
Phalaenopsis-gene649408               1    0    1    0    0   2
Phalaenopsis-gene662901               1    1    1    1    1   5
Phalaenopsis-gene664800               1    1    1    1    1   5
Phalaenopsis-gene681225               0    0    0    1    0   1
Phalaenopsis-gene682294               0    0    0    1    0   1
Phalaenopsis-gene689675               0    0    1    1    0   2
Phalaenopsis-gene693121               0    0    0    1    0   1
Phalaenopsis-gene694420               1    0    0    1    0   2
Phalaenopsis-gene713448               1    0    1    0    0   2
Phalaenopsis-gene718789               1    0    1    0    0   2
Phalaenopsis-gene718829               0    0    0    0    0   0
Phalaenopsis-gene723835               1    1    1    1    1   5
Phalaenopsis-gene725498               0    0    1    0    0   1
Phalaenopsis-gene729409               0    0    1    0    0   1
Phalaenopsis-gene73706                1    0    0    1    0   2
Phalaenopsis-gene741168               1    1    1    0    0   3
Phalaenopsis-gene744082               1    1    1    1    1   5
Phalaenopsis-gene745807               1    0    0    1    0   2
Phalaenopsis-gene753945               1    1    1    1    1   5
Phalaenopsis-gene76032                1    0    0    1    0   2
Phalaenopsis-gene763176               1    1    0    1    0   3
Phalaenopsis-gene764817               1    1    1    1    1   5
Phalaenopsis-gene767866               0    0    1    1    0   2
Phalaenopsis-gene774276               1    0    1    1    0   3
Phalaenopsis-gene775491               1    1    1    1    1   5
Phalaenopsis-gene786151               0    0    1    1    0   2
Phalaenopsis-gene789593               1    1    1    1    0   4
Phalaenopsis-gene807861               1    1    1    1    1   5
Phalaenopsis-gene819181               0    0    0    1    0   1
Phalaenopsis-gene856124               1    0    0    1    0   2
Phalaenopsis-gene860403               1    1    1    1    1   5
Phalaenopsis-gene871016               1    1    1    1    1   5
Phalaenopsis-gene876515               1    1    0    1    0   3
Phalaenopsis-gene88501                0    0    0    0    0   0
Phalaenopsis-gene908038               1    0    1    1    0   3
Phalaenopsis-gene92940                1    0    0    1    0   2
Phalaenopsis-gene128918               1    0    0    1    0   2
Phalaenopsis-gene143540               0    0    1    1    0   2
Phalaenopsis-gene172173               1    0    0    0    0   1
Phalaenopsis-gene189056               1    0    1    1    0   3
Phalaenopsis-gene199476               0    0    1    0    0   1
Phalaenopsis-gene201552               1    1    1    0    0   3
Phalaenopsis-gene216892               0    0    0    0    0   0
Phalaenopsis-gene22176                1    1    1    1    1   5
Phalaenopsis-gene222490               0    0    1    1    0   2
Phalaenopsis-gene223063               0    0    1    1    0   2
Phalaenopsis-gene229641               1    1    1    1    1   5
Phalaenopsis-gene233045               1    1    1    1    1   5
Phalaenopsis-gene258756               1    1    1    1    1   5
Phalaenopsis-gene266893               0    0    0    0    0   0
Phalaenopsis-gene267105               1    0    1    1    0   3
Phalaenopsis-gene27062                1    1    1    1    1   5
Phalaenopsis-gene276815               1    1    1    1    1   5
Phalaenopsis-gene276865               0    0    1    1    0   2
Phalaenopsis-gene293039               1    0    1    1    0   3
Phalaenopsis-gene300399               0    0    0    1    0   1
Phalaenopsis-gene306662               1    0    1    0    0   2
Phalaenopsis-gene307372               1    1    1    1    1   5
Phalaenopsis-gene309665               1    1    1    1    1   5
Phalaenopsis-gene319838               1    1    1    1    1   5
Phalaenopsis-gene320054               1    0    1    1    0   3
Phalaenopsis-gene320123               0    0    1    0    0   1
Phalaenopsis-gene343863               1    0    1    1    0   3
Phalaenopsis-gene399111               0    0    1    1    0   2
Phalaenopsis-gene400425               1    1    1    1    1   5
Phalaenopsis-gene437871               1    1    1    0    0   3
Phalaenopsis-gene455665               1    1    1    1    0   4
Phalaenopsis-gene488092               1    1    1    1    1   5
Phalaenopsis-gene493541               1    1    1    1    1   5
Phalaenopsis-gene51447                1    1    1    1    1   5
Phalaenopsis-gene556814               1    1    0    1    0   3
Phalaenopsis-gene570136               1    0    1    0    0   2
Phalaenopsis-gene623454               0    0    0    0    0   0
Phalaenopsis-gene635284               1    0    1    0    0   2
Phalaenopsis-gene694162               0    0    1    1    0   2
Phalaenopsis-gene720877               1    0    0    1    0   2
Phalaenopsis-gene73929                1    0    1    1    0   3
Phalaenopsis-gene751416               1    0    0    0    0   1
Phalaenopsis-gene792182               0    0    1    1    0   2
Phalaenopsis-gene807112               1    0    0    0    0   1
Phalaenopsis-gene820050               1    0    1    1    0   3
Phalaenopsis-gene836699               0    0    1    1    0   2
Phalaenopsis-gene871570               1    0    0    1    0   2
Phalaenopsis-gene893491               1    0    1    1    0   3
Phalaenopsis-gene899784               1    1    1    1    1   5
Phalaenopsis-gene96806                1    1    1    1    1   5
Phalaenopsis-gene155991               1    1    1    1    1   5
Phalaenopsis-gene170859               1    0    0    0    0   1
Phalaenopsis-gene448072               1    0    1    1    0   3
Phalaenopsis-gene456697               1    1    1    1    1   5
Phalaenopsis-gene548168               0    0    1    1    0   2
Phalaenopsis-gene579479               1    0    1    0    1   3
Phalaenopsis-gene741114               1    1    1    1    1   5
Phalaenopsis-gene826085               0    0    1    1    0   2
Phalaenopsis-gene255031               1    1    1    1    1   5
Phalaenopsis-gene286132               1    1    1    1    1   5
Phalaenopsis-gene518603               1    1    1    0    0   3
Phalaenopsis-gene528275               1    1    1    1    1   5
Phalaenopsis-gene309949               1    0    0    0    0   1
Phalaenopsis-gene594721               0    0    0    0    0   0
Phalaenopsis-gene720238               1    1    1    1    1   5
Phalaenopsis-gene734                  1    0    0    1    0   2
Phalaenopsis-gene149919               1    0    1    1    0   3
Phalaenopsis-gene178890               1    1    1    1    1   5
Phalaenopsis-gene188616               1    1    1    1    1   5
Phalaenopsis-gene203182               1    1    1    1    1   5
Phalaenopsis-gene209207               1    0    1    1    0   3
Phalaenopsis-gene228731               1    1    1    1    1   5
Phalaenopsis-gene256517               1    1    1    1    1   5
Phalaenopsis-gene281351               1    0    1    1    0   3
Phalaenopsis-gene319713               1    0    1    1    0   3
Phalaenopsis-gene320062               0    0    0    0    0   0
Phalaenopsis-gene320137               1    1    1    1    1   5
Phalaenopsis-gene339890               1    0    1    1    0   3
Phalaenopsis-gene343566               1    0    0    1    0   2
Phalaenopsis-gene387321               1    0    0    0    0   1
Phalaenopsis-gene402564               1    1    1    1    0   4
Phalaenopsis-gene430585               1    0    0    1    0   2
Phalaenopsis-gene471149               1    0    0    1    0   2
Phalaenopsis-gene494234               1    1    1    1    1   5
Phalaenopsis-gene512225               1    0    1    1    0   3
Phalaenopsis-gene573444               1    1    1    1    1   5
Phalaenopsis-gene611215               0    0    1    1    0   2
Phalaenopsis-gene640365               1    0    1    1    0   3
Phalaenopsis-gene651483               1    1    1    1    1   5
Phalaenopsis-gene668412               1    1    1    1    1   5
Phalaenopsis-gene689641               1    0    1    0    0   2
Phalaenopsis-gene697892               1    0    1    1    0   3
Phalaenopsis-gene774367               1    1    1    1    1   5
Phalaenopsis-gene793289               1    1    1    1    1   5
Phalaenopsis-gene814600               1    1    1    1    0   4
Phalaenopsis-gene819131               1    0    0    0    0   1
Phalaenopsis-gene822773               1    0    1    1    0   3
Phalaenopsis-gene836666               1    0    1    1    0   3
Phalaenopsis-gene859409               1    0    0    1    0   2
Phalaenopsis-gene88283                1    1    1    1    1   5
Phalaenopsis-gene888949               1    0    1    1    0   3
Phalaenopsis-gene891512               1    0    1    1    0   3
Phalaenopsis-gene893889               1    1    1    1    0   4
Phalaenopsis-gene107064               1    1    1    1    1   5
Phalaenopsis-gene361750               0    0    0    0    0   0
Phalaenopsis-gene374336               1    0    1    0    0   2
Phalaenopsis-gene633000               0    1    0    1    0   2
Phalaenopsis-gene640503               1    0    1    1    0   3
Phalaenopsis-gene721704               1    0    0    0    0   1
Phalaenopsis-gene905174               1    1    1    1    1   5
Phalaenopsis-gene93000                1    0    0    1    0   2
Phalaenopsis-gene291478               0    0    0    0    0   0
Phalaenopsis-gene305766               1    0    1    1    0   3
Phalaenopsis-gene447183               1    0    1    1    0   3
Phalaenopsis-gene626948               1    0    0    0    0   1
Phalaenopsis-gene627018               1    0    0    0    0   1
Phalaenopsis-gene11986                0    1    0    1    0   2
Phalaenopsis-gene149944               1    1    1    1    1   5
Phalaenopsis-gene156103               1    1    1    1    1   5
Phalaenopsis-gene163310               1    0    1    0    0   2
Phalaenopsis-gene172220               1    0    1    0    0   2
Phalaenopsis-gene186889               1    1    1    1    1   5
Phalaenopsis-gene232336               1    1    1    1    1   5
Phalaenopsis-gene23869                1    1    1    1    1   5
Phalaenopsis-gene282220               1    0    1    1    0   3
Phalaenopsis-gene287358               0    1    0    1    0   2
Phalaenopsis-gene294872               0    0    1    1    0   2
Phalaenopsis-gene317365               0    0    1    0    0   1
Phalaenopsis-gene320145               1    1    1    1    1   5
Phalaenopsis-gene335172               1    1    0    0    0   2
Phalaenopsis-gene340418               1    0    1    0    0   2
Phalaenopsis-gene344582               1    0    1    0    0   2
Phalaenopsis-gene361825               0    0    0    1    0   1
Phalaenopsis-gene364605               1    1    1    1    1   5
Phalaenopsis-gene371944               1    0    0    0    0   1
Phalaenopsis-gene387290               1    0    1    1    0   3
Phalaenopsis-gene397738               1    1    1    1    1   5
Phalaenopsis-gene407698               0    0    0    1    0   1
Phalaenopsis-gene412715               1    0    0    1    0   2
Phalaenopsis-gene419333               1    1    1    1    1   5
Phalaenopsis-gene429892               1    1    1    1    1   5
Phalaenopsis-gene440011               1    0    1    1    0   3
Phalaenopsis-gene441589               1    1    1    1    1   5
Phalaenopsis-gene455829               1    1    1    1    0   4
Phalaenopsis-gene487537               1    0    1    0    0   2
Phalaenopsis-gene488006               1    0    1    0    0   2
Phalaenopsis-gene495448               1    1    1    1    1   5
Phalaenopsis-gene564809               1    0    0    1    0   2
Phalaenopsis-gene602452               1    0    1    1    0   3
Phalaenopsis-gene621496               1    0    1    0    0   2
Phalaenopsis-gene662893               0    0    0    1    0   1
Phalaenopsis-gene690063               1    0    1    0    0   2
Phalaenopsis-gene690130               0    0    1    1    0   2
Phalaenopsis-gene694123               1    0    0    1    0   2
Phalaenopsis-gene714256               1    0    0    1    0   2
Phalaenopsis-gene731570               1    1    1    1    1   5
Phalaenopsis-gene735126               0    0    0    1    0   1
Phalaenopsis-gene73707                1    0    1    1    0   3
Phalaenopsis-gene749649               1    0    0    0    0   1
Phalaenopsis-gene758217               1    0    1    1    0   3
Phalaenopsis-gene774313               1    0    1    1    0   3
Phalaenopsis-gene807104               1    1    1    1    1   5
Phalaenopsis-gene839551               0    0    1    0    0   1
Phalaenopsis-gene841131               1    1    1    1    1   5
Phalaenopsis-gene857630               1    0    1    0    0   2
Phalaenopsis-gene857690               1    0    1    0    0   2
Phalaenopsis-gene857806               0    1    0    1    0   2
Phalaenopsis-gene875205               1    1    1    1    1   5
Phalaenopsis-gene881706               1    1    0    1    0   3
Phalaenopsis-gene892952               0    0    1    1    0   2
Phalaenopsis-gene89408                1    0    0    0    0   1
Phalaenopsis-gene90083                1    0    1    1    0   3
Phalaenopsis-gene906774               0    1    1    1    0   3
Phalaenopsis-gene158856               1    0    0    1    0   2
Phalaenopsis-gene194622               1    0    0    1    0   2
Phalaenopsis-gene202332               1    1    0    1    0   3
Phalaenopsis-gene351269               1    1    1    0    0   3
Phalaenopsis-gene351342               1    0    0    0    0   1
Phalaenopsis-gene374233               1    1    1    1    1   5
Phalaenopsis-gene440003               0    1    1    0    0   2
Phalaenopsis-gene447138               0    1    1    1    0   3
Phalaenopsis-gene463194               1    0    1    0    0   2
Phalaenopsis-gene463315               0    1    1    1    0   3
Phalaenopsis-gene520224               1    0    0    1    0   2
Phalaenopsis-gene545653               0    0    0    1    0   1
Phalaenopsis-gene546743               1    0    0    1    0   2
Phalaenopsis-gene587733               1    1    0    1    0   3
Phalaenopsis-gene633045               1    1    1    1    1   5
Phalaenopsis-gene673408               0    0    0    1    0   1
Phalaenopsis-gene720015               1    0    1    1    0   3
Phalaenopsis-gene733626               1    0    1    0    0   2
Phalaenopsis-gene882305               0    0    1    1    0   2
Phalaenopsis-gene8865                 0    0    0    1    0   1
Phalaenopsis-gene9086                 0    1    0    0    0   1
Phalaenopsis-gene135349               1    1    1    1    0   4
Phalaenopsis-gene155986               1    0    0    0    0   1
Phalaenopsis-gene15964                1    0    1    0    0   2
Phalaenopsis-gene166761               0    0    1    1    0   2
Phalaenopsis-gene194434               1    1    1    1    1   5
Phalaenopsis-gene274159               1    0    0    1    0   2
Phalaenopsis-gene308832               1    0    0    0    0   1
Phalaenopsis-gene317735               1    0    0    1    0   2
Phalaenopsis-gene37265                0    0    1    1    0   2
Phalaenopsis-gene376077               1    0    1    0    0   2
Phalaenopsis-gene431964               0    0    0    1    0   1
Phalaenopsis-gene433541               1    1    1    0    0   3
Phalaenopsis-gene444156               1    0    0    1    0   2
Phalaenopsis-gene470102               1    0    0    0    0   1
Phalaenopsis-gene549241               1    1    1    1    1   5
Phalaenopsis-gene550166               1    1    1    1    1   5
Phalaenopsis-gene564779               1    0    1    0    0   2
Phalaenopsis-gene567101               1    1    1    1    1   5
Phalaenopsis-gene573426               0    0    1    1    0   2
Phalaenopsis-gene617876               1    0    0    0    0   1
Phalaenopsis-gene677429               1    1    1    1    1   5
Phalaenopsis-gene680786               1    0    1    1    0   3
Phalaenopsis-gene731566               1    0    1    1    0   3
Phalaenopsis-gene734353               1    1    1    1    1   5
Phalaenopsis-gene744229               0    0    1    1    0   2
Phalaenopsis-gene746110               0    0    1    1    0   2
Phalaenopsis-gene753720               1    0    1    1    0   3
Phalaenopsis-gene759070               1    1    1    1    0   4
Phalaenopsis-gene790757               1    1    1    1    1   5
Phalaenopsis-gene819476               0    1    1    0    0   2
Phalaenopsis-gene90326                0    0    0    1    0   1
Phalaenopsis-gene908043               1    1    1    1    1   5
Phalaenopsis-gene150026               0    1    0    1    0   2
Phalaenopsis-gene465777               1    0    0    0    0   1
Phalaenopsis-gene516443               1    0    1    0    0   2
Phalaenopsis-gene516478               1    0    0    1    0   2
Phalaenopsis-gene570116               0    0    0    1    0   1
Phalaenopsis-gene593008               1    0    0    0    0   1
Phalaenopsis-gene664962               1    1    0    1    0   3
Phalaenopsis-gene693167               1    1    1    1    1   5
Phalaenopsis-gene693252               1    0    1    1    0   3
Phalaenopsis-gene831782               0    0    0    0    0   0
Phalaenopsis-gene293386               1    1    1    1    1   5
Phalaenopsis-gene415075               1    1    1    1    1   5
Phalaenopsis-gene416572               1    1    1    1    1   5
Phalaenopsis-gene499359               1    1    1    1    1   5
Phalaenopsis-gene503968               1    1    1    1    1   5
Phalaenopsis-gene555552               1    1    0    1    0   3
Phalaenopsis-gene606957               1    0    0    1    0   2
Phalaenopsis-gene6120                 1    0    0    1    0   2
Phalaenopsis-gene668248               1    1    1    1    1   5
Phalaenopsis-gene759478               1    1    1    1    1   5
Phalaenopsis-gene137224               1    1    1    1    1   5
Phalaenopsis-gene245533               1    0    0    0    0   1
Phalaenopsis-gene252389               1    0    1    1    0   3
Phalaenopsis-gene256060               0    0    0    1    0   1
Phalaenopsis-gene46760                1    1    1    1    1   5
Phalaenopsis-gene495419               1    1    1    1    1   5
Phalaenopsis-gene575471               1    1    1    1    1   5
Phalaenopsis-gene592234               1    0    1    1    0   3
Phalaenopsis-gene592876               1    0    1    0    0   2
Phalaenopsis-gene625496               0    0    1    1    0   2
Phalaenopsis-gene668436               1    0    1    0    0   2
Phalaenopsis-gene694121               1    0    1    1    0   3
Phalaenopsis-gene704930               1    0    0    1    0   2
Phalaenopsis-gene734462               1    1    1    1    1   5
Phalaenopsis-gene763717               1    1    1    1    1   5
Phalaenopsis-gene84823                1    1    1    1    1   5
Phalaenopsis-gene85149                0    0    0    1    0   1
Phalaenopsis-gene903704               1    0    0    1    0   2
Phalaenopsis-gene97149                0    0    0    1    0   1
Phalaenopsis-gene22928                1    0    0    0    0   1
Phalaenopsis-gene259031               1    0    0    1    0   2
Phalaenopsis-gene340312               1    0    0    1    0   2
Phalaenopsis-gene505450               1    1    1    1    1   5
Phalaenopsis-gene652186               1    0    0    0    0   1
Phalaenopsis-gene229626               1    0    1    1    0   3
Phalaenopsis-gene322281               1    0    0    0    1   2
Phalaenopsis-gene322323               1    1    0    0    0   2
Phalaenopsis-gene742476               1    0    0    1    1   3
Phalaenopsis-gene156936               1    1    1    1    1   5
Phalaenopsis-gene349279               1    1    1    1    1   5
Phalaenopsis-gene378879               0    0    1    0    0   1
Phalaenopsis-gene471604               1    1    0    1    0   3
Phalaenopsis-gene547245               0    0    0    1    0   1
Phalaenopsis-gene741157               1    1    1    1    1   5
Phalaenopsis-gene786500               0    0    1    0    0   1
Phalaenopsis-gene150188               1    0    1    1    0   3
Phalaenopsis-gene150346               1    0    1    1    0   3
Phalaenopsis-gene244734               1    0    0    1    0   2
Phalaenopsis-gene281470               1    1    1    1    1   5
Phalaenopsis-gene284034               1    0    1    1    0   3
Phalaenopsis-gene306770               1    1    1    1    1   5
Phalaenopsis-gene309592               1    1    1    1    1   5
Phalaenopsis-gene310207               1    0    1    1    0   3
Phalaenopsis-gene332836               1    0    1    0    0   2
Phalaenopsis-gene338640               1    0    1    1    0   3
Phalaenopsis-gene349680               1    1    1    1    1   5
Phalaenopsis-gene350457               1    0    1    1    0   3
Phalaenopsis-gene353045               1    1    1    1    1   5
Phalaenopsis-gene376119               1    0    0    0    0   1
Phalaenopsis-gene47649                1    1    1    1    1   5
Phalaenopsis-gene520601               1    0    1    1    0   3
Phalaenopsis-gene530312               1    1    1    1    0   4
Phalaenopsis-gene563146               1    0    0    1    0   2
Phalaenopsis-gene566003               1    0    0    1    0   2
Phalaenopsis-gene60882                1    0    1    1    0   3
Phalaenopsis-gene624139               1    0    1    1    0   3
Phalaenopsis-gene640361               1    0    0    1    0   2
Phalaenopsis-gene662896               0    0    1    1    0   2
Phalaenopsis-gene717906               1    0    0    1    0   2
Phalaenopsis-gene74207                1    0    1    1    0   3
Phalaenopsis-gene79022                1    0    1    0    0   2
Phalaenopsis-gene852532               1    1    1    1    1   5
Phalaenopsis-gene852563               1    1    1    1    1   5
Phalaenopsis-gene149867               0    0    0    1    0   1
Phalaenopsis-gene149943               0    0    0    1    0   1
Phalaenopsis-gene172907               1    1    1    1    1   5
Phalaenopsis-gene22987                0    0    0    1    0   1
Phalaenopsis-gene257447               0    0    1    1    0   2
Phalaenopsis-gene258781               1    0    1    1    0   3
Phalaenopsis-gene340038               0    0    0    1    0   1
Phalaenopsis-gene341994               0    0    0    0    0   0
Phalaenopsis-gene364321               1    1    0    1    0   3
Phalaenopsis-gene36640                1    0    0    1    0   2
Phalaenopsis-gene36664                1    1    1    1    0   4
Phalaenopsis-gene385331               1    0    0    1    0   2
Phalaenopsis-gene409426               1    0    0    1    1   3
Phalaenopsis-gene413916               1    0    0    1    0   2
Phalaenopsis-gene437628               1    0    0    0    0   1
Phalaenopsis-gene447229               1    1    1    1    1   5
Phalaenopsis-gene458670               1    0    0    1    0   2
Phalaenopsis-gene482171               1    0    0    1    0   2
Phalaenopsis-gene499356               1    1    1    1    1   5
Phalaenopsis-gene504361               1    1    1    1    1   5
Phalaenopsis-gene51680                1    1    1    1    1   5
Phalaenopsis-gene524294               1    1    1    1    1   5
Phalaenopsis-gene526733               1    0    1    1    0   3
Phalaenopsis-gene539560               1    1    1    1    1   5
Phalaenopsis-gene575602               0    0    0    1    0   1
Phalaenopsis-gene585959               1    1    0    1    0   3
Phalaenopsis-gene593321               1    1    1    1    1   5
Phalaenopsis-gene605252               1    1    1    1    1   5
Phalaenopsis-gene636668               0    0    1    1    0   2
Phalaenopsis-gene682143               1    1    1    1    1   5
Phalaenopsis-gene689771               1    1    1    1    1   5
Phalaenopsis-gene708131               1    1    1    1    1   5
Phalaenopsis-gene709947               1    1    1    1    1   5
Phalaenopsis-gene725472               1    0    0    0    0   1
Phalaenopsis-gene794964               1    1    0    1    0   3
Phalaenopsis-gene797177               1    0    0    1    0   2
Phalaenopsis-gene806945               1    1    1    1    1   5
Phalaenopsis-gene815665               1    0    0    1    0   2
Phalaenopsis-gene815720               1    0    0    0    0   1
Phalaenopsis-gene149896               0    0    1    1    0   2
Phalaenopsis-gene155732               1    0    0    1    0   2
Phalaenopsis-gene322391               1    1    1    1    0   4
Phalaenopsis-gene364325               1    1    0    0    0   2
Phalaenopsis-gene470914               1    0    1    1    0   3
Phalaenopsis-gene628582               1    1    1    1    0   4
Phalaenopsis-gene643305               1    1    1    1    1   5
Phalaenopsis-gene716037               1    0    1    1    0   3
Phalaenopsis-gene744466               1    1    1    1    1   5
Phalaenopsis-gene840876               0    0    1    0    0   1
Phalaenopsis-gene856701               1    0    1    1    0   3
Phalaenopsis-gene867488               1    0    1    0    0   2
Phalaenopsis-gene888256               1    0    1    1    0   3
Phalaenopsis-gene89214                0    1    0    1    0   2
Phalaenopsis-gene122898               1    0    1    0    0   2
Phalaenopsis-gene347334               0    0    0    1    0   1
Phalaenopsis-gene504544               1    1    0    1    0   3
Phalaenopsis-gene504614               1    0    0    1    0   2
Phalaenopsis-gene504638               0    0    0    1    0   1
Phalaenopsis-gene685777               1    1    1    1    0   4
Phalaenopsis-gene851453               0    0    1    1    0   2
Phalaenopsis-gene250493               1    1    1    1    1   5
Phalaenopsis-gene338569               0    0    1    1    0   2
Phalaenopsis-gene381434               1    1    0    1    0   3
Phalaenopsis-gene409454               1    0    0    1    0   2
Phalaenopsis-gene439365               1    0    0    0    0   1
Phalaenopsis-gene526372               1    0    1    1    0   3
Phalaenopsis-gene158874               1    0    1    0    0   2
Phalaenopsis-gene209244               1    0    0    0    0   1
Phalaenopsis-gene209309               0    0    0    1    0   1
Phalaenopsis-gene305331               1    0    1    1    0   3
Phalaenopsis-gene351241               0    0    1    1    0   2
Phalaenopsis-gene419167               0    0    1    1    0   2
Phalaenopsis-gene419226               0    0    1    0    0   1
Phalaenopsis-gene419256               1    0    0    1    0   2
Phalaenopsis-gene419285               1    0    1    1    0   3
Phalaenopsis-gene621285               1    0    0    1    0   2
Phalaenopsis-gene694756               1    1    1    1    1   5
Phalaenopsis-gene141724               1    1    0    0    0   2
Phalaenopsis-gene276801               1    1    1    1    1   5
Phalaenopsis-gene280877               1    1    1    1    1   5
Phalaenopsis-gene317706               1    0    0    1    0   2
Phalaenopsis-gene364350               1    0    0    0    0   1
Phalaenopsis-gene405347               1    0    1    0    0   2
Phalaenopsis-gene433689               1    1    1    1    0   4
Phalaenopsis-gene469149               0    0    1    0    0   1
Phalaenopsis-gene571971               1    0    1    1    0   3
Phalaenopsis-gene573422               1    0    1    0    0   2
Phalaenopsis-gene621406               1    0    1    1    0   3
Phalaenopsis-gene6296                 1    1    1    1    1   5
Phalaenopsis-gene658812               1    0    1    1    0   3
Phalaenopsis-gene772701               0    0    0    1    0   1
Phalaenopsis-gene815137               1    1    1    1    1   5
Phalaenopsis-gene36585                1    1    0    1    0   3
Phalaenopsis-gene475244               1    0    1    1    0   3
Phalaenopsis-gene495512               1    0    0    1    0   2
Phalaenopsis-gene723987               1    1    0    1    0   3
Phalaenopsis-gene146105               1    0    1    1    1   4
Phalaenopsis-gene22711                1    1    1    1    1   5
Phalaenopsis-gene322359               1    1    1    1    1   5
Phalaenopsis-gene340037               1    1    1    1    1   5
Phalaenopsis-gene413167               1    0    1    0    0   2
Phalaenopsis-gene482233               1    0    1    1    0   3
Phalaenopsis-gene488082               1    1    1    1    1   5
Phalaenopsis-gene641210               0    0    1    1    0   2
Phalaenopsis-gene767931               1    1    1    1    1   5
Phalaenopsis-gene904621               0    1    1    1    0   3
Phalaenopsis-gene96063                0    0    1    0    0   1
Phalaenopsis-gene107716               1    0    1    1    0   3
Phalaenopsis-gene405732               0    0    1    1    0   2
Phalaenopsis-gene460935               0    1    1    1    0   3
Phalaenopsis-gene460959               1    1    1    1    1   5
Phalaenopsis-gene548234               1    0    0    0    0   1
Phalaenopsis-gene570142               0    1    0    0    0   1
Phalaenopsis-gene276851               1    1    1    1    1   5
Phalaenopsis-gene326064               1    0    0    0    0   1
Phalaenopsis-gene351201               1    1    1    1    1   5
Phalaenopsis-gene363226               0    0    0    1    0   1
Phalaenopsis-gene38096                1    0    1    0    0   2
Phalaenopsis-gene486890               1    1    1    1    1   5
Phalaenopsis-gene529887               0    1    1    1    0   3
Phalaenopsis-gene645777               0    0    1    1    0   2
Phalaenopsis-gene645848               1    0    1    1    0   3
Phalaenopsis-gene675911               1    0    1    1    0   3
Phalaenopsis-gene688320               1    0    0    1    0   2
Phalaenopsis-gene728198               1    1    1    1    1   5
Phalaenopsis-gene767195               1    1    1    1    1   5
Phalaenopsis-gene839628               0    0    0    0    0   0
Phalaenopsis-gene839854               1    0    1    1    0   3
Ophrys-gene28                         1    1    1    1    1   5
Ophrys-gene2037                       1    0    0    1    0   2
Ophrys-gene2854                       1    0    1    1    0   3
Ophrys-gene4659                       0    0    1    0    0   1
Ophrys-gene4715                       1    1    1    1    1   5
Ophrys-gene5265                       1    0    0    0    0   1
Ophrys-gene5393                       1    0    1    0    0   2
Ophrys-gene5527                       1    1    1    1    1   5
Ophrys-gene5953                       1    1    1    1    1   5
Ophrys-gene7406                       1    0    0    0    0   1
Ophrys-gene7754                       1    0    0    1    0   2
Ophrys-gene8096                       1    1    1    1    1   5
Ophrys-gene8327                       1    0    0    0    0   1
Ophrys-gene8594                       1    1    1    1    1   5
Ophrys-gene8607                       1    0    1    0    0   2
Ophrys-gene8637                       1    1    1    1    1   5
Ophrys-gene9231                       1    0    0    0    0   1
Ophrys-gene9612                       1    1    1    1    1   5
Ophrys-gene9775                       1    1    1    1    1   5
Ophrys-gene10136                      1    0    1    0    0   2
Ophrys-gene10279                      1    1    1    1    1   5
Ophrys-gene11095                      1    1    1    1    1   5
Ophrys-gene11772                      0    0    1    1    0   2
Ophrys-gene12318                      1    1    1    1    1   5
Ophrys-gene20065                      1    1    1    1    1   5
Ophrys-gene20229                      1    1    1    1    1   5
Ophrys-gene20209                      1    0    1    1    0   3
Ophrys-gene20342                      1    0    0    0    0   1
Ophrys-gene21644                      0    0    0    1    0   1
Ophrys-gene23839                      1    0    1    1    0   3
Ophrys-gene24972                      1    1    1    1    1   5
Ophrys-gene38161                      0    0    1    1    0   2
Ophrys-gene47996                      1    1    1    1    1   5
Ophrys-gene48005                      1    1    1    1    1   5
Ophrys-gene48541                      1    1    1    1    1   5
Ophrys-gene51273                      0    0    0    1    0   1
Ophrys-gene54134                      0    1    0    1    0   2
Ophrys-gene56715                      1    1    1    1    1   5
Ophrys-gene57941                      0    1    0    1    0   2
Ophrys-gene58889                      1    0    0    0    0   1
Ophrys-gene59466                      1    1    1    1    1   5
Ophrys-gene59964                      1    0    1    1    0   3
Ophrys-gene60537                      0    0    0    1    0   1
Ophrys-gene61775                      1    0    0    0    0   1
Ophrys-gene62106                      1    0    0    0    0   1
Ophrys-gene63017                      1    0    1    1    0   3
Ophrys-gene67374                      1    1    1    1    1   5
Ophrys-gene67497                      1    0    0    1    0   2
Ophrys-gene68628                      1    1    0    0    0   2
Ophrys-gene72121                      1    0    0    0    0   1
Ophrys-gene73857                      1    1    0    1    0   3
Ophrys-gene75163                      1    0    0    1    0   2
Ophrys-gene76838                      1    0    0    1    0   2
Ophrys-gene80985                      1    1    1    1    1   5
Ophrys-gene81687                      1    1    1    1    1   5
Ophrys-gene82065                      1    1    1    1    1   5
Ophrys-gene85600                      0    1    1    1    1   4
Ophrys-gene87875                      1    0    0    0    0   1
Ophrys-gene92059                      0    0    0    0    0   0
Ophrys-gene94021                      1    1    1    1    1   5
Ophrys-gene96726                      1    0    0    1    0   2
Ophrys-gene97048                      1    1    0    0    0   2
Ophrys-gene99903                      1    0    0    1    0   2
Ophrys-gene99983                      1    1    1    1    1   5
Ophrys-gene102226                     1    0    0    1    0   2
Ophrys-gene102644                     1    0    1    1    0   3
Ophrys-gene103998                     1    0    0    1    0   2
Ophrys-gene104617                     1    1    1    1    1   5
Ophrys-gene105583                     1    0    0    1    0   2
Ophrys-gene113101                     1    0    0    1    0   2
Ophrys-gene116955                     0    0    0    1    0   1
Ophrys-gene118870                     1    1    1    1    1   5
Ophrys-gene118889                     0    0    0    1    0   1
Ophrys-gene118954                     1    0    1    1    0   3
Ophrys-gene120107                     0    0    0    1    0   1
Ophrys-gene120459                     1    1    1    1    1   5
Ophrys-gene124298                     1    1    1    1    1   5
Ophrys-gene132869                     1    0    1    1    0   3
Ophrys-gene132990                     0    0    1    0    0   1
Ophrys-gene134798                     0    1    0    1    1   3
Ophrys-gene134882                     1    0    1    1    0   3
Ophrys-gene136043                     1    0    0    1    0   2
Ophrys-gene136713                     1    0    1    1    0   3
Ophrys-gene136917                     1    1    1    1    1   5
Ophrys-gene137149                     1    1    1    1    1   5
Ophrys-gene139364                     1    0    1    0    0   2
Ophrys-gene140665                     1    0    1    0    0   2
Ophrys-gene140668                     1    1    1    1    1   5
Ophrys-gene141550                     0    0    1    1    0   2
Ophrys-gene144431                     1    1    1    1    1   5
Ophrys-gene145860                     0    0    0    1    0   1
Ophrys-gene147327                     1    1    1    1    1   5
Ophrys-gene149093                     1    0    0    1    0   2
Ophrys-gene149095                     1    0    0    1    0   2
Ophrys-gene149124                     1    0    1    1    0   3
Ophrys-gene149290                     1    0    0    1    0   2
Ophrys-gene152671                     1    0    0    1    0   2
Ophrys-gene157140                     1    0    0    0    0   1
Ophrys-gene158436                     1    0    1    0    0   2
Ophrys-gene159102                     1    1    1    1    1   5
Ophrys-gene159228                     1    1    1    1    1   5
Ophrys-gene166453                     1    1    1    1    1   5
Ophrys-gene166635                     1    1    1    1    1   5
Ophrys-gene168848                     1    1    1    1    1   5
Ophrys-gene169563                     1    1    1    1    0   4
Ophrys-gene173494                     1    0    0    1    0   2
Ophrys-gene173778                     1    0    1    1    0   3
Ophrys-gene174582                     1    1    1    1    1   5
Ophrys-gene174835                     1    1    1    1    1   5
Ophrys-gene176175                     1    1    1    1    1   5
Ophrys-gene177784                     1    1    1    1    1   5
Ophrys-gene178209                     1    1    1    1    1   5
Ophrys-gene180192                     1    0    0    1    0   2
Ophrys-gene180224                     1    1    1    1    1   5
Ophrys-gene180226                     1    0    1    1    0   3
Ophrys-gene180616                     1    0    0    1    0   2
Ophrys-gene181743                     1    1    1    1    1   5
Ophrys-gene181828                     1    1    0    0    0   2
Ophrys-gene182679                     1    0    0    0    0   1
Ophrys-gene182708                     1    1    1    1    1   5
Ophrys-gene183080                     1    0    0    0    0   1
Ophrys-gene184140                     0    0    0    1    0   1
Ophrys-gene184141                     1    0    0    1    0   2
Ophrys-gene184186                     0    0    0    0    0   0
Ophrys-gene187131                     1    0    1    0    0   2
Ophrys-gene194111                     1    1    0    1    0   3
Ophrys-gene194118                     1    1    1    1    1   5
Ophrys-gene194119                     1    1    1    1    1   5
Ophrys-gene203631                     1    0    0    1    0   2
Ophrys-gene205972                     1    1    1    1    1   5
Ophrys-gene206086                     0    0    0    1    0   1
Ophrys-gene207559                     1    1    1    1    1   5
Ophrys-gene208040                     1    0    0    1    0   2
Ophrys-gene210373                     1    1    1    1    1   5
Ophrys-gene210666                     1    0    0    1    0   2
Ophrys-gene210843                     1    0    0    0    0   1
Ophrys-gene213092                     0    0    0    1    0   1
Ophrys-gene215603                     1    0    0    0    0   1
Ophrys-gene219177                     1    0    0    1    0   2
Ophrys-gene219240                     1    0    0    1    0   2
Ophrys-gene222214                     1    1    1    1    1   5
Ophrys-gene222215                     1    0    0    1    0   2
Ophrys-gene224207                     0    0    1    0    0   1
Ophrys-gene226846                     1    1    1    1    1   5
Ophrys-gene238554                     1    0    0    1    0   2
Ophrys-gene238490                     1    1    0    1    0   3
Ophrys-gene240124                     0    0    0    1    0   1
Ophrys-gene240666                     1    0    0    1    1   3
Ophrys-gene243843                     1    1    1    1    1   5
Ophrys-gene246028                     1    1    1    1    1   5
Ophrys-gene247332                     0    0    1    1    0   2
Ophrys-gene251356                     1    1    0    1    0   3
Ophrys-gene252998                     1    1    1    1    1   5
Ophrys-gene254213                     1    1    1    1    1   5
Ophrys-gene258324                     1    1    1    1    1   5
Ophrys-gene258506                     0    0    0    0    0   0
Ophrys-gene263435                     0    0    0    0    0   0
Ophrys-gene265650                     1    1    1    1    1   5
Ophrys-gene268040                     1    1    1    1    1   5
Ophrys-gene270081                     0    0    0    0    0   0
Ophrys-gene271122                     0    0    1    0    0   1
Ophrys-gene272085                     1    1    1    1    0   4
Ophrys-gene272633                     1    0    0    1    0   2
Ophrys-gene272865                     0    0    0    1    0   1
Ophrys-gene273811                     1    0    0    1    0   2
Ophrys-gene273812                     1    0    0    1    0   2
Ophrys-gene274852                     1    1    1    1    1   5
Ophrys-gene277963                     1    1    1    1    1   5
Ophrys-gene278347                     1    1    1    1    1   5
Ophrys-gene280505                     1    1    1    1    1   5
Ophrys-gene281290                     1    1    1    1    1   5
Ophrys-gene282213                     1    1    1    1    1   5
Ophrys-gene282262                     1    0    1    0    0   2
Ophrys-gene286162                     1    1    1    1    1   5
Ophrys-gene288727                     1    0    1    1    0   3
Ophrys-gene288737                     1    1    1    1    1   5
Ophrys-gene289111                     1    1    1    1    1   5
Ophrys-gene289546                     1    0    0    1    0   2
Ophrys-gene291932                     1    1    1    1    1   5
Ophrys-gene292689                     1    0    1    1    0   3
Ophrys-gene292980                     1    1    1    1    1   5
Ophrys-gene293010                     1    1    1    1    1   5
Ophrys-gene293198                     1    0    1    1    0   3
Ophrys-gene293779                     0    1    1    1    0   3
Ophrys-gene293800                     1    0    0    0    0   1
Ophrys-gene294453                     1    0    0    0    0   1
Ophrys-gene295407                     1    0    0    1    0   2
Ophrys-gene295494                     1    1    1    1    1   5
Ophrys-gene295472                     0    0    0    1    0   1
Ophrys-gene295535                     1    1    1    1    1   5
Ophrys-gene295568                     1    0    0    0    0   1
Ophrys-gene296650                     1    1    1    1    1   5
Ophrys-gene298054                     1    1    1    1    1   5
Ophrys-gene298985                     1    0    0    1    0   2
Ophrys-gene299739                     0    0    1    1    0   2
Ophrys-gene305042                     1    0    0    1    0   2
Ophrys-gene305093                     1    0    1    1    0   3
Ophrys-gene318881                     1    0    1    1    0   3
Ophrys-gene324675                     0    0    0    1    0   1
Ophrys-gene324757                     1    1    1    1    1   5
Ophrys-gene331584                     1    1    1    1    1   5
Ophrys-gene337427                     1    1    1    1    1   5
Ophrys-gene339115                     1    0    0    1    0   2
Ophrys-gene339393                     1    0    1    1    0   3
Ophrys-gene341160                     1    1    1    1    1   5
Ophrys-gene341511                     1    1    1    1    1   5
Ophrys-gene342458                     1    1    1    1    1   5
Ophrys-gene343574                     1    1    1    1    1   5
Ophrys-gene346417                     1    1    1    1    1   5
Ophrys-gene351524                     1    1    1    1    1   5
Ophrys-gene352185                     0    1    1    1    0   3
Ophrys-gene358417                     0    0    0    1    1   2
Ophrys-gene359474                     0    0    1    0    0   1
Ophrys-gene365353                     0    0    0    0    0   0
Ophrys-gene365582                     1    0    0    1    0   2
Ophrys-gene367996                     1    1    1    1    1   5
Ophrys-gene370802                     1    1    1    1    1   5
Ophrys-gene375163                     1    1    1    1    1   5
Ophrys-gene380368                     0    0    0    1    0   1
Ophrys-gene382304                     1    0    1    0    0   2
Ophrys-gene384538                     1    0    0    0    0   1
Ophrys-gene385888                     1    0    0    1    0   2
Ophrys-gene386370                     1    0    1    1    0   3
Ophrys-gene386344                     1    1    0    0    0   2
Ophrys-gene386396                     1    1    1    1    1   5
Ophrys-gene386391                     1    0    0    1    0   2
Ophrys-gene390644                     1    0    1    1    0   3
Ophrys-gene392595                     1    0    1    1    0   3
Ophrys-gene392629                     1    0    0    1    0   2
Ophrys-gene393165                     1    1    1    1    1   5
Ophrys-gene395570                     1    1    1    1    1   5
Ophrys-gene395664                     1    1    1    1    1   5
Ophrys-gene395725                     1    1    1    1    1   5
Ophrys-gene398929                     1    1    1    1    1   5
Ophrys-gene399432                     0    0    0    0    0   0
Ophrys-gene399451                     1    1    1    1    1   5
Ophrys-gene404130                     1    0    0    0    0   1
Ophrys-gene409489                     1    1    1    1    1   5
Ophrys-gene413918                     1    1    1    1    1   5
Ophrys-gene413962                     1    0    0    1    0   2
Ophrys-gene416894                     1    1    1    1    1   5
Ophrys-gene419483                     1    1    1    1    1   5
Ophrys-gene425097                     1    1    1    1    1   5
Ophrys-gene425281                     1    0    0    1    0   2
Ophrys-gene427705                     1    0    0    0    0   1
Ophrys-gene427708                     1    0    1    0    0   2
Ophrys-gene428946                     1    1    1    1    1   5
Ophrys-gene428994                     1    0    0    1    0   2
Ophrys-gene430224                     1    0    0    0    0   1
Ophrys-gene430218                     1    1    1    1    1   5
Ophrys-gene431781                     1    0    0    1    0   2
Ophrys-gene432213                     1    1    0    1    0   3
Ophrys-gene432605                     1    0    0    1    0   2
Ophrys-gene434967                     1    1    1    1    1   5
Ophrys-gene435142                     1    0    0    1    0   2
Ophrys-gene440793                     1    0    1    1    0   3
Ophrys-gene441049                     1    0    0    1    0   2
Ophrys-gene444686                     1    0    0    1    0   2
Ophrys-gene444791                     1    0    0    1    0   2
Ophrys-gene444844                     1    0    0    0    0   1
Ophrys-gene445099                     0    0    0    1    0   1
Ophrys-gene445149                     0    1    0    0    1   2
Ophrys-gene446252                     1    1    1    1    1   5
Ophrys-gene446233                     1    1    1    1    1   5
Ophrys-gene447275                     1    1    1    1    1   5
Ophrys-gene447337                     1    0    1    0    0   2
Ophrys-gene449254                     0    0    0    0    0   0
Ophrys-gene449889                     1    1    1    1    1   5
Ophrys-gene451815                     1    0    1    1    0   3
Ophrys-gene458080                     1    0    0    0    0   1
Ophrys-gene460121                     1    1    1    1    1   5
Ophrys-gene464239                     1    1    1    1    1   5
Ophrys-gene467394                     1    1    1    1    1   5
Ophrys-gene469740                     0    0    0    1    0   1
Ophrys-gene470315                     1    1    1    1    1   5
Ophrys-gene470310                     1    1    1    1    1   5
Ophrys-gene474611                     0    0    1    1    0   2
Ophrys-gene475285                     1    1    1    1    1   5
Ophrys-gene477487                     1    1    1    1    1   5
Ophrys-gene479131                     1    0    0    1    0   2
Ophrys-gene481226                     1    1    1    1    0   4
Ophrys-gene488600                     0    0    0    1    0   1
Ophrys-gene488705                     0    0    1    1    0   2
Ophrys-gene490062                     1    1    0    1    0   3
Ophrys-gene491438                     1    1    0    0    0   2
Ophrys-gene492790                     1    1    1    1    1   5
Ophrys-gene492791                     1    0    0    1    0   2
Ophrys-gene493112                     1    1    1    1    1   5
Ophrys-gene495171                     1    0    0    0    0   1
Ophrys-gene495172                     1    0    0    0    0   1
Ophrys-gene495238                     1    0    0    1    0   2
Ophrys-gene495301                     1    0    0    0    0   1
Ophrys-gene495302                     1    0    0    1    0   2
Ophrys-gene495363                     1    0    0    0    0   1
Ophrys-gene495433                     1    0    0    0    0   1
Ophrys-gene495598                     1    0    0    1    0   2
Ophrys-gene495663                     1    0    0    0    0   1
Ophrys-gene495720                     1    0    0    1    0   2
Ophrys-gene495933                     1    1    0    1    0   3
Ophrys-gene496288                     0    0    0    0    0   0
Ophrys-gene496289                     1    0    1    1    0   3
Ophrys-gene496387                     1    0    0    0    0   1
Ophrys-gene499065                     1    1    1    1    1   5
Ophrys-gene499782                     1    1    1    1    1   5
Ophrys-gene499784                     0    1    0    1    0   2
Ophrys-gene505093                     1    1    1    1    1   5
Ophrys-gene505097                     0    0    1    1    0   2
Ophrys-gene506609                     0    0    1    1    0   2
Ophrys-gene508457                     1    0    0    0    0   1
Ophrys-gene508487                     1    1    1    1    1   5
Ophrys-gene508468                     1    0    0    0    0   1
Ophrys-gene509217                     1    1    1    1    1   5
Ophrys-gene511080                     1    1    1    1    1   5
Ophrys-gene511150                     1    0    0    1    0   2
Ophrys-gene511195                     0    0    0    0    0   0
Ophrys-gene511191                     1    0    1    0    0   2
Ophrys-gene511799                     1    1    1    1    1   5
Ophrys-gene511952                     1    0    0    1    1   3
Ophrys-gene514642                     0    0    0    1    0   1
Ophrys-gene514737                     1    0    0    0    0   1
Ophrys-gene514826                     1    1    1    1    1   5
Ophrys-gene515091                     1    0    0    0    0   1
Ophrys-gene518808                     0    0    1    0    0   1
Ophrys-gene525664                     1    0    1    1    0   3
Ophrys-gene525812                     0    0    1    1    0   2
Ophrys-gene525914                     1    0    1    1    0   3
Ophrys-gene526021                     1    0    0    0    0   1
Ophrys-gene526318                     1    0    0    1    0   2
Ophrys-gene536834                     1    0    0    0    0   1
Ophrys-gene542820                     1    1    1    1    1   5
Ophrys-gene542931                     1    0    1    1    0   3
Ophrys-gene543951                     1    1    1    1    1   5
Ophrys-gene543967                     1    1    1    1    1   5
Ophrys-gene543981                     1    0    0    0    1   2
Ophrys-gene549815                     1    0    0    1    0   2
Ophrys-gene549804                     1    0    1    0    0   2
Ophrys-gene552143                     1    0    0    1    0   2
Ophrys-gene552146                     1    0    0    0    0   1
Ophrys-gene553971                     0    0    1    0    0   1
Ophrys-gene554228                     1    1    1    1    1   5
Ophrys-gene560313                     0    0    1    0    0   1
Ophrys-gene563052                     1    0    1    1    0   3
Ophrys-gene564450                     0    0    1    1    0   2
Ophrys-gene564565                     0    0    0    1    0   1
Ophrys-gene564545                     1    0    0    0    0   1
Ophrys-gene564546                     1    0    1    1    0   3
Ophrys-gene565171                     1    1    1    1    1   5
Ophrys-gene565191                     1    0    1    1    0   3
Ophrys-gene565913                     1    0    1    1    0   3
Ophrys-gene565946                     1    1    1    1    1   5
Ophrys-gene566201                     0    1    0    1    0   2
Ophrys-gene566335                     1    0    0    1    0   2
Ophrys-gene566346                     1    0    1    1    0   3
Ophrys-gene566467                     0    1    1    1    0   3
Ophrys-gene568044                     1    1    1    1    1   5
Ophrys-gene568672                     0    0    0    1    0   1
Ophrys-gene569473                     1    0    0    1    0   2
Ophrys-gene583214                     1    0    0    0    0   1
Ophrys-gene586443                     1    0    0    0    0   1
Ophrys-gene587276                     1    1    1    1    1   5
Ophrys-gene587284                     1    1    1    1    1   5
Ophrys-gene590113                     1    1    1    1    1   5
Ophrys-gene591134                     0    0    0    0    0   0
Ophrys-gene591114                     1    1    1    1    1   5
Ophrys-gene592675                     1    0    1    1    0   3
Ophrys-gene595641                     0    0    1    1    0   2
Ophrys-gene597723                     0    0    1    0    0   1
Ophrys-gene601270                     1    0    0    1    0   2
Ophrys-gene601271                     1    0    0    1    0   2
Ophrys-gene603348                     1    1    1    1    1   5
Ophrys-gene605830                     1    1    1    1    1   5
Ophrys-gene606512                     1    0    0    1    0   2
Ophrys-gene608032                     1    1    1    1    1   5
Ophrys-gene609120                     1    1    1    1    1   5
Ophrys-gene609323                     1    1    1    1    1   5
Ophrys-gene609386                     1    0    0    1    0   2
Ophrys-gene609848                     1    0    1    1    0   3
Ophrys-gene609849                     1    1    1    1    1   5
Ophrys-gene610214                     1    0    1    1    0   3
Ophrys-gene610390                     1    1    1    1    1   5
Ophrys-gene612097                     1    0    0    1    0   2
Ophrys-gene613535                     1    0    0    1    0   2
Ophrys-gene613825                     1    0    0    1    0   2
Ophrys-gene616637                     1    0    1    1    0   3
Ophrys-gene616685                     1    0    0    0    0   1
Ophrys-gene617149                     0    0    1    1    0   2
Ophrys-gene617297                     1    1    1    1    1   5
Ophrys-gene617307                     1    1    1    1    1   5
Ophrys-gene617311                     1    0    0    1    0   2
Ophrys-gene618426                     1    0    1    1    0   3
Ophrys-gene619122                     1    1    1    1    1   5
Ophrys-gene619123                     1    0    0    1    0   2
Ophrys-gene625919                     1    1    1    1    1   5
Ophrys-gene632788                     0    1    1    1    0   3
Ophrys-gene638850                     1    1    1    1    1   5
Ophrys-gene639907                     1    1    1    1    1   5
Ophrys-gene639909                     1    0    0    1    0   2
Ophrys-gene645127                     1    1    1    1    1   5
Ophrys-gene646763                     1    1    1    1    1   5
Ophrys-gene649378                     1    0    1    1    0   3
Ophrys-gene650962                     1    0    0    1    0   2
Ophrys-gene654366                     1    0    1    1    0   3
Ophrys-gene661408                     1    0    1    1    0   3
Ophrys-gene663847                     1    0    0    0    0   1
Ophrys-gene665538                     1    1    1    1    1   5
Ophrys-gene679574                     1    1    1    1    1   5
Ophrys-gene680819                     1    0    0    1    0   2
Ophrys-gene681948                     1    0    0    0    0   1
Ophrys-gene683247                     0    1    0    1    0   2
Ophrys-gene685355                     0    0    1    1    0   2
Ophrys-gene687427                     1    1    0    1    0   3
Ophrys-gene689692                     1    1    1    1    1   5
Ophrys-gene690172                     0    0    0    1    0   1
Ophrys-gene690204                     1    1    1    1    1   5
Ophrys-gene691161                     1    1    1    1    1   5
Ophrys-gene692917                     0    1    1    0    0   2
Ophrys-gene693181                     1    1    1    1    1   5
Ophrys-gene693540                     1    1    1    1    1   5
Ophrys-gene695747                     0    0    1    0    0   1
Ophrys-gene698634                     0    0    0    1    0   1
Ophrys-gene702034                     1    1    1    1    1   5
Ophrys-gene702176                     1    1    1    1    1   5
Ophrys-gene702657                     1    0    0    1    0   2
Ophrys-gene702661                     1    1    1    1    1   5
Ophrys-gene707990                     1    1    1    1    1   5
Ophrys-gene709085                     1    1    1    1    1   5
Ophrys-gene709216                     1    0    0    1    0   2
Ophrys-gene709644                     1    0    1    1    0   3
Ophrys-gene711649                     1    0    1    1    0   3
Ophrys-gene713587                     1    0    1    1    0   3
Ophrys-gene715328                     0    0    1    1    0   2
Ophrys-gene716457                     1    1    1    1    1   5
Ophrys-gene717301                     1    1    1    1    1   5
Ophrys-gene717354                     1    0    0    1    0   2
Ophrys-gene722360                     1    0    0    1    0   2
Ophrys-gene722777                     1    0    1    1    0   3
Ophrys-gene726168                     1    0    0    1    0   2
Ophrys-gene731305                     1    0    1    0    0   2
Ophrys-gene731484                     1    0    0    1    0   2
Ophrys-gene731616                     1    0    0    1    0   2
Ophrys-gene731822                     0    0    1    1    0   2
Ophrys-gene731983                     0    0    0    1    0   1
Ophrys-gene742035                     1    1    1    1    1   5
Ophrys-gene746812                     1    1    1    1    1   5
Ophrys-gene746815                     1    1    1    1    1   5
Ophrys-gene747977                     1    1    1    1    1   5
Ophrys-gene753625                     0    0    0    1    0   1
Ophrys-gene756317                     1    0    1    0    0   2
Ophrys-gene757508                     1    0    0    1    0   2
Ophrys-gene758895                     1    0    0    1    0   2
Ophrys-gene762874                     1    0    1    1    0   3
Ophrys-gene762993                     1    0    1    1    0   3
Ophrys-gene763266                     1    1    1    1    1   5
Ophrys-gene764480                     1    0    0    0    0   1
Ophrys-gene765626                     1    1    1    1    1   5
Ophrys-gene769393                     1    1    1    1    1   5
Phalaenopsis-gene106761               1    0    0    1    0   2
Phalaenopsis-gene11232                1    1    1    1    1   5
Phalaenopsis-gene120527               1    0    0    1    0   2
Phalaenopsis-gene120548               1    0    1    1    1   4
Phalaenopsis-gene125021               1    1    1    1    1   5
Phalaenopsis-gene125026               1    0    0    1    0   2
Phalaenopsis-gene1259                 1    1    1    1    1   5
Phalaenopsis-gene129432               1    0    1    1    0   3
Phalaenopsis-gene130043               1    1    1    1    1   5
Phalaenopsis-gene131303               1    1    1    1    1   5
Phalaenopsis-gene131334               1    1    1    1    1   5
Phalaenopsis-gene133126               1    1    1    1    1   5
Phalaenopsis-gene136100               1    0    0    0    0   1
Phalaenopsis-gene138420               0    0    1    1    0   2
Phalaenopsis-gene140100               1    1    1    1    1   5
Phalaenopsis-gene143487               1    1    1    1    1   5
Phalaenopsis-gene146106               1    1    1    1    1   5
Phalaenopsis-gene149868               1    1    1    1    1   5
Phalaenopsis-gene149997               1    0    0    1    0   2
Phalaenopsis-gene149998               1    0    0    1    0   2
Phalaenopsis-gene150080               0    0    1    1    0   2
Phalaenopsis-gene150153               1    1    1    1    0   4
Phalaenopsis-gene150229               1    1    1    1    1   5
Phalaenopsis-gene150302               1    0    1    1    0   3
Phalaenopsis-gene150936               1    0    1    0    0   2
Phalaenopsis-gene151720               1    0    1    1    0   3
Phalaenopsis-gene156146               1    0    1    1    0   3
Phalaenopsis-gene156952               1    1    1    1    1   5
Phalaenopsis-gene157336               1    0    1    1    0   3
Phalaenopsis-gene163314               1    1    0    1    0   3
Phalaenopsis-gene163423               1    0    1    0    0   2
Phalaenopsis-gene164222               1    1    1    1    1   5
Phalaenopsis-gene166762               1    0    1    1    0   3
Phalaenopsis-gene166824               1    0    1    0    0   2
Phalaenopsis-gene169226               1    0    1    0    0   2
Phalaenopsis-gene171935               0    0    1    0    0   1
Phalaenopsis-gene1800                 1    0    0    1    0   2
Phalaenopsis-gene183186               1    0    1    0    1   3
Phalaenopsis-gene187963               1    1    1    1    1   5
Phalaenopsis-gene189055               1    1    1    1    1   5
Phalaenopsis-gene189087               0    0    1    0    0   1
Phalaenopsis-gene189121               1    0    0    0    0   1
Phalaenopsis-gene194628               1    1    1    1    1   5
Phalaenopsis-gene197934               0    0    0    1    0   1
Phalaenopsis-gene198541               1    0    0    0    0   1
Phalaenopsis-gene202325               1    0    1    0    0   2
Phalaenopsis-gene203095               1    1    1    1    0   4
Phalaenopsis-gene209213               1    1    1    1    1   5
Phalaenopsis-gene214689               0    0    1    1    0   2
Phalaenopsis-gene214734               0    0    1    1    0   2
Phalaenopsis-gene216890               1    1    1    1    1   5
Phalaenopsis-gene216891               0    1    0    1    0   2
Phalaenopsis-gene216915               1    0    0    1    0   2
Phalaenopsis-gene217416               1    1    1    1    1   5
Phalaenopsis-gene22123                1    0    1    1    0   3
Phalaenopsis-gene22175                1    0    0    1    0   2
Phalaenopsis-gene222050               1    0    0    0    0   1
Phalaenopsis-gene223203               1    0    1    1    0   3
Phalaenopsis-gene228736               1    1    1    1    1   5
Phalaenopsis-gene229640               1    0    1    1    0   3
Phalaenopsis-gene230011               1    1    1    1    1   5
Phalaenopsis-gene233156               1    0    0    1    0   2
Phalaenopsis-gene233193               1    1    1    1    1   5
Phalaenopsis-gene233205               1    0    1    0    0   2
Phalaenopsis-gene234930               1    1    1    1    1   5
Phalaenopsis-gene234969               0    0    0    1    0   1
Phalaenopsis-gene23681                1    0    1    1    0   3
Phalaenopsis-gene242891               1    1    1    1    1   5
Phalaenopsis-gene244683               0    0    0    1    0   1
Phalaenopsis-gene247275               0    0    0    1    0   1
Phalaenopsis-gene250367               1    1    1    1    1   5
Phalaenopsis-gene251219               1    0    1    1    0   3
Phalaenopsis-gene251571               0    0    0    1    0   1
Phalaenopsis-gene252346               1    0    1    1    0   3
Phalaenopsis-gene252369               1    0    0    1    0   2
Phalaenopsis-gene254648               0    0    1    1    0   2
Phalaenopsis-gene254654               0    0    1    1    0   2
Phalaenopsis-gene254674               1    1    1    0    0   3
Phalaenopsis-gene254701               1    0    1    1    0   3
Phalaenopsis-gene254702               1    0    1    1    0   3
Phalaenopsis-gene25480                1    1    1    1    1   5
Phalaenopsis-gene25583                1    0    1    1    0   3
Phalaenopsis-gene258419               1    0    1    0    0   2
Phalaenopsis-gene258575               1    0    1    0    0   2
Phalaenopsis-gene260032               0    0    1    0    0   1
Phalaenopsis-gene266997               0    0    0    1    0   1
Phalaenopsis-gene269460               1    0    1    1    0   3
Phalaenopsis-gene276232               1    1    1    1    1   5
Phalaenopsis-gene281362               1    0    1    1    0   3
Phalaenopsis-gene281385               0    0    1    1    0   2
Phalaenopsis-gene281871               1    1    1    1    1   5
Phalaenopsis-gene282156               0    0    0    1    0   1
Phalaenopsis-gene28446                1    0    1    1    0   3
Phalaenopsis-gene28710                1    0    1    1    0   3
Phalaenopsis-gene287294               1    0    1    0    0   2
Phalaenopsis-gene287348               1    0    1    0    0   2
Phalaenopsis-gene290563               1    0    0    1    0   2
Phalaenopsis-gene291303               1    0    1    1    0   3
Phalaenopsis-gene29354                1    1    1    1    1   5
Phalaenopsis-gene296616               0    0    1    1    0   2
Phalaenopsis-gene297284               1    1    1    1    1   5
Phalaenopsis-gene298416               1    1    1    1    0   4
Phalaenopsis-gene302392               1    0    1    1    0   3
Phalaenopsis-gene303211               1    1    1    0    0   3
Phalaenopsis-gene306661               1    1    1    1    1   5
Phalaenopsis-gene309660               1    0    1    1    0   3
Phalaenopsis-gene309930               1    0    1    1    0   3
Phalaenopsis-gene309936               0    0    1    0    0   1
Phalaenopsis-gene312197               1    0    1    0    0   2
Phalaenopsis-gene318888               1    1    1    0    0   3
Phalaenopsis-gene322623               1    1    1    1    1   5
Phalaenopsis-gene322867               1    0    0    1    0   2
Phalaenopsis-gene324324               0    0    1    1    0   2
Phalaenopsis-gene324556               0    0    1    1    0   2
Phalaenopsis-gene324589               1    0    1    1    0   3
Phalaenopsis-gene332785               1    1    1    1    1   5
Phalaenopsis-gene340025               0    1    1    0    0   2
Phalaenopsis-gene340092               1    0    1    0    0   2
Phalaenopsis-gene340267               1    1    1    1    1   5
Phalaenopsis-gene340331               1    1    1    1    1   5
Phalaenopsis-gene340525               1    0    1    0    0   2
Phalaenopsis-gene341256               1    1    1    1    1   5
Phalaenopsis-gene342816               1    0    0    1    0   2
Phalaenopsis-gene34318                1    1    1    0    0   3
Phalaenopsis-gene343530               0    0    0    0    0   0
Phalaenopsis-gene344771               0    0    0    1    0   1
Phalaenopsis-gene345102               0    0    1    1    0   2
Phalaenopsis-gene347356               1    1    0    1    0   3
Phalaenopsis-gene348501               1    0    0    1    0   2
Phalaenopsis-gene348548               1    1    1    1    1   5
Phalaenopsis-gene349222               1    0    0    0    0   1
Phalaenopsis-gene349380               1    0    1    0    0   2
Phalaenopsis-gene350813               1    1    1    1    1   5
Phalaenopsis-gene355224               0    0    0    1    0   1
Phalaenopsis-gene362136               1    0    1    0    0   2
Phalaenopsis-gene362880               1    1    1    1    1   5
Phalaenopsis-gene365818               1    0    0    1    0   2
Phalaenopsis-gene36690                1    1    1    1    1   5
Phalaenopsis-gene36761                1    0    0    1    0   2
Phalaenopsis-gene36786                1    0    0    1    0   2
Phalaenopsis-gene370132               0    1    1    1    0   3
Phalaenopsis-gene371863               1    1    0    1    0   3
Phalaenopsis-gene371943               1    0    0    0    1   2
Phalaenopsis-gene371985               1    0    0    1    0   2
Phalaenopsis-gene37272                1    1    0    0    0   2
Phalaenopsis-gene375285               1    0    1    1    0   3
Phalaenopsis-gene38111                1    1    1    1    1   5
Phalaenopsis-gene381659               1    0    1    1    0   3
Phalaenopsis-gene384540               1    1    1    1    1   5
Phalaenopsis-gene388066               1    1    1    1    1   5
Phalaenopsis-gene388641               1    0    0    1    0   2
Phalaenopsis-gene391163               0    1    1    1    0   3
Phalaenopsis-gene393768               1    1    0    1    0   3
Phalaenopsis-gene393785               1    0    1    0    0   2
Phalaenopsis-gene393862               0    0    0    0    0   0
Phalaenopsis-gene393879               1    1    0    1    0   3
Phalaenopsis-gene397646               1    0    0    0    0   1
Phalaenopsis-gene397740               1    0    1    0    0   2
Phalaenopsis-gene400452               1    0    1    1    0   3
Phalaenopsis-gene400839               1    0    1    1    0   3
Phalaenopsis-gene402726               1    0    0    1    0   2
Phalaenopsis-gene405069               1    0    0    1    0   2
Phalaenopsis-gene405350               1    1    1    1    1   5
Phalaenopsis-gene409842               1    1    1    1    1   5
Phalaenopsis-gene409873               1    1    1    1    1   5
Phalaenopsis-gene409992               1    1    1    1    1   5
Phalaenopsis-gene410158               0    0    1    1    0   2
Phalaenopsis-gene410962               1    0    1    1    1   4
Phalaenopsis-gene412716               1    0    0    1    0   2
Phalaenopsis-gene412742               1    0    0    1    0   2
Phalaenopsis-gene413705               0    0    1    1    0   2
Phalaenopsis-gene421995               1    1    1    1    1   5
Phalaenopsis-gene425655               0    1    1    1    0   3
Phalaenopsis-gene429912               0    0    0    0    0   0
Phalaenopsis-gene430809               1    0    0    1    0   2
Phalaenopsis-gene431419               1    1    1    1    1   5
Phalaenopsis-gene431479               1    1    1    0    0   3
Phalaenopsis-gene433664               1    1    1    1    1   5
Phalaenopsis-gene433672               1    1    1    1    1   5
Phalaenopsis-gene437872               1    0    1    1    0   3
Phalaenopsis-gene44218                1    1    1    1    1   5
Phalaenopsis-gene44309                1    1    0    1    0   3
Phalaenopsis-gene447181               0    0    0    1    0   1
Phalaenopsis-gene447227               1    0    0    0    0   1
Phalaenopsis-gene447272               1    1    1    0    0   3
Phalaenopsis-gene448119               1    1    1    1    1   5
Phalaenopsis-gene448502               1    0    1    1    0   3
Phalaenopsis-gene455636               1    0    1    1    0   3
Phalaenopsis-gene455840               1    1    1    1    1   5
Phalaenopsis-gene456683               1    0    0    1    0   2
Phalaenopsis-gene456981               1    1    1    1    1   5
Phalaenopsis-gene457322               1    1    1    1    1   5
Phalaenopsis-gene457337               0    0    1    1    0   2
Phalaenopsis-gene460194               0    0    0    1    1   2
Phalaenopsis-gene460228               1    0    0    1    0   2
Phalaenopsis-gene460485               1    0    1    1    0   3
Phalaenopsis-gene465982               1    0    1    1    0   3
Phalaenopsis-gene467114               1    1    1    1    1   5
Phalaenopsis-gene467790               1    1    1    1    1   5
Phalaenopsis-gene467945               1    0    0    1    0   2
Phalaenopsis-gene471141               1    0    1    1    0   3
Phalaenopsis-gene471603               1    0    1    1    0   3
Phalaenopsis-gene482162               1    1    1    1    1   5
Phalaenopsis-gene485488               1    0    1    1    0   3
Phalaenopsis-gene485628               0    0    0    1    0   1
Phalaenopsis-gene485711               1    0    0    1    0   2
Phalaenopsis-gene485876               1    0    0    1    0   2
Phalaenopsis-gene486068               1    1    1    1    1   5
Phalaenopsis-gene486201               1    0    1    0    0   2
Phalaenopsis-gene486289               1    0    0    1    0   2
Phalaenopsis-gene486810               1    1    1    1    1   5
Phalaenopsis-gene486849               1    1    1    1    1   5
Phalaenopsis-gene487927               1    0    1    1    0   3
Phalaenopsis-gene487953               1    0    1    0    0   2
Phalaenopsis-gene487979               1    0    1    1    0   3
Phalaenopsis-gene488041               1    1    1    0    0   3
Phalaenopsis-gene49051                1    0    1    1    0   3
Phalaenopsis-gene495439               1    1    1    1    1   5
Phalaenopsis-gene501020               1    0    0    1    0   2
Phalaenopsis-gene501302               1    0    0    0    0   1
Phalaenopsis-gene501524               1    1    1    1    1   5
Phalaenopsis-gene503900               1    0    1    1    0   3
Phalaenopsis-gene503959               1    1    1    1    1   5
Phalaenopsis-gene504302               1    1    1    1    0   4
Phalaenopsis-gene50877                0    0    1    1    0   2
Phalaenopsis-gene513784               1    0    1    0    0   2
Phalaenopsis-gene514713               1    1    1    1    1   5
Phalaenopsis-gene520248               1    1    1    1    1   5
Phalaenopsis-gene526660               1    1    1    1    1   5
Phalaenopsis-gene531066               1    0    1    0    0   2
Phalaenopsis-gene531074               1    0    0    0    0   1
Phalaenopsis-gene5324                 1    1    1    1    1   5
Phalaenopsis-gene532605               0    0    0    1    0   1
Phalaenopsis-gene533157               1    1    1    1    1   5
Phalaenopsis-gene533985               0    0    1    0    0   1
Phalaenopsis-gene538022               1    1    1    1    1   5
Phalaenopsis-gene539394               1    1    1    1    1   5
Phalaenopsis-gene543759               0    0    0    1    0   1
Phalaenopsis-gene543764               1    0    1    1    1   4
Phalaenopsis-gene543779               1    1    1    1    1   5
Phalaenopsis-gene545605               1    1    1    1    1   5
Phalaenopsis-gene547237               1    1    1    1    1   5
Phalaenopsis-gene547246               1    0    1    1    0   3
Phalaenopsis-gene547702               0    0    1    0    0   1
Phalaenopsis-gene548163               1    0    1    1    0   3
Phalaenopsis-gene548165               1    0    0    1    0   2
Phalaenopsis-gene548277               1    0    1    1    0   3
Phalaenopsis-gene548685               0    0    1    0    0   1
Phalaenopsis-gene549255               0    0    1    1    0   2
Phalaenopsis-gene551025               1    1    1    1    1   5
Phalaenopsis-gene551475               1    1    1    1    1   5
Phalaenopsis-gene551714               1    0    1    1    0   3
Phalaenopsis-gene551734               1    0    1    1    0   3
Phalaenopsis-gene557532               1    1    1    1    1   5
Phalaenopsis-gene564696               1    1    1    1    1   5
Phalaenopsis-gene564697               1    1    1    1    1   5
Phalaenopsis-gene571260               1    1    1    1    1   5
Phalaenopsis-gene571723               0    1    1    1    0   3
Phalaenopsis-gene57413                1    1    1    1    1   5
Phalaenopsis-gene574464               0    0    1    0    0   1
Phalaenopsis-gene576394               1    1    1    1    1   5
Phalaenopsis-gene579483               1    1    1    1    0   4
Phalaenopsis-gene585488               1    1    1    1    1   5
Phalaenopsis-gene588148               0    0    0    1    0   1
Phalaenopsis-gene59001                1    0    1    0    0   2
Phalaenopsis-gene595296               0    0    1    1    0   2
Phalaenopsis-gene595733               1    0    1    1    0   3
Phalaenopsis-gene595734               1    1    1    1    1   5
Phalaenopsis-gene600777               1    1    0    1    0   3
Phalaenopsis-gene604583               1    0    0    1    0   2
Phalaenopsis-gene605750               1    1    1    1    1   5
Phalaenopsis-gene606558               0    0    1    0    0   1
Phalaenopsis-gene60665                1    0    1    1    0   3
Phalaenopsis-gene609251               1    0    1    1    0   3
Phalaenopsis-gene609356               0    0    1    0    0   1
Phalaenopsis-gene614511               0    0    0    1    0   1
Phalaenopsis-gene615297               0    0    1    0    0   1
Phalaenopsis-gene615890               1    0    1    0    0   2
Phalaenopsis-gene615896               1    0    0    0    0   1
Phalaenopsis-gene615901               1    0    1    1    0   3
Phalaenopsis-gene615951               0    0    1    1    0   2
Phalaenopsis-gene616008               0    0    0    1    0   1
Phalaenopsis-gene616019               1    1    1    1    1   5
Phalaenopsis-gene616027               0    0    1    1    0   2
Phalaenopsis-gene616319               1    0    1    1    0   3
Phalaenopsis-gene617864               0    0    1    1    0   2
Phalaenopsis-gene621610               1    1    1    1    1   5
Phalaenopsis-gene621648               1    0    1    1    0   3
Phalaenopsis-gene621673               1    0    1    1    0   3
Phalaenopsis-gene623791               0    0    1    1    0   2
Phalaenopsis-gene624142               0    0    0    1    0   1
Phalaenopsis-gene624193               1    1    0    1    0   3
Phalaenopsis-gene624198               0    0    0    1    0   1
Phalaenopsis-gene624703               1    0    0    1    0   2
Phalaenopsis-gene628942               1    1    1    1    1   5
Phalaenopsis-gene628973               1    0    1    0    0   2
Phalaenopsis-gene630818               0    0    0    1    0   1
Phalaenopsis-gene630999               1    0    1    0    0   2
Phalaenopsis-gene636663               1    0    1    1    0   3
Phalaenopsis-gene644318               1    0    0    1    0   2
Phalaenopsis-gene648773               1    1    1    1    1   5
Phalaenopsis-gene650824               1    0    0    0    1   2
Phalaenopsis-gene651051               1    0    1    1    0   3
Phalaenopsis-gene651108               0    0    1    1    0   2
Phalaenopsis-gene65134                1    1    1    1    1   5
Phalaenopsis-gene656287               1    0    0    1    0   2
Phalaenopsis-gene658961               1    0    1    1    1   4
Phalaenopsis-gene662755               0    0    0    1    0   1
Phalaenopsis-gene662858               1    0    1    1    0   3
Phalaenopsis-gene662915               0    0    1    1    0   2
Phalaenopsis-gene664799               1    0    1    1    0   3
Phalaenopsis-gene664836               1    0    0    1    0   2
Phalaenopsis-gene664837               0    0    0    1    0   1
Phalaenopsis-gene664951               1    1    1    1    1   5
Phalaenopsis-gene67292                1    0    1    1    0   3
Phalaenopsis-gene676708               1    1    1    1    1   5
Phalaenopsis-gene681381               1    1    1    1    1   5
Phalaenopsis-gene682264               1    0    0    1    0   2
Phalaenopsis-gene68395                0    0    1    0    0   1
Phalaenopsis-gene684266               1    1    1    1    1   5
Phalaenopsis-gene687792               1    0    1    1    0   3
Phalaenopsis-gene689118               1    0    1    1    0   3
Phalaenopsis-gene689789               1    1    1    1    1   5
Phalaenopsis-gene690189               1    0    1    1    0   3
Phalaenopsis-gene690198               0    0    0    1    0   1
Phalaenopsis-gene694126               0    0    1    0    0   1
Phalaenopsis-gene694167               1    0    0    1    0   2
Phalaenopsis-gene69657                1    0    1    1    0   3
Phalaenopsis-gene697724               1    0    0    1    0   2
Phalaenopsis-gene698048               0    0    1    1    0   2
Phalaenopsis-gene698184               0    0    0    0    0   0
Phalaenopsis-gene698585               1    1    1    1    1   5
Phalaenopsis-gene698935               1    1    0    1    0   3
Phalaenopsis-gene709600               0    0    0    0    0   0
Phalaenopsis-gene710164               1    1    1    1    1   5
Phalaenopsis-gene713                  1    1    1    1    1   5
Phalaenopsis-gene714278               1    0    1    0    0   2
Phalaenopsis-gene714285               1    1    1    1    1   5
Phalaenopsis-gene714297               1    1    1    1    1   5
Phalaenopsis-gene716025               1    0    1    0    0   2
Phalaenopsis-gene716209               1    1    1    1    1   5
Phalaenopsis-gene718830               1    1    1    1    1   5
Phalaenopsis-gene720885               1    1    1    1    0   4
Phalaenopsis-gene723865               1    1    1    1    1   5
Phalaenopsis-gene723896               0    1    1    1    0   3
Phalaenopsis-gene724606               1    1    1    1    1   5
Phalaenopsis-gene724613               1    0    0    1    0   2
Phalaenopsis-gene729430               1    0    1    1    0   3
Phalaenopsis-gene729575               1    1    1    1    1   5
Phalaenopsis-gene733026               1    1    1    1    0   4
Phalaenopsis-gene734960               1    0    1    1    0   3
Phalaenopsis-gene734961               1    0    0    1    0   2
Phalaenopsis-gene734994               1    0    0    0    0   1
Phalaenopsis-gene734995               1    0    1    1    0   3
Phalaenopsis-gene73672                0    0    1    1    0   2
Phalaenopsis-gene73974                1    1    1    1    1   5
Phalaenopsis-gene746007               1    0    1    1    0   3
Phalaenopsis-gene747381               1    0    1    1    0   3
Phalaenopsis-gene750413               0    0    1    1    0   2
Phalaenopsis-gene754091               0    0    1    1    0   2
Phalaenopsis-gene754092               1    1    1    0    0   3
Phalaenopsis-gene755139               0    0    1    1    0   2
Phalaenopsis-gene75768                1    0    1    0    0   2
Phalaenopsis-gene759076               1    0    0    1    0   2
Phalaenopsis-gene76037                1    0    1    1    0   3
Phalaenopsis-gene764439               0    1    1    0    0   2
Phalaenopsis-gene764821               1    1    1    1    1   5
Phalaenopsis-gene767783               1    1    0    1    0   3
Phalaenopsis-gene767865               1    0    1    1    0   3
Phalaenopsis-gene767985               1    0    1    1    0   3
Phalaenopsis-gene773891               1    0    0    1    0   2
Phalaenopsis-gene774262               0    0    1    1    0   2
Phalaenopsis-gene774329               1    1    1    1    1   5
Phalaenopsis-gene774336               1    0    0    1    0   2
Phalaenopsis-gene774383               1    1    1    1    1   5
Phalaenopsis-gene775350               1    1    1    0    0   3
Phalaenopsis-gene778914               1    1    1    1    0   4
Phalaenopsis-gene778948               0    0    1    1    1   3
Phalaenopsis-gene786505               1    0    1    1    0   3
Phalaenopsis-gene788726               1    1    1    1    1   5
Phalaenopsis-gene799782               1    0    1    0    0   2
Phalaenopsis-gene799865               1    1    1    1    0   4
Phalaenopsis-gene799909               1    0    1    0    0   2
Phalaenopsis-gene803461               0    0    1    1    0   2
Phalaenopsis-gene807136               1    1    1    1    1   5
Phalaenopsis-gene808536               1    0    0    1    0   2
Phalaenopsis-gene81195                1    0    0    1    0   2
Phalaenopsis-gene819079               1    0    0    1    1   3
Phalaenopsis-gene827635               1    1    1    1    1   5
Phalaenopsis-gene830994               1    1    1    1    1   5
Phalaenopsis-gene830995               1    1    1    1    1   5
Phalaenopsis-gene839767               1    1    1    1    1   5
Phalaenopsis-gene841519               1    1    1    1    1   5
Phalaenopsis-gene844985               1    1    1    1    1   5
Phalaenopsis-gene849414               1    0    1    1    0   3
Phalaenopsis-gene851693               1    1    1    0    0   3
Phalaenopsis-gene85173                1    0    0    1    0   2
Phalaenopsis-gene851789               1    0    0    0    0   1
Phalaenopsis-gene854743               1    1    1    1    1   5
Phalaenopsis-gene8555                 0    0    1    1    0   2
Phalaenopsis-gene857642               1    0    0    0    0   1
Phalaenopsis-gene857721               0    0    0    1    0   1
Phalaenopsis-gene8588                 1    0    0    1    0   2
Phalaenopsis-gene859290               0    0    1    1    0   2
Phalaenopsis-gene859317               1    1    1    1    1   5
Phalaenopsis-gene865362               0    0    1    1    0   2
Phalaenopsis-gene865889               1    1    1    1    1   5
Phalaenopsis-gene866784               0    0    1    1    0   2
Phalaenopsis-gene871012               1    1    1    1    1   5
Phalaenopsis-gene871938               0    0    1    0    0   1
Phalaenopsis-gene876735               0    0    0    0    0   0
Phalaenopsis-gene878381               1    1    1    1    1   5
Phalaenopsis-gene879641               1    1    1    1    1   5
Phalaenopsis-gene881711               0    1    1    1    0   3
Phalaenopsis-gene887000               1    1    1    1    1   5
Phalaenopsis-gene887105               1    0    0    1    0   2
Phalaenopsis-gene888219               1    1    1    1    1   5
Phalaenopsis-gene888266               1    0    1    1    0   3
Phalaenopsis-gene892956               1    0    0    0    0   1
Phalaenopsis-gene895202               0    0    0    1    0   1
Phalaenopsis-gene898919               1    0    1    0    0   2
Phalaenopsis-gene90144                1    1    1    1    1   5
Phalaenopsis-gene901630               0    0    0    1    0   1
Phalaenopsis-gene903707               1    1    1    1    1   5
Phalaenopsis-gene90387                1    1    1    1    1   5
Phalaenopsis-gene905103               0    0    1    1    0   2
Phalaenopsis-gene905641               1    1    1    1    1   5
Phalaenopsis-gene905645               1    0    1    0    0   2
Phalaenopsis-gene905671               1    1    1    1    1   5
Phalaenopsis-gene906807               1    0    0    1    0   2
Phalaenopsis-gene93723                1    0    1    1    0   3
Phalaenopsis-gene96113                1    0    1    0    0   2
Phalaenopsis-gene96891                1    0    1    1    0   3
Phalaenopsis-gene96976                1    0    1    1    0   3
Phalaenopsis-gene99480                1    1    1    1    1   5
Ophrys-gene920                        1    0    0    1    0   2
Ophrys-gene75071                      1    1    1    1    1   5
Ophrys-gene83999                      1    1    1    1    1   5
Ophrys-gene94891                      0    0    0    0    0   0
Ophrys-gene113671                     1    1    1    1    1   5
Ophrys-gene133861                     1    1    1    1    1   5
Ophrys-gene246907                     1    1    1    1    1   5
Ophrys-gene257642                     1    1    1    1    1   5
Ophrys-gene292981                     1    1    1    1    1   5
Ophrys-gene395603                     0    0    1    1    0   2
Ophrys-gene432927                     1    0    0    1    0   2
Ophrys-gene490467                     1    1    1    1    0   4
Ophrys-gene592066                     1    0    1    1    0   3
Ophrys-gene765623                     0    0    0    1    0   1
Ophrys-gene146316                     1    1    1    1    1   5
Ophrys-gene184028                     0    0    0    1    0   1
Ophrys-gene511916                     1    0    0    0    0   1
Ophrys-gene923                        1    1    1    1    1   5
Ophrys-gene2226                       1    0    1    0    0   2
Ophrys-gene4892                       1    1    1    1    1   5
Ophrys-gene7727                       1    1    1    1    1   5
Ophrys-gene8003                       1    0    1    1    0   3
Ophrys-gene10475                      1    1    1    1    1   5
Ophrys-gene10624                      0    1    1    1    0   3
Ophrys-gene12122                      0    0    1    1    0   2
Ophrys-gene23105                      1    1    1    1    1   5
Ophrys-gene26382                      1    1    1    1    1   5
Ophrys-gene50731                      1    0    0    1    0   2
Ophrys-gene51274                      0    0    1    1    0   2
Ophrys-gene51462                      0    0    1    0    0   1
Ophrys-gene53505                      1    0    0    1    0   2
Ophrys-gene55263                      1    0    0    1    0   2
Ophrys-gene56612                      1    1    1    1    1   5
Ophrys-gene59942                      1    0    0    1    0   2
Ophrys-gene60224                      1    1    1    1    1   5
Ophrys-gene68703                      1    0    0    1    0   2
Ophrys-gene84587                      0    0    1    1    0   2
Ophrys-gene101208                     1    1    1    1    1   5
Ophrys-gene102174                     1    1    1    1    1   5
Ophrys-gene103686                     1    1    1    1    1   5
Ophrys-gene112204                     1    1    1    1    1   5
Ophrys-gene126227                     1    1    1    1    1   5
Ophrys-gene132871                     1    1    1    1    1   5
Ophrys-gene136714                     1    1    1    1    1   5
Ophrys-gene139714                     1    0    0    1    0   2
Ophrys-gene144430                     1    1    1    1    1   5
Ophrys-gene147328                     1    1    1    1    1   5
Ophrys-gene151328                     1    0    0    1    0   2
Ophrys-gene155404                     1    1    1    1    1   5
Ophrys-gene155985                     1    1    1    1    1   5
Ophrys-gene169564                     1    0    1    0    0   2
Ophrys-gene169604                     1    1    1    1    1   5
Ophrys-gene173239                     0    0    0    1    0   1
Ophrys-gene175404                     1    0    0    1    0   2
Ophrys-gene178511                     1    1    1    1    1   5
Ophrys-gene180209                     1    0    0    1    0   2
Ophrys-gene182854                     0    0    1    1    0   2
Ophrys-gene183177                     1    1    1    1    1   5
Ophrys-gene183903                     1    0    0    0    0   1
Ophrys-gene200763                     1    1    1    1    1   5
Ophrys-gene203712                     1    0    0    0    0   1
Ophrys-gene205854                     1    1    1    1    1   5
Ophrys-gene210847                     1    0    1    0    0   2
Ophrys-gene217617                     1    0    0    1    0   2
Ophrys-gene230467                     1    0    1    0    0   2
Ophrys-gene234913                     1    1    1    1    1   5
Ophrys-gene243839                     1    1    1    1    1   5
Ophrys-gene252460                     0    0    1    1    0   2
Ophrys-gene258668                     1    0    1    1    0   3
Ophrys-gene259027                     0    0    0    0    0   0
Ophrys-gene268061                     0    0    0    1    0   1
Ophrys-gene268410                     1    1    1    1    1   5
Ophrys-gene271125                     1    1    1    1    1   5
Ophrys-gene272615                     1    1    1    1    1   5
Ophrys-gene278481                     0    1    1    1    0   3
Ophrys-gene278952                     1    0    1    1    0   3
Ophrys-gene287277                     1    1    1    1    1   5
Ophrys-gene290436                     1    0    1    1    0   3
Ophrys-gene291895                     1    1    1    1    1   5
Ophrys-gene293810                     1    0    1    1    0   3
Ophrys-gene295603                     0    0    0    1    0   1
Ophrys-gene299811                     1    0    1    1    0   3
Ophrys-gene302149                     1    0    0    1    1   3
Ophrys-gene303797                     1    0    0    1    0   2
Ophrys-gene304406                     1    1    1    1    1   5
Ophrys-gene324709                     1    1    1    1    1   5
Ophrys-gene331583                     0    0    0    0    0   0
Ophrys-gene339385                     1    0    1    1    0   3
Ophrys-gene339391                     1    1    1    1    1   5
Ophrys-gene375828                     1    0    1    1    0   3
Ophrys-gene377611                     1    1    1    1    1   5
Ophrys-gene390539                     1    0    0    0    0   1
Ophrys-gene392653                     1    1    1    1    1   5
Ophrys-gene396871                     1    0    0    1    0   2
Ophrys-gene399440                     1    1    1    1    1   5
Ophrys-gene413903                     1    1    1    1    1   5
Ophrys-gene429438                     1    1    1    1    1   5
Ophrys-gene434947                     0    0    1    1    0   2
Ophrys-gene434976                     1    1    1    1    1   5
Ophrys-gene440802                     1    1    1    1    1   5
Ophrys-gene440936                     1    0    0    1    0   2
Ophrys-gene444890                     1    0    0    1    0   2
Ophrys-gene444948                     1    0    0    0    0   1
Ophrys-gene446255                     0    0    1    0    0   1
Ophrys-gene454341                     1    0    1    1    0   3
Ophrys-gene473126                     1    1    1    1    1   5
Ophrys-gene479119                     1    1    1    1    1   5
Ophrys-gene481276                     1    1    1    1    1   5
Ophrys-gene495239                     0    0    0    1    0   1
Ophrys-gene495549                     1    0    0    1    0   2
Ophrys-gene498140                     1    1    1    1    1   5
Ophrys-gene499445                     1    1    1    1    1   5
Ophrys-gene508811                     1    1    1    1    1   5
Ophrys-gene509229                     1    1    1    1    1   5
Ophrys-gene511800                     0    0    0    1    0   1
Ophrys-gene513857                     1    1    1    1    0   4
Ophrys-gene518816                     1    1    1    1    1   5
Ophrys-gene519088                     1    0    0    1    0   2
Ophrys-gene526321                     1    1    1    1    1   5
Ophrys-gene527699                     1    0    0    0    0   1
Ophrys-gene536785                     1    1    1    1    1   5
Ophrys-gene542127                     0    0    0    1    0   1
Ophrys-gene547546                     0    0    0    1    0   1
Ophrys-gene554488                     1    0    0    1    0   2
Ophrys-gene555163                     1    0    0    1    0   2
Ophrys-gene568674                     0    0    0    1    0   1
Ophrys-gene571439                     1    1    1    1    1   5
Ophrys-gene586730                     1    0    0    1    0   2
Ophrys-gene587294                     1    1    1    1    1   5
Ophrys-gene587317                     1    0    0    1    1   3
Ophrys-gene591166                     1    0    0    1    0   2
Ophrys-gene607167                     1    0    0    0    0   1
Ophrys-gene609367                     1    1    1    1    1   5
Ophrys-gene610361                     0    0    0    1    0   1
Ophrys-gene613539                     1    1    1    1    1   5
Ophrys-gene618332                     0    0    0    0    0   0
Ophrys-gene628382                     1    0    1    1    0   3
Ophrys-gene646750                     1    1    1    1    1   5
Ophrys-gene650966                     0    0    0    1    0   1
Ophrys-gene660370                     0    0    0    1    0   1
Ophrys-gene669139                     1    1    1    1    1   5
Ophrys-gene681951                     1    1    1    1    1   5
Ophrys-gene692739                     0    1    0    1    0   2
Ophrys-gene694828                     1    0    0    0    0   1
Ophrys-gene709156                     1    0    0    1    0   2
Ophrys-gene709212                     0    0    0    1    0   1
Ophrys-gene709764                     1    1    0    1    0   3
Ophrys-gene710966                     1    0    0    0    1   2
Ophrys-gene714341                     0    0    0    1    0   1
Ophrys-gene714803                     1    1    1    0    0   3
Ophrys-gene731541                     1    0    1    1    0   3
Ophrys-gene757389                     0    0    0    0    0   0
Ophrys-gene190309                     0    0    0    1    0   1
Ophrys-gene38739                      0    0    1    0    0   1
Ophrys-gene141876                     1    0    0    1    0   2
Ophrys-gene220973                     0    0    1    1    0   2
Ophrys-gene229478                     1    0    1    0    0   2
Ophrys-gene238504                     1    0    1    0    0   2
Ophrys-gene375216                     1    1    1    1    1   5
Ophrys-gene441385                     1    1    1    1    1   5
Ophrys-gene542223                     1    1    1    1    1   5
Ophrys-gene577265                     1    1    1    1    1   5
Ophrys-gene2836                       1    1    1    1    1   5
Ophrys-gene3453                       1    0    0    1    0   2
Ophrys-gene4658                       1    1    0    0    0   2
Ophrys-gene6800                       1    1    1    1    1   5
Ophrys-gene8278                       1    1    1    1    1   5
Ophrys-gene11858                      1    1    1    1    1   5
Ophrys-gene12315                      1    1    1    1    1   5
Ophrys-gene12370                      0    0    1    0    0   1
Ophrys-gene21338                      1    1    1    1    1   5
Ophrys-gene21386                      1    0    1    1    0   3
Ophrys-gene21647                      1    0    1    0    0   2
Ophrys-gene24111                      1    1    1    1    1   5
Ophrys-gene24272                      1    0    0    0    0   1
Ophrys-gene46936                      1    0    0    1    1   3
Ophrys-gene50719                      1    1    1    1    1   5
Ophrys-gene60401                      1    1    1    1    1   5
Ophrys-gene60409                      1    1    1    0    0   3
Ophrys-gene61755                      1    1    1    1    1   5
Ophrys-gene62685                      1    0    0    1    0   2
Ophrys-gene62974                      1    1    1    1    1   5
Ophrys-gene63437                      1    1    1    1    1   5
Ophrys-gene64296                      1    1    1    1    1   5
Ophrys-gene69730                      0    0    0    1    0   1
Ophrys-gene86837                      1    0    1    1    0   3
Ophrys-gene88638                      1    0    0    0    0   1
Ophrys-gene93911                      0    0    1    1    0   2
Ophrys-gene101218                     1    0    1    1    0   3
Ophrys-gene102221                     1    1    1    1    1   5
Ophrys-gene108189                     0    1    0    1    0   2
Ophrys-gene122202                     1    0    1    0    0   2
Ophrys-gene123137                     1    0    0    1    0   2
Ophrys-gene131877                     1    0    1    1    0   3
Ophrys-gene132988                     1    1    1    1    1   5
Ophrys-gene134877                     0    1    0    1    0   2
Ophrys-gene136547                     1    0    0    1    0   2
Ophrys-gene140669                     1    1    0    1    0   3
Ophrys-gene143308                     1    1    1    1    1   5
Ophrys-gene156552                     1    0    1    1    0   3
Ophrys-gene160689                     1    0    1    1    0   3
Ophrys-gene169607                     1    1    1    1    0   4
Ophrys-gene178828                     1    1    1    1    1   5
Ophrys-gene178870                     1    0    0    1    0   2
Ophrys-gene180225                     1    1    1    1    1   5
Ophrys-gene182661                     1    0    1    0    0   2
Ophrys-gene183736                     0    0    1    1    0   2
Ophrys-gene184279                     1    0    0    1    0   2
Ophrys-gene199099                     1    1    1    1    1   5
Ophrys-gene203630                     0    0    1    1    0   2
Ophrys-gene205893                     1    0    0    0    0   1
Ophrys-gene205933                     1    1    1    1    1   5
Ophrys-gene205975                     0    0    0    1    0   1
Ophrys-gene210667                     0    0    0    1    0   1
Ophrys-gene211088                     1    0    0    0    0   1
Ophrys-gene212069                     1    1    1    1    1   5
Ophrys-gene213439                     1    0    0    1    0   2
Ophrys-gene216805                     1    0    0    1    0   2
Ophrys-gene217622                     0    0    0    1    0   1
Ophrys-gene218282                     1    0    0    1    0   2
Ophrys-gene234784                     1    1    1    1    1   5
Ophrys-gene236651                     1    0    0    1    0   2
Ophrys-gene252988                     0    0    1    1    0   2
Ophrys-gene272644                     1    1    1    1    1   5
Ophrys-gene280475                     1    0    1    1    0   3
Ophrys-gene286806                     1    1    1    1    1   5
Ophrys-gene286848                     1    0    1    1    0   3
Ophrys-gene289497                     1    0    0    1    0   2
Ophrys-gene293009                     1    0    0    1    1   3
Ophrys-gene294468                     1    1    1    1    1   5
Ophrys-gene295938                     1    1    1    1    1   5
Ophrys-gene296628                     1    1    1    1    1   5
Ophrys-gene298056                     1    0    0    1    0   2
Ophrys-gene299821                     1    1    1    1    1   5
Ophrys-gene299858                     0    0    0    1    0   1
Ophrys-gene303926                     0    0    0    1    0   1
Ophrys-gene307414                     1    1    1    1    1   5
Ophrys-gene324719                     1    0    1    1    0   3
Ophrys-gene355234                     1    0    0    1    0   2
Ophrys-gene369021                     1    0    1    1    0   3
Ophrys-gene369993                     0    0    0    0    0   0
Ophrys-gene370141                     0    0    0    0    0   0
Ophrys-gene375376                     1    0    0    1    0   2
Ophrys-gene375814                     1    1    1    1    1   5
Ophrys-gene379516                     1    1    1    1    1   5
Ophrys-gene384531                     1    0    1    0    0   2
Ophrys-gene390937                     1    1    0    1    0   3
Ophrys-gene392577                     1    0    0    1    0   2
Ophrys-gene395696                     1    1    1    1    1   5
Ophrys-gene396532                     1    0    1    0    0   2
Ophrys-gene398689                     1    1    1    1    1   5
Ophrys-gene399430                     1    1    1    1    1   5
Ophrys-gene406838                     1    0    1    0    0   2
Ophrys-gene409491                     1    0    0    1    0   2
Ophrys-gene410688                     0    0    0    0    0   0
Ophrys-gene411940                     1    1    1    1    1   5
Ophrys-gene418717                     0    0    1    1    0   2
Ophrys-gene422273                     1    0    1    1    0   3
Ophrys-gene424053                     0    0    1    1    0   2
Ophrys-gene425279                     1    1    1    1    1   5
Ophrys-gene427775                     1    0    0    1    0   2
Ophrys-gene429528                     1    1    1    1    1   5
Ophrys-gene444838                     1    1    1    1    1   5
Ophrys-gene444896                     1    0    0    0    0   1
Ophrys-gene446232                     1    1    1    1    1   5
Ophrys-gene449233                     1    0    1    1    0   3
Ophrys-gene457276                     1    1    1    1    1   5
Ophrys-gene461696                     1    1    1    1    1   5
Ophrys-gene469973                     1    1    1    1    1   5
Ophrys-gene483192                     1    0    0    1    0   2
Ophrys-gene484948                     1    1    1    1    1   5
Ophrys-gene488643                     1    0    1    1    0   3
Ophrys-gene488873                     1    0    0    0    0   1
Ophrys-gene489500                     1    0    0    1    0   2
Ophrys-gene492796                     1    1    1    1    1   5
Ophrys-gene495362                     1    0    0    1    0   2
Ophrys-gene495597                     1    0    0    1    0   2
Ophrys-gene503701                     1    1    1    1    1   5
Ophrys-gene508805                     1    1    1    1    1   5
Ophrys-gene512021                     0    0    0    1    0   1
Ophrys-gene512109                     1    0    0    1    0   2
Ophrys-gene512378                     1    0    1    1    0   3
Ophrys-gene516165                     1    0    0    0    0   1
Ophrys-gene523165                     1    0    0    0    0   1
Ophrys-gene525691                     1    0    0    0    0   1
Ophrys-gene526209                     1    0    0    1    0   2
Ophrys-gene527628                     0    0    1    1    0   2
Ophrys-gene532880                     1    0    0    1    0   2
Ophrys-gene542229                     1    1    1    1    1   5
Ophrys-gene542821                     1    1    1    1    1   5
Ophrys-gene547741                     0    0    0    0    0   0
Ophrys-gene555937                     1    1    1    1    0   4
Ophrys-gene588915                     1    0    0    1    0   2
Ophrys-gene591164                     1    1    1    1    1   5
Ophrys-gene591399                     1    1    1    1    1   5
Ophrys-gene594804                     0    0    0    0    1   1
Ophrys-gene605295                     0    0    0    0    0   0
Ophrys-gene608033                     1    0    0    1    0   2
Ophrys-gene609913                     1    0    0    1    0   2
Ophrys-gene612202                     1    1    1    1    1   5
Ophrys-gene616642                     0    0    0    1    0   1
Ophrys-gene619553                     1    0    1    1    0   3
Ophrys-gene632791                     0    0    0    1    0   1
Ophrys-gene632823                     1    0    1    1    0   3
Ophrys-gene670541                     1    1    1    1    1   5
Ophrys-gene677168                     1    1    1    1    1   5
Ophrys-gene679576                     1    1    1    1    1   5
Ophrys-gene696640                     1    0    0    1    0   2
Ophrys-gene700204                     1    1    1    1    1   5
Ophrys-gene700917                     1    0    1    1    0   3
Ophrys-gene701025                     1    0    0    0    0   1
Ophrys-gene709211                     1    0    1    0    0   2
Ophrys-gene710962                     0    0    1    1    0   2
Ophrys-gene712483                     1    1    1    1    1   5
Ophrys-gene718521                     0    1    0    1    0   2
Ophrys-gene724437                     1    1    1    1    1   5
Ophrys-gene730638                     1    1    1    1    1   5
Ophrys-gene746805                     1    0    0    0    1   2
Ophrys-gene747170                     1    0    0    0    0   1
Ophrys-gene753290                     1    1    1    1    1   5
Ophrys-gene759887                     1    0    0    1    0   2
Ophrys-gene765247                     1    1    1    1    1   5
Ophrys-gene489212                     1    1    1    1    1   5
Ophrys-gene206617                     1    0    1    1    0   3
Ophrys-gene213024                     1    0    0    0    0   1
Ophrys-gene220972                     1    1    1    1    1   5
Ophrys-gene625031                     1    1    1    1    1   5
Ophrys-gene77942                      1    1    1    1    1   5
Ophrys-gene182850                     0    0    0    0    0   0
Ophrys-gene200203                     1    0    1    1    0   3
Ophrys-gene210752                     1    0    0    0    0   1
Ophrys-gene271981                     0    0    1    0    0   1
Ophrys-gene274853                     1    1    1    1    1   5
Ophrys-gene275187                     1    0    0    1    0   2
Ophrys-gene513991                     1    1    1    1    1   5
Ophrys-gene570985                     1    1    1    1    1   5
Ophrys-gene694365                     1    1    1    1    1   5
Ophrys-gene2018                       1    1    1    1    1   5
Ophrys-gene2683                       1    0    0    0    0   1
Ophrys-gene3472                       0    0    1    1    0   2
Ophrys-gene3775                       1    0    0    0    0   1
Ophrys-gene6098                       1    0    1    0    0   2
Ophrys-gene23826                      1    0    0    0    0   1
Ophrys-gene59970                      1    0    1    1    0   3
Ophrys-gene60398                      1    1    1    1    1   5
Ophrys-gene77254                      1    1    1    1    1   5
Ophrys-gene88140                      1    0    0    1    0   2
Ophrys-gene88968                      1    1    1    1    1   5
Ophrys-gene152276                     0    0    0    1    0   1
Ophrys-gene154925                     1    0    0    1    0   2
Ophrys-gene154954                     1    1    1    1    1   5
Ophrys-gene155351                     1    0    0    1    0   2
Ophrys-gene159153                     1    1    1    1    1   5
Ophrys-gene170940                     0    0    0    1    0   1
Ophrys-gene183181                     0    0    0    1    0   1
Ophrys-gene184022                     0    0    0    1    0   1
Ophrys-gene199056                     1    1    1    1    1   5
Ophrys-gene213091                     1    1    1    1    1   5
Ophrys-gene243831                     1    1    1    1    1   5
Ophrys-gene250617                     1    0    0    1    0   2
Ophrys-gene254212                     1    1    1    1    1   5
Ophrys-gene254923                     1    1    1    1    1   5
Ophrys-gene262700                     1    1    1    1    1   5
Ophrys-gene281579                     1    1    1    1    1   5
Ophrys-gene282615                     1    1    1    1    1   5
Ophrys-gene291896                     1    1    1    1    1   5
Ophrys-gene294944                     0    1    1    1    0   3
Ophrys-gene295254                     1    0    0    0    0   1
Ophrys-gene307415                     1    1    1    1    1   5
Ophrys-gene337511                     1    0    0    0    0   1
Ophrys-gene356187                     1    0    1    0    0   2
Ophrys-gene369987                     1    1    1    1    1   5
Ophrys-gene395865                     1    1    0    1    0   3
Ophrys-gene398920                     1    1    1    1    1   5
Ophrys-gene432604                     1    1    0    1    0   3
Ophrys-gene444626                     1    0    1    0    0   2
Ophrys-gene462294                     1    1    1    1    1   5
Ophrys-gene478963                     0    0    0    1    0   1
Ophrys-gene488929                     1    1    1    1    1   5
Ophrys-gene491440                     1    1    1    1    1   5
Ophrys-gene495492                     0    0    0    0    0   0
Ophrys-gene503526                     0    0    0    1    0   1
Ophrys-gene503704                     1    1    1    1    1   5
Ophrys-gene525694                     1    0    0    1    0   2
Ophrys-gene543931                     1    1    1    1    1   5
Ophrys-gene545816                     1    1    1    1    1   5
Ophrys-gene551671                     1    0    0    1    0   2
Ophrys-gene565915                     1    0    1    1    0   3
Ophrys-gene568675                     1    1    1    1    1   5
Ophrys-gene570884                     1    0    0    1    0   2
Ophrys-gene590710                     1    1    1    1    1   5
Ophrys-gene595512                     1    1    1    1    1   5
Ophrys-gene633269                     1    1    1    1    1   5
Ophrys-gene640537                     1    1    1    1    1   5
Ophrys-gene645082                     0    0    1    1    0   2
Ophrys-gene646764                     1    1    1    1    1   5
Ophrys-gene661514                     0    0    0    1    0   1
Ophrys-gene676201                     1    1    1    1    1   5
Ophrys-gene690491                     1    0    0    1    0   2
Ophrys-gene693031                     1    0    0    0    0   1
Ophrys-gene700029                     0    1    0    1    0   2
Ophrys-gene701037                     1    0    0    1    0   2
Ophrys-gene710466                     0    1    0    0    0   1
Ophrys-gene722679                     1    1    1    1    1   5
Ophrys-gene748952                     1    1    1    1    1   5
Ophrys-gene769412                     1    1    1    1    1   5
Ophrys-gene9037                       1    1    1    1    1   5
Ophrys-gene10550                      1    1    1    1    1   5
Ophrys-gene10556                      1    1    1    1    1   5
Ophrys-gene11087                      1    1    1    1    1   5
Ophrys-gene11198                      1    1    1    1    1   5
Ophrys-gene36127                      1    1    1    1    1   5
Ophrys-gene50844                      1    0    1    0    0   2
Ophrys-gene77825                      1    1    1    1    1   5
Ophrys-gene77906                      1    1    1    1    0   4
Ophrys-gene84212                      0    0    0    1    0   1
Ophrys-gene93994                      1    0    0    1    0   2
Ophrys-gene94157                      0    0    1    1    0   2
Ophrys-gene131889                     1    1    1    1    1   5
Ophrys-gene136583                     1    0    0    0    0   1
Ophrys-gene143305                     0    0    0    1    0   1
Ophrys-gene146580                     1    0    0    1    0   2
Ophrys-gene149034                     1    0    0    0    0   1
Ophrys-gene166643                     0    0    1    0    0   1
Ophrys-gene168579                     1    0    0    1    0   2
Ophrys-gene195028                     1    1    1    1    1   5
Ophrys-gene206011                     0    0    0    1    0   1
Ophrys-gene210892                     1    1    1    1    1   5
Ophrys-gene217196                     1    1    1    1    1   5
Ophrys-gene235599                     1    0    1    1    0   3
Ophrys-gene254741                     1    0    1    1    0   3
Ophrys-gene258726                     1    0    0    1    0   2
Ophrys-gene275650                     1    0    0    1    0   2
Ophrys-gene279296                     1    1    1    1    1   5
Ophrys-gene300033                     1    0    0    1    0   2
Ophrys-gene305024                     1    0    0    1    0   2
Ophrys-gene305988                     1    0    0    1    0   2
Ophrys-gene326490                     1    0    0    1    0   2
Ophrys-gene338306                     1    0    0    1    0   2
Ophrys-gene341508                     1    0    1    1    0   3
Ophrys-gene385045                     1    1    1    1    1   5
Ophrys-gene385615                     1    0    0    1    0   2
Ophrys-gene385749                     0    0    0    1    0   1
Ophrys-gene392589                     1    0    0    1    0   2
Ophrys-gene396669                     0    0    0    0    0   0
Ophrys-gene397346                     1    1    1    1    1   5
Ophrys-gene406839                     1    0    1    1    0   3
Ophrys-gene419750                     1    0    0    0    0   1
Ophrys-gene431760                     1    1    1    1    1   5
Ophrys-gene444632                     0    0    0    0    0   0
Ophrys-gene445199                     1    0    0    1    0   2
Ophrys-gene452742                     1    0    0    1    1   3
Ophrys-gene484949                     1    0    0    1    0   2
Ophrys-gene515090                     1    0    0    1    0   2
Ophrys-gene518810                     1    1    1    1    1   5
Ophrys-gene518989                     1    0    1    1    0   3
Ophrys-gene525696                     1    0    0    0    0   1
Ophrys-gene525982                     1    0    1    0    0   2
Ophrys-gene538881                     1    0    0    1    0   2
Ophrys-gene565189                     1    0    0    1    0   2
Ophrys-gene566433                     1    0    0    1    0   2
Ophrys-gene568780                     1    1    1    1    1   5
Ophrys-gene579499                     1    1    1    1    1   5
Ophrys-gene601001                     1    0    1    1    0   3
Ophrys-gene616681                     1    1    1    1    1   5
Ophrys-gene617201                     1    0    1    1    0   3
Ophrys-gene692826                     1    0    0    0    0   1
Ophrys-gene695714                     0    0    1    0    0   1
Ophrys-gene695871                     1    1    1    1    1   5
Ophrys-gene698768                     1    0    1    1    0   3
Ophrys-gene708984                     1    1    1    1    1   5
Ophrys-gene710547                     1    1    1    1    1   5
Ophrys-gene715344                     1    1    1    1    1   5
Ophrys-gene715363                     1    1    0    1    0   3
Ophrys-gene722353                     1    1    1    1    1   5
Ophrys-gene746821                     1    1    1    1    1   5
Ophrys-gene747683                     1    1    0    0    0   2
Ophrys-gene763580                     0    0    1    1    0   2
Ophrys-gene765582                     1    1    0    1    0   3
Ophrys-gene3892                       0    0    0    0    0   0
Ophrys-gene6180                       1    0    0    1    0   2
Ophrys-gene8060                       1    1    1    1    1   5
Ophrys-gene10905                      0    0    0    0    0   0
Ophrys-gene20914                      1    0    0    1    1   3
Ophrys-gene23032                      1    1    1    1    1   5
Ophrys-gene54042                      1    1    1    1    1   5
Ophrys-gene57534                      0    0    1    1    0   2
Ophrys-gene71246                      1    1    0    1    0   3
Ophrys-gene76254                      1    0    0    0    0   1
Ophrys-gene94907                      1    1    1    0    0   3
Ophrys-gene107734                     1    1    1    1    1   5
Ophrys-gene111697                     0    0    0    1    0   1
Ophrys-gene123207                     1    1    1    1    1   5
Ophrys-gene177777                     1    0    0    0    1   2
Ophrys-gene181535                     1    1    1    1    1   5
Ophrys-gene184179                     1    0    1    1    0   3
Ophrys-gene210372                     1    0    0    1    0   2
Ophrys-gene258318                     0    0    1    1    0   2
Ophrys-gene272081                     1    1    1    0    0   3
Ophrys-gene278294                     1    0    1    1    0   3
Ophrys-gene282210                     1    0    1    0    0   2
Ophrys-gene341186                     1    0    1    0    0   2
Ophrys-gene352180                     1    0    1    1    0   3
Ophrys-gene373794                     1    1    1    1    1   5
Ophrys-gene375071                     1    0    1    1    0   3
Ophrys-gene375826                     1    0    0    1    0   2
Ophrys-gene378143                     1    1    1    1    1   5
Ophrys-gene384536                     0    0    1    1    0   2
Ophrys-gene391030                     1    0    0    1    0   2
Ophrys-gene406862                     1    1    1    1    1   5
Ophrys-gene433111                     1    0    0    1    0   2
Ophrys-gene444680                     1    0    0    1    0   2
Ophrys-gene444942                     1    0    0    1    0   2
Ophrys-gene445341                     0    0    0    1    0   1
Ophrys-gene475284                     1    0    0    1    0   2
Ophrys-gene484151                     0    0    1    1    0   2
Ophrys-gene487203                     1    1    1    1    1   5
Ophrys-gene508812                     0    1    0    1    0   2
Ophrys-gene531881                     0    0    0    1    0   1
Ophrys-gene567573                     1    1    1    1    1   5
Ophrys-gene586416                     1    1    1    1    1   5
Ophrys-gene586418                     1    1    1    1    1   5
Ophrys-gene590114                     1    0    1    1    0   3
Ophrys-gene591444                     1    1    1    1    1   5
Ophrys-gene616735                     1    1    1    1    1   5
Ophrys-gene617266                     0    0    0    1    0   1
Ophrys-gene617366                     0    0    0    0    0   0
Ophrys-gene619113                     1    1    1    0    0   3
Ophrys-gene634728                     1    1    1    1    1   5
Ophrys-gene636281                     1    0    0    1    0   2
Ophrys-gene661400                     0    0    0    1    0   1
Ophrys-gene675346                     1    0    0    1    0   2
Ophrys-gene693182                     1    0    1    0    0   2
Ophrys-gene698736                     1    1    1    1    1   5
Ophrys-gene700175                     1    0    1    1    0   3
Ophrys-gene707679                     1    1    1    1    0   4
Ophrys-gene708973                     1    0    1    0    0   2
Ophrys-gene752687                     1    0    1    1    0   3
Ophrys-gene758894                     0    1    0    1    0   2
Ophrys-gene21738                      1    1    1    1    1   5
Ophrys-gene99087                      1    1    0    1    0   3
Ophrys-gene189236                     1    0    1    1    0   3
Ophrys-gene364762                     1    1    1    1    1   5
Ophrys-gene456717                     0    0    0    0    0   0
Ophrys-gene609336                     1    0    0    0    0   1
Ophrys-gene676647                     1    1    1    1    1   5
Ophrys-gene722788                     0    0    0    1    0   1
Ophrys-gene29                         1    1    1    0    0   3
Ophrys-gene88648                      1    0    1    1    1   4
Ophrys-gene190060                     0    0    0    1    0   1
Ophrys-gene303832                     1    0    0    0    0   1
Ophrys-gene459917                     0    0    0    0    0   0
Ophrys-gene712809                     0    0    0    1    1   2
Ophrys-gene742036                     1    0    0    1    0   2
Ophrys-gene263925                     1    0    0    1    0   2
Ophrys-gene385570                     0    0    0    0    0   0
Ophrys-gene385706                     0    0    0    1    0   1
Ophrys-gene432716                     1    0    0    0    0   1
Ophrys-gene656691                     1    0    0    0    0   1
Ophrys-gene687837                     1    0    0    1    0   2
Ophrys-gene50724                      1    0    0    0    0   1
Phalaenopsis-gene317458               1    0    0    0    0   1
Phalaenopsis-gene317522               1    1    1    1    1   5
Phalaenopsis-gene351875               1    1    1    1    1   5
Phalaenopsis-gene393779               0    0    1    1    0   2
Phalaenopsis-gene393873               1    0    0    1    0   2
Phalaenopsis-gene44920                1    1    1    1    0   4
Phalaenopsis-gene567821               1    0    0    1    0   2
Phalaenopsis-gene651658               1    1    1    1    1   5
Phalaenopsis-gene737103               0    0    1    0    0   1
Phalaenopsis-gene839568               1    0    1    1    0   3
Phalaenopsis-gene798363               1    1    1    1    1   5
Phalaenopsis-gene817596               1    0    0    1    0   2
Phalaenopsis-gene848968               0    0    0    0    0   0
Phalaenopsis-gene851608               0    0    0    1    0   1
Phalaenopsis-gene113084               0    0    1    0    0   1
Phalaenopsis-gene113138               1    1    1    0    0   3
Phalaenopsis-gene11920                1    1    1    1    1   5
Phalaenopsis-gene135350               1    1    1    1    1   5
Phalaenopsis-gene138424               1    0    1    1    0   3
Phalaenopsis-gene138435               1    0    0    1    1   3
Phalaenopsis-gene138874               1    1    1    1    1   5
Phalaenopsis-gene138877               1    1    1    1    1   5
Phalaenopsis-gene143640               1    1    1    1    1   5
Phalaenopsis-gene165987               1    0    0    1    0   2
Phalaenopsis-gene168018               1    0    1    1    0   3
Phalaenopsis-gene170757               0    0    1    0    0   1
Phalaenopsis-gene172167               1    1    1    1    1   5
Phalaenopsis-gene173904               0    0    0    1    0   1
Phalaenopsis-gene183206               1    1    1    1    1   5
Phalaenopsis-gene184323               1    1    1    1    1   5
Phalaenopsis-gene188771               1    1    1    1    1   5
Phalaenopsis-gene197933               0    0    1    1    0   2
Phalaenopsis-gene202308               1    0    1    1    0   3
Phalaenopsis-gene203578               1    0    0    1    0   2
Phalaenopsis-gene209169               0    0    1    1    0   2
Phalaenopsis-gene216669               1    0    1    1    0   3
Phalaenopsis-gene216865               1    0    1    1    0   3
Phalaenopsis-gene22248                1    0    0    1    0   2
Phalaenopsis-gene229637               0    0    1    1    0   2
Phalaenopsis-gene233160               1    0    1    0    0   2
Phalaenopsis-gene241275               1    0    0    0    0   1
Phalaenopsis-gene247255               1    1    0    0    0   2
Phalaenopsis-gene24944                1    1    1    1    1   5
Phalaenopsis-gene260503               1    1    1    1    1   5
Phalaenopsis-gene27076                1    1    1    1    1   5
Phalaenopsis-gene281468               1    0    1    0    0   2
Phalaenopsis-gene28366                1    0    0    0    0   1
Phalaenopsis-gene295449               1    1    1    1    1   5
Phalaenopsis-gene296605               0    0    0    0    0   0
Phalaenopsis-gene306776               1    1    1    1    1   5
Phalaenopsis-gene325319               1    1    1    1    1   5
Phalaenopsis-gene340024               1    0    0    0    0   1
Phalaenopsis-gene342019               0    0    1    1    0   2
Phalaenopsis-gene342825               1    0    0    1    0   2
Phalaenopsis-gene343527               1    1    1    1    0   4
Phalaenopsis-gene344812               1    1    0    0    0   2
Phalaenopsis-gene351243               1    0    1    1    0   3
Phalaenopsis-gene351870               1    0    0    0    0   1
Phalaenopsis-gene361737               0    0    0    0    1   1
Phalaenopsis-gene370224               1    1    1    1    1   5
Phalaenopsis-gene371984               1    0    0    1    0   2
Phalaenopsis-gene375141               0    0    0    0    0   0
Phalaenopsis-gene38619                1    1    0    1    0   3
Phalaenopsis-gene387297               1    0    0    0    1   2
Phalaenopsis-gene388030               1    1    1    1    1   5
Phalaenopsis-gene391164               1    1    1    1    1   5
Phalaenopsis-gene410941               1    0    1    1    0   3
Phalaenopsis-gene412971               1    1    1    1    1   5
Phalaenopsis-gene423729               1    1    1    1    1   5
Phalaenopsis-gene442696               1    0    1    1    0   3
Phalaenopsis-gene453896               1    1    1    1    1   5
Phalaenopsis-gene456684               1    1    1    1    1   5
Phalaenopsis-gene457370               1    0    0    1    0   2
Phalaenopsis-gene462895               1    0    1    1    0   3
Phalaenopsis-gene471597               0    0    0    0    0   0
Phalaenopsis-gene499453               1    0    1    1    0   3
Phalaenopsis-gene505497               1    1    1    1    1   5
Phalaenopsis-gene513870               0    0    1    0    0   1
Phalaenopsis-gene51440                1    1    1    1    1   5
Phalaenopsis-gene520894               0    0    0    1    0   1
Phalaenopsis-gene522407               0    0    1    0    1   2
Phalaenopsis-gene525841               1    1    1    1    1   5
Phalaenopsis-gene548227               0    0    1    0    0   1
Phalaenopsis-gene558238               0    0    1    0    0   1
Phalaenopsis-gene570898               0    0    1    1    0   2
Phalaenopsis-gene57197                1    1    1    1    1   5
Phalaenopsis-gene572029               1    0    0    1    0   2
Phalaenopsis-gene593073               1    1    1    0    0   3
Phalaenopsis-gene595297               1    0    1    0    0   2
Phalaenopsis-gene604563               1    0    1    0    0   2
Phalaenopsis-gene60780                1    0    1    1    0   3
Phalaenopsis-gene60960                1    1    1    1    1   5
Phalaenopsis-gene61093                1    1    1    1    1   5
Phalaenopsis-gene61164                1    0    0    1    0   2
Phalaenopsis-gene612776               0    0    0    0    0   0
Phalaenopsis-gene616024               0    0    0    1    0   1
Phalaenopsis-gene629908               1    0    0    1    0   2
Phalaenopsis-gene640319               0    1    1    0    0   2
Phalaenopsis-gene640991               1    1    1    1    1   5
Phalaenopsis-gene640997               1    0    1    0    0   2
Phalaenopsis-gene677428               1    1    0    1    0   3
Phalaenopsis-gene679852               1    0    0    1    0   2
Phalaenopsis-gene682265               0    0    0    0    0   0
Phalaenopsis-gene687760               1    0    1    1    0   3
Phalaenopsis-gene68969                1    1    1    1    1   5
Phalaenopsis-gene692236               1    1    1    1    1   5
Phalaenopsis-gene694164               1    0    1    1    0   3
Phalaenopsis-gene698985               0    0    0    1    0   1
Phalaenopsis-gene706369               1    1    1    1    1   5
Phalaenopsis-gene706457               1    0    1    1    0   3
Phalaenopsis-gene717828               0    0    1    0    0   1
Phalaenopsis-gene725475               0    0    0    1    0   1
Phalaenopsis-gene733043               1    1    1    1    1   5
Phalaenopsis-gene760142               1    0    1    1    0   3
Phalaenopsis-gene767832               1    0    1    0    0   2
Phalaenopsis-gene784199               1    0    1    1    0   3
Phalaenopsis-gene79021                0    0    0    0    0   0
Phalaenopsis-gene790650               1    0    1    1    0   3
Phalaenopsis-gene794053               1    1    1    1    1   5
Phalaenopsis-gene805642               0    0    1    1    0   2
Phalaenopsis-gene816820               1    1    1    1    1   5
Phalaenopsis-gene851592               0    0    1    1    0   2
Phalaenopsis-gene857616               1    0    1    1    0   3
Phalaenopsis-gene868261               1    0    0    1    0   2
Phalaenopsis-gene872009               0    0    1    0    0   1
Phalaenopsis-gene872301               1    1    1    1    0   4
Phalaenopsis-gene895180               1    0    1    1    0   3
Phalaenopsis-gene905636               1    1    1    1    1   5
Phalaenopsis-gene96802                0    0    1    1    0   2
Phalaenopsis-gene97063                0    0    0    1    0   1
Phalaenopsis-gene123358               1    1    1    1    1   5
Phalaenopsis-gene317462               1    0    1    1    0   3
Phalaenopsis-gene317526               0    0    0    1    0   1
Phalaenopsis-gene403481               0    0    0    1    0   1
Phalaenopsis-gene536705               1    1    1    1    1   5
Phalaenopsis-gene714254               0    0    0    1    0   1
Phalaenopsis-gene726785               1    0    0    1    0   2
Phalaenopsis-gene17907                0    0    0    0    1   1
Phalaenopsis-gene36876                1    0    0    0    1   2
Phalaenopsis-gene403242               1    1    1    1    1   5
Phalaenopsis-gene547247               1    1    1    1    1   5
Phalaenopsis-gene560625               1    1    1    1    1   5
Phalaenopsis-gene817570               1    0    0    1    0   2
Phalaenopsis-gene824884               1    1    1    1    1   5
Phalaenopsis-gene859283               1    0    1    1    0   3
Phalaenopsis-gene108040               1    1    0    1    0   3
Phalaenopsis-gene110694               1    0    1    0    0   2
Phalaenopsis-gene116639               1    0    0    1    0   2
Phalaenopsis-gene116640               1    1    1    1    1   5
Phalaenopsis-gene127758               1    1    1    1    1   5
Phalaenopsis-gene134527               1    1    1    1    1   5
Phalaenopsis-gene146204               0    0    1    1    0   2
Phalaenopsis-gene156058               1    1    1    1    1   5
Phalaenopsis-gene156992               1    0    1    0    0   2
Phalaenopsis-gene163756               1    0    0    1    0   2
Phalaenopsis-gene166695               0    0    0    0    0   0
Phalaenopsis-gene166825               1    0    1    0    0   2
Phalaenopsis-gene166884               1    1    1    1    1   5
Phalaenopsis-gene170991               0    0    1    1    0   2
Phalaenopsis-gene172035               1    0    1    1    0   3
Phalaenopsis-gene172116               0    0    1    1    0   2
Phalaenopsis-gene174055               1    0    0    1    0   2
Phalaenopsis-gene183080               1    1    1    1    1   5
Phalaenopsis-gene183192               1    1    1    1    1   5
Phalaenopsis-gene188753               1    1    1    1    1   5
Phalaenopsis-gene189134               1    1    1    1    1   5
Phalaenopsis-gene202279               1    0    0    1    0   2
Phalaenopsis-gene233144               1    0    1    1    0   3
Phalaenopsis-gene233165               1    0    1    1    0   3
Phalaenopsis-gene243613               1    0    1    1    0   3
Phalaenopsis-gene244873               1    1    1    1    1   5
Phalaenopsis-gene245584               1    0    1    0    0   2
Phalaenopsis-gene258424               1    1    1    1    1   5
Phalaenopsis-gene258713               0    0    1    1    0   2
Phalaenopsis-gene266999               1    0    1    0    0   2
Phalaenopsis-gene27072                0    0    1    1    0   2
Phalaenopsis-gene279793               1    0    0    0    0   1
Phalaenopsis-gene28447                1    1    1    1    0   4
Phalaenopsis-gene289312               1    0    1    1    0   3
Phalaenopsis-gene292537               0    0    1    0    0   1
Phalaenopsis-gene299781               1    0    1    1    0   3
Phalaenopsis-gene307477               1    1    1    0    0   3
Phalaenopsis-gene309476               0    0    1    1    0   2
Phalaenopsis-gene309947               1    0    1    0    0   2
Phalaenopsis-gene320120               1    1    1    1    1   5
Phalaenopsis-gene320144               1    0    0    1    0   2
Phalaenopsis-gene320168               1    0    1    0    0   2
Phalaenopsis-gene325561               1    0    1    1    0   3
Phalaenopsis-gene345044               1    1    1    1    1   5
Phalaenopsis-gene345058               1    0    0    0    0   1
Phalaenopsis-gene347873               1    1    1    1    1   5
Phalaenopsis-gene348396               1    1    0    1    0   3
Phalaenopsis-gene351337               1    0    1    1    0   3
Phalaenopsis-gene361709               1    0    0    1    0   2
Phalaenopsis-gene361796               1    0    0    0    0   1
Phalaenopsis-gene364788               0    0    0    0    0   0
Phalaenopsis-gene36762                1    0    1    1    0   3
Phalaenopsis-gene36838                0    0    1    1    0   2
Phalaenopsis-gene372159               1    1    1    1    1   5
Phalaenopsis-gene387277               0    1    1    1    0   3
Phalaenopsis-gene409919               1    0    0    0    0   1
Phalaenopsis-gene410940               1    1    1    1    1   5
Phalaenopsis-gene423804               0    0    1    1    0   2
Phalaenopsis-gene425261               1    1    1    1    1   5
Phalaenopsis-gene425539               1    0    0    1    0   2
Phalaenopsis-gene432335               1    0    1    1    0   3
Phalaenopsis-gene432907               0    0    1    0    0   1
Phalaenopsis-gene432966               1    0    1    1    0   3
Phalaenopsis-gene433670               0    0    1    1    0   2
Phalaenopsis-gene438947               1    0    0    0    0   1
Phalaenopsis-gene44326                1    1    1    1    1   5
Phalaenopsis-gene451704               0    0    0    0    0   0
Phalaenopsis-gene460462               1    0    1    1    0   3
Phalaenopsis-gene470239               1    1    1    1    1   5
Phalaenopsis-gene471623               1    0    0    0    0   1
Phalaenopsis-gene482167               1    0    0    1    0   2
Phalaenopsis-gene482604               1    0    0    0    0   1
Phalaenopsis-gene492354               1    1    1    1    1   5
Phalaenopsis-gene504432               0    0    1    1    0   2
Phalaenopsis-gene508535               1    0    0    1    0   2
Phalaenopsis-gene520721               1    1    1    1    1   5
Phalaenopsis-gene520887               1    1    1    1    1   5
Phalaenopsis-gene522543               1    0    1    1    0   3
Phalaenopsis-gene523072               1    1    1    1    1   5
Phalaenopsis-gene523123               0    0    0    1    0   1
Phalaenopsis-gene523462               1    0    1    1    0   3
Phalaenopsis-gene523495               1    0    0    1    0   2
Phalaenopsis-gene533159               1    1    1    1    1   5
Phalaenopsis-gene539541               1    1    1    1    1   5
Phalaenopsis-gene545606               1    0    1    0    0   2
Phalaenopsis-gene545610               1    0    0    1    0   2
Phalaenopsis-gene547109               1    1    1    1    1   5
Phalaenopsis-gene549250               1    0    1    1    0   3
Phalaenopsis-gene551723               1    0    0    1    0   2
Phalaenopsis-gene551858               1    0    1    0    0   2
Phalaenopsis-gene556792               1    1    1    1    1   5
Phalaenopsis-gene556809               1    0    0    0    0   1
Phalaenopsis-gene557536               1    1    1    1    1   5
Phalaenopsis-gene563027               1    0    1    0    0   2
Phalaenopsis-gene563095               1    1    1    1    1   5
Phalaenopsis-gene563188               1    1    1    1    1   5
Phalaenopsis-gene579491               1    0    0    1    0   2
Phalaenopsis-gene592646               1    1    1    1    1   5
Phalaenopsis-gene593030               1    0    1    1    0   3
Phalaenopsis-gene609282               1    0    1    1    0   3
Phalaenopsis-gene609283               1    1    1    1    1   5
Phalaenopsis-gene615962               1    1    1    1    1   5
Phalaenopsis-gene615966               1    0    1    0    0   2
Phalaenopsis-gene625550               1    1    1    1    1   5
Phalaenopsis-gene627918               0    0    1    0    0   1
Phalaenopsis-gene628688               1    0    0    1    0   2
Phalaenopsis-gene629664               1    1    0    0    0   2
Phalaenopsis-gene629692               1    0    1    0    1   3
Phalaenopsis-gene665233               1    0    0    1    0   2
Phalaenopsis-gene668703               0    0    0    1    0   1
Phalaenopsis-gene67368                1    1    1    1    1   5
Phalaenopsis-gene676369               1    0    0    1    0   2
Phalaenopsis-gene676442               1    0    0    0    0   1
Phalaenopsis-gene676955               1    0    1    1    0   3
Phalaenopsis-gene680728               0    0    0    0    0   0
Phalaenopsis-gene706395               1    0    0    1    0   2
Phalaenopsis-gene714275               1    1    1    1    1   5
Phalaenopsis-gene715312               1    0    1    1    0   3
Phalaenopsis-gene735140               0    0    0    0    0   0
Phalaenopsis-gene75764                1    0    1    1    0   3
Phalaenopsis-gene75766                1    0    1    1    0   3
Phalaenopsis-gene786146               1    1    1    1    1   5
Phalaenopsis-gene794275               0    0    1    1    0   2
Phalaenopsis-gene803462               1    0    0    0    0   1
Phalaenopsis-gene817662               1    0    1    0    0   2
Phalaenopsis-gene823735               1    0    0    1    0   2
Phalaenopsis-gene825730               1    1    1    1    1   5
Phalaenopsis-gene831917               1    1    1    0    0   3
Phalaenopsis-gene833738               1    1    1    1    1   5
Phalaenopsis-gene836713               1    0    0    1    0   2
Phalaenopsis-gene841141               1    1    1    1    1   5
Phalaenopsis-gene857702               1    0    0    1    0   2
Phalaenopsis-gene865568               1    0    1    1    0   3
Phalaenopsis-gene872083               1    0    1    1    0   3
Phalaenopsis-gene882574               0    0    1    1    0   2
Phalaenopsis-gene895278               1    1    1    1    1   5
Phalaenopsis-gene904594               0    0    1    0    0   1
Phalaenopsis-gene95566                1    0    0    1    0   2
Phalaenopsis-gene96977                1    0    0    1    0   2
Phalaenopsis-gene155731               0    0    1    1    0   2
Phalaenopsis-gene335932               1    1    1    1    1   5
Phalaenopsis-gene635573               1    1    1    1    1   5
Phalaenopsis-gene763728               1    1    0    1    0   3
Phalaenopsis-gene856579               1    1    1    1    1   5
Phalaenopsis-gene239693               0    0    1    0    0   1
Phalaenopsis-gene248162               1    1    1    1    1   5
Phalaenopsis-gene322357               0    0    0    1    0   1
Phalaenopsis-gene369415               1    0    1    1    0   3
Phalaenopsis-gene38404                1    0    0    1    0   2
Phalaenopsis-gene469227               1    0    0    1    0   2
Phalaenopsis-gene907577               0    0    0    1    0   1
Phalaenopsis-gene214101               1    1    1    1    1   5
Phalaenopsis-gene25805                0    1    0    0    0   1
Phalaenopsis-gene326654               1    0    1    1    0   3
Phalaenopsis-gene520712               1    1    1    1    1   5
Phalaenopsis-gene643365               1    1    1    1    0   4
Phalaenopsis-gene690052               1    0    0    1    0   2
Phalaenopsis-gene702921               1    1    1    1    1   5
Phalaenopsis-gene734406               1    0    1    0    0   2
Phalaenopsis-gene12015                1    0    1    1    0   3
Phalaenopsis-gene125356               1    0    1    0    1   3
Phalaenopsis-gene133164               0    0    0    0    0   0
Phalaenopsis-gene150109               0    0    0    1    0   1
Phalaenopsis-gene150187               0    1    0    1    0   2
Phalaenopsis-gene151240               1    0    1    1    0   3
Phalaenopsis-gene154102               1    0    1    1    0   3
Phalaenopsis-gene172212               0    1    1    1    0   3
Phalaenopsis-gene189075               1    1    1    1    1   5
Phalaenopsis-gene199477               1    1    1    1    1   5
Phalaenopsis-gene223201               1    1    1    1    0   4
Phalaenopsis-gene232324               1    1    1    1    1   5
Phalaenopsis-gene23680                1    1    0    1    0   3
Phalaenopsis-gene23701                1    0    1    1    0   3
Phalaenopsis-gene243617               0    0    0    1    0   1
Phalaenopsis-gene256092               1    0    0    1    0   2
Phalaenopsis-gene260035               1    0    0    1    0   2
Phalaenopsis-gene281346               1    0    1    0    0   2
Phalaenopsis-gene282140               0    0    1    1    0   2
Phalaenopsis-gene297297               1    0    0    1    0   2
Phalaenopsis-gene298350               1    0    1    1    0   3
Phalaenopsis-gene309637               0    0    1    1    0   2
Phalaenopsis-gene319674               0    0    1    1    0   2
Phalaenopsis-gene319796               1    1    1    1    1   5
Phalaenopsis-gene335880               1    0    0    0    0   1
Phalaenopsis-gene344324               1    1    1    1    1   5
Phalaenopsis-gene344770               1    0    0    0    0   1
Phalaenopsis-gene345041               1    1    1    1    1   5
Phalaenopsis-gene370883               1    0    0    1    0   2
Phalaenopsis-gene370956               0    1    1    1    0   3
Phalaenopsis-gene37266                0    0    0    1    0   1
Phalaenopsis-gene375444               1    0    0    0    1   2
Phalaenopsis-gene376069               1    0    1    1    0   3
Phalaenopsis-gene384556               0    0    1    1    0   2
Phalaenopsis-gene410146               1    1    1    1    1   5
Phalaenopsis-gene432800               0    1    1    0    0   2
Phalaenopsis-gene437890               1    1    1    1    1   5
Phalaenopsis-gene444141               1    0    0    1    0   2
Phalaenopsis-gene456984               1    1    1    1    1   5
Phalaenopsis-gene486043               0    0    1    0    0   1
Phalaenopsis-gene522340               0    0    1    0    0   1
Phalaenopsis-gene522514               1    0    1    1    0   3
Phalaenopsis-gene531069               1    0    1    1    0   3
Phalaenopsis-gene543758               1    0    0    1    0   2
Phalaenopsis-gene547091               0    0    1    1    0   2
Phalaenopsis-gene551470               0    0    1    1    0   2
Phalaenopsis-gene556695               0    0    0    0    0   0
Phalaenopsis-gene557479               1    0    1    1    0   3
Phalaenopsis-gene563281               1    0    0    1    0   2
Phalaenopsis-gene56454                1    1    1    1    1   5
Phalaenopsis-gene592052               0    0    0    1    0   1
Phalaenopsis-gene596508               1    1    1    1    1   5
Phalaenopsis-gene605257               0    0    1    1    0   2
Phalaenopsis-gene609353               1    1    1    1    1   5
Phalaenopsis-gene615965               1    0    1    1    0   3
Phalaenopsis-gene616972               1    0    0    0    0   1
Phalaenopsis-gene632033               1    1    1    1    1   5
Phalaenopsis-gene651096               1    0    0    1    0   2
Phalaenopsis-gene662660               1    0    0    1    0   2
Phalaenopsis-gene662846               1    1    1    1    1   5
Phalaenopsis-gene663831               1    1    1    1    1   5
Phalaenopsis-gene670521               0    0    1    0    0   1
Phalaenopsis-gene684267               1    0    1    1    0   3
Phalaenopsis-gene685678               1    1    1    1    1   5
Phalaenopsis-gene690131               1    0    1    0    0   2
Phalaenopsis-gene764277               1    1    1    1    1   5
Phalaenopsis-gene774325               1    1    1    1    1   5
Phalaenopsis-gene790944               1    0    1    1    0   3
Phalaenopsis-gene794276               1    1    1    1    1   5
Phalaenopsis-gene794962               0    0    0    1    0   1
Phalaenopsis-gene814612               0    0    1    0    0   1
Phalaenopsis-gene819678               1    1    1    1    0   4
Phalaenopsis-gene876514               0    0    1    1    0   2
Phalaenopsis-gene882586               0    0    1    1    0   2
Phalaenopsis-gene889539               0    0    0    1    0   1
Phalaenopsis-gene905163               1    0    0    1    0   2
Phalaenopsis-gene106763               1    0    1    0    0   2
Phalaenopsis-gene129417               1    1    1    1    1   5
Phalaenopsis-gene143582               0    0    0    0    0   0
Phalaenopsis-gene148916               1    1    1    1    1   5
Phalaenopsis-gene164219               0    0    0    1    0   1
Phalaenopsis-gene187917               1    1    1    1    1   5
Phalaenopsis-gene197935               0    0    1    0    0   1
Phalaenopsis-gene202849               1    0    1    1    0   3
Phalaenopsis-gene22920                1    1    1    1    1   5
Phalaenopsis-gene2366                 1    1    1    1    1   5
Phalaenopsis-gene250179               1    0    0    1    0   2
Phalaenopsis-gene250368               1    0    1    1    0   3
Phalaenopsis-gene253798               0    0    0    0    0   0
Phalaenopsis-gene281359               1    0    1    1    0   3
Phalaenopsis-gene301869               0    0    1    1    0   2
Phalaenopsis-gene302661               1    1    1    1    1   5
Phalaenopsis-gene320086               0    0    1    1    0   2
Phalaenopsis-gene324592               0    0    0    0    0   0
Phalaenopsis-gene339364               1    1    1    1    1   5
Phalaenopsis-gene339732               1    0    1    0    0   2
Phalaenopsis-gene349628               1    0    0    1    0   2
Phalaenopsis-gene351877               1    1    1    1    1   5
Phalaenopsis-gene360645               1    0    1    1    0   3
Phalaenopsis-gene36572                0    0    0    0    0   0
Phalaenopsis-gene381634               1    1    1    1    1   5
Phalaenopsis-gene384542               1    1    1    1    1   5
Phalaenopsis-gene413953               1    0    0    0    0   1
Phalaenopsis-gene423720               1    0    0    1    0   2
Phalaenopsis-gene432857               0    0    1    1    0   2
Phalaenopsis-gene440012               1    1    1    1    1   5
Phalaenopsis-gene440221               1    1    1    1    1   5
Phalaenopsis-gene446786               1    1    1    1    1   5
Phalaenopsis-gene46                   1    1    1    1    1   5
Phalaenopsis-gene508016               1    1    1    1    1   5
Phalaenopsis-gene508546               1    1    1    1    1   5
Phalaenopsis-gene51341                1    0    0    1    0   2
Phalaenopsis-gene522335               0    0    1    1    0   2
Phalaenopsis-gene534059               1    0    0    0    0   1
Phalaenopsis-gene539551               1    1    1    1    1   5
Phalaenopsis-gene54222                1    0    0    1    0   2
Phalaenopsis-gene547842               0    0    1    1    0   2
Phalaenopsis-gene560447               1    1    1    1    1   5
Phalaenopsis-gene566033               1    0    0    1    0   2
Phalaenopsis-gene571160               0    0    1    1    0   2
Phalaenopsis-gene598118               1    0    1    1    0   3
Phalaenopsis-gene615972               1    0    1    0    0   2
Phalaenopsis-gene617851               0    0    0    1    1   2
Phalaenopsis-gene625068               0    0    1    1    0   2
Phalaenopsis-gene658919               1    1    1    1    1   5
Phalaenopsis-gene662906               1    0    1    1    0   3
Phalaenopsis-gene67400                0    0    0    1    0   1
Phalaenopsis-gene693473               0    0    1    0    0   1
Phalaenopsis-gene715128               1    1    1    1    1   5
Phalaenopsis-gene723898               1    1    1    1    1   5
Phalaenopsis-gene747891               1    1    1    1    0   4
Phalaenopsis-gene754293               1    1    0    1    0   3
Phalaenopsis-gene754645               1    0    0    1    0   2
Phalaenopsis-gene768593               0    1    1    1    0   3
Phalaenopsis-gene771747               1    1    1    1    0   4
Phalaenopsis-gene789588               1    1    1    1    1   5
Phalaenopsis-gene839744               1    0    1    0    0   2
Phalaenopsis-gene862348               1    0    0    1    0   2
Phalaenopsis-gene887111               1    0    0    1    0   2
Phalaenopsis-gene89307                0    0    1    1    0   2
Phalaenopsis-gene97147                0    0    1    1    0   2
Phalaenopsis-gene679287               1    1    1    1    1   5
Phalaenopsis-gene120526               1    1    1    1    1   5
Phalaenopsis-gene124813               1    0    1    1    0   3
Phalaenopsis-gene128913               1    0    1    1    0   3
Phalaenopsis-gene132965               1    1    1    1    1   5
Phalaenopsis-gene135385               1    0    1    0    0   2
Phalaenopsis-gene136146               1    0    1    0    0   2
Phalaenopsis-gene141031               1    1    1    1    1   5
Phalaenopsis-gene150197               1    0    0    1    0   2
Phalaenopsis-gene151661               1    0    1    1    0   3
Phalaenopsis-gene201617               1    0    0    1    0   2
Phalaenopsis-gene23023                1    1    1    1    1   5
Phalaenopsis-gene244708               1    0    0    1    0   2
Phalaenopsis-gene247723               1    0    1    1    0   3
Phalaenopsis-gene297282               1    1    1    1    1   5
Phalaenopsis-gene300880               1    1    0    1    0   3
Phalaenopsis-gene319650               0    0    0    1    0   1
Phalaenopsis-gene320169               1    0    1    0    0   2
Phalaenopsis-gene320227               1    1    0    1    0   3
Phalaenopsis-gene32151                0    0    1    1    0   2
Phalaenopsis-gene32195                1    1    1    1    1   5
Phalaenopsis-gene342314               1    0    1    1    0   3
Phalaenopsis-gene345088               1    0    1    1    0   3
Phalaenopsis-gene349637               1    0    1    1    0   3
Phalaenopsis-gene359858               1    1    1    1    1   5
Phalaenopsis-gene363232               1    1    1    1    1   5
Phalaenopsis-gene36429                1    0    0    1    0   2
Phalaenopsis-gene39877                1    0    1    0    0   2
Phalaenopsis-gene414188               1    0    1    1    0   3
Phalaenopsis-gene432912               0    0    1    0    0   1
Phalaenopsis-gene448132               1    1    1    1    0   4
Phalaenopsis-gene484242               1    1    1    1    1   5
Phalaenopsis-gene503875               0    0    0    1    0   1
Phalaenopsis-gene505360               1    0    0    0    0   1
Phalaenopsis-gene513690               1    0    1    1    0   3
Phalaenopsis-gene526732               1    1    1    1    1   5
Phalaenopsis-gene528826               1    1    1    1    1   5
Phalaenopsis-gene530313               1    0    1    1    0   3
Phalaenopsis-gene533571               1    1    1    1    1   5
Phalaenopsis-gene556688               1    1    1    1    1   5
Phalaenopsis-gene556812               1    0    1    1    0   3
Phalaenopsis-gene561557               1    0    0    0    1   2
Phalaenopsis-gene592872               1    1    1    1    1   5
Phalaenopsis-gene593114               1    0    0    0    0   1
Phalaenopsis-gene594292               1    1    1    1    1   5
Phalaenopsis-gene620923               1    1    1    1    1   5
Phalaenopsis-gene630747               1    0    0    0    0   1
Phalaenopsis-gene635866               1    1    1    1    1   5
Phalaenopsis-gene640315               0    1    0    0    0   1
Phalaenopsis-gene644315               1    1    1    1    1   5
Phalaenopsis-gene663276               1    0    1    1    0   3
Phalaenopsis-gene677323               1    1    1    1    1   5
Phalaenopsis-gene678684               1    1    1    1    1   5
Phalaenopsis-gene682138               1    0    0    0    0   1
Phalaenopsis-gene706439               0    0    0    0    0   0
Phalaenopsis-gene716129               1    0    1    1    0   3
Phalaenopsis-gene731508               1    0    1    1    0   3
Phalaenopsis-gene757733               1    1    1    1    1   5
Phalaenopsis-gene764036               1    1    1    1    1   5
Phalaenopsis-gene766207               1    0    0    1    0   2
Phalaenopsis-gene789591               1    0    1    0    0   2
Phalaenopsis-gene797205               1    0    0    0    0   1
Phalaenopsis-gene807157               1    1    1    1    1   5
Phalaenopsis-gene820049               1    0    0    1    0   2
Phalaenopsis-gene83821                1    1    0    1    0   3
Phalaenopsis-gene876736               1    1    1    1    1   5
Phalaenopsis-gene882069               1    1    0    1    0   3
Phalaenopsis-gene888265               1    0    0    0    0   1
Phalaenopsis-gene906861               1    0    1    1    0   3
Phalaenopsis-gene403998               1    0    1    0    0   2
Phalaenopsis-gene487880               1    0    0    1    0   2
Phalaenopsis-gene716164               1    0    0    0    0   1
Phalaenopsis-gene799783               1    0    0    0    0   1
Phalaenopsis-gene181705               1    0    1    1    0   3
Phalaenopsis-gene200763               1    0    0    1    0   2
Phalaenopsis-gene357647               0    0    0    0    0   0
Phalaenopsis-gene526497               1    1    1    1    1   5
Phalaenopsis-gene558183               1    0    0    1    0   2
Phalaenopsis-gene795002               1    0    1    1    0   3
Phalaenopsis-gene83913                1    0    0    1    0   2
Phalaenopsis-gene96621                1    0    1    0    0   2
Phalaenopsis-gene573135               1    0    0    1    0   2
Phalaenopsis-gene631486               1    0    0    1    0   2
Ophrys-gene24160                      0    0    1    1    0   2
Ophrys-gene39279                      1    0    0    0    0   1
Ophrys-gene54186                      1    0    0    1    0   2
Ophrys-gene55656                      1    1    1    1    1   5
Ophrys-gene77255                      1    0    1    1    0   3
Ophrys-gene100838                     1    1    1    1    1   5
Ophrys-gene131898                     1    1    1    1    1   5
Ophrys-gene139688                     1    1    1    1    1   5
Ophrys-gene157180                     1    0    0    1    0   2
Ophrys-gene176248                     1    0    0    1    0   2
Ophrys-gene212076                     1    0    0    1    0   2
Ophrys-gene215346                     1    1    1    1    1   5
Ophrys-gene229480                     1    0    0    1    0   2
Ophrys-gene230322                     1    1    1    1    1   5
Ophrys-gene277969                     1    1    1    1    1   5
Ophrys-gene283706                     1    0    0    1    0   2
Ophrys-gene297852                     1    0    0    1    0   2
Ophrys-gene304413                     1    1    1    1    1   5
Ophrys-gene304867                     1    0    1    0    0   2
Ophrys-gene356177                     1    1    1    1    1   5
Ophrys-gene375169                     1    0    0    1    0   2
Ophrys-gene451806                     1    1    1    1    1   5
Ophrys-gene462503                     1    0    1    1    0   3
Ophrys-gene476820                     1    0    0    1    0   2
Ophrys-gene488931                     1    1    1    1    1   5
Ophrys-gene490073                     1    0    0    1    0   2
Ophrys-gene537560                     0    0    0    0    0   0
Ophrys-gene567289                     1    1    1    1    1   5
Ophrys-gene583015                     0    0    0    0    0   0
Ophrys-gene586781                     1    0    1    0    0   2
Ophrys-gene638786                     1    0    1    1    0   3
Ophrys-gene661338                     1    1    1    0    0   3
Ophrys-gene697781                     1    0    0    1    0   2
Ophrys-gene5670                       1    0    0    1    0   2
Ophrys-gene62145                      1    1    1    1    1   5
Ophrys-gene206541                     1    1    1    1    1   5
Ophrys-gene538785                     1    0    1    1    0   3
Ophrys-gene566212                     0    0    1    0    0   1
Ophrys-gene708725                     1    1    1    1    1   5
Ophrys-gene717313                     0    0    0    1    0   1
Ophrys-gene156399                     1    0    1    0    0   2
Ophrys-gene399278                     1    1    1    1    1   5
Ophrys-gene430980                     0    1    1    1    0   3
Ophrys-gene9036                       1    1    1    1    1   5
Ophrys-gene10716                      1    1    1    1    1   5
Ophrys-gene57802                      1    0    1    0    0   2
Ophrys-gene69728                      1    0    0    1    1   3
Ophrys-gene75303                      1    1    1    1    1   5
Ophrys-gene126481                     1    1    0    1    0   3
Ophrys-gene143573                     1    0    0    1    0   2
Ophrys-gene145225                     1    0    1    1    0   3
Ophrys-gene167718                     1    0    1    1    0   3
Ophrys-gene173611                     1    1    1    1    1   5
Ophrys-gene231628                     0    1    1    0    0   2
Ophrys-gene265725                     1    0    0    0    0   1
Ophrys-gene268602                     1    0    0    0    0   1
Ophrys-gene271113                     1    0    0    1    0   2
Ophrys-gene271584                     1    1    1    1    1   5
Ophrys-gene284626                     1    0    1    1    0   3
Ophrys-gene295682                     1    1    1    1    1   5
Ophrys-gene375009                     1    0    0    1    0   2
Ophrys-gene383155                     1    0    0    0    0   1
Ophrys-gene393491                     0    0    0    1    0   1
Ophrys-gene395195                     1    1    1    1    0   4
Ophrys-gene400016                     1    1    1    1    1   5
Ophrys-gene437529                     1    1    1    1    1   5
Ophrys-gene490072                     0    1    1    0    0   2
Ophrys-gene494094                     1    1    1    1    1   5
Ophrys-gene508453                     1    1    0    0    0   2
Ophrys-gene528196                     1    0    0    1    0   2
Ophrys-gene608078                     1    1    1    1    1   5
Ophrys-gene628393                     0    0    0    1    0   1
Ophrys-gene640145                     1    0    0    1    0   2
Ophrys-gene689682                     1    0    1    1    0   3
Ophrys-gene693538                     1    0    1    1    0   3
Ophrys-gene695853                     1    1    0    1    0   3
Ophrys-gene702664                     1    1    1    1    1   5
Ophrys-gene140874                     1    0    0    1    0   2
Ophrys-gene140981                     0    0    0    0    0   0
Ophrys-gene154978                     1    0    1    1    0   3
Ophrys-gene304187                     0    0    1    0    0   1
Ophrys-gene318998                     1    1    1    1    1   5
Ophrys-gene731520                     1    0    0    1    0   2
Ophrys-gene731980                     1    0    0    1    0   2
Ophrys-gene290863                     1    0    0    1    0   2
Ophrys-gene673502                     1    0    0    1    0   2
Ophrys-gene1838                       1    0    0    1    0   2
Ophrys-gene24979                      1    1    1    1    1   5
Ophrys-gene75062                      1    1    1    1    1   5
Ophrys-gene77032                      1    0    0    1    0   2
Ophrys-gene80183                      1    1    0    1    0   3
Ophrys-gene113703                     1    0    0    1    0   2
Ophrys-gene132443                     1    1    0    0    0   2
Ophrys-gene155336                     1    1    1    1    1   5
Ophrys-gene173242                     1    0    0    1    0   2
Ophrys-gene183712                     1    0    0    1    0   2
Ophrys-gene195080                     0    0    1    1    0   2
Ophrys-gene210786                     1    1    1    1    1   5
Ophrys-gene263434                     0    0    0    0    0   0
Ophrys-gene271634                     0    0    0    1    0   1
Ophrys-gene271956                     1    0    0    0    0   1
Ophrys-gene299724                     0    1    1    1    0   3
Ophrys-gene304865                     1    0    1    1    0   3
Ophrys-gene386156                     0    0    0    1    0   1
Ophrys-gene404713                     0    0    0    1    0   1
Ophrys-gene409887                     1    0    0    0    0   1
Ophrys-gene430223                     1    0    1    0    0   2
Ophrys-gene434505                     1    0    1    0    0   2
Ophrys-gene439160                     1    0    1    0    0   2
Ophrys-gene508802                     1    1    1    1    1   5
Ophrys-gene525981                     1    0    1    1    0   3
Ophrys-gene546686                     1    1    1    1    1   5
Ophrys-gene566947                     1    0    1    0    0   2
Ophrys-gene567013                     1    1    1    1    1   5
Ophrys-gene567913                     1    0    1    1    0   3
Ophrys-gene577091                     1    0    1    0    0   2
Ophrys-gene612426                     1    1    1    1    0   4
Ophrys-gene658347                     1    1    1    1    1   5
Ophrys-gene677162                     1    0    1    1    0   3
Ophrys-gene697145                     1    0    0    1    0   2
Ophrys-gene697374                     1    1    1    1    1   5
Ophrys-gene710697                     1    0    1    0    0   2
Ophrys-gene726741                     1    0    0    1    0   2
Ophrys-gene739693                     1    1    1    1    1   5
Ophrys-gene110494                     1    1    1    1    1   5
Ophrys-gene207941                     1    1    1    1    1   5
Ophrys-gene681949                     1    1    1    1    1   5
Ophrys-gene793                        1    1    1    1    1   5
Ophrys-gene9050                       1    0    0    1    0   2
Ophrys-gene47387                      1    0    0    0    0   1
Ophrys-gene140934                     1    0    0    0    0   1
Ophrys-gene239558                     1    0    0    1    0   2
Ophrys-gene303829                     1    0    1    1    1   4
Ophrys-gene748141                     1    1    1    1    1   5
Ophrys-gene141679                     0    1    0    1    0   2
Ophrys-gene144735                     1    1    1    1    1   5
Ophrys-gene196111                     0    0    1    1    0   2
Ophrys-gene203610                     1    0    1    1    0   3
Ophrys-gene213083                     1    0    1    1    0   3
Ophrys-gene238847                     1    0    1    1    0   3
Ophrys-gene258720                     1    0    1    0    0   2
Ophrys-gene258783                     0    0    0    1    0   1
Ophrys-gene263959                     0    1    0    0    0   1
Ophrys-gene289127                     1    0    0    1    0   2
Ophrys-gene353382                     1    1    1    1    1   5
Ophrys-gene370049                     1    1    1    1    1   5
Ophrys-gene375124                     1    0    0    0    0   1
Ophrys-gene478180                     1    1    1    1    1   5
Ophrys-gene493848                     1    1    1    1    0   4
Ophrys-gene580004                     1    0    0    1    0   2
Ophrys-gene609399                     1    0    0    1    0   2
Ophrys-gene693100                     0    0    0    1    0   1
Ophrys-gene708009                     1    1    1    1    1   5
Ophrys-gene709670                     1    0    0    1    0   2
Ophrys-gene47420                      1    1    1    1    1   5
Ophrys-gene93956                      1    0    0    0    0   1
Ophrys-gene339383                     1    1    1    1    1   5
Ophrys-gene344537                     1    0    1    0    0   2
Ophrys-gene504467                     1    0    0    1    0   2
Ophrys-gene569495                     1    0    0    1    0   2
Ophrys-gene587170                     1    1    1    1    1   5
Ophrys-gene65760                      1    1    1    1    1   5
Ophrys-gene113688                     1    1    1    1    1   5
Ophrys-gene421016                     0    0    1    1    0   2
Ophrys-gene483836                     1    0    1    0    0   2
Ophrys-gene656917                     1    1    1    1    1   5
Ophrys-gene731737                     1    0    0    1    0   2
Ophrys-gene4964                       1    1    1    1    1   5
Ophrys-gene10049                      1    1    1    1    1   5
Ophrys-gene22537                      1    1    1    1    1   5
Ophrys-gene57605                      0    0    1    0    0   1
Ophrys-gene63208                      1    1    1    1    1   5
Ophrys-gene100045                     1    1    1    1    1   5
Ophrys-gene114866                     1    1    1    1    1   5
Ophrys-gene117386                     1    1    1    1    1   5
Ophrys-gene123770                     1    1    0    0    0   2
Ophrys-gene154921                     1    0    0    1    0   2
Ophrys-gene181429                     1    1    1    1    1   5
Ophrys-gene246641                     1    0    1    1    0   3
Ophrys-gene264043                     1    1    1    1    1   5
Ophrys-gene271472                     0    0    0    0    0   0
Ophrys-gene274366                     1    0    0    1    0   2
Ophrys-gene366721                     0    0    1    1    0   2
Ophrys-gene368597                     1    0    1    0    0   2
Ophrys-gene384416                     1    0    1    1    0   3
Ophrys-gene408975                     1    1    1    1    1   5
Ophrys-gene586925                     0    0    1    1    0   2
Ophrys-gene646681                     1    1    1    1    1   5
Ophrys-gene726143                     0    1    0    0    0   1
Ophrys-gene23375                      1    1    1    1    1   5
Ophrys-gene52111                      1    0    0    0    0   1
Ophrys-gene60567                      1    0    0    1    0   2
Ophrys-gene85396                      1    0    1    1    0   3
Ophrys-gene289112                     0    0    1    1    0   2
Ophrys-gene296022                     0    0    0    1    0   1
Ophrys-gene448028                     1    1    1    1    1   5
Ophrys-gene498139                     1    1    1    1    1   5
Ophrys-gene509227                     1    0    1    1    0   3
Ophrys-gene566466                     1    1    1    1    1   5
Ophrys-gene609783                     1    0    0    1    0   2
Ophrys-gene610098                     1    0    0    0    0   1
Ophrys-gene610272                     1    1    0    1    0   3
Ophrys-gene610273                     1    0    0    1    0   2
Ophrys-gene189907                     1    0    0    1    0   2
Ophrys-gene393305                     0    0    1    1    0   2
Ophrys-gene536727                     1    0    0    0    0   1
Ophrys-gene712230                     1    0    0    0    0   1
Ophrys-gene731284                     1    1    1    1    1   5
Ophrys-gene731613                     1    0    0    1    0   2
Ophrys-gene759320                     1    1    1    1    1   5
Ophrys-gene5174                       1    0    0    0    0   1
Ophrys-gene21220                      1    0    0    1    0   2
Ophrys-gene66521                      1    1    1    1    1   5
Ophrys-gene731402                     1    0    0    0    0   1
Ophrys-gene731819                     1    0    0    1    0   2
Ophrys-gene24804                      1    1    1    1    1   5
Ophrys-gene183878                     1    0    0    0    0   1
Ophrys-gene297435                     1    0    0    1    0   2
Ophrys-gene566472                     0    0    0    1    0   1
Ophrys-gene708703                     1    0    1    1    0   3
Ophrys-gene126136                     1    1    0    1    0   3
Ophrys-gene181161                     1    0    1    1    0   3
Ophrys-gene186371                     1    0    0    0    0   1
Ophrys-gene189852                     1    0    0    0    0   1
Ophrys-gene190108                     1    0    0    1    0   2
Ophrys-gene247325                     1    1    0    1    0   3
Ophrys-gene251010                     0    0    0    1    0   1
Ophrys-gene286728                     1    0    0    1    0   2
Ophrys-gene306066                     1    1    1    1    1   5
Ophrys-gene338183                     1    1    0    0    0   2
Ophrys-gene383101                     1    0    0    0    0   1
Ophrys-gene390545                     1    1    1    1    1   5
Ophrys-gene396518                     1    1    1    1    1   5
Ophrys-gene466318                     0    0    0    1    0   1
Ophrys-gene480114                     0    0    0    0    0   0
Ophrys-gene509910                     1    1    1    1    1   5
Ophrys-gene526281                     0    0    0    1    0   1
Ophrys-gene549182                     1    1    1    1    1   5
Ophrys-gene587329                     1    0    0    0    0   1
Ophrys-gene605724                     1    0    1    1    0   3
Ophrys-gene656822                     1    1    0    0    0   2
Ophrys-gene694821                     1    0    0    1    0   2
Ophrys-gene709768                     1    0    0    1    0   2
Ophrys-gene757618                     1    1    0    1    0   3
Phalaenopsis-gene122358               1    0    1    1    0   3
Phalaenopsis-gene129540               1    0    1    0    0   2
Phalaenopsis-gene156020               1    1    1    1    1   5
Phalaenopsis-gene165616               1    0    0    0    0   1
Phalaenopsis-gene214665               0    0    1    0    0   1
Phalaenopsis-gene247765               1    0    1    1    0   3
Phalaenopsis-gene249034               1    0    0    1    0   2
Phalaenopsis-gene286591               1    0    0    1    0   2
Phalaenopsis-gene293544               1    0    1    1    0   3
Phalaenopsis-gene298408               1    1    1    1    1   5
Phalaenopsis-gene324560               1    1    1    1    1   5
Phalaenopsis-gene345794               1    1    0    0    0   2
Phalaenopsis-gene354956               1    1    1    1    0   4
Phalaenopsis-gene376092               1    0    0    1    0   2
Phalaenopsis-gene430586               1    0    0    0    0   1
Phalaenopsis-gene471985               1    0    1    1    0   3
Phalaenopsis-gene486772               1    1    1    1    0   4
Phalaenopsis-gene487532               1    1    1    1    1   5
Phalaenopsis-gene487990               1    0    1    1    0   3
Phalaenopsis-gene512087               1    0    1    1    0   3
Phalaenopsis-gene549236               1    0    0    0    0   1
Phalaenopsis-gene592977               1    1    1    1    1   5
Phalaenopsis-gene594444               1    0    0    1    0   2
Phalaenopsis-gene615905               0    0    1    1    0   2
Phalaenopsis-gene662756               0    0    1    1    0   2
Phalaenopsis-gene673972               1    0    0    0    1   2
Phalaenopsis-gene676911               1    0    1    1    0   3
Phalaenopsis-gene687172               1    1    1    1    1   5
Phalaenopsis-gene694118               1    0    1    1    0   3
Phalaenopsis-gene744484               1    0    1    1    0   3
Phalaenopsis-gene754214               1    0    1    1    0   3
Phalaenopsis-gene757730               0    0    0    1    0   1
Phalaenopsis-gene766074               1    1    1    1    1   5
Phalaenopsis-gene784862               0    0    0    1    0   1
Phalaenopsis-gene808547               1    1    1    1    1   5
Phalaenopsis-gene839670               1    0    0    1    0   2
Phalaenopsis-gene887283               1    0    0    0    0   1
Phalaenopsis-gene892955               0    0    0    1    0   1
Phalaenopsis-gene96081                1    0    1    1    0   3
Phalaenopsis-gene129590               1    0    1    1    0   3
Phalaenopsis-gene162029               1    0    0    1    0   2
Phalaenopsis-gene219132               0    0    1    1    0   2
Phalaenopsis-gene256260               1    1    1    1    1   5
Phalaenopsis-gene309564               1    0    1    0    0   2
Phalaenopsis-gene402725               1    1    1    1    1   5
Phalaenopsis-gene410768               1    0    1    1    0   3
Phalaenopsis-gene566572               1    1    1    1    1   5
Phalaenopsis-gene59344                1    0    0    0    0   1
Phalaenopsis-gene62567                1    0    1    1    0   3
Phalaenopsis-gene676288               1    0    0    1    0   2
Phalaenopsis-gene676361               1    0    0    1    0   2
Phalaenopsis-gene184594               0    0    1    0    0   1
Phalaenopsis-gene318799               1    0    1    1    0   3
Phalaenopsis-gene354256               0    0    1    1    0   2
Phalaenopsis-gene354292               1    0    1    1    0   3
Phalaenopsis-gene357691               1    1    0    0    0   2
Phalaenopsis-gene369554               1    0    0    1    0   2
Phalaenopsis-gene558120               1    0    0    1    0   2
Phalaenopsis-gene82095                1    1    1    1    1   5
Phalaenopsis-gene214750               0    1    0    1    0   2
Phalaenopsis-gene234983               0    0    0    1    0   1
Phalaenopsis-gene247763               1    1    1    1    1   5
Phalaenopsis-gene325331               1    1    1    1    1   5
Phalaenopsis-gene337884               1    1    0    0    0   2
Phalaenopsis-gene364389               1    0    0    1    0   2
Phalaenopsis-gene408258               1    0    1    1    0   3
Phalaenopsis-gene430764               1    0    0    1    0   2
Phalaenopsis-gene465773               1    1    1    1    1   5
Phalaenopsis-gene503903               1    0    1    0    0   2
Phalaenopsis-gene520236               1    0    0    1    0   2
Phalaenopsis-gene547240               1    1    1    1    1   5
Phalaenopsis-gene570200               0    1    0    0    0   1
Phalaenopsis-gene60815                1    0    0    1    0   2
Phalaenopsis-gene639379               1    0    1    1    0   3
Phalaenopsis-gene649982               1    0    0    1    1   3
Phalaenopsis-gene714298               1    1    1    1    1   5
Phalaenopsis-gene740419               1    1    1    1    1   5
Phalaenopsis-gene808546               1    0    0    0    0   1
Phalaenopsis-gene357827               0    0    0    1    0   1
Phalaenopsis-gene369347               1    1    0    1    0   3
Phalaenopsis-gene430140               1    0    0    0    0   1
Phalaenopsis-gene430165               1    0    1    0    0   2
Phalaenopsis-gene625130               1    0    1    1    0   3
Phalaenopsis-gene370029               1    1    1    1    1   5
Phalaenopsis-gene370121               1    0    1    1    0   3
Phalaenopsis-gene370213               1    0    1    1    0   3
Phalaenopsis-gene615814               0    0    0    1    0   1
Phalaenopsis-gene62837                1    1    1    1    0   4
Phalaenopsis-gene725123               1    0    1    0    0   2
Phalaenopsis-gene116777               1    1    1    1    1   5
Phalaenopsis-gene129706               0    0    1    1    0   2
Phalaenopsis-gene143537               0    1    1    0    0   2
Phalaenopsis-gene145265               1    0    0    1    1   3
Phalaenopsis-gene146259               1    0    0    1    0   2
Phalaenopsis-gene203181               1    1    1    1    1   5
Phalaenopsis-gene204349               1    0    1    0    0   2
Phalaenopsis-gene214754               1    0    1    1    0   3
Phalaenopsis-gene222238               1    0    1    1    0   3
Phalaenopsis-gene247721               0    1    1    1    1   4
Phalaenopsis-gene257315               0    0    1    1    0   2
Phalaenopsis-gene270018               1    0    1    0    0   2
Phalaenopsis-gene280132               1    0    0    1    0   2
Phalaenopsis-gene289339               1    0    1    1    0   3
Phalaenopsis-gene318757               0    1    1    1    0   3
Phalaenopsis-gene320063               0    0    1    1    0   2
Phalaenopsis-gene322511               1    0    1    0    0   2
Phalaenopsis-gene326442               1    1    1    1    1   5
Phalaenopsis-gene332890               1    0    1    1    1   4
Phalaenopsis-gene339698               1    0    1    1    0   3
Phalaenopsis-gene340281               1    1    0    1    0   3
Phalaenopsis-gene348678               1    0    1    1    0   3
Phalaenopsis-gene355441               1    0    0    0    0   1
Phalaenopsis-gene357826               1    1    1    1    1   5
Phalaenopsis-gene363230               1    1    1    1    1   5
Phalaenopsis-gene372187               1    1    1    1    1   5
Phalaenopsis-gene408353               0    0    0    1    0   1
Phalaenopsis-gene413464               1    1    1    1    1   5
Phalaenopsis-gene426327               1    1    1    1    1   5
Phalaenopsis-gene449624               1    0    0    1    0   2
Phalaenopsis-gene478084               1    1    1    1    1   5
Phalaenopsis-gene485867               1    0    1    1    0   3
Phalaenopsis-gene499442               1    0    1    1    0   3
Phalaenopsis-gene510866               1    1    1    1    1   5
Phalaenopsis-gene522546               1    0    1    0    0   2
Phalaenopsis-gene529931               0    0    0    0    0   0
Phalaenopsis-gene533172               1    0    1    1    0   3
Phalaenopsis-gene551223               1    1    1    1    1   5
Phalaenopsis-gene558786               1    0    1    1    0   3
Phalaenopsis-gene573091               1    0    0    1    0   2
Phalaenopsis-gene59482                1    0    1    1    0   3
Phalaenopsis-gene630840               1    1    1    1    1   5
Phalaenopsis-gene630998               0    1    0    1    0   2
Phalaenopsis-gene651010               0    0    1    0    0   1
Phalaenopsis-gene651039               1    0    0    1    0   2
Phalaenopsis-gene658758               1    1    1    1    1   5
Phalaenopsis-gene678191               0    0    1    1    0   2
Phalaenopsis-gene679850               1    0    1    0    0   2
Phalaenopsis-gene693290               1    1    1    1    1   5
Phalaenopsis-gene728114               1    0    0    1    0   2
Phalaenopsis-gene74773                0    0    1    1    0   2
Phalaenopsis-gene747890               1    0    1    0    0   2
Phalaenopsis-gene776726               1    1    1    1    1   5
Phalaenopsis-gene792379               1    1    0    1    0   3
Phalaenopsis-gene796226               1    1    1    1    1   5
Phalaenopsis-gene887236               1    0    0    1    0   2
Phalaenopsis-gene97061                0    0    1    1    0   2
Phalaenopsis-gene364604               0    0    1    1    0   2
Phalaenopsis-gene381658               0    0    1    0    0   1
Phalaenopsis-gene588945               1    0    1    1    0   3
Phalaenopsis-gene201791               1    0    0    1    0   2
Phalaenopsis-gene357735               1    0    0    0    0   1
Phalaenopsis-gene616309               0    0    1    1    0   2
Phalaenopsis-gene626983               1    1    1    1    1   5
Phalaenopsis-gene640488               1    0    1    1    0   3
Phalaenopsis-gene653008               0    0    0    1    0   1
Phalaenopsis-gene852603               1    0    1    1    0   3
Phalaenopsis-gene137333               1    1    1    1    1   5
Phalaenopsis-gene369622               1    0    0    1    0   2
Phalaenopsis-gene567854               1    0    1    1    0   3
Phalaenopsis-gene646909               1    0    0    1    0   2
Phalaenopsis-gene786135               1    0    0    0    0   1
Phalaenopsis-gene83621                1    0    1    1    0   3
Phalaenopsis-gene189088               0    0    1    1    1   3
Phalaenopsis-gene204250               1    1    1    1    1   5
Phalaenopsis-gene204315               1    0    0    0    0   1
Phalaenopsis-gene342817               1    1    1    1    1   5
Phalaenopsis-gene349578               1    1    1    1    1   5
Phalaenopsis-gene355283               0    1    1    0    1   3
Phalaenopsis-gene432365               0    0    1    0    0   1
Phalaenopsis-gene442963               1    0    0    0    0   1
Phalaenopsis-gene44365                1    1    1    1    1   5
Phalaenopsis-gene467215               1    0    1    0    0   2
Phalaenopsis-gene522517               1    0    0    1    0   2
Phalaenopsis-gene70467                1    1    1    1    1   5
Phalaenopsis-gene784891               1    1    1    1    1   5
Phalaenopsis-gene848969               1    0    0    1    0   2
Phalaenopsis-gene908028               1    0    0    1    0   2
Phalaenopsis-gene129881               1    1    1    1    1   5
Phalaenopsis-gene129916               0    0    0    0    0   0
Phalaenopsis-gene184450               1    1    1    1    1   5
Phalaenopsis-gene762303               1    0    0    0    0   1
Phalaenopsis-gene84014                1    1    0    0    0   2
Phalaenopsis-gene164213               0    0    0    0    0   0
Phalaenopsis-gene229722               1    1    1    1    1   5
Phalaenopsis-gene230010               0    1    0    0    0   1
Phalaenopsis-gene311684               1    0    1    1    0   3
Phalaenopsis-gene663367               1    0    0    1    0   2
Phalaenopsis-gene76033                1    1    1    1    1   5
Phalaenopsis-gene814580               1    0    0    1    0   2
Phalaenopsis-gene908037               1    0    0    1    0   2
Phalaenopsis-gene124815               1    1    1    1    1   5
Phalaenopsis-gene206124               1    1    1    1    1   5
Phalaenopsis-gene266946               0    0    1    1    0   2
Phalaenopsis-gene317821               0    0    1    1    0   2
Phalaenopsis-gene326485               1    1    1    1    1   5
Phalaenopsis-gene33373                0    1    0    0    0   1
Phalaenopsis-gene363288               1    0    1    1    0   3
Phalaenopsis-gene369636               0    0    1    1    0   2
Phalaenopsis-gene442771               1    1    1    1    1   5
Phalaenopsis-gene67282                0    0    1    1    0   2
Phalaenopsis-gene807135               1    0    1    0    0   2
Phalaenopsis-gene837326               1    1    1    1    1   5
Phalaenopsis-gene90325                1    1    1    1    1   5
Phalaenopsis-gene164221               1    1    1    1    1   5
Phalaenopsis-gene186839               0    0    0    1    0   1
Phalaenopsis-gene187918               1    1    1    1    1   5
Phalaenopsis-gene198616               1    0    0    1    0   2
Phalaenopsis-gene234974               1    0    0    1    0   2
Phalaenopsis-gene23708                1    0    0    1    1   3
Phalaenopsis-gene307180               1    1    1    1    1   5
Phalaenopsis-gene308050               1    0    1    1    0   3
Phalaenopsis-gene342354               1    1    1    1    1   5
Phalaenopsis-gene408056               1    1    1    1    1   5
Phalaenopsis-gene444168               1    1    1    1    1   5
Phalaenopsis-gene531078               1    0    1    0    0   2
Phalaenopsis-gene657922               1    0    1    1    0   3
Phalaenopsis-gene658039               1    0    1    1    0   3
Phalaenopsis-gene68392                1    1    1    1    1   5
Phalaenopsis-gene723895               1    0    1    0    0   2
Phalaenopsis-gene734297               1    0    0    0    0   1
Phalaenopsis-gene744199               0    0    1    1    0   2
Phalaenopsis-gene790765               1    1    1    1    1   5
Phalaenopsis-gene792639               0    0    1    1    0   2
Phalaenopsis-gene827243               1    0    0    1    0   2
Phalaenopsis-gene827634               1    1    1    1    1   5
Phalaenopsis-gene868292               1    1    1    1    1   5
Phalaenopsis-gene159355               1    1    1    1    1   5
Phalaenopsis-gene235682               1    1    1    1    1   5
Phalaenopsis-gene369484               1    0    1    0    0   2
Phalaenopsis-gene476339               1    1    1    1    0   4
Phalaenopsis-gene763672               1    0    1    0    0   2
Phalaenopsis-gene77882                0    0    0    1    0   1
Phalaenopsis-gene429502               0    0    0    1    0   1
Phalaenopsis-gene63385                1    1    1    1    1   5
Phalaenopsis-gene780766               1    0    1    1    0   3
Phalaenopsis-gene864479               1    0    0    0    0   1
Phalaenopsis-gene214070               1    1    0    1    0   3
Phalaenopsis-gene241284               1    1    1    1    1   5
Phalaenopsis-gene278590               1    0    0    1    0   2
Phalaenopsis-gene558279               0    1    1    0    0   2
Phalaenopsis-gene559397               1    0    1    1    0   3
Phalaenopsis-gene583359               1    0    1    0    1   3
Phalaenopsis-gene663247               1    1    0    1    1   4
Phalaenopsis-gene758016               1    1    1    1    1   5
Phalaenopsis-gene907557               1    1    1    1    1   5
Phalaenopsis-gene122339               1    0    0    1    0   2
Phalaenopsis-gene122377               0    0    0    1    0   1
Phalaenopsis-gene171957               1    1    1    1    1   5
Phalaenopsis-gene181371               1    1    1    1    1   5
Phalaenopsis-gene247271               1    0    1    1    0   3
Phalaenopsis-gene289794               1    1    1    1    1   5
Phalaenopsis-gene322394               1    0    0    1    0   2
Phalaenopsis-gene347885               1    1    1    1    1   5
Phalaenopsis-gene413498               0    0    1    0    0   1
Phalaenopsis-gene426326               1    0    0    1    0   2
Phalaenopsis-gene479603               1    0    1    0    0   2
Phalaenopsis-gene517954               1    0    1    1    0   3
Phalaenopsis-gene59387                1    0    1    1    0   3
Phalaenopsis-gene650390               1    1    1    1    1   5
Phalaenopsis-gene659060               1    0    1    1    0   3
Phalaenopsis-gene67728                1    1    1    1    1   5
Phalaenopsis-gene724106               1    1    1    1    1   5
Phalaenopsis-gene751345               0    0    1    1    0   2
Phalaenopsis-gene786206               1    1    1    1    1   5
Phalaenopsis-gene90082                1    0    1    1    0   3
Phalaenopsis-gene95603                1    0    0    0    0   1
Phalaenopsis-gene96883                1    1    1    1    1   5
Ophrys-gene839                        1    1    1    1    1   5
Ophrys-gene1167                       1    1    1    1    1   5
Ophrys-gene1573                       0    0    1    1    0   2
Ophrys-gene1840                       1    1    1    1    1   5
Ophrys-gene1984                       1    1    1    1    1   5
Ophrys-gene2828                       1    1    1    1    1   5
Ophrys-gene2863                       1    0    1    0    0   2
Ophrys-gene4708                       0    0    0    1    0   1
Ophrys-gene5283                       1    1    1    1    1   5
Ophrys-gene5531                       0    0    0    1    0   1
Ophrys-gene5626                       1    0    0    0    0   1
Ophrys-gene7407                       1    1    1    1    1   5
Ophrys-gene7711                       1    0    1    1    0   3
Ophrys-gene8310                       1    1    1    1    1   5
Ophrys-gene8587                       0    0    1    1    0   2
Ophrys-gene8925                       1    1    1    1    1   5
Ophrys-gene9780                       1    1    1    1    1   5
Ophrys-gene10552                      1    0    1    1    0   3
Ophrys-gene10724                      1    1    1    1    1   5
Ophrys-gene12282                      1    1    1    1    1   5
Ophrys-gene12319                      1    1    1    1    1   5
Ophrys-gene20222                      1    0    1    0    0   2
Ophrys-gene20941                      1    0    1    0    0   2
Ophrys-gene21384                      1    1    1    1    1   5
Ophrys-gene21619                      1    1    1    1    1   5
Ophrys-gene21645                      0    0    1    0    0   1
Ophrys-gene22112                      1    1    1    1    1   5
Ophrys-gene23224                      1    0    1    1    0   3
Ophrys-gene23798                      0    0    1    1    0   2
Ophrys-gene23837                      0    0    1    1    0   2
Ophrys-gene24970                      1    1    1    1    1   5
Ophrys-gene38185                      1    1    1    1    1   5
Ophrys-gene38727                      1    1    1    1    1   5
Ophrys-gene49965                      0    0    0    1    0   1
Ophrys-gene49940                      1    1    1    1    1   5
Ophrys-gene50796                      0    0    1    1    0   2
Ophrys-gene51276                      1    0    0    0    0   1
Ophrys-gene53504                      1    0    0    1    0   2
Ophrys-gene53867                      1    1    1    1    1   5
Ophrys-gene55262                      1    0    0    1    0   2
Ophrys-gene56602                      1    1    1    1    1   5
Ophrys-gene57972                      1    1    1    1    1   5
Ophrys-gene58862                      0    0    0    1    0   1
Ophrys-gene60395                      1    0    0    1    0   2
Ophrys-gene60416                      1    0    0    1    0   2
Ophrys-gene60745                      0    0    1    1    0   2
Ophrys-gene61776                      1    0    0    0    0   1
Ophrys-gene62117                      1    0    1    0    0   2
Ophrys-gene62350                      1    1    1    1    1   5
Ophrys-gene62967                      1    1    1    1    1   5
Ophrys-gene62975                      0    0    1    1    0   2
Ophrys-gene63438                      1    0    1    1    0   3
Ophrys-gene67373                      1    1    1    1    1   5
Ophrys-gene68457                      1    1    1    1    1   5
Ophrys-gene68466                      1    0    1    1    0   3
Ophrys-gene69875                      1    0    0    1    0   2
Ophrys-gene72071                      1    0    1    0    0   2
Ophrys-gene72087                      1    0    0    0    0   1
Ophrys-gene72063                      1    0    0    1    0   2
Ophrys-gene74227                      0    0    0    1    0   1
Ophrys-gene76601                      1    0    1    1    0   3
Ophrys-gene77023                      1    0    0    1    0   2
Ophrys-gene76843                      0    0    0    1    0   1
Ophrys-gene76881                      1    1    1    1    1   5
Ophrys-gene76821                      1    0    0    0    0   1
Ophrys-gene76903                      0    1    0    0    0   1
Ophrys-gene76826                      1    0    0    1    0   2
Ophrys-gene77251                      1    1    1    1    1   5
Ophrys-gene78242                      0    0    1    1    1   3
Ophrys-gene80982                      1    0    0    0    0   1
Ophrys-gene82080                      0    0    0    1    0   1
Ophrys-gene83763                      1    1    1    1    1   5
Ophrys-gene84019                      1    0    0    1    0   2
Ophrys-gene84203                      0    0    1    1    0   2
Ophrys-gene85601                      1    1    1    0    0   3
Ophrys-gene88151                      1    1    1    1    1   5
Ophrys-gene92090                      0    0    0    0    0   0
Ophrys-gene97047                      1    1    1    1    1   5
Ophrys-gene98992                      0    0    1    0    0   1
Ophrys-gene99904                      1    1    1    1    1   5
Ophrys-gene101209                     0    1    1    1    0   3
Ophrys-gene105580                     0    0    1    1    0   2
Ophrys-gene105581                     1    1    1    1    1   5
Ophrys-gene112200                     1    1    1    1    1   5
Ophrys-gene113712                     1    1    1    1    1   5
Ophrys-gene113693                     1    1    1    1    1   5
Ophrys-gene114875                     1    1    1    1    1   5
Ophrys-gene117398                     1    1    1    1    1   5
Ophrys-gene118632                     1    1    1    1    1   5
Ophrys-gene123138                     1    0    0    1    0   2
Ophrys-gene126683                     1    1    1    1    1   5
Ophrys-gene127053                     0    0    1    0    0   1
Ophrys-gene132865                     1    1    1    1    1   5
Ophrys-gene133094                     1    0    1    1    0   3
Ophrys-gene133879                     1    0    0    1    0   2
Ophrys-gene134800                     1    1    1    1    1   5
Ophrys-gene134818                     1    1    1    1    1   5
Ophrys-gene136052                     1    0    0    1    0   2
Ophrys-gene136770                     1    1    1    1    1   5
Ophrys-gene137143                     1    0    1    1    0   3
Ophrys-gene139707                     1    1    1    1    1   5
Ophrys-gene139739                     1    1    1    1    1   5
Ophrys-gene140670                     1    0    1    1    0   3
Ophrys-gene143560                     0    0    1    1    0   2
Ophrys-gene144841                     1    0    0    0    0   1
Ophrys-gene145127                     0    0    1    1    0   2
Ophrys-gene149031                     1    1    1    1    1   5
Ophrys-gene154542                     1    1    0    1    0   3
Ophrys-gene154584                     1    0    0    1    0   2
Ophrys-gene159145                     1    1    0    1    0   3
Ophrys-gene162942                     1    0    0    1    0   2
Ophrys-gene167696                     1    1    1    1    1   5
Ophrys-gene171670                     0    0    0    1    0   1
Ophrys-gene173236                     1    0    0    1    0   2
Ophrys-gene173618                     1    0    0    1    0   2
Ophrys-gene174186                     0    0    0    0    0   0
Ophrys-gene174187                     1    1    1    1    1   5
Ophrys-gene178479                     1    0    1    1    0   3
Ophrys-gene178623                     1    0    0    1    0   2
Ophrys-gene178657                     1    0    0    1    0   2
Ophrys-gene178781                     1    0    0    1    0   2
Ophrys-gene180055                     0    0    1    1    0   2
Ophrys-gene180223                     1    1    1    1    1   5
Ophrys-gene180380                     1    1    1    1    1   5
Ophrys-gene181546                     0    0    0    1    0   1
Ophrys-gene181736                     1    0    0    1    0   2
Ophrys-gene182818                     0    1    0    0    0   1
Ophrys-gene182819                     1    0    1    1    0   3
Ophrys-gene182855                     0    0    0    1    0   1
Ophrys-gene183705                     1    1    1    1    1   5
Ophrys-gene183708                     1    1    1    1    1   5
Ophrys-gene183734                     0    0    1    0    0   1
Ophrys-gene183740                     1    0    0    0    1   2
Ophrys-gene186812                     1    1    1    1    1   5
Ophrys-gene187126                     1    0    0    1    0   2
Ophrys-gene195024                     1    0    0    0    0   1
Ophrys-gene195078                     1    0    0    1    0   2
Ophrys-gene196120                     1    0    0    1    0   2
Ophrys-gene196236                     1    0    0    0    0   1
Ophrys-gene197269                     0    1    1    0    0   2
Ophrys-gene198677                     1    1    1    1    1   5
Ophrys-gene205853                     1    0    0    1    0   2
Ophrys-gene205894                     0    0    0    1    0   1
Ophrys-gene205934                     0    0    0    0    0   0
Ophrys-gene205974                     0    0    0    0    0   0
Ophrys-gene207560                     1    0    0    0    0   1
Ophrys-gene210362                     1    1    1    1    1   5
Ophrys-gene210811                     0    0    1    0    0   1
Ophrys-gene210789                     1    1    1    1    1   5
Ophrys-gene210853                     0    0    0    0    0   0
Ophrys-gene211134                     0    0    0    1    0   1
Ophrys-gene212070                     1    1    1    1    1   5
Ophrys-gene213017                     1    0    1    1    0   3
Ophrys-gene213023                     1    0    0    1    0   2
Ophrys-gene213085                     1    1    1    1    1   5
Ophrys-gene214612                     1    1    1    1    1   5
Ophrys-gene214624                     1    0    0    1    1   3
Ophrys-gene215379                     1    0    0    1    0   2
Ophrys-gene215609                     1    1    1    1    1   5
Ophrys-gene217613                     0    0    0    1    0   1
Ophrys-gene218727                     1    1    1    1    1   5
Ophrys-gene222219                     1    1    1    1    1   5
Ophrys-gene225718                     1    1    1    1    1   5
Ophrys-gene234723                     1    0    0    1    0   2
Ophrys-gene234785                     1    1    1    1    1   5
Ophrys-gene234906                     1    0    1    1    0   3
Ophrys-gene234907                     1    0    0    1    0   2
Ophrys-gene236065                     1    0    0    1    0   2
Ophrys-gene236626                     1    0    0    1    0   2
Ophrys-gene236650                     1    0    0    1    0   2
Ophrys-gene238553                     1    0    0    1    0   2
Ophrys-gene238929                     0    0    1    1    1   3
Ophrys-gene239209                     1    1    1    1    1   5
Ophrys-gene239518                     1    0    0    1    0   2
Ophrys-gene240119                     1    0    1    1    0   3
Ophrys-gene247329                     1    1    1    1    1   5
Ophrys-gene251047                     1    1    1    1    1   5
Ophrys-gene252096                     1    1    1    1    1   5
Ophrys-gene252087                     1    1    1    1    1   5
Ophrys-gene255010                     1    0    1    1    1   4
Ophrys-gene257616                     1    1    1    1    1   5
Ophrys-gene263867                     0    0    0    1    0   1
Ophrys-gene264197                     1    1    1    1    1   5
Ophrys-gene265619                     1    1    1    0    0   3
Ophrys-gene265607                     1    1    1    1    1   5
Ophrys-gene265645                     1    1    1    1    1   5
Ophrys-gene265750                     0    1    0    1    0   2
Ophrys-gene271079                     0    0    1    1    0   2
Ophrys-gene271083                     1    0    1    1    0   3
Ophrys-gene271844                     1    0    1    0    0   2
Ophrys-gene278477                     0    0    1    0    0   1
Ophrys-gene278512                     1    0    0    1    0   2
Ophrys-gene280679                     1    1    1    1    1   5
Ophrys-gene281580                     1    1    1    1    1   5
Ophrys-gene282215                     1    1    1    1    1   5
Ophrys-gene284686                     1    1    1    1    1   5
Ophrys-gene286811                     1    1    1    1    1   5
Ophrys-gene291706                     1    1    1    1    1   5
Ophrys-gene291893                     1    0    0    1    0   2
Ophrys-gene293062                     1    0    0    0    0   1
Ophrys-gene293144                     1    0    1    1    0   3
Ophrys-gene293145                     1    1    1    1    1   5
Ophrys-gene293199                     1    0    1    1    0   3
Ophrys-gene293200                     1    1    1    1    1   5
Ophrys-gene294674                     1    0    0    1    0   2
Ophrys-gene295270                     1    1    1    1    1   5
Ophrys-gene295391                     1    0    0    1    0   2
Ophrys-gene295408                     0    0    1    1    0   2
Ophrys-gene295534                     1    0    0    1    0   2
Ophrys-gene295786                     0    0    1    0    0   1
Ophrys-gene295981                     1    1    1    1    1   5
Ophrys-gene296680                     0    0    1    1    0   2
Ophrys-gene297041                     1    1    1    1    1   5
Ophrys-gene298733                     1    1    1    1    1   5
Ophrys-gene298875                     1    0    1    0    0   2
Ophrys-gene300502                     1    1    1    1    1   5
Ophrys-gene304054                     1    0    1    1    0   3
Ophrys-gene304046                     1    0    0    1    0   2
Ophrys-gene304388                     1    1    1    1    1   5
Ophrys-gene304565                     1    0    0    0    0   1
Ophrys-gene304847                     1    1    1    1    1   5
Ophrys-gene305109                     1    0    1    1    0   3
Ophrys-gene306697                     1    0    0    1    0   2
Ophrys-gene306833                     1    1    1    1    1   5
Ophrys-gene306899                     1    0    0    0    0   1
Ophrys-gene307204                     1    1    1    1    1   5
Ophrys-gene312979                     1    0    1    1    0   3
Ophrys-gene317257                     1    1    1    1    1   5
Ophrys-gene318876                     1    0    1    0    0   2
Ophrys-gene319015                     0    0    1    1    1   3
Ophrys-gene321694                     0    0    1    1    0   2
Ophrys-gene324705                     1    1    1    1    1   5
Ophrys-gene324653                     1    0    0    0    0   1
Ophrys-gene324786                     1    1    1    1    1   5
Ophrys-gene326516                     1    0    0    1    0   2
Ophrys-gene335715                     1    1    1    1    1   5
Ophrys-gene338352                     1    1    1    1    1   5
Ophrys-gene339111                     0    0    1    1    0   2
Ophrys-gene340184                     1    1    1    1    1   5
Ophrys-gene341512                     1    0    0    0    0   1
Ophrys-gene342483                     1    1    1    1    1   5
Ophrys-gene344510                     1    1    1    1    1   5
Ophrys-gene344517                     0    0    0    0    0   0
Ophrys-gene349566                     1    1    1    1    1   5
Ophrys-gene349574                     1    0    0    1    0   2
Ophrys-gene351522                     1    0    1    1    0   3
Ophrys-gene352579                     1    1    1    1    1   5
Ophrys-gene355304                     1    0    0    1    0   2
Ophrys-gene359475                     1    0    0    1    0   2
Ophrys-gene364504                     1    0    1    1    0   3
Ophrys-gene364505                     1    1    1    1    1   5
Ophrys-gene365184                     1    0    0    1    0   2
Ophrys-gene367981                     1    1    1    1    1   5
Ophrys-gene368596                     1    0    0    1    0   2
Ophrys-gene370138                     1    1    1    1    1   5
Ophrys-gene374336                     1    1    1    1    1   5
Ophrys-gene375010                     1    0    1    1    0   3
Ophrys-gene375114                     1    1    1    1    1   5
Ophrys-gene378104                     0    0    0    1    0   1
Ophrys-gene378147                     1    1    1    1    1   5
Ophrys-gene379517                     1    1    1    1    1   5
Ophrys-gene382149                     1    0    0    0    0   1
Ophrys-gene382150                     0    0    0    1    0   1
Ophrys-gene382230                     1    0    0    0    0   1
Ophrys-gene382303                     1    0    0    0    0   1
Ophrys-gene384598                     1    0    0    1    0   2
Ophrys-gene386151                     1    1    0    1    0   3
Ophrys-gene386397                     1    1    1    1    1   5
Ophrys-gene386418                     1    0    1    1    0   3
Ophrys-gene388303                     0    0    0    1    0   1
Ophrys-gene391035                     1    1    0    1    0   3
Ophrys-gene391762                     1    0    1    1    0   3
Ophrys-gene392592                     1    1    0    1    0   3
Ophrys-gene392883                     1    1    1    1    1   5
Ophrys-gene393488                     0    0    1    1    0   2
Ophrys-gene395175                     1    1    1    1    1   5
Ophrys-gene395633                     1    1    1    1    0   4
Ophrys-gene395634                     1    0    0    0    0   1
Ophrys-gene395755                     1    0    0    1    0   2
Ophrys-gene395784                     0    0    0    0    0   0
Ophrys-gene395811                     1    0    0    0    0   1
Ophrys-gene396936                     0    1    0    1    0   2
Ophrys-gene397499                     1    1    1    1    1   5
Ophrys-gene398681                     1    0    0    1    0   2
Ophrys-gene399311                     1    1    1    1    1   5
Ophrys-gene399435                     0    0    1    0    0   1
Ophrys-gene399941                     1    0    0    0    0   1
Ophrys-gene400018                     1    0    1    0    0   2
Ophrys-gene402531                     1    0    0    1    0   2
Ophrys-gene403140                     1    1    1    1    1   5
Ophrys-gene404764                     1    1    1    1    1   5
Ophrys-gene408974                     1    0    0    0    0   1
Ophrys-gene409831                     1    1    1    1    1   5
Ophrys-gene414675                     1    0    1    0    0   2
Ophrys-gene415543                     1    0    1    1    0   3
Ophrys-gene419141                     1    0    0    0    0   1
Ophrys-gene419623                     0    0    0    1    0   1
Ophrys-gene421462                     0    0    1    1    0   2
Ophrys-gene424046                     1    1    0    1    0   3
Ophrys-gene425635                     0    0    0    1    0   1
Ophrys-gene425694                     1    0    0    0    0   1
Ophrys-gene427282                     1    0    1    1    0   3
Ophrys-gene427284                     1    1    1    1    1   5
Ophrys-gene427285                     1    0    1    1    0   3
Ophrys-gene427706                     1    1    1    1    1   5
Ophrys-gene429372                     1    0    0    1    0   2
Ophrys-gene430667                     1    0    1    0    0   2
Ophrys-gene434975                     1    0    0    1    0   2
Ophrys-gene435310                     1    1    1    1    1   5
Ophrys-gene440797                     1    1    1    1    1   5
Ophrys-gene440781                     1    0    0    0    0   1
Ophrys-gene441025                     1    0    0    0    0   1
Ophrys-gene441461                     1    1    1    1    1   5
Ophrys-gene444786                     0    0    0    0    0   0
Ophrys-gene445143                     1    1    1    1    1   5
Ophrys-gene445193                     1    0    0    1    0   2
Ophrys-gene445335                     1    0    1    1    0   3
Ophrys-gene446083                     1    0    0    1    1   3
Ophrys-gene446298                     1    1    1    1    1   5
Ophrys-gene446307                     1    1    1    1    1   5
Ophrys-gene446632                     1    0    0    0    0   1
Ophrys-gene446672                     1    1    1    1    1   5
Ophrys-gene447261                     1    1    1    1    1   5
Ophrys-gene447262                     0    0    1    1    0   2
Ophrys-gene447370                     1    1    1    1    1   5
Ophrys-gene447553                     1    1    1    1    1   5
Ophrys-gene449424                     1    0    1    1    0   3
Ophrys-gene453191                     1    0    0    1    0   2
Ophrys-gene458229                     1    0    1    1    0   3
Ophrys-gene461903                     1    0    1    1    0   3
Ophrys-gene462548                     0    0    1    0    0   1
Ophrys-gene473590                     0    0    0    1    0   1
Ophrys-gene475061                     0    1    0    1    0   2
Ophrys-gene479120                     1    0    0    1    0   2
Ophrys-gene479130                     1    1    1    1    1   5
Ophrys-gene483193                     0    1    0    1    0   2
Ophrys-gene483267                     1    0    1    1    1   4
Ophrys-gene483268                     1    1    1    1    1   5
Ophrys-gene483715                     0    0    1    1    0   2
Ophrys-gene487971                     1    0    1    0    0   2
Ophrys-gene488928                     1    1    1    1    1   5
Ophrys-gene488932                     1    0    0    0    0   1
Ophrys-gene490161                     1    0    0    1    0   2
Ophrys-gene491445                     1    1    1    1    1   5
Ophrys-gene493071                     0    0    0    0    1   1
Ophrys-gene493804                     1    1    1    1    1   5
Ophrys-gene495434                     1    0    0    1    0   2
Ophrys-gene495557                     1    1    0    0    0   2
Ophrys-gene495719                     1    0    1    1    0   3
Ophrys-gene495732                     1    0    1    1    0   3
Ophrys-gene495828                     1    0    0    1    1   3
Ophrys-gene495841                     1    1    1    1    0   4
Ophrys-gene495934                     0    0    0    1    0   1
Ophrys-gene495993                     1    0    0    0    0   1
Ophrys-gene496138                     1    1    1    1    1   5
Ophrys-gene496386                     1    0    0    1    0   2
Ophrys-gene496392                     0    0    0    1    0   1
Ophrys-gene496546                     1    1    1    1    1   5
Ophrys-gene497662                     1    1    1    1    1   5
Ophrys-gene504473                     1    1    1    1    1   5
Ophrys-gene504669                     1    1    1    1    1   5
Ophrys-gene504670                     1    0    1    1    0   3
Ophrys-gene506607                     0    0    0    1    0   1
Ophrys-gene508458                     0    0    1    1    0   2
Ophrys-gene508452                     1    1    1    1    1   5
Ophrys-gene509051                     1    1    1    1    1   5
Ophrys-gene509228                     1    1    1    1    1   5
Ophrys-gene511880                     1    1    1    1    1   5
Ophrys-gene515408                     1    1    1    1    1   5
Ophrys-gene518274                     1    0    0    1    0   2
Ophrys-gene519091                     1    1    1    1    1   5
Ophrys-gene520841                     1    0    1    1    0   3
Ophrys-gene525781                     1    0    0    0    0   1
Ophrys-gene525997                     1    0    1    1    0   3
Ophrys-gene525998                     0    0    1    1    0   2
Ophrys-gene526303                     1    1    1    1    1   5
Ophrys-gene528743                     1    0    0    1    0   2
Ophrys-gene528736                     0    0    1    1    0   2
Ophrys-gene531865                     1    0    1    0    0   2
Ophrys-gene532871                     1    0    0    1    0   2
Ophrys-gene538839                     1    0    0    0    0   1
Ophrys-gene542099                     0    0    1    0    0   1
Ophrys-gene542818                     1    0    1    1    0   3
Ophrys-gene543940                     1    1    1    1    1   5
Ophrys-gene549816                     1    0    0    0    0   1
Ophrys-gene549847                     1    1    1    1    1   5
Ophrys-gene549848                     1    1    1    1    1   5
Ophrys-gene551602                     1    0    0    0    0   1
Ophrys-gene554243                     1    0    1    0    0   2
Ophrys-gene554833                     1    0    1    1    0   3
Ophrys-gene555136                     1    1    1    1    1   5
Ophrys-gene564560                     0    0    0    1    0   1
Ophrys-gene565183                     1    1    1    1    1   5
Ophrys-gene566434                     1    1    1    1    1   5
Ophrys-gene574045                     1    0    1    1    0   3
Ophrys-gene577090                     1    0    1    0    1   3
Ophrys-gene577851                     1    1    1    1    1   5
Ophrys-gene578285                     1    0    0    0    0   1
Ophrys-gene578293                     1    0    1    1    0   3
Ophrys-gene579279                     0    0    1    0    0   1
Ophrys-gene579504                     0    0    0    1    0   1
Ophrys-gene585651                     1    0    0    1    0   2
Ophrys-gene586880                     1    1    1    1    1   5
Ophrys-gene589804                     1    0    0    0    0   1
Ophrys-gene591320                     1    1    1    1    1   5
Ophrys-gene591323                     1    1    1    1    1   5
Ophrys-gene591658                     0    0    1    0    0   1
Ophrys-gene592676                     0    0    1    1    0   2
Ophrys-gene595292                     0    0    1    1    0   2
Ophrys-gene597861                     1    1    1    1    1   5
Ophrys-gene598189                     1    0    1    1    0   3
Ophrys-gene598824                     1    0    0    0    0   1
Ophrys-gene601269                     1    1    1    1    1   5
Ophrys-gene603332                     1    1    1    1    1   5
Ophrys-gene603370                     1    1    1    1    1   5
Ophrys-gene606551                     1    1    1    1    1   5
Ophrys-gene606640                     1    1    1    0    0   3
Ophrys-gene608039                     0    0    1    1    0   2
Ophrys-gene608075                     1    1    0    1    0   3
Ophrys-gene610362                     1    0    1    0    0   2
Ophrys-gene611991                     1    0    0    0    0   1
Ophrys-gene617206                     1    0    0    1    0   2
Ophrys-gene617210                     1    0    1    1    0   3
Ophrys-gene618379                     1    1    1    1    0   4
Ophrys-gene619611                     1    0    1    1    0   3
Ophrys-gene623433                     0    0    0    1    0   1
Ophrys-gene632826                     1    1    1    1    1   5
Ophrys-gene638846                     1    0    0    0    0   1
Ophrys-gene639902                     1    1    1    1    1   5
Ophrys-gene640775                     1    0    1    1    0   3
Ophrys-gene646714                     1    1    0    0    0   2
Ophrys-gene646909                     0    0    0    1    0   1
Ophrys-gene656916                     1    0    0    1    0   2
Ophrys-gene658630                     1    1    1    1    1   5
Ophrys-gene658622                     0    0    0    1    0   1
Ophrys-gene661575                     1    0    0    1    0   2
Ophrys-gene661643                     1    0    0    1    0   2
Ophrys-gene662237                     1    1    1    1    1   5
Ophrys-gene676888                     1    1    1    1    1   5
Ophrys-gene677163                     1    0    0    0    0   1
Ophrys-gene678652                     1    0    1    0    0   2
Ophrys-gene680607                     1    0    0    1    1   3
Ophrys-gene680608                     1    0    0    0    0   1
Ophrys-gene680822                     1    0    0    1    0   2
Ophrys-gene681255                     0    0    0    1    0   1
Ophrys-gene681263                     0    1    1    1    0   3
Ophrys-gene681270                     1    1    1    1    1   5
Ophrys-gene683102                     1    0    1    1    0   3
Ophrys-gene683893                     1    0    0    1    0   2
Ophrys-gene687412                     1    1    1    1    1   5
Ophrys-gene689686                     1    0    1    0    0   2
Ophrys-gene689886                     1    0    0    0    0   1
Ophrys-gene690205                     1    0    0    0    0   1
Ophrys-gene690452                     1    1    1    1    1   5
Ophrys-gene690458                     1    1    1    1    1   5
Ophrys-gene692028                     1    0    0    1    0   2
Ophrys-gene701003                     0    0    1    0    0   1
Ophrys-gene702651                     1    1    1    1    1   5
Ophrys-gene702660                     1    0    0    1    0   2
Ophrys-gene702662                     1    0    1    1    0   3
Ophrys-gene708010                     0    0    1    1    0   2
Ophrys-gene708053                     1    1    0    1    0   3
Ophrys-gene708092                     0    0    1    0    0   1
Ophrys-gene709236                     1    1    1    1    1   5
Ophrys-gene709313                     1    1    0    0    0   2
Ophrys-gene709666                     1    1    1    1    1   5
Ophrys-gene709653                     1    1    1    1    1   5
Ophrys-gene715379                     0    0    0    0    0   0
Ophrys-gene716404                     0    1    0    1    0   2
Ophrys-gene718524                     1    1    1    1    1   5
Ophrys-gene722676                     1    1    1    1    1   5
Ophrys-gene730671                     1    1    1    1    1   5
Ophrys-gene739685                     1    0    1    1    0   3
Ophrys-gene739884                     1    0    1    1    1   4
Ophrys-gene740740                     0    0    0    1    0   1
Ophrys-gene757568                     1    1    1    1    1   5
Ophrys-gene757569                     1    0    0    1    0   2
Ophrys-gene758075                     1    0    0    0    0   1
Ophrys-gene758218                     1    1    0    1    0   3
Ophrys-gene758227                     1    0    0    1    0   2
Ophrys-gene758228                     1    0    0    1    0   2
Ophrys-gene758424                     0    0    0    0    0   0
Ophrys-gene762996                     1    0    1    0    0   2
Ophrys-gene769422                     1    1    1    1    1   5
Phalaenopsis-gene100347               1    0    0    1    0   2
Phalaenopsis-gene107351               1    1    1    1    1   5
Phalaenopsis-gene110586               1    0    1    1    0   3
Phalaenopsis-gene11245                1    0    0    0    0   1
Phalaenopsis-gene113076               1    0    0    1    0   2
Phalaenopsis-gene113123               1    0    0    1    0   2
Phalaenopsis-gene11955                0    0    1    0    0   1
Phalaenopsis-gene121253               1    1    1    1    1   5
Phalaenopsis-gene1260                 1    0    1    1    0   3
Phalaenopsis-gene1268                 1    0    1    1    0   3
Phalaenopsis-gene128024               1    1    1    1    1   5
Phalaenopsis-gene128917               0    0    1    1    0   2
Phalaenopsis-gene132963               1    0    0    1    0   2
Phalaenopsis-gene133020               1    0    0    1    0   2
Phalaenopsis-gene133058               1    1    1    1    0   4
Phalaenopsis-gene135374               0    0    0    0    0   0
Phalaenopsis-gene141000               1    1    1    1    1   5
Phalaenopsis-gene141026               1    0    1    1    0   3
Phalaenopsis-gene141030               1    0    1    1    0   3
Phalaenopsis-gene143498               1    1    1    1    1   5
Phalaenopsis-gene143502               0    0    0    1    0   1
Phalaenopsis-gene143539               1    0    1    1    0   3
Phalaenopsis-gene145306               1    0    0    1    0   2
Phalaenopsis-gene146053               1    0    1    1    0   3
Phalaenopsis-gene146208               0    0    0    1    0   1
Phalaenopsis-gene149609               1    0    1    1    0   3
Phalaenopsis-gene149610               1    0    1    1    0   3
Phalaenopsis-gene149971               1    0    1    0    0   2
Phalaenopsis-gene150027               0    0    1    0    0   1
Phalaenopsis-gene150171               1    0    0    0    0   1
Phalaenopsis-gene150258               0    1    1    1    0   3
Phalaenopsis-gene150314               1    1    1    1    1   5
Phalaenopsis-gene150320               1    1    1    1    1   5
Phalaenopsis-gene150336               1    0    1    1    0   3
Phalaenopsis-gene150844               1    0    0    1    0   2
Phalaenopsis-gene151097               1    0    1    1    0   3
Phalaenopsis-gene156082               1    1    1    1    1   5
Phalaenopsis-gene156976               1    0    0    1    0   2
Phalaenopsis-gene159561               1    1    1    1    1   5
Phalaenopsis-gene163097               1    0    1    1    0   3
Phalaenopsis-gene170932               1    0    0    0    0   1
Phalaenopsis-gene172040               1    0    1    1    0   3
Phalaenopsis-gene172202               1    0    0    1    0   2
Phalaenopsis-gene173801               1    1    1    1    1   5
Phalaenopsis-gene174050               0    0    0    0    0   0
Phalaenopsis-gene174075               0    0    0    1    0   1
Phalaenopsis-gene184598               1    0    0    0    0   1
Phalaenopsis-gene188619               1    1    1    1    0   4
Phalaenopsis-gene188860               0    0    0    0    0   0
Phalaenopsis-gene188914               1    0    1    1    0   3
Phalaenopsis-gene189132               1    1    1    1    1   5
Phalaenopsis-gene189133               1    0    0    1    0   2
Phalaenopsis-gene197892               0    0    1    1    0   2
Phalaenopsis-gene198686               1    0    0    1    0   2
Phalaenopsis-gene199505               0    0    1    1    0   2
Phalaenopsis-gene201112               1    0    1    0    0   2
Phalaenopsis-gene204254               1    1    1    1    1   5
Phalaenopsis-gene204255               1    1    1    1    1   5
Phalaenopsis-gene206121               1    0    1    1    0   3
Phalaenopsis-gene209175               0    0    1    1    0   2
Phalaenopsis-gene214709               1    0    0    1    0   2
Phalaenopsis-gene216670               1    1    1    1    1   5
Phalaenopsis-gene216912               1    0    0    1    0   2
Phalaenopsis-gene217980               1    1    0    1    0   3
Phalaenopsis-gene222063               1    1    1    1    1   5
Phalaenopsis-gene222328               0    0    1    1    0   2
Phalaenopsis-gene225289               0    0    1    1    0   2
Phalaenopsis-gene233161               0    0    0    0    0   0
Phalaenopsis-gene233209               1    0    1    1    0   3
Phalaenopsis-gene233210               1    0    1    1    0   3
Phalaenopsis-gene233214               1    0    1    0    0   2
Phalaenopsis-gene242330               1    1    1    1    1   5
Phalaenopsis-gene244761               0    0    0    1    0   1
Phalaenopsis-gene24632                1    0    1    1    0   3
Phalaenopsis-gene249209               1    0    0    0    0   1
Phalaenopsis-gene250181               1    1    1    1    1   5
Phalaenopsis-gene250757               1    1    1    1    1   5
Phalaenopsis-gene252345               0    0    1    1    0   2
Phalaenopsis-gene252383               0    1    1    1    0   3
Phalaenopsis-gene252390               0    0    0    1    0   1
Phalaenopsis-gene25336                1    0    1    1    0   3
Phalaenopsis-gene253835               0    0    1    1    0   2
Phalaenopsis-gene25418                1    0    1    0    0   2
Phalaenopsis-gene254644               1    0    1    1    1   4
Phalaenopsis-gene254670               1    1    1    1    1   5
Phalaenopsis-gene254697               1    0    1    1    0   3
Phalaenopsis-gene255717               1    1    1    1    1   5
Phalaenopsis-gene255921               1    0    1    0    0   2
Phalaenopsis-gene256094               1    0    0    0    0   1
Phalaenopsis-gene256460               0    0    1    0    1   2
Phalaenopsis-gene25789                1    1    1    1    1   5
Phalaenopsis-gene259958               1    1    1    1    1   5
Phalaenopsis-gene274268               1    0    0    1    0   2
Phalaenopsis-gene276807               1    0    0    0    0   1
Phalaenopsis-gene276816               1    0    0    1    0   2
Phalaenopsis-gene276857               1    1    1    1    1   5
Phalaenopsis-gene276866               1    0    1    1    0   3
Phalaenopsis-gene278589               1    1    1    1    1   5
Phalaenopsis-gene279779               1    1    1    1    1   5
Phalaenopsis-gene279830               0    0    1    1    0   2
Phalaenopsis-gene280009               0    0    0    0    0   0
Phalaenopsis-gene281595               0    1    1    1    0   3
Phalaenopsis-gene281870               1    0    0    0    0   1
Phalaenopsis-gene285359               0    0    0    0    0   0
Phalaenopsis-gene285857               1    0    1    1    0   3
Phalaenopsis-gene287230               1    1    1    1    1   5
Phalaenopsis-gene287349               1    1    1    1    1   5
Phalaenopsis-gene287357               1    0    0    1    0   2
Phalaenopsis-gene289091               1    1    1    1    1   5
Phalaenopsis-gene291790               1    1    1    1    1   5
Phalaenopsis-gene293574               0    0    0    0    0   0
Phalaenopsis-gene298354               1    0    1    1    0   3
Phalaenopsis-gene298415               1    1    1    1    1   5
Phalaenopsis-gene307182               1    0    0    0    0   1
Phalaenopsis-gene307249               1    0    0    1    0   2
Phalaenopsis-gene307602               1    0    1    1    0   3
Phalaenopsis-gene308052               1    0    0    1    0   2
Phalaenopsis-gene309585               1    1    0    0    0   2
Phalaenopsis-gene309659               1    1    1    1    1   5
Phalaenopsis-gene309666               0    1    1    0    0   2
Phalaenopsis-gene317366               1    0    0    1    0   2
Phalaenopsis-gene319775               0    1    0    1    0   2
Phalaenopsis-gene320037               1    1    1    1    1   5
Phalaenopsis-gene320052               1    0    1    1    0   3
Phalaenopsis-gene320061               0    0    0    1    0   1
Phalaenopsis-gene320065               0    0    1    0    0   1
Phalaenopsis-gene320085               1    0    0    0    0   1
Phalaenopsis-gene320135               1    0    1    1    0   3
Phalaenopsis-gene320148               1    0    1    1    0   3
Phalaenopsis-gene325291               1    1    1    1    1   5
Phalaenopsis-gene325298               1    1    1    1    1   5
Phalaenopsis-gene331278               1    0    1    1    0   3
Phalaenopsis-gene333979               1    1    1    1    1   5
Phalaenopsis-gene334345               1    0    0    1    0   2
Phalaenopsis-gene334940               1    0    0    1    0   2
Phalaenopsis-gene337380               1    0    0    1    0   2
Phalaenopsis-gene337924               1    1    1    1    1   5
Phalaenopsis-gene338587               1    1    1    1    1   5
Phalaenopsis-gene340328               1    1    1    1    1   5
Phalaenopsis-gene348751               1    0    0    1    0   2
Phalaenopsis-gene34976                0    0    1    0    0   1
Phalaenopsis-gene350693               0    0    1    0    0   1
Phalaenopsis-gene350941               1    1    1    1    1   5
Phalaenopsis-gene351005               1    1    1    1    1   5
Phalaenopsis-gene351006               1    1    1    1    1   5
Phalaenopsis-gene351264               1    0    0    0    0   1
Phalaenopsis-gene351411               0    0    0    1    0   1
Phalaenopsis-gene351429               0    0    0    1    0   1
Phalaenopsis-gene351471               0    0    1    0    0   1
Phalaenopsis-gene355123               0    0    1    1    0   2
Phalaenopsis-gene355255               1    0    1    1    0   3
Phalaenopsis-gene361681               1    0    0    0    0   1
Phalaenopsis-gene362135               1    0    1    0    0   2
Phalaenopsis-gene363229               1    1    1    1    1   5
Phalaenopsis-gene36546                1    0    1    1    0   3
Phalaenopsis-gene36616                1    0    1    0    0   2
Phalaenopsis-gene366984               1    0    1    0    0   2
Phalaenopsis-gene36837                1    0    1    1    0   3
Phalaenopsis-gene370950               1    1    1    1    1   5
Phalaenopsis-gene370991               1    0    1    0    0   2
Phalaenopsis-gene371022               1    0    0    0    0   1
Phalaenopsis-gene371028               1    0    1    0    0   2
Phalaenopsis-gene371906               1    1    0    0    0   2
Phalaenopsis-gene375266               1    0    1    1    0   3
Phalaenopsis-gene375467               0    0    1    0    0   1
Phalaenopsis-gene375659               0    0    1    0    0   1
Phalaenopsis-gene377197               1    0    0    1    0   2
Phalaenopsis-gene379970               1    0    1    1    0   3
Phalaenopsis-gene383944               0    0    1    1    0   2
Phalaenopsis-gene384535               1    1    1    1    1   5
Phalaenopsis-gene387330               1    0    1    0    0   2
Phalaenopsis-gene388028               1    1    1    1    1   5
Phalaenopsis-gene388144               0    0    1    0    0   1
Phalaenopsis-gene388594               1    1    1    1    1   5
Phalaenopsis-gene390151               1    0    0    0    0   1
Phalaenopsis-gene394029               1    0    0    0    0   1
Phalaenopsis-gene394198               1    1    0    1    0   3
Phalaenopsis-gene403479               1    1    1    0    0   3
Phalaenopsis-gene409843               1    1    1    1    1   5
Phalaenopsis-gene409988               1    1    1    1    1   5
Phalaenopsis-gene412719               0    0    0    0    0   0
Phalaenopsis-gene421985               1    1    1    1    1   5
Phalaenopsis-gene421991               1    0    1    1    0   3
Phalaenopsis-gene422002               1    0    1    1    0   3
Phalaenopsis-gene423848               1    0    0    1    0   2
Phalaenopsis-gene424272               1    1    1    1    1   5
Phalaenopsis-gene429902               1    1    1    1    1   5
Phalaenopsis-gene431420               1    1    1    1    1   5
Phalaenopsis-gene431500               0    0    1    1    0   2
Phalaenopsis-gene432795               0    0    1    1    0   2
Phalaenopsis-gene432816               0    0    0    0    0   0
Phalaenopsis-gene432852               1    0    1    1    0   3
Phalaenopsis-gene432961               1    1    1    1    1   5
Phalaenopsis-gene432982               1    0    1    1    0   3
Phalaenopsis-gene436731               1    0    1    0    0   2
Phalaenopsis-gene43737                1    0    1    1    0   3
Phalaenopsis-gene437894               1    0    1    0    0   2
Phalaenopsis-gene44296                1    0    0    1    0   2
Phalaenopsis-gene442980               1    0    0    1    0   2
Phalaenopsis-gene443153               1    1    1    1    1   5
Phalaenopsis-gene443156               1    1    1    1    1   5
Phalaenopsis-gene444151               1    0    1    1    0   3
Phalaenopsis-gene446775               1    0    1    1    0   3
Phalaenopsis-gene446776               1    1    1    1    1   5
Phalaenopsis-gene447140               1    0    0    0    0   1
Phalaenopsis-gene447274               1    0    1    1    1   4
Phalaenopsis-gene455609               1    0    0    1    0   2
Phalaenopsis-gene460170               1    1    1    0    0   3
Phalaenopsis-gene460463               0    0    1    1    0   2
Phalaenopsis-gene467115               1    1    1    1    1   5
Phalaenopsis-gene467823               1    1    1    1    1   5
Phalaenopsis-gene468332               1    0    1    1    0   3
Phalaenopsis-gene470461               1    1    1    1    1   5
Phalaenopsis-gene471630               0    1    0    1    0   2
Phalaenopsis-gene471901               1    0    1    1    0   3
Phalaenopsis-gene475219               1    0    0    0    0   1
Phalaenopsis-gene477895               1    0    1    1    0   3
Phalaenopsis-gene478578               0    1    0    0    0   1
Phalaenopsis-gene480048               1    0    1    1    0   3
Phalaenopsis-gene481093               1    0    1    1    0   3
Phalaenopsis-gene482605               1    1    1    1    1   5
Phalaenopsis-gene485903               0    0    1    1    0   2
Phalaenopsis-gene486039               1    0    1    1    0   3
Phalaenopsis-gene486228               1    0    1    1    0   3
Phalaenopsis-gene486285               1    0    0    0    0   1
Phalaenopsis-gene487539               1    1    1    0    0   3
Phalaenopsis-gene487928               0    0    1    0    0   1
Phalaenopsis-gene487954               1    1    1    1    1   5
Phalaenopsis-gene487964               1    0    1    1    0   3
Phalaenopsis-gene495344               0    1    0    1    0   2
Phalaenopsis-gene495345               1    1    1    1    1   5
Phalaenopsis-gene499483               1    1    1    1    1   5
Phalaenopsis-gene499484               1    0    0    1    0   2
Phalaenopsis-gene503985               1    1    1    1    1   5
Phalaenopsis-gene504411               0    0    1    0    0   1
Phalaenopsis-gene505473               1    0    0    1    0   2
Phalaenopsis-gene507989               1    0    1    1    0   3
Phalaenopsis-gene513648               0    0    1    1    0   2
Phalaenopsis-gene513739               1    0    1    1    0   3
Phalaenopsis-gene515742               1    1    1    1    1   5
Phalaenopsis-gene515887               1    1    1    1    0   4
Phalaenopsis-gene516540               1    0    1    1    0   3
Phalaenopsis-gene518616               1    1    1    1    1   5
Phalaenopsis-gene520718               1    1    1    1    1   5
Phalaenopsis-gene520881               0    0    0    0    0   0
Phalaenopsis-gene520882               0    0    1    1    0   2
Phalaenopsis-gene523444               0    0    0    1    0   1
Phalaenopsis-gene523463               1    1    1    1    1   5
Phalaenopsis-gene526113               1    1    1    1    1   5
Phalaenopsis-gene528276               1    0    1    1    0   3
Phalaenopsis-gene532603               1    1    1    1    1   5
Phalaenopsis-gene538064               1    1    1    1    1   5
Phalaenopsis-gene54228                1    1    1    1    1   5
Phalaenopsis-gene543451               1    1    1    1    1   5
Phalaenopsis-gene543772               1    0    1    0    0   2
Phalaenopsis-gene545648               1    1    1    1    1   5
Phalaenopsis-gene545649               1    0    1    1    0   3
Phalaenopsis-gene545912               1    1    1    1    1   5
Phalaenopsis-gene545913               1    1    1    1    1   5
Phalaenopsis-gene547699               1    1    1    1    1   5
Phalaenopsis-gene551739               1    0    0    1    0   2
Phalaenopsis-gene556689               1    1    1    1    1   5
Phalaenopsis-gene557672               0    0    1    1    0   2
Phalaenopsis-gene558052               1    1    1    1    1   5
Phalaenopsis-gene559861               1    1    0    1    0   3
Phalaenopsis-gene562522               1    0    1    1    0   3
Phalaenopsis-gene56265                1    1    1    1    1   5
Phalaenopsis-gene563887               0    0    0    1    0   1
Phalaenopsis-gene563901               1    1    0    0    0   2
Phalaenopsis-gene565258               1    1    1    1    1   5
Phalaenopsis-gene567100               1    0    1    1    1   4
Phalaenopsis-gene568293               1    1    1    1    1   5
Phalaenopsis-gene570110               1    0    1    0    0   2
Phalaenopsis-gene571722               1    0    0    0    0   1
Phalaenopsis-gene571725               1    0    1    1    0   3
Phalaenopsis-gene57990                1    1    1    1    1   5
Phalaenopsis-gene585576               1    0    0    0    0   1
Phalaenopsis-gene588946               1    0    1    0    0   2
Phalaenopsis-gene590953               1    1    1    1    1   5
Phalaenopsis-gene592039               1    0    0    1    0   2
Phalaenopsis-gene593064               1    0    1    0    0   2
Phalaenopsis-gene60772                1    0    1    1    0   3
Phalaenopsis-gene610697               1    0    1    1    0   3
Phalaenopsis-gene61262                1    1    1    1    1   5
Phalaenopsis-gene61264                0    0    1    1    0   2
Phalaenopsis-gene614509               1    1    1    1    1   5
Phalaenopsis-gene615970               0    0    1    1    0   2
Phalaenopsis-gene616023               1    0    1    0    0   2
Phalaenopsis-gene616996               1    0    0    1    0   2
Phalaenopsis-gene623656               1    1    1    1    1   5
Phalaenopsis-gene624199               0    0    1    0    0   1
Phalaenopsis-gene628573               1    0    1    0    0   2
Phalaenopsis-gene628574               1    0    0    1    0   2
Phalaenopsis-gene630177               1    0    1    1    0   3
Phalaenopsis-gene630605               0    0    0    1    0   1
Phalaenopsis-gene630783               0    0    0    0    0   0
Phalaenopsis-gene641030               1    1    1    1    1   5
Phalaenopsis-gene641206               1    0    1    0    0   2
Phalaenopsis-gene644661               0    0    1    1    0   2
Phalaenopsis-gene644735               1    0    0    0    0   1
Phalaenopsis-gene645439               0    0    0    1    0   1
Phalaenopsis-gene65111                0    0    0    0    0   0
Phalaenopsis-gene651574               1    1    1    1    1   5
Phalaenopsis-gene653007               1    0    0    1    0   2
Phalaenopsis-gene658038               1    0    1    1    0   3
Phalaenopsis-gene658878               1    0    1    1    0   3
Phalaenopsis-gene659577               1    0    0    1    0   2
Phalaenopsis-gene662773               1    1    1    1    1   5
Phalaenopsis-gene662810               1    0    0    1    0   2
Phalaenopsis-gene662865               1    0    0    1    0   2
Phalaenopsis-gene662916               1    1    1    1    1   5
Phalaenopsis-gene66310                1    1    0    1    0   3
Phalaenopsis-gene66322                1    1    1    1    1   5
Phalaenopsis-gene664961               1    1    0    0    0   2
Phalaenopsis-gene664980               1    1    1    1    1   5
Phalaenopsis-gene668708               1    0    1    1    0   3
Phalaenopsis-gene676901               1    1    1    1    1   5
Phalaenopsis-gene676908               0    0    1    1    0   2
Phalaenopsis-gene676952               1    1    1    1    1   5
Phalaenopsis-gene679849               1    1    1    1    1   5
Phalaenopsis-gene680733               1    0    1    1    0   3
Phalaenopsis-gene681224               1    1    1    1    1   5
Phalaenopsis-gene682256               1    0    0    1    0   2
Phalaenopsis-gene682286               1    1    1    1    1   5
Phalaenopsis-gene68571                1    1    1    1    1   5
Phalaenopsis-gene685726               0    0    1    1    0   2
Phalaenopsis-gene687026               1    0    0    1    0   2
Phalaenopsis-gene689014               0    0    0    1    0   1
Phalaenopsis-gene692157               0    0    1    0    0   1
Phalaenopsis-gene692234               1    0    1    0    0   2
Phalaenopsis-gene692405               1    0    0    1    0   2
Phalaenopsis-gene692408               1    0    1    1    0   3
Phalaenopsis-gene692427               0    0    1    1    0   2
Phalaenopsis-gene694160               1    1    1    1    1   5
Phalaenopsis-gene697975               1    0    0    1    0   2
Phalaenopsis-gene698043               1    0    0    0    0   1
Phalaenopsis-gene698623               0    0    0    1    0   1
Phalaenopsis-gene706456               1    0    1    1    0   3
Phalaenopsis-gene706561               1    0    0    1    0   2
Phalaenopsis-gene707876               1    0    1    1    0   3
Phalaenopsis-gene715313               1    1    1    1    1   5
Phalaenopsis-gene716115               1    0    1    0    0   2
Phalaenopsis-gene716128               1    0    1    0    0   2
Phalaenopsis-gene716222               1    1    1    1    1   5
Phalaenopsis-gene716716               1    0    1    1    0   3
Phalaenopsis-gene721126               1    0    1    1    0   3
Phalaenopsis-gene725471               0    1    0    0    0   1
Phalaenopsis-gene731572               1    1    1    1    1   5
Phalaenopsis-gene731573               1    1    1    1    1   5
Phalaenopsis-gene734298               1    1    1    1    1   5
Phalaenopsis-gene744268               0    0    0    0    0   0
Phalaenopsis-gene744576               0    0    1    1    0   2
Phalaenopsis-gene745978               1    1    1    1    1   5
Phalaenopsis-gene746315               1    1    1    1    1   5
Phalaenopsis-gene747426               1    0    0    1    0   2
Phalaenopsis-gene750435               1    1    1    1    1   5
Phalaenopsis-gene751028               1    0    1    0    0   2
Phalaenopsis-gene755129               1    0    0    1    0   2
Phalaenopsis-gene758259               1    1    1    1    1   5
Phalaenopsis-gene766022               1    0    1    1    0   3
Phalaenopsis-gene766065               1    1    1    1    1   5
Phalaenopsis-gene767782               1    0    0    1    0   2
Phalaenopsis-gene767834               1    1    1    0    0   3
Phalaenopsis-gene772367               1    1    1    1    1   5
Phalaenopsis-gene774277               1    1    1    1    1   5
Phalaenopsis-gene774372               1    1    1    1    1   5
Phalaenopsis-gene774379               1    1    1    1    1   5
Phalaenopsis-gene774389               1    1    1    0    0   3
Phalaenopsis-gene780197               1    0    1    0    0   2
Phalaenopsis-gene784868               1    0    1    1    0   3
Phalaenopsis-gene786501               1    0    0    1    0   2
Phalaenopsis-gene789585               1    1    1    1    1   5
Phalaenopsis-gene792364               1    1    1    1    1   5
Phalaenopsis-gene793243               1    1    1    1    1   5
Phalaenopsis-gene794273               1    1    1    1    1   5
Phalaenopsis-gene799831               1    0    0    0    0   1
Phalaenopsis-gene806412               1    1    1    1    1   5
Phalaenopsis-gene819230               1    1    1    1    0   4
Phalaenopsis-gene819231               1    0    1    1    0   3
Phalaenopsis-gene819628               1    1    1    1    1   5
Phalaenopsis-gene820711               0    0    0    1    0   1
Phalaenopsis-gene823721               1    0    1    0    0   2
Phalaenopsis-gene823768               1    0    0    1    0   2
Phalaenopsis-gene831072               1    0    1    1    0   3
Phalaenopsis-gene8314                 1    1    1    1    0   4
Phalaenopsis-gene832743               1    1    1    0    0   3
Phalaenopsis-gene835963               1    1    1    1    1   5
Phalaenopsis-gene836680               1    0    1    0    0   2
Phalaenopsis-gene839686               1    1    1    1    1   5
Phalaenopsis-gene841130               0    1    0    1    0   2
Phalaenopsis-gene84233                1    0    0    1    0   2
Phalaenopsis-gene84245                0    0    1    1    0   2
Phalaenopsis-gene845368               1    0    1    0    0   2
Phalaenopsis-gene845769               1    0    0    1    0   2
Phalaenopsis-gene849415               1    0    1    0    0   2
Phalaenopsis-gene8532                 1    1    1    1    1   5
Phalaenopsis-gene853547               1    0    1    0    1   3
Phalaenopsis-gene856125               1    0    1    0    0   2
Phalaenopsis-gene857643               1    0    1    1    0   3
Phalaenopsis-gene857703               0    0    1    1    0   2
Phalaenopsis-gene857819               0    0    1    1    0   2
Phalaenopsis-gene8591                 1    1    1    1    1   5
Phalaenopsis-gene859293               1    1    1    0    0   3
Phalaenopsis-gene859407               0    1    0    1    0   2
Phalaenopsis-gene859410               1    0    0    1    0   2
Phalaenopsis-gene865364               1    1    1    1    1   5
Phalaenopsis-gene865587               0    0    1    1    0   2
Phalaenopsis-gene866796               0    0    1    1    0   2
Phalaenopsis-gene871013               1    0    0    0    0   1
Phalaenopsis-gene871591               0    0    1    1    0   2
Phalaenopsis-gene87441                1    1    1    1    1   5
Phalaenopsis-gene879632               1    1    1    1    1   5
Phalaenopsis-gene88243                1    1    1    1    1   5
Phalaenopsis-gene887145               0    0    1    0    0   1
Phalaenopsis-gene888277               1    0    1    1    0   3
Phalaenopsis-gene893464               1    0    1    1    0   3
Phalaenopsis-gene893519               1    0    1    1    0   3
Phalaenopsis-gene895171               0    0    1    1    0   2
Phalaenopsis-gene90333                1    0    1    1    0   3
Phalaenopsis-gene903715               0    0    0    1    0   1
Phalaenopsis-gene905225               0    1    0    0    0   1
Phalaenopsis-gene906838               1    1    1    1    1   5
Phalaenopsis-gene908071               1    0    1    1    0   3
Phalaenopsis-gene9347                 1    0    0    0    1   2
Phalaenopsis-gene93685                1    1    1    1    1   5
Phalaenopsis-gene9378                 1    0    1    1    0   3
Phalaenopsis-gene9507                 1    1    1    1    1   5
Phalaenopsis-gene95567                0    0    0    0    0   0
Phalaenopsis-gene95639                1    0    0    1    0   2
Phalaenopsis-gene96887                0    0    1    1    0   2
Phalaenopsis-gene98825                1    1    1    1    1   5
Ophrys-gene1570                       0    0    0    1    0   1
Ophrys-gene3776                       1    0    0    1    1   3
Ophrys-gene7661                       1    1    0    1    0   3
Ophrys-gene8066                       1    1    1    1    1   5
Ophrys-gene8335                       1    1    1    1    1   5
Ophrys-gene10219                      1    0    0    1    0   2
Ophrys-gene10908                      1    1    1    1    1   5
Ophrys-gene11766                      1    1    0    1    1   4
Ophrys-gene12137                      1    1    1    1    1   5
Ophrys-gene21038                      1    0    1    0    0   2
Ophrys-gene23540                      1    0    0    1    0   2
Ophrys-gene56843                      1    0    1    1    0   3
Ophrys-gene56867                      1    0    0    1    0   2
Ophrys-gene59581                      1    0    0    1    0   2
Ophrys-gene61757                      1    0    0    0    0   1
Ophrys-gene69969                      1    0    0    1    0   2
Ophrys-gene73044                      1    0    0    1    0   2
Ophrys-gene76990                      1    1    1    1    1   5
Ophrys-gene80567                      0    0    0    1    0   1
Ophrys-gene86265                      1    0    1    0    0   2
Ophrys-gene88971                      1    0    0    1    0   2
Ophrys-gene100057                     1    0    1    1    0   3
Ophrys-gene104648                     1    1    1    1    1   5
Ophrys-gene104679                     0    0    1    1    0   2
Ophrys-gene109051                     0    1    1    1    0   3
Ophrys-gene110807                     1    0    1    1    0   3
Ophrys-gene112449                     1    0    0    0    0   1
Ophrys-gene114879                     1    0    1    1    0   3
Ophrys-gene117151                     1    1    1    1    1   5
Ophrys-gene129275                     1    0    0    1    0   2
Ophrys-gene134897                     1    1    1    1    1   5
Ophrys-gene136036                     1    0    1    1    0   3
Ophrys-gene136774                     1    0    0    0    0   1
Ophrys-gene139693                     1    0    0    1    0   2
Ophrys-gene143315                     0    0    0    1    0   1
Ophrys-gene149147                     1    0    1    1    0   3
Ophrys-gene162941                     1    0    0    1    0   2
Ophrys-gene166518                     1    1    0    0    0   2
Ophrys-gene166634                     1    1    1    1    1   5
Ophrys-gene168525                     1    1    1    1    1   5
Ophrys-gene169601                     1    1    1    1    1   5
Ophrys-gene173581                     1    0    0    1    0   2
Ophrys-gene174189                     1    1    1    1    1   5
Ophrys-gene175062                     0    0    1    0    0   1
Ophrys-gene175407                     1    1    1    1    1   5
Ophrys-gene178472                     1    0    0    1    0   2
Ophrys-gene190460                     0    1    0    1    0   2
Ophrys-gene194031                     1    0    0    1    0   2
Ophrys-gene194046                     0    0    0    0    0   0
Ophrys-gene194968                     1    0    0    1    0   2
Ophrys-gene204385                     0    0    0    0    0   0
Ophrys-gene206087                     1    0    0    1    0   2
Ophrys-gene220441                     1    0    0    0    0   1
Ophrys-gene228900                     1    1    1    1    1   5
Ophrys-gene238839                     1    0    0    1    0   2
Ophrys-gene239091                     1    0    1    0    0   2
Ophrys-gene240134                     1    1    1    1    1   5
Ophrys-gene240161                     1    1    1    1    1   5
Ophrys-gene247241                     0    0    1    1    0   2
Ophrys-gene263166                     1    1    1    1    1   5
Ophrys-gene263287                     1    0    1    0    0   2
Ophrys-gene264789                     0    0    0    0    0   0
Ophrys-gene272178                     1    0    0    0    0   1
Ophrys-gene272982                     0    0    0    1    0   1
Ophrys-gene273006                     1    1    1    1    1   5
Ophrys-gene286888                     0    0    0    1    0   1
Ophrys-gene286953                     0    0    1    1    0   2
Ophrys-gene289803                     0    1    0    0    0   1
Ophrys-gene292690                     0    0    0    1    0   1
Ophrys-gene294454                     1    1    1    1    1   5
Ophrys-gene295527                     1    1    1    1    1   5
Ophrys-gene304866                     1    0    0    1    0   2
Ophrys-gene305108                     1    1    1    1    1   5
Ophrys-gene305997                     1    0    1    1    0   3
Ophrys-gene324694                     1    0    1    0    0   2
Ophrys-gene341049                     1    0    0    0    0   1
Ophrys-gene341067                     0    0    1    1    0   2
Ophrys-gene349745                     1    0    1    1    0   3
Ophrys-gene374927                     1    1    1    1    1   5
Ophrys-gene375060                     1    0    1    1    0   3
Ophrys-gene378941                     1    1    1    1    1   5
Ophrys-gene383943                     1    1    1    1    1   5
Ophrys-gene386401                     1    0    0    0    0   1
Ophrys-gene386394                     1    1    1    1    1   5
Ophrys-gene390988                     1    0    1    0    0   2
Ophrys-gene394556                     0    0    0    1    0   1
Ophrys-gene399437                     1    1    1    1    1   5
Ophrys-gene413926                     1    1    1    1    1   5
Ophrys-gene424995                     0    0    0    1    0   1
Ophrys-gene444779                     1    0    0    1    0   2
Ophrys-gene445094                     1    0    0    1    0   2
Ophrys-gene447379                     1    0    0    1    0   2
Ophrys-gene447554                     1    0    1    1    0   3
Ophrys-gene448063                     1    1    1    1    1   5
Ophrys-gene449144                     1    1    1    1    1   5
Ophrys-gene456710                     1    0    1    1    0   3
Ophrys-gene457585                     1    0    1    1    0   3
Ophrys-gene458187                     1    1    1    1    1   5
Ophrys-gene460230                     1    0    0    1    0   2
Ophrys-gene461697                     1    1    1    1    1   5
Ophrys-gene466288                     1    1    0    1    0   3
Ophrys-gene470181                     1    1    1    1    1   5
Ophrys-gene480113                     1    0    0    1    0   2
Ophrys-gene483200                     1    0    0    1    0   2
Ophrys-gene484152                     0    0    0    0    0   0
Ophrys-gene484571                     0    0    1    0    0   1
Ophrys-gene487337                     0    0    0    1    0   1
Ophrys-gene490075                     1    1    1    1    1   5
Ophrys-gene490169                     1    0    0    0    0   1
Ophrys-gene495985                     0    0    0    0    0   0
Ophrys-gene496552                     1    1    1    1    1   5
Ophrys-gene508291                     1    0    1    1    0   3
Ophrys-gene508476                     1    1    1    1    1   5
Ophrys-gene509368                     1    0    1    1    0   3
Ophrys-gene514738                     1    1    1    1    1   5
Ophrys-gene525695                     1    0    0    1    0   2
Ophrys-gene526018                     1    1    1    1    1   5
Ophrys-gene532176                     0    0    1    1    0   2
Ophrys-gene549812                     1    0    0    1    0   2
Ophrys-gene554467                     1    1    1    1    1   5
Ophrys-gene562776                     1    1    1    1    1   5
Ophrys-gene567002                     0    0    0    1    0   1
Ophrys-gene571415                     1    1    1    1    1   5
Ophrys-gene578279                     1    0    0    1    0   2
Ophrys-gene579592                     1    0    1    1    0   3
Ophrys-gene582198                     1    0    0    1    0   2
Ophrys-gene586715                     0    0    1    0    0   1
Ophrys-gene591397                     0    0    1    1    0   2
Ophrys-gene596333                     1    0    0    1    0   2
Ophrys-gene609782                     1    1    1    1    1   5
Ophrys-gene612007                     0    0    1    0    0   1
Ophrys-gene612323                     1    0    0    1    0   2
Ophrys-gene612511                     1    0    0    0    0   1
Ophrys-gene616647                     1    0    0    1    0   2
Ophrys-gene617126                     1    0    0    0    0   1
Ophrys-gene617390                     0    0    1    0    0   1
Ophrys-gene641683                     1    0    1    0    0   2
Ophrys-gene641685                     1    1    1    1    1   5
Ophrys-gene644134                     1    1    1    1    1   5
Ophrys-gene649415                     1    1    1    1    1   5
Ophrys-gene651224                     1    0    0    1    0   2
Ophrys-gene651225                     0    0    1    0    0   1
Ophrys-gene653616                     1    1    1    1    1   5
Ophrys-gene665441                     1    1    1    1    1   5
Ophrys-gene671376                     0    1    0    1    0   2
Ophrys-gene676444                     1    1    1    1    1   5
Ophrys-gene677097                     1    1    1    1    1   5
Ophrys-gene679572                     1    1    1    1    1   5
Ophrys-gene679714                     1    1    0    1    0   3
Ophrys-gene692644                     1    0    0    1    0   2
Ophrys-gene697317                     1    1    1    1    1   5
Ophrys-gene697712                     0    0    0    1    0   1
Ophrys-gene700927                     1    1    1    1    1   5
Ophrys-gene707988                     1    1    1    1    1   5
Ophrys-gene712253                     1    0    0    1    0   2
Ophrys-gene718048                     0    0    0    1    0   1
Ophrys-gene722774                     1    0    1    0    0   2
Ophrys-gene731572                     0    0    0    1    0   1
Ophrys-gene758425                     0    0    1    1    0   2
Ophrys-gene762871                     1    1    1    1    1   5
Ophrys-gene1040                       1    0    1    1    0   3
Ophrys-gene3120                       0    0    1    1    0   2
Ophrys-gene3454                       1    1    1    1    1   5
Ophrys-gene5631                       1    0    0    1    0   2
Ophrys-gene7426                       1    1    1    1    1   5
Ophrys-gene7726                       1    1    1    1    1   5
Ophrys-gene10124                      1    1    1    1    1   5
Ophrys-gene10935                      1    1    1    1    1   5
Ophrys-gene12320                      1    1    1    1    1   5
Ophrys-gene20067                      1    0    1    1    0   3
Ophrys-gene21138                      0    0    0    0    0   0
Ophrys-gene21558                      1    1    0    1    0   3
Ophrys-gene21646                      1    0    0    0    0   1
Ophrys-gene48532                      1    0    0    0    0   1
Ophrys-gene49061                      1    0    0    1    0   2
Ophrys-gene50236                      1    0    1    1    0   3
Ophrys-gene56601                      1    0    0    1    0   2
Ophrys-gene62531                      1    0    0    0    0   1
Ophrys-gene68424                      0    0    0    1    0   1
Ophrys-gene73025                      1    0    0    0    0   1
Ophrys-gene73955                      1    1    1    1    1   5
Ophrys-gene75179                      1    0    0    0    0   1
Ophrys-gene76284                      1    1    1    1    1   5
Ophrys-gene84011                      0    0    1    1    0   2
Ophrys-gene84015                      1    1    1    1    1   5
Ophrys-gene86103                      1    0    0    1    0   2
Ophrys-gene92093                      1    0    0    0    0   1
Ophrys-gene96592                      1    1    1    1    1   5
Ophrys-gene100018                     0    0    0    1    0   1
Ophrys-gene101546                     1    1    1    1    1   5
Ophrys-gene109413                     1    1    1    1    0   4
Ophrys-gene113674                     1    1    1    1    1   5
Ophrys-gene134820                     1    1    1    1    1   5
Ophrys-gene136912                     1    0    0    1    0   2
Ophrys-gene137163                     1    1    1    1    1   5
Ophrys-gene139727                     1    1    1    1    1   5
Ophrys-gene149090                     1    1    1    1    1   5
Ophrys-gene152670                     1    0    0    1    0   2
Ophrys-gene156322                     0    0    0    1    0   1
Ophrys-gene159590                     1    0    1    1    0   3
Ophrys-gene160465                     0    0    1    1    0   2
Ophrys-gene162743                     1    1    1    1    1   5
Ophrys-gene166452                     0    1    0    0    0   1
Ophrys-gene166776                     1    0    1    1    0   3
Ophrys-gene173238                     1    1    1    1    1   5
Ophrys-gene178063                     1    0    1    0    0   2
Ophrys-gene180217                     0    0    0    1    0   1
Ophrys-gene180615                     1    1    1    1    1   5
Ophrys-gene182726                     1    0    0    1    0   2
Ophrys-gene183870                     0    0    0    1    0   1
Ophrys-gene187509                     1    1    1    1    1   5
Ophrys-gene205692                     1    0    1    1    0   3
Ophrys-gene205852                     1    1    0    1    0   3
Ophrys-gene206051                     0    0    0    0    0   0
Ophrys-gene209737                     1    0    1    1    0   3
Ophrys-gene210755                     1    0    1    1    0   3
Ophrys-gene210806                     1    1    1    1    1   5
Ophrys-gene215359                     1    1    1    1    1   5
Ophrys-gene230468                     1    0    0    1    0   2
Ophrys-gene231084                     1    0    1    1    0   3
Ophrys-gene257617                     1    1    1    1    1   5
Ophrys-gene272335                     1    1    1    1    1   5
Ophrys-gene272914                     1    0    1    1    0   3
Ophrys-gene275730                     1    1    1    1    1   5
Ophrys-gene281584                     1    1    1    1    1   5
Ophrys-gene282663                     1    0    0    1    0   2
Ophrys-gene286217                     1    0    0    1    0   2
Ophrys-gene295213                     1    1    1    1    1   5
Ophrys-gene295372                     1    1    1    1    1   5
Ophrys-gene299810                     1    0    0    1    0   2
Ophrys-gene299812                     1    0    1    0    0   2
Ophrys-gene303769                     1    1    1    1    1   5
Ophrys-gene305027                     1    1    1    1    1   5
Ophrys-gene305983                     1    1    1    1    1   5
Ophrys-gene313031                     1    1    1    0    0   3
Ophrys-gene317258                     1    1    1    1    1   5
Ophrys-gene317297                     1    1    1    1    1   5
Ophrys-gene331585                     0    0    0    1    0   1
Ophrys-gene338179                     1    1    1    1    1   5
Ophrys-gene346421                     1    0    0    1    0   2
Ophrys-gene367989                     1    0    1    1    0   3
Ophrys-gene377613                     1    1    1    1    1   5
Ophrys-gene383213                     0    0    0    1    0   1
Ophrys-gene384220                     0    0    1    1    0   2
Ophrys-gene384530                     1    0    1    1    0   3
Ophrys-gene392649                     0    0    0    0    0   0
Ophrys-gene395839                     1    0    0    1    0   2
Ophrys-gene396517                     1    0    1    0    0   2
Ophrys-gene427620                     1    0    0    1    0   2
Ophrys-gene427624                     1    1    1    1    1   5
Ophrys-gene437437                     1    0    1    1    0   3
Ophrys-gene446335                     1    0    0    1    0   2
Ophrys-gene446723                     0    0    0    1    0   1
Ophrys-gene447283                     1    1    1    1    1   5
Ophrys-gene458076                     1    0    0    1    0   2
Ophrys-gene461873                     1    0    1    1    0   3
Ophrys-gene470192                     1    1    1    1    1   5
Ophrys-gene475748                     1    1    1    1    1   5
Ophrys-gene484496                     1    1    1    1    1   5
Ophrys-gene491505                     1    1    1    1    1   5
Ophrys-gene492450                     0    1    0    0    0   1
Ophrys-gene493805                     1    1    1    1    1   5
Ophrys-gene496477                     1    1    0    1    0   3
Ophrys-gene528207                     0    0    1    0    0   1
Ophrys-gene528195                     1    0    1    1    0   3
Ophrys-gene528735                     1    0    0    1    0   2
Ophrys-gene538306                     1    0    1    0    0   2
Ophrys-gene538767                     1    1    1    1    0   4
Ophrys-gene540469                     1    1    1    1    1   5
Ophrys-gene544567                     1    0    1    1    0   3
Ophrys-gene546685                     1    1    1    1    1   5
Ophrys-gene554470                     1    0    0    1    0   2
Ophrys-gene567390                     1    0    1    1    0   3
Ophrys-gene567484                     1    0    1    1    0   3
Ophrys-gene594984                     1    0    0    0    0   1
Ophrys-gene595775                     1    0    0    1    0   2
Ophrys-gene609112                     1    0    1    1    0   3
Ophrys-gene609324                     1    1    1    0    0   3
Ophrys-gene612527                     1    0    0    0    1   2
Ophrys-gene616639                     1    1    0    1    0   3
Ophrys-gene617362                     1    0    0    1    0   2
Ophrys-gene626188                     1    0    0    0    0   1
Ophrys-gene640199                     1    1    1    1    1   5
Ophrys-gene640721                     1    0    0    1    0   2
Ophrys-gene646078                     1    1    1    1    1   5
Ophrys-gene653673                     0    0    0    1    0   1
Ophrys-gene662244                     0    0    0    1    0   1
Ophrys-gene675304                     1    0    1    1    0   3
Ophrys-gene684592                     1    0    0    1    0   2
Ophrys-gene689918                     1    1    0    0    0   2
Ophrys-gene697388                     1    0    0    1    0   2
Ophrys-gene697617                     1    0    0    0    0   1
Ophrys-gene698647                     1    0    1    0    0   2
Ophrys-gene698633                     1    1    1    1    1   5
Ophrys-gene706398                     1    1    1    1    1   5
Ophrys-gene709677                     1    0    1    1    0   3
Ophrys-gene710702                     1    0    0    1    0   2
Ophrys-gene726146                     1    1    1    1    1   5
Ophrys-gene726151                     0    0    0    1    0   1
Ophrys-gene752686                     1    1    1    1    1   5
Ophrys-gene757388                     1    1    0    0    0   2
Ophrys-gene758415                     1    1    1    1    1   5
Ophrys-gene759886                     1    1    0    1    0   3
Ophrys-gene763280                     0    0    0    0    0   0
Ophrys-gene768793                     1    1    1    1    1   5
Ophrys-gene769419                     1    1    1    1    1   5
Ophrys-gene65318                      1    1    1    1    1   5
Ophrys-gene138185                     1    0    0    1    0   2
Ophrys-gene186811                     1    1    1    1    1   5
Ophrys-gene234766                     1    1    1    1    1   5
Ophrys-gene277372                     0    0    0    1    0   1
Ophrys-gene300497                     1    0    1    1    0   3
Ophrys-gene554811                     1    1    1    1    1   5
Ophrys-gene566377                     1    0    1    0    0   2
Ophrys-gene658618                     1    1    1    1    1   5
Ophrys-gene706383                     1    1    1    1    1   5
Ophrys-gene93868                      0    0    1    0    0   1
Ophrys-gene258310                     1    0    0    1    0   2
Ophrys-gene258712                     1    1    0    1    0   3
Ophrys-gene347935                     1    1    1    1    1   5
Ophrys-gene355119                     0    0    1    1    0   2
Ophrys-gene421140                     0    0    1    1    0   2
Ophrys-gene181162                     1    1    1    1    1   5
Ophrys-gene385136                     1    0    0    0    0   1
Ophrys-gene6799                       1    0    1    1    0   3
Ophrys-gene2832                       1    1    1    1    1   5
Ophrys-gene11099                      1    0    1    1    0   3
Ophrys-gene11971                      1    0    0    1    0   2
Ophrys-gene38098                      1    0    0    0    0   1
Ophrys-gene49354                      0    1    0    1    0   2
Ophrys-gene56093                      1    1    1    1    1   5
Ophrys-gene76925                      0    0    0    1    0   1
Ophrys-gene78252                      1    1    1    1    1   5
Ophrys-gene79455                      0    0    0    1    0   1
Ophrys-gene84014                      0    0    1    1    0   2
Ophrys-gene92988                      1    1    1    1    1   5
Ophrys-gene118890                     1    1    1    1    1   5
Ophrys-gene122508                     1    1    1    1    1   5
Ophrys-gene138858                     1    1    1    1    1   5
Ophrys-gene139676                     1    1    1    1    1   5
Ophrys-gene151294                     1    1    1    1    1   5
Ophrys-gene153216                     1    1    1    1    1   5
Ophrys-gene177041                     0    0    0    1    0   1
Ophrys-gene184189                     1    0    0    1    0   2
Ophrys-gene197373                     1    1    1    1    0   4
Ophrys-gene205973                     1    0    0    0    0   1
Ophrys-gene226337                     1    0    0    0    0   1
Ophrys-gene234908                     1    1    1    1    1   5
Ophrys-gene243833                     1    1    1    1    1   5
Ophrys-gene272955                     1    0    1    1    0   3
Ophrys-gene290385                     1    0    0    1    0   2
Ophrys-gene297201                     1    0    0    0    0   1
Ophrys-gene298987                     1    0    0    1    0   2
Ophrys-gene331574                     1    1    1    1    1   5
Ophrys-gene339116                     1    1    1    1    1   5
Ophrys-gene342459                     0    0    0    1    0   1
Ophrys-gene348256                     1    0    0    1    0   2
Ophrys-gene348900                     1    1    1    1    1   5
Ophrys-gene375219                     0    0    1    1    0   2
Ophrys-gene379304                     1    0    0    1    0   2
Ophrys-gene392593                     0    0    0    1    0   1
Ophrys-gene404129                     1    0    1    1    0   3
Ophrys-gene418455                     1    0    1    1    1   4
Ophrys-gene425266                     1    0    0    1    0   2
Ophrys-gene449890                     1    1    1    1    0   4
Ophrys-gene474420                     1    0    0    1    0   2
Ophrys-gene475290                     1    0    0    1    0   2
Ophrys-gene475747                     1    1    0    1    0   3
Ophrys-gene477586                     1    0    0    0    0   1
Ophrys-gene487078                     1    1    1    1    1   5
Ophrys-gene491262                     0    0    0    1    0   1
Ophrys-gene493550                     1    0    0    1    0   2
Ophrys-gene495725                     0    0    0    0    0   0
Ophrys-gene496148                     1    0    0    0    1   2
Ophrys-gene496287                     1    1    1    1    1   5
Ophrys-gene496587                     1    0    0    1    1   3
Ophrys-gene503605                     1    0    0    1    0   2
Ophrys-gene508471                     0    0    0    1    0   1
Ophrys-gene525804                     1    0    0    0    0   1
Ophrys-gene525811                     1    0    0    0    0   1
Ophrys-gene552148                     1    0    1    1    0   3
Ophrys-gene565179                     1    1    1    1    1   5
Ophrys-gene565166                     1    1    1    1    1   5
Ophrys-gene591243                     1    1    1    1    1   5
Ophrys-gene603355                     0    0    0    1    0   1
Ophrys-gene613695                     1    0    0    1    0   2
Ophrys-gene615020                     1    0    0    1    0   2
Ophrys-gene617231                     1    0    1    1    0   3
Ophrys-gene618380                     0    0    1    1    0   2
Ophrys-gene630251                     1    0    1    0    0   2
Ophrys-gene654379                     1    0    1    0    0   2
Ophrys-gene656893                     1    0    0    1    0   2
Ophrys-gene656988                     1    1    0    1    0   3
Ophrys-gene658629                     1    1    1    1    1   5
Ophrys-gene680089                     0    0    1    1    0   2
Ophrys-gene692735                     1    0    0    0    0   1
Ophrys-gene696115                     1    1    1    1    1   5
Ophrys-gene697216                     1    0    0    1    0   2
Ophrys-gene697273                     1    1    0    1    0   3
Ophrys-gene709285                     0    0    0    0    0   0
Ophrys-gene709676                     0    0    0    0    0   0
Ophrys-gene720747                     0    0    1    0    0   1
Ophrys-gene739688                     1    1    1    1    1   5
Ophrys-gene20889                      0    0    1    1    0   2
Ophrys-gene134141                     1    1    1    1    1   5
Ophrys-gene231498                     1    0    0    1    0   2
Ophrys-gene231547                     0    0    0    1    0   1
Ophrys-gene275191                     1    0    0    1    0   2
Ophrys-gene365583                     1    1    1    1    1   5
Ophrys-gene421033                     1    0    1    1    0   3
Ophrys-gene421157                     0    1    1    0    0   2
Ophrys-gene458124                     1    1    1    1    1   5
Ophrys-gene612407                     1    1    1    1    1   5
Ophrys-gene739679                     1    0    1    1    0   3
Ophrys-gene7174                       0    0    0    0    0   0
Ophrys-gene7833                       1    1    1    1    1   5
Ophrys-gene12203                      1    0    0    1    0   2
Ophrys-gene69707                      1    0    0    0    0   1
Ophrys-gene84583                      1    0    0    0    0   1
Ophrys-gene109013                     1    1    1    1    0   4
Ophrys-gene116202                     1    1    1    1    1   5
Ophrys-gene122509                     0    0    0    1    0   1
Ophrys-gene126764                     0    0    0    0    0   0
Ophrys-gene153775                     1    1    1    1    1   5
Ophrys-gene156601                     1    1    1    1    0   4
Ophrys-gene164412                     1    1    1    1    1   5
Ophrys-gene180612                     1    0    1    1    0   3
Ophrys-gene183733                     1    0    0    1    0   2
Ophrys-gene190199                     0    0    0    0    0   0
Ophrys-gene190200                     1    1    1    1    1   5
Ophrys-gene209349                     1    1    1    1    1   5
Ophrys-gene217599                     1    0    0    1    0   2
Ophrys-gene219405                     1    1    1    1    1   5
Ophrys-gene254744                     1    0    0    1    0   2
Ophrys-gene263480                     1    0    0    1    0   2
Ophrys-gene271558                     1    0    0    1    0   2
Ophrys-gene282208                     0    0    0    1    0   1
Ophrys-gene352784                     0    0    0    1    0   1
Ophrys-gene375113                     1    0    0    1    0   2
Ophrys-gene384541                     1    1    1    1    1   5
Ophrys-gene394511                     1    0    1    1    0   3
Ophrys-gene423269                     0    0    1    1    0   2
Ophrys-gene429053                     1    1    1    1    1   5
Ophrys-gene431810                     1    0    0    0    0   1
Ophrys-gene461016                     1    0    1    1    0   3
Ophrys-gene462543                     1    0    1    1    0   3
Ophrys-gene491550                     1    1    1    1    1   5
Ophrys-gene495502                     1    0    0    1    1   3
Ophrys-gene496101                     1    0    1    1    0   3
Ophrys-gene510604                     1    1    1    1    1   5
Ophrys-gene512377                     1    1    1    1    1   5
Ophrys-gene518100                     1    0    0    1    0   2
Ophrys-gene539277                     1    1    1    1    1   5
Ophrys-gene566213                     0    0    1    0    0   1
Ophrys-gene566958                     1    1    1    1    1   5
Ophrys-gene567615                     1    1    1    1    1   5
Ophrys-gene587281                     1    1    1    1    1   5
Ophrys-gene616686                     0    0    1    1    0   2
Ophrys-gene617234                     1    0    0    1    0   2
Ophrys-gene640722                     1    1    1    1    1   5
Ophrys-gene656624                     0    1    0    1    0   2
Ophrys-gene693198                     1    1    1    1    1   5
Ophrys-gene696111                     1    1    1    1    1   5
Ophrys-gene696757                     1    1    1    1    0   4
Ophrys-gene697202                     1    1    0    1    0   3
Ophrys-gene697274                     1    0    0    1    0   2
Ophrys-gene697501                     1    0    0    1    0   2
Ophrys-gene722357                     1    1    1    1    1   5
Ophrys-gene731462                     1    1    1    1    1   5
Ophrys-gene8971                       1    0    0    1    0   2
Ophrys-gene10557                      1    1    0    1    0   3
Ophrys-gene102230                     1    0    1    0    0   2
Ophrys-gene173265                     1    0    0    1    0   2
Ophrys-gene183187                     1    1    1    1    1   5
Ophrys-gene186405                     0    0    1    1    0   2
Ophrys-gene271985                     1    1    1    1    1   5
Ophrys-gene383353                     0    1    0    0    1   2
Ophrys-gene480805                     1    0    1    1    0   3
Ophrys-gene534667                     1    0    0    1    0   2
Ophrys-gene92                         1    0    0    1    0   2
Ophrys-gene5680                       1    1    0    0    0   2
Ophrys-gene8924                       1    1    1    1    1   5
Ophrys-gene24484                      0    0    0    0    0   0
Ophrys-gene104613                     1    1    0    1    0   3
Ophrys-gene116300                     0    0    1    0    0   1
Ophrys-gene136263                     1    1    1    1    1   5
Ophrys-gene138019                     1    1    1    1    1   5
Ophrys-gene155479                     1    0    1    0    0   2
Ophrys-gene258434                     1    1    1    1    1   5
Ophrys-gene306900                     1    0    0    0    0   1
Ophrys-gene536115                     1    1    1    1    1   5
Ophrys-gene582230                     1    1    1    1    1   5
Ophrys-gene3361                       1    0    1    1    0   3
Ophrys-gene5633                       0    0    1    1    0   2
Ophrys-gene12149                      1    0    0    1    0   2
Ophrys-gene22349                      1    1    1    1    1   5
Ophrys-gene38184                      1    1    1    1    1   5
Ophrys-gene48535                      1    0    0    1    0   2
Ophrys-gene73022                      1    0    0    0    0   1
Ophrys-gene73858                      0    1    0    1    0   2
Ophrys-gene76258                      1    0    0    1    0   2
Ophrys-gene102169                     1    0    0    0    0   1
Ophrys-gene126199                     1    1    1    1    1   5
Ophrys-gene127054                     1    1    1    1    1   5
Ophrys-gene136029                     1    1    1    1    1   5
Ophrys-gene141672                     1    0    0    1    0   2
Ophrys-gene143557                     0    0    1    1    0   2
Ophrys-gene159132                     1    1    1    1    1   5
Ophrys-gene159471                     0    0    0    0    0   0
Ophrys-gene160824                     0    0    1    1    0   2
Ophrys-gene171672                     1    1    1    0    0   3
Ophrys-gene177059                     1    1    1    1    1   5
Ophrys-gene182678                     0    1    0    1    0   2
Ophrys-gene183990                     0    0    0    0    0   0
Ophrys-gene189958                     0    0    0    0    0   0
Ophrys-gene190154                     1    0    1    1    0   3
Ophrys-gene214122                     1    0    0    1    0   2
Ophrys-gene258682                     0    0    0    1    0   1
Ophrys-gene263863                     1    0    1    1    0   3
Ophrys-gene264095                     1    0    1    0    0   2
Ophrys-gene272050                     1    0    0    1    0   2
Ophrys-gene282618                     1    1    1    1    1   5
Ophrys-gene295198                     1    1    1    1    1   5
Ophrys-gene303735                     0    0    0    1    0   1
Ophrys-gene303751                     0    0    1    1    0   2
Ophrys-gene305029                     1    0    1    0    0   2
Ophrys-gene338341                     1    0    1    1    0   3
Ophrys-gene353787                     1    1    1    1    1   5
Ophrys-gene390942                     0    0    0    1    0   1
Ophrys-gene392618                     1    0    1    1    0   3
Ophrys-gene393483                     1    0    0    1    0   2
Ophrys-gene425180                     1    0    0    1    0   2
Ophrys-gene427698                     0    0    0    1    0   1
Ophrys-gene439162                     0    0    0    1    1   2
Ophrys-gene480121                     1    0    1    1    0   3
Ophrys-gene490071                     1    0    0    0    0   1
Ophrys-gene492792                     1    0    0    1    0   2
Ophrys-gene493557                     1    0    1    0    0   2
Ophrys-gene493891                     1    1    1    1    1   5
Ophrys-gene496280                     1    0    0    1    0   2
Ophrys-gene518105                     1    0    0    0    0   1
Ophrys-gene528187                     1    0    1    0    0   2
Ophrys-gene567530                     1    0    1    1    0   3
Ophrys-gene567656                     1    0    1    1    0   3
Ophrys-gene591163                     0    0    0    0    0   0
Ophrys-gene597589                     1    0    0    1    0   2
Ophrys-gene608276                     1    1    1    1    1   5
Ophrys-gene612106                     1    1    1    1    1   5
Ophrys-gene617148                     1    1    0    0    0   2
Ophrys-gene623952                     1    0    0    0    0   1
Ophrys-gene640536                     1    0    0    1    1   3
Ophrys-gene645074                     1    0    1    1    0   3
Ophrys-gene649416                     0    1    0    1    0   2
Ophrys-gene656860                     1    0    1    1    0   3
Ophrys-gene708646                     0    0    0    1    0   1
Ophrys-gene709249                     1    1    1    1    1   5
Phalaenopsis-gene107620               1    0    1    1    0   3
Phalaenopsis-gene113131               1    0    1    0    0   2
Phalaenopsis-gene119451               1    1    1    1    1   5
Phalaenopsis-gene120547               1    0    1    1    0   3
Phalaenopsis-gene122357               1    0    0    1    0   2
Phalaenopsis-gene133056               1    0    0    1    0   2
Phalaenopsis-gene138868               1    1    1    1    1   5
Phalaenopsis-gene145893               1    0    0    1    0   2
Phalaenopsis-gene150053               1    1    1    1    1   5
Phalaenopsis-gene150172               1    1    0    1    0   3
Phalaenopsis-gene152903               1    0    0    0    0   1
Phalaenopsis-gene160792               1    0    0    1    0   2
Phalaenopsis-gene163431               1    0    1    1    0   3
Phalaenopsis-gene163734               1    1    1    1    1   5
Phalaenopsis-gene192007               1    1    1    1    1   5
Phalaenopsis-gene199495               1    1    1    1    1   5
Phalaenopsis-gene206821               1    0    0    1    1   3
Phalaenopsis-gene214698               1    1    1    1    1   5
Phalaenopsis-gene216888               0    0    0    1    0   1
Phalaenopsis-gene222235               1    0    1    1    0   3
Phalaenopsis-gene23025                1    1    1    1    1   5
Phalaenopsis-gene234963               0    0    0    1    0   1
Phalaenopsis-gene23700                1    0    1    1    0   3
Phalaenopsis-gene244682               1    0    0    1    0   2
Phalaenopsis-gene251208               1    0    1    0    1   3
Phalaenopsis-gene251238               0    0    1    1    0   2
Phalaenopsis-gene252368               1    0    1    1    0   3
Phalaenopsis-gene255254               1    1    1    1    0   4
Phalaenopsis-gene256028               0    0    1    0    0   1
Phalaenopsis-gene260                  1    1    1    1    1   5
Phalaenopsis-gene274106               1    0    1    0    0   2
Phalaenopsis-gene281355               0    0    1    1    0   2
Phalaenopsis-gene286646               1    0    1    1    0   3
Phalaenopsis-gene287422               1    0    0    1    0   2
Phalaenopsis-gene293698               1    0    0    1    0   2
Phalaenopsis-gene297283               1    0    1    0    0   2
Phalaenopsis-gene302948               1    1    1    1    1   5
Phalaenopsis-gene309466               1    1    1    1    0   4
Phalaenopsis-gene309638               1    0    1    0    0   2
Phalaenopsis-gene309931               0    0    1    1    0   2
Phalaenopsis-gene310215               1    1    0    1    0   3
Phalaenopsis-gene321160               0    0    1    1    0   2
Phalaenopsis-gene324623               1    0    0    1    0   2
Phalaenopsis-gene326394               1    0    1    1    0   3
Phalaenopsis-gene340330               1    1    1    1    1   5
Phalaenopsis-gene342345               1    0    1    1    0   3
Phalaenopsis-gene342893               1    0    0    0    0   1
Phalaenopsis-gene345793               0    0    1    0    0   1
Phalaenopsis-gene348655               1    0    1    0    0   2
Phalaenopsis-gene34985                1    0    0    1    0   2
Phalaenopsis-gene351403               1    0    1    0    0   2
Phalaenopsis-gene351489               1    0    0    1    0   2
Phalaenopsis-gene354927               0    0    1    1    0   2
Phalaenopsis-gene355317               1    0    1    1    0   3
Phalaenopsis-gene361592               1    0    1    1    0   3
Phalaenopsis-gene364971               0    0    0    1    0   1
Phalaenopsis-gene36810                0    0    1    0    1   2
Phalaenopsis-gene370446               1    0    1    0    0   2
Phalaenopsis-gene376697               1    1    1    1    1   5
Phalaenopsis-gene379961               1    0    0    1    0   2
Phalaenopsis-gene399537               1    1    1    1    1   5
Phalaenopsis-gene400838               1    1    1    1    1   5
Phalaenopsis-gene413800               1    0    1    1    0   3
Phalaenopsis-gene421992               1    0    1    0    0   2
Phalaenopsis-gene425519               0    1    1    1    0   3
Phalaenopsis-gene425665               1    1    1    1    1   5
Phalaenopsis-gene428566               1    0    1    1    0   3
Phalaenopsis-gene432873               1    0    0    1    0   2
Phalaenopsis-gene434199               0    0    0    0    0   0
Phalaenopsis-gene451676               0    0    0    1    0   1
Phalaenopsis-gene455843               1    1    1    1    1   5
Phalaenopsis-gene456990               1    1    1    1    1   5
Phalaenopsis-gene462971               0    0    1    0    0   1
Phalaenopsis-gene465774               1    0    1    1    0   3
Phalaenopsis-gene468556               1    0    0    1    0   2
Phalaenopsis-gene470906               1    0    0    1    0   2
Phalaenopsis-gene471023               1    0    1    0    0   2
Phalaenopsis-gene485624               1    1    1    1    1   5
Phalaenopsis-gene486067               0    0    1    0    0   1
Phalaenopsis-gene499448               1    0    1    1    0   3
Phalaenopsis-gene499454               1    1    1    1    1   5
Phalaenopsis-gene508021               1    0    1    1    0   3
Phalaenopsis-gene512907               1    1    1    1    1   5
Phalaenopsis-gene5178                 1    1    1    1    1   5
Phalaenopsis-gene522336               1    0    1    1    0   3
Phalaenopsis-gene526668               1    1    1    1    1   5
Phalaenopsis-gene528183               1    0    0    1    0   2
Phalaenopsis-gene529217               0    1    1    1    0   3
Phalaenopsis-gene533117               1    0    0    1    0   2
Phalaenopsis-gene533486               1    0    1    0    0   2
Phalaenopsis-gene536706               1    1    1    1    1   5
Phalaenopsis-gene538046               1    1    1    1    0   4
Phalaenopsis-gene539393               1    0    0    1    0   2
Phalaenopsis-gene548249               1    1    1    1    1   5
Phalaenopsis-gene551024               1    1    1    1    1   5
Phalaenopsis-gene556140               1    0    1    0    0   2
Phalaenopsis-gene557682               0    0    1    1    0   2
Phalaenopsis-gene557749               1    0    1    1    0   3
Phalaenopsis-gene56081                1    0    1    0    0   2
Phalaenopsis-gene563239               1    1    0    1    1   4
Phalaenopsis-gene570084               0    0    1    1    0   2
Phalaenopsis-gene576202               0    0    0    1    0   1
Phalaenopsis-gene585487               0    0    1    1    0   2
Phalaenopsis-gene592750               1    0    1    1    0   3
Phalaenopsis-gene593021               1    0    1    1    0   3
Phalaenopsis-gene593319               1    1    1    1    1   5
Phalaenopsis-gene59337                0    0    1    0    0   1
Phalaenopsis-gene598855               1    0    1    1    0   3
Phalaenopsis-gene60640                0    1    0    1    0   2
Phalaenopsis-gene615744               1    1    0    0    0   2
Phalaenopsis-gene621587               1    1    1    1    1   5
Phalaenopsis-gene623607               0    0    1    0    0   1
Phalaenopsis-gene625193               1    0    1    1    0   3
Phalaenopsis-gene629264               1    0    0    1    0   2
Phalaenopsis-gene650378               1    1    1    1    1   5
Phalaenopsis-gene650430               1    1    1    1    1   5
Phalaenopsis-gene662783               1    0    0    0    0   1
Phalaenopsis-gene663336               1    0    1    0    0   2
Phalaenopsis-gene663396               1    0    1    0    0   2
Phalaenopsis-gene66378                0    0    1    1    0   2
Phalaenopsis-gene673423               1    0    0    0    0   1
Phalaenopsis-gene698116               1    0    1    1    0   3
Phalaenopsis-gene698592               0    0    1    1    0   2
Phalaenopsis-gene702465               1    1    1    1    1   5
Phalaenopsis-gene706525               1    0    0    0    0   1
Phalaenopsis-gene709682               1    0    1    1    0   3
Phalaenopsis-gene716509               1    0    0    1    0   2
Phalaenopsis-gene718796               1    0    0    1    0   2
Phalaenopsis-gene730525               1    1    1    1    1   5
Phalaenopsis-gene741115               0    0    0    0    0   0
Phalaenopsis-gene74813                1    0    1    0    0   2
Phalaenopsis-gene74844                1    0    1    0    0   2
Phalaenopsis-gene75255                1    1    1    1    1   5
Phalaenopsis-gene753807               1    1    1    1    1   5
Phalaenopsis-gene763926               0    0    0    1    0   1
Phalaenopsis-gene767194               1    0    0    1    0   2
Phalaenopsis-gene773177               1    0    0    1    0   2
Phalaenopsis-gene774235               1    1    1    1    0   4
Phalaenopsis-gene774318               1    0    1    1    0   3
Phalaenopsis-gene776198               1    0    1    0    0   2
Phalaenopsis-gene781297               1    0    1    1    0   3
Phalaenopsis-gene784050               1    0    0    1    0   2
Phalaenopsis-gene784187               1    0    1    1    0   3
Phalaenopsis-gene786506               0    0    0    1    0   1
Phalaenopsis-gene789573               1    1    1    1    1   5
Phalaenopsis-gene8002                 1    0    1    1    0   3
Phalaenopsis-gene839755               1    1    1    1    1   5
Phalaenopsis-gene847529               1    1    1    1    1   5
Phalaenopsis-gene859560               1    0    0    1    0   2
Phalaenopsis-gene868323               1    1    1    1    1   5
Phalaenopsis-gene876680               1    0    1    1    0   3
Phalaenopsis-gene893852               0    0    0    0    0   0
Phalaenopsis-gene89407                1    1    1    1    0   4
Phalaenopsis-gene898186               1    0    1    1    0   3
Phalaenopsis-gene90090                1    0    1    0    0   2
Phalaenopsis-gene905162               1    1    0    1    0   3
Phalaenopsis-gene906833               0    1    1    1    0   3
Phalaenopsis-gene96070                1    0    0    1    0   2
Phalaenopsis-gene11261                1    1    1    1    1   5
Phalaenopsis-gene125520               1    0    1    0    0   2
Phalaenopsis-gene130045               1    1    1    1    1   5
Phalaenopsis-gene133292               1    1    1    1    1   5
Phalaenopsis-gene143486               1    0    1    1    0   3
Phalaenopsis-gene146209               1    0    0    0    0   1
Phalaenopsis-gene146254               1    0    1    0    0   2
Phalaenopsis-gene150321               1    1    1    1    1   5
Phalaenopsis-gene157350               1    1    1    1    1   5
Phalaenopsis-gene159117               0    0    0    1    0   1
Phalaenopsis-gene159283               1    0    0    0    0   1
Phalaenopsis-gene162991               1    0    0    1    0   2
Phalaenopsis-gene164224               1    1    1    1    1   5
Phalaenopsis-gene165204               1    0    0    1    0   2
Phalaenopsis-gene165989               1    0    0    1    0   2
Phalaenopsis-gene172184               1    1    1    1    1   5
Phalaenopsis-gene172206               1    0    0    0    0   1
Phalaenopsis-gene174111               1    0    0    1    0   2
Phalaenopsis-gene18206                0    1    1    1    0   3
Phalaenopsis-gene184454               1    0    0    0    0   1
Phalaenopsis-gene184493               0    0    0    1    0   1
Phalaenopsis-gene189014               1    0    1    0    0   2
Phalaenopsis-gene189086               1    0    0    1    0   2
Phalaenopsis-gene192342               1    1    1    1    1   5
Phalaenopsis-gene202330               1    1    1    1    1   5
Phalaenopsis-gene205306               1    1    1    1    0   4
Phalaenopsis-gene216671               0    1    1    1    0   3
Phalaenopsis-gene224029               1    0    1    0    0   2
Phalaenopsis-gene22934                1    0    1    0    0   2
Phalaenopsis-gene255852               1    1    1    0    0   3
Phalaenopsis-gene25790                1    0    1    1    0   3
Phalaenopsis-gene267765               0    1    1    0    0   2
Phalaenopsis-gene274105               0    0    1    1    0   2
Phalaenopsis-gene281361               1    1    1    1    1   5
Phalaenopsis-gene291264               1    1    1    1    0   4
Phalaenopsis-gene297293               1    1    1    1    1   5
Phalaenopsis-gene302471               1    1    1    1    1   5
Phalaenopsis-gene307228               0    0    1    1    1   3
Phalaenopsis-gene307498               1    0    0    1    0   2
Phalaenopsis-gene312178               0    0    1    0    0   1
Phalaenopsis-gene314279               1    1    1    1    1   5
Phalaenopsis-gene32271                1    1    1    1    1   5
Phalaenopsis-gene324321               1    1    1    1    1   5
Phalaenopsis-gene324593               0    0    1    1    0   2
Phalaenopsis-gene338593               1    1    1    1    1   5
Phalaenopsis-gene345925               1    0    1    1    0   3
Phalaenopsis-gene34749                1    0    0    1    0   2
Phalaenopsis-gene348714               0    0    0    1    0   1
Phalaenopsis-gene34978                0    0    0    1    0   1
Phalaenopsis-gene365860               1    1    1    1    1   5
Phalaenopsis-gene370919               1    0    0    0    0   1
Phalaenopsis-gene372204               1    1    1    1    0   4
Phalaenopsis-gene38323                1    0    1    1    0   3
Phalaenopsis-gene393789               0    0    0    1    0   1
Phalaenopsis-gene39817                1    0    1    1    0   3
Phalaenopsis-gene409747               1    0    1    1    0   3
Phalaenopsis-gene432928               0    0    0    1    0   1
Phalaenopsis-gene442728               1    1    1    1    1   5
Phalaenopsis-gene446785               1    0    0    1    0   2
Phalaenopsis-gene447341               1    0    1    1    0   3
Phalaenopsis-gene462970               1    1    1    1    1   5
Phalaenopsis-gene471943               0    0    0    1    0   1
Phalaenopsis-gene478586               1    0    1    1    0   3
Phalaenopsis-gene478614               1    0    1    1    0   3
Phalaenopsis-gene479653               0    0    0    1    0   1
Phalaenopsis-gene485460               1    1    1    1    1   5
Phalaenopsis-gene487935               1    0    0    1    0   2
Phalaenopsis-gene494562               1    1    1    1    1   5
Phalaenopsis-gene504014               1    1    1    1    1   5
Phalaenopsis-gene509242               1    1    1    1    1   5
Phalaenopsis-gene523493               1    0    0    1    0   2
Phalaenopsis-gene536675               0    0    0    1    0   1
Phalaenopsis-gene547311               1    1    1    1    1   5
Phalaenopsis-gene551503               1    0    0    1    0   2
Phalaenopsis-gene553061               1    1    1    1    1   5
Phalaenopsis-gene556962               0    0    0    1    0   1
Phalaenopsis-gene559187               1    1    1    1    1   5
Phalaenopsis-gene562669               1    1    1    0    0   3
Phalaenopsis-gene564728               1    1    1    1    1   5
Phalaenopsis-gene580134               1    1    1    1    1   5
Phalaenopsis-gene593022               0    0    0    1    0   1
Phalaenopsis-gene593065               0    0    0    1    0   1
Phalaenopsis-gene593299               1    1    1    1    1   5
Phalaenopsis-gene611567               1    1    1    1    1   5
Phalaenopsis-gene616315               1    0    0    1    0   2
Phalaenopsis-gene630795               1    0    0    0    0   1
Phalaenopsis-gene637043               0    0    0    0    0   0
Phalaenopsis-gene645729               1    0    1    0    0   2
Phalaenopsis-gene647255               1    1    1    1    1   5
Phalaenopsis-gene647280               0    0    1    1    0   2
Phalaenopsis-gene662687               0    0    0    1    0   1
Phalaenopsis-gene663811               1    0    0    1    0   2
Phalaenopsis-gene677353               0    1    0    0    0   1
Phalaenopsis-gene687173               1    0    1    1    0   3
Phalaenopsis-gene690248               1    0    0    1    0   2
Phalaenopsis-gene706524               1    0    0    1    0   2
Phalaenopsis-gene706536               0    0    0    1    0   1
Phalaenopsis-gene709946               1    1    1    1    1   5
Phalaenopsis-gene714277               1    1    1    1    1   5
Phalaenopsis-gene715363               1    1    1    1    1   5
Phalaenopsis-gene718787               1    0    0    1    0   2
Phalaenopsis-gene720886               0    0    0    1    0   1
Phalaenopsis-gene724607               0    0    1    1    0   2
Phalaenopsis-gene725496               1    0    1    1    0   3
Phalaenopsis-gene729568               0    0    0    1    0   1
Phalaenopsis-gene744160               1    0    1    0    0   2
Phalaenopsis-gene744572               0    0    0    1    0   1
Phalaenopsis-gene74784                1    0    0    1    0   2
Phalaenopsis-gene74875                0    0    1    0    0   1
Phalaenopsis-gene757731               1    1    1    1    1   5
Phalaenopsis-gene764115               1    1    1    1    1   5
Phalaenopsis-gene764196               1    1    1    1    1   5
Phalaenopsis-gene766230               0    0    0    0    0   0
Phalaenopsis-gene766231               1    0    1    1    0   3
Phalaenopsis-gene767867               0    0    0    1    0   1
Phalaenopsis-gene78472                1    0    0    1    0   2
Phalaenopsis-gene784885               0    1    1    1    0   3
Phalaenopsis-gene789590               0    0    0    1    0   1
Phalaenopsis-gene7932                 1    0    0    0    0   1
Phalaenopsis-gene793332               1    1    1    1    1   5
Phalaenopsis-gene7943                 0    0    0    1    0   1
Phalaenopsis-gene807153               1    0    1    1    0   3
Phalaenopsis-gene819182               1    1    1    1    0   4
Phalaenopsis-gene823707               1    0    1    1    0   3
Phalaenopsis-gene823782               1    1    0    1    0   3
Phalaenopsis-gene838872               1    0    0    0    0   1
Phalaenopsis-gene83915                1    0    0    1    0   2
Phalaenopsis-gene84390                1    0    1    1    0   3
Phalaenopsis-gene84396                1    0    0    0    0   1
Phalaenopsis-gene84818                1    1    1    1    1   5
Phalaenopsis-gene84831                1    1    1    1    1   5
Phalaenopsis-gene85177                1    0    0    1    0   2
Phalaenopsis-gene876724               1    0    1    1    0   3
Phalaenopsis-gene878082               1    0    0    1    1   3
Phalaenopsis-gene882575               1    1    1    1    1   5
Phalaenopsis-gene89275                1    0    0    1    0   2
Phalaenopsis-gene892995               1    0    0    0    0   1
Phalaenopsis-gene898908               1    1    1    1    1   5
Phalaenopsis-gene92941                1    0    0    1    0   2
Phalaenopsis-gene95602                0    0    0    0    0   0
Phalaenopsis-gene95640                1    0    0    1    0   2
Phalaenopsis-gene97140                1    1    1    1    1   5
Phalaenopsis-gene239614               1    1    1    1    1   5
Phalaenopsis-gene356200               1    0    1    1    0   3
Phalaenopsis-gene439961               1    1    1    1    1   5
Phalaenopsis-gene455709               1    1    1    1    1   5
Phalaenopsis-gene504420               0    0    0    1    0   1
Phalaenopsis-gene513913               1    1    1    1    1   5
Phalaenopsis-gene557741               1    0    0    0    0   1
Phalaenopsis-gene594181               0    0    1    0    0   1
Phalaenopsis-gene679221               1    1    1    1    1   5
Phalaenopsis-gene767764               1    0    1    1    0   3
Phalaenopsis-gene885719               1    0    0    1    0   2
Phalaenopsis-gene908065               1    1    1    1    1   5
Phalaenopsis-gene9464                 1    0    0    1    1   3
Phalaenopsis-gene230787               0    0    1    1    0   2
Phalaenopsis-gene568306               1    1    1    1    1   5
Phalaenopsis-gene670530               1    1    1    1    1   5
Phalaenopsis-gene876533               1    0    0    1    0   2
Phalaenopsis-gene145223               1    1    1    1    1   5
Phalaenopsis-gene33044                1    0    1    1    0   3
Phalaenopsis-gene469223               1    0    0    1    0   2
Phalaenopsis-gene476444               1    0    1    1    0   3
Phalaenopsis-gene476491               1    0    1    1    0   3
Phalaenopsis-gene488277               1    0    0    1    0   2
Phalaenopsis-gene706729               1    0    1    1    0   3
Phalaenopsis-gene780768               1    1    1    1    1   5
Phalaenopsis-gene119452               1    1    1    1    1   5
Phalaenopsis-gene184374               1    0    0    1    0   2
Phalaenopsis-gene306150               1    0    0    1    0   2
Phalaenopsis-gene728181               1    0    1    1    0   3
Phalaenopsis-gene122376               1    0    0    1    0   2
Phalaenopsis-gene129804               1    0    1    0    0   2
Phalaenopsis-gene138751               1    0    1    0    0   2
Phalaenopsis-gene169746               1    0    1    1    0   3
Phalaenopsis-gene189074               1    1    1    1    1   5
Phalaenopsis-gene192510               1    0    0    1    0   2
Phalaenopsis-gene197887               1    1    1    1    1   5
Phalaenopsis-gene201114               1    1    1    1    1   5
Phalaenopsis-gene201551               1    1    1    1    1   5
Phalaenopsis-gene201565               1    1    1    1    1   5
Phalaenopsis-gene217417               1    0    0    0    0   1
Phalaenopsis-gene223769               1    1    1    1    1   5
Phalaenopsis-gene22963                1    1    1    1    1   5
Phalaenopsis-gene231259               1    1    1    1    1   5
Phalaenopsis-gene259974               1    0    1    0    0   2
Phalaenopsis-gene279768               1    1    1    1    1   5
Phalaenopsis-gene292415               1    1    1    1    1   5
Phalaenopsis-gene325292               0    0    0    0    0   0
Phalaenopsis-gene338592               0    0    1    1    0   2
Phalaenopsis-gene344611               0    0    0    1    0   1
Phalaenopsis-gene348728               1    1    1    1    0   4
Phalaenopsis-gene363287               0    0    0    0    0   0
Phalaenopsis-gene36430                1    1    1    1    1   5
Phalaenopsis-gene374613               1    1    1    1    1   5
Phalaenopsis-gene393883               1    0    0    1    0   2
Phalaenopsis-gene405436               1    1    1    1    1   5
Phalaenopsis-gene43485                1    1    1    1    1   5
Phalaenopsis-gene446736               1    0    1    1    0   3
Phalaenopsis-gene456986               1    1    1    1    1   5
Phalaenopsis-gene479515               1    0    1    1    0   3
Phalaenopsis-gene493064               1    1    1    1    1   5
Phalaenopsis-gene520728               1    1    1    1    1   5
Phalaenopsis-gene538074               1    1    1    1    1   5
Phalaenopsis-gene558116               1    0    1    0    0   2
Phalaenopsis-gene585511               1    0    0    1    0   2
Phalaenopsis-gene588961               1    0    0    0    0   1
Phalaenopsis-gene593108               1    0    1    1    0   3
Phalaenopsis-gene594722               1    0    1    1    0   3
Phalaenopsis-gene594780               1    1    1    1    1   5
Phalaenopsis-gene597891               1    1    1    1    1   5
Phalaenopsis-gene60664                1    0    1    0    0   2
Phalaenopsis-gene611495               1    0    1    0    0   2
Phalaenopsis-gene629301               1    0    1    1    0   3
Phalaenopsis-gene648774               1    1    1    1    1   5
Phalaenopsis-gene662782               1    1    1    1    1   5
Phalaenopsis-gene687041               1    1    1    1    1   5
Phalaenopsis-gene714                  1    1    1    1    1   5
Phalaenopsis-gene716751               1    1    0    1    0   3
Phalaenopsis-gene742124               1    0    0    0    0   1
Phalaenopsis-gene751324               1    1    1    1    1   5
Phalaenopsis-gene765802               0    0    1    1    0   2
Phalaenopsis-gene780234               1    0    0    1    0   2
Phalaenopsis-gene795005               0    0    1    1    0   2
Phalaenopsis-gene820040               1    0    0    0    1   2
Phalaenopsis-gene823852               0    0    1    1    0   2
Phalaenopsis-gene835969               1    0    0    0    0   1
Phalaenopsis-gene839780               1    1    1    1    1   5
Phalaenopsis-gene846606               1    1    1    1    1   5
Phalaenopsis-gene847528               1    0    1    1    0   3
Phalaenopsis-gene865473               0    0    0    0    0   0
Phalaenopsis-gene898909               0    0    1    1    0   2
Phalaenopsis-gene905644               0    0    0    1    0   1
Phalaenopsis-gene97144                1    1    1    1    1   5
Phalaenopsis-gene151974               0    1    1    1    0   3
Phalaenopsis-gene206923               1    0    0    0    0   1
Phalaenopsis-gene239692               1    1    1    1    1   5
Phalaenopsis-gene255718               1    0    0    1    0   2
Phalaenopsis-gene306196               1    1    0    0    0   2
Phalaenopsis-gene319649               1    0    1    1    1   4
Phalaenopsis-gene339842               1    0    0    1    0   2
Phalaenopsis-gene347338               1    1    1    1    1   5
Phalaenopsis-gene398147               0    0    1    1    0   2
Phalaenopsis-gene413801               1    0    1    1    0   3
Phalaenopsis-gene522103               1    0    0    0    0   1
Phalaenopsis-gene65336                1    0    0    1    0   2
Phalaenopsis-gene734997               1    1    1    1    1   5
Phalaenopsis-gene846609               1    0    1    1    0   3
Phalaenopsis-gene9383                 0    0    0    1    0   1
Phalaenopsis-gene145399               1    1    1    1    1   5
Phalaenopsis-gene222528               0    0    1    1    0   2
Phalaenopsis-gene223056               0    1    1    1    0   3
Phalaenopsis-gene348792               1    0    1    1    0   3
Phalaenopsis-gene413882               1    0    1    1    0   3
Phalaenopsis-gene614579               1    1    1    1    1   5
Phalaenopsis-gene633384               1    0    1    1    0   3
Phalaenopsis-gene694124               1    0    0    1    0   2
Phalaenopsis-gene796232               1    1    1    1    1   5
Phalaenopsis-gene839565               1    0    0    1    0   2
Phalaenopsis-gene839741               1    1    1    1    0   4
Phalaenopsis-gene107363               1    1    1    1    1   5
Phalaenopsis-gene122899               1    1    1    1    1   5
Phalaenopsis-gene148836               1    1    1    1    1   5
Phalaenopsis-gene159116               1    0    0    1    0   2
Phalaenopsis-gene186907               0    0    1    1    0   2
Phalaenopsis-gene205950               0    0    1    1    0   2
Phalaenopsis-gene217420               0    0    1    1    0   2
Phalaenopsis-gene265661               1    1    1    1    0   4
Phalaenopsis-gene270193               1    1    1    1    1   5
Phalaenopsis-gene280133               1    0    1    1    0   3
Phalaenopsis-gene289320               1    1    1    1    1   5
Phalaenopsis-gene290160               1    1    1    1    1   5
Phalaenopsis-gene293606               1    0    0    1    0   2
Phalaenopsis-gene299286               0    0    1    1    0   2
Phalaenopsis-gene32324                1    1    1    1    1   5
Phalaenopsis-gene324615               1    0    0    1    0   2
Phalaenopsis-gene326491               1    0    0    0    0   1
Phalaenopsis-gene341725               1    0    0    0    0   1
Phalaenopsis-gene348803               1    1    1    1    1   5
Phalaenopsis-gene349471               1    0    0    1    0   2
Phalaenopsis-gene354756               1    1    1    1    1   5
Phalaenopsis-gene362907               1    1    1    1    1   5
Phalaenopsis-gene372180               0    0    1    0    0   1
Phalaenopsis-gene38322                0    1    1    1    0   3
Phalaenopsis-gene391030               1    1    1    1    1   5
Phalaenopsis-gene394135               1    0    0    1    0   2
Phalaenopsis-gene39846                1    0    0    0    0   1
Phalaenopsis-gene405038               1    0    1    1    0   3
Phalaenopsis-gene42501                1    0    1    0    0   2
Phalaenopsis-gene457321               1    0    1    1    0   3
Phalaenopsis-gene479545               1    0    0    1    0   2
Phalaenopsis-gene479574               0    0    0    1    0   1
Phalaenopsis-gene479817               0    1    1    0    0   2
Phalaenopsis-gene509660               1    1    1    1    1   5
Phalaenopsis-gene522142               1    0    0    1    0   2
Phalaenopsis-gene543422               1    0    1    1    0   3
Phalaenopsis-gene546584               1    1    1    1    1   5
Phalaenopsis-gene551501               1    0    1    0    0   2
Phalaenopsis-gene551857               1    1    1    1    1   5
Phalaenopsis-gene575613               1    0    1    0    0   2
Phalaenopsis-gene588956               1    0    1    1    0   3
Phalaenopsis-gene588973               1    1    1    1    1   5
Phalaenopsis-gene594030               1    1    1    1    1   5
Phalaenopsis-gene594471               1    0    1    1    0   3
Phalaenopsis-gene605244               1    0    0    0    0   1
Phalaenopsis-gene626116               1    0    1    1    0   3
Phalaenopsis-gene629324               1    1    1    1    1   5
Phalaenopsis-gene630192               1    0    0    1    0   2
Phalaenopsis-gene657921               0    0    1    0    0   1
Phalaenopsis-gene670531               1    0    1    0    0   2
Phalaenopsis-gene754642               1    0    1    1    0   3
Phalaenopsis-gene825722               1    1    1    1    1   5
Phalaenopsis-gene831780               1    1    0    1    0   3
Phalaenopsis-gene84827                0    0    1    0    0   1
Phalaenopsis-gene868265               1    0    0    1    0   2
Phalaenopsis-gene876678               1    0    0    0    0   1
Phalaenopsis-gene137336               1    1    1    1    1   5
Phalaenopsis-gene137500               1    0    0    0    0   1
Phalaenopsis-gene146288               1    0    1    0    0   2
Phalaenopsis-gene149969               0    0    0    1    0   1
Phalaenopsis-gene149996               1    0    0    0    0   1
Phalaenopsis-gene150025               0    0    0    1    0   1
Phalaenopsis-gene156156               1    1    1    1    1   5
Phalaenopsis-gene22520                0    0    1    1    0   2
Phalaenopsis-gene388142               1    1    1    1    1   5
Phalaenopsis-gene511852               1    1    1    1    1   5
Phalaenopsis-gene557527               1    0    0    0    0   1
Phalaenopsis-gene583390               1    0    1    0    0   2
Phalaenopsis-gene61107                1    0    1    1    0   3
Phalaenopsis-gene643249               1    1    1    1    1   5
Phalaenopsis-gene690054               0    0    0    1    0   1
Phalaenopsis-gene174894               1    0    1    0    0   2
Phalaenopsis-gene232754               1    0    1    0    0   2
Phalaenopsis-gene258387               0    1    1    1    0   3
Phalaenopsis-gene444137               1    0    0    1    0   2
Phalaenopsis-gene105013               1    1    0    0    0   2
Phalaenopsis-gene115945               1    1    1    1    1   5
Phalaenopsis-gene242331               0    0    0    1    0   1
Phalaenopsis-gene414622               1    1    1    1    1   5
Phalaenopsis-gene476285               1    0    0    1    0   2
Phalaenopsis-gene482386               1    1    1    1    1   5
Phalaenopsis-gene518581               0    0    0    1    0   1
Phalaenopsis-gene118512               1    1    1    1    0   4
Phalaenopsis-gene130039               1    0    0    1    0   2
Phalaenopsis-gene144946               1    0    1    0    0   2
Phalaenopsis-gene155734               1    1    1    1    1   5
Phalaenopsis-gene163939               1    1    1    0    0   3
Phalaenopsis-gene165005               0    1    1    1    0   3
Phalaenopsis-gene169697               1    0    1    1    0   3
Phalaenopsis-gene170895               1    0    0    0    0   1
Phalaenopsis-gene1801                 1    0    0    1    0   2
Phalaenopsis-gene184666               1    0    0    1    0   2
Phalaenopsis-gene202415               1    0    1    0    0   2
Phalaenopsis-gene206855               0    0    1    1    0   2
Phalaenopsis-gene209211               1    1    1    1    1   5
Phalaenopsis-gene22986                1    1    1    1    1   5
Phalaenopsis-gene256174               1    1    1    1    1   5
Phalaenopsis-gene296619               1    1    1    1    1   5
Phalaenopsis-gene299209               1    1    1    1    1   5
Phalaenopsis-gene351867               1    0    0    1    0   2
Phalaenopsis-gene361604               0    0    0    1    0   1
Phalaenopsis-gene364897               1    1    1    1    1   5
Phalaenopsis-gene370874               1    0    0    1    0   2
Phalaenopsis-gene378107               1    0    0    1    0   2
Phalaenopsis-gene399577               0    0    1    1    0   2
Phalaenopsis-gene399616               1    0    1    1    0   3
Phalaenopsis-gene408376               1    1    0    1    0   3
Phalaenopsis-gene408684               1    1    1    1    1   5
Phalaenopsis-gene410934               1    1    1    1    1   5
Phalaenopsis-gene412745               1    0    1    1    0   3
Phalaenopsis-gene430769               1    0    0    1    1   3
Phalaenopsis-gene434180               0    1    0    1    0   2
Phalaenopsis-gene43749                1    0    1    1    0   3
Phalaenopsis-gene443161               1    1    1    1    1   5
Phalaenopsis-gene444203               0    0    1    1    0   2
Phalaenopsis-gene475241               1    0    1    0    0   2
Phalaenopsis-gene482046               1    0    1    0    0   2
Phalaenopsis-gene516539               0    0    1    1    0   2
Phalaenopsis-gene517952               1    0    1    0    0   2
Phalaenopsis-gene518582               1    0    1    0    0   2
Phalaenopsis-gene536701               1    0    1    1    0   3
Phalaenopsis-gene543780               0    1    1    1    0   3
Phalaenopsis-gene560999               1    1    0    1    0   3
Phalaenopsis-gene588972               0    0    1    0    0   1
Phalaenopsis-gene593106               1    1    1    1    1   5
Phalaenopsis-gene626058               0    0    1    1    1   3
Phalaenopsis-gene638074               1    1    1    1    1   5
Phalaenopsis-gene65368                0    0    0    1    0   1
Phalaenopsis-gene662899               1    0    1    0    0   2
Phalaenopsis-gene670468               1    1    1    0    0   3
Phalaenopsis-gene682101               0    0    0    0    0   0
Phalaenopsis-gene704926               1    0    0    1    0   2
Phalaenopsis-gene725501               0    0    0    0    1   1
Phalaenopsis-gene732320               1    0    1    1    0   3
Phalaenopsis-gene749562               1    1    1    1    1   5
Phalaenopsis-gene763262               1    0    0    0    0   1
Phalaenopsis-gene764037               1    1    1    1    1   5
Phalaenopsis-gene766208               0    0    1    0    0   1
Phalaenopsis-gene773093               0    0    0    0    0   0
Phalaenopsis-gene793288               1    0    1    1    0   3
Phalaenopsis-gene805641               1    1    1    1    1   5
Phalaenopsis-gene816282               1    1    1    1    1   5
Phalaenopsis-gene837385               0    0    0    0    0   0
Phalaenopsis-gene839641               1    0    1    1    0   3
Phalaenopsis-gene841526               1    0    1    0    0   2
Phalaenopsis-gene849402               1    1    1    1    1   5
Phalaenopsis-gene859320               0    1    1    1    0   3
Phalaenopsis-gene876512               0    0    0    1    0   1
Phalaenopsis-gene904225               1    0    1    1    0   3
Phalaenopsis-gene96069                0    0    1    1    0   2
Phalaenopsis-gene97179                1    0    0    1    0   2
Ophrys-gene149233                     1    0    0    1    0   2
Ophrys-gene183939                     1    0    1    1    0   3
Ophrys-gene189959                     1    1    1    1    1   5
Ophrys-gene190374                     1    0    0    1    0   2
Ophrys-gene214092                     1    1    1    1    1   5
Ophrys-gene248813                     1    1    1    1    1   5
Ophrys-gene272821                     1    0    1    1    0   3
Ophrys-gene299722                     1    0    1    1    0   3
Ophrys-gene360853                     1    1    1    1    1   5
Ophrys-gene411596                     1    1    1    1    1   5
Ophrys-gene413227                     1    1    0    0    0   2
Ophrys-gene413919                     1    1    0    0    0   2
Ophrys-gene435638                     1    1    1    1    1   5
Ophrys-gene440275                     1    1    1    1    1   5
Ophrys-gene505287                     1    0    1    1    0   3
Ophrys-gene546711                     1    0    0    0    0   1
Ophrys-gene597523                     1    1    1    1    1   5
Ophrys-gene612200                     1    0    0    1    0   2
Ophrys-gene636279                     1    1    1    1    1   5
Ophrys-gene697502                     1    1    1    1    1   5
Ophrys-gene717275                     1    1    1    1    1   5
Ophrys-gene3447                       1    0    0    1    0   2
Ophrys-gene3471                       1    1    1    1    1   5
Ophrys-gene3946                       1    0    0    1    0   2
Ophrys-gene10589                      1    0    0    1    0   2
Ophrys-gene23219                      0    0    0    1    0   1
Ophrys-gene51117                      1    1    1    1    1   5
Ophrys-gene65442                      1    0    0    1    0   2
Ophrys-gene68901                      1    1    1    1    1   5
Ophrys-gene112087                     1    0    0    0    0   1
Ophrys-gene171058                     1    1    1    1    1   5
Ophrys-gene194104                     1    0    0    0    0   1
Ophrys-gene220442                     1    1    1    1    0   4
Ophrys-gene253063                     1    1    1    1    1   5
Ophrys-gene258514                     1    0    0    0    0   1
Ophrys-gene271086                     0    1    0    1    0   2
Ophrys-gene317373                     1    1    1    1    1   5
Ophrys-gene317410                     1    1    1    1    1   5
Ophrys-gene378078                     1    1    1    1    1   5
Ophrys-gene396676                     1    0    0    1    0   2
Ophrys-gene448062                     1    0    0    0    0   1
Ophrys-gene498145                     0    0    0    1    0   1
Ophrys-gene510611                     1    1    1    1    1   5
Ophrys-gene537475                     1    1    1    1    1   5
Ophrys-gene554821                     1    0    1    1    0   3
Ophrys-gene656779                     0    0    1    0    0   1
Ophrys-gene656930                     0    0    0    1    0   1
Ophrys-gene709062                     1    0    1    1    0   3
Ophrys-gene258373                     0    0    0    1    0   1
Ophrys-gene302510                     1    0    0    0    0   1
Ophrys-gene306698                     0    0    0    0    0   0
Ophrys-gene536141                     1    0    0    0    0   1
Ophrys-gene200648                     1    0    0    0    0   1
Ophrys-gene68460                      1    0    1    1    0   3
Ophrys-gene189201                     1    1    1    1    1   5
Ophrys-gene306775                     1    0    1    1    0   3
Ophrys-gene380147                     1    1    1    1    1   5
Ophrys-gene386189                     1    0    0    1    0   2
Ophrys-gene397847                     0    0    0    0    0   0
Ophrys-gene514000                     1    0    0    1    0   2
Ophrys-gene614319                     1    1    0    0    0   2
Ophrys-gene10667                      1    1    1    1    1   5
Ophrys-gene51312                      1    0    0    1    0   2
Ophrys-gene56847                      1    1    1    1    1   5
Ophrys-gene97449                      1    1    1    1    1   5
Ophrys-gene100044                     1    1    0    0    0   2
Ophrys-gene102178                     0    0    1    0    0   1
Ophrys-gene170373                     1    0    1    0    0   2
Ophrys-gene184174                     1    0    0    0    0   1
Ophrys-gene207430                     1    1    1    1    1   5
Ophrys-gene258363                     0    1    0    1    0   2
Ophrys-gene258554                     1    1    1    1    1   5
Ophrys-gene258610                     0    0    1    1    0   2
Ophrys-gene258651                     0    0    0    1    0   1
Ophrys-gene318860                     1    1    1    1    1   5
Ophrys-gene349576                     0    0    0    0    0   0
Ophrys-gene392085                     0    0    0    1    0   1
Ophrys-gene496516                     0    0    0    0    0   0
Ophrys-gene536726                     1    0    0    1    1   3
Ophrys-gene571323                     1    1    1    1    1   5
Ophrys-gene619525                     1    0    1    0    0   2
Ophrys-gene619549                     0    0    0    1    0   1
Ophrys-gene629937                     1    1    1    1    1   5
Ophrys-gene681279                     0    0    1    1    0   2
Ophrys-gene708054                     1    0    0    0    0   1
Ophrys-gene709675                     1    1    1    1    1   5
Ophrys-gene753537                     1    0    1    1    0   3
Ophrys-gene757378                     0    0    0    0    0   0
Ophrys-gene758884                     1    0    0    1    0   2
Ophrys-gene57671                      1    0    0    1    0   2
Ophrys-gene74502                      1    1    1    1    1   5
Ophrys-gene24186                      1    1    1    1    1   5
Ophrys-gene247246                     1    1    1    1    1   5
Ophrys-gene400271                     1    1    1    1    1   5
Ophrys-gene537947                     1    0    0    1    0   2
Ophrys-gene8620                       1    0    0    0    0   1
Ophrys-gene66906                      1    1    1    1    1   5
Ophrys-gene67551                      1    1    1    1    0   4
Ophrys-gene67611                      1    1    1    1    0   4
Ophrys-gene92366                      0    0    0    1    0   1
Ophrys-gene190011                     0    0    0    0    1   1
Ophrys-gene203601                     1    0    0    1    0   2
Ophrys-gene441105                     1    1    1    0    0   3
Ophrys-gene586729                     0    0    1    0    0   1
Ophrys-gene616765                     1    0    0    0    0   1
Ophrys-gene616795                     0    0    0    1    0   1
Ophrys-gene758414                     1    0    0    1    0   2
Ophrys-gene364                        1    1    1    1    1   5
Ophrys-gene1298                       1    0    0    0    0   1
Ophrys-gene4889                       1    0    1    1    0   3
Ophrys-gene7692                       1    1    0    1    0   3
Ophrys-gene9328                       1    1    1    1    1   5
Ophrys-gene22091                      1    0    0    1    0   2
Ophrys-gene24413                      1    1    1    1    1   5
Ophrys-gene59150                      1    0    0    0    0   1
Ophrys-gene73043                      1    1    1    1    0   4
Ophrys-gene94905                      1    0    1    1    1   4
Ophrys-gene106579                     1    1    1    1    1   5
Ophrys-gene112183                     1    1    1    1    1   5
Ophrys-gene133884                     1    0    0    1    0   2
Ophrys-gene139712                     0    0    1    1    0   2
Ophrys-gene149283                     1    1    1    1    1   5
Ophrys-gene173231                     1    0    1    1    0   3
Ophrys-gene199094                     1    1    0    0    0   2
Ophrys-gene211207                     1    1    1    1    1   5
Ophrys-gene215386                     0    0    1    1    0   2
Ophrys-gene228316                     1    0    0    1    0   2
Ophrys-gene238930                     0    0    1    1    0   2
Ophrys-gene263856                     1    1    1    1    1   5
Ophrys-gene272825                     1    1    1    1    1   5
Ophrys-gene284681                     1    0    0    1    0   2
Ophrys-gene300034                     1    0    0    1    0   2
Ophrys-gene300503                     1    0    1    0    0   2
Ophrys-gene341050                     1    1    1    1    1   5
Ophrys-gene345734                     1    0    0    0    0   1
Ophrys-gene377615                     0    0    1    1    0   2
Ophrys-gene392594                     1    0    0    1    0   2
Ophrys-gene397846                     0    0    1    1    0   2
Ophrys-gene478660                     1    1    1    1    1   5
Ophrys-gene498143                     1    1    1    1    1   5
Ophrys-gene518807                     1    1    1    1    1   5
Ophrys-gene657020                     1    0    0    0    0   1
Ophrys-gene689735                     1    0    0    0    0   1
Ophrys-gene709253                     1    1    1    1    1   5
Ophrys-gene710845                     1    1    1    1    1   5
Ophrys-gene762862                     1    1    1    1    1   5
Ophrys-gene205389                     1    1    1    1    1   5
Ophrys-gene273810                     1    1    1    0    0   3
Ophrys-gene341969                     1    1    1    1    1   5
Ophrys-gene123208                     1    1    1    1    1   5
Ophrys-gene138820                     1    1    1    1    1   5
Ophrys-gene160116                     1    0    0    1    0   2
Ophrys-gene274608                     1    0    0    1    0   2
Ophrys-gene434821                     1    1    1    1    1   5
Ophrys-gene221391                     1    1    1    1    1   5
Ophrys-gene272979                     1    1    1    0    0   3
Ophrys-gene521924                     0    0    1    1    0   2
Ophrys-gene670577                     1    1    1    1    1   5
Ophrys-gene675381                     1    0    0    1    0   2
Ophrys-gene4735                       1    1    1    1    0   4
Ophrys-gene8588                       1    0    1    0    0   2
Ophrys-gene24460                      1    1    1    1    1   5
Ophrys-gene70005                      1    1    0    1    0   3
Ophrys-gene113102                     1    1    1    1    1   5
Ophrys-gene244517                     1    1    1    1    1   5
Ophrys-gene264044                     1    1    1    1    1   5
Ophrys-gene276443                     1    0    0    1    0   2
Ophrys-gene276483                     1    1    0    1    0   3
Ophrys-gene276598                     1    1    1    1    1   5
Ophrys-gene277347                     0    0    1    1    0   2
Ophrys-gene304641                     1    1    1    1    1   5
Ophrys-gene364265                     0    0    1    0    0   1
Ophrys-gene410651                     0    0    0    0    0   0
Ophrys-gene425278                     1    1    1    1    1   5
Ophrys-gene432717                     1    0    0    1    0   2
Ophrys-gene432929                     1    0    0    1    0   2
Ophrys-gene440568                     1    1    1    1    1   5
Ophrys-gene547676                     1    0    0    1    0   2
Ophrys-gene612520                     0    0    0    1    0   1
Ophrys-gene61413                      1    1    1    1    1   5
Ophrys-gene240131                     1    0    0    1    0   2
Ophrys-gene252637                     1    1    1    1    1   5
Ophrys-gene258719                     1    0    1    1    0   3
Ophrys-gene278346                     1    1    1    1    1   5
Ophrys-gene357063                     1    0    0    0    0   1
Ophrys-gene394141                     0    0    0    0    0   0
Ophrys-gene442637                     1    0    0    0    0   1
Ophrys-gene462547                     1    1    1    1    1   5
Ophrys-gene477108                     1    0    0    1    0   2
Ophrys-gene532879                     1    1    0    1    0   3
Ophrys-gene566372                     1    0    0    1    0   2
Ophrys-gene570063                     1    0    0    1    0   2
Ophrys-gene610037                     1    1    1    1    1   5
Ophrys-gene610157                     1    0    0    1    0   2
Ophrys-gene610215                     1    0    0    1    0   2
Ophrys-gene614820                     1    0    1    0    0   2
Ophrys-gene715343                     1    1    1    1    1   5
Ophrys-gene37                         0    0    1    1    0   2
Ophrys-gene73                         1    0    0    0    0   1
Ophrys-gene74                         0    0    0    1    0   1
Ophrys-gene77                         1    1    0    0    0   2
Phalaenopsis-gene103619               1    0    0    1    0   2
Phalaenopsis-gene150051               1    0    0    0    0   1
Phalaenopsis-gene194615               1    0    0    1    0   2
Phalaenopsis-gene267405               1    1    1    1    1   5
Phalaenopsis-gene326042               0    0    0    0    0   0
Phalaenopsis-gene339329               1    0    1    0    0   2
Phalaenopsis-gene339398               1    1    1    1    1   5
Phalaenopsis-gene348775               1    1    1    1    1   5
Phalaenopsis-gene365861               0    0    1    0    0   1
Phalaenopsis-gene38593                1    0    0    1    0   2
Phalaenopsis-gene455810               1    0    1    0    0   2
Phalaenopsis-gene500240               1    0    0    1    0   2
Phalaenopsis-gene630805               0    1    1    1    0   3
Phalaenopsis-gene653040               1    1    0    1    0   3
Phalaenopsis-gene772653               1    0    0    1    0   2
Phalaenopsis-gene77846                0    0    1    1    0   2
Phalaenopsis-gene792422               0    0    1    1    0   2
Phalaenopsis-gene792656               0    0    0    1    1   2
Phalaenopsis-gene812703               1    1    1    1    1   5
Phalaenopsis-gene129882               1    0    0    1    1   3
Phalaenopsis-gene130592               1    0    0    1    0   2
Phalaenopsis-gene149869               1    0    0    1    0   2
Phalaenopsis-gene240906               0    0    1    0    0   1
Phalaenopsis-gene244443               1    0    1    1    0   3
Phalaenopsis-gene258714               1    1    1    1    1   5
Phalaenopsis-gene259957               1    1    1    1    1   5
Phalaenopsis-gene36063                0    0    0    1    0   1
Phalaenopsis-gene36943                1    0    0    1    0   2
Phalaenopsis-gene391008               1    1    1    1    1   5
Phalaenopsis-gene398139               1    0    1    1    0   3
Phalaenopsis-gene409893               0    0    0    1    0   1
Phalaenopsis-gene413786               1    0    0    1    0   2
Phalaenopsis-gene430629               1    1    1    1    1   5
Phalaenopsis-gene433032               1    1    1    1    1   5
Phalaenopsis-gene471626               0    0    1    1    0   2
Phalaenopsis-gene483833               0    0    1    1    0   2
Phalaenopsis-gene500234               1    1    1    1    0   4
Phalaenopsis-gene501182               1    0    0    0    0   1
Phalaenopsis-gene525842               1    0    1    1    0   3
Phalaenopsis-gene555627               1    0    0    0    0   1
Phalaenopsis-gene594161               1    1    1    1    1   5
Phalaenopsis-gene629711               1    0    0    0    0   1
Phalaenopsis-gene630748               1    0    1    1    0   3
Phalaenopsis-gene662811               1    0    0    1    0   2
Phalaenopsis-gene744269               1    0    0    0    0   1
Phalaenopsis-gene77771                1    1    1    1    1   5
Phalaenopsis-gene781274               1    0    0    0    0   1
Phalaenopsis-gene783703               1    0    0    0    0   1
Phalaenopsis-gene819132               1    1    1    1    0   4
Phalaenopsis-gene839853               1    1    1    1    1   5
Phalaenopsis-gene872156               1    0    1    1    0   3
Phalaenopsis-gene873750               1    1    1    1    1   5
Phalaenopsis-gene2062                 1    0    1    1    0   3
Phalaenopsis-gene515897               1    0    0    1    0   2
Phalaenopsis-gene549111               0    0    0    1    0   1
Phalaenopsis-gene843787               1    0    0    1    0   2
Phalaenopsis-gene848545               0    0    0    1    0   1
Phalaenopsis-gene887246               1    1    1    1    1   5
Phalaenopsis-gene341716               0    0    0    0    0   0
Phalaenopsis-gene688353               1    0    1    1    0   3
Phalaenopsis-gene707706               1    0    1    1    0   3
Phalaenopsis-gene812154               0    0    0    0    0   0
Phalaenopsis-gene812206               1    0    0    1    0   2
Phalaenopsis-gene230760               1    1    1    1    1   5
Phalaenopsis-gene312148               1    1    1    1    1   5
Phalaenopsis-gene355321               1    0    0    1    0   2
Phalaenopsis-gene360646               1    0    0    0    0   1
Phalaenopsis-gene414212               1    0    0    1    0   2
Phalaenopsis-gene430019               1    0    0    0    0   1
Phalaenopsis-gene475189               1    0    1    1    0   3
Phalaenopsis-gene512123               1    0    1    0    0   2
Phalaenopsis-gene594750               1    1    1    0    0   3
Phalaenopsis-gene649465               1    1    1    1    1   5
Phalaenopsis-gene190016               1    0    1    0    0   2
Phalaenopsis-gene214733               0    0    1    0    0   1
Phalaenopsis-gene222379               1    0    0    0    0   1
Phalaenopsis-gene247257               1    0    0    0    0   1
Phalaenopsis-gene260514               1    1    1    1    1   5
Phalaenopsis-gene326487               1    1    1    1    1   5
Phalaenopsis-gene335881               1    1    1    1    1   5
Phalaenopsis-gene351476               1    0    1    1    0   3
Phalaenopsis-gene36655                1    0    1    1    0   3
Phalaenopsis-gene370947               0    0    1    1    0   2
Phalaenopsis-gene432963               1    0    1    1    0   3
Phalaenopsis-gene467216               0    0    0    1    0   1
Phalaenopsis-gene546574               1    0    1    0    0   2
Phalaenopsis-gene556365               1    0    1    0    0   2
Phalaenopsis-gene578764               1    0    0    0    0   1
Phalaenopsis-gene582549               1    1    1    1    1   5
Phalaenopsis-gene615937               1    0    1    1    0   3
Phalaenopsis-gene645447               0    0    0    1    0   1
Phalaenopsis-gene651928               1    0    1    0    0   2
Phalaenopsis-gene676206               0    1    0    1    1   3
Phalaenopsis-gene688250               1    0    0    1    0   2
Phalaenopsis-gene718831               1    1    1    0    0   3
Phalaenopsis-gene733135               1    0    1    1    0   3
Phalaenopsis-gene734461               1    0    1    1    0   3
Phalaenopsis-gene744573               1    1    1    1    1   5
Phalaenopsis-gene76046                0    0    0    0    0   0
Phalaenopsis-gene770635               1    0    1    1    0   3
Phalaenopsis-gene775494               1    1    1    1    1   5
Phalaenopsis-gene798394               0    0    0    1    0   1
Phalaenopsis-gene83819                1    1    1    1    1   5
Phalaenopsis-gene856114               0    0    0    1    0   1
Phalaenopsis-gene859310               0    0    1    0    0   1
Phalaenopsis-gene905113               1    0    0    1    0   2
Phalaenopsis-gene381633               1    0    1    1    0   3
Phalaenopsis-gene444140               1    0    1    1    0   3
Phalaenopsis-gene470460               1    0    1    0    0   2
Phalaenopsis-gene509654               1    1    1    1    1   5
Phalaenopsis-gene184328               0    0    1    1    0   2
Phalaenopsis-gene326725               1    1    1    1    1   5
Phalaenopsis-gene101421               1    1    1    1    1   5
Phalaenopsis-gene146402               1    1    1    1    1   5
Phalaenopsis-gene154107               1    1    1    1    1   5
Phalaenopsis-gene161838               1    1    1    1    0   4
Phalaenopsis-gene21033                1    0    1    0    0   2
Phalaenopsis-gene287229               1    0    0    1    0   2
Phalaenopsis-gene292555               0    0    0    0    0   0
Phalaenopsis-gene312207               1    1    1    1    1   5
Phalaenopsis-gene342222               1    0    0    1    0   2
Phalaenopsis-gene347814               1    0    1    1    0   3
Phalaenopsis-gene355445               1    1    0    1    0   3
Phalaenopsis-gene36350                1    1    0    0    0   2
Phalaenopsis-gene365005               1    0    1    1    0   3
Phalaenopsis-gene36801                1    0    1    0    0   2
Phalaenopsis-gene374237               1    0    1    1    0   3
Phalaenopsis-gene394032               1    1    1    1    0   4
Phalaenopsis-gene398881               0    0    0    1    0   1
Phalaenopsis-gene429897               0    1    1    0    0   2
Phalaenopsis-gene432797               1    0    0    0    0   1
Phalaenopsis-gene468366               0    0    0    1    1   2
Phalaenopsis-gene482060               0    0    1    1    0   2
Phalaenopsis-gene532786               1    1    1    1    1   5
Phalaenopsis-gene593024               1    0    1    1    0   3
Phalaenopsis-gene647288               0    0    0    1    0   1
Phalaenopsis-gene654391               0    0    0    0    0   0
Phalaenopsis-gene677321               1    1    1    1    0   4
Phalaenopsis-gene839541               1    0    1    1    0   3
Phalaenopsis-gene896895               1    1    1    1    1   5
Phalaenopsis-gene98830                1    0    1    1    0   3
Phalaenopsis-gene294752               1    1    0    1    0   3
Phalaenopsis-gene309099               1    1    1    1    1   5
Phalaenopsis-gene456541               1    0    0    1    0   2
Phalaenopsis-gene595898               1    0    0    1    0   2
Phalaenopsis-gene794965               1    0    1    0    0   2
Phalaenopsis-gene905277               1    0    1    1    0   3
Phalaenopsis-gene340249               1    0    0    1    0   2
Phalaenopsis-gene388067               0    0    0    1    1   2
Phalaenopsis-gene688319               0    0    0    1    0   1
Phalaenopsis-gene97806                1    0    0    1    0   2
Phalaenopsis-gene214497               1    0    1    1    0   3
Phalaenopsis-gene262619               0    0    0    1    0   1
Phalaenopsis-gene291791               0    0    0    0    0   0
Phalaenopsis-gene322360               1    0    0    1    0   2
Phalaenopsis-gene447340               1    1    1    1    1   5
Phalaenopsis-gene479848               1    0    1    1    0   3
Phalaenopsis-gene557526               1    1    1    1    1   5
Phalaenopsis-gene573226               0    0    0    1    0   1
Phalaenopsis-gene149866               1    1    0    1    0   3
Phalaenopsis-gene155372               1    1    1    1    1   5
Phalaenopsis-gene410741               1    0    1    1    0   3
Phalaenopsis-gene558086               1    1    1    1    1   5
Phalaenopsis-gene887242               0    0    0    0    0   0
Phalaenopsis-gene887289               0    1    0    0    0   1
Phalaenopsis-gene135529               1    0    1    1    0   3
Phalaenopsis-gene202822               0    0    0    0    0   0
Phalaenopsis-gene206889               1    0    1    1    0   3
Phalaenopsis-gene23012                1    1    1    0    0   3
Phalaenopsis-gene318711               1    0    0    1    0   2
Phalaenopsis-gene341632               1    0    1    1    0   3
Phalaenopsis-gene345939               1    0    0    0    0   1
Phalaenopsis-gene357874               1    0    0    1    0   2
Phalaenopsis-gene370922               1    1    1    1    1   5
Phalaenopsis-gene371426               1    1    1    1    1   5
Phalaenopsis-gene394201               1    0    1    1    1   4
Phalaenopsis-gene432854               0    0    0    1    0   1
Phalaenopsis-gene46249                1    1    1    1    1   5
Phalaenopsis-gene462896               0    0    1    0    0   1
Phalaenopsis-gene488023               0    0    0    0    0   0
Phalaenopsis-gene558249               1    1    1    1    1   5
Phalaenopsis-gene593067               1    0    1    0    0   2
Phalaenopsis-gene65435                1    1    1    1    1   5
Phalaenopsis-gene689674               1    1    0    0    0   2
Phalaenopsis-gene905101               1    0    0    1    0   2
Phalaenopsis-gene905223               1    0    0    1    0   2
Phalaenopsis-gene270178               1    1    1    1    1   5
Phalaenopsis-gene325313               1    1    1    1    1   5
Phalaenopsis-gene342293               1    0    1    1    0   3
Phalaenopsis-gene351282               0    0    0    0    0   0
Phalaenopsis-gene351355               0    0    0    1    0   1
Phalaenopsis-gene36345                1    0    1    1    1   4
Phalaenopsis-gene367938               0    0    0    1    0   1
Phalaenopsis-gene394167               1    0    1    1    0   3
Phalaenopsis-gene437866               1    1    1    1    1   5
Phalaenopsis-gene56262                1    1    1    1    1   5
Phalaenopsis-gene676296               1    0    0    1    0   2
Phalaenopsis-gene759949               1    0    0    1    0   2
Phalaenopsis-gene767987               1    0    0    1    0   2
Phalaenopsis-gene843752               1    1    1    1    1   5
Phalaenopsis-gene85174                1    0    1    1    0   3
Phalaenopsis-gene85175                0    0    0    0    0   0
Phalaenopsis-gene897675               1    0    1    1    0   3
Phalaenopsis-gene9459                 1    0    1    1    0   3
Ophrys-gene93                         1    0    0    0    0   1
Ophrys-gene294                        0    0    0    1    0   1
Ophrys-gene295                        1    1    1    1    1   5
Ophrys-gene427                        1    1    1    1    1   5
Ophrys-gene720                        1    1    1    1    1   5
Ophrys-gene787                        1    1    1    1    1   5
Ophrys-gene788                        1    1    1    1    1   5
Ophrys-gene932                        1    0    0    0    0   1
Ophrys-gene1088                       1    0    0    1    0   2
Ophrys-gene1120                       1    0    0    1    0   2
Ophrys-gene1145                       1    1    1    1    1   5
Ophrys-gene1166                       0    0    1    1    0   2
Ophrys-gene1299                       1    0    0    1    0   2
Ophrys-gene1306                       1    0    0    1    0   2
Ophrys-gene1499                       1    0    0    1    0   2
Ophrys-gene1572                       1    0    1    1    0   3
Ophrys-gene1752                       1    0    0    0    0   1
Ophrys-gene1753                       1    1    1    1    1   5
Ophrys-gene1816                       1    1    1    1    1   5
Ophrys-gene1873                       1    1    1    1    1   5
Ophrys-gene1881                       1    1    1    1    1   5
Ophrys-gene1882                       1    1    1    1    1   5
Ophrys-gene2021                       1    1    1    1    1   5
Ophrys-gene2038                       1    0    1    1    0   3
Ophrys-gene2041                       1    1    1    1    1   5
Ophrys-gene2050                       0    0    0    0    0   0
Ophrys-gene2097                       1    1    1    1    1   5
Ophrys-gene2100                       1    1    1    1    1   5
Ophrys-gene2123                       1    0    0    0    0   1
Ophrys-gene2172                       0    1    0    1    0   2
Ophrys-gene2225                       0    0    1    0    0   1
Ophrys-gene2398                       1    0    0    0    0   1
Ophrys-gene2399                       0    0    0    0    0   0
Ophrys-gene2420                       1    1    1    1    1   5
Ophrys-gene2676                       1    0    0    1    0   2
Ophrys-gene2677                       0    0    1    1    0   2
Ophrys-gene2684                       0    0    1    1    0   2
Ophrys-gene2686                       0    0    0    1    0   1
Ophrys-gene2751                       1    1    1    1    1   5
Ophrys-gene2833                       1    1    1    1    1   5
Ophrys-gene2827                       1    1    1    1    1   5
Ophrys-gene2837                       1    0    0    0    0   1
Ophrys-gene2853                       1    1    0    0    0   2
Ophrys-gene2981                       1    1    1    1    1   5
Ophrys-gene3053                       1    1    1    1    1   5
Ophrys-gene3054                       1    1    1    1    1   5
Ophrys-gene3117                       1    1    1    1    1   5
Ophrys-gene3136                       0    0    1    1    0   2
Ophrys-gene3153                       1    0    1    1    0   3
Ophrys-gene3192                       1    1    1    1    1   5
Ophrys-gene3348                       1    1    1    1    1   5
Ophrys-gene3349                       1    0    0    1    0   2
Ophrys-gene3353                       1    1    1    1    1   5
Ophrys-gene3364                       1    0    1    1    0   3
Ophrys-gene3370                       1    1    1    1    1   5
Ophrys-gene3446                       1    1    1    1    1   5
Ophrys-gene3451                       0    1    0    0    0   1
Ophrys-gene3636                       1    1    1    1    1   5
Ophrys-gene3711                       0    0    1    0    0   1
Ophrys-gene3731                       1    0    1    1    0   3
Ophrys-gene3893                       1    1    1    1    1   5
Ophrys-gene4101                       1    1    0    0    0   2
Ophrys-gene4102                       1    0    0    0    0   1
Ophrys-gene4131                       1    0    0    0    0   1
Ophrys-gene4164                       1    1    1    1    1   5
Ophrys-gene4166                       1    1    1    1    1   5
Ophrys-gene4172                       1    1    1    1    0   4
Ophrys-gene4173                       1    0    0    1    0   2
Ophrys-gene4313                       1    1    1    1    0   4
Ophrys-gene4314                       1    0    0    0    0   1
Ophrys-gene4327                       0    0    1    1    0   2
Ophrys-gene4328                       1    1    1    1    1   5
Ophrys-gene4459                       1    1    1    0    0   3
Ophrys-gene4462                       1    1    1    1    1   5
Ophrys-gene4538                       1    0    0    0    0   1
Ophrys-gene4702                       1    0    0    1    0   2
Ophrys-gene4703                       1    1    0    0    0   2
Ophrys-gene4713                       1    1    1    1    1   5
Ophrys-gene4734                       1    1    1    1    1   5
Ophrys-gene4743                       1    1    1    1    1   5
Ophrys-gene4744                       0    0    0    0    0   0
Ophrys-gene4681                       1    0    1    1    0   3
Ophrys-gene4682                       1    0    0    1    0   2
Ophrys-gene4784                       1    1    1    1    1   5
Ophrys-gene4785                       1    1    1    1    1   5
Ophrys-gene4821                       1    0    0    1    1   3
Ophrys-gene4822                       1    1    1    1    1   5
Ophrys-gene4888                       0    0    0    1    0   1
Ophrys-gene4890                       1    0    1    0    0   2
Ophrys-gene4965                       0    1    1    1    0   3
Ophrys-gene4987                       1    1    1    1    1   5
Ophrys-gene4993                       1    1    1    1    1   5
Ophrys-gene4994                       1    0    0    1    0   2
Ophrys-gene5023                       1    1    1    1    1   5
Ophrys-gene5024                       1    1    1    1    1   5
Ophrys-gene5096                       1    1    1    1    1   5
Ophrys-gene5104                       1    1    1    1    1   5
Ophrys-gene5107                       1    1    1    1    1   5
Ophrys-gene5124                       0    0    0    0    0   0
Ophrys-gene5158                       1    1    1    1    1   5
Ophrys-gene5159                       1    1    1    1    1   5
Ophrys-gene5160                       1    0    0    1    0   2
Ophrys-gene5161                       1    1    0    1    0   3
Ophrys-gene5186                       1    1    1    1    1   5
Ophrys-gene5188                       1    0    0    1    0   2
Ophrys-gene5189                       0    1    1    1    0   3
Ophrys-gene5192                       1    0    1    1    0   3
Ophrys-gene5254                       1    1    1    1    1   5
Ophrys-gene5255                       1    1    1    1    1   5
Ophrys-gene5256                       1    1    1    1    1   5
Ophrys-gene5257                       1    1    1    1    1   5
Ophrys-gene5274                       1    1    1    1    1   5
Ophrys-gene5279                       0    0    0    1    0   1
Ophrys-gene5347                       0    0    0    1    0   1
Ophrys-gene5385                       0    0    0    1    0   1
Ophrys-gene5529                       1    0    0    1    0   2
Ophrys-gene5591                       1    1    1    1    1   5
Ophrys-gene5600                       0    0    0    1    0   1
Ophrys-gene6034                       1    0    1    1    0   3
Ophrys-gene6008                       1    0    0    1    0   2
Ophrys-gene6011                       1    0    1    1    0   3
Ophrys-gene6100                       1    1    1    1    1   5
Ophrys-gene6184                       1    0    0    1    0   2
Ophrys-gene6178                       1    1    1    1    1   5
Ophrys-gene6284                       1    1    1    1    1   5
Ophrys-gene6365                       1    1    1    1    1   5
Ophrys-gene6650                       1    1    1    1    1   5
Ophrys-gene6652                       1    1    1    1    1   5
Ophrys-gene6713                       1    1    1    1    1   5
Ophrys-gene6714                       1    0    1    0    0   2
Ophrys-gene6778                       1    0    0    0    0   1
Ophrys-gene7121                       1    1    1    1    1   5
Ophrys-gene7122                       1    0    0    1    0   2
Ophrys-gene7124                       1    1    1    1    1   5
Ophrys-gene7148                       1    0    0    1    0   2
Ophrys-gene7151                       1    1    1    1    1   5
Ophrys-gene7184                       1    0    0    1    0   2
Ophrys-gene7173                       1    0    0    1    0   2
Ophrys-gene7177                       1    0    0    1    0   2
Ophrys-gene7369                       1    1    1    1    1   5
Ophrys-gene7370                       1    1    1    1    1   5
Ophrys-gene7400                       1    0    1    0    0   2
Ophrys-gene7527                       0    0    0    1    0   1
Ophrys-gene7543                       0    0    0    1    0   1
Ophrys-gene7544                       1    1    1    1    1   5
Ophrys-gene7607                       1    1    1    1    1   5
Ophrys-gene7824                       1    1    1    1    1   5
Ophrys-gene7907                       1    1    1    1    1   5
Ophrys-gene8100                       0    0    0    0    0   0
Ophrys-gene8101                       1    1    1    1    1   5
Ophrys-gene8102                       1    1    1    1    1   5
Ophrys-gene8159                       1    0    0    0    0   1
Ophrys-gene8293                       1    1    1    1    1   5
Ophrys-gene8294                       1    1    1    1    1   5
Ophrys-gene8317                       1    1    1    1    0   4
Ophrys-gene8318                       0    1    1    0    0   2
Ophrys-gene8320                       0    0    1    1    0   2
Ophrys-gene8330                       1    1    1    1    1   5
Ophrys-gene8332                       1    1    1    1    1   5
Ophrys-gene8336                       1    1    1    1    1   5
Ophrys-gene8343                       1    0    0    1    0   2
Ophrys-gene8311                       1    1    1    1    1   5
Ophrys-gene8527                       1    1    1    1    1   5
Ophrys-gene8528                       1    1    1    1    1   5
Ophrys-gene8510                       1    1    1    1    1   5
Ophrys-gene8593                       1    1    1    1    1   5
Ophrys-gene8608                       1    0    1    1    0   3
Ophrys-gene8611                       1    1    1    1    1   5
Ophrys-gene8612                       1    1    1    1    1   5
Ophrys-gene8613                       1    1    1    1    1   5
Ophrys-gene8621                       1    1    1    1    1   5
Ophrys-gene8585                       1    1    1    1    1   5
Ophrys-gene8586                       1    1    1    1    1   5
Ophrys-gene8839                       1    0    1    1    0   3
Ophrys-gene8840                       0    0    0    1    0   1
Ophrys-gene8916                       1    0    0    1    0   2
Ophrys-gene8929                       1    1    1    1    1   5
Ophrys-gene8930                       1    0    0    1    0   2
Ophrys-gene8973                       0    0    0    1    0   1
Ophrys-gene8987                       1    1    1    1    1   5
Ophrys-gene8990                       0    0    0    0    0   0
Ophrys-gene8966                       0    0    0    1    0   1
Ophrys-gene8970                       1    1    1    1    1   5
Ophrys-gene9049                       1    1    1    1    1   5
Ophrys-gene9082                       1    1    1    1    1   5
Ophrys-gene9109                       1    1    0    0    0   2
Ophrys-gene9117                       1    1    1    1    1   5
Ophrys-gene9146                       0    1    0    1    0   2
Ophrys-gene9147                       1    0    0    0    0   1
Ophrys-gene9177                       0    0    0    1    0   1
Ophrys-gene9227                       1    0    1    1    0   3
Ophrys-gene9228                       0    0    0    1    0   1
Ophrys-gene9322                       1    1    0    1    0   3
Ophrys-gene9323                       1    1    0    1    0   3
Ophrys-gene9295                       1    0    0    1    0   2
Ophrys-gene9297                       1    1    1    1    1   5
Ophrys-gene9301                       1    1    1    1    1   5
Ophrys-gene9386                       1    0    0    1    0   2
Ophrys-gene9396                       1    0    0    1    0   2
Ophrys-gene9397                       1    1    1    1    1   5
Ophrys-gene9611                       1    0    1    1    0   3
Ophrys-gene9597                       0    0    0    1    0   1
Ophrys-gene9598                       1    0    1    1    0   3
Ophrys-gene9607                       1    0    0    0    0   1
Ophrys-gene9642                       1    1    1    1    1   5
Ophrys-gene9643                       1    0    0    0    0   1
Ophrys-gene9662                       1    1    1    1    1   5
Ophrys-gene9706                       1    1    1    1    1   5
Ophrys-gene9827                       1    1    1    1    1   5
Ophrys-gene9828                       1    0    0    1    0   2
Ophrys-gene9933                       0    0    1    0    0   1
Ophrys-gene9934                       1    1    1    1    1   5
Ophrys-gene9993                       1    1    1    1    1   5
Ophrys-gene10007                      1    0    0    0    0   1
Ophrys-gene10010                      1    1    1    1    1   5
Ophrys-gene10068                      1    0    1    1    0   3
Ophrys-gene10073                      1    0    0    1    0   2
Ophrys-gene10131                      1    0    0    1    0   2
Ophrys-gene10102                      1    0    0    0    0   1
Ophrys-gene10108                      0    0    0    1    0   1
Ophrys-gene10304                      1    1    1    1    1   5
Ophrys-gene10288                      1    0    0    0    0   1
Ophrys-gene10393                      1    1    1    1    0   4
Ophrys-gene10394                      1    0    0    1    0   2
Ophrys-gene10412                      1    0    1    1    0   3
Ophrys-gene10480                      1    1    1    1    1   5
Ophrys-gene10547                      1    1    1    1    1   5
Ophrys-gene10548                      1    1    1    1    1   5
Ophrys-gene10555                      1    1    1    1    1   5
Ophrys-gene10620                      1    0    0    1    0   2
Ophrys-gene10621                      1    1    1    1    1   5
Ophrys-gene10622                      0    1    1    0    0   2
Ophrys-gene10668                      0    0    1    1    0   2
Ophrys-gene10725                      1    1    1    1    1   5
Ophrys-gene10711                      1    0    0    1    0   2
Ophrys-gene10714                      1    1    1    1    1   5
Ophrys-gene10753                      0    0    0    1    0   1
Ophrys-gene10754                      1    1    1    1    1   5
Ophrys-gene10924                      1    1    1    1    1   5
Ophrys-gene10934                      1    0    0    1    0   2
Ophrys-gene10902                      1    0    0    1    0   2
Ophrys-gene10904                      1    0    0    1    0   2
Ophrys-gene11104                      1    0    0    1    0   2
Ophrys-gene11116                      1    1    1    1    1   5
Ophrys-gene11119                      0    0    1    1    0   2
Ophrys-gene11120                      1    1    1    1    1   5
Ophrys-gene11096                      1    1    1    1    1   5
Ophrys-gene11097                      1    1    1    1    1   5
Ophrys-gene11199                      0    0    0    0    0   0
Ophrys-gene11259                      1    1    1    1    1   5
Ophrys-gene11385                      0    1    0    0    0   1
Ophrys-gene11386                      0    0    1    1    0   2
Ophrys-gene11445                      1    0    0    1    0   2
Ophrys-gene11446                      1    0    0    1    0   2
Ophrys-gene11572                      1    1    1    1    1   5
Ophrys-gene11593                      1    1    1    1    1   5
Ophrys-gene11573                      1    1    1    1    1   5
Ophrys-gene11589                      1    1    1    1    1   5
Ophrys-gene11669                      1    1    1    1    1   5
Ophrys-gene11670                      0    0    1    0    0   1
Ophrys-gene11700                      1    0    0    1    0   2
Ophrys-gene11701                      0    0    1    1    0   2
Ophrys-gene11706                      1    1    1    1    1   5
Ophrys-gene11738                      1    1    1    1    1   5
Ophrys-gene11742                      1    1    1    1    1   5
Ophrys-gene11970                      1    1    1    1    1   5
Ophrys-gene12019                      1    0    1    1    0   3
Ophrys-gene12113                      1    1    1    1    1   5
Ophrys-gene12121                      1    1    1    1    1   5
Ophrys-gene12123                      0    0    0    1    0   1
Ophrys-gene12138                      1    1    1    1    1   5
Ophrys-gene12148                      1    0    0    1    0   2
Ophrys-gene12168                      1    0    1    1    0   3
Ophrys-gene12178                      1    1    1    1    1   5
Ophrys-gene12193                      1    1    1    1    1   5
Ophrys-gene19687                      1    1    1    1    1   5
Ophrys-gene20063                      1    1    1    1    1   5
Ophrys-gene20069                      0    1    1    1    0   3
Ophrys-gene20191                      1    1    1    1    1   5
Ophrys-gene20240                      0    0    0    0    0   0
Ophrys-gene20214                      1    1    1    1    1   5
Ophrys-gene20346                      0    0    0    1    0   1
Ophrys-gene20285                      1    0    0    1    0   2
Ophrys-gene20286                      1    1    1    1    1   5
Ophrys-gene20296                      1    1    0    1    1   4
Ophrys-gene20463                      1    0    0    1    0   2
Ophrys-gene20627                      1    1    1    1    1   5
Ophrys-gene20605                      1    0    1    1    0   3
Ophrys-gene20648                      1    1    1    1    1   5
Ophrys-gene20849                      1    1    1    1    1   5
Ophrys-gene20874                      1    0    0    0    0   1
Ophrys-gene20888                      1    1    0    1    0   3
Ophrys-gene20892                      1    0    0    1    1   3
Ophrys-gene20893                      0    0    1    1    0   2
Ophrys-gene20907                      1    0    0    0    0   1
Ophrys-gene20908                      1    0    1    0    0   2
Ophrys-gene20915                      1    1    1    1    1   5
Ophrys-gene20917                      1    0    1    0    0   2
Ophrys-gene20947                      1    0    0    0    0   1
Ophrys-gene20948                      1    1    1    1    1   5
Ophrys-gene21010                      1    0    0    1    0   2
Ophrys-gene21030                      1    1    1    1    1   5
Ophrys-gene21139                      1    1    1    1    1   5
Ophrys-gene21222                      1    0    0    1    0   2
Ophrys-gene21223                      1    1    1    1    1   5
Ophrys-gene21352                      1    1    0    0    0   2
Ophrys-gene21355                      1    0    1    0    0   2
Ophrys-gene21356                      1    1    1    1    1   5
Ophrys-gene21344                      1    1    1    1    1   5
Ophrys-gene21448                      1    1    1    1    1   5
Ophrys-gene21449                      1    1    1    1    1   5
Ophrys-gene21473                      1    1    1    1    1   5
Ophrys-gene21557                      1    1    1    1    1   5
Ophrys-gene21620                      1    0    1    1    0   3
Ophrys-gene21809                      1    0    0    1    0   2
Ophrys-gene22113                      1    1    1    1    1   5
Ophrys-gene22139                      0    0    0    1    0   1
Ophrys-gene22299                      1    1    1    1    1   5
Ophrys-gene22348                      1    0    0    1    0   2
Ophrys-gene22366                      0    1    0    1    0   2
Ophrys-gene22481                      1    0    0    0    0   1
Ophrys-gene22659                      1    1    1    1    1   5
Ophrys-gene22660                      1    1    1    1    1   5
Ophrys-gene22700                      0    0    1    0    0   1
Ophrys-gene23059                      1    0    0    0    0   1
Ophrys-gene23100                      1    0    1    0    0   2
Ophrys-gene23223                      1    1    1    1    1   5
Ophrys-gene23286                      1    0    0    1    0   2
Ophrys-gene23366                      1    1    1    1    1   5
Ophrys-gene23367                      1    0    1    1    0   3
Ophrys-gene23530                      1    1    1    1    1   5
Ophrys-gene23531                      0    1    0    1    0   2
Ophrys-gene23682                      1    1    1    1    1   5
Ophrys-gene23703                      1    0    0    1    0   2
Ophrys-gene23736                      1    1    1    1    1   5
Ophrys-gene23797                      1    0    0    1    0   2
Ophrys-gene23827                      1    1    1    1    1   5
Ophrys-gene23878                      1    1    1    1    1   5
Ophrys-gene24110                      1    1    1    1    1   5
Ophrys-gene24161                      0    0    0    1    0   1
Ophrys-gene24187                      0    0    0    1    0   1
Ophrys-gene24275                      1    1    1    1    1   5
Ophrys-gene24390                      0    0    0    1    0   1
Ophrys-gene24391                      1    1    1    1    1   5
Ophrys-gene24417                      0    1    1    1    0   3
Ophrys-gene24459                      1    1    1    1    1   5
Ophrys-gene24468                      1    0    0    1    0   2
Ophrys-gene24492                      1    0    1    1    0   3
Ophrys-gene24628                      0    0    0    0    0   0
Ophrys-gene24630                      1    1    1    1    1   5
Ophrys-gene24631                      1    1    1    1    1   5
Ophrys-gene24803                      0    0    0    1    1   2
Ophrys-gene24976                      1    1    1    1    1   5
Ophrys-gene25249                      1    1    1    1    1   5
Ophrys-gene25733                      1    1    1    1    1   5
Ophrys-gene25734                      1    1    1    1    1   5
Ophrys-gene25774                      1    1    1    1    1   5
Ophrys-gene26381                      0    0    0    0    0   0
Ophrys-gene37265                      1    0    0    0    0   1
Ophrys-gene38099                      1    1    1    1    1   5
Ophrys-gene38176                      1    1    1    1    1   5
Ophrys-gene38260                      1    0    0    1    0   2
Ophrys-gene38281                      1    0    0    0    0   1
Ophrys-gene38726                      1    1    1    1    1   5
Ophrys-gene40734                      1    0    0    0    0   1
Ophrys-gene45676                      0    0    1    1    0   2
Ophrys-gene45659                      0    0    0    1    0   1
Ophrys-gene45663                      1    1    1    1    1   5
Ophrys-gene45664                      1    1    1    1    1   5
Ophrys-gene45669                      0    0    1    1    0   2
Ophrys-gene45671                      1    1    1    1    1   5
Ophrys-gene45781                      1    0    0    0    0   1
Ophrys-gene45782                      1    1    1    1    1   5
Ophrys-gene46705                      1    0    0    1    0   2
Ophrys-gene46815                      0    1    0    0    0   1
Ophrys-gene46816                      1    1    0    1    0   3
Ophrys-gene46871                      1    0    1    1    0   3
Ophrys-gene46873                      1    0    0    0    0   1
Ophrys-gene46874                      1    1    1    1    1   5
Ophrys-gene46893                      1    0    0    1    0   2
Ophrys-gene46937                      0    0    0    0    0   0
Ophrys-gene47226                      1    1    1    1    1   5
Ophrys-gene47227                      0    0    0    1    0   1
Ophrys-gene47217                      1    0    0    1    0   2
Ophrys-gene47419                      1    0    0    1    0   2
Ophrys-gene48097                      1    1    1    1    1   5
Ophrys-gene48116                      0    0    1    0    0   1
Ophrys-gene48119                      1    0    0    1    0   2
Ophrys-gene48158                      1    1    1    1    1   5
Ophrys-gene48181                      1    0    0    1    0   2
Ophrys-gene48542                      1    0    0    0    0   1
Ophrys-gene48675                      1    1    1    1    1   5
Ophrys-gene49060                      1    0    1    1    0   3
Ophrys-gene49319                      1    0    0    1    0   2
Ophrys-gene49320                      1    1    1    1    1   5
Ophrys-gene49656                      1    0    0    0    1   2
Ophrys-gene49919                      0    0    0    1    0   1
Ophrys-gene49956                      1    0    1    1    0   3
Ophrys-gene49942                      0    1    1    0    0   2
Ophrys-gene50605                      1    0    1    0    0   2
Ophrys-gene50607                      1    0    0    1    0   2
Ophrys-gene50608                      1    0    0    1    0   2
Ophrys-gene50636                      1    0    1    1    0   3
Ophrys-gene50639                      1    1    1    1    1   5
Ophrys-gene51118                      1    0    0    0    0   1
Ophrys-gene51311                      1    0    0    0    0   1
Ophrys-gene51369                      1    1    1    1    1   5
Ophrys-gene51359                      1    0    1    1    0   3
Ophrys-gene51360                      0    0    0    0    0   0
Ophrys-gene51467                      1    1    1    1    1   5
Ophrys-gene52110                      1    0    0    1    0   2
Ophrys-gene52331                      1    1    1    1    1   5
Ophrys-gene52337                      1    0    0    0    0   1
Ophrys-gene52338                      1    1    1    1    1   5
Ophrys-gene52378                      1    1    1    1    1   5
Ophrys-gene53269                      0    0    1    1    0   2
Ophrys-gene53377                      0    0    0    1    0   1
Ophrys-gene53386                      0    0    0    1    0   1
Ophrys-gene53509                      1    0    1    1    0   3
Ophrys-gene53510                      0    0    1    1    0   2
Ophrys-gene53544                      1    0    1    0    0   2
Ophrys-gene53976                      1    1    1    1    1   5
Ophrys-gene53977                      1    0    1    1    0   3
Ophrys-gene54050                      0    0    0    1    0   1
Ophrys-gene54140                      0    0    0    1    0   1
Ophrys-gene54127                      1    1    1    1    1   5
Ophrys-gene54172                      1    1    1    1    1   5
Ophrys-gene54173                      1    1    1    1    1   5
Ophrys-gene54180                      1    1    1    1    1   5
Ophrys-gene54187                      1    1    1    1    1   5
Ophrys-gene54400                      1    0    0    0    0   1
Ophrys-gene54381                      0    0    0    1    0   1
Ophrys-gene54385                      1    1    1    1    1   5
Ophrys-gene54813                      0    0    1    1    0   2
Ophrys-gene54932                      1    1    1    1    0   4
Ophrys-gene54933                      0    0    0    1    0   1
Ophrys-gene55649                      1    1    1    1    0   4
Ophrys-gene55690                      1    0    1    1    0   3
Ophrys-gene55689                      1    0    1    1    0   3
Ophrys-gene56092                      1    0    0    0    0   1
Ophrys-gene56521                      1    1    1    1    1   5
Ophrys-gene56711                      1    0    0    1    0   2
Ophrys-gene56716                      1    1    1    1    1   5
Ophrys-gene56844                      1    1    1    1    1   5
Ophrys-gene56848                      1    0    0    1    0   2
Ophrys-gene56871                      1    0    0    0    0   1
Ophrys-gene57411                      1    0    1    1    0   3
Ophrys-gene57449                      1    1    1    1    1   5
Ophrys-gene57450                      1    1    1    1    1   5
Ophrys-gene57546                      1    0    0    1    0   2
Ophrys-gene57547                      0    0    0    1    0   1
Ophrys-gene57604                      1    1    0    1    0   3
Ophrys-gene57689                      1    0    0    0    0   1
Ophrys-gene57690                      1    0    0    1    0   2
Ophrys-gene57670                      1    1    1    1    1   5
Ophrys-gene57929                      1    1    1    1    1   5
Ophrys-gene58258                      1    1    1    1    1   5
Ophrys-gene58259                      1    1    1    1    1   5
Ophrys-gene58600                      1    1    1    1    1   5
Ophrys-gene58697                      1    1    1    1    1   5
Ophrys-gene58868                      1    1    1    1    1   5
Ophrys-gene58861                      1    0    0    1    0   2
Ophrys-gene58887                      1    0    0    1    0   2
Ophrys-gene58894                      0    0    0    1    1   2
Ophrys-gene58895                      1    0    0    1    0   2
Ophrys-gene58926                      1    1    1    1    1   5
Ophrys-gene58927                      1    1    1    1    1   5
Ophrys-gene58965                      1    1    0    0    0   2
Ophrys-gene59151                      1    0    0    1    0   2
Ophrys-gene59467                      0    1    1    1    0   3
Ophrys-gene59580                      1    1    1    1    1   5
Ophrys-gene59965                      1    1    1    1    1   5
Ophrys-gene60225                      1    1    1    1    1   5
Ophrys-gene60385                      1    0    0    0    0   1
Ophrys-gene60536                      0    0    0    1    0   1
Ophrys-gene60543                      1    0    1    1    0   3
Ophrys-gene60569                      1    1    1    1    0   4
Ophrys-gene60570                      0    0    0    1    0   1
Ophrys-gene60576                      0    0    1    1    0   2
Ophrys-gene60746                      1    1    1    1    0   4
Ophrys-gene60730                      1    1    1    1    1   5
Ophrys-gene60912                      1    1    1    1    1   5
Ophrys-gene61412                      1    1    1    1    1   5
Ophrys-gene61414                      1    0    0    1    0   2
Ophrys-gene61419                      1    0    1    1    0   3
Ophrys-gene61536                      1    0    0    1    0   2
Ophrys-gene61537                      1    1    1    1    1   5
Ophrys-gene61771                      1    0    1    0    0   2
Ophrys-gene61774                      1    0    1    0    0   2
Ophrys-gene61781                      1    1    1    1    1   5
Ophrys-gene61754                      1    0    0    1    1   3
Ophrys-gene61838                      1    1    1    1    1   5
Ophrys-gene61943                      1    1    1    1    1   5
Ophrys-gene61942                      0    0    1    0    0   1
Ophrys-gene62115                      1    0    0    1    0   2
Ophrys-gene62124                      0    0    1    0    0   1
Ophrys-gene62498                      1    1    1    1    1   5
Ophrys-gene62623                      1    1    1    1    1   5
Ophrys-gene62625                      1    0    0    1    0   2
Ophrys-gene62636                      1    1    1    1    1   5
Ophrys-gene62676                      1    0    0    1    0   2
Ophrys-gene62677                      1    0    0    1    0   2
Ophrys-gene62684                      1    0    0    1    0   2
Ophrys-gene62764                      1    1    1    1    1   5
Ophrys-gene62765                      1    1    1    1    1   5
Ophrys-gene62953                      1    0    1    0    0   2
Ophrys-gene62955                      0    0    0    1    0   1
Ophrys-gene62973                      1    0    1    1    0   3
Ophrys-gene62976                      1    0    0    1    0   2
Ophrys-gene62982                      1    1    1    1    1   5
Ophrys-gene63018                      0    0    0    1    0   1
Ophrys-gene63209                      1    1    1    1    1   5
Ophrys-gene63424                      1    1    1    1    1   5
Ophrys-gene64487                      1    1    0    0    0   2
Ophrys-gene64850                      1    1    1    1    1   5
Ophrys-gene65138                      1    1    1    1    1   5
Ophrys-gene65213                      0    0    0    0    0   0
Ophrys-gene65319                      0    0    1    1    0   2
Ophrys-gene65434                      0    0    0    1    0   1
Ophrys-gene65486                      0    0    0    1    0   1
Ophrys-gene65864                      1    0    0    0    0   1
Ophrys-gene65920                      0    0    0    0    0   0
Ophrys-gene66979                      1    0    1    0    0   2
Ophrys-gene67438                      1    1    1    1    1   5
Ophrys-gene67439                      1    0    0    0    0   1
Ophrys-gene67441                      1    0    0    0    0   1
Ophrys-gene67493                      1    0    0    1    0   2
Ophrys-gene68384                      1    1    1    1    1   5
Ophrys-gene68398                      1    1    1    1    1   5
Ophrys-gene68446                      1    1    1    1    1   5
Ophrys-gene68448                      1    1    1    1    1   5
Ophrys-gene68456                      1    1    1    1    1   5
Ophrys-gene68459                      1    0    1    0    0   2
Ophrys-gene68465                      1    0    0    1    0   2
Ophrys-gene68405                      1    0    0    0    0   1
Ophrys-gene68406                      1    0    0    0    0   1
Ophrys-gene69221                      1    1    1    1    1   5
Ophrys-gene69222                      1    1    1    1    1   5
Ophrys-gene69300                      1    1    0    0    0   2
Ophrys-gene69301                      0    0    0    1    0   1
Ophrys-gene69315                      1    0    0    1    0   2
Ophrys-gene69316                      0    0    0    1    0   1
Ophrys-gene69879                      1    1    1    1    1   5
Ophrys-gene70615                      0    0    0    0    0   0
Ophrys-gene71242                      1    0    1    0    0   2
Ophrys-gene71249                      1    0    0    1    0   2
Ophrys-gene71255                      1    1    1    1    1   5
Ophrys-gene71236                      1    1    1    1    1   5
Ophrys-gene71817                      0    0    1    1    0   2
Ophrys-gene71836                      1    1    1    1    1   5
Ophrys-gene72070                      0    0    1    1    0   2
Ophrys-gene72088                      1    1    1    1    1   5
Ophrys-gene72060                      1    1    1    1    1   5
Ophrys-gene72064                      1    0    0    1    0   2
Ophrys-gene72113                      1    0    1    1    0   3
Ophrys-gene72114                      1    1    1    1    1   5
Ophrys-gene72656                      1    0    0    0    0   1
Ophrys-gene72722                      1    0    0    0    0   1
Ophrys-gene73023                      1    1    0    0    0   2
Ophrys-gene73024                      0    0    0    1    0   1
Ophrys-gene73011                      1    1    1    1    1   5
Ophrys-gene73968                      1    0    0    1    0   2
Ophrys-gene74200                      0    0    0    1    0   1
Ophrys-gene74194                      1    1    1    1    1   5
Ophrys-gene74836                      1    1    1    1    1   5
Ophrys-gene74837                      1    1    1    1    1   5
Ophrys-gene74872                      1    1    1    1    1   5
Ophrys-gene74829                      1    1    1    1    1   5
Ophrys-gene75059                      1    1    1    1    1   5
Ophrys-gene75054                      1    0    0    1    0   2
Ophrys-gene75174                      1    1    1    1    1   5
Ophrys-gene75167                      1    0    0    1    0   2
Ophrys-gene75169                      1    0    0    1    0   2
Ophrys-gene75170                      1    0    0    1    0   2
Ophrys-gene75342                      1    0    0    1    0   2
Ophrys-gene75343                      1    0    0    1    0   2
Ophrys-gene75561                      1    0    0    1    0   2
Ophrys-gene75697                      1    0    0    0    0   1
Ophrys-gene75698                      1    1    1    1    1   5
Ophrys-gene75723                      1    1    1    1    1   5
Ophrys-gene75770                      1    0    0    1    0   2
Ophrys-gene76262                      1    1    1    1    1   5
Ophrys-gene76319                      1    1    1    1    1   5
Ophrys-gene76325                      1    0    0    1    0   2
Ophrys-gene76602                      1    1    1    1    1   5
Ophrys-gene76686                      1    0    0    0    0   1
Ophrys-gene77031                      1    1    1    1    1   5
Ophrys-gene76854                      1    0    1    0    0   2
Ophrys-gene76855                      1    1    1    1    1   5
Ophrys-gene76856                      1    1    1    1    1   5
Ophrys-gene76857                      0    0    0    1    0   1
Ophrys-gene76864                      1    1    1    1    1   5
Ophrys-gene76872                      0    0    0    1    0   1
Ophrys-gene76873                      1    0    0    1    0   2
Ophrys-gene76882                      1    0    1    1    0   3
Ophrys-gene76888                      1    0    1    1    0   3
Ophrys-gene76924                      1    0    0    1    0   2
Ophrys-gene76938                      1    1    1    1    1   5
Ophrys-gene76945                      1    0    0    0    0   1
Ophrys-gene76953                      0    1    0    1    0   2
Ophrys-gene76971                      1    0    0    1    0   2
Ophrys-gene76981                      0    0    1    0    0   1
Ophrys-gene77000                      1    0    0    0    0   1
Ophrys-gene77220                      1    1    1    1    1   5
Ophrys-gene77828                      1    1    1    1    1   5
Ophrys-gene77824                      1    1    0    1    0   3
Ophrys-gene77901                      0    0    0    1    0   1
Ophrys-gene77971                      1    0    0    1    0   2
Ophrys-gene78006                      1    0    0    1    0   2
Ophrys-gene78229                      1    1    1    1    1   5
Ophrys-gene78230                      1    1    1    1    1   5
Ophrys-gene78372                      1    1    1    1    1   5
Ophrys-gene78373                      1    0    1    0    0   2
Ophrys-gene78681                      1    1    1    1    1   5
Ophrys-gene78790                      1    1    1    1    1   5
Ophrys-gene78792                      1    1    1    1    1   5
Ophrys-gene79964                      1    1    1    1    1   5
Ophrys-gene80502                      1    0    0    1    0   2
Ophrys-gene80511                      1    1    1    1    1   5
Ophrys-gene80524                      1    0    0    1    0   2
Ophrys-gene80558                      1    0    0    1    0   2
Ophrys-gene80580                      1    0    0    1    0   2
Ophrys-gene81078                      1    0    0    0    0   1
Ophrys-gene81085                      1    1    1    1    1   5
Ophrys-gene81086                      1    0    0    0    0   1
Ophrys-gene81588                      1    1    1    1    1   5
Ophrys-gene81589                      1    1    1    1    1   5
Ophrys-gene81631                      1    1    1    1    1   5
Ophrys-gene82079                      1    1    1    1    1   5
Ophrys-gene82081                      1    1    1    1    1   5
Ophrys-gene82086                      1    0    0    1    0   2
Ophrys-gene82088                      1    1    1    1    1   5
Ophrys-gene82093                      1    1    1    1    1   5
Ophrys-gene82094                      1    1    1    1    1   5
Ophrys-gene82064                      1    1    1    1    1   5
Ophrys-gene82918                      1    1    1    1    1   5
Ophrys-gene82919                      1    1    1    1    1   5
Ophrys-gene82911                      1    1    1    1    1   5
Ophrys-gene83740                      1    1    1    1    1   5
Ophrys-gene83764                      1    1    1    1    1   5
Ophrys-gene84013                      1    1    1    1    1   5
Ophrys-gene83998                      1    1    1    1    1   5
Ophrys-gene84185                      1    1    1    1    1   5
Ophrys-gene84207                      1    1    1    1    1   5
Ophrys-gene84582                      1    1    1    1    1   5
Ophrys-gene85397                      0    1    1    1    0   3
Ophrys-gene85598                      1    0    0    1    0   2
Ophrys-gene85599                      1    0    0    1    0   2
Ophrys-gene85955                      1    1    1    1    1   5
Ophrys-gene86270                      1    0    1    1    0   3
Ophrys-gene86267                      1    1    1    1    1   5
Ophrys-gene86418                      1    1    1    1    1   5
Ophrys-gene86833                      1    0    0    1    0   2
Ophrys-gene86863                      1    1    1    1    1   5
Ophrys-gene86965                      1    1    1    1    1   5
Ophrys-gene87873                      0    0    0    1    0   1
Ophrys-gene87874                      1    0    0    0    0   1
Ophrys-gene87881                      0    0    1    0    0   1
Ophrys-gene88144                      1    0    0    1    0   2
Ophrys-gene88414                      1    1    1    1    1   5
Ophrys-gene88639                      1    0    0    0    0   1
Ophrys-gene88949                      1    1    1    1    1   5
Ophrys-gene90414                      1    1    1    1    1   5
Ophrys-gene90422                      0    0    1    0    0   1
Ophrys-gene90424                      0    0    1    1    0   2
Ophrys-gene90425                      1    1    1    1    1   5
Ophrys-gene90397                      1    1    1    1    1   5
Ophrys-gene90398                      1    1    1    1    1   5
Ophrys-gene90404                      1    0    0    0    0   1
Ophrys-gene90405                      1    1    1    1    1   5
Ophrys-gene91039                      1    1    1    1    1   5
Ophrys-gene91040                      1    0    1    1    0   3
Ophrys-gene91662                      1    1    1    1    1   5
Ophrys-gene92060                      0    0    0    1    0   1
Ophrys-gene92061                      1    1    0    1    0   3
Ophrys-gene92091                      1    0    0    1    0   2
Ophrys-gene92284                      1    1    0    0    0   2
Ophrys-gene92283                      1    1    0    1    0   3
Ophrys-gene92312                      1    1    1    1    1   5
Ophrys-gene92340                      1    0    0    1    0   2
Ophrys-gene92367                      0    0    0    0    0   0
Ophrys-gene92778                      1    1    1    1    1   5
Ophrys-gene92976                      1    0    0    1    0   2
Ophrys-gene93334                      1    0    1    1    0   3
Ophrys-gene93912                      1    0    0    1    0   2
Ophrys-gene94156                      0    1    1    0    0   2
Ophrys-gene94164                      1    1    1    1    1   5
Ophrys-gene94890                      0    0    0    0    0   0
Ophrys-gene96322                      0    0    1    1    0   2
Ophrys-gene96323                      1    1    1    1    1   5
Ophrys-gene97051                      1    1    1    1    1   5
Ophrys-gene97448                      0    0    0    1    0   1
Ophrys-gene97739                      1    0    1    1    0   3
Ophrys-gene98451                      1    0    0    1    0   2
Ophrys-gene98819                      1    1    1    1    1   5
Ophrys-gene99009                      1    0    0    1    0   2
Ophrys-gene98991                      0    0    0    0    0   0
Ophrys-gene99088                      1    1    1    1    1   5
Ophrys-gene99071                      1    0    0    0    0   1
Ophrys-gene99224                      1    1    1    1    1   5
Ophrys-gene99898                      1    0    0    1    0   2
Ophrys-gene99902                      1    0    0    1    0   2
Ophrys-gene99938                      1    0    0    1    0   2
Ophrys-gene99939                      1    1    1    1    1   5
Ophrys-gene99944                      1    1    1    1    1   5
Ophrys-gene99978                      1    0    0    0    0   1
Ophrys-gene99982                      0    0    0    1    0   1
Ophrys-gene99964                      1    1    1    1    1   5
Ophrys-gene100017                     1    0    0    1    0   2
Ophrys-gene100021                     0    0    0    1    0   1
Ophrys-gene100023                     0    0    0    0    0   0
Ophrys-gene100059                     1    0    0    1    0   2
Ophrys-gene100062                     1    1    0    1    0   3
Ophrys-gene100054                     1    0    1    1    0   3
Ophrys-gene100055                     1    1    1    1    1   5
Ophrys-gene100630                     1    0    1    0    0   2
Ophrys-gene100867                     1    0    0    1    0   2
Ophrys-gene100839                     0    0    1    1    0   2
Ophrys-gene100891                     1    1    1    1    1   5
Ophrys-gene100978                     0    1    0    1    0   2
Ophrys-gene100979                     1    1    1    1    1   5
Ophrys-gene101214                     0    0    1    1    0   2
Ophrys-gene101215                     1    1    1    1    1   5
Ophrys-gene101547                     1    1    1    1    1   5
Ophrys-gene101549                     0    0    0    1    0   1
Ophrys-gene101990                     1    1    1    1    1   5
Ophrys-gene102127                     1    1    1    1    1   5
Ophrys-gene102128                     0    0    1    1    0   2
Ophrys-gene102171                     1    0    0    1    0   2
Ophrys-gene102223                     1    1    1    1    1   5
Ophrys-gene102643                     1    1    1    1    1   5
Ophrys-gene103678                     1    0    1    1    0   3
Ophrys-gene103684                     0    0    0    1    0   1
Ophrys-gene103685                     0    0    0    1    0   1
Ophrys-gene103991                     1    0    0    0    0   1
Ophrys-gene104090                     1    1    1    1    1   5
Ophrys-gene104527                     0    1    0    0    0   1
Ophrys-gene104614                     1    1    1    1    1   5
Ophrys-gene104646                     1    0    0    1    0   2
Ophrys-gene104677                     1    0    0    1    0   2
Ophrys-gene105317                     1    1    1    1    0   4
Ophrys-gene105320                     1    0    1    1    0   3
Ophrys-gene105321                     1    0    0    1    0   2
Ophrys-gene105361                     1    1    1    1    1   5
Ophrys-gene105364                     1    1    1    1    1   5
Ophrys-gene105365                     0    0    0    1    0   1
Ophrys-gene105588                     1    0    0    1    0   2
Ophrys-gene105589                     1    0    1    1    0   3
Ophrys-gene105585                     1    0    0    0    0   1
Ophrys-gene105821                     1    0    1    1    0   3
Ophrys-gene105898                     1    1    1    1    1   5
Ophrys-gene106556                     1    1    1    1    1   5
Ophrys-gene106544                     1    1    1    1    1   5
Ophrys-gene107733                     1    0    0    0    0   1
Ophrys-gene107749                     1    1    0    1    0   3
Ophrys-gene107905                     0    0    0    0    0   0
Ophrys-gene107919                     1    0    0    1    0   2
Ophrys-gene107920                     1    0    1    1    0   3
Ophrys-gene108032                     0    0    0    0    0   0
Ophrys-gene108161                     1    0    1    1    0   3
Ophrys-gene108188                     1    1    1    1    1   5
Ophrys-gene108201                     1    1    1    1    1   5
Ophrys-gene108544                     0    0    0    1    0   1
Ophrys-gene108545                     1    1    1    1    1   5
Ophrys-gene108951                     1    1    1    1    1   5
Ophrys-gene109007                     1    0    0    1    0   2
Ophrys-gene109012                     1    0    0    1    0   2
Ophrys-gene109056                     1    1    1    1    0   4
Ophrys-gene109094                     1    1    0    0    0   2
Ophrys-gene109338                     1    1    1    1    1   5
Ophrys-gene109409                     1    1    1    1    1   5
Ophrys-gene109412                     0    0    1    1    0   2
Ophrys-gene110019                     1    1    1    1    1   5
Ophrys-gene110404                     1    0    1    1    0   3
Ophrys-gene110405                     1    1    1    1    1   5
Ophrys-gene110487                     0    0    0    1    0   1
Ophrys-gene110626                     1    0    0    1    0   2
Ophrys-gene110627                     1    1    1    1    1   5
Ophrys-gene110806                     1    0    0    1    0   2
Ophrys-gene111088                     1    1    1    1    1   5
Ophrys-gene111089                     1    1    1    1    1   5
Ophrys-gene111090                     1    1    1    1    1   5
Ophrys-gene111851                     0    0    1    1    0   2
Ophrys-gene111887                     0    0    1    1    0   2
Ophrys-gene112093                     1    1    1    1    1   5
Ophrys-gene112191                     1    1    1    1    0   4
Ophrys-gene112192                     1    1    1    0    0   3
Ophrys-gene112205                     1    1    1    1    1   5
Ophrys-gene112217                     1    1    1    1    1   5
Ophrys-gene112218                     1    1    1    1    1   5
Ophrys-gene112184                     1    1    1    1    1   5
Ophrys-gene112448                     1    1    1    1    1   5
Ophrys-gene112872                     1    1    1    1    1   5
Ophrys-gene113050                     1    1    1    1    1   5
Ophrys-gene113672                     1    1    1    1    1   5
Ophrys-gene113676                     1    0    0    1    0   2
Ophrys-gene113704                     1    0    0    1    0   2
Ophrys-gene113717                     1    1    1    1    1   5
Ophrys-gene113689                     1    1    1    1    1   5
Ophrys-gene113695                     0    1    0    1    0   2
Ophrys-gene114163                     1    1    1    1    1   5
Ophrys-gene114627                     1    0    0    1    0   2
Ophrys-gene114628                     0    0    0    0    0   0
Ophrys-gene114850                     1    0    1    1    0   3
Ophrys-gene114855                     1    1    1    1    1   5
Ophrys-gene114874                     1    1    1    1    1   5
Ophrys-gene114880                     1    1    1    1    1   5
Ophrys-gene114907                     1    1    1    1    1   5
Ophrys-gene114908                     1    1    1    1    1   5
Ophrys-gene114916                     1    0    0    0    0   1
Ophrys-gene115139                     1    1    1    1    1   5
Ophrys-gene115171                     1    1    1    1    1   5
Ophrys-gene116205                     1    1    1    1    1   5
Ophrys-gene116206                     1    1    1    1    1   5
Ophrys-gene116291                     1    1    1    1    1   5
Ophrys-gene116387                     1    1    1    1    1   5
Ophrys-gene116388                     1    1    1    1    1   5
Ophrys-gene117164                     1    0    0    1    0   2
Ophrys-gene117384                     1    0    1    1    0   3
Ophrys-gene117399                     0    0    0    0    0   0
Ophrys-gene117747                     1    1    1    1    1   5
Ophrys-gene118627                     1    0    0    1    0   2
Ophrys-gene118635                     1    1    1    1    1   5
Ophrys-gene118636                     1    0    0    1    0   2
Ophrys-gene118741                     1    1    1    1    1   5
Ophrys-gene118880                     1    0    1    1    0   3
Ophrys-gene118888                     0    0    0    1    0   1
Ophrys-gene118955                     1    0    0    0    0   1
Ophrys-gene120145                     1    1    1    1    1   5
Ophrys-gene120871                     1    1    1    1    1   5
Ophrys-gene120899                     1    1    1    1    1   5
Ophrys-gene120863                     1    0    0    0    0   1
Ophrys-gene120925                     1    1    1    1    1   5
Ophrys-gene120931                     1    1    1    1    1   5
Ophrys-gene120932                     0    0    0    0    0   0
Ophrys-gene121377                     1    1    1    1    1   5
Ophrys-gene121379                     1    0    1    1    0   3
Ophrys-gene121388                     1    0    0    1    0   2
Ophrys-gene121391                     0    0    0    1    0   1
Ophrys-gene121392                     0    0    0    1    0   1
Ophrys-gene122064                     1    0    0    1    0   2
Ophrys-gene122166                     1    0    0    1    1   3
Ophrys-gene122141                     1    0    0    1    0   2
Ophrys-gene122741                     1    1    0    0    0   2
Ophrys-gene123130                     1    1    1    1    1   5
Ophrys-gene123205                     1    0    0    0    0   1
Ophrys-gene123769                     1    1    1    1    1   5
Ophrys-gene124299                     1    1    1    1    1   5
Ophrys-gene124293                     1    1    1    1    1   5
Ophrys-gene124988                     1    1    1    1    1   5
Ophrys-gene124989                     1    1    1    1    1   5
Ophrys-gene124992                     1    1    1    1    1   5
Ophrys-gene125642                     1    0    0    1    1   3
Ophrys-gene125851                     1    0    0    1    0   2
Ophrys-gene125882                     1    0    0    0    0   1
Ophrys-gene126147                     1    1    1    1    1   5
Ophrys-gene126148                     1    1    1    1    1   5
Ophrys-gene126205                     1    0    1    1    0   3
Ophrys-gene126206                     1    1    1    1    1   5
Ophrys-gene126482                     1    1    1    1    0   4
Ophrys-gene126682                     1    1    1    1    1   5
Ophrys-gene126726                     1    1    1    1    1   5
Ophrys-gene126804                     1    1    1    1    1   5
Ophrys-gene127205                     1    0    1    1    0   3
Ophrys-gene127480                     1    1    1    1    1   5
Ophrys-gene127481                     1    1    1    1    1   5
Ophrys-gene127536                     1    0    1    0    0   2
Ophrys-gene127537                     1    1    1    1    1   5
Ophrys-gene127580                     1    0    0    0    0   1
Ophrys-gene127640                     1    0    0    0    0   1
Ophrys-gene127641                     1    0    1    1    0   3
Ophrys-gene127687                     1    0    0    1    0   2
Ophrys-gene127710                     1    0    0    0    0   1
Ophrys-gene128926                     0    1    0    0    0   1
Ophrys-gene128919                     1    1    1    1    1   5
Ophrys-gene130042                     1    1    1    1    1   5
Ophrys-gene130043                     1    1    0    1    0   3
Ophrys-gene130247                     1    0    0    1    0   2
Ophrys-gene130248                     1    0    0    1    0   2
Ophrys-gene130298                     1    0    0    1    0   2
Ophrys-gene130299                     1    1    1    1    1   5
Ophrys-gene130349                     1    0    0    1    0   2
Ophrys-gene130398                     0    0    0    1    0   1
Ophrys-gene130446                     1    0    0    1    0   2
Ophrys-gene130447                     0    1    0    1    0   2
Ophrys-gene130495                     0    0    0    1    0   1
Ophrys-gene130542                     1    0    0    1    0   2
Ophrys-gene130587                     1    0    0    1    0   2
Ophrys-gene130588                     1    0    0    0    0   1
Ophrys-gene130711                     1    0    0    1    0   2
Ophrys-gene130791                     0    0    0    1    0   1
Ophrys-gene131324                     1    0    0    1    0   2
Ophrys-gene131842                     1    1    1    1    1   5
Ophrys-gene131826                     1    1    1    1    1   5
Ophrys-gene131832                     1    1    0    1    0   3
Ophrys-gene131885                     0    0    1    1    0   2
Ophrys-gene131907                     1    0    0    0    0   1
Ophrys-gene132031                     0    0    1    1    0   2
Ophrys-gene132026                     1    0    1    1    0   3
Ophrys-gene132076                     0    0    0    1    0   1
Ophrys-gene132438                     1    0    0    0    0   1
Ophrys-gene132439                     0    0    0    0    0   0
Ophrys-gene132864                     1    0    0    1    0   2
Ophrys-gene132959                     1    0    0    1    0   2
Ophrys-gene133060                     1    1    1    1    1   5
Ophrys-gene133095                     1    1    1    1    1   5
Ophrys-gene133462                     1    1    0    0    0   2
Ophrys-gene133463                     1    1    1    1    1   5
Ophrys-gene133886                     1    0    0    0    0   1
Ophrys-gene134119                     1    1    1    1    1   5
Ophrys-gene134469                     1    1    1    1    0   4
Ophrys-gene134791                     1    1    1    1    1   5
Ophrys-gene134797                     1    0    0    1    0   2
Ophrys-gene134821                     1    1    1    1    0   4
Ophrys-gene134822                     0    0    1    1    0   2
Ophrys-gene134774                     1    1    1    1    1   5
Ophrys-gene134784                     1    1    1    1    1   5
Ophrys-gene134785                     1    0    0    1    0   2
Ophrys-gene134899                     0    0    1    0    0   1
Ophrys-gene134900                     1    0    0    1    0   2
Ophrys-gene134901                     1    0    1    1    0   3
Ophrys-gene134863                     0    0    0    1    0   1
Ophrys-gene134864                     0    0    0    1    0   1
Ophrys-gene134870                     1    1    1    1    1   5
Ophrys-gene134871                     1    0    0    0    0   1
Ophrys-gene136059                     1    0    0    1    0   2
Ophrys-gene136544                     1    0    0    0    0   1
Ophrys-gene136545                     1    0    0    1    0   2
Ophrys-gene136584                     1    0    0    1    0   2
Ophrys-gene136585                     1    1    1    1    1   5
Ophrys-gene136586                     1    1    1    1    1   5
Ophrys-gene136723                     1    0    0    1    1   3
Ophrys-gene136725                     1    0    0    1    0   2
Ophrys-gene136726                     1    0    1    1    0   3
Ophrys-gene136699                     1    1    1    1    1   5
Ophrys-gene136771                     1    1    1    1    1   5
Ophrys-gene136773                     1    1    0    1    0   3
Ophrys-gene136911                     1    0    0    1    0   2
Ophrys-gene137138                     1    0    0    1    0   2
Ophrys-gene137139                     1    1    1    1    1   5
Ophrys-gene137305                     0    0    1    1    0   2
Ophrys-gene137306                     1    1    1    1    1   5
Ophrys-gene137377                     1    1    1    1    1   5
Ophrys-gene137752                     1    1    0    0    0   2
Ophrys-gene137753                     1    0    1    1    0   3
Ophrys-gene137891                     1    1    0    1    0   3
Ophrys-gene137892                     0    0    0    1    0   1
Ophrys-gene138193                     1    0    1    1    0   3
Ophrys-gene138259                     1    1    1    1    1   5
Ophrys-gene138260                     1    1    1    1    1   5
Ophrys-gene138454                     0    0    0    1    0   1
Ophrys-gene138814                     1    1    1    1    1   5
Ophrys-gene138819                     1    1    1    1    1   5
Ophrys-gene139671                     1    0    1    1    0   3
Ophrys-gene139677                     1    0    0    1    0   2
Ophrys-gene139699                     1    1    1    1    1   5
Ophrys-gene139706                     1    1    0    0    0   2
Ophrys-gene139713                     1    1    1    1    1   5
Ophrys-gene139721                     1    0    0    1    0   2
Ophrys-gene139728                     1    0    0    1    0   2
Ophrys-gene139729                     1    0    1    1    0   3
Ophrys-gene139738                     1    1    1    1    1   5
Ophrys-gene139809                     1    1    1    1    1   5
Ophrys-gene140533                     1    0    1    1    0   3
Ophrys-gene140680                     1    0    0    1    0   2
Ophrys-gene140808                     0    0    1    0    0   1
Ophrys-gene140879                     0    0    1    1    0   2
Ophrys-gene140939                     1    0    0    0    0   1
Ophrys-gene140986                     1    0    0    0    0   1
Ophrys-gene141560                     1    1    1    1    1   5
Ophrys-gene141581                     1    0    1    1    0   3
Ophrys-gene141582                     1    1    1    1    1   5
Ophrys-gene141592                     1    0    0    0    0   1
Ophrys-gene141674                     1    0    0    1    0   2
Ophrys-gene141675                     0    1    1    1    0   3
Ophrys-gene141677                     1    0    1    1    0   3
Ophrys-gene141875                     1    0    0    1    0   2
Ophrys-gene141907                     1    1    1    1    1   5
Ophrys-gene142025                     1    1    1    1    1   5
Ophrys-gene142026                     1    1    1    1    1   5
Ophrys-gene142030                     1    1    1    1    1   5
Ophrys-gene142031                     1    1    1    1    1   5
Ophrys-gene142032                     1    1    1    1    1   5
Ophrys-gene142033                     1    0    1    1    0   3
Ophrys-gene141988                     1    1    1    1    1   5
Ophrys-gene142098                     1    1    1    1    1   5
Ophrys-gene142099                     1    1    1    1    1   5
Ophrys-gene142435                     1    1    1    1    1   5
Ophrys-gene142438                     0    0    1    1    0   2
Ophrys-gene142418                     1    0    1    1    0   3
Ophrys-gene142503                     0    0    0    0    0   0
Ophrys-gene142505                     1    0    0    0    1   2
Ophrys-gene142566                     0    1    0    1    0   2
Ophrys-gene142568                     1    0    0    1    0   2
Ophrys-gene142569                     0    0    1    0    0   1
Ophrys-gene143288                     1    1    1    1    1   5
Ophrys-gene143296                     0    0    1    1    0   2
Ophrys-gene143298                     1    1    1    1    1   5
Ophrys-gene143307                     1    1    1    0    0   3
Ophrys-gene143314                     1    0    0    1    0   2
Ophrys-gene143319                     0    0    1    1    0   2
Ophrys-gene143556                     1    1    1    1    1   5
Ophrys-gene144369                     1    0    0    0    0   1
Ophrys-gene144476                     1    1    1    1    1   5
Ophrys-gene144744                     1    1    1    1    1   5
Ophrys-gene144842                     1    1    1    1    1   5
Ophrys-gene145221                     1    1    1    1    1   5
Ophrys-gene145222                     1    0    0    1    0   2
Ophrys-gene145417                     0    0    0    1    0   1
Ophrys-gene145516                     1    1    1    1    1   5
Ophrys-gene145570                     1    1    1    1    1   5
Ophrys-gene145571                     1    1    1    1    1   5
Ophrys-gene145920                     0    0    0    1    0   1
Ophrys-gene145921                     1    1    1    1    1   5
Ophrys-gene145922                     1    0    0    1    0   2
Ophrys-gene145923                     1    0    0    0    0   1
Ophrys-gene147803                     1    0    0    0    0   1
Ophrys-gene147897                     0    0    0    1    0   1
Ophrys-gene147901                     1    1    1    1    1   5
Ophrys-gene147956                     0    0    1    0    0   1
Ophrys-gene147957                     1    1    1    1    1   5
Ophrys-gene147964                     1    1    1    1    1   5
Ophrys-gene147968                     1    1    1    1    1   5
Ophrys-gene149015                     1    1    1    1    1   5
Ophrys-gene149037                     1    1    1    1    1   5
Ophrys-gene149010                     1    1    1    1    1   5
Ophrys-gene149078                     1    0    0    1    0   2
Ophrys-gene149150                     1    0    0    0    0   1
Ophrys-gene149123                     1    0    0    0    0   1
Ophrys-gene149128                     1    0    0    1    0   2
Ophrys-gene149191                     1    0    0    1    0   2
Ophrys-gene149248                     1    1    0    1    0   3
Ophrys-gene149251                     1    1    1    1    1   5
Ophrys-gene149253                     1    0    1    1    0   3
Ophrys-gene149978                     0    1    0    0    0   1
Ophrys-gene149974                     1    1    1    1    1   5
Ophrys-gene149975                     1    0    0    1    0   2
Ophrys-gene150011                     1    0    1    0    0   2
Ophrys-gene150007                     0    0    0    1    0   1
Ophrys-gene150008                     1    0    0    1    1   3
Ophrys-gene150084                     1    1    1    1    1   5
Ophrys-gene150255                     0    0    0    0    0   0
Ophrys-gene151332                     1    1    1    1    1   5
Ophrys-gene151333                     1    1    1    1    1   5
Ophrys-gene151356                     1    1    1    1    1   5
Ophrys-gene151912                     1    1    1    1    1   5
Ophrys-gene151913                     1    1    1    1    1   5
Ophrys-gene151924                     1    0    1    1    0   3
Ophrys-gene152275                     1    1    1    1    1   5
Ophrys-gene152675                     1    1    1    1    1   5
Ophrys-gene153768                     1    0    0    0    0   1
Ophrys-gene153829                     1    0    0    1    0   2
Ophrys-gene153836                     1    1    1    1    1   5
Ophrys-gene153904                     1    0    0    0    0   1
Ophrys-gene153905                     1    1    1    1    1   5
Ophrys-gene153934                     1    1    1    1    1   5
Ophrys-gene153962                     1    1    0    0    0   2
Ophrys-gene154547                     0    1    1    1    0   3
Ophrys-gene154549                     1    1    1    1    1   5
Ophrys-gene154550                     1    0    0    1    0   2
Ophrys-gene154587                     1    0    0    1    0   2
Ophrys-gene154580                     1    1    1    1    1   5
Ophrys-gene154626                     1    1    1    1    0   4
Ophrys-gene154660                     1    1    1    1    1   5
Ophrys-gene154698                     1    1    1    1    1   5
Ophrys-gene154933                     1    1    1    1    1   5
Ophrys-gene154934                     0    0    0    1    0   1
Ophrys-gene154920                     1    1    1    1    0   4
Ophrys-gene154962                     1    0    0    1    0   2
Ophrys-gene154949                     0    0    0    0    0   0
Ophrys-gene154951                     1    1    1    1    1   5
Ophrys-gene154952                     1    1    1    1    1   5
Ophrys-gene154953                     1    1    1    1    1   5
Ophrys-gene154983                     1    1    1    1    1   5
Ophrys-gene154998                     0    0    1    0    0   1
Ophrys-gene155003                     1    1    1    1    1   5
Ophrys-gene155004                     1    0    0    1    0   2
Ophrys-gene155312                     0    0    0    0    0   0
Ophrys-gene155320                     1    1    1    1    1   5
Ophrys-gene155358                     1    1    1    1    1   5
Ophrys-gene155359                     1    1    1    1    1   5
Ophrys-gene155360                     1    1    1    1    1   5
Ophrys-gene155361                     1    1    1    1    1   5
Ophrys-gene155367                     1    1    1    1    1   5
Ophrys-gene155368                     1    0    0    0    0   1
Ophrys-gene155372                     1    1    1    1    1   5
Ophrys-gene155403                     1    0    0    1    0   2
Ophrys-gene155475                     1    1    1    1    1   5
Ophrys-gene155533                     0    0    1    1    0   2
Ophrys-gene155952                     1    0    1    1    0   3
Ophrys-gene155954                     1    1    1    0    0   3
Ophrys-gene155955                     1    1    1    1    1   5
Ophrys-gene155962                     1    1    1    1    1   5
Ophrys-gene155982                     1    1    1    1    1   5
Ophrys-gene155983                     0    0    0    1    0   1
Ophrys-gene155986                     1    1    1    1    1   5
Ophrys-gene156610                     1    1    1    1    1   5
Ophrys-gene157139                     0    0    0    0    0   0
Ophrys-gene157144                     1    0    0    0    0   1
Ophrys-gene157179                     0    0    0    1    0   1
Ophrys-gene157184                     0    0    0    1    0   1
Ophrys-gene157514                     1    0    0    0    0   1
Ophrys-gene157515                     0    0    0    1    0   1
Ophrys-gene157976                     1    1    1    1    1   5
Ophrys-gene158064                     1    1    1    1    1   5
Ophrys-gene158085                     0    0    0    0    0   0
Ophrys-gene158424                     1    0    0    1    0   2
Ophrys-gene158725                     1    1    1    1    1   5
Ophrys-gene158730                     0    0    1    1    0   2
Ophrys-gene158731                     1    0    0    0    0   1
Ophrys-gene158724                     1    1    1    1    1   5
Ophrys-gene159117                     0    0    0    1    0   1
Ophrys-gene159125                     1    1    1    1    1   5
Ophrys-gene159154                     0    0    1    1    0   2
Ophrys-gene159207                     1    0    0    0    0   1
Ophrys-gene159220                     1    1    1    1    1   5
Ophrys-gene159229                     0    0    1    1    0   2
Ophrys-gene159968                     1    0    1    1    0   3
Ophrys-gene159969                     0    0    0    0    0   0
Ophrys-gene159999                     1    1    1    1    0   4
Ophrys-gene160030                     0    0    0    1    0   1
Ophrys-gene160059                     1    0    1    0    1   3
Ophrys-gene160088                     1    0    1    1    0   3
Ophrys-gene160115                     1    1    1    0    0   3
Ophrys-gene160145                     1    0    1    0    0   2
Ophrys-gene160136                     1    1    1    1    1   5
Ophrys-gene160195                     1    1    1    1    1   5
Ophrys-gene160206                     1    0    0    1    0   2
Ophrys-gene160453                     1    1    1    1    1   5
Ophrys-gene160688                     1    1    1    1    1   5
Ophrys-gene160768                     1    0    1    1    0   3
Ophrys-gene160770                     0    0    0    1    0   1
Ophrys-gene160826                     0    0    0    0    0   0
Ophrys-gene162739                     1    1    1    1    1   5
Ophrys-gene162742                     1    1    1    1    1   5
Ophrys-gene162785                     1    1    1    1    1   5
Ophrys-gene162943                     0    0    0    1    0   1
Ophrys-gene162944                     0    0    0    1    0   1
Ophrys-gene163654                     1    1    1    1    1   5
Ophrys-gene163733                     1    1    1    1    1   5
Ophrys-gene163867                     1    1    1    1    1   5
Ophrys-gene164410                     1    0    1    1    0   3
Ophrys-gene164381                     1    1    1    1    1   5
Ophrys-gene164506                     0    0    0    1    1   2
Ophrys-gene164461                     1    1    1    1    1   5
Ophrys-gene164462                     1    0    0    1    0   2
Ophrys-gene164463                     1    0    1    0    0   2
Ophrys-gene164466                     1    0    1    1    0   3
Ophrys-gene164604                     1    1    1    1    1   5
Ophrys-gene165847                     1    1    1    1    1   5
Ophrys-gene166304                     1    1    1    1    1   5
Ophrys-gene166464                     1    0    0    0    0   1
Ophrys-gene166530                     1    0    1    0    0   2
Ophrys-gene166519                     1    0    1    1    1   4
Ophrys-gene166638                     1    0    1    1    0   3
Ophrys-gene166640                     1    0    1    1    0   3
Ophrys-gene166802                     1    1    1    1    1   5
Ophrys-gene166943                     1    0    1    0    0   2
Ophrys-gene166932                     1    1    1    1    1   5
Ophrys-gene166933                     1    1    1    1    1   5
Ophrys-gene167045                     1    1    1    1    1   5
Ophrys-gene167052                     1    1    1    1    1   5
Ophrys-gene167243                     1    0    0    0    0   1
Ophrys-gene167234                     1    1    0    1    0   3
Ophrys-gene167695                     1    1    1    1    1   5
Ophrys-gene167699                     1    1    1    1    1   5
Ophrys-gene167713                     1    1    1    1    1   5
Ophrys-gene168106                     1    1    1    1    1   5
Ophrys-gene168107                     1    0    1    1    0   3
Ophrys-gene168097                     1    1    1    1    1   5
Ophrys-gene168164                     1    0    1    1    0   3
Ophrys-gene168165                     1    1    1    1    1   5
Ophrys-gene168155                     1    0    1    1    0   3
Ophrys-gene168516                     1    1    1    1    1   5
Ophrys-gene168526                     1    0    1    0    0   2
Ophrys-gene168576                     1    0    1    0    0   2
Ophrys-gene168604                     1    1    1    1    1   5
Ophrys-gene168660                     0    0    0    1    0   1
Ophrys-gene168820                     1    0    1    1    0   3
Ophrys-gene168826                     1    0    0    0    0   1
Ophrys-gene168847                     1    1    1    1    1   5
Ophrys-gene169454                     0    0    0    0    0   0
Ophrys-gene169606                     1    1    1    1    1   5
Ophrys-gene170262                     1    1    1    1    1   5
Ophrys-gene170269                     1    0    0    1    0   2
Ophrys-gene170270                     0    0    1    1    0   2
Ophrys-gene170374                     1    1    1    1    1   5
Ophrys-gene170419                     1    0    1    1    0   3
Ophrys-gene170783                     1    1    1    1    1   5
Ophrys-gene170784                     1    1    1    0    0   3
Ophrys-gene170938                     1    0    0    1    0   2
Ophrys-gene171061                     1    1    1    1    1   5
Ophrys-gene171085                     1    1    0    1    0   3
Ophrys-gene171386                     1    0    0    1    0   2
Ophrys-gene172279                     1    0    1    1    0   3
Ophrys-gene172516                     1    0    1    1    0   3
Ophrys-gene172517                     1    1    1    0    0   3
Ophrys-gene173261                     1    0    0    1    0   2
Ophrys-gene173262                     1    1    1    1    1   5
Ophrys-gene173266                     1    1    1    1    1   5
Ophrys-gene173268                     1    1    1    1    1   5
Ophrys-gene173269                     1    1    1    1    1   5
Ophrys-gene173272                     0    0    0    1    0   1
Ophrys-gene173274                     1    1    1    1    1   5
Ophrys-gene173283                     1    1    1    1    1   5
Ophrys-gene173235                     1    1    1    1    1   5
Ophrys-gene173576                     1    1    1    1    1   5
Ophrys-gene173587                     1    0    0    1    0   2
Ophrys-gene173588                     0    0    0    1    0   1
Ophrys-gene173571                     1    0    0    1    0   2
Ophrys-gene173624                     1    0    0    1    0   2
Ophrys-gene173625                     1    1    1    1    1   5
Ophrys-gene173608                     1    1    1    1    1   5
Ophrys-gene173613                     1    0    0    0    0   1
Ophrys-gene173782                     1    0    0    1    0   2
Ophrys-gene173820                     1    1    1    1    1   5
Ophrys-gene173969                     1    0    0    0    0   1
Ophrys-gene174022                     1    1    1    1    1   5
Ophrys-gene174179                     1    1    1    1    1   5
Ophrys-gene174180                     1    1    1    1    1   5
Ophrys-gene174333                     0    0    0    1    0   1
Ophrys-gene174836                     1    1    1    1    1   5
Ophrys-gene175063                     1    1    1    1    1   5
Ophrys-gene175065                     0    0    0    1    0   1
Ophrys-gene175066                     1    0    0    1    0   2
Ophrys-gene175406                     1    1    1    1    1   5
Ophrys-gene175844                     1    1    1    1    1   5
Ophrys-gene175911                     0    0    0    1    0   1
Ophrys-gene175912                     1    1    1    1    1   5
Ophrys-gene176161                     0    0    0    1    0   1
Ophrys-gene176162                     1    1    1    1    1   5
Ophrys-gene176174                     1    1    1    1    1   5
Ophrys-gene176234                     1    0    0    1    0   2
Ophrys-gene176235                     1    1    0    1    0   3
Ophrys-gene176247                     1    0    0    1    0   2
Ophrys-gene176454                     1    0    0    1    0   2
Ophrys-gene176455                     1    0    0    1    0   2
Ophrys-gene176494                     1    0    0    0    0   1
Ophrys-gene176495                     1    1    0    0    0   2
Ophrys-gene176527                     1    0    0    1    0   2
Ophrys-gene176528                     1    0    0    1    0   2
Ophrys-gene176997                     1    1    0    1    0   3
Ophrys-gene176983                     1    1    1    1    1   5
Ophrys-gene176984                     1    0    0    1    0   2
Ophrys-gene177235                     1    0    0    1    0   2
Ophrys-gene177236                     1    0    1    1    0   3
Ophrys-gene177684                     1    0    1    1    0   3
Ophrys-gene177691                     1    1    1    1    1   5
Ophrys-gene177695                     1    1    1    1    1   5
Ophrys-gene177696                     1    0    1    1    0   3
Ophrys-gene177678                     1    0    1    1    0   3
Ophrys-gene177679                     0    0    0    1    0   1
Ophrys-gene177733                     0    1    0    1    0   2
Ophrys-gene177734                     1    0    1    0    0   2
Ophrys-gene177787                     1    1    1    1    1   5
Ophrys-gene177788                     1    0    1    1    0   3
Ophrys-gene178061                     1    1    1    0    0   3
Ophrys-gene178062                     1    1    1    1    1   5
Ophrys-gene178160                     1    0    1    1    0   3
Ophrys-gene178222                     1    1    1    1    1   5
Ophrys-gene178475                     1    1    1    1    1   5
Ophrys-gene178514                     0    0    1    0    0   1
Ophrys-gene178510                     1    0    1    1    0   3
Ophrys-gene178609                     1    1    1    1    1   5
Ophrys-gene178610                     1    0    0    1    0   2
Ophrys-gene178599                     1    0    0    1    0   2
Ophrys-gene178656                     1    0    0    0    0   1
Ophrys-gene178665                     1    1    1    1    1   5
Ophrys-gene178669                     1    0    0    1    0   2
Ophrys-gene178645                     0    0    0    1    0   1
Ophrys-gene178701                     1    0    0    1    0   2
Ophrys-gene178714                     0    0    0    1    0   1
Ophrys-gene178745                     0    0    0    1    0   1
Ophrys-gene178744                     1    0    1    0    0   2
Ophrys-gene178780                     1    0    1    0    0   2
Ophrys-gene178814                     1    1    1    1    1   5
Ophrys-gene178815                     1    0    0    1    0   2
Ophrys-gene178979                     1    1    1    1    1   5
Ophrys-gene179816                     1    0    1    1    0   3
Ophrys-gene179915                     1    1    1    1    1   5
Ophrys-gene179916                     1    1    1    1    1   5
Ophrys-gene179925                     0    0    0    1    0   1
Ophrys-gene180053                     1    0    0    1    0   2
Ophrys-gene180065                     1    1    1    1    1   5
Ophrys-gene180066                     1    0    1    0    0   2
Ophrys-gene180184                     1    1    1    1    1   5
Ophrys-gene180203                     1    0    0    1    0   2
Ophrys-gene180204                     1    1    1    1    1   5
Ophrys-gene180213                     1    1    1    1    1   5
Ophrys-gene180325                     1    0    0    1    0   2
Ophrys-gene180333                     1    1    1    1    1   5
Ophrys-gene180370                     1    0    0    1    0   2
Ophrys-gene180371                     1    1    1    1    1   5
Ophrys-gene181542                     1    1    1    1    1   5
Ophrys-gene181551                     1    1    1    1    1   5
Ophrys-gene181720                     1    0    0    0    0   1
Ophrys-gene181721                     0    0    1    0    0   1
Ophrys-gene181728                     1    0    0    1    0   2
Ophrys-gene181734                     1    0    0    0    0   1
Ophrys-gene181744                     0    1    0    0    0   1
Ophrys-gene181806                     1    0    0    1    0   2
Ophrys-gene181807                     1    0    0    1    0   2
Ophrys-gene181814                     1    1    1    1    1   5
Ophrys-gene181820                     1    1    1    1    1   5
Ophrys-gene181822                     1    1    1    1    1   5
Ophrys-gene181823                     1    1    1    1    1   5
Ophrys-gene181830                     1    0    1    1    0   3
Ophrys-gene182416                     1    1    1    1    1   5
Ophrys-gene182675                     0    0    0    0    0   0
Ophrys-gene182676                     1    0    0    0    0   1
Ophrys-gene182686                     1    1    1    1    1   5
Ophrys-gene182687                     1    0    0    1    0   2
Ophrys-gene182660                     1    1    1    1    1   5
Ophrys-gene182723                     0    0    0    1    0   1
Ophrys-gene182734                     0    0    0    1    0   1
Ophrys-gene182735                     1    0    0    0    0   1
Ophrys-gene182707                     0    0    0    1    0   1
Ophrys-gene183081                     1    1    1    1    1   5
Ophrys-gene183093                     1    1    1    1    1   5
Ophrys-gene183139                     1    1    1    1    1   5
Ophrys-gene183378                     1    0    1    0    0   2
Ophrys-gene183409                     1    1    1    1    1   5
Ophrys-gene183490                     1    0    0    1    0   2
Ophrys-gene183494                     1    0    0    0    0   1
Ophrys-gene183700                     1    0    0    1    0   2
Ophrys-gene183711                     0    0    0    0    0   0
Ophrys-gene183871                     1    0    0    1    0   2
Ophrys-gene183874                     0    0    0    0    0   0
Ophrys-gene183908                     1    0    0    1    0   2
Ophrys-gene183901                     1    1    1    1    1   5
Ophrys-gene183902                     1    1    0    1    0   3
Ophrys-gene183905                     1    0    0    1    0   2
Ophrys-gene183969                     1    0    0    0    0   1
Ophrys-gene183991                     1    0    0    1    0   2
Ophrys-gene183994                     0    0    0    1    0   1
Ophrys-gene184027                     1    0    0    1    0   2
Ophrys-gene184020                     0    0    1    1    0   2
Ophrys-gene184021                     1    1    1    1    1   5
Ophrys-gene184024                     1    1    1    1    1   5
Ophrys-gene184086                     0    0    0    1    0   1
Ophrys-gene184107                     0    0    1    1    0   2
Ophrys-gene184151                     1    0    0    1    0   2
Ophrys-gene184190                     0    0    1    0    0   1
Ophrys-gene184368                     1    1    1    1    1   5
Ophrys-gene184845                     1    0    1    0    0   2
Ophrys-gene184885                     1    1    1    1    1   5
Ophrys-gene184919                     1    0    0    1    0   2
Ophrys-gene184952                     1    0    1    1    0   3
Ophrys-gene184985                     0    0    1    0    0   1
Ophrys-gene185016                     1    1    1    1    1   5
Ophrys-gene185047                     1    1    1    1    1   5
Ophrys-gene185077                     0    0    0    0    0   0
Ophrys-gene185134                     1    0    1    1    0   3
Ophrys-gene186387                     1    0    0    0    0   1
Ophrys-gene186368                     1    1    1    1    1   5
Ophrys-gene186369                     1    0    1    0    0   2
Ophrys-gene186876                     1    1    1    1    1   5
Ophrys-gene186974                     1    0    0    1    0   2
Ophrys-gene187064                     1    1    1    1    1   5
Ophrys-gene187130                     1    0    0    1    0   2
Ophrys-gene187508                     1    1    1    1    1   5
Ophrys-gene188307                     0    1    0    1    0   2
Ophrys-gene188628                     1    1    1    1    1   5
Ophrys-gene188629                     1    1    1    1    1   5
Ophrys-gene188695                     1    1    1    1    1   5
Ophrys-gene189233                     1    0    0    0    0   1
Ophrys-gene189235                     1    0    1    1    0   3
Ophrys-gene189906                     1    0    0    1    0   2
Ophrys-gene190237                     1    1    0    0    0   2
Ophrys-gene190404                     1    0    0    0    0   1
Ophrys-gene190719                     1    1    1    0    1   4
Ophrys-gene190703                     1    1    1    1    1   5
Ophrys-gene192917                     1    1    1    1    1   5
Ophrys-gene192918                     1    1    1    1    1   5
Ophrys-gene194052                     1    1    1    1    1   5
Ophrys-gene194970                     1    1    1    1    1   5
Ophrys-gene195025                     1    0    0    0    0   1
Ophrys-gene195027                     1    1    1    1    1   5
Ophrys-gene195076                     1    1    0    1    0   3
Ophrys-gene195130                     1    1    1    1    1   5
Ophrys-gene195209                     1    1    1    1    1   5
Ophrys-gene195210                     1    1    1    1    1   5
Ophrys-gene195212                     1    1    1    1    1   5
Ophrys-gene195910                     1    0    0    1    0   2
Ophrys-gene196114                     1    1    1    1    1   5
Ophrys-gene196229                     1    1    1    1    1   5
Ophrys-gene197014                     1    1    1    1    1   5
Ophrys-gene197368                     1    1    0    1    0   3
Ophrys-gene197369                     1    0    0    1    0   2
Ophrys-gene198676                     0    0    0    0    0   0
Ophrys-gene198684                     1    1    1    1    1   5
Ophrys-gene199098                     1    1    1    1    1   5
Ophrys-gene199102                     1    1    1    1    1   5
Ophrys-gene199427                     1    1    1    1    1   5
Ophrys-gene199431                     1    1    1    1    1   5
Ophrys-gene200238                     1    1    1    1    1   5
Ophrys-gene200557                     0    1    1    1    0   3
Ophrys-gene200579                     1    1    1    1    1   5
Ophrys-gene200580                     0    0    1    0    0   1
Ophrys-gene200920                     1    1    1    1    1   5
Ophrys-gene203629                     1    1    0    1    0   3
Ophrys-gene203660                     1    1    0    1    1   4
Ophrys-gene203661                     1    1    1    1    1   5
Ophrys-gene203695                     0    0    0    1    0   1
Ophrys-gene203713                     1    0    0    1    0   2
Ophrys-gene203744                     0    0    0    1    0   1
Ophrys-gene203684                     1    1    1    1    1   5
Ophrys-gene203685                     1    0    0    0    0   1
Ophrys-gene203693                     0    1    0    1    0   2
Ophrys-gene203796                     1    0    0    1    0   2
Ophrys-gene203827                     1    0    0    1    0   2
Ophrys-gene203768                     1    0    0    0    0   1
Ophrys-gene203776                     0    0    0    1    0   1
Ophrys-gene204081                     1    0    0    1    0   2
Ophrys-gene204362                     1    0    0    0    0   1
Ophrys-gene204382                     1    1    1    1    1   5
Ophrys-gene204383                     1    0    1    0    0   2
Ophrys-gene204384                     1    1    1    1    1   5
Ophrys-gene205146                     1    1    1    1    1   5
Ophrys-gene205387                     1    1    1    1    1   5
Ophrys-gene205685                     1    1    1    1    1   5
Ophrys-gene205696                     0    0    0    1    0   1
Ophrys-gene205705                     1    1    1    1    1   5
Ophrys-gene205896                     1    0    0    0    0   1
Ophrys-gene205936                     1    0    0    1    0   2
Ophrys-gene206014                     1    0    0    1    0   2
Ophrys-gene206049                     1    0    0    0    0   1
Ophrys-gene206085                     0    0    0    0    0   0
Ophrys-gene207950                     0    0    0    1    0   1
Ophrys-gene208021                     1    1    1    1    1   5
Ophrys-gene209720                     1    1    1    1    1   5
Ophrys-gene210359                     1    1    1    1    1   5
Ophrys-gene210363                     1    0    1    1    0   3
Ophrys-gene210368                     1    0    0    1    0   2
Ophrys-gene210460                     1    0    0    0    1   2
Ophrys-gene210495                     1    0    0    1    0   2
Ophrys-gene210496                     1    0    0    1    0   2
Ophrys-gene210668                     1    0    0    1    0   2
Ophrys-gene210761                     1    1    1    1    1   5
Ophrys-gene210762                     1    0    0    1    0   2
Ophrys-gene210802                     1    0    0    1    0   2
Ophrys-gene210782                     1    1    1    1    1   5
Ophrys-gene210788                     1    0    0    0    0   1
Ophrys-gene210846                     1    0    0    1    0   2
Ophrys-gene210849                     1    0    0    1    0   2
Ophrys-gene210851                     1    1    1    1    1   5
Ophrys-gene210888                     1    1    1    1    1   5
Ophrys-gene210890                     1    0    1    1    0   3
Ophrys-gene211091                     1    0    1    1    0   3
Ophrys-gene211137                     1    1    1    1    1   5
Ophrys-gene211138                     0    0    1    1    0   2
Ophrys-gene211184                     1    0    0    1    0   2
Ophrys-gene211208                     1    1    1    1    1   5
Ophrys-gene212078                     1    0    0    1    0   2
Ophrys-gene212088                     1    1    1    1    1   5
Ophrys-gene212089                     1    1    1    1    1   5
Ophrys-gene212091                     1    1    1    1    1   5
Ophrys-gene212092                     1    0    0    0    0   1
Ophrys-gene212093                     1    0    0    1    0   2
Ophrys-gene212094                     0    0    0    0    0   0
Ophrys-gene212132                     1    1    1    1    1   5
Ophrys-gene212135                     1    1    1    1    1   5
Ophrys-gene212136                     1    1    1    1    1   5
Ophrys-gene212193                     1    1    1    1    1   5
Ophrys-gene212194                     1    0    1    0    0   2
Ophrys-gene212237                     1    1    1    1    1   5
Ophrys-gene212480                     1    0    0    1    0   2
Ophrys-gene213018                     1    0    0    0    0   1
Ophrys-gene213028                     1    0    1    0    0   2
Ophrys-gene213029                     1    0    0    0    0   1
Ophrys-gene213086                     1    0    1    1    0   3
Ophrys-gene213096                     0    1    0    1    0   2
Ophrys-gene213097                     1    0    1    0    0   2
Ophrys-gene213166                     0    0    0    1    0   1
Ophrys-gene213440                     0    0    0    1    1   2
Ophrys-gene213432                     1    1    1    1    1   5
Ophrys-gene213731                     1    1    1    1    1   5
Ophrys-gene213716                     1    0    1    1    0   3
Ophrys-gene213717                     1    1    1    1    1   5
Ophrys-gene214091                     1    0    1    1    0   3
Ophrys-gene214165                     1    1    0    1    0   3
Ophrys-gene214607                     1    1    1    1    1   5
Ophrys-gene214617                     1    0    1    1    0   3
Ophrys-gene214649                     1    1    1    1    1   5
Ophrys-gene214650                     1    1    1    1    1   5
Ophrys-gene215336                     1    0    1    1    0   3
Ophrys-gene215337                     1    0    0    1    0   2
Ophrys-gene215347                     1    0    0    1    0   2
Ophrys-gene215392                     1    1    1    1    1   5
Ophrys-gene215360                     1    1    1    1    1   5
Ophrys-gene215602                     1    1    1    1    1   5
Ophrys-gene216004                     1    0    1    1    0   3
Ophrys-gene216064                     1    1    1    1    1   5
Ophrys-gene216762                     1    1    0    1    0   3
Ophrys-gene216806                     0    0    0    0    0   0
Ophrys-gene216953                     1    0    1    1    0   3
Ophrys-gene217598                     1    1    1    1    1   5
Ophrys-gene217621                     1    0    0    1    0   2
Ophrys-gene217593                     1    1    1    1    1   5
Ophrys-gene217594                     1    1    1    1    1   5
Ophrys-gene218110                     1    0    1    0    0   2
Ophrys-gene218281                     1    0    1    1    0   3
Ophrys-gene218271                     0    0    0    1    0   1
Ophrys-gene218272                     1    0    0    1    0   2
Ophrys-gene218728                     1    0    0    0    0   1
Ophrys-gene218723                     1    0    0    1    0   2
Ophrys-gene219173                     1    1    1    1    1   5
Ophrys-gene219174                     1    1    1    1    1   5
Ophrys-gene219241                     1    0    1    1    0   3
Ophrys-gene219406                     1    1    1    1    1   5
Ophrys-gene220385                     1    1    1    1    1   5
Ophrys-gene220651                     1    0    0    1    0   2
Ophrys-gene221392                     1    1    1    1    1   5
Ophrys-gene221403                     1    1    1    1    1   5
Ophrys-gene221405                     1    1    1    0    0   3
Ophrys-gene221406                     1    1    1    1    1   5
Ophrys-gene221469                     1    0    1    1    0   3
Ophrys-gene221480                     1    1    1    1    1   5
Ophrys-gene221481                     1    1    0    1    0   3
Ophrys-gene221482                     1    1    1    1    1   5
Ophrys-gene221483                     1    1    1    1    1   5
Ophrys-gene221542                     1    1    1    1    1   5
Ophrys-gene221574                     1    0    0    0    0   1
Ophrys-gene222057                     1    1    1    1    1   5
Ophrys-gene222058                     0    0    0    0    0   0
Ophrys-gene222103                     0    0    0    0    0   0
Ophrys-gene222104                     1    0    0    1    0   2
Ophrys-gene222210                     1    0    0    0    0   1
Ophrys-gene222204                     0    0    0    1    0   1
Ophrys-gene222538                     1    1    1    1    1   5
Ophrys-gene222544                     1    1    1    1    1   5
Ophrys-gene222520                     1    0    0    1    0   2
Ophrys-gene222583                     0    0    1    1    0   2
Ophrys-gene223299                     1    0    0    0    0   1
Ophrys-gene223300                     1    1    1    1    1   5
Ophrys-gene225531                     1    0    0    1    0   2
Ophrys-gene225592                     1    1    0    1    0   3
Ophrys-gene225905                     1    1    1    1    1   5
Ophrys-gene226447                     0    0    0    1    0   1
Ophrys-gene226448                     1    0    1    1    0   3
Ophrys-gene226751                     1    1    1    1    1   5
Ophrys-gene226775                     1    0    0    0    0   1
Ophrys-gene226844                     1    0    0    1    0   2
Ophrys-gene226845                     0    0    1    1    0   2
Ophrys-gene228315                     0    1    0    1    0   2
Ophrys-gene228698                     1    1    1    1    1   5
Ophrys-gene228699                     0    0    0    0    0   0
Ophrys-gene228846                     1    1    1    1    1   5
Ophrys-gene228848                     1    1    1    1    1   5
Ophrys-gene228849                     1    1    1    1    1   5
Ophrys-gene228897                     1    1    1    1    1   5
Ophrys-gene228899                     1    0    0    0    0   1
Ophrys-gene229522                     1    0    0    1    0   2
Ophrys-gene229523                     1    0    0    1    0   2
Ophrys-gene230469                     1    1    1    1    1   5
Ophrys-gene231083                     1    1    1    1    1   5
Ophrys-gene231101                     1    1    1    1    1   5
Ophrys-gene231102                     0    0    1    1    0   2
Ophrys-gene231488                     0    0    0    0    0   0
Ophrys-gene231537                     0    0    0    1    0   1
Ophrys-gene231580                     1    1    1    1    1   5
Ophrys-gene231629                     1    0    0    1    0   2
Ophrys-gene231634                     1    0    0    0    0   1
Ophrys-gene231654                     1    1    1    1    1   5
Ophrys-gene231655                     1    0    0    1    0   2
Ophrys-gene231660                     1    1    1    1    1   5
Ophrys-gene231689                     1    1    1    1    1   5
Ophrys-gene231690                     1    1    1    1    1   5
Ophrys-gene232154                     1    1    1    1    0   4
Ophrys-gene232155                     1    0    0    1    0   2
Ophrys-gene232493                     1    1    0    1    0   3
Ophrys-gene234728                     1    1    1    1    1   5
Ophrys-gene234733                     1    0    0    1    0   2
Ophrys-gene234704                     1    0    0    0    0   1
Ophrys-gene234705                     1    1    1    1    1   5
Ophrys-gene234789                     1    1    1    1    1   5
Ophrys-gene234790                     1    0    0    1    0   2
Ophrys-gene234794                     1    1    1    1    1   5
Ophrys-gene234795                     1    1    0    0    0   2
Ophrys-gene234796                     1    0    0    1    0   2
Ophrys-gene234896                     1    0    0    1    0   2
Ophrys-gene234897                     0    0    0    1    0   1
Ophrys-gene235259                     0    0    1    1    0   2
Ophrys-gene235260                     1    0    1    1    0   3
Ophrys-gene235307                     1    1    1    1    0   4
Ophrys-gene235612                     0    0    0    0    0   0
Ophrys-gene235633                     1    1    1    1    1   5
Ophrys-gene235647                     1    0    1    0    0   2
Ophrys-gene236067                     1    1    1    1    1   5
Ophrys-gene238095                     1    1    1    1    1   5
Ophrys-gene238094                     1    0    0    0    0   1
Ophrys-gene238503                     1    0    1    1    0   3
Ophrys-gene238505                     1    1    0    0    0   2
Ophrys-gene238535                     0    0    0    1    0   1
Ophrys-gene238536                     1    1    1    1    1   5
Ophrys-gene238550                     1    1    1    1    1   5
Ophrys-gene238860                     1    0    0    0    0   1
Ophrys-gene238861                     0    0    0    1    0   1
Ophrys-gene238862                     1    1    1    1    1   5
Ophrys-gene238863                     1    1    1    1    0   4
Ophrys-gene238903                     1    1    1    1    1   5
Ophrys-gene238838                     0    1    0    0    0   1
Ophrys-gene239200                     1    1    1    1    1   5
Ophrys-gene239555                     1    0    0    1    0   2
Ophrys-gene239559                     1    1    1    1    1   5
Ophrys-gene239602                     1    1    1    1    1   5
Ophrys-gene239608                     1    1    1    1    1   5
Ophrys-gene239610                     1    1    1    1    1   5
Ophrys-gene240122                     1    1    1    1    1   5
Ophrys-gene240128                     1    1    1    1    1   5
Ophrys-gene240142                     0    0    1    1    1   3
Ophrys-gene240143                     0    0    0    0    0   0
Ophrys-gene240342                     1    1    1    1    1   5
Ophrys-gene240664                     1    1    1    1    1   5
Ophrys-gene240652                     0    0    0    1    0   1
Ophrys-gene240753                     1    1    1    1    1   5
Ophrys-gene242732                     1    0    0    0    0   1
Ophrys-gene242733                     1    1    1    1    1   5
Ophrys-gene242986                     1    0    1    1    0   3
Ophrys-gene243842                     0    0    0    1    0   1
Ophrys-gene243827                     1    1    1    1    1   5
Ophrys-gene243832                     1    1    1    1    1   5
Ophrys-gene243835                     1    1    1    1    1   5
Ophrys-gene244538                     1    1    1    1    1   5
Ophrys-gene244547                     1    1    1    1    1   5
Ophrys-gene244518                     1    1    1    1    1   5
Ophrys-gene245754                     0    0    1    1    0   2
Ophrys-gene246642                     1    0    0    1    0   2
Ophrys-gene246643                     1    1    1    1    1   5
Ophrys-gene247349                     1    1    1    1    1   5
Ophrys-gene247350                     1    1    1    1    1   5
Ophrys-gene248809                     1    1    1    1    1   5
Ophrys-gene248810                     1    0    1    0    0   2
Ophrys-gene248812                     1    0    1    0    0   2
Ophrys-gene248802                     0    0    0    0    0   0
Ophrys-gene249138                     1    1    1    1    1   5
Ophrys-gene249114                     0    0    0    1    0   1
Ophrys-gene249453                     1    1    1    1    1   5
Ophrys-gene250069                     1    1    1    1    1   5
Ophrys-gene250097                     1    1    1    1    1   5
Ophrys-gene250098                     1    0    0    1    0   2
Ophrys-gene250125                     1    0    1    0    0   2
Ophrys-gene250152                     1    1    1    1    1   5
Ophrys-gene250616                     1    1    1    1    1   5
Ophrys-gene250662                     1    0    0    1    0   2
Ophrys-gene250704                     1    1    1    1    1   5
Ophrys-gene251630                     1    1    0    1    0   3
Ophrys-gene251866                     0    1    0    1    0   2
Ophrys-gene252095                     1    1    1    1    1   5
Ophrys-gene252080                     1    1    1    1    1   5
Ophrys-gene252090                     1    0    1    1    0   3
Ophrys-gene252091                     1    1    1    1    1   5
Ophrys-gene252459                     1    1    1    1    1   5
Ophrys-gene252461                     1    1    1    1    1   5
Ophrys-gene252465                     1    1    1    1    1   5
Ophrys-gene253007                     1    1    1    1    1   5
Ophrys-gene252997                     1    0    0    1    0   2
Ophrys-gene253322                     1    1    1    1    1   5
Ophrys-gene254107                     1    1    1    1    1   5
Ophrys-gene254186                     1    1    1    1    1   5
Ophrys-gene254217                     1    0    0    1    0   2
Ophrys-gene254218                     1    1    1    1    1   5
Ophrys-gene254757                     1    0    0    1    0   2
Ophrys-gene254831                     1    0    1    0    0   2
Ophrys-gene254834                     1    0    0    1    0   2
Ophrys-gene254847                     1    0    0    1    0   2
Ophrys-gene254920                     1    1    1    1    1   5
Ophrys-gene254937                     1    0    0    0    0   1
Ophrys-gene255011                     0    0    0    0    0   0
Ophrys-gene255024                     1    0    0    0    0   1
Ophrys-gene255099                     1    0    1    1    0   3
Ophrys-gene255100                     0    0    0    0    0   0
Ophrys-gene255854                     1    0    0    1    0   2
Ophrys-gene255847                     1    1    1    1    1   5
Ophrys-gene256423                     0    1    0    1    0   2
Ophrys-gene257618                     1    0    0    1    0   2
Ophrys-gene257620                     1    1    1    1    1   5
Ophrys-gene257621                     1    0    0    1    0   2
Ophrys-gene257641                     1    1    1    1    1   5
Ophrys-gene258300                     1    1    1    1    1   5
Ophrys-gene258301                     1    1    1    1    1   5
Ophrys-gene258312                     0    0    0    1    0   1
Ophrys-gene258317                     1    0    1    1    0   3
Ophrys-gene258331                     1    0    0    1    0   2
Ophrys-gene258364                     1    0    1    0    0   2
Ophrys-gene258375                     1    0    0    0    1   2
Ophrys-gene258379                     1    0    0    1    0   2
Ophrys-gene258381                     1    0    1    1    0   3
Ophrys-gene258387                     1    1    0    1    0   3
Ophrys-gene258393                     0    0    0    1    0   1
Ophrys-gene258394                     1    0    0    1    0   2
Ophrys-gene258425                     0    0    1    1    0   2
Ophrys-gene258436                     1    1    0    1    0   3
Ophrys-gene258440                     1    1    1    1    1   5
Ophrys-gene258442                     0    0    1    1    0   2
Ophrys-gene258448                     1    0    0    0    0   1
Ophrys-gene258454                     1    0    0    1    0   2
Ophrys-gene258455                     1    0    0    1    0   2
Ophrys-gene258484                     1    1    1    1    1   5
Ophrys-gene258493                     1    0    0    1    0   2
Ophrys-gene258500                     1    0    1    0    0   2
Ophrys-gene258502                     1    0    1    1    0   3
Ophrys-gene258515                     0    1    0    0    0   1
Ophrys-gene258542                     1    1    1    1    1   5
Ophrys-gene258543                     1    1    1    1    1   5
Ophrys-gene258552                     1    0    0    0    0   1
Ophrys-gene258553                     1    0    0    1    0   2
Ophrys-gene258559                     1    1    1    1    0   4
Ophrys-gene258573                     1    0    0    1    0   2
Ophrys-gene258599                     1    1    1    1    1   5
Ophrys-gene258608                     1    0    0    1    1   3
Ophrys-gene258615                     0    0    1    0    0   1
Ophrys-gene258629                     1    0    0    0    0   1
Ophrys-gene258598                     1    1    1    1    1   5
Ophrys-gene258663                     1    0    0    1    0   2
Ophrys-gene258667                     1    1    1    1    1   5
Ophrys-gene258681                     1    1    1    1    1   5
Ophrys-gene258714                     1    1    0    1    0   3
Ophrys-gene258718                     1    1    1    1    1   5
Ophrys-gene258732                     1    1    0    1    0   3
Ophrys-gene258733                     1    0    0    1    0   2
Ophrys-gene258703                     1    0    1    1    0   3
Ophrys-gene258761                     0    0    0    1    0   1
Ophrys-gene258768                     1    0    1    1    0   3
Ophrys-gene258770                     1    1    1    1    1   5
Ophrys-gene258782                     1    1    1    1    1   5
Ophrys-gene258752                     1    1    1    1    1   5
Ophrys-gene259014                     1    1    1    1    1   5
Ophrys-gene259024                     1    1    1    1    1   5
Ophrys-gene259334                     1    1    1    1    1   5
Ophrys-gene259345                     1    0    1    1    0   3
Ophrys-gene259393                     1    0    1    1    0   3
Ophrys-gene259394                     0    0    0    1    0   1
Ophrys-gene259382                     1    0    0    0    0   1
Ophrys-gene259440                     1    0    1    0    1   3
Ophrys-gene259441                     0    0    0    1    0   1
Ophrys-gene259487                     1    0    1    1    0   3
Ophrys-gene259476                     1    0    0    1    0   2
Ophrys-gene259533                     1    0    1    1    0   3
Ophrys-gene259578                     1    0    0    1    0   2
Ophrys-gene259579                     0    0    1    1    0   2
Ophrys-gene259666                     1    0    1    1    0   3
Ophrys-gene259709                     0    0    1    1    0   2
Ophrys-gene262551                     1    1    1    1    1   5
Ophrys-gene262663                     1    0    0    0    0   1
Ophrys-gene262827                     0    0    0    0    0   0
Ophrys-gene263185                     1    1    1    1    1   5
Ophrys-gene263288                     1    1    1    1    1   5
Ophrys-gene263432                     1    0    0    1    0   2
Ophrys-gene263433                     1    1    0    1    0   3
Ophrys-gene263417                     1    1    1    1    1   5
Ophrys-gene263479                     0    0    0    1    0   1
Ophrys-gene263692                     1    0    0    0    0   1
Ophrys-gene263693                     1    1    1    1    1   5
Ophrys-gene263862                     0    0    0    1    0   1
Ophrys-gene263874                     1    1    1    1    1   5
Ophrys-gene263846                     1    1    1    1    1   5
Ophrys-gene263903                     1    0    0    1    0   2
Ophrys-gene263898                     1    1    1    1    1   5
Ophrys-gene263902                     1    0    0    1    0   2
Ophrys-gene263929                     0    0    0    0    0   0
Ophrys-gene263930                     1    1    1    1    1   5
Ophrys-gene263965                     1    1    1    1    1   5
Ophrys-gene264034                     1    1    1    1    1   5
Ophrys-gene264051                     0    0    0    1    0   1
Ophrys-gene264100                     1    1    1    1    1   5
Ophrys-gene264152                     1    1    1    1    1   5
Ophrys-gene264196                     1    1    0    1    0   3
Ophrys-gene264790                     1    0    0    0    0   1
Ophrys-gene264786                     0    0    1    0    0   1
Ophrys-gene265052                     1    0    1    1    0   3
Ophrys-gene265308                     1    0    0    1    0   2
Ophrys-gene265337                     0    0    0    1    0   1
Ophrys-gene265338                     0    0    1    1    0   2
Ophrys-gene265341                     0    1    1    1    0   3
Ophrys-gene265371                     1    1    1    1    1   5
Ophrys-gene265620                     1    1    1    1    1   5
Ophrys-gene265662                     1    1    1    1    1   5
Ophrys-gene265647                     1    1    1    1    1   5
Ophrys-gene265762                     1    1    1    1    1   5
Ophrys-gene265726                     0    0    1    1    0   2
Ophrys-gene266108                     1    1    1    1    1   5
Ophrys-gene266116                     0    0    1    1    0   2
Ophrys-gene266291                     1    1    1    1    1   5
Ophrys-gene266344                     1    1    1    1    1   5
Ophrys-gene267550                     1    0    0    1    0   2
Ophrys-gene268053                     1    1    1    1    1   5
Ophrys-gene268054                     1    1    1    1    1   5
Ophrys-gene268057                     1    1    1    1    1   5
Ophrys-gene268037                     1    1    1    1    1   5
Ophrys-gene268115                     0    0    1    0    0   1
Ophrys-gene268089                     0    0    1    0    0   1
Ophrys-gene268389                     1    1    1    1    1   5
Ophrys-gene268394                     1    0    0    1    0   2
Ophrys-gene268413                     0    0    1    1    0   2
Ophrys-gene268414                     1    1    1    1    1   5
Ophrys-gene268506                     0    0    0    1    0   1
Ophrys-gene268604                     1    1    1    1    1   5
Ophrys-gene268605                     1    1    1    1    1   5
Ophrys-gene270096                     1    1    1    1    1   5
Ophrys-gene270098                     1    1    1    1    1   5
Ophrys-gene270112                     1    1    1    1    1   5
Ophrys-gene270114                     1    0    0    1    0   2
Ophrys-gene270116                     0    0    0    1    0   1
Ophrys-gene270080                     1    0    0    1    0   2
Ophrys-gene270082                     1    1    1    1    1   5
Ophrys-gene270493                     1    0    1    1    0   3
Ophrys-gene270492                     1    1    1    1    0   4
Ophrys-gene270621                     1    0    1    1    0   3
Ophrys-gene271076                     1    1    1    1    1   5
Ophrys-gene271119                     0    0    0    1    0   1
Ophrys-gene271126                     1    0    0    1    0   2
Ophrys-gene271129                     1    1    1    1    1   5
Ophrys-gene271585                     1    1    0    1    0   3
Ophrys-gene271625                     1    1    1    1    1   5
Ophrys-gene271626                     1    0    0    1    0   2
Ophrys-gene271608                     1    1    1    1    1   5
Ophrys-gene271672                     1    1    1    1    1   5
Ophrys-gene271717                     1    1    1    1    1   5
Ophrys-gene271724                     1    1    1    1    1   5
Ophrys-gene271882                     1    1    0    1    0   3
Ophrys-gene271885                     1    0    0    1    0   2
Ophrys-gene272023                     1    0    0    1    0   2
Ophrys-gene272046                     1    1    0    0    0   2
Ophrys-gene272175                     1    0    0    1    0   2
Ophrys-gene272336                     1    1    0    1    0   3
Ophrys-gene272365                     1    0    0    0    0   1
Ophrys-gene272393                     0    0    0    1    0   1
Ophrys-gene272423                     0    1    0    0    0   1
Ophrys-gene272424                     1    0    0    1    0   2
Ophrys-gene272450                     1    0    0    1    0   2
Ophrys-gene272479                     1    0    0    1    0   2
Ophrys-gene272506                     0    0    0    0    0   0
Ophrys-gene272638                     1    0    0    1    0   2
Ophrys-gene272645                     1    1    1    1    1   5
Ophrys-gene272646                     1    1    1    1    1   5
Ophrys-gene272647                     1    1    1    1    1   5
Ophrys-gene272823                     1    0    1    1    0   3
Ophrys-gene272864                     1    1    1    1    1   5
Ophrys-gene272866                     1    1    1    1    1   5
Ophrys-gene272868                     1    1    1    1    1   5
Ophrys-gene272956                     0    0    0    0    0   0
Ophrys-gene272980                     1    1    1    1    1   5
Ophrys-gene272981                     1    0    0    1    1   3
Ophrys-gene273038                     1    1    0    1    0   3
Ophrys-gene274367                     1    1    1    1    1   5
Ophrys-gene274336                     1    0    1    1    0   3
Ophrys-gene274522                     1    1    1    1    1   5
Ophrys-gene274523                     1    1    1    1    1   5
Ophrys-gene274534                     1    0    0    1    0   2
Ophrys-gene274509                     0    0    0    1    0   1
Ophrys-gene274861                     1    1    1    1    1   5
Ophrys-gene274858                     1    0    0    0    0   1
Ophrys-gene275023                     1    0    0    1    0   2
Ophrys-gene275024                     1    0    0    1    0   2
Ophrys-gene275026                     1    0    0    1    0   2
Ophrys-gene275649                     1    1    1    1    1   5
Ophrys-gene275635                     1    1    1    1    1   5
Ophrys-gene275731                     1    1    1    1    1   5
Ophrys-gene276444                     1    1    1    1    1   5
Ophrys-gene276448                     0    0    0    1    0   1
Ophrys-gene276484                     0    0    0    0    0   0
Ophrys-gene276488                     1    0    0    1    0   2
Ophrys-gene276528                     1    0    0    1    0   2
Ophrys-gene276566                     1    0    0    0    0   1
Ophrys-gene276603                     1    0    0    1    0   2
Ophrys-gene276634                     1    1    1    1    1   5
Ophrys-gene276639                     1    0    0    0    0   1
Ophrys-gene276670                     1    1    1    1    1   5
Ophrys-gene276674                     1    1    1    1    1   5
Ophrys-gene276675                     0    0    0    1    0   1
Ophrys-gene276705                     1    0    0    1    0   2
Ophrys-gene276706                     1    1    1    1    1   5
Ophrys-gene276710                     1    0    0    1    0   2
Ophrys-gene276745                     0    0    0    1    0   1
Ophrys-gene276774                     1    0    0    0    0   1
Ophrys-gene276779                     1    0    0    0    0   1
Ophrys-gene276812                     1    0    0    1    0   2
Ophrys-gene276813                     1    1    1    1    1   5
Ophrys-gene276846                     1    0    0    1    0   2
Ophrys-gene276879                     0    0    0    1    0   1
Ophrys-gene276910                     1    0    0    1    0   2
Ophrys-gene276995                     0    1    1    1    0   3
Ophrys-gene276996                     1    0    1    1    0   3
Ophrys-gene277032                     1    1    1    1    0   4
Ophrys-gene277033                     1    1    1    1    1   5
Ophrys-gene277349                     1    0    1    1    0   3
Ophrys-gene277387                     0    0    0    1    0   1
Ophrys-gene277483                     0    0    1    1    0   2
Ophrys-gene277546                     1    0    0    0    0   1
Ophrys-gene277598                     1    0    0    1    0   2
Ophrys-gene277650                     1    1    1    1    1   5
Ophrys-gene277702                     1    0    0    1    0   2
Ophrys-gene277703                     1    0    0    1    1   3
Ophrys-gene278039                     1    0    1    1    0   3
Ophrys-gene278050                     1    1    1    1    0   4
Ophrys-gene278293                     1    1    1    1    1   5
Ophrys-gene278300                     1    1    1    1    1   5
Ophrys-gene278302                     1    1    1    1    1   5
Ophrys-gene278303                     0    0    0    1    0   1
Ophrys-gene278353                     1    0    0    1    0   2
Ophrys-gene278355                     1    0    0    1    0   2
Ophrys-gene278356                     1    1    1    1    1   5
Ophrys-gene278486                     1    0    0    1    0   2
Ophrys-gene278521                     0    1    0    0    0   1
Ophrys-gene278570                     1    0    1    1    0   3
Ophrys-gene278945                     1    1    1    1    1   5
Ophrys-gene278949                     1    0    1    1    0   3
Ophrys-gene278960                     1    1    1    1    1   5
Ophrys-gene279225                     1    0    1    1    0   3
Ophrys-gene279295                     1    1    1    1    1   5
Ophrys-gene280258                     0    0    0    0    0   0
Ophrys-gene280576                     1    1    1    1    1   5
Ophrys-gene280631                     0    0    0    1    0   1
Ophrys-gene280693                     1    1    1    1    1   5
Ophrys-gene280694                     1    0    0    1    0   2
Ophrys-gene282209                     0    0    0    1    0   1
Ophrys-gene282221                     1    1    1    1    1   5
Ophrys-gene282258                     1    1    0    1    0   3
Ophrys-gene282270                     1    1    1    1    1   5
Ophrys-gene282664                     0    0    0    1    0   1
Ophrys-gene283705                     1    0    0    1    0   2
Ophrys-gene284330                     1    1    1    1    1   5
Ophrys-gene284341                     0    0    0    1    0   1
Ophrys-gene284346                     1    0    1    0    0   2
Ophrys-gene284313                     0    1    1    0    0   2
Ophrys-gene284402                     0    1    0    0    0   1
Ophrys-gene284403                     1    1    1    1    1   5
Ophrys-gene284413                     1    0    0    1    0   2
Ophrys-gene284418                     0    0    1    1    0   2
Ophrys-gene284385                     1    0    1    0    0   2
Ophrys-gene284473                     1    1    1    1    1   5
Ophrys-gene284484                     1    1    1    1    1   5
Ophrys-gene284489                     1    1    1    1    1   5
Ophrys-gene284456                     1    0    1    1    0   3
Ophrys-gene284542                     1    0    0    1    0   2
Ophrys-gene284543                     1    1    1    1    1   5
Ophrys-gene284553                     1    1    1    1    1   5
Ophrys-gene284558                     1    1    1    1    1   5
Ophrys-gene284525                     1    0    0    1    0   2
Ophrys-gene284528                     1    0    0    1    0   2
Ophrys-gene284610                     0    0    1    0    0   1
Ophrys-gene284611                     1    1    1    1    1   5
Ophrys-gene284621                     1    0    0    1    0   2
Ophrys-gene284622                     1    1    1    1    1   5
Ophrys-gene284593                     0    0    1    1    0   2
Ophrys-gene284594                     1    1    1    1    1   5
Ophrys-gene284689                     1    1    1    1    1   5
Ophrys-gene284692                     0    1    0    1    0   2
Ophrys-gene284693                     1    0    0    0    0   1
Ophrys-gene286516                     1    1    1    1    1   5
Ophrys-gene286538                     1    1    1    1    1   5
Ophrys-gene286541                     0    0    0    1    0   1
Ophrys-gene286713                     0    0    1    1    0   2
Ophrys-gene286727                     1    1    1    1    1   5
Ophrys-gene286781                     1    1    1    1    1   5
Ophrys-gene286787                     1    1    1    1    1   5
Ophrys-gene286793                     1    1    1    1    1   5
Ophrys-gene286807                     1    1    1    1    1   5
Ophrys-gene286849                     1    1    1    1    1   5
Ophrys-gene286871                     1    0    0    1    1   3
Ophrys-gene286872                     1    0    1    1    0   3
Ophrys-gene286875                     0    0    0    1    0   1
Ophrys-gene286876                     1    1    1    1    1   5
Ophrys-gene286882                     1    1    1    1    1   5
Ophrys-gene287026                     1    1    1    1    1   5
Ophrys-gene287000                     1    0    0    1    1   3
Ophrys-gene287270                     0    0    0    0    0   0
Ophrys-gene287271                     1    0    1    1    0   3
Ophrys-gene287698                     1    1    1    1    1   5
Ophrys-gene288266                     1    0    0    0    0   1
Ophrys-gene288481                     1    0    0    0    0   1
Ophrys-gene288537                     1    0    0    1    0   2
Ophrys-gene288728                     1    0    0    0    0   1
Ophrys-gene288729                     1    0    0    0    0   1
Ophrys-gene288722                     0    0    0    1    0   1
Ophrys-gene289108                     0    0    0    0    0   0
Ophrys-gene289109                     1    0    0    0    0   1
Ophrys-gene289113                     1    0    1    1    1   4
Ophrys-gene289491                     1    0    0    1    1   3
Ophrys-gene289496                     1    1    1    1    1   5
Ophrys-gene289509                     1    0    0    1    0   2
Ophrys-gene289510                     0    0    0    1    0   1
Ophrys-gene289516                     1    0    0    1    0   2
Ophrys-gene289559                     1    0    0    1    0   2
Ophrys-gene289565                     1    1    1    1    1   5
Ophrys-gene289566                     1    1    1    1    1   5
Ophrys-gene289540                     1    0    0    0    0   1
Ophrys-gene290304                     1    0    0    1    0   2
Ophrys-gene290305                     1    1    1    1    1   5
Ophrys-gene290388                     1    1    1    1    1   5
Ophrys-gene290440                     1    1    1    1    1   5
Ophrys-gene290435                     1    1    1    1    1   5
Ophrys-gene290634                     0    0    0    1    0   1
Ophrys-gene290635                     1    0    1    0    0   2
Ophrys-gene290672                     1    0    0    1    0   2
Ophrys-gene290727                     1    1    1    1    1   5
Ophrys-gene290728                     1    0    1    1    0   3
Ophrys-gene290862                     1    0    0    1    0   2
Ophrys-gene291006                     1    1    1    1    1   5
Ophrys-gene291010                     1    0    0    0    0   1
Ophrys-gene291449                     1    0    0    1    0   2
Ophrys-gene291891                     1    1    1    1    1   5
Ophrys-gene292001                     1    1    1    1    1   5
Ophrys-gene292002                     1    1    1    1    1   5
Ophrys-gene292982                     1    0    0    1    0   2
Ophrys-gene293035                     1    1    1    1    1   5
Ophrys-gene293157                     1    0    1    1    0   3
Ophrys-gene293132                     1    0    0    1    0   2
Ophrys-gene293212                     0    0    0    0    0   0
Ophrys-gene293213                     0    0    0    1    0   1
Ophrys-gene293955                     1    0    0    1    0   2
Ophrys-gene293956                     0    0    0    0    0   0
Ophrys-gene294124                     1    1    1    1    1   5
Ophrys-gene294173                     1    1    1    1    1   5
Ophrys-gene294215                     1    1    1    1    1   5
Ophrys-gene294452                     1    1    1    1    1   5
Ophrys-gene294469                     1    1    1    1    1   5
Ophrys-gene294521                     1    0    1    1    0   3
Ophrys-gene294534                     0    0    0    1    0   1
Ophrys-gene294538                     1    1    1    1    1   5
Ophrys-gene294583                     1    1    1    1    1   5
Ophrys-gene294584                     1    1    1    1    1   5
Ophrys-gene294596                     1    0    0    1    0   2
Ophrys-gene294597                     1    1    1    1    1   5
Ophrys-gene294600                     1    1    1    1    1   5
Ophrys-gene294601                     1    0    0    0    0   1
Ophrys-gene294954                     1    1    1    1    1   5
Ophrys-gene295192                     1    1    1    1    1   5
Ophrys-gene295199                     1    1    1    1    1   5
Ophrys-gene295201                     1    0    0    1    0   2
Ophrys-gene295214                     1    1    1    1    1   5
Ophrys-gene295221                     1    0    1    1    0   3
Ophrys-gene295234                     1    1    1    1    1   5
Ophrys-gene295235                     1    0    0    1    0   2
Ophrys-gene295256                     0    0    0    1    0   1
Ophrys-gene295266                     1    1    1    1    1   5
Ophrys-gene295267                     1    0    0    0    0   1
Ophrys-gene295371                     1    1    0    0    0   2
Ophrys-gene295393                     1    1    1    1    1   5
Ophrys-gene295403                     1    0    0    1    0   2
Ophrys-gene295404                     1    0    0    1    0   2
Ophrys-gene295483                     0    0    0    1    0   1
Ophrys-gene295465                     0    1    0    0    0   1
Ophrys-gene295541                     1    1    1    1    1   5
Ophrys-gene295522                     1    0    0    0    0   1
Ophrys-gene295530                     1    0    0    1    0   2
Ophrys-gene295531                     1    0    0    0    0   1
Ophrys-gene295532                     1    1    1    1    1   5
Ophrys-gene295533                     1    1    1    1    1   5
Ophrys-gene295565                     1    0    0    0    0   1
Ophrys-gene295572                     1    0    1    1    0   3
Ophrys-gene295599                     1    1    1    1    1   5
Ophrys-gene295600                     1    0    0    0    0   1
Ophrys-gene295606                     0    0    1    0    0   1
Ophrys-gene295679                     0    0    0    0    0   0
Ophrys-gene295797                     1    0    1    0    0   2
Ophrys-gene295780                     1    1    1    1    1   5
Ophrys-gene295781                     0    0    0    1    0   1
Ophrys-gene295926                     1    1    1    1    1   5
Ophrys-gene295939                     1    0    0    0    0   1
Ophrys-gene295982                     1    1    1    1    1   5
Ophrys-gene296023                     0    0    0    1    0   1
Ophrys-gene296118                     1    0    0    1    0   2
Ophrys-gene296119                     1    0    0    1    0   2
Ophrys-gene296416                     1    1    1    1    1   5
Ophrys-gene296419                     0    0    1    0    0   1
Ophrys-gene296627                     1    0    1    1    0   3
Ophrys-gene296629                     1    0    1    1    0   3
Ophrys-gene296653                     1    0    0    0    0   1
Ophrys-gene296690                     1    0    1    1    0   3
Ophrys-gene296685                     1    0    0    1    0   2
Ophrys-gene297054                     1    1    1    1    1   5
Ophrys-gene297152                     1    1    1    1    1   5
Ophrys-gene297193                     0    0    1    1    0   2
Ophrys-gene297231                     1    1    1    1    1   5
Ophrys-gene297434                     1    1    1    1    1   5
Ophrys-gene297480                     1    1    1    1    1   5
Ophrys-gene297773                     0    0    0    0    0   0
Ophrys-gene297774                     1    0    0    1    0   2
Ophrys-gene297848                     0    1    1    1    0   3
Ophrys-gene297859                     1    1    1    1    1   5
Ophrys-gene298043                     1    0    0    0    0   1
Ophrys-gene298048                     0    0    0    0    0   0
Ophrys-gene298049                     1    1    1    1    1   5
Ophrys-gene298777                     1    1    1    1    1   5
Ophrys-gene298735                     1    1    1    1    1   5
Ophrys-gene298892                     0    0    1    1    0   2
Ophrys-gene298893                     0    1    1    1    0   3
Ophrys-gene298973                     0    0    0    1    0   1
Ophrys-gene298974                     1    1    1    1    1   5
Ophrys-gene299742                     0    0    0    1    0   1
Ophrys-gene299725                     1    1    1    1    1   5
Ophrys-gene299839                     1    0    1    0    0   2
Ophrys-gene299946                     0    1    0    0    0   1
Ophrys-gene300263                     1    0    0    1    1   3
Ophrys-gene300252                     1    1    1    1    1   5
Ophrys-gene300293                     1    0    0    1    0   2
Ophrys-gene300513                     1    1    1    1    1   5
Ophrys-gene300492                     1    1    1    1    1   5
Ophrys-gene300553                     1    0    0    1    0   2
Ophrys-gene300554                     1    1    1    1    1   5
Ophrys-gene300682                     1    1    1    1    1   5
Ophrys-gene300683                     1    1    0    0    0   2
Ophrys-gene302150                     1    1    0    1    0   3
Ophrys-gene302240                     1    0    0    1    0   2
Ophrys-gene302376                     1    0    0    0    0   1
Ophrys-gene302377                     1    0    1    1    0   3
Ophrys-gene302477                     1    0    0    1    0   2
Ophrys-gene302478                     1    0    0    1    0   2
Ophrys-gene302509                     1    0    0    1    0   2
Ophrys-gene303758                     0    1    0    0    0   1
Ophrys-gene303759                     1    1    1    1    1   5
Ophrys-gene303772                     1    1    1    1    1   5
Ophrys-gene303795                     1    1    1    1    1   5
Ophrys-gene303828                     1    0    0    1    0   2
Ophrys-gene303925                     1    1    1    1    1   5
Ophrys-gene304055                     1    0    0    1    0   2
Ophrys-gene304063                     1    1    1    1    1   5
Ophrys-gene304045                     0    0    0    1    1   2
Ophrys-gene304136                     1    1    1    1    1   5
Ophrys-gene304111                     1    1    1    1    1   5
Ophrys-gene304223                     1    1    1    1    1   5
Ophrys-gene304272                     1    0    0    1    0   2
Ophrys-gene304287                     1    1    1    1    1   5
Ophrys-gene304323                     1    0    0    1    0   2
Ophrys-gene304405                     1    0    0    1    0   2
Ophrys-gene304412                     1    1    1    1    0   4
Ophrys-gene304387                     0    0    0    1    0   1
Ophrys-gene304559                     1    0    1    1    0   3
Ophrys-gene304680                     1    1    1    1    1   5
Ophrys-gene304681                     1    0    0    1    0   2
Ophrys-gene304829                     1    1    1    1    1   5
Ophrys-gene304877                     1    1    1    1    1   5
Ophrys-gene304869                     1    0    0    1    0   2
Ophrys-gene304870                     1    1    1    1    1   5
Ophrys-gene304875                     1    1    0    0    0   2
Ophrys-gene305020                     1    0    1    1    0   3
Ophrys-gene305028                     1    0    1    0    0   2
Ophrys-gene306006                     1    0    1    1    0   3
Ophrys-gene306007                     1    0    1    1    0   3
Ophrys-gene305951                     0    0    0    1    0   1
Ophrys-gene305952                     1    1    1    1    1   5
Ophrys-gene306065                     1    1    1    1    1   5
Ophrys-gene306134                     1    0    0    1    0   2
Ophrys-gene306248                     1    0    0    1    0   2
Ophrys-gene306692                     1    0    0    0    0   1
Ophrys-gene306760                     1    0    1    1    0   3
Ophrys-gene306765                     0    0    0    1    0   1
Ophrys-gene306827                     1    1    1    1    1   5
Ophrys-gene306832                     1    1    1    1    1   5
Ophrys-gene306834                     1    1    1    1    1   5
Ophrys-gene306842                     0    1    1    0    0   2
Ophrys-gene306894                     1    0    0    0    0   1
Ophrys-gene307205                     1    1    1    0    0   3
Ophrys-gene307211                     1    0    0    1    0   2
Ophrys-gene307420                     1    1    1    1    1   5
Ophrys-gene307421                     1    0    1    1    0   3
Ophrys-gene307473                     1    1    1    1    1   5
Ophrys-gene307474                     1    0    1    1    0   3
Ophrys-gene307488                     1    1    1    1    1   5
Ophrys-gene313006                     1    0    1    1    0   3
Ophrys-gene313007                     1    0    1    1    0   3
Ophrys-gene313032                     1    0    0    1    0   2
Ophrys-gene313033                     1    0    1    1    0   3
Ophrys-gene313056                     1    0    0    1    0   2
Ophrys-gene313764                     1    0    0    1    0   2
Ophrys-gene313908                     1    1    1    1    1   5
Ophrys-gene317217                     1    1    1    1    1   5
Ophrys-gene317335                     1    1    1    1    1   5
Ophrys-gene318872                     1    1    1    1    1   5
Ophrys-gene318873                     1    1    0    1    0   3
Ophrys-gene318875                     1    0    0    0    0   1
Ophrys-gene318863                     1    1    1    1    1   5
Ophrys-gene319010                     1    0    0    1    0   2
Ophrys-gene319006                     0    0    0    1    0   1
Ophrys-gene320747                     1    1    1    1    1   5
Ophrys-gene320927                     0    0    0    1    0   1
Ophrys-gene321681                     0    0    0    1    0   1
Ophrys-gene324643                     1    1    1    1    1   5
Ophrys-gene324664                     1    0    0    1    0   2
Ophrys-gene324681                     1    0    0    0    0   1
Ophrys-gene324685                     1    1    1    1    1   5
Ophrys-gene324693                     1    0    0    1    0   2
Ophrys-gene324714                     0    0    0    0    0   0
Ophrys-gene324720                     1    1    1    1    1   5
Ophrys-gene324657                     1    1    1    1    0   4
Ophrys-gene324658                     1    1    1    1    1   5
Ophrys-gene326491                     1    1    1    1    1   5
Ophrys-gene329154                     1    0    1    0    0   2
Ophrys-gene330879                     0    0    0    0    0   0
Ophrys-gene330883                     0    0    0    1    0   1
Ophrys-gene331082                     0    0    0    1    0   1
Ophrys-gene331109                     1    1    1    1    1   5
Ophrys-gene331134                     1    1    0    1    0   3
Ophrys-gene331571                     1    0    0    1    0   2
Ophrys-gene331919                     1    1    1    1    1   5
Ophrys-gene332729                     1    0    0    1    0   2
Ophrys-gene332721                     1    0    0    0    0   1
Ophrys-gene332761                     1    0    0    0    0   1
Ophrys-gene332770                     1    1    1    1    1   5
Ophrys-gene332800                     1    1    0    1    0   3
Ophrys-gene332835                     1    0    0    0    0   1
Ophrys-gene332836                     1    1    1    1    1   5
Ophrys-gene332844                     1    0    0    1    0   2
Ophrys-gene332845                     1    1    1    1    1   5
Ophrys-gene332870                     0    0    0    1    0   1
Ophrys-gene332878                     1    1    1    1    1   5
Ophrys-gene335152                     1    0    0    0    0   1
Ophrys-gene335160                     1    1    1    1    1   5
Ophrys-gene335714                     1    0    1    1    0   3
Ophrys-gene335794                     1    0    0    0    0   1
Ophrys-gene335796                     1    1    1    1    1   5
Ophrys-gene335799                     1    0    1    1    0   3
Ophrys-gene335809                     1    1    0    1    0   3
Ophrys-gene336253                     1    1    1    1    1   5
Ophrys-gene336254                     1    1    1    1    1   5
Ophrys-gene336278                     1    1    1    1    1   5
Ophrys-gene336341                     0    0    0    0    0   0
Ophrys-gene336342                     1    1    0    1    0   3
Ophrys-gene336401                     1    0    0    0    1   2
Ophrys-gene336402                     1    0    0    1    0   2
Ophrys-gene336661                     1    1    0    1    0   3
Ophrys-gene336662                     0    0    0    1    0   1
Ophrys-gene336704                     0    0    0    1    0   1
Ophrys-gene336705                     0    1    0    1    0   2
Ophrys-gene336744                     1    0    0    0    0   1
Ophrys-gene337425                     1    0    0    1    0   2
Ophrys-gene337431                     1    1    1    1    1   5
Ophrys-gene337432                     1    1    1    1    1   5
Ophrys-gene337460                     1    1    1    1    1   5
Ophrys-gene337463                     1    0    1    1    0   3
Ophrys-gene338182                     1    1    0    1    0   3
Ophrys-gene338184                     0    0    0    0    0   0
Ophrys-gene338294                     1    1    1    1    1   5
Ophrys-gene338295                     1    0    0    0    0   1
Ophrys-gene338286                     1    0    0    0    0   1
Ophrys-gene338353                     0    0    0    1    0   1
Ophrys-gene338382                     1    0    0    0    0   1
Ophrys-gene338386                     0    1    1    1    0   3
Ophrys-gene339114                     0    1    0    1    0   2
Ophrys-gene339118                     1    1    1    1    1   5
Ophrys-gene339409                     0    0    0    1    0   1
Ophrys-gene340852                     0    0    0    1    0   1
Ophrys-gene340880                     1    0    0    1    0   2
Ophrys-gene341058                     1    1    0    0    0   2
Ophrys-gene341081                     1    0    0    1    0   2
Ophrys-gene341108                     1    1    1    1    1   5
Ophrys-gene341109                     1    1    1    1    1   5
Ophrys-gene341159                     1    0    1    1    0   3
Ophrys-gene341194                     1    0    1    1    0   3
Ophrys-gene341181                     1    1    1    1    1   5
Ophrys-gene341182                     1    1    1    1    1   5
Ophrys-gene341187                     1    0    0    0    0   1
Ophrys-gene341499                     1    0    0    0    0   1
Ophrys-gene341500                     1    0    0    0    0   1
Ophrys-gene341507                     1    0    0    0    0   1
Ophrys-gene341966                     1    0    0    1    0   2
Ophrys-gene341967                     1    0    0    0    0   1
Ophrys-gene342046                     0    0    0    1    0   1
Ophrys-gene342455                     1    0    0    1    0   2
Ophrys-gene343292                     1    0    0    1    0   2
Ophrys-gene343320                     1    1    1    1    1   5
Ophrys-gene343323                     1    1    1    1    1   5
Ophrys-gene343341                     0    0    0    0    0   0
Ophrys-gene343342                     0    0    0    1    0   1
Ophrys-gene344501                     1    1    1    1    1   5
Ophrys-gene344511                     1    1    1    1    1   5
Ophrys-gene344519                     1    0    0    1    0   2
Ophrys-gene344527                     1    1    0    1    0   3
Ophrys-gene344536                     0    0    0    0    0   0
Ophrys-gene344487                     1    0    0    1    0   2
Ophrys-gene344496                     0    0    0    0    0   0
Ophrys-gene344497                     1    1    1    1    1   5
Ophrys-gene345735                     1    1    1    1    1   5
Ophrys-gene346393                     0    0    0    1    0   1
Ophrys-gene346408                     1    1    1    1    1   5
Ophrys-gene346410                     0    0    1    1    0   2
Ophrys-gene346436                     1    1    1    1    1   5
Ophrys-gene346437                     1    0    0    0    0   1
Ophrys-gene347930                     1    1    1    1    1   5
Ophrys-gene348255                     1    1    1    1    1   5
Ophrys-gene348907                     1    1    1    1    1   5
Ophrys-gene348910                     1    1    1    1    1   5
Ophrys-gene348970                     1    1    1    1    1   5
Ophrys-gene349069                     1    1    1    1    1   5
Ophrys-gene349070                     1    0    0    0    0   1
Ophrys-gene349565                     0    0    0    1    0   1
Ophrys-gene349570                     1    1    1    1    1   5
Ophrys-gene349558                     1    1    1    1    1   5
Ophrys-gene349744                     1    0    0    0    0   1
Ophrys-gene349947                     1    1    1    1    1   5
Ophrys-gene349950                     1    0    1    0    0   2
Ophrys-gene349951                     1    0    0    1    0   2
Ophrys-gene349946                     1    1    1    1    1   5
Ophrys-gene351521                     1    0    1    1    0   3
Ophrys-gene351657                     1    0    0    1    0   2
Ophrys-gene351797                     1    1    1    1    1   5
Ophrys-gene352178                     1    1    1    1    1   5
Ophrys-gene352182                     1    0    0    1    0   2
Ophrys-gene352330                     0    0    0    0    0   0
Ophrys-gene352331                     1    1    1    1    1   5
Ophrys-gene352334                     1    0    0    0    0   1
Ophrys-gene352339                     1    1    1    1    1   5
Ophrys-gene352340                     1    1    1    1    1   5
Ophrys-gene352565                     0    0    1    1    0   2
Ophrys-gene352566                     1    0    0    1    0   2
Ophrys-gene352580                     1    0    0    0    0   1
Ophrys-gene352797                     0    0    0    1    0   1
Ophrys-gene353319                     1    1    1    1    1   5
Ophrys-gene353336                     1    1    1    1    1   5
Ophrys-gene353398                     1    1    1    1    1   5
Ophrys-gene353399                     1    1    1    1    1   5
Ophrys-gene353461                     1    0    0    1    0   2
Ophrys-gene353522                     0    0    0    0    0   0
Ophrys-gene353948                     1    1    1    1    1   5
Ophrys-gene354972                     1    0    0    1    0   2
Ophrys-gene354973                     0    1    1    0    1   3
Ophrys-gene355046                     1    1    1    1    1   5
Ophrys-gene355118                     0    0    0    0    1   1
Ophrys-gene355601                     0    0    1    1    0   2
Ophrys-gene355778                     1    1    1    1    1   5
Ophrys-gene355779                     1    1    1    1    1   5
Ophrys-gene356178                     1    1    1    1    1   5
Ophrys-gene356188                     1    0    0    1    0   2
Ophrys-gene357590                     1    0    0    1    0   2
Ophrys-gene357609                     1    1    1    1    1   5
Ophrys-gene358380                     1    1    1    1    1   5
Ophrys-gene358418                     1    0    0    1    0   2
Ophrys-gene359806                     1    1    1    1    1   5
Ophrys-gene359807                     1    1    0    1    0   3
Ophrys-gene359782                     1    1    1    0    0   3
Ophrys-gene359845                     1    1    1    1    1   5
Ophrys-gene359846                     1    0    1    0    0   2
Ophrys-gene359885                     1    1    1    1    1   5
Ophrys-gene360168                     0    0    0    0    0   0
Ophrys-gene360169                     1    1    0    1    0   3
Ophrys-gene360620                     0    0    0    1    0   1
Ophrys-gene360621                     1    0    0    1    0   2
Ophrys-gene360830                     1    1    1    1    1   5
Ophrys-gene361367                     1    1    1    1    1   5
Ophrys-gene361410                     0    0    0    1    0   1
Ophrys-gene361865                     0    0    1    1    1   3
Ophrys-gene361955                     1    0    0    0    0   1
Ophrys-gene361956                     1    1    1    1    1   5
Ophrys-gene361960                     1    0    0    1    0   2
Ophrys-gene361927                     1    0    1    1    0   3
Ophrys-gene363345                     1    1    1    1    1   5
Ophrys-gene363359                     1    0    1    1    0   3
Ophrys-gene363361                     1    0    0    1    0   2
Ophrys-gene363362                     1    1    1    1    1   5
Ophrys-gene364176                     1    1    1    1    1   5
Ophrys-gene364177                     1    0    0    1    0   2
Ophrys-gene364233                     1    1    1    1    1   5
Ophrys-gene364258                     1    0    1    1    0   3
Ophrys-gene364507                     1    1    1    1    1   5
Ophrys-gene364761                     0    0    1    0    0   1
Ophrys-gene365037                     1    0    0    1    0   2
Ophrys-gene365038                     1    0    0    1    0   2
Ophrys-gene365143                     1    0    0    0    0   1
Ophrys-gene365145                     1    0    0    1    0   2
Ophrys-gene365196                     1    0    0    1    0   2
Ophrys-gene365198                     1    0    0    1    0   2
Ophrys-gene365183                     0    0    0    1    0   1
Ophrys-gene365352                     1    0    0    1    0   2
Ophrys-gene365354                     1    0    0    1    0   2
Ophrys-gene365571                     0    0    0    1    0   1
Ophrys-gene365572                     1    1    1    1    1   5
Ophrys-gene367632                     1    0    1    1    0   3
Ophrys-gene367633                     1    0    0    1    0   2
Ophrys-gene367681                     1    0    0    1    1   3
Ophrys-gene367990                     1    0    1    0    0   2
Ophrys-gene369837                     1    1    1    1    0   4
Ophrys-gene370045                     1    1    1    1    1   5
Ophrys-gene370046                     1    0    0    0    0   1
Ophrys-gene370142                     1    0    0    0    0   1
Ophrys-gene370897                     0    0    0    1    0   1
Ophrys-gene370898                     1    1    0    0    0   2
Ophrys-gene372273                     1    1    1    1    1   5
Ophrys-gene372274                     1    1    1    1    1   5
Ophrys-gene373538                     1    1    1    1    1   5
Ophrys-gene373795                     1    0    0    1    0   2
Ophrys-gene374342                     1    0    1    1    0   3
Ophrys-gene374340                     0    0    0    0    0   0
Ophrys-gene374341                     0    0    0    1    0   1
Ophrys-gene374920                     1    1    1    1    1   5
Ophrys-gene374921                     1    0    0    1    0   2
Ophrys-gene375006                     0    0    1    1    0   2
Ophrys-gene375013                     0    1    0    0    0   1
Ophrys-gene375016                     1    0    0    1    0   2
Ophrys-gene375020                     1    0    0    1    0   2
Ophrys-gene374992                     1    1    1    1    1   5
Ophrys-gene375061                     1    0    0    1    0   2
Ophrys-gene375068                     1    0    0    0    0   1
Ophrys-gene375075                     1    1    1    1    1   5
Ophrys-gene375110                     1    0    1    1    0   3
Ophrys-gene375117                     1    1    1    0    0   3
Ophrys-gene375120                     0    0    0    0    0   0
Ophrys-gene375159                     1    0    0    1    0   2
Ophrys-gene375166                     1    0    1    1    0   3
Ophrys-gene375173                     1    0    0    1    0   2
Ophrys-gene375212                     1    1    1    1    1   5
Ophrys-gene375215                     1    1    1    1    1   5
Ophrys-gene375205                     0    0    1    1    0   2
Ophrys-gene375206                     1    1    1    1    1   5
Ophrys-gene375739                     0    0    0    1    0   1
Ophrys-gene375816                     0    0    0    1    0   1
Ophrys-gene375829                     0    0    1    0    0   1
Ophrys-gene376676                     1    1    1    1    1   5
Ophrys-gene377169                     1    1    1    1    1   5
Ophrys-gene377614                     1    1    1    1    1   5
Ophrys-gene377852                     1    1    1    1    1   5
Ophrys-gene377854                     1    1    1    1    1   5
Ophrys-gene378074                     0    0    0    1    0   1
Ophrys-gene378085                     1    1    1    1    1   5
Ophrys-gene378105                     1    1    0    0    0   2
Ophrys-gene378109                     1    1    1    1    1   5
Ophrys-gene378110                     1    1    1    1    1   5
Ophrys-gene378158                     0    0    0    1    0   1
Ophrys-gene378265                     0    0    0    1    0   1
Ophrys-gene378266                     1    0    0    1    0   2
Ophrys-gene378274                     1    0    0    1    0   2
Ophrys-gene378275                     1    0    0    0    0   1
Ophrys-gene378942                     1    1    1    1    1   5
Ophrys-gene378972                     0    0    0    0    0   0
Ophrys-gene378937                     1    1    1    1    1   5
Ophrys-gene378938                     1    1    1    1    1   5
Ophrys-gene379151                     1    1    1    1    1   5
Ophrys-gene379152                     1    0    0    0    0   1
Ophrys-gene379313                     0    0    0    1    0   1
Ophrys-gene379314                     1    1    1    1    1   5
Ophrys-gene379515                     1    1    1    1    1   5
Ophrys-gene379518                     1    1    1    1    1   5
Ophrys-gene380145                     1    0    1    1    0   3
Ophrys-gene380146                     0    0    1    1    0   2
Ophrys-gene380366                     1    1    1    1    1   5
Ophrys-gene380679                     1    0    0    1    0   2
Ophrys-gene382143                     1    0    0    1    0   2
Ophrys-gene382145                     1    0    0    1    0   2
Ophrys-gene382223                     1    1    1    1    1   5
Ophrys-gene382225                     1    1    0    1    0   3
Ophrys-gene382308                     1    0    0    1    0   2
Ophrys-gene383105                     1    1    1    1    1   5
Ophrys-gene383159                     1    1    0    0    0   2
Ophrys-gene383209                     0    0    0    1    0   1
Ophrys-gene383351                     1    0    0    1    0   2
Ophrys-gene383357                     1    0    0    1    0   2
Ophrys-gene383402                     1    1    1    1    1   5
Ophrys-gene383405                     0    0    0    0    1   1
Ophrys-gene383944                     1    0    1    0    0   2
Ophrys-gene384415                     1    1    0    0    0   2
Ophrys-gene384535                     1    0    0    1    0   2
Ophrys-gene384526                     1    0    0    1    0   2
Ophrys-gene384602                     0    0    0    1    0   1
Ophrys-gene385069                     1    1    1    1    1   5
Ophrys-gene385429                     1    1    1    1    1   5
Ophrys-gene385476                     1    1    1    1    1   5
Ophrys-gene385569                     1    0    1    1    1   4
Ophrys-gene385614                     1    1    1    1    1   5
Ophrys-gene385783                     1    0    1    1    0   3
Ophrys-gene385874                     0    1    0    0    0   1
Ophrys-gene385877                     1    0    0    1    0   2
Ophrys-gene385883                     0    0    0    1    0   1
Ophrys-gene385889                     1    0    0    1    0   2
Ophrys-gene386155                     1    0    0    1    0   2
Ophrys-gene386137                     1    1    0    0    0   2
Ophrys-gene386354                     1    0    0    1    0   2
Ophrys-gene386363                     1    0    1    0    0   2
Ophrys-gene386364                     0    0    0    1    0   1
Ophrys-gene386366                     1    1    1    1    1   5
Ophrys-gene386400                     0    0    0    1    0   1
Ophrys-gene386650                     1    0    0    1    0   2
Ophrys-gene386707                     1    1    1    1    1   5
Ophrys-gene386762                     1    0    0    1    0   2
Ophrys-gene387154                     1    1    1    1    1   5
Ophrys-gene388302                     0    0    0    1    0   1
Ophrys-gene389366                     1    0    0    1    0   2
Ophrys-gene389883                     1    1    1    1    1   5
Ophrys-gene390538                     1    0    0    1    0   2
Ophrys-gene390544                     1    1    1    1    1   5
Ophrys-gene390650                     0    0    1    1    0   2
Ophrys-gene390950                     1    0    0    0    0   1
Ophrys-gene390938                     1    0    1    0    0   2
Ophrys-gene390939                     1    0    0    1    0   2
Ophrys-gene390999                     1    0    0    0    0   1
Ophrys-gene390987                     1    0    0    1    1   3
Ophrys-gene390990                     1    0    0    1    0   2
Ophrys-gene391043                     0    0    0    0    0   0
Ophrys-gene391032                     1    0    0    1    0   2
Ophrys-gene391086                     1    1    0    1    0   3
Ophrys-gene391074                     1    1    1    1    1   5
Ophrys-gene391075                     0    0    0    1    0   1
Ophrys-gene391078                     1    0    0    1    0   2
Ophrys-gene391767                     1    1    1    1    1   5
Ophrys-gene391768                     1    1    1    1    1   5
Ophrys-gene391761                     0    1    0    1    0   2
Ophrys-gene392568                     1    0    0    1    0   2
Ophrys-gene392576                     1    0    1    0    0   2
Ophrys-gene392637                     1    1    1    1    1   5
Ophrys-gene392638                     0    0    0    1    0   1
Ophrys-gene392647                     1    1    1    1    1   5
Ophrys-gene392655                     0    0    0    0    0   0
Ophrys-gene392928                     1    1    1    1    1   5
Ophrys-gene393012                     1    0    0    1    0   2
Ophrys-gene393153                     1    1    1    1    1   5
Ophrys-gene393492                     0    0    1    1    0   2
Ophrys-gene394144                     1    1    0    1    0   3
Ophrys-gene394134                     1    1    1    1    1   5
Ophrys-gene394136                     1    1    1    1    1   5
Ophrys-gene394140                     1    0    0    0    0   1
Ophrys-gene394389                     1    0    0    0    0   1
Ophrys-gene394390                     0    0    0    0    0   0
Ophrys-gene394512                     0    0    1    0    0   1
Ophrys-gene394839                     1    1    1    1    1   5
Ophrys-gene395174                     1    1    1    1    1   5
Ophrys-gene395635                     1    1    1    1    1   5
Ophrys-gene395756                     1    1    1    1    1   5
Ophrys-gene395812                     1    1    1    1    1   5
Ophrys-gene396065                     1    0    0    0    0   1
Ophrys-gene396535                     1    1    1    1    1   5
Ophrys-gene396668                     1    0    1    1    0   3
Ophrys-gene396675                     0    0    0    1    0   1
Ophrys-gene396864                     1    0    0    1    0   2
Ophrys-gene396929                     0    0    0    0    0   0
Ophrys-gene397283                     1    0    0    1    0   2
Ophrys-gene397284                     1    0    0    1    0   2
Ophrys-gene397347                     1    1    1    1    1   5
Ophrys-gene397312                     1    0    1    0    0   2
Ophrys-gene397313                     1    0    0    1    0   2
Ophrys-gene397411                     0    0    0    1    0   1
Ophrys-gene397377                     1    1    1    1    1   5
Ophrys-gene397380                     0    0    1    0    0   1
Ophrys-gene397457                     1    1    1    1    1   5
Ophrys-gene397498                     1    1    1    1    1   5
Ophrys-gene397842                     0    1    0    0    0   1
Ophrys-gene398690                     1    1    1    1    1   5
Ophrys-gene398915                     0    1    0    1    0   2
Ophrys-gene398961                     1    0    0    1    0   2
Ophrys-gene399315                     0    0    0    1    0   1
Ophrys-gene399321                     1    1    1    1    1   5
Ophrys-gene399322                     1    1    1    1    1   5
Ophrys-gene399339                     1    0    0    0    0   1
Ophrys-gene399429                     0    0    0    0    0   0
Ophrys-gene399446                     1    0    0    1    0   2
Ophrys-gene399465                     0    0    0    1    0   1
Ophrys-gene399724                     1    0    1    1    0   3
Ophrys-gene399958                     1    1    0    1    0   3
Ophrys-gene399969                     1    1    1    1    1   5
Ophrys-gene399971                     1    1    1    1    1   5
Ophrys-gene399949                     1    0    0    0    0   1
Ophrys-gene400005                     1    1    1    1    0   4
Ophrys-gene400017                     1    1    1    1    1   5
Ophrys-gene399987                     1    0    1    0    0   2
Ophrys-gene399988                     1    0    0    0    0   1
Ophrys-gene399996                     1    1    1    1    1   5
Ophrys-gene400272                     0    0    0    1    0   1
Ophrys-gene400252                     1    1    1    1    1   5
Ophrys-gene400557                     1    0    1    1    0   3
Ophrys-gene400558                     1    1    1    1    1   5
Ophrys-gene400542                     1    1    1    1    1   5
Ophrys-gene400546                     1    0    0    0    0   1
Ophrys-gene400547                     1    1    1    1    1   5
Ophrys-gene401536                     1    1    1    1    1   5
Ophrys-gene401537                     0    0    1    0    0   1
Ophrys-gene401638                     0    0    1    1    0   2
Ophrys-gene402485                     1    0    0    0    0   1
Ophrys-gene402517                     1    1    1    1    1   5
Ophrys-gene402530                     0    0    1    1    0   2
Ophrys-gene403135                     1    0    1    1    0   3
Ophrys-gene403137                     1    1    1    1    1   5
Ophrys-gene403139                     1    0    0    0    0   1
Ophrys-gene403144                     1    0    0    0    0   1
Ophrys-gene403184                     0    0    0    1    0   1
Ophrys-gene404333                     1    0    1    0    0   2
Ophrys-gene404712                     0    0    0    1    0   1
Ophrys-gene404894                     0    0    0    1    0   1
Ophrys-gene404978                     1    1    1    1    1   5
Ophrys-gene405019                     1    1    1    1    1   5
Ophrys-gene405798                     1    0    0    1    0   2
Ophrys-gene405799                     1    0    1    1    0   3
Ophrys-gene405833                     1    0    0    1    0   2
Ophrys-gene409016                     1    0    0    1    0   2
Ophrys-gene409513                     1    1    1    1    1   5
Ophrys-gene409514                     1    1    1    1    1   5
Ophrys-gene409492                     1    1    1    1    1   5
Ophrys-gene409560                     1    1    1    1    1   5
Ophrys-gene409830                     1    0    1    1    0   3
Ophrys-gene409835                     1    0    0    0    0   1
Ophrys-gene410568                     1    1    1    1    1   5
Ophrys-gene410652                     1    0    1    1    0   3
Ophrys-gene410771                     1    0    1    1    1   4
Ophrys-gene411532                     1    0    0    0    0   1
Ophrys-gene411533                     1    1    1    1    1   5
Ophrys-gene411548                     1    1    0    1    0   3
Ophrys-gene411549                     1    1    1    1    1   5
Ophrys-gene411595                     1    1    1    1    1   5
Ophrys-gene411579                     1    1    1    1    1   5
Ophrys-gene411580                     1    1    1    1    1   5
Ophrys-gene411920                     1    0    1    1    0   3
Ophrys-gene411936                     1    0    0    1    0   2
Ophrys-gene411937                     1    0    1    0    0   2
Ophrys-gene412147                     1    0    1    1    0   3
Ophrys-gene412151                     1    1    1    1    1   5
Ophrys-gene412240                     1    1    1    1    0   4
Ophrys-gene412245                     1    1    1    1    1   5
Ophrys-gene412859                     1    0    0    1    0   2
Ophrys-gene412941                     0    1    0    0    0   1
Ophrys-gene413228                     1    1    1    1    1   5
Ophrys-gene413433                     1    1    0    0    0   2
Ophrys-gene413434                     0    0    0    1    0   1
Ophrys-gene413450                     1    1    1    1    1   5
Ophrys-gene413909                     1    1    1    1    1   5
Ophrys-gene413921                     1    1    1    1    1   5
Ophrys-gene413902                     1    1    1    1    1   5
Ophrys-gene413906                     1    1    1    1    0   4
Ophrys-gene413946                     1    0    0    1    0   2
Ophrys-gene413963                     1    1    1    1    1   5
Ophrys-gene414641                     1    0    0    1    0   2
Ophrys-gene414693                     1    1    1    1    1   5
Ophrys-gene414674                     1    0    1    1    0   3
Ophrys-gene415326                     1    1    1    1    1   5
Ophrys-gene415327                     1    1    1    1    1   5
Ophrys-gene415526                     1    1    1    1    1   5
Ophrys-gene415530                     1    1    1    1    1   5
Ophrys-gene415544                     1    1    1    0    0   3
Ophrys-gene416873                     1    1    1    1    1   5
Ophrys-gene416886                     1    1    1    1    1   5
Ophrys-gene416893                     1    1    1    1    1   5
Ophrys-gene416899                     1    1    1    1    1   5
Ophrys-gene416910                     1    1    1    1    1   5
Ophrys-gene418450                     1    1    1    1    1   5
Ophrys-gene418451                     0    0    1    1    0   2
Ophrys-gene418452                     0    0    0    1    0   1
Ophrys-gene418453                     1    1    1    1    1   5
Ophrys-gene418454                     1    1    1    1    1   5
Ophrys-gene418469                     1    1    1    1    1   5
Ophrys-gene418470                     1    1    1    1    1   5
Ophrys-gene418716                     1    0    1    1    0   3
Ophrys-gene419059                     1    0    1    1    0   3
Ophrys-gene419060                     1    1    1    1    1   5
Ophrys-gene419061                     1    0    0    1    0   2
Ophrys-gene419140                     1    0    0    1    0   2
Ophrys-gene419128                     1    1    1    1    1   5
Ophrys-gene419136                     1    0    0    1    0   2
Ophrys-gene419254                     1    1    1    1    1   5
Ophrys-gene419748                     0    0    0    0    0   0
Ophrys-gene419749                     0    0    0    1    0   1
Ophrys-gene420304                     1    0    0    1    0   2
Ophrys-gene420356                     1    0    0    1    0   2
Ophrys-gene420423                     1    0    0    0    0   1
Ophrys-gene420424                     1    1    0    0    0   2
Ophrys-gene420489                     1    0    0    1    0   2
Ophrys-gene420490                     1    0    0    0    0   1
Ophrys-gene420521                     0    0    0    1    0   1
Ophrys-gene421032                     1    0    0    0    0   1
Ophrys-gene421040                     1    0    0    0    0   1
Ophrys-gene421041                     1    1    1    1    1   5
Ophrys-gene421044                     1    0    1    1    0   3
Ophrys-gene421156                     0    0    0    1    0   1
Ophrys-gene421164                     1    0    0    1    0   2
Ophrys-gene421168                     0    0    0    1    0   1
Ophrys-gene421461                     1    0    1    0    0   2
Ophrys-gene422265                     1    0    0    1    0   2
Ophrys-gene422266                     1    0    0    1    0   2
Ophrys-gene423270                     1    0    0    1    0   2
Ophrys-gene423280                     1    0    1    1    0   3
Ophrys-gene424052                     1    1    1    1    1   5
Ophrys-gene424050                     0    0    0    1    0   1
Ophrys-gene424994                     1    1    1    1    1   5
Ophrys-gene425001                     0    1    0    0    0   1
Ophrys-gene425005                     1    0    0    1    0   2
Ophrys-gene425082                     1    0    0    0    0   1
Ophrys-gene425088                     1    1    1    1    1   5
Ophrys-gene425094                     1    0    1    1    0   3
Ophrys-gene425098                     1    1    1    1    0   4
Ophrys-gene425175                     1    1    1    1    1   5
Ophrys-gene425181                     1    1    1    1    1   5
Ophrys-gene425187                     0    0    0    1    0   1
Ophrys-gene425191                     1    1    0    1    0   3
Ophrys-gene425272                     1    1    1    1    1   5
Ophrys-gene425282                     0    1    1    0    0   2
Ophrys-gene425575                     1    0    0    1    0   2
Ophrys-gene426374                     1    0    1    1    0   3
Ophrys-gene427281                     1    0    1    1    0   3
Ophrys-gene427286                     0    0    1    1    1   3
Ophrys-gene427617                     1    1    1    1    1   5
Ophrys-gene427768                     1    1    1    1    1   5
Ophrys-gene428947                     1    1    0    0    0   2
Ophrys-gene428949                     0    0    1    1    0   2
Ophrys-gene428993                     0    0    0    1    0   1
Ophrys-gene428996                     0    0    0    1    1   2
Ophrys-gene429033                     1    1    1    1    1   5
Ophrys-gene429052                     0    0    1    0    0   1
Ophrys-gene429055                     0    0    1    1    0   2
Ophrys-gene429183                     1    1    1    1    1   5
Ophrys-gene429377                     0    0    0    1    0   1
Ophrys-gene429439                     1    0    0    1    0   2
Ophrys-gene429522                     1    0    1    1    0   3
Ophrys-gene430217                     1    0    0    1    0   2
Ophrys-gene430314                     0    0    0    0    0   0
Ophrys-gene430679                     1    1    1    1    1   5
Ophrys-gene430681                     1    1    1    1    1   5
Ophrys-gene430682                     1    1    1    1    0   4
Ophrys-gene430668                     1    0    1    1    0   3
Ophrys-gene430967                     1    1    1    1    1   5
Ophrys-gene430970                     1    0    0    0    0   1
Ophrys-gene431187                     1    1    1    1    1   5
Ophrys-gene431188                     1    0    0    0    0   1
Ophrys-gene431761                     0    1    0    1    0   2
Ophrys-gene431783                     0    0    0    1    0   1
Ophrys-gene431785                     1    0    0    1    0   2
Ophrys-gene431811                     1    1    1    1    1   5
Ophrys-gene432221                     1    0    1    0    0   2
Ophrys-gene432606                     1    1    1    1    1   5
Ophrys-gene433110                     1    0    0    1    0   2
Ophrys-gene433165                     1    1    1    1    1   5
Ophrys-gene433167                     1    0    1    1    0   3
Ophrys-gene433168                     1    1    1    1    1   5
Ophrys-gene433141                     1    0    0    0    0   1
Ophrys-gene433142                     1    1    1    1    1   5
Ophrys-gene433746                     1    1    1    1    1   5
Ophrys-gene434504                     1    0    0    1    0   2
Ophrys-gene434941                     1    1    1    1    1   5
Ophrys-gene434970                     0    0    1    0    0   1
Ophrys-gene434974                     1    0    0    0    0   1
Ophrys-gene435138                     1    0    0    1    0   2
Ophrys-gene435309                     1    1    1    1    1   5
Ophrys-gene435312                     0    0    1    1    0   2
Ophrys-gene436007                     1    0    0    1    0   2
Ophrys-gene436058                     1    1    1    1    1   5
Ophrys-gene436072                     1    1    1    1    1   5
Ophrys-gene436110                     0    0    1    0    0   1
Ophrys-gene436111                     1    0    0    1    0   2
Ophrys-gene436161                     1    0    1    1    0   3
Ophrys-gene436262                     1    0    0    1    0   2
Ophrys-gene436311                     1    1    0    1    0   3
Ophrys-gene436355                     1    0    0    1    0   2
Ophrys-gene436399                     1    0    0    1    0   2
Ophrys-gene437202                     0    0    0    0    0   0
Ophrys-gene437204                     1    1    1    1    1   5
Ophrys-gene437483                     1    1    1    1    1   5
Ophrys-gene437484                     1    1    1    1    1   5
Ophrys-gene439260                     1    0    1    1    0   3
Ophrys-gene440179                     1    1    1    1    1   5
Ophrys-gene440280                     1    0    0    1    0   2
Ophrys-gene440569                     1    0    0    1    0   2
Ophrys-gene440941                     1    0    0    1    0   2
Ophrys-gene440935                     1    0    0    1    0   2
Ophrys-gene440972                     1    0    0    0    0   1
Ophrys-gene440966                     1    0    0    0    0   1
Ophrys-gene440996                     1    0    0    1    0   2
Ophrys-gene440997                     1    0    0    1    0   2
Ophrys-gene441029                     0    0    0    1    0   1
Ophrys-gene441030                     1    0    0    0    0   1
Ophrys-gene441024                     1    0    0    1    0   2
Ophrys-gene441048                     0    1    0    0    0   1
Ophrys-gene441054                     0    0    0    1    0   1
Ophrys-gene441131                     1    1    1    1    1   5
Ophrys-gene441132                     1    1    1    1    1   5
Ophrys-gene441384                     1    1    1    1    1   5
Ophrys-gene441816                     1    0    0    1    0   2
Ophrys-gene441858                     1    0    0    1    1   3
Ophrys-gene441896                     0    0    0    1    0   1
Ophrys-gene442548                     1    0    0    1    0   2
Ophrys-gene442718                     0    0    0    0    0   0
Ophrys-gene444122                     1    1    1    1    1   5
Ophrys-gene444565                     1    0    0    1    0   2
Ophrys-gene444572                     1    0    0    1    0   2
Ophrys-gene444548                     1    0    0    0    0   1
Ophrys-gene444726                     1    1    1    1    1   5
Ophrys-gene444733                     1    0    0    0    0   1
Ophrys-gene445037                     1    1    1    1    1   5
Ophrys-gene445044                     1    0    0    1    1   3
Ophrys-gene445087                     1    1    1    1    1   5
Ophrys-gene445282                     1    1    1    1    1   5
Ophrys-gene445289                     1    0    0    1    0   2
Ophrys-gene446078                     1    1    1    1    1   5
Ophrys-gene446082                     1    1    1    0    0   3
Ophrys-gene446242                     1    0    0    1    0   2
Ophrys-gene446314                     1    0    0    1    0   2
Ophrys-gene446317                     1    1    1    0    0   3
Ophrys-gene446329                     1    0    0    1    0   2
Ophrys-gene446330                     1    0    0    1    0   2
Ophrys-gene446333                     1    0    0    0    0   1
Ophrys-gene446241                     0    0    0    1    0   1
Ophrys-gene446637                     1    1    1    1    1   5
Ophrys-gene446615                     1    1    1    1    1   5
Ophrys-gene446616                     1    1    1    1    1   5
Ophrys-gene446670                     1    0    0    1    0   2
Ophrys-gene446671                     1    0    0    1    1   3
Ophrys-gene446721                     1    0    0    1    0   2
Ophrys-gene446722                     1    1    1    1    1   5
Ophrys-gene447260                     0    0    0    0    0   0
Ophrys-gene447272                     1    1    1    1    1   5
Ophrys-gene447336                     1    1    1    1    1   5
Ophrys-gene447345                     0    0    0    1    0   1
Ophrys-gene447563                     1    1    1    1    1   5
Ophrys-gene447564                     1    1    1    1    0   4
Ophrys-gene447551                     1    1    1    1    1   5
Ophrys-gene447557                     1    1    1    1    1   5
Ophrys-gene447635                     1    1    1    1    1   5
Ophrys-gene447641                     0    0    0    0    0   0
Ophrys-gene448039                     1    1    1    1    1   5
Ophrys-gene448045                     1    0    0    0    0   1
Ophrys-gene448055                     1    1    1    1    1   5
Ophrys-gene448132                     1    1    1    1    1   5
Ophrys-gene448133                     1    0    1    0    0   2
Ophrys-gene448155                     1    1    1    1    1   5
Ophrys-gene448156                     1    0    0    1    0   2
Ophrys-gene448176                     0    0    1    0    0   1
Ophrys-gene448715                     1    1    1    1    1   5
Ophrys-gene448728                     0    1    1    1    0   3
Ophrys-gene448797                     1    1    1    1    1   5
Ophrys-gene449139                     1    0    0    1    0   2
Ophrys-gene449132                     1    0    0    0    0   1
Ophrys-gene449423                     1    1    1    1    0   4
Ophrys-gene449427                     1    1    1    1    1   5
Ophrys-gene449428                     0    0    0    0    0   0
Ophrys-gene449802                     1    1    1    1    1   5
Ophrys-gene451808                     1    0    0    1    0   2
Ophrys-gene452242                     0    0    0    1    0   1
Ophrys-gene452338                     0    0    0    0    0   0
Ophrys-gene452624                     1    1    1    1    1   5
Ophrys-gene452799                     1    1    1    1    1   5
Ophrys-gene452857                     1    1    1    1    1   5
Ophrys-gene452975                     1    0    0    1    0   2
Ophrys-gene453578                     1    0    0    0    0   1
Ophrys-gene453579                     1    0    0    1    0   2
Ophrys-gene454243                     1    0    1    1    0   3
Ophrys-gene454244                     1    1    1    1    1   5
Ophrys-gene454329                     1    0    0    0    0   1
Ophrys-gene454330                     1    1    1    1    1   5
Ophrys-gene454333                     1    0    0    1    0   2
Ophrys-gene454337                     1    1    1    1    1   5
Ophrys-gene454338                     1    1    1    1    1   5
Ophrys-gene454564                     1    1    1    1    1   5
Ophrys-gene454565                     1    1    1    1    1   5
Ophrys-gene454570                     1    0    1    1    0   3
Ophrys-gene454548                     1    0    0    0    0   1
Ophrys-gene454998                     1    0    0    1    0   2
Ophrys-gene455364                     1    0    1    0    0   2
Ophrys-gene455391                     0    0    0    1    0   1
Ophrys-gene455398                     1    1    1    1    1   5
Ophrys-gene456414                     0    0    0    1    0   1
Ophrys-gene456687                     1    1    1    1    1   5
Ophrys-gene456711                     1    0    0    0    0   1
Ophrys-gene456716                     1    0    0    1    0   2
Ophrys-gene456724                     1    1    1    1    1   5
Ophrys-gene456677                     1    0    0    1    0   2
Ophrys-gene456678                     1    1    1    1    0   4
Ophrys-gene457265                     1    0    0    0    0   1
Ophrys-gene457556                     1    0    0    1    0   2
Ophrys-gene457558                     1    1    1    1    1   5
Ophrys-gene457559                     1    1    0    1    0   3
Ophrys-gene458058                     1    0    0    1    0   2
Ophrys-gene458059                     0    0    1    0    0   1
Ophrys-gene458062                     1    1    1    1    1   5
Ophrys-gene458077                     1    1    1    1    1   5
Ophrys-gene458120                     1    1    1    1    1   5
Ophrys-gene458137                     0    1    0    1    0   2
Ophrys-gene458138                     0    0    0    0    0   0
Ophrys-gene458189                     0    0    0    1    0   1
Ophrys-gene458231                     0    1    0    1    0   2
Ophrys-gene459612                     1    0    0    1    1   3
Ophrys-gene459613                     1    1    1    1    1   5
Ophrys-gene459576                     1    1    1    1    1   5
Ophrys-gene459822                     1    1    1    1    1   5
Ophrys-gene459823                     1    0    0    0    0   1
Ophrys-gene459855                     1    1    1    1    1   5
Ophrys-gene459856                     1    0    0    1    0   2
Ophrys-gene459887                     1    0    0    0    0   1
Ophrys-gene459888                     1    1    0    0    0   2
Ophrys-gene459944                     1    1    1    1    1   5
Ophrys-gene459945                     1    0    0    1    0   2
Ophrys-gene461306                     1    1    0    0    0   2
Ophrys-gene461874                     1    0    0    0    0   1
Ophrys-gene461889                     1    0    0    0    0   1
Ophrys-gene461899                     1    1    1    1    1   5
Ophrys-gene461900                     1    0    1    1    0   3
Ophrys-gene461872                     1    1    0    1    0   3
Ophrys-gene462291                     1    0    0    0    0   1
Ophrys-gene462544                     0    0    1    1    0   2
Ophrys-gene462546                     1    1    1    1    1   5
Ophrys-gene464734                     1    0    1    1    0   3
Ophrys-gene464735                     0    1    0    1    0   2
Ophrys-gene464756                     1    1    1    1    1   5
Ophrys-gene464788                     1    1    1    1    1   5
Ophrys-gene465302                     1    1    1    1    1   5
Ophrys-gene465379                     0    0    0    0    0   0
Ophrys-gene465380                     1    1    1    1    1   5
Ophrys-gene465381                     0    1    1    1    0   3
Ophrys-gene466316                     1    0    0    1    0   2
Ophrys-gene466317                     1    1    0    1    0   3
Ophrys-gene466320                     1    1    1    1    1   5
Ophrys-gene466315                     1    1    1    1    1   5
Ophrys-gene466371                     1    1    1    1    1   5
Ophrys-gene466372                     1    1    1    1    1   5
Ophrys-gene467202                     1    0    0    1    0   2
Ophrys-gene467270                     1    0    0    0    0   1
Ophrys-gene467388                     1    0    0    1    0   2
Ophrys-gene467392                     0    0    0    1    0   1
Ophrys-gene468024                     1    1    1    1    1   5
Ophrys-gene468094                     1    1    1    1    1   5
Ophrys-gene468095                     1    1    1    1    1   5
Ophrys-gene469277                     0    0    0    0    0   0
Ophrys-gene469318                     1    1    1    1    1   5
Ophrys-gene469612                     1    0    0    1    0   2
Ophrys-gene469736                     1    0    0    0    0   1
Ophrys-gene469737                     1    1    1    1    1   5
Ophrys-gene470171                     1    0    0    0    0   1
Ophrys-gene470172                     1    1    1    1    1   5
Ophrys-gene470179                     1    1    1    1    1   5
Ophrys-gene470332                     0    0    0    1    0   1
Ophrys-gene471349                     1    0    0    1    0   2
Ophrys-gene471350                     1    0    0    1    0   2
Ophrys-gene471416                     1    1    1    1    1   5
Ophrys-gene471420                     1    1    1    1    1   5
Ophrys-gene471421                     1    1    1    1    1   5
Ophrys-gene471426                     1    1    1    1    1   5
Ophrys-gene471427                     1    1    1    1    1   5
Ophrys-gene471747                     1    1    1    1    1   5
Ophrys-gene473123                     1    1    1    1    1   5
Ophrys-gene473124                     1    1    1    1    1   5
Ophrys-gene473125                     1    1    1    1    1   5
Ophrys-gene473581                     1    1    1    1    1   5
Ophrys-gene473588                     1    0    0    0    0   1
Ophrys-gene473630                     0    0    1    0    0   1
Ophrys-gene474610                     1    0    0    1    0   2
Ophrys-gene474711                     1    1    1    1    1   5
Ophrys-gene474712                     1    0    0    1    0   2
Ophrys-gene475291                     1    1    0    1    0   3
Ophrys-gene475729                     1    0    0    0    0   1
Ophrys-gene476693                     1    1    1    1    1   5
Ophrys-gene477139                     1    1    1    1    0   4
Ophrys-gene477484                     1    1    1    1    1   5
Ophrys-gene477665                     1    0    1    1    1   4
Ophrys-gene478193                     1    0    0    1    0   2
Ophrys-gene478194                     1    0    1    0    0   2
Ophrys-gene478196                     1    0    0    1    0   2
Ophrys-gene478197                     1    1    1    1    1   5
Ophrys-gene478179                     1    1    1    1    1   5
Ophrys-gene478954                     0    0    0    0    0   0
Ophrys-gene478955                     1    0    0    0    0   1
Ophrys-gene478964                     1    1    1    1    1   5
Ophrys-gene478998                     1    0    0    1    0   2
Ophrys-gene478999                     1    1    1    1    1   5
Ophrys-gene479007                     1    0    0    1    0   2
Ophrys-gene479008                     1    1    1    1    1   5
Ophrys-gene479101                     1    1    1    1    1   5
Ophrys-gene479102                     1    1    1    1    1   5
Ophrys-gene479110                     1    0    0    1    0   2
Ophrys-gene479124                     1    0    1    1    0   3
Ophrys-gene479125                     1    1    1    1    1   5
Ophrys-gene479096                     1    0    0    1    0   2
Ophrys-gene480120                     1    1    1    1    1   5
Ophrys-gene480108                     1    1    1    1    1   5
Ophrys-gene480109                     0    0    0    1    0   1
Ophrys-gene480797                     1    1    1    1    1   5
Ophrys-gene480804                     1    1    1    1    1   5
Ophrys-gene480782                     1    0    0    0    0   1
Ophrys-gene483164                     0    1    0    0    0   1
Ophrys-gene483197                     0    0    1    1    0   2
Ophrys-gene483198                     1    1    1    0    0   3
Ophrys-gene483199                     1    0    0    1    0   2
Ophrys-gene483202                     0    0    1    1    0   2
Ophrys-gene483203                     1    0    1    1    0   3
Ophrys-gene483204                     1    0    0    1    0   2
Ophrys-gene483265                     1    0    0    0    0   1
Ophrys-gene483266                     1    0    0    0    0   1
Ophrys-gene483269                     0    0    0    1    0   1
Ophrys-gene483270                     1    0    1    0    0   2
Ophrys-gene483271                     0    0    0    1    0   1
Ophrys-gene484008                     1    1    1    1    1   5
Ophrys-gene484010                     1    0    0    0    0   1
Ophrys-gene484468                     0    0    0    0    0   0
Ophrys-gene484469                     1    1    0    0    0   2
Ophrys-gene484497                     1    0    0    1    0   2
Ophrys-gene484572                     1    1    1    1    1   5
Ophrys-gene484573                     1    0    1    1    0   3
Ophrys-gene484550                     0    0    0    1    0   1
Ophrys-gene484554                     1    1    0    0    0   2
Ophrys-gene484615                     0    1    0    1    0   2
Ophrys-gene484616                     1    1    1    1    1   5
Ophrys-gene484950                     1    0    0    1    0   2
Ophrys-gene485150                     1    1    1    1    1   5
Ophrys-gene485286                     1    1    1    1    1   5
Ophrys-gene487070                     1    0    0    1    0   2
Ophrys-gene487120                     1    1    1    1    1   5
Ophrys-gene487202                     1    0    0    1    0   2
Ophrys-gene487269                     1    0    0    1    0   2
Ophrys-gene487338                     1    1    1    1    1   5
Ophrys-gene487966                     1    0    0    1    0   2
Ophrys-gene487973                     1    1    1    1    1   5
Ophrys-gene487974                     1    1    1    1    1   5
Ophrys-gene488613                     1    0    0    1    0   2
Ophrys-gene488605                     1    0    1    1    0   3
Ophrys-gene488877                     1    1    1    1    1   5
Ophrys-gene489506                     1    1    1    1    1   5
Ophrys-gene489575                     1    0    0    1    0   2
Ophrys-gene489912                     1    0    0    1    0   2
Ophrys-gene490049                     1    0    0    1    0   2
Ophrys-gene490050                     0    0    0    0    0   0
Ophrys-gene490081                     1    0    0    0    0   1
Ophrys-gene490138                     1    1    1    1    1   5
Ophrys-gene490139                     1    0    0    1    0   2
Ophrys-gene490152                     1    0    0    1    0   2
Ophrys-gene490162                     1    1    1    1    1   5
Ophrys-gene490164                     1    1    1    1    0   4
Ophrys-gene490170                     1    0    0    1    0   2
Ophrys-gene490480                     1    1    1    1    1   5
Ophrys-gene491273                     1    1    1    1    1   5
Ophrys-gene491448                     1    1    1    1    1   5
Ophrys-gene491435                     1    0    1    1    0   3
Ophrys-gene491506                     1    1    1    1    1   5
Ophrys-gene491777                     1    0    0    0    0   1
Ophrys-gene491841                     0    0    0    1    0   1
Ophrys-gene491842                     1    1    1    1    1   5
Ophrys-gene492778                     1    1    0    1    0   3
Ophrys-gene493052                     0    0    0    1    0   1
Ophrys-gene493075                     1    0    0    1    0   2
Ophrys-gene493055                     0    0    0    1    0   1
Ophrys-gene493068                     1    1    1    1    1   5
Ophrys-gene493104                     1    1    1    1    1   5
Ophrys-gene493111                     0    0    0    1    0   1
Ophrys-gene493324                     1    1    1    1    1   5
Ophrys-gene493517                     1    1    1    1    1   5
Ophrys-gene493523                     1    1    1    1    1   5
Ophrys-gene493524                     1    1    1    1    1   5
Ophrys-gene494368                     0    0    0    0    0   0
Ophrys-gene494369                     1    0    0    1    0   2
Ophrys-gene494694                     1    1    1    1    1   5
Ophrys-gene494696                     1    1    1    1    1   5
Ophrys-gene495050                     1    1    1    1    1   5
Ophrys-gene495031                     0    0    1    0    0   1
Ophrys-gene495032                     0    0    0    1    0   1
Ophrys-gene495033                     1    0    0    1    0   2
Ophrys-gene495114                     1    1    1    1    1   5
Ophrys-gene495182                     1    1    0    0    0   2
Ophrys-gene495299                     1    0    0    0    0   1
Ophrys-gene495364                     0    0    0    1    0   1
Ophrys-gene495373                     1    1    0    0    0   2
Ophrys-gene495412                     1    0    0    1    0   2
Ophrys-gene495562                     1    1    1    1    1   5
Ophrys-gene495608                     1    0    0    1    0   2
Ophrys-gene495673                     1    1    1    1    1   5
Ophrys-gene495727                     1    1    1    1    1   5
Ophrys-gene495834                     1    0    0    0    0   1
Ophrys-gene495836                     1    1    1    1    1   5
Ophrys-gene496055                     0    0    0    0    0   0
Ophrys-gene496204                     1    1    1    1    1   5
Ophrys-gene496339                     0    1    0    0    0   1
Ophrys-gene496394                     1    1    1    1    1   5
Ophrys-gene496509                     1    0    0    1    0   2
Ophrys-gene496554                     0    0    0    1    0   1
Ophrys-gene496732                     1    1    0    0    0   2
Ophrys-gene496943                     1    1    1    1    1   5
Ophrys-gene497661                     1    1    1    1    1   5
Ophrys-gene498170                     1    1    1    1    1   5
Ophrys-gene499058                     1    1    1    1    1   5
Ophrys-gene499066                     1    0    0    1    0   2
Ophrys-gene499116                     1    1    1    1    1   5
Ophrys-gene499117                     1    1    1    1    1   5
Ophrys-gene499360                     1    1    1    1    1   5
Ophrys-gene499341                     0    0    0    1    0   1
Ophrys-gene499342                     1    0    1    0    0   2
Ophrys-gene499788                     1    0    0    1    0   2
Ophrys-gene503133                     1    0    0    1    0   2
Ophrys-gene503134                     1    1    1    1    1   5
Ophrys-gene503206                     1    1    0    1    0   3
Ophrys-gene503207                     1    0    0    1    0   2
Ophrys-gene503527                     1    1    1    1    1   5
Ophrys-gene503705                     1    1    1    1    1   5
Ophrys-gene504472                     1    1    1    1    1   5
Ophrys-gene504679                     1    0    0    0    0   1
Ophrys-gene505099                     1    0    1    1    0   3
Ophrys-gene505081                     1    0    0    1    0   2
Ophrys-gene505082                     0    0    0    1    0   1
Ophrys-gene505288                     1    1    1    1    1   5
Ophrys-gene506604                     1    0    0    1    0   2
Ophrys-gene506605                     1    0    1    0    0   2
Ophrys-gene506612                     1    1    1    1    1   5
Ophrys-gene507980                     0    0    0    1    0   1
Ophrys-gene507984                     1    1    1    1    1   5
Ophrys-gene507985                     1    1    1    1    1   5
Ophrys-gene508460                     1    1    1    1    1   5
Ophrys-gene508462                     1    1    1    1    1   5
Ophrys-gene508484                     0    0    0    1    0   1
Ophrys-gene508489                     1    1    1    1    1   5
Ophrys-gene508490                     1    1    1    1    1   5
Ophrys-gene508473                     1    1    1    1    1   5
Ophrys-gene508477                     1    0    0    0    0   1
Ophrys-gene508801                     0    0    1    0    0   1
Ophrys-gene508804                     1    0    1    0    0   2
Ophrys-gene508809                     1    0    0    1    0   2
Ophrys-gene509057                     1    0    0    1    0   2
Ophrys-gene509043                     1    1    1    1    1   5
Ophrys-gene509222                     1    0    0    1    0   2
Ophrys-gene509212                     1    0    1    0    0   2
Ophrys-gene509346                     1    1    1    1    1   5
Ophrys-gene509336                     0    0    0    0    0   0
Ophrys-gene509378                     1    1    1    1    1   5
Ophrys-gene509410                     1    0    1    1    0   3
Ophrys-gene509411                     1    1    1    1    0   4
Ophrys-gene510325                     1    0    0    1    0   2
Ophrys-gene510326                     1    1    1    1    1   5
Ophrys-gene510614                     1    1    1    0    0   3
Ophrys-gene510600                     1    0    0    1    0   2
Ophrys-gene511202                     1    1    1    1    1   5
Ophrys-gene511739                     1    0    1    1    0   3
Ophrys-gene512121                     1    1    1    1    1   5
Ophrys-gene512122                     1    0    0    0    0   1
Ophrys-gene512333                     1    1    1    1    1   5
Ophrys-gene513856                     1    1    1    1    1   5
Ophrys-gene513876                     1    1    1    1    1   5
Ophrys-gene513993                     1    1    1    1    1   5
Ophrys-gene514161                     1    1    1    1    1   5
Ophrys-gene514623                     1    1    1    1    1   5
Ophrys-gene514736                     0    0    0    0    0   0
Ophrys-gene514782                     0    0    0    1    0   1
Ophrys-gene514825                     1    0    0    1    0   2
Ophrys-gene515107                     1    0    0    1    0   2
Ophrys-gene515144                     1    0    0    0    0   1
Ophrys-gene518248                     0    0    0    1    0   1
Ophrys-gene518226                     1    1    0    1    0   3
Ophrys-gene518227                     0    0    1    1    0   2
Ophrys-gene518232                     0    0    1    1    0   2
Ophrys-gene518271                     1    0    0    0    0   1
Ophrys-gene518817                     1    1    1    1    1   5
Ophrys-gene518953                     1    1    1    1    1   5
Ophrys-gene519097                     1    0    0    0    0   1
Ophrys-gene519089                     1    1    1    1    1   5
Ophrys-gene521847                     1    1    1    1    1   5
Ophrys-gene521879                     0    0    1    1    0   2
Ophrys-gene523100                     1    1    1    1    1   5
Ophrys-gene523083                     1    1    0    0    0   2
Ophrys-gene523086                     0    1    1    1    0   3
Ophrys-gene523171                     1    1    1    1    1   5
Ophrys-gene523194                     1    1    1    1    1   5
Ophrys-gene525628                     1    1    1    1    1   5
Ophrys-gene525641                     1    1    1    1    1   5
Ophrys-gene525642                     1    0    0    1    0   2
Ophrys-gene525659                     1    1    1    1    1   5
Ophrys-gene525660                     0    0    1    1    0   2
Ophrys-gene525663                     1    0    0    1    0   2
Ophrys-gene525665                     1    0    0    1    0   2
Ophrys-gene525666                     1    1    1    1    1   5
Ophrys-gene525743                     1    1    1    1    1   5
Ophrys-gene525744                     1    0    1    0    0   2
Ophrys-gene525757                     1    0    1    0    0   2
Ophrys-gene525775                     1    1    1    1    1   5
Ophrys-gene525803                     1    1    1    1    1   5
Ophrys-gene525806                     1    0    0    0    0   1
Ophrys-gene525810                     1    0    0    1    0   2
Ophrys-gene525736                     0    0    0    1    0   1
Ophrys-gene525737                     1    1    1    1    1   5
Ophrys-gene525905                     1    1    1    1    1   5
Ophrys-gene525906                     1    0    0    0    0   1
Ophrys-gene525908                     1    1    1    1    1   5
Ophrys-gene525912                     1    1    1    1    1   5
Ophrys-gene525913                     1    1    1    1    1   5
Ophrys-gene525979                     1    1    1    1    1   5
Ophrys-gene526002                     0    1    0    1    0   2
Ophrys-gene526008                     1    0    1    0    0   2
Ophrys-gene526022                     0    0    1    1    0   2
Ophrys-gene526208                     1    1    0    1    0   3
Ophrys-gene526211                     1    1    1    1    1   5
Ophrys-gene526215                     1    0    0    0    0   1
Ophrys-gene526230                     1    0    0    1    0   2
Ophrys-gene526280                     1    1    1    1    1   5
Ophrys-gene526301                     1    1    0    0    0   2
Ophrys-gene526304                     1    0    0    1    0   2
Ophrys-gene526307                     1    1    1    1    1   5
Ophrys-gene527004                     1    0    1    1    0   3
Ophrys-gene527019                     1    0    0    0    0   1
Ophrys-gene527708                     0    1    1    0    0   2
Ophrys-gene527701                     1    0    1    1    0   3
Ophrys-gene527704                     1    0    0    1    0   2
Ophrys-gene527705                     1    1    1    1    1   5
Ophrys-gene528014                     1    1    1    1    1   5
Ophrys-gene528197                     1    1    1    1    1   5
Ophrys-gene528198                     1    1    1    1    1   5
Ophrys-gene528358                     1    0    0    1    0   2
Ophrys-gene528742                     1    1    1    1    1   5
Ophrys-gene528746                     1    1    1    1    1   5
Ophrys-gene528729                     1    1    1    1    1   5
Ophrys-gene528788                     1    0    0    1    0   2
Ophrys-gene528789                     1    0    0    1    0   2
Ophrys-gene528782                     1    0    0    1    0   2
Ophrys-gene530200                     1    0    0    1    0   2
Ophrys-gene530544                     1    1    1    1    1   5
Ophrys-gene530593                     0    0    1    1    0   2
Ophrys-gene530992                     1    0    0    1    0   2
Ophrys-gene531538                     1    1    1    1    1   5
Ophrys-gene531539                     1    1    1    1    1   5
Ophrys-gene531857                     1    0    0    1    0   2
Ophrys-gene531856                     0    0    1    1    0   2
Ophrys-gene531915                     1    0    1    1    0   3
Ophrys-gene531937                     1    1    1    1    1   5
Ophrys-gene531978                     1    1    0    1    0   3
Ophrys-gene532067                     1    1    1    1    1   5
Ophrys-gene532177                     1    1    1    1    1   5
Ophrys-gene532851                     1    0    0    0    0   1
Ophrys-gene532862                     1    1    1    1    1   5
Ophrys-gene532866                     1    1    1    1    1   5
Ophrys-gene532867                     0    0    0    1    0   1
Ophrys-gene532872                     1    1    1    1    1   5
Ophrys-gene534666                     1    1    1    1    1   5
Ophrys-gene535249                     0    0    0    1    0   1
Ophrys-gene535439                     1    1    1    1    1   5
Ophrys-gene536110                     1    1    1    1    1   5
Ophrys-gene536139                     1    1    1    1    1   5
Ophrys-gene536129                     1    1    1    1    1   5
Ophrys-gene536136                     1    1    1    1    1   5
Ophrys-gene536138                     1    0    0    1    0   2
Ophrys-gene536165                     1    1    1    1    1   5
Ophrys-gene536291                     1    0    0    1    0   2
Ophrys-gene536292                     1    0    0    0    0   1
Ophrys-gene536328                     1    0    0    1    0   2
Ophrys-gene536329                     1    1    0    0    0   2
Ophrys-gene536740                     1    1    1    1    1   5
Ophrys-gene536733                     1    1    1    1    1   5
Ophrys-gene536790                     1    0    0    1    0   2
Ophrys-gene536791                     1    1    1    1    1   5
Ophrys-gene536777                     1    0    0    0    0   1
Ophrys-gene536840                     0    0    0    0    0   0
Ophrys-gene536841                     1    1    1    1    1   5
Ophrys-gene536890                     1    1    1    1    1   5
Ophrys-gene536883                     0    0    0    1    0   1
Ophrys-gene537472                     1    1    0    1    0   3
Ophrys-gene537532                     1    1    1    1    1   5
Ophrys-gene537534                     1    0    0    1    0   2
Ophrys-gene537535                     1    1    1    1    1   5
Ophrys-gene537561                     1    0    0    1    0   2
Ophrys-gene538309                     0    0    0    1    0   1
Ophrys-gene538576                     0    0    0    1    0   1
Ophrys-gene538577                     1    0    1    1    0   3
Ophrys-gene538612                     1    0    1    1    0   3
Ophrys-gene538645                     1    0    1    1    0   3
Ophrys-gene538676                     1    0    1    1    0   3
Ophrys-gene538840                     0    0    0    1    0   1
Ophrys-gene539299                     1    0    0    0    0   1
Ophrys-gene539324                     1    1    0    1    0   3
Ophrys-gene539325                     0    0    0    0    0   0
Ophrys-gene539627                     1    0    0    1    0   2
Ophrys-gene539628                     1    0    0    1    0   2
Ophrys-gene541035                     1    0    0    1    0   2
Ophrys-gene541080                     1    0    0    1    0   2
Ophrys-gene541203                     1    0    0    1    0   2
Ophrys-gene541195                     1    1    1    1    1   5
Ophrys-gene541231                     1    0    0    1    1   3
Ophrys-gene541301                     0    0    1    0    0   1
Ophrys-gene541336                     1    1    1    1    1   5
Ophrys-gene541337                     1    0    1    0    0   2
Ophrys-gene541619                     1    1    1    1    1   5
Ophrys-gene541831                     1    1    1    1    1   5
Ophrys-gene541832                     1    0    1    0    0   2
Ophrys-gene541920                     1    1    1    1    1   5
Ophrys-gene543932                     1    1    1    1    1   5
Ophrys-gene543933                     0    0    1    0    1   2
Ophrys-gene543941                     1    1    1    1    1   5
Ophrys-gene543946                     1    0    0    1    1   3
Ophrys-gene543947                     0    0    0    1    0   1
Ophrys-gene543956                     0    0    0    0    0   0
Ophrys-gene543968                     1    0    1    1    0   3
Ophrys-gene543970                     1    0    0    1    0   2
Ophrys-gene543990                     1    1    1    1    1   5
Ophrys-gene543929                     0    0    0    0    0   0
Ophrys-gene544269                     1    1    1    1    1   5
Ophrys-gene544326                     1    1    1    1    1   5
Ophrys-gene544566                     1    0    1    1    0   3
Ophrys-gene545801                     1    1    1    1    1   5
Ophrys-gene545802                     1    0    0    0    0   1
Ophrys-gene546704                     0    0    0    1    0   1
Ophrys-gene546710                     1    1    1    1    1   5
Ophrys-gene546756                     1    1    1    1    1   5
Ophrys-gene546757                     1    1    1    1    1   5
Ophrys-gene546789                     1    0    0    0    0   1
Ophrys-gene546845                     1    0    0    1    0   2
Ophrys-gene546897                     1    0    0    1    0   2
Ophrys-gene546898                     1    0    0    1    0   2
Ophrys-gene547545                     1    0    0    0    0   1
Ophrys-gene547618                     1    0    0    1    0   2
Ophrys-gene547677                     0    0    0    0    0   0
Ophrys-gene547740                     1    0    0    1    0   2
Ophrys-gene547746                     1    1    1    1    1   5
Ophrys-gene547815                     1    1    1    1    1   5
Ophrys-gene547872                     1    1    1    1    1   5
Ophrys-gene548958                     1    1    1    1    1   5
Ophrys-gene548947                     1    0    1    1    0   3
Ophrys-gene548993                     1    0    0    1    0   2
Ophrys-gene548997                     1    0    0    1    0   2
Ophrys-gene549039                     1    1    1    1    1   5
Ophrys-gene549181                     1    0    1    1    0   3
Ophrys-gene549542                     1    0    0    1    0   2
Ophrys-gene549813                     1    0    0    0    0   1
Ophrys-gene549851                     1    1    1    1    1   5
Ophrys-gene551664                     1    1    1    1    1   5
Ophrys-gene552110                     0    0    0    0    0   0
Ophrys-gene552111                     1    1    1    1    1   5
Ophrys-gene552134                     1    0    0    1    0   2
Ophrys-gene552135                     1    1    1    1    1   5
Ophrys-gene552142                     1    0    1    1    0   3
Ophrys-gene552278                     1    1    1    1    1   5
Ophrys-gene552279                     1    1    1    1    1   5
Ophrys-gene553956                     0    0    1    1    0   2
Ophrys-gene554501                     1    0    0    1    0   2
Ophrys-gene554824                     1    0    1    1    0   3
Ophrys-gene554812                     1    1    1    1    1   5
Ophrys-gene554820                     1    0    0    1    0   2
Ophrys-gene555135                     1    0    0    1    1   3
Ophrys-gene555162                     1    1    1    1    1   5
Ophrys-gene555938                     1    0    1    1    1   4
Ophrys-gene555922                     1    0    1    1    0   3
Ophrys-gene555927                     1    0    0    1    0   2
Ophrys-gene556125                     0    0    0    1    0   1
Ophrys-gene556436                     1    1    0    0    0   2
Ophrys-gene556464                     0    0    1    0    1   2
Ophrys-gene556968                     1    0    1    1    1   4
Ophrys-gene558101                     1    1    1    1    1   5
Ophrys-gene559759                     0    1    0    0    0   1
Ophrys-gene560304                     1    0    1    1    0   3
Ophrys-gene562785                     1    1    1    1    1   5
Ophrys-gene562892                     0    0    0    0    0   0
Ophrys-gene562969                     1    1    1    1    1   5
Ophrys-gene563047                     0    0    0    1    0   1
Ophrys-gene563049                     1    1    1    1    1   5
Ophrys-gene563050                     1    1    1    1    1   5
Ophrys-gene564449                     1    1    1    1    1   5
Ophrys-gene564554                     1    0    0    1    0   2
Ophrys-gene564555                     1    1    1    1    1   5
Ophrys-gene564556                     0    0    1    1    0   2
Ophrys-gene564906                     1    1    1    1    1   5
Ophrys-gene564917                     1    0    0    0    1   2
Ophrys-gene565479                     1    1    1    1    1   5
Ophrys-gene565947                     1    0    0    1    0   2
Ophrys-gene565976                     1    1    0    1    0   3
Ophrys-gene566207                     1    1    1    1    1   5
Ophrys-gene566365                     0    0    0    1    0   1
Ophrys-gene566366                     1    1    1    1    1   5
Ophrys-gene566371                     1    0    1    1    0   3
Ophrys-gene566378                     0    0    0    0    0   0
Ophrys-gene566429                     0    0    0    1    0   1
Ophrys-gene566460                     1    1    0    1    0   3
Ophrys-gene566465                     1    1    1    1    1   5
Ophrys-gene566473                     1    1    1    1    1   5
Ophrys-gene566944                     1    1    1    1    1   5
Ophrys-gene567290                     0    0    1    1    0   2
Ophrys-gene567342                     1    0    1    0    0   2
Ophrys-gene567574                     1    0    0    0    0   1
Ophrys-gene567616                     1    1    1    1    1   5
Ophrys-gene567695                     0    0    0    1    0   1
Ophrys-gene567977                     1    0    0    1    0   2
Ophrys-gene568046                     1    0    1    1    0   3
Ophrys-gene568714                     1    1    1    1    1   5
Ophrys-gene568715                     0    0    0    1    0   1
Ophrys-gene568779                     1    0    0    0    0   1
Ophrys-gene569478                     1    1    1    1    1   5
Ophrys-gene569479                     1    0    0    1    0   2
Ophrys-gene570047                     1    1    1    1    1   5
Ophrys-gene570062                     1    1    1    1    1   5
Ophrys-gene570929                     1    0    0    1    0   2
Ophrys-gene570992                     1    1    1    1    1   5
Ophrys-gene570988                     1    0    1    1    0   3
Ophrys-gene570989                     1    1    1    1    1   5
Ophrys-gene571322                     1    1    1    1    1   5
Ophrys-gene571409                     1    0    0    0    0   1
Ophrys-gene571410                     1    1    1    1    1   5
Ophrys-gene571432                     1    0    1    0    0   2
Ophrys-gene571440                     1    1    1    1    1   5
Ophrys-gene571451                     1    1    1    1    1   5
Ophrys-gene571460                     1    1    1    1    1   5
Ophrys-gene574054                     1    1    1    1    1   5
Ophrys-gene574035                     1    1    1    1    1   5
Ophrys-gene574044                     1    1    1    1    1   5
Ophrys-gene574626                     1    0    0    1    0   2
Ophrys-gene577064                     0    0    0    1    1   2
Ophrys-gene577670                     1    0    0    1    0   2
Ophrys-gene577852                     1    1    1    1    1   5
Ophrys-gene578288                     1    0    1    1    0   3
Ophrys-gene578280                     1    1    1    1    1   5
Ophrys-gene578282                     1    1    1    1    1   5
Ophrys-gene579272                     1    0    0    1    0   2
Ophrys-gene579292                     1    1    1    1    1   5
Ophrys-gene579293                     1    0    1    1    0   3
Ophrys-gene579498                     1    1    1    1    1   5
Ophrys-gene579501                     1    1    1    1    1   5
Ophrys-gene579616                     0    0    0    0    0   0
Ophrys-gene579778                     0    0    0    1    0   1
Ophrys-gene579779                     1    1    1    1    1   5
Ophrys-gene579924                     1    1    1    1    1   5
Ophrys-gene579862                     1    1    1    0    0   3
Ophrys-gene579945                     1    1    1    1    1   5
Ophrys-gene580006                     1    0    1    0    0   2
Ophrys-gene580007                     1    1    1    1    1   5
Ophrys-gene581700                     1    1    1    1    1   5
Ophrys-gene581752                     1    0    0    0    0   1
Ophrys-gene582197                     1    0    0    0    0   1
Ophrys-gene582226                     0    0    1    1    0   2
Ophrys-gene582227                     1    0    1    0    0   2
Ophrys-gene582797                     0    0    0    0    0   0
Ophrys-gene582798                     1    0    1    1    0   3
Ophrys-gene583213                     1    1    1    0    0   3
Ophrys-gene583215                     1    1    1    1    1   5
Ophrys-gene584002                     0    0    0    1    0   1
Ophrys-gene585657                     1    0    1    1    0   3
Ophrys-gene585638                     1    1    1    1    1   5
Ophrys-gene585790                     1    1    1    1    1   5
Ophrys-gene586421                     1    0    0    1    0   2
Ophrys-gene586422                     1    1    1    1    1   5
Ophrys-gene586448                     0    0    0    1    0   1
Ophrys-gene586449                     1    0    0    1    0   2
Ophrys-gene586445                     1    0    0    1    0   2
Ophrys-gene586767                     1    1    1    1    1   5
Ophrys-gene586831                     1    1    0    1    0   3
Ophrys-gene586970                     1    0    0    1    0   2
Ophrys-gene587025                     1    1    1    1    1   5
Ophrys-gene587026                     1    0    1    1    0   3
Ophrys-gene587119                     1    1    1    1    1   5
Ophrys-gene587278                     1    1    1    1    1   5
Ophrys-gene587299                     0    1    1    0    0   2
Ophrys-gene587304                     1    0    1    1    0   3
Ophrys-gene587316                     0    0    0    1    0   1
Ophrys-gene588676                     1    1    1    1    1   5
Ophrys-gene588785                     1    1    1    1    1   5
Ophrys-gene588814                     1    1    1    1    1   5
Ophrys-gene588880                     1    1    1    1    1   5
Ophrys-gene588881                     1    1    1    1    1   5
Ophrys-gene588914                     0    0    0    1    0   1
Ophrys-gene588950                     1    1    1    1    1   5
Ophrys-gene589805                     1    1    0    1    0   3
Ophrys-gene590123                     0    0    0    1    0   1
Ophrys-gene590479                     1    0    0    0    0   1
Ophrys-gene590715                     1    0    1    1    0   3
Ophrys-gene591088                     1    1    1    1    0   4
Ophrys-gene591396                     0    0    0    1    0   1
Ophrys-gene591636                     1    0    0    0    0   1
Ophrys-gene592067                     1    1    1    1    1   5
Ophrys-gene592685                     1    0    0    1    0   2
Ophrys-gene594808                     1    0    1    0    1   3
Ophrys-gene594809                     1    1    1    1    1   5
Ophrys-gene594983                     1    0    0    1    1   3
Ophrys-gene595294                     1    1    1    1    1   5
Ophrys-gene595298                     1    1    1    1    1   5
Ophrys-gene595278                     1    1    1    1    1   5
Ophrys-gene595279                     1    0    0    1    0   2
Ophrys-gene596025                     0    0    0    1    0   1
Ophrys-gene597443                     1    0    0    0    0   1
Ophrys-gene597496                     1    0    0    1    0   2
Ophrys-gene597524                     1    1    1    1    1   5
Ophrys-gene597561                     1    1    1    1    1   5
Ophrys-gene597614                     1    0    0    1    0   2
Ophrys-gene597641                     1    1    0    0    0   2
Ophrys-gene597737                     1    1    1    1    1   5
Ophrys-gene597747                     1    1    1    1    1   5
Ophrys-gene597812                     1    0    0    1    0   2
Ophrys-gene597875                     1    1    1    1    1   5
Ophrys-gene597885                     1    1    1    1    1   5
Ophrys-gene597862                     1    1    1    1    1   5
Ophrys-gene597936                     1    0    0    1    0   2
Ophrys-gene598179                     1    0    0    1    0   2
Ophrys-gene598203                     0    1    0    1    0   2
Ophrys-gene598838                     0    0    0    1    0   1
Ophrys-gene598839                     1    1    1    1    1   5
Ophrys-gene598817                     1    1    1    1    1   5
Ophrys-gene598821                     1    1    1    1    1   5
Ophrys-gene599150                     1    0    0    0    0   1
Ophrys-gene599151                     0    0    1    1    0   2
Ophrys-gene599506                     1    0    0    1    0   2
Ophrys-gene600871                     1    0    1    1    0   3
Ophrys-gene600872                     1    1    1    1    1   5
Ophrys-gene601140                     0    0    0    1    0   1
Ophrys-gene601281                     1    0    0    1    0   2
Ophrys-gene601273                     1    0    0    0    0   1
Ophrys-gene602948                     1    1    1    1    1   5
Ophrys-gene602930                     1    0    0    1    0   2
Ophrys-gene602931                     1    1    1    1    1   5
Ophrys-gene603321                     0    0    1    1    0   2
Ophrys-gene603341                     0    0    0    1    0   1
Ophrys-gene603375                     1    1    1    1    1   5
Ophrys-gene603376                     1    1    1    1    1   5
Ophrys-gene603310                     0    1    1    1    0   3
Ophrys-gene603320                     1    1    1    1    1   5
Ophrys-gene604843                     1    1    0    1    0   3
Ophrys-gene604838                     1    0    0    1    0   2
Ophrys-gene604842                     0    0    0    1    0   1
Ophrys-gene605134                     1    1    1    1    1   5
Ophrys-gene605135                     1    1    1    1    1   5
Ophrys-gene605137                     1    1    1    0    0   3
Ophrys-gene605138                     1    0    1    1    0   3
Ophrys-gene605283                     0    0    0    1    0   1
Ophrys-gene605725                     0    1    0    1    0   2
Ophrys-gene606641                     1    1    1    1    0   4
Ophrys-gene606713                     1    1    0    1    0   3
Ophrys-gene606875                     1    0    0    1    0   2
Ophrys-gene607159                     0    0    0    0    0   0
Ophrys-gene607160                     1    0    0    1    0   2
Ophrys-gene607150                     1    0    0    0    0   1
Ophrys-gene608034                     1    1    1    1    1   5
Ophrys-gene608036                     1    0    1    0    0   2
Ophrys-gene608038                     1    1    1    1    1   5
Ophrys-gene608076                     1    0    0    0    0   1
Ophrys-gene608079                     1    0    1    1    0   3
Ophrys-gene608081                     1    0    1    0    0   2
Ophrys-gene608255                     1    1    1    1    1   5
Ophrys-gene608277                     1    0    0    1    0   2
Ophrys-gene608283                     1    1    1    1    1   5
Ophrys-gene608252                     0    0    0    1    0   1
Ophrys-gene608253                     1    1    0    0    0   2
Ophrys-gene608254                     1    1    0    1    0   3
Ophrys-gene608780                     1    0    0    1    0   2
Ophrys-gene609121                     1    1    1    1    1   5
Ophrys-gene609127                     1    0    0    0    0   1
Ophrys-gene609113                     1    1    1    1    1   5
Ophrys-gene609335                     1    1    1    1    1   5
Ophrys-gene609320                     1    1    1    1    1   5
Ophrys-gene609321                     1    0    0    0    0   1
Ophrys-gene609383                     0    0    0    1    0   1
Ophrys-gene609384                     1    0    0    1    0   2
Ophrys-gene609387                     0    0    1    1    0   2
Ophrys-gene609851                     1    1    1    1    1   5
Ophrys-gene609978                     1    1    1    1    1   5
Ophrys-gene610039                     1    0    1    0    0   2
Ophrys-gene610159                     1    1    1    1    1   5
Ophrys-gene610217                     1    0    1    1    0   3
Ophrys-gene610331                     0    0    1    1    1   3
Ophrys-gene610360                     1    0    0    1    0   2
Ophrys-gene610393                     1    0    1    0    0   2
Ophrys-gene611136                     1    1    1    1    1   5
Ophrys-gene611988                     1    1    1    1    1   5
Ophrys-gene611989                     1    1    1    1    1   5
Ophrys-gene611990                     1    0    0    1    0   2
Ophrys-gene612000                     0    0    0    1    0   1
Ophrys-gene612006                     1    1    0    0    0   2
Ophrys-gene612094                     0    0    0    0    0   0
Ophrys-gene612095                     0    0    1    1    0   2
Ophrys-gene612096                     1    0    0    1    0   2
Ophrys-gene612113                     1    0    0    1    0   2
Ophrys-gene612218                     0    0    0    1    0   1
Ophrys-gene612304                     1    1    1    1    1   5
Ophrys-gene612306                     1    1    0    1    0   3
Ophrys-gene612316                     1    0    0    1    0   2
Ophrys-gene612322                     1    0    0    1    0   2
Ophrys-gene612409                     1    1    1    1    1   5
Ophrys-gene612419                     1    0    0    1    0   2
Ophrys-gene612425                     1    0    0    0    0   1
Ophrys-gene612508                     0    0    0    0    0   0
Ophrys-gene612509                     1    1    1    0    0   3
Ophrys-gene612510                     0    0    0    1    0   1
Ophrys-gene612526                     1    1    1    1    1   5
Ophrys-gene612567                     1    1    1    1    1   5
Ophrys-gene612591                     1    1    1    1    1   5
Ophrys-gene612566                     1    1    1    1    1   5
Ophrys-gene613471                     1    1    1    1    1   5
Ophrys-gene613472                     1    0    0    1    0   2
Ophrys-gene613549                     1    0    0    1    0   2
Ophrys-gene613553                     1    1    1    1    1   5
Ophrys-gene613621                     1    0    0    0    0   1
Ophrys-gene613696                     1    1    1    1    1   5
Ophrys-gene613744                     1    1    1    1    1   5
Ophrys-gene613745                     1    1    1    1    1   5
Ophrys-gene613750                     1    0    0    1    0   2
Ophrys-gene613751                     0    1    0    1    0   2
Ophrys-gene613850                     0    0    1    1    0   2
Ophrys-gene613875                     1    0    0    1    0   2
Ophrys-gene614320                     1    1    0    1    0   3
Ophrys-gene614943                     1    1    1    1    1   5
Ophrys-gene614944                     1    1    1    1    1   5
Ophrys-gene615021                     1    1    1    1    1   5
Ophrys-gene615405                     1    1    1    1    0   4
Ophrys-gene616641                     1    1    1    1    1   5
Ophrys-gene616645                     1    1    1    1    1   5
Ophrys-gene616684                     1    1    1    1    1   5
Ophrys-gene616662                     1    0    1    1    0   3
Ophrys-gene616663                     1    1    1    1    1   5
Ophrys-gene616734                     1    1    1    1    1   5
Ophrys-gene617117                     1    1    0    0    0   2
Ophrys-gene617119                     0    1    1    0    0   2
Ophrys-gene617150                     1    0    0    1    0   2
Ophrys-gene617200                     0    0    0    0    0   0
Ophrys-gene617203                     0    1    0    0    0   1
Ophrys-gene617232                     0    0    1    1    0   2
Ophrys-gene617233                     0    1    0    0    0   1
Ophrys-gene617279                     1    0    1    0    0   2
Ophrys-gene617281                     0    0    0    0    0   0
Ophrys-gene617284                     1    0    0    1    0   2
Ophrys-gene617310                     1    0    1    0    0   2
Ophrys-gene617312                     1    0    0    1    0   2
Ophrys-gene617356                     1    1    1    1    1   5
Ophrys-gene617357                     1    0    0    0    0   1
Ophrys-gene617359                     1    0    0    1    0   2
Ophrys-gene617387                     1    1    1    1    1   5
Ophrys-gene617388                     1    0    1    0    0   2
Ophrys-gene617389                     0    0    0    1    0   1
Ophrys-gene618203                     1    1    0    1    0   3
Ophrys-gene618330                     0    0    0    1    0   1
Ophrys-gene618337                     0    0    1    1    0   2
Ophrys-gene618338                     0    0    0    1    0   1
Ophrys-gene618378                     1    1    1    1    1   5
Ophrys-gene618384                     1    1    0    1    0   3
Ophrys-gene618385                     1    0    0    1    0   2
Ophrys-gene618424                     1    0    0    1    0   2
Ophrys-gene618431                     1    0    1    1    0   3
Ophrys-gene618432                     0    0    0    0    0   0
Ophrys-gene619121                     1    1    0    1    0   3
Ophrys-gene619104                     1    0    0    1    0   2
Ophrys-gene619105                     1    1    1    1    1   5
Ophrys-gene619112                     1    1    1    1    1   5
Ophrys-gene619486                     1    1    1    1    1   5
Ophrys-gene619606                     1    1    1    1    1   5
Ophrys-gene619607                     0    0    0    1    0   1
Ophrys-gene623434                     1    0    0    1    0   2
Ophrys-gene625920                     1    1    1    1    1   5
Ophrys-gene626198                     1    0    0    0    0   1
Ophrys-gene626266                     0    0    0    1    0   1
Ophrys-gene628377                     1    0    1    0    0   2
Ophrys-gene628383                     1    1    1    1    1   5
Ophrys-gene628431                     1    0    0    0    0   1
Ophrys-gene628425                     1    0    1    1    0   3
Ophrys-gene628430                     0    0    1    0    0   1
Ophrys-gene629888                     1    0    0    1    0   2
Ophrys-gene629938                     1    0    0    1    0   2
Ophrys-gene629936                     1    0    0    1    0   2
Ophrys-gene630001                     1    1    1    1    1   5
Ophrys-gene630002                     1    1    1    1    1   5
Ophrys-gene630252                     0    0    0    0    0   0
Ophrys-gene632792                     1    0    0    1    0   2
Ophrys-gene632827                     1    0    0    1    0   2
Ophrys-gene633270                     1    0    0    0    0   1
Ophrys-gene633357                     1    1    1    1    0   4
Ophrys-gene633364                     1    0    0    0    0   1
Ophrys-gene634116                     0    0    0    0    0   0
Ophrys-gene634117                     1    0    0    1    0   2
Ophrys-gene634133                     1    1    1    1    1   5
Ophrys-gene634134                     1    0    1    1    0   3
Ophrys-gene634135                     1    0    0    1    0   2
Ophrys-gene635223                     1    0    0    1    0   2
Ophrys-gene635224                     0    0    0    1    0   1
Ophrys-gene635317                     0    0    0    1    0   1
Ophrys-gene635298                     1    0    1    0    0   2
Ophrys-gene635826                     0    0    0    1    0   1
Ophrys-gene636288                     1    0    1    1    0   3
Ophrys-gene637464                     1    1    1    1    1   5
Ophrys-gene637465                     1    0    0    1    0   2
Ophrys-gene637466                     1    0    1    1    0   3
Ophrys-gene637467                     1    0    0    1    0   2
Ophrys-gene637468                     1    0    1    1    0   3
Ophrys-gene637469                     1    0    1    1    0   3
Ophrys-gene638849                     1    1    1    1    1   5
Ophrys-gene638834                     1    1    0    1    0   3
Ophrys-gene639687                     1    1    1    1    1   5
Ophrys-gene639886                     1    0    1    1    0   3
Ophrys-gene640091                     1    0    0    1    0   2
Ophrys-gene640092                     1    0    0    1    0   2
Ophrys-gene640093                     1    0    0    0    0   1
Ophrys-gene640074                     1    1    1    1    1   5
Ophrys-gene640142                     1    0    0    1    0   2
Ophrys-gene640143                     1    1    1    1    1   5
Ophrys-gene640144                     1    1    1    1    1   5
Ophrys-gene640149                     1    0    1    1    1   4
Ophrys-gene640198                     1    0    0    0    0   1
Ophrys-gene640180                     1    1    1    1    1   5
Ophrys-gene640181                     1    1    1    1    1   5
Ophrys-gene640272                     1    1    1    1    1   5
Ophrys-gene640541                     1    1    1    1    1   5
Ophrys-gene640774                     1    1    1    1    1   5
Ophrys-gene641681                     1    0    0    0    0   1
Ophrys-gene641684                     1    1    1    1    1   5
Ophrys-gene641774                     1    1    1    1    1   5
Ophrys-gene641780                     1    0    0    1    0   2
Ophrys-gene642579                     0    0    0    0    0   0
Ophrys-gene642586                     0    0    1    1    0   2
Ophrys-gene644196                     1    0    0    1    0   2
Ophrys-gene644197                     1    0    0    0    0   1
Ophrys-gene644489                     1    1    1    1    1   5
Ophrys-gene644529                     1    0    0    1    0   2
Ophrys-gene645076                     1    1    1    1    1   5
Ophrys-gene645081                     1    0    0    1    0   2
Ophrys-gene645126                     1    0    0    1    0   2
Ophrys-gene645252                     1    0    1    1    0   3
Ophrys-gene645254                     1    1    1    1    1   5
Ophrys-gene645854                     1    1    1    1    1   5
Ophrys-gene645855                     1    1    0    1    0   3
Ophrys-gene646079                     1    1    1    1    1   5
Ophrys-gene646703                     1    1    1    1    1   5
Ophrys-gene646710                     1    0    1    1    0   3
Ophrys-gene646682                     1    1    1    1    0   4
Ophrys-gene646782                     1    0    0    0    0   1
Ophrys-gene648797                     1    1    1    1    1   5
Ophrys-gene648798                     1    1    1    1    1   5
Ophrys-gene649358                     1    0    0    1    0   2
Ophrys-gene649395                     1    1    1    1    1   5
Ophrys-gene649688                     1    0    0    1    0   2
Ophrys-gene651231                     1    0    0    1    0   2
Ophrys-gene651232                     1    1    1    1    1   5
Ophrys-gene651226                     1    1    1    1    0   4
Ophrys-gene651229                     0    0    0    0    0   0
Ophrys-gene651532                     1    1    1    1    1   5
Ophrys-gene651534                     1    0    0    0    0   1
Ophrys-gene651587                     0    0    1    1    0   2
Ophrys-gene651589                     1    0    0    0    0   1
Ophrys-gene651590                     1    0    0    1    0   2
Ophrys-gene651648                     1    1    1    1    1   5
Ophrys-gene651781                     0    0    1    1    0   2
Ophrys-gene651783                     0    0    0    1    0   1
Ophrys-gene651784                     1    1    0    1    0   3
Ophrys-gene651822                     1    0    0    1    0   2
Ophrys-gene651824                     1    0    0    1    0   2
Ophrys-gene651897                     1    0    0    1    0   2
Ophrys-gene651898                     1    0    1    0    0   2
Ophrys-gene651900                     0    0    0    1    0   1
Ophrys-gene652043                     0    0    0    0    0   0
Ophrys-gene652058                     1    0    0    1    0   2
Ophrys-gene652059                     0    0    0    1    0   1
Ophrys-gene652060                     1    0    0    1    0   2
Ophrys-gene652061                     1    1    0    1    0   3
Ophrys-gene652910                     1    1    1    1    1   5
Ophrys-gene652911                     1    0    0    1    0   2
Ophrys-gene653615                     0    0    1    1    0   2
Ophrys-gene653627                     1    1    1    1    1   5
Ophrys-gene653672                     0    0    1    1    0   2
Ophrys-gene653684                     1    1    1    1    1   5
Ophrys-gene653904                     1    0    0    1    0   2
Ophrys-gene653906                     1    1    1    1    1   5
Ophrys-gene653918                     0    0    0    0    0   0
Ophrys-gene654359                     1    1    1    1    1   5
Ophrys-gene654344                     1    1    1    1    1   5
Ophrys-gene654799                     1    1    1    1    1   5
Ophrys-gene654800                     1    0    0    1    0   2
Ophrys-gene654889                     1    1    1    1    1   5
Ophrys-gene654980                     1    0    0    1    0   2
Ophrys-gene656625                     1    1    1    1    1   5
Ophrys-gene656666                     1    1    1    1    1   5
Ophrys-gene656692                     1    1    1    1    1   5
Ophrys-gene656780                     1    1    1    1    1   5
Ophrys-gene656782                     0    0    1    0    0   1
Ophrys-gene656819                     1    1    1    1    0   4
Ophrys-gene656821                     1    0    0    1    0   2
Ophrys-gene656857                     1    0    0    1    0   2
Ophrys-gene656858                     1    0    0    1    0   2
Ophrys-gene656859                     1    0    0    1    0   2
Ophrys-gene656894                     1    1    1    1    1   5
Ophrys-gene656896                     1    0    1    0    0   2
Ophrys-gene656927                     0    0    0    1    0   1
Ophrys-gene656929                     1    0    0    1    1   3
Ophrys-gene656958                     1    1    1    1    1   5
Ophrys-gene656959                     1    0    0    0    0   1
Ophrys-gene656950                     0    0    0    0    0   0
Ophrys-gene656989                     0    0    1    1    0   2
Ophrys-gene656991                     1    0    1    1    1   4
Ophrys-gene657021                     0    0    0    1    0   1
Ophrys-gene657018                     1    0    1    1    0   3
Ophrys-gene658627                     1    1    1    1    1   5
Ophrys-gene658645                     1    1    0    1    0   3
Ophrys-gene658617                     0    0    1    1    0   2
Ophrys-gene658765                     1    0    0    1    0   2
Ophrys-gene658884                     1    1    1    1    1   5
Ophrys-gene658885                     1    1    1    1    1   5
Ophrys-gene660141                     1    1    1    1    1   5
Ophrys-gene660346                     0    0    1    1    0   2
Ophrys-gene660366                     1    1    1    1    1   5
Ophrys-gene660367                     1    0    1    0    0   2
Ophrys-gene661213                     1    0    0    0    0   1
Ophrys-gene661214                     1    1    1    1    1   5
Ophrys-gene661337                     1    1    1    1    1   5
Ophrys-gene661409                     1    1    0    1    0   3
Ophrys-gene661401                     0    1    0    0    0   1
Ophrys-gene661496                     1    1    1    1    1   5
Ophrys-gene661497                     1    0    0    1    0   2
Ophrys-gene661515                     1    0    0    0    0   1
Ophrys-gene661550                     1    0    0    1    0   2
Ophrys-gene661552                     1    0    0    1    0   2
Ophrys-gene661539                     1    1    1    1    1   5
Ophrys-gene661648                     1    1    0    0    0   2
Ophrys-gene661671                     1    0    0    0    0   1
Ophrys-gene661635                     1    0    0    1    0   2
Ophrys-gene661636                     1    0    0    0    0   1
Ophrys-gene662243                     1    1    1    1    1   5
Ophrys-gene662671                     0    0    0    1    0   1
Ophrys-gene662672                     1    1    1    1    1   5
Ophrys-gene663410                     1    1    1    1    1   5
Ophrys-gene663846                     1    0    0    1    0   2
Ophrys-gene663848                     1    1    1    1    1   5
Ophrys-gene663853                     1    0    0    1    0   2
Ophrys-gene664876                     1    0    1    1    0   3
Ophrys-gene665869                     1    1    1    1    1   5
Ophrys-gene665825                     1    0    0    1    0   2
Ophrys-gene665826                     1    0    0    1    0   2
Ophrys-gene665833                     1    0    0    1    0   2
Ophrys-gene665834                     1    0    0    1    0   2
Ophrys-gene665958                     1    0    0    0    0   1
Ophrys-gene666830                     1    1    1    1    1   5
Ophrys-gene669056                     1    1    1    1    0   4
Ophrys-gene669081                     1    0    0    1    0   2
Ophrys-gene670083                     1    1    1    1    1   5
Ophrys-gene670613                     1    0    0    1    0   2
Ophrys-gene670659                     0    0    0    1    0   1
Ophrys-gene671029                     1    0    1    1    0   3
Ophrys-gene671031                     0    0    1    0    0   1
Ophrys-gene671379                     1    1    1    1    1   5
Ophrys-gene671380                     1    0    0    0    0   1
Ophrys-gene672397                     1    1    1    1    1   5
Ophrys-gene672409                     1    0    1    1    0   3
Ophrys-gene672414                     1    1    1    1    1   5
Ophrys-gene672568                     1    1    1    1    1   5
Ophrys-gene672572                     1    1    1    1    1   5
Ophrys-gene673009                     1    0    0    1    0   2
Ophrys-gene673498                     1    0    0    0    1   2
Ophrys-gene673499                     1    0    0    1    0   2
Ophrys-gene673503                     0    0    1    1    0   2
Ophrys-gene673508                     1    0    0    0    0   1
Ophrys-gene674286                     1    0    0    1    0   2
Ophrys-gene674287                     1    1    1    1    1   5
Ophrys-gene675345                     1    0    0    1    0   2
Ophrys-gene675317                     1    0    0    1    0   2
Ophrys-gene675409                     0    0    0    1    0   1
Ophrys-gene676226                     1    0    0    1    0   2
Ophrys-gene676402                     1    0    0    0    0   1
Ophrys-gene676443                     1    1    1    1    1   5
Ophrys-gene676485                     1    1    1    1    1   5
Ophrys-gene676623                     1    1    1    1    1   5
Ophrys-gene676887                     1    1    0    1    0   3
Ophrys-gene676891                     0    0    0    1    0   1
Ophrys-gene676954                     1    0    1    1    0   3
Ophrys-gene676971                     1    1    1    1    1   5
Ophrys-gene676947                     1    0    0    1    0   2
Ophrys-gene677063                     0    0    0    1    0   1
Ophrys-gene677064                     0    0    0    1    0   1
Ophrys-gene677078                     0    0    1    1    0   2
Ophrys-gene677092                     1    0    1    1    0   3
Ophrys-gene677094                     1    0    0    1    0   2
Ophrys-gene677096                     1    1    1    1    1   5
Ophrys-gene677099                     0    0    0    1    0   1
Ophrys-gene677166                     0    1    0    0    0   1
Ophrys-gene677167                     0    0    1    1    0   2
Ophrys-gene677169                     1    0    0    1    0   2
Ophrys-gene677956                     1    1    1    1    1   5
Ophrys-gene677957                     0    0    0    0    0   0
Ophrys-gene677965                     1    1    1    1    1   5
Ophrys-gene679681                     0    0    1    0    1   2
Ophrys-gene680084                     1    1    1    1    1   5
Ophrys-gene680088                     1    1    1    1    1   5
Ophrys-gene680823                     1    1    1    1    1   5
Ophrys-gene680825                     1    1    1    1    1   5
Ophrys-gene680826                     1    0    1    1    0   3
Ophrys-gene681248                     1    0    1    0    0   2
Ophrys-gene681261                     1    0    1    1    0   3
Ophrys-gene681280                     1    1    1    1    1   5
Ophrys-gene681238                     1    1    1    1    1   5
Ophrys-gene681956                     1    0    1    0    0   2
Ophrys-gene681947                     0    0    0    1    0   1
Ophrys-gene681950                     0    0    0    1    0   1
Ophrys-gene682214                     1    0    0    1    0   2
Ophrys-gene682215                     1    1    1    1    1   5
Ophrys-gene683101                     1    0    1    1    0   3
Ophrys-gene683105                     0    0    0    1    0   1
Ophrys-gene683711                     1    1    1    1    1   5
Ophrys-gene683712                     1    1    1    1    1   5
Ophrys-gene683889                     1    0    1    0    0   2
Ophrys-gene684264                     0    0    0    1    0   1
Ophrys-gene684348                     1    1    1    1    1   5
Ophrys-gene684613                     1    1    1    1    1   5
Ophrys-gene684614                     1    1    0    1    0   3
Ophrys-gene684593                     0    0    0    1    0   1
Ophrys-gene684659                     1    1    1    1    1   5
Ophrys-gene684771                     1    1    1    1    1   5
Ophrys-gene685357                     1    1    1    1    1   5
Ophrys-gene685733                     1    0    1    0    0   2
Ophrys-gene687401                     1    0    1    1    0   3
Ophrys-gene687407                     0    0    0    1    0   1
Ophrys-gene687539                     1    0    0    1    0   2
Ophrys-gene687540                     1    0    1    1    0   3
Ophrys-gene687647                     1    1    1    1    1   5
Ophrys-gene687838                     1    1    1    1    1   5
Ophrys-gene689350                     1    1    1    1    1   5
Ophrys-gene689684                     1    1    1    1    1   5
Ophrys-gene689693                     0    0    0    1    0   1
Ophrys-gene689733                     1    1    1    1    0   4
Ophrys-gene689734                     1    1    1    1    1   5
Ophrys-gene689746                     1    0    0    1    0   2
Ophrys-gene689786                     1    1    1    1    1   5
Ophrys-gene689930                     0    0    0    1    0   1
Ophrys-gene690166                     0    1    1    0    0   2
Ophrys-gene690167                     1    0    0    1    0   2
Ophrys-gene690168                     1    0    0    0    0   1
Ophrys-gene690217                     1    0    0    1    0   2
Ophrys-gene690360                     1    1    1    1    1   5
Ophrys-gene690451                     1    1    0    1    0   3
Ophrys-gene690503                     1    1    1    1    1   5
Ophrys-gene690882                     1    0    0    1    0   2
Ophrys-gene690884                     1    1    1    1    1   5
Ophrys-gene691174                     1    0    1    1    1   4
Ophrys-gene691425                     1    0    1    1    0   3
Ophrys-gene691927                     1    0    0    0    0   1
Ophrys-gene692040                     1    0    0    1    0   2
Ophrys-gene692041                     0    0    0    0    0   0
Ophrys-gene692653                     1    0    0    1    0   2
Ophrys-gene692642                     0    0    0    1    0   1
Ophrys-gene692701                     0    0    0    0    0   0
Ophrys-gene692702                     0    0    0    1    0   1
Ophrys-gene692736                     0    1    0    0    0   1
Ophrys-gene692734                     1    0    0    1    0   2
Ophrys-gene692824                     0    0    0    0    1   1
Ophrys-gene692825                     1    0    0    0    1   2
Ophrys-gene692918                     1    1    1    1    1   5
Ophrys-gene693032                     1    1    1    1    1   5
Ophrys-gene693101                     1    1    1    1    1   5
Ophrys-gene693204                     0    0    1    1    0   2
Phalaenopsis-gene100346               0    0    1    0    0   1
Phalaenopsis-gene100350               1    0    1    0    0   2
Phalaenopsis-gene100593               1    1    1    1    0   4
Phalaenopsis-gene100600               1    1    1    1    1   5
Phalaenopsis-gene100601               1    1    1    1    0   4
Phalaenopsis-gene101265               1    1    1    1    0   4
Phalaenopsis-gene101420               1    1    1    1    1   5
Phalaenopsis-gene103181               1    1    1    1    1   5
Phalaenopsis-gene103260               0    0    0    0    0   0
Phalaenopsis-gene103288               1    1    1    0    0   3
Phalaenopsis-gene103328               1    0    1    1    0   3
Phalaenopsis-gene103368               1    1    1    1    1   5
Phalaenopsis-gene103618               1    1    1    1    1   5
Phalaenopsis-gene103738               0    0    0    0    0   0
Phalaenopsis-gene104554               1    0    1    0    0   2
Phalaenopsis-gene105014               1    1    1    1    1   5
Phalaenopsis-gene105035               1    1    1    1    1   5
Phalaenopsis-gene105651               1    1    1    1    1   5
Phalaenopsis-gene105652               0    0    0    1    0   1
Phalaenopsis-gene107063               1    1    1    1    1   5
Phalaenopsis-gene107352               0    0    0    0    0   0
Phalaenopsis-gene107362               1    0    1    1    0   3
Phalaenopsis-gene107619               1    0    1    1    0   3
Phalaenopsis-gene107715               1    1    1    1    1   5
Phalaenopsis-gene107762               0    1    1    1    0   3
Phalaenopsis-gene108048               1    1    1    1    1   5
Phalaenopsis-gene110585               1    1    1    1    1   5
Phalaenopsis-gene110603               1    1    1    1    1   5
Phalaenopsis-gene11231                1    0    1    0    0   2
Phalaenopsis-gene11243                0    0    0    1    0   1
Phalaenopsis-gene11263                1    1    1    1    1   5
Phalaenopsis-gene11264                1    0    1    1    0   3
Phalaenopsis-gene113083               1    0    1    1    0   3
Phalaenopsis-gene113130               0    0    1    1    0   2
Phalaenopsis-gene113162               1    0    1    1    0   3
Phalaenopsis-gene113299               1    0    0    0    0   1
Phalaenopsis-gene113326               1    0    0    1    0   2
Phalaenopsis-gene115257               1    1    1    1    1   5
Phalaenopsis-gene115946               1    1    1    1    0   4
Phalaenopsis-gene116607               0    0    1    1    0   2
Phalaenopsis-gene117158               1    1    1    1    1   5
Phalaenopsis-gene118543               0    0    0    1    0   1
Phalaenopsis-gene118575               1    0    0    1    0   2
Phalaenopsis-gene118576               1    0    1    1    0   3
Phalaenopsis-gene118639               1    1    0    0    0   2
Phalaenopsis-gene118673               1    0    1    1    0   3
Phalaenopsis-gene11884                1    0    1    1    0   3
Phalaenopsis-gene119445               0    0    1    1    0   2
Phalaenopsis-gene11956                0    0    0    0    0   0
Phalaenopsis-gene120197               1    1    1    1    1   5
Phalaenopsis-gene120220               1    1    1    1    1   5
Phalaenopsis-gene120227               1    0    1    1    0   3
Phalaenopsis-gene120531               1    1    1    1    0   4
Phalaenopsis-gene120534               1    1    1    1    1   5
Phalaenopsis-gene120535               1    1    1    1    0   4
Phalaenopsis-gene120551               1    1    1    1    1   5
Phalaenopsis-gene120562               1    1    1    1    0   4
Phalaenopsis-gene120563               1    1    1    1    1   5
Phalaenopsis-gene122340               1    0    0    1    0   2
Phalaenopsis-gene122393               1    0    0    1    0   2
Phalaenopsis-gene122626               0    0    0    1    0   1
Phalaenopsis-gene122627               1    0    0    0    0   1
Phalaenopsis-gene122663               0    0    0    1    0   1
Phalaenopsis-gene122664               0    0    0    1    0   1
Phalaenopsis-gene122703               1    0    0    0    0   1
Phalaenopsis-gene122796               1    0    0    1    0   2
Phalaenopsis-gene124812               1    0    1    1    0   3
Phalaenopsis-gene124872               1    1    1    1    1   5
Phalaenopsis-gene125020               1    0    1    1    0   3
Phalaenopsis-gene125025               1    1    1    1    0   4
Phalaenopsis-gene125029               0    0    0    1    0   1
Phalaenopsis-gene125081               1    1    1    1    1   5
Phalaenopsis-gene125092               1    0    1    1    0   3
Phalaenopsis-gene125354               1    1    1    1    1   5
Phalaenopsis-gene1273                 0    0    0    1    0   1
Phalaenopsis-gene1274                 1    0    0    1    0   2
Phalaenopsis-gene127762               1    1    1    1    1   5
Phalaenopsis-gene128029               0    0    1    1    0   2
Phalaenopsis-gene128031               1    1    1    1    1   5
Phalaenopsis-gene128279               0    1    0    1    0   2
Phalaenopsis-gene128280               0    0    0    1    0   1
Phalaenopsis-gene128301               0    0    0    1    0   1
Phalaenopsis-gene128302               1    1    1    1    1   5
Phalaenopsis-gene128418               1    1    1    1    1   5
Phalaenopsis-gene128905               0    0    1    1    0   2
Phalaenopsis-gene128906               1    1    1    0    0   3
Phalaenopsis-gene128914               1    0    1    1    0   3
Phalaenopsis-gene128915               1    0    0    0    0   1
Phalaenopsis-gene128924               1    1    1    1    1   5
Phalaenopsis-gene129539               1    0    1    0    0   2
Phalaenopsis-gene129636               1    0    1    1    0   3
Phalaenopsis-gene129637               0    0    0    1    0   1
Phalaenopsis-gene129707               1    0    0    1    0   2
Phalaenopsis-gene129801               1    0    0    1    0   2
Phalaenopsis-gene129803               1    1    1    1    1   5
Phalaenopsis-gene129805               1    0    1    0    0   2
Phalaenopsis-gene129814               1    0    1    1    0   3
Phalaenopsis-gene129815               1    0    1    1    0   3
Phalaenopsis-gene129917               0    0    1    1    0   2
Phalaenopsis-gene130031               0    0    0    1    0   1
Phalaenopsis-gene130032               1    0    0    0    0   1
Phalaenopsis-gene130305               0    0    0    1    0   1
Phalaenopsis-gene130333               1    0    1    1    0   3
Phalaenopsis-gene130389               1    0    0    1    0   2
Phalaenopsis-gene130418               1    0    0    0    0   1
Phalaenopsis-gene130554               0    0    0    1    0   1
Phalaenopsis-gene130556               0    0    1    1    0   2
Phalaenopsis-gene130560               0    0    1    1    0   2
Phalaenopsis-gene130564               1    1    1    1    1   5
Phalaenopsis-gene130581               0    0    1    0    1   2
Phalaenopsis-gene130583               0    0    0    1    0   1
Phalaenopsis-gene130587               1    1    1    1    1   5
Phalaenopsis-gene130591               0    0    0    1    0   1
Phalaenopsis-gene130949               1    0    1    1    0   3
Phalaenopsis-gene130950               1    0    0    1    0   2
Phalaenopsis-gene131001               1    1    1    1    1   5
Phalaenopsis-gene131002               1    0    0    1    0   2
Phalaenopsis-gene131310               1    1    1    1    1   5
Phalaenopsis-gene132402               1    1    1    1    1   5
Phalaenopsis-gene132797               0    0    1    1    0   2
Phalaenopsis-gene132799               1    0    1    0    0   2
Phalaenopsis-gene132913               1    0    0    1    0   2
Phalaenopsis-gene132966               1    0    1    1    0   3
Phalaenopsis-gene132997               0    1    0    0    0   1
Phalaenopsis-gene133018               0    0    0    1    0   1
Phalaenopsis-gene133111               0    0    0    1    0   1
Phalaenopsis-gene133112               1    1    1    1    1   5
Phalaenopsis-gene133127               1    0    0    0    0   1
Phalaenopsis-gene134380               1    1    0    1    0   3
Phalaenopsis-gene134499               1    0    0    0    0   1
Phalaenopsis-gene135384               1    0    1    1    0   3
Phalaenopsis-gene135572               0    0    1    1    0   2
Phalaenopsis-gene135765               1    0    0    1    0   2
Phalaenopsis-gene136104               1    1    1    1    1   5
Phalaenopsis-gene136105               1    0    1    0    0   2
Phalaenopsis-gene136114               1    0    0    1    0   2
Phalaenopsis-gene136574               0    0    0    1    0   1
Phalaenopsis-gene137169               1    0    0    0    0   1
Phalaenopsis-gene137332               1    1    1    1    1   5
Phalaenopsis-gene137335               0    0    1    1    0   2
Phalaenopsis-gene137504               1    1    1    1    1   5
Phalaenopsis-gene137505               1    1    1    1    1   5
Phalaenopsis-gene137833               1    0    0    0    0   1
Phalaenopsis-gene137849               1    0    0    1    0   2
Phalaenopsis-gene138421               1    0    0    1    0   2
Phalaenopsis-gene138453               1    0    1    0    1   3
Phalaenopsis-gene138716               1    1    1    1    1   5
Phalaenopsis-gene138858               1    1    1    1    1   5
Phalaenopsis-gene139211               1    0    0    1    0   2
Phalaenopsis-gene139212               0    0    0    1    0   1
Phalaenopsis-gene140600               0    0    0    1    0   1
Phalaenopsis-gene140605               1    1    1    1    1   5
Phalaenopsis-gene140609               1    0    1    1    0   3
Phalaenopsis-gene140995               0    0    0    1    0   1
Phalaenopsis-gene141866               1    1    0    1    0   3
Phalaenopsis-gene141904               1    1    1    1    1   5
Phalaenopsis-gene142521               0    0    0    1    0   1
Phalaenopsis-gene142522               0    0    1    0    0   1
Phalaenopsis-gene142545               1    0    1    0    0   2
Phalaenopsis-gene142546               1    1    1    1    1   5
Phalaenopsis-gene143080               1    0    1    1    0   3
Phalaenopsis-gene143081               1    1    1    1    1   5
Phalaenopsis-gene143099               1    1    1    1    1   5
Phalaenopsis-gene143100               1    0    0    1    0   2
Phalaenopsis-gene143164               1    0    1    1    1   4
Phalaenopsis-gene143165               1    0    0    0    0   1
Phalaenopsis-gene143171               1    1    1    1    1   5
Phalaenopsis-gene143172               1    0    0    1    0   2
Phalaenopsis-gene143197               0    0    1    0    0   1
Phalaenopsis-gene143198               1    1    1    1    1   5
Phalaenopsis-gene143481               1    1    1    1    1   5
Phalaenopsis-gene143503               0    1    0    0    0   1
Phalaenopsis-gene143509               1    1    1    1    1   5
Phalaenopsis-gene143516               1    1    1    1    1   5
Phalaenopsis-gene143530               1    1    1    1    1   5
Phalaenopsis-gene143548               1    0    0    1    0   2
Phalaenopsis-gene143585               0    0    0    1    0   1
Phalaenopsis-gene143613               0    0    0    1    0   1
Phalaenopsis-gene143642               1    0    1    0    1   3
Phalaenopsis-gene143754               1    1    1    1    0   4
Phalaenopsis-gene14382                0    0    1    1    0   2
Phalaenopsis-gene14383                0    0    1    0    0   1
Phalaenopsis-gene144863               1    1    1    1    0   4
Phalaenopsis-gene144888               1    0    1    1    0   3
Phalaenopsis-gene144900               1    0    0    0    1   2
Phalaenopsis-gene144901               1    1    1    0    0   3
Phalaenopsis-gene144933               1    0    0    1    0   2
Phalaenopsis-gene144945               1    0    0    1    0   2
Phalaenopsis-gene145021               1    0    1    1    0   3
Phalaenopsis-gene145022               0    0    0    1    0   1
Phalaenopsis-gene145034               0    0    1    1    0   2
Phalaenopsis-gene145110               1    0    1    0    1   3
Phalaenopsis-gene145111               0    0    0    1    0   1
Phalaenopsis-gene145123               1    1    1    1    1   5
Phalaenopsis-gene145152               1    0    0    1    0   2
Phalaenopsis-gene145153               1    0    0    0    0   1
Phalaenopsis-gene145164               1    0    0    1    0   2
Phalaenopsis-gene145165               1    0    1    1    0   3
Phalaenopsis-gene145181               1    0    0    1    0   2
Phalaenopsis-gene145191               1    0    0    1    0   2
Phalaenopsis-gene145192               1    0    0    1    0   2
Phalaenopsis-gene145264               1    0    1    0    0   2
Phalaenopsis-gene145305               1    0    1    1    0   3
Phalaenopsis-gene145555               1    1    1    1    1   5
Phalaenopsis-gene145560               1    1    1    1    1   5
Phalaenopsis-gene145561               1    1    1    1    1   5
Ophrys-gene11088                      1    1    1    1    1   5
Ophrys-gene21822                      1    1    1    1    1   5
Ophrys-gene21823                      1    1    1    1    1   5
Ophrys-gene22140                      1    1    1    1    1   5
Ophrys-gene23702                      1    0    0    1    0   2
Ophrys-gene24863                      1    1    1    1    1   5
Ophrys-gene46809                      1    1    1    1    1   5
Ophrys-gene47388                      1    1    1    1    1   5
Ophrys-gene68291                      1    0    1    0    0   2
Ophrys-gene68447                      1    1    1    1    1   5
Ophrys-gene73730                      0    0    0    1    0   1
Ophrys-gene76052                      1    1    1    1    1   5
Ophrys-gene76833                      1    1    1    1    1   5
Ophrys-gene76880                      1    1    0    1    0   3
Ophrys-gene76972                      0    0    0    1    0   1
Ophrys-gene82967                      1    0    1    1    0   3
Ophrys-gene104616                     1    1    1    1    1   5
Ophrys-gene104745                     1    0    0    1    0   2
Ophrys-gene108200                     1    0    0    1    0   2
Ophrys-gene111993                     1    0    0    1    0   2
Ophrys-gene134450                     1    1    1    1    1   5
Ophrys-gene134917                     1    0    0    1    0   2
Ophrys-gene140935                     1    0    0    0    1   2
Ophrys-gene142565                     1    1    0    1    0   3
Ophrys-gene147898                     1    0    0    1    0   2
Ophrys-gene150247                     1    1    1    1    1   5
Ophrys-gene159907                     1    0    0    1    0   2
Ophrys-gene163655                     1    1    1    1    1   5
Ophrys-gene166637                     1    1    1    1    1   5
Ophrys-gene168096                     0    0    0    1    0   1
Ophrys-gene168154                     1    1    1    1    1   5
Ophrys-gene174339                     1    1    1    1    1   5
Ophrys-gene176443                     0    0    0    1    0   1
Ophrys-gene176483                     0    0    0    1    0   1
Ophrys-gene177774                     1    1    1    1    1   5
Ophrys-gene207829                     1    0    1    0    0   2
Ophrys-gene208447                     1    0    0    0    0   1
Ophrys-gene210743                     1    0    0    1    0   2
Ophrys-gene211195                     1    1    1    1    1   5
Ophrys-gene225014                     1    0    0    0    0   1
Ophrys-gene225015                     0    0    1    1    0   2
Ophrys-gene225055                     0    0    0    1    0   1
Ophrys-gene226774                     1    0    1    0    0   2
Ophrys-gene231633                     1    0    0    1    0   2
Ophrys-gene247324                     1    0    0    1    0   2
Ophrys-gene274566                     0    0    0    0    0   0
Ophrys-gene277597                     1    1    1    1    1   5
Ophrys-gene279226                     0    0    0    0    0   0
Ophrys-gene280606                     1    0    0    1    0   2
Ophrys-gene284927                     1    1    1    1    1   5
Ophrys-gene288480                     0    0    0    0    0   0
Ophrys-gene288536                     1    0    1    1    0   3
Ophrys-gene289122                     1    0    0    0    0   1
Ophrys-gene290730                     1    0    1    1    0   3
Ophrys-gene291016                     1    1    1    1    1   5
Ophrys-gene332809                     1    0    0    1    0   2
Ophrys-gene332879                     1    0    0    1    0   2
Ophrys-gene340174                     1    1    1    1    1   5
Ophrys-gene340175                     1    0    1    1    0   3
Ophrys-gene360852                     1    1    1    1    1   5
Ophrys-gene378320                     1    0    0    1    0   2
Ophrys-gene378365                     1    0    0    1    0   2
Ophrys-gene378436                     0    0    1    0    0   1
Ophrys-gene398686                     1    0    0    1    0   2
Ophrys-gene399942                     1    1    1    1    0   4
Ophrys-gene399943                     1    0    1    1    0   3
Ophrys-gene399989                     1    0    1    1    0   3
Ophrys-gene399990                     1    0    0    0    0   1
Ophrys-gene400541                     1    1    0    1    1   4
Ophrys-gene405800                     0    0    0    0    0   0
Ophrys-gene407299                     1    0    0    1    0   2
Ophrys-gene407321                     1    1    1    1    1   5
Ophrys-gene407344                     1    0    1    1    0   3
Ophrys-gene422290                     1    0    0    0    0   1
Ophrys-gene429061                     1    1    1    1    1   5
Ophrys-gene435955                     0    0    0    0    0   0
Ophrys-gene436071                     1    1    1    1    1   5
Ophrys-gene436213                     1    0    0    1    0   2
Ophrys-gene441575                     1    0    1    0    0   2
Ophrys-gene442835                     1    1    1    1    1   5
Ophrys-gene444891                     1    0    1    1    1   4
Ophrys-gene455001                     1    1    1    1    1   5
Ophrys-gene455002                     1    1    1    1    1   5
Ophrys-gene457591                     1    0    0    1    0   2
Ophrys-gene476821                     1    1    1    1    1   5
Ophrys-gene487783                     1    1    1    1    1   5
Ophrys-gene487807                     1    0    1    1    0   3
Ophrys-gene494361                     1    0    1    0    0   2
Ophrys-gene494362                     0    0    1    1    0   2
Ophrys-gene508461                     1    1    1    1    1   5
Ophrys-gene528889                     1    1    1    1    1   5
Ophrys-gene531295                     1    1    1    1    1   5
Ophrys-gene535250                     1    1    1    1    1   5
Ophrys-gene537707                     1    1    1    1    1   5
Ophrys-gene564587                     1    1    1    1    1   5
Ophrys-gene564588                     1    0    0    1    0   2
Ophrys-gene564657                     1    1    1    1    1   5
Ophrys-gene564658                     1    0    0    0    0   1
Ophrys-gene564711                     1    0    0    1    0   2
Ophrys-gene574629                     1    1    1    1    1   5
Ophrys-gene579273                     1    0    0    1    0   2
Ophrys-gene581707                     1    1    1    1    1   5
Ophrys-gene585659                     0    0    1    0    0   1
Ophrys-gene587027                     1    1    1    1    1   5
Ophrys-gene587028                     0    0    1    1    0   2
Ophrys-gene587121                     0    0    1    1    0   2
Ophrys-gene601429                     1    1    1    1    1   5
Ophrys-gene603953                     1    1    1    1    1   5
Ophrys-gene609110                     1    1    1    1    1   5
Ophrys-gene609111                     1    1    1    1    1   5
Ophrys-gene612112                     0    1    1    1    0   3
Ophrys-gene616736                     0    0    0    1    0   1
Ophrys-gene616766                     1    1    1    1    1   5
Ophrys-gene627293                     1    1    1    1    1   5
Ophrys-gene636290                     0    0    0    1    0   1
Ophrys-gene646939                     1    0    1    1    0   3
Ophrys-gene646981                     1    0    0    0    0   1
Ophrys-gene646985                     1    1    1    1    1   5
Ophrys-gene651228                     1    1    1    1    1   5
Ophrys-gene653917                     0    1    0    1    0   2
Ophrys-gene664288                     1    0    0    1    0   2
Ophrys-gene664766                     1    0    1    0    0   2
Ophrys-gene664767                     1    0    1    1    0   3
Ophrys-gene680818                     1    0    1    1    0   3
Ophrys-gene689931                     1    0    0    0    0   1
Ophrys-gene690218                     1    0    0    1    0   2
Ophrys-gene691426                     1    1    1    1    1   5
Ophrys-gene703534                     1    1    1    1    1   5
Ophrys-gene726169                     1    0    1    1    0   3
Ophrys-gene730976                     1    1    1    1    1   5
Ophrys-gene748104                     1    1    1    1    1   5
Ophrys-gene756284                     1    1    1    1    1   5
Ophrys-gene756285                     0    0    1    1    0   2
Ophrys-gene756314                     1    1    1    1    1   5
Ophrys-gene756315                     1    0    1    1    0   3
Phalaenopsis-gene203687               1    0    0    1    0   2
Phalaenopsis-gene107951               1    0    0    0    0   1
Phalaenopsis-gene107940               1    0    0    0    0   1
Phalaenopsis-gene107985               0    0    1    0    0   1
Phalaenopsis-gene575561               1    0    1    1    0   3
Phalaenopsis-gene203743               1    0    0    1    0   2
Phalaenopsis-gene107928               1    1    0    0    0   2
Phalaenopsis-gene575520               1    0    1    0    0   2
Phalaenopsis-gene203686               0    0    1    0    0   1
Phalaenopsis-gene108054               1    0    1    1    0   3
Phalaenopsis-gene575521               0    0    1    1    0   2
Phalaenopsis-gene575544               1    0    1    1    0   3
Phalaenopsis-gene107934               0    0    0    0    0   0
Phalaenopsis-gene108034               1    0    0    1    0   2
Phalaenopsis-gene859027               1    0    1    1    0   3
Phalaenopsis-gene108064               0    0    0    0    0   0
Phalaenopsis-gene575543               1    0    1    0    0   2
Phalaenopsis-gene107941               0    0    1    1    0   2
Phalaenopsis-gene575560               1    1    1    1    0   4
Phalaenopsis-gene432585               1    1    1    1    1   5
Phalaenopsis-gene107991               1    0    1    0    0   2
Phalaenopsis-gene203579               1    0    1    0    0   2
Phalaenopsis-gene575539               1    0    0    1    0   2
Phalaenopsis-gene107927               1    0    1    1    0   3
Phalaenopsis-gene107920               0    0    0    0    0   0
Phalaenopsis-gene666                  1    0    0    1    0   2
Phalaenopsis-gene9999                 1    1    1    1    1   5
Phalaenopsis-gene107977               0    0    0    1    0   1
Phalaenopsis-gene859026               0    0    0    1    0   1
Phalaenopsis-gene108007               1    0    0    1    0   2
Phalaenopsis-gene203526               0    0    0    1    0   1
Phalaenopsis-gene107983               1    0    0    1    1   3
Phalaenopsis-gene432582               1    0    1    1    0   3
Phalaenopsis-gene108042               1    1    1    1    0   4
Phalaenopsis-gene108065               1    1    1    0    0   3
Phalaenopsis-gene107998               1    0    1    0    0   2
Phalaenopsis-gene203525               1    0    0    0    0   1
Phalaenopsis-gene575493               1    0    1    1    0   3
Phalaenopsis-gene575565               0    0    1    1    0   2
Phalaenopsis-gene107984               1    1    1    1    1   5
Phalaenopsis-gene203470               1    0    0    1    0   2
Phalaenopsis-gene108033               1    1    1    1    1   5
Phalaenopsis-gene203471               1    0    1    0    0   2
Phalaenopsis-gene107926               0    0    0    1    0   1
Phalaenopsis-gene145575               1    1    1    1    1   5
Phalaenopsis-gene145576               1    1    1    1    1   5
Phalaenopsis-gene145892               1    1    1    1    1   5
Phalaenopsis-gene146056               0    0    0    1    0   1
Phalaenopsis-gene146057               1    1    1    1    1   5
Phalaenopsis-gene146203               1    1    1    1    0   4
Phalaenopsis-gene146401               1    1    1    1    1   5
Phalaenopsis-gene146701               0    0    1    1    0   2
Phalaenopsis-gene146712               1    0    1    1    0   3
Phalaenopsis-gene146732               1    1    1    1    1   5
Phalaenopsis-gene146736               1    1    1    1    0   4
Phalaenopsis-gene146737               1    0    0    0    0   1
Phalaenopsis-gene146773               1    1    1    1    1   5
Phalaenopsis-gene147092               1    1    1    1    1   5
Phalaenopsis-gene147093               0    0    0    1    0   1
Phalaenopsis-gene147626               1    1    1    1    1   5
Phalaenopsis-gene148783               1    0    0    0    0   1
Phalaenopsis-gene148790               1    0    1    1    0   3
Phalaenopsis-gene148791               0    0    1    1    0   2
Phalaenopsis-gene149263               1    0    0    1    0   2
Phalaenopsis-gene149288               1    0    0    1    0   2
Phalaenopsis-gene149289               1    1    0    1    0   3
Phalaenopsis-gene149467               0    1    1    0    0   2
Phalaenopsis-gene149486               1    1    1    0    0   3
Phalaenopsis-gene149487               1    1    1    1    1   5
Phalaenopsis-gene149799               1    0    1    1    0   3
Phalaenopsis-gene149895               1    0    0    1    0   2
Phalaenopsis-gene149917               0    0    0    1    0   1
Phalaenopsis-gene149942               1    0    0    1    0   2
Phalaenopsis-gene150116               0    0    1    1    0   2
Phalaenopsis-gene150121               1    0    1    1    0   3
Phalaenopsis-gene150135               1    0    1    0    0   2
Phalaenopsis-gene150136               0    0    1    1    0   2
Phalaenopsis-gene150142               1    0    1    1    0   3
Phalaenopsis-gene150159               1    1    1    0    0   3
Phalaenopsis-gene150163               0    1    0    0    0   1
Phalaenopsis-gene150192               0    0    1    1    0   2
Phalaenopsis-gene150270               0    0    0    0    0   0
Phalaenopsis-gene150284               1    0    1    1    0   3
Phalaenopsis-gene150291               1    0    1    0    0   2
Phalaenopsis-gene150308               1    1    1    1    1   5
Phalaenopsis-gene150312               1    0    0    1    1   3
Phalaenopsis-gene150341               0    0    0    1    0   1
Phalaenopsis-gene150377               1    1    1    1    1   5
Phalaenopsis-gene150589               1    0    0    1    0   2
Phalaenopsis-gene150603               1    1    1    1    1   5
Phalaenopsis-gene150604               1    0    0    1    0   2
Phalaenopsis-gene150845               1    0    0    0    0   1
Phalaenopsis-gene151068               0    0    0    1    0   1
Phalaenopsis-gene151712               1    0    0    0    0   1
Phalaenopsis-gene151719               1    0    0    1    0   2
Phalaenopsis-gene151750               0    0    1    1    0   2
Phalaenopsis-gene151757               1    0    0    1    0   2
Phalaenopsis-gene152821               1    1    1    1    1   5
Phalaenopsis-gene152822               1    1    1    1    1   5
Phalaenopsis-gene152902               0    0    1    0    0   1
Phalaenopsis-gene152943               0    0    1    1    0   2
Phalaenopsis-gene152978               1    1    1    1    1   5
Phalaenopsis-gene152989               1    0    1    1    0   3
Phalaenopsis-gene154092               1    1    0    1    0   3
Phalaenopsis-gene154112               1    1    1    1    1   5
Phalaenopsis-gene154160               1    0    1    0    0   2
Phalaenopsis-gene154161               1    0    1    1    0   3
Phalaenopsis-gene1546                 1    0    0    1    1   3
Phalaenopsis-gene1547                 1    1    1    1    1   5
Phalaenopsis-gene155095               1    1    1    1    1   5
Phalaenopsis-gene155386               1    0    1    1    0   3
Phalaenopsis-gene155736               1    0    0    1    0   2
Phalaenopsis-gene156003               1    0    1    1    0   3
Phalaenopsis-gene156022               1    1    1    1    1   5
Phalaenopsis-gene156029               1    1    1    1    1   5
Phalaenopsis-gene156030               1    1    1    1    1   5
Phalaenopsis-gene156038               1    1    1    1    1   5
Phalaenopsis-gene156048               1    0    0    1    0   2
Phalaenopsis-gene156053               0    0    1    0    0   1
Phalaenopsis-gene156090               1    0    0    1    0   2
Phalaenopsis-gene156110               1    1    1    1    1   5
Phalaenopsis-gene156111               1    1    1    1    1   5
Phalaenopsis-gene156119               1    1    1    1    1   5
Phalaenopsis-gene156155               1    0    0    1    0   2
Phalaenopsis-gene156159               0    0    1    1    0   2
Phalaenopsis-gene156165               1    1    1    1    1   5
Phalaenopsis-gene156166               1    0    1    0    0   2
Phalaenopsis-gene156188               1    1    1    1    1   5
Phalaenopsis-gene156197               1    0    1    1    0   3
Phalaenopsis-gene1567                 1    0    0    0    0   1
Phalaenopsis-gene156935               1    0    1    0    0   2
Phalaenopsis-gene156951               1    1    1    1    1   5
Phalaenopsis-gene156983               1    1    0    1    0   3
Phalaenopsis-gene156993               1    1    1    1    1   5
Phalaenopsis-gene156994               0    0    1    1    0   2
Phalaenopsis-gene156995               1    1    1    1    1   5
Phalaenopsis-gene157423               0    0    1    0    0   1
Phalaenopsis-gene157424               1    1    1    1    1   5
Phalaenopsis-gene158193               1    1    1    1    1   5
Phalaenopsis-gene158875               0    0    0    1    0   1
Phalaenopsis-gene158920               0    0    0    0    0   0
Phalaenopsis-gene158921               1    1    0    1    0   3
Phalaenopsis-gene158985               1    1    0    0    0   2
Phalaenopsis-gene158986               1    1    0    1    0   3
Phalaenopsis-gene159004               1    1    0    1    0   3
Phalaenopsis-gene159135               1    1    1    0    0   3
Phalaenopsis-gene159201               1    0    0    0    0   1
Phalaenopsis-gene159219               1    0    1    1    0   3
Phalaenopsis-gene159302               1    0    1    1    0   3
Phalaenopsis-gene159562               1    1    0    0    0   2
Phalaenopsis-gene15965                0    1    1    0    0   2
Phalaenopsis-gene160570               1    1    1    1    1   5
Phalaenopsis-gene161505               1    0    1    0    0   2
Phalaenopsis-gene161506               0    1    1    0    0   2
Phalaenopsis-gene161834               1    0    1    1    0   3
Phalaenopsis-gene161835               0    0    1    0    0   1
Phalaenopsis-gene161837               1    1    1    1    1   5
Phalaenopsis-gene161844               0    0    0    0    0   0
Phalaenopsis-gene162030               1    0    0    1    0   2
Phalaenopsis-gene162057               1    0    0    1    0   2
Phalaenopsis-gene162058               0    0    0    0    0   0
Phalaenopsis-gene162105               1    0    1    0    0   2
Phalaenopsis-gene163034               1    0    1    1    0   3
Phalaenopsis-gene163035               1    0    0    1    0   2
Phalaenopsis-gene163069               0    0    0    1    0   1
Phalaenopsis-gene163096               0    0    0    0    0   0
Phalaenopsis-gene16323                1    0    1    1    0   3
Phalaenopsis-gene163540               1    1    1    1    1   5
Phalaenopsis-gene163757               1    0    1    1    0   3
Phalaenopsis-gene163805               0    0    0    0    0   0
Phalaenopsis-gene163806               1    1    1    1    1   5
Phalaenopsis-gene163829               1    1    1    1    1   5
Phalaenopsis-gene163855               1    1    1    1    1   5
Phalaenopsis-gene163856               1    1    1    1    1   5
Phalaenopsis-gene163860               1    1    1    1    1   5
Phalaenopsis-gene163940               1    0    0    1    0   2
Phalaenopsis-gene163945               1    0    1    1    0   3
Phalaenopsis-gene163948               0    0    1    1    0   2
Phalaenopsis-gene163949               0    0    0    1    0   1
Phalaenopsis-gene164050               1    1    1    1    1   5
Phalaenopsis-gene164225               1    0    1    1    0   3
Phalaenopsis-gene164226               1    1    1    1    1   5
Phalaenopsis-gene164227               1    0    0    1    0   2
Phalaenopsis-gene164616               1    1    1    1    1   5
Phalaenopsis-gene164617               0    0    0    1    0   1
Phalaenopsis-gene165022               0    0    1    0    0   1
Phalaenopsis-gene165023               1    1    0    0    1   3
Phalaenopsis-gene165105               1    0    1    1    0   3
Phalaenopsis-gene165122               1    0    1    1    0   3
Phalaenopsis-gene165123               1    0    0    0    0   1
Phalaenopsis-gene165221               1    0    1    1    0   3
Phalaenopsis-gene165222               1    0    0    0    0   1
Phalaenopsis-gene165476               1    1    1    0    0   3
Phalaenopsis-gene165609               1    0    1    0    0   2
Phalaenopsis-gene165617               1    1    1    1    1   5
Phalaenopsis-gene165642               0    1    0    1    0   2
Phalaenopsis-gene165678               1    0    1    1    0   3
Phalaenopsis-gene165679               1    0    1    1    0   3
Phalaenopsis-gene1657                 1    1    1    1    1   5
Phalaenopsis-gene165704               1    0    0    1    0   2
Phalaenopsis-gene1659                 1    0    1    1    0   3
Phalaenopsis-gene165979               1    1    1    1    1   5
Phalaenopsis-gene165991               0    0    0    1    1   2
Phalaenopsis-gene165992               1    0    0    1    0   2
Phalaenopsis-gene166522               1    1    1    1    1   5
Phalaenopsis-gene166523               0    0    1    1    0   2
Phalaenopsis-gene166905               1    0    0    0    0   1
Phalaenopsis-gene166906               1    1    1    1    1   5
Phalaenopsis-gene166990               0    0    1    0    0   1
Phalaenopsis-gene167055               1    0    1    0    0   2
Phalaenopsis-gene167240               0    0    1    1    0   2
Phalaenopsis-gene168009               1    1    1    1    1   5
Phalaenopsis-gene168520               1    0    1    1    0   3
Phalaenopsis-gene1689                 1    1    1    1    1   5
Phalaenopsis-gene168934               0    0    0    0    0   0
Phalaenopsis-gene168953               1    0    0    1    0   2
Phalaenopsis-gene169238               0    0    0    1    0   1
Phalaenopsis-gene169374               1    1    1    1    1   5
Phalaenopsis-gene1694                 1    1    1    1    1   5
Phalaenopsis-gene1695                 1    0    1    0    0   2
Phalaenopsis-gene169615               1    0    1    1    0   3
Phalaenopsis-gene169639               0    0    1    1    0   2
Phalaenopsis-gene169640               1    0    0    0    0   1
Phalaenopsis-gene169648               1    0    1    1    0   3
Phalaenopsis-gene169668               1    1    1    1    1   5
Phalaenopsis-gene169720               1    0    0    1    0   2
Phalaenopsis-gene169728               1    0    0    1    0   2
Phalaenopsis-gene169776               1    0    1    1    0   3
Phalaenopsis-gene169777               0    0    1    1    0   2
Phalaenopsis-gene169785               1    0    0    1    0   2
Phalaenopsis-gene170897               1    0    0    0    0   1
Phalaenopsis-gene170898               1    1    0    1    0   3
Phalaenopsis-gene170903               0    0    0    0    0   0
Phalaenopsis-gene170990               0    0    1    1    0   2
Phalaenopsis-gene171260               1    0    0    1    0   2
Phalaenopsis-gene171300               1    0    0    0    0   1
Phalaenopsis-gene171338               1    0    0    1    0   2
Phalaenopsis-gene17184                1    1    1    1    1   5
Phalaenopsis-gene171956               0    0    1    1    0   2
Phalaenopsis-gene171991               1    1    1    0    0   3
Phalaenopsis-gene172016               0    0    1    1    0   2
Phalaenopsis-gene172022               1    0    1    1    0   3
Phalaenopsis-gene172077               1    1    1    1    1   5
Phalaenopsis-gene172100               1    1    1    1    0   4
Phalaenopsis-gene172110               1    0    0    1    0   2
Phalaenopsis-gene172208               1    0    0    1    1   3
Phalaenopsis-gene172215               1    1    1    1    1   5
Phalaenopsis-gene173413               1    1    1    1    1   5
Phalaenopsis-gene173596               1    1    1    1    1   5
Phalaenopsis-gene173653               1    1    1    1    1   5
Phalaenopsis-gene173654               1    1    0    1    0   3
Phalaenopsis-gene173802               0    0    0    1    0   1
Phalaenopsis-gene173905               1    0    0    1    0   2
Phalaenopsis-gene173912               1    0    0    1    0   2
Phalaenopsis-gene173925               1    0    0    0    0   1
Phalaenopsis-gene173928               1    1    1    1    1   5
Phalaenopsis-gene174058               1    0    0    1    0   2
Phalaenopsis-gene174071               1    0    0    1    0   2
Phalaenopsis-gene174074               1    0    1    1    0   3
Phalaenopsis-gene174107               1    0    0    0    0   1
Phalaenopsis-gene174124               1    0    0    0    0   1
Phalaenopsis-gene174127               1    0    0    1    0   2
Phalaenopsis-gene176314               1    0    1    0    0   2
Phalaenopsis-gene177208               1    0    1    1    0   3
Phalaenopsis-gene177219               1    1    0    1    0   3
Phalaenopsis-gene177250               1    1    1    0    0   3
Phalaenopsis-gene177261               1    1    0    1    0   3
Phalaenopsis-gene17835                0    0    0    1    0   1
Phalaenopsis-gene17879                1    0    1    1    0   3
Phalaenopsis-gene178888               1    0    0    1    0   2
Phalaenopsis-gene178898               1    1    1    1    1   5
Phalaenopsis-gene178925               1    0    0    1    0   2
Phalaenopsis-gene178935               1    1    1    1    1   5
Phalaenopsis-gene17949                1    0    0    1    0   2
Phalaenopsis-gene17950                1    0    1    1    0   3
Phalaenopsis-gene1799                 1    0    0    1    0   2
Phalaenopsis-gene181370               1    0    0    0    0   1
Phalaenopsis-gene181557               1    1    1    1    1   5
Phalaenopsis-gene18210                1    0    0    1    0   2
Phalaenopsis-gene18211                1    1    1    1    1   5
Phalaenopsis-gene18223                1    1    1    1    1   5
Phalaenopsis-gene183073               1    0    1    1    0   3
Phalaenopsis-gene183079               1    0    1    0    0   2
Phalaenopsis-gene184379               1    0    0    1    0   2
Phalaenopsis-gene184416               1    1    1    1    1   5
Phalaenopsis-gene184559               1    0    0    1    0   2
Phalaenopsis-gene184633               0    0    0    0    0   0
Phalaenopsis-gene184662               1    0    1    0    0   2
Phalaenopsis-gene186314               1    1    1    1    1   5
Phalaenopsis-gene186847               0    0    0    0    0   0
Phalaenopsis-gene186857               0    0    1    1    0   2
Phalaenopsis-gene186858               1    0    0    1    0   2
Phalaenopsis-gene186897               0    0    0    1    0   1
Phalaenopsis-gene186908               1    1    0    0    0   2
Phalaenopsis-gene18709                1    0    0    0    0   1
Phalaenopsis-gene188594               1    0    0    0    0   1
Phalaenopsis-gene188608               1    0    1    1    0   3
Phalaenopsis-gene188742               1    0    0    0    0   1
Phalaenopsis-gene188770               1    0    1    1    0   3
Phalaenopsis-gene188832               1    0    1    0    0   2
Phalaenopsis-gene188833               0    0    0    0    0   0
Phalaenopsis-gene188887               1    1    1    1    1   5
Phalaenopsis-gene188888               1    0    1    1    0   3
Phalaenopsis-gene188938               1    0    0    1    0   2
Phalaenopsis-gene189011               1    1    1    1    1   5
Phalaenopsis-gene189069               1    0    1    1    0   3
Phalaenopsis-gene189079               1    1    1    1    1   5
Phalaenopsis-gene189080               1    0    1    1    0   3
Phalaenopsis-gene189116               1    1    1    1    1   5
Phalaenopsis-gene189126               0    0    1    0    0   1
Phalaenopsis-gene189135               1    0    1    1    0   3
Phalaenopsis-gene189254               1    0    0    0    0   1
Phalaenopsis-gene190006               1    0    0    1    0   2
Phalaenopsis-gene190007               1    1    1    1    1   5
Phalaenopsis-gene190045               1    0    1    1    1   4
Phalaenopsis-gene190046               1    1    1    1    1   5
Phalaenopsis-gene190055               1    0    1    0    0   2
Phalaenopsis-gene190956               1    1    1    1    1   5
Phalaenopsis-gene190973               1    1    1    1    1   5
Phalaenopsis-gene191929               0    0    1    1    0   2
Phalaenopsis-gene19209                0    0    1    1    0   2
Phalaenopsis-gene192492               1    1    1    1    1   5
Phalaenopsis-gene192493               1    0    1    0    0   2
Phalaenopsis-gene19251                1    0    1    1    0   3
Phalaenopsis-gene19252                1    0    0    1    0   2
Phalaenopsis-gene19293                1    0    0    1    0   2
Phalaenopsis-gene19294                0    0    0    1    0   1
Phalaenopsis-gene193088               1    1    1    1    1   5
Phalaenopsis-gene193147               1    1    1    1    1   5
Phalaenopsis-gene19336                1    1    1    1    1   5
Phalaenopsis-gene19337                1    0    0    1    0   2
Phalaenopsis-gene194396               1    0    1    1    0   3
Phalaenopsis-gene194397               1    0    1    0    0   2
Phalaenopsis-gene194432               1    1    1    1    1   5
Phalaenopsis-gene194616               0    1    0    1    0   2
Phalaenopsis-gene194623               0    0    1    0    0   1
Phalaenopsis-gene194899               1    1    0    1    0   3
Phalaenopsis-gene195126               0    0    0    1    0   1
Phalaenopsis-gene195130               1    0    1    1    0   3
Phalaenopsis-gene197871               1    0    1    1    0   3
Phalaenopsis-gene197886               1    0    1    1    0   3
Phalaenopsis-gene197894               1    0    1    1    0   3
Phalaenopsis-gene197899               1    0    0    1    0   2
Phalaenopsis-gene197911               1    1    1    1    1   5
Phalaenopsis-gene197912               1    0    1    1    0   3
Phalaenopsis-gene197927               1    0    0    1    0   2
Phalaenopsis-gene197940               0    0    1    0    0   1
Phalaenopsis-gene197941               1    1    1    1    1   5
Phalaenopsis-gene198615               1    0    1    1    0   3
Phalaenopsis-gene198685               1    1    1    0    0   3
Phalaenopsis-gene199067               1    0    1    1    0   3
Phalaenopsis-gene199402               0    0    1    1    0   2
Phalaenopsis-gene199504               1    1    0    1    0   3
Phalaenopsis-gene199637               1    1    1    1    1   5
Phalaenopsis-gene199738               1    0    1    1    1   4
Phalaenopsis-gene199739               1    1    1    1    1   5
Phalaenopsis-gene199801               1    1    0    1    0   3
Phalaenopsis-gene199802               1    1    1    1    0   4
Phalaenopsis-gene199864               1    0    0    1    0   2
Phalaenopsis-gene199865               1    0    1    1    0   3
Phalaenopsis-gene2                    1    1    1    1    1   5
Phalaenopsis-gene201115               1    0    0    0    0   1
Phalaenopsis-gene201116               1    1    1    1    0   4
Phalaenopsis-gene201122               1    0    0    1    0   2
Phalaenopsis-gene201560               1    0    0    0    0   1
Phalaenopsis-gene201568               1    0    0    1    0   2
Phalaenopsis-gene201591               1    1    1    1    1   5
Phalaenopsis-gene201593               1    0    0    1    0   2
Phalaenopsis-gene202309               1    0    0    1    0   2
Phalaenopsis-gene202319               1    1    1    1    1   5
Phalaenopsis-gene202394               1    1    1    1    1   5
Phalaenopsis-gene202404               1    0    0    1    0   2
Phalaenopsis-gene202405               0    0    1    1    0   2
Phalaenopsis-gene202407               0    0    0    0    0   0
Phalaenopsis-gene202417               1    1    1    1    1   5
Phalaenopsis-gene202850               1    0    0    1    0   2
Phalaenopsis-gene203067               0    0    0    0    0   0
Phalaenopsis-gene203068               1    0    0    0    0   1
Phalaenopsis-gene203096               0    0    1    1    0   2
Phalaenopsis-gene203180               0    0    1    1    0   2
Phalaenopsis-gene203741               1    0    1    1    0   3
Phalaenopsis-gene203782               1    0    0    1    0   2
Phalaenopsis-gene203784               1    1    1    1    1   5
Phalaenopsis-gene203804               1    0    0    1    0   2
Phalaenopsis-gene203837               0    0    0    1    0   1
Phalaenopsis-gene203839               1    0    1    0    0   2
Phalaenopsis-gene203859               1    0    1    0    0   2
Phalaenopsis-gene204316               1    1    0    1    0   3
Phalaenopsis-gene204350               1    0    1    0    0   2
Phalaenopsis-gene204456               1    1    1    1    1   5
Phalaenopsis-gene204482               0    0    1    1    0   2
Phalaenopsis-gene204483               1    0    1    1    0   3
Phalaenopsis-gene204502               1    0    0    1    0   2
Phalaenopsis-gene204583               1    0    1    1    0   3
Phalaenopsis-gene204584               0    0    1    0    0   1
Phalaenopsis-gene2046                 1    1    1    1    1   5
Phalaenopsis-gene204603               1    1    1    1    0   4
Phalaenopsis-gene205944               0    0    1    0    0   1
Phalaenopsis-gene205949               1    0    0    1    0   2
Phalaenopsis-gene205956               1    1    1    1    1   5
Phalaenopsis-gene205957               0    0    1    0    0   1
Phalaenopsis-gene206120               0    1    1    1    0   3
Phalaenopsis-gene208566               1    0    1    1    0   3
Phalaenopsis-gene208567               1    0    0    1    0   2
Phalaenopsis-gene208587               1    0    0    1    1   3
Phalaenopsis-gene209127               1    1    1    1    1   5
Phalaenopsis-gene209140               1    0    1    1    0   3
Phalaenopsis-gene209141               0    0    1    0    0   1
Phalaenopsis-gene209173               1    0    1    1    0   3
Phalaenopsis-gene209180               0    1    0    1    0   2
Phalaenopsis-gene209197               1    0    0    1    0   2
Phalaenopsis-gene209216               1    0    0    1    0   2
Phalaenopsis-gene209251               1    1    1    1    1   5
Phalaenopsis-gene209268               0    0    0    1    0   1
Phalaenopsis-gene209288               1    0    0    1    0   2
Phalaenopsis-gene209328               1    0    0    1    0   2
Phalaenopsis-gene209430               1    0    1    1    0   3
Phalaenopsis-gene209431               1    1    1    1    0   4
Phalaenopsis-gene209450               0    1    1    0    0   2
Phalaenopsis-gene209451               1    1    1    1    1   5
Phalaenopsis-gene209609               1    1    1    1    1   5
Phalaenopsis-gene209618               0    0    1    1    0   2
Phalaenopsis-gene209736               0    0    1    1    0   2
Phalaenopsis-gene209865               0    0    1    1    0   2
Phalaenopsis-gene21088                1    0    1    1    0   3
Phalaenopsis-gene211375               1    0    1    1    0   3
Phalaenopsis-gene21140                0    0    1    1    0   2
Phalaenopsis-gene211561               1    0    1    1    0   3
Phalaenopsis-gene211562               1    0    1    0    0   2
Phalaenopsis-gene211683               1    1    1    1    1   5
Phalaenopsis-gene212307               1    0    0    0    0   1
Phalaenopsis-gene212392               1    0    0    1    0   2
Phalaenopsis-gene212434               1    0    1    0    0   2
Phalaenopsis-gene212435               1    1    1    1    1   5
Phalaenopsis-gene212468               1    0    0    1    0   2
Phalaenopsis-gene212469               0    0    1    1    1   3
Phalaenopsis-gene212470               1    0    1    1    0   3
Phalaenopsis-gene213333               0    0    1    1    0   2
Phalaenopsis-gene213338               1    1    1    1    1   5
Phalaenopsis-gene213575               1    0    0    0    0   1
Phalaenopsis-gene214102               1    0    0    0    0   1
Phalaenopsis-gene214496               1    0    1    1    0   3
Phalaenopsis-gene214644               0    1    1    0    0   2
Phalaenopsis-gene214654               1    0    1    0    0   2
Phalaenopsis-gene214660               0    0    1    0    0   1
Phalaenopsis-gene214661               0    0    0    1    0   1
Phalaenopsis-gene214671               1    0    0    1    0   2
Phalaenopsis-gene214672               1    0    0    1    0   2
Phalaenopsis-gene214688               1    0    1    1    0   3
Phalaenopsis-gene214704               1    0    1    1    0   3
Phalaenopsis-gene214705               1    0    1    1    0   3
Phalaenopsis-gene214716               1    0    0    1    0   2
Phalaenopsis-gene214743               0    1    0    1    0   2
Phalaenopsis-gene214749               0    0    1    1    0   2
Phalaenopsis-gene214760               1    0    0    1    0   2
Phalaenopsis-gene214761               1    0    0    1    0   2
Phalaenopsis-gene214851               0    0    0    1    0   1
Phalaenopsis-gene214954               1    0    0    1    0   2
Phalaenopsis-gene215654               1    1    1    1    1   5
Phalaenopsis-gene215674               0    1    0    0    0   1
Phalaenopsis-gene216026               1    0    1    1    0   3
Phalaenopsis-gene216040               1    0    1    0    0   2
Phalaenopsis-gene216676               0    0    0    1    0   1
Phalaenopsis-gene216677               1    0    1    1    0   3
Phalaenopsis-gene216853               1    0    0    1    0   2
Phalaenopsis-gene216862               1    1    0    0    0   2
Phalaenopsis-gene216867               1    0    0    1    0   2
Phalaenopsis-gene216878               0    0    1    1    0   2
Phalaenopsis-gene216879               1    1    1    1    0   4
Phalaenopsis-gene216902               0    0    1    1    0   2
Phalaenopsis-gene21791                1    1    1    1    1   5
Phalaenopsis-gene217976               0    0    1    0    0   1
Phalaenopsis-gene217977               0    0    0    0    0   0
Phalaenopsis-gene218044               1    1    1    1    1   5
Phalaenopsis-gene2185                 0    0    0    0    1   1
Phalaenopsis-gene2186                 1    0    0    1    0   2
Phalaenopsis-gene21866                1    0    1    1    0   3
Phalaenopsis-gene218948               0    0    1    0    0   1
Phalaenopsis-gene21944                1    0    1    1    0   3
Phalaenopsis-gene219608               1    1    1    1    1   5
Phalaenopsis-gene219609               0    0    0    1    0   1
Phalaenopsis-gene219877               1    1    1    1    1   5
Phalaenopsis-gene219878               1    0    1    1    0   3
Phalaenopsis-gene219902               0    0    1    1    0   2
Phalaenopsis-gene219903               1    1    1    1    0   4
Phalaenopsis-gene220340               0    0    0    1    1   2
Phalaenopsis-gene220373               1    0    0    0    0   1
Phalaenopsis-gene220406               1    0    0    1    0   2
Phalaenopsis-gene220407               1    1    1    1    1   5
Phalaenopsis-gene221725               1    1    1    1    1   5
Phalaenopsis-gene222019               0    0    0    0    0   0
Phalaenopsis-gene222051               1    1    1    1    0   4
Phalaenopsis-gene222076               1    1    1    1    1   5
Phalaenopsis-gene222206               1    1    1    1    1   5
Phalaenopsis-gene222212               1    0    1    1    0   3
Phalaenopsis-gene222213               1    1    1    1    1   5
Phalaenopsis-gene222236               1    1    1    1    0   4
Phalaenopsis-gene222244               0    0    0    1    0   1
Phalaenopsis-gene222255               0    0    1    1    0   2
Phalaenopsis-gene222330               1    0    1    1    0   3
Phalaenopsis-gene222331               1    0    0    1    0   2
Phalaenopsis-gene222839               1    1    1    1    1   5
Phalaenopsis-gene222840               1    0    1    1    0   3
Phalaenopsis-gene222966               1    0    0    0    0   1
Phalaenopsis-gene222967               0    0    1    1    0   2
Phalaenopsis-gene223025               1    1    1    1    1   5
Phalaenopsis-gene223380               1    1    1    1    1   5
Phalaenopsis-gene223381               0    0    1    1    0   2
Phalaenopsis-gene223508               1    1    1    1    1   5
Phalaenopsis-gene223546               1    0    1    1    0   3
Phalaenopsis-gene223549               1    1    1    1    1   5
Phalaenopsis-gene223550               0    0    0    1    0   1
Phalaenopsis-gene223551               1    1    1    1    1   5
Phalaenopsis-gene223617               1    1    1    1    1   5
Phalaenopsis-gene223618               1    1    1    1    1   5
Phalaenopsis-gene223768               1    1    1    1    1   5
Phalaenopsis-gene223790               1    1    1    1    1   5
Phalaenopsis-gene223791               1    0    1    1    0   3
Phalaenopsis-gene223828               0    0    1    0    0   1
Phalaenopsis-gene223909               1    0    0    1    0   2
Phalaenopsis-gene224000               1    0    0    1    1   3
Phalaenopsis-gene224028               1    0    0    1    0   2
Phalaenopsis-gene22444                1    0    1    1    0   3
Phalaenopsis-gene225290               1    1    1    1    1   5
Phalaenopsis-gene22565                1    1    1    1    1   5
Phalaenopsis-gene22566                1    1    1    1    1   5
Phalaenopsis-gene225690               0    0    1    1    0   2
Phalaenopsis-gene225726               1    1    1    1    1   5
Phalaenopsis-gene225754               0    0    1    1    0   2
Phalaenopsis-gene226392               1    0    0    1    0   2
Phalaenopsis-gene226741               1    1    1    1    1   5
Phalaenopsis-gene22709                1    1    1    1    1   5
Phalaenopsis-gene227640               1    0    1    1    0   3
Phalaenopsis-gene228214               1    0    0    0    0   1
Phalaenopsis-gene228229               1    1    0    0    0   2
Phalaenopsis-gene228717               0    0    0    1    0   1
Phalaenopsis-gene228719               1    0    0    0    1   2
Phalaenopsis-gene228720               0    0    0    1    0   1
Phalaenopsis-gene229237               1    1    1    1    1   5
Phalaenopsis-gene229628               1    1    1    1    1   5
Phalaenopsis-gene22965                1    1    1    1    1   5
Phalaenopsis-gene229869               1    0    1    1    0   3
Phalaenopsis-gene23029                1    1    1    1    1   5
Phalaenopsis-gene230788               1    1    1    1    0   4
Phalaenopsis-gene231256               1    1    1    1    1   5
Phalaenopsis-gene231258               0    0    0    0    0   0
Phalaenopsis-gene231261               1    1    1    1    1   5
Phalaenopsis-gene231262               1    1    1    1    1   5
Phalaenopsis-gene231364               1    1    1    1    1   5
Phalaenopsis-gene232060               0    0    0    1    0   1
Phalaenopsis-gene232085               1    0    1    0    0   2
Phalaenopsis-gene232323               1    1    1    1    1   5
Phalaenopsis-gene232325               1    0    1    0    0   2
Phalaenopsis-gene23268                1    0    1    0    0   2
Phalaenopsis-gene232755               1    1    1    1    1   5
Phalaenopsis-gene232840               0    1    1    1    0   3
Phalaenopsis-gene232871               1    1    1    1    0   4
Phalaenopsis-gene23288                1    1    1    1    1   5
Phalaenopsis-gene233155               1    0    1    1    0   3
Phalaenopsis-gene233204               0    1    1    1    0   3
Phalaenopsis-gene233332               1    0    0    1    0   2
Phalaenopsis-gene234002               1    0    1    1    1   4
Phalaenopsis-gene23432                1    1    1    1    1   5
Phalaenopsis-gene23465                1    0    0    1    0   2
Phalaenopsis-gene234767               0    1    0    1    0   2
Phalaenopsis-gene234768               0    0    0    1    0   1
Phalaenopsis-gene234880               1    0    0    0    0   1
Phalaenopsis-gene234881               0    0    1    0    0   1
Phalaenopsis-gene234964               1    0    1    0    0   2
Phalaenopsis-gene234976               0    0    1    0    0   1
Phalaenopsis-gene234977               0    0    0    0    0   0
Phalaenopsis-gene234984               1    1    1    1    1   5
Phalaenopsis-gene23506                1    1    1    1    1   5
Phalaenopsis-gene23509                1    1    1    1    1   5
Phalaenopsis-gene235470               1    1    1    1    1   5
Phalaenopsis-gene235471               1    0    0    0    0   1
Phalaenopsis-gene236249               1    0    0    1    0   2
Phalaenopsis-gene236250               1    1    1    1    1   5
Phalaenopsis-gene236293               0    0    0    1    1   2
Phalaenopsis-gene236294               1    1    1    1    1   5
Phalaenopsis-gene2367                 1    1    1    1    1   5
Phalaenopsis-gene237768               1    0    1    0    0   2
Phalaenopsis-gene237825               0    0    0    1    0   1
Phalaenopsis-gene237826               1    1    1    1    1   5
Phalaenopsis-gene23833                1    1    1    1    1   5
Phalaenopsis-gene238426               1    0    0    1    0   2
Phalaenopsis-gene238507               1    0    1    1    0   3
Phalaenopsis-gene238508               1    1    1    1    1   5
Phalaenopsis-gene239091               1    0    1    1    0   3
Phalaenopsis-gene239092               1    0    1    1    0   3
Phalaenopsis-gene239613               0    0    1    0    0   1
Phalaenopsis-gene240943               1    0    0    1    1   3
Phalaenopsis-gene241285               1    1    1    0    0   3
Phalaenopsis-gene241289               1    1    1    1    1   5
Phalaenopsis-gene242185               1    1    1    1    1   5
Phalaenopsis-gene242186               1    0    1    1    0   3
Phalaenopsis-gene242376               0    0    0    0    0   0
Phalaenopsis-gene242543               1    1    1    1    1   5
Phalaenopsis-gene242890               1    1    1    1    1   5
Phalaenopsis-gene244872               1    1    1    0    0   3
Phalaenopsis-gene244874               1    0    1    1    0   3
Phalaenopsis-gene245492               1    1    1    1    0   4
Phalaenopsis-gene245493               1    1    1    1    1   5
Phalaenopsis-gene245507               0    0    0    1    0   1
Phalaenopsis-gene245534               1    0    0    1    0   2
Phalaenopsis-gene245585               0    0    0    0    0   0
Phalaenopsis-gene247185               0    0    1    1    0   2
Phalaenopsis-gene247188               0    0    1    0    0   1
Phalaenopsis-gene247189               0    0    0    0    0   0
Phalaenopsis-gene247197               0    0    0    1    0   1
Phalaenopsis-gene247203               1    0    1    1    0   3
Phalaenopsis-gene247212               1    0    1    1    1   4
Phalaenopsis-gene247213               1    0    1    0    0   2
Phalaenopsis-gene247224               1    0    1    0    0   2
Phalaenopsis-gene247226               1    0    0    0    0   1
Phalaenopsis-gene247227               1    0    0    1    0   2
Phalaenopsis-gene247253               1    0    0    0    0   1
Phalaenopsis-gene247256               1    0    1    1    0   3
Phalaenopsis-gene247265               0    0    0    0    0   0
Phalaenopsis-gene247270               1    1    1    1    1   5
Phalaenopsis-gene247280               1    0    0    1    0   2
Phalaenopsis-gene247292               1    0    0    1    1   3
Phalaenopsis-gene247294               0    0    1    1    0   2
Phalaenopsis-gene247314               1    1    1    0    0   3
Phalaenopsis-gene247315               0    0    0    1    0   1
Phalaenopsis-gene247674               1    1    1    1    1   5
Phalaenopsis-gene247676               0    0    1    1    0   2
Phalaenopsis-gene247699               1    1    1    1    1   5
Phalaenopsis-gene247701               1    0    1    1    0   3
Phalaenopsis-gene247720               1    0    0    0    0   1
Phalaenopsis-gene247762               1    1    1    1    1   5
Phalaenopsis-gene248114               1    1    1    1    1   5
Phalaenopsis-gene248115               0    0    1    1    0   2
Phalaenopsis-gene249111               1    0    1    1    0   3
Phalaenopsis-gene24936                1    1    1    1    1   5
Phalaenopsis-gene24940                1    0    0    1    0   2
Phalaenopsis-gene24943                1    0    1    0    0   2
Phalaenopsis-gene24966                1    0    0    0    0   1
Phalaenopsis-gene24967                1    0    1    0    0   2
Phalaenopsis-gene25008                1    0    0    1    0   2
Phalaenopsis-gene250492               1    0    1    0    0   2
Phalaenopsis-gene250543               1    0    0    0    0   1
Phalaenopsis-gene250611               1    0    1    0    0   2
Phalaenopsis-gene250758               0    0    0    0    0   0
Phalaenopsis-gene250759               1    1    1    1    1   5
Phalaenopsis-gene250940               1    1    1    1    1   5
Phalaenopsis-gene251140               1    0    0    1    0   2
Phalaenopsis-gene251141               1    0    1    0    0   2
Phalaenopsis-gene251578               0    0    0    1    0   1
Phalaenopsis-gene251911               1    1    1    1    1   5
Phalaenopsis-gene251912               1    0    0    1    0   2
Phalaenopsis-gene251923               1    0    1    1    0   3
Phalaenopsis-gene252201               1    0    0    1    0   2
Phalaenopsis-gene252337               1    0    1    0    0   2
Phalaenopsis-gene252338               1    1    1    1    1   5
Phalaenopsis-gene252360               1    0    1    1    0   3
Phalaenopsis-gene252361               0    0    1    0    0   1
Phalaenopsis-gene252701               0    0    0    1    0   1
Phalaenopsis-gene252702               1    0    0    1    0   2
Phalaenopsis-gene25281                1    1    1    1    1   5
Phalaenopsis-gene25341                1    0    0    0    0   1
Phalaenopsis-gene25342                1    1    1    1    1   5
Phalaenopsis-gene253802               1    0    1    1    0   3
Phalaenopsis-gene253834               1    1    1    1    1   5
Phalaenopsis-gene25423                1    0    0    1    0   2
Phalaenopsis-gene25424                1    0    1    1    0   3
Phalaenopsis-gene254394               1    1    1    1    1   5
Phalaenopsis-gene254395               1    0    1    1    0   3
Phalaenopsis-gene254405               1    0    1    1    0   3
Phalaenopsis-gene254653               1    0    1    0    0   2
Phalaenopsis-gene254679               0    0    1    1    0   2
Phalaenopsis-gene254706               0    0    0    0    0   0
Phalaenopsis-gene254712               1    0    1    0    0   2
Phalaenopsis-gene25484                0    1    1    1    0   3
Phalaenopsis-gene255168               1    0    1    1    0   3
Phalaenopsis-gene255183               1    0    0    0    0   1
Phalaenopsis-gene255256               0    0    1    0    0   1
Phalaenopsis-gene255269               1    0    0    1    0   2
Phalaenopsis-gene255271               1    1    1    1    1   5
Phalaenopsis-gene255398               0    0    1    1    0   2
Phalaenopsis-gene25560                1    0    0    1    0   2
Phalaenopsis-gene255771               1    1    1    1    1   5
Phalaenopsis-gene25584                1    1    1    1    1   5
Phalaenopsis-gene255933               1    0    1    1    0   3
Phalaenopsis-gene255934               0    0    0    0    0   0
Phalaenopsis-gene256061               1    0    0    0    0   1
Phalaenopsis-gene256074               1    0    1    1    0   3
Phalaenopsis-gene256154               1    1    1    1    1   5
Phalaenopsis-gene256158               1    1    1    1    1   5
Phalaenopsis-gene256159               0    0    1    0    1   2
Phalaenopsis-gene25631                1    0    0    1    0   2
Phalaenopsis-gene256455               1    0    0    0    0   1
Phalaenopsis-gene256462               1    0    1    0    0   2
Phalaenopsis-gene256512               1    0    0    1    0   2
Phalaenopsis-gene256518               1    1    1    1    1   5
Phalaenopsis-gene256519               0    1    0    1    0   2
Phalaenopsis-gene256520               1    0    0    1    0   2
Phalaenopsis-gene25663                1    0    1    0    0   2
Phalaenopsis-gene25690                1    1    0    1    0   3
Phalaenopsis-gene257403               1    0    1    1    0   3
Phalaenopsis-gene257537               0    0    1    0    0   1
Phalaenopsis-gene257547               1    0    1    1    0   3
Phalaenopsis-gene257548               1    0    1    1    0   3
Phalaenopsis-gene25796                1    1    1    1    0   4
Phalaenopsis-gene258420               1    1    1    1    1   5
Phalaenopsis-gene258576               1    0    0    0    0   1
Phalaenopsis-gene258754               1    1    1    1    1   5
Phalaenopsis-gene258764               1    0    0    1    0   2
Phalaenopsis-gene258779               1    0    1    1    0   3
Phalaenopsis-gene258782               1    1    1    1    1   5
Phalaenopsis-gene259030               1    0    0    0    0   1
Phalaenopsis-gene259934               1    0    0    0    0   1
Phalaenopsis-gene259948               0    0    1    1    0   2
Phalaenopsis-gene259966               0    0    1    1    0   2
Phalaenopsis-gene260506               1    1    1    1    1   5
Phalaenopsis-gene260507               1    0    0    1    0   2
Phalaenopsis-gene260511               1    1    1    1    1   5
Phalaenopsis-gene260515               1    0    0    0    0   1
Phalaenopsis-gene261015               0    0    0    1    0   1
Phalaenopsis-gene262                  1    0    1    1    0   3
Phalaenopsis-gene262410               0    0    0    1    0   1
Phalaenopsis-gene262411               1    0    0    1    0   2
Phalaenopsis-gene262473               1    0    0    1    0   2
Phalaenopsis-gene262474               1    0    0    0    0   1
Phalaenopsis-gene262575               0    0    1    0    0   1
Phalaenopsis-gene262576               1    1    1    1    1   5
Phalaenopsis-gene262598               1    0    1    0    0   2
Phalaenopsis-gene262599               0    0    1    0    0   1
Phalaenopsis-gene262617               1    1    1    1    1   5
Phalaenopsis-gene263                  0    0    0    0    0   0
Phalaenopsis-gene264                  1    0    0    1    0   2
Phalaenopsis-gene264433               0    0    0    0    0   0
Phalaenopsis-gene264538               1    0    0    1    1   3
Phalaenopsis-gene265208               0    1    1    1    0   3
Phalaenopsis-gene265686               1    1    1    1    1   5
Phalaenopsis-gene265694               1    1    1    1    1   5
Phalaenopsis-gene266943               0    0    0    1    0   1
Phalaenopsis-gene267049               0    0    1    1    0   2
Phalaenopsis-gene267052               0    0    1    1    0   2
Phalaenopsis-gene267413               1    1    1    1    1   5
Phalaenopsis-gene267773               1    1    1    1    1   5
Phalaenopsis-gene26933                1    1    1    1    1   5
Phalaenopsis-gene26934                1    1    1    1    1   5
Phalaenopsis-gene269457               1    1    1    1    1   5
Phalaenopsis-gene269494               1    0    1    0    0   2
Phalaenopsis-gene269528               1    1    1    1    1   5
Phalaenopsis-gene270032               1    0    1    1    1   4
Phalaenopsis-gene270172               1    1    1    1    1   5
Phalaenopsis-gene270177               1    0    1    1    0   3
Phalaenopsis-gene270194               1    1    1    1    1   5
Phalaenopsis-gene27061                1    1    1    1    1   5
Phalaenopsis-gene27068                0    0    1    1    0   2
Phalaenopsis-gene27069                1    1    1    1    1   5
Phalaenopsis-gene272538               0    0    0    1    0   1
Phalaenopsis-gene272539               1    0    0    1    0   2
Phalaenopsis-gene273172               1    1    1    0    0   3
Phalaenopsis-gene273263               1    1    1    1    1   5
Phalaenopsis-gene273293               1    1    1    1    1   5
Phalaenopsis-gene273447               1    0    1    1    0   3
Phalaenopsis-gene273920               1    1    1    1    1   5
Phalaenopsis-gene274102               1    0    1    0    0   2
Phalaenopsis-gene274109               1    0    1    0    0   2
Phalaenopsis-gene274158               0    0    1    0    0   1
Phalaenopsis-gene274162               0    1    0    0    0   1
Phalaenopsis-gene274261               1    0    1    1    0   3
Phalaenopsis-gene27491                1    0    0    1    0   2
Phalaenopsis-gene27561                1    1    1    1    1   5
Phalaenopsis-gene27562                0    0    0    1    0   1
Phalaenopsis-gene27661                1    1    1    1    1   5
Phalaenopsis-gene276806               0    0    0    1    0   1
Phalaenopsis-gene276819               0    1    0    1    0   2
Phalaenopsis-gene276869               1    0    1    1    0   3
Phalaenopsis-gene278406               1    1    1    1    1   5
Phalaenopsis-gene278657               1    1    1    1    1   5
Phalaenopsis-gene279624               0    0    1    1    0   2
Phalaenopsis-gene279625               0    0    1    0    0   1
Phalaenopsis-gene279769               0    0    1    0    0   1
Phalaenopsis-gene279778               1    1    1    1    1   5
Phalaenopsis-gene279788               1    0    1    1    0   3
Phalaenopsis-gene280052               1    0    1    0    1   3
Phalaenopsis-gene280710               1    0    0    1    0   2
Phalaenopsis-gene280711               0    0    1    0    0   1
Phalaenopsis-gene280876               1    0    0    1    0   2
Phalaenopsis-gene281417               1    0    1    1    0   3
Phalaenopsis-gene281429               0    0    0    1    0   1
Phalaenopsis-gene281459               1    0    1    0    0   2
Phalaenopsis-gene281482               1    0    0    1    0   2
Phalaenopsis-gene281591               1    0    0    0    0   1
Phalaenopsis-gene281592               1    1    1    1    1   5
Phalaenopsis-gene281596               1    1    1    1    1   5
Phalaenopsis-gene281892               1    0    0    1    0   2
Phalaenopsis-gene281893               0    0    1    0    0   1
Phalaenopsis-gene282138               1    0    1    1    0   3
Phalaenopsis-gene282139               1    1    1    1    1   5
Phalaenopsis-gene282152               1    0    1    1    0   3
Phalaenopsis-gene282153               0    0    0    0    0   0
Phalaenopsis-gene282218               1    0    1    1    0   3
Phalaenopsis-gene282231               0    0    1    0    0   1
Phalaenopsis-gene282287               1    0    0    1    0   2
Phalaenopsis-gene283679               0    1    1    1    0   3
Phalaenopsis-gene28414                1    0    1    1    0   3
Phalaenopsis-gene28415                1    1    1    1    1   5
Phalaenopsis-gene284155               1    0    1    1    0   3
Phalaenopsis-gene284156               1    0    0    1    0   2
Phalaenopsis-gene28432                0    0    1    0    0   1
Phalaenopsis-gene28443                1    1    1    1    1   5
Phalaenopsis-gene284710               1    1    1    1    1   5
Phalaenopsis-gene284711               1    0    1    1    0   3
Phalaenopsis-gene28483                1    1    1    0    0   3
Phalaenopsis-gene28500                1    0    1    1    0   3
Phalaenopsis-gene28501                0    1    0    1    0   2
Phalaenopsis-gene28511                1    1    1    1    1   5
Phalaenopsis-gene28516                1    1    1    1    1   5
Phalaenopsis-gene285360               0    0    1    0    0   1
Phalaenopsis-gene28552                1    0    1    1    0   3
Phalaenopsis-gene285571               1    0    0    1    0   2
Phalaenopsis-gene28569                0    0    0    0    0   0
Phalaenopsis-gene28570                0    0    1    0    0   1
Phalaenopsis-gene28580                0    0    1    0    0   1
Phalaenopsis-gene285821               1    1    1    1    1   5
Phalaenopsis-gene28585                1    0    1    1    0   3
Phalaenopsis-gene28615                0    0    1    1    0   2
Phalaenopsis-gene28632                1    0    1    1    0   3
Phalaenopsis-gene28633                1    0    1    0    0   2
Phalaenopsis-gene286477               1    0    0    0    0   1
Phalaenopsis-gene28648                1    1    1    1    1   5
Phalaenopsis-gene286532               1    1    1    1    1   5
Phalaenopsis-gene286533               1    1    1    1    1   5
Phalaenopsis-gene286590               0    1    1    1    0   3
Phalaenopsis-gene286642               1    0    1    1    0   3
Phalaenopsis-gene28677                1    1    1    1    1   5
Phalaenopsis-gene28678                1    1    1    1    1   5
Phalaenopsis-gene28679                1    1    1    1    1   5
Phalaenopsis-gene28695                1    0    1    0    0   2
Phalaenopsis-gene287202               1    1    1    1    1   5
Phalaenopsis-gene287203               1    1    1    1    0   4
Phalaenopsis-gene287286               1    0    1    0    0   2
Phalaenopsis-gene287295               0    0    1    0    0   1
Phalaenopsis-gene287327               1    1    1    1    1   5
Phalaenopsis-gene287390               1    0    0    0    0   1
Phalaenopsis-gene287421               1    0    0    1    0   2
Phalaenopsis-gene287442               0    0    0    1    0   1
Phalaenopsis-gene287443               0    0    1    1    0   2
Phalaenopsis-gene287832               1    1    1    1    1   5
Phalaenopsis-gene288456               1    1    1    1    1   5
Phalaenopsis-gene288457               1    1    1    1    1   5
Phalaenopsis-gene289077               0    0    1    1    0   2
Phalaenopsis-gene289089               1    1    1    1    1   5
Phalaenopsis-gene289090               1    1    1    1    1   5
Phalaenopsis-gene289303               1    0    0    1    0   2
Phalaenopsis-gene289311               0    0    0    0    0   0
Phalaenopsis-gene289321               1    0    0    1    0   2
Phalaenopsis-gene289795               0    0    0    1    0   1
Phalaenopsis-gene290153               0    0    1    1    0   2
Phalaenopsis-gene290162               1    1    1    1    1   5
Phalaenopsis-gene290167               1    1    1    1    1   5
Phalaenopsis-gene290523               0    0    1    1    0   2
Phalaenopsis-gene290586               1    1    1    1    1   5
Phalaenopsis-gene290950               1    1    1    1    1   5
Phalaenopsis-gene291302               1    1    1    1    1   5
Phalaenopsis-gene291305               1    0    0    1    0   2
Phalaenopsis-gene291369               1    0    1    1    0   3
Phalaenopsis-gene291424               1    0    0    0    0   1
Phalaenopsis-gene291477               1    0    0    1    0   2
Phalaenopsis-gene291718               1    0    1    1    0   3
Phalaenopsis-gene292573               0    0    1    1    0   2
Phalaenopsis-gene292574               1    1    1    1    1   5
Phalaenopsis-gene293040               1    1    1    1    1   5
Phalaenopsis-gene293387               1    1    1    1    1   5
Phalaenopsis-gene293543               0    0    0    1    0   1
Phalaenopsis-gene293605               1    0    1    0    0   2
Phalaenopsis-gene293638               1    0    0    0    0   1
Phalaenopsis-gene293667               0    1    0    1    0   2
Phalaenopsis-gene293668               1    0    0    0    0   1
Phalaenopsis-gene294814               1    0    0    1    0   2
Phalaenopsis-gene294871               1    0    0    0    0   1
Phalaenopsis-gene294988               1    0    1    0    0   2
Phalaenopsis-gene296604               1    0    1    1    0   3
Phalaenopsis-gene297288               1    0    0    0    0   1
Phalaenopsis-gene297289               1    1    1    1    1   5
Phalaenopsis-gene297296               1    1    0    0    0   2
Phalaenopsis-gene298346               1    1    1    0    0   3
Phalaenopsis-gene298347               1    0    1    0    0   2
Phalaenopsis-gene298409               1    0    0    1    0   2
Phalaenopsis-gene298412               0    0    1    0    0   1
Phalaenopsis-gene298728               0    0    1    0    0   1
Phalaenopsis-gene299287               1    0    1    0    0   2
Phalaenopsis-gene299705               1    0    1    1    0   3
Phalaenopsis-gene299782               0    0    0    1    0   1
Phalaenopsis-gene3                    1    1    1    1    1   5
Phalaenopsis-gene300763               1    0    0    1    0   2
Phalaenopsis-gene300764               1    0    1    1    0   3
Phalaenopsis-gene300853               1    1    1    1    1   5
Phalaenopsis-gene300854               1    0    0    1    0   2
Phalaenopsis-gene300879               1    0    0    0    0   1
Phalaenopsis-gene301608               1    0    0    0    0   1
Phalaenopsis-gene301862               1    0    1    1    0   3
Phalaenopsis-gene301868               0    0    1    0    0   1
Phalaenopsis-gene302372               1    1    1    1    1   5
Phalaenopsis-gene302373               0    0    0    0    0   0
Phalaenopsis-gene302388               0    0    1    1    0   2
Phalaenopsis-gene302389               1    0    0    1    0   2
Phalaenopsis-gene302391               0    0    1    1    0   2
Phalaenopsis-gene302399               0    0    1    1    0   2
Phalaenopsis-gene302400               1    0    1    1    0   3
Phalaenopsis-gene302468               1    0    1    1    0   3
Phalaenopsis-gene302479               1    1    1    1    1   5
Phalaenopsis-gene302660               0    0    1    1    0   2
Phalaenopsis-gene302820               1    0    1    1    0   3
Phalaenopsis-gene302947               1    0    1    0    0   2
Phalaenopsis-gene304728               1    1    1    1    1   5
Phalaenopsis-gene305765               0    0    1    0    0   1
Phalaenopsis-gene306650               1    1    1    1    1   5
Phalaenopsis-gene306651               1    0    0    1    0   2
Phalaenopsis-gene306656               0    0    0    0    0   0
Phalaenopsis-gene306771               0    0    0    0    0   0
Phalaenopsis-gene306999               1    0    0    0    0   1
Phalaenopsis-gene307000               1    0    0    1    0   2
Phalaenopsis-gene307066               1    0    0    1    0   2
Phalaenopsis-gene307189               1    0    1    1    1   4
Phalaenopsis-gene307227               0    0    1    0    0   1
Phalaenopsis-gene307261               1    0    0    1    0   2
Phalaenopsis-gene307385               1    0    0    1    0   2
Phalaenopsis-gene307386               1    0    0    0    0   1
Phalaenopsis-gene307476               1    0    0    0    0   1
Phalaenopsis-gene307510               1    0    0    1    0   2
Phalaenopsis-gene307511               1    0    0    0    0   1
Phalaenopsis-gene307601               1    0    1    1    0   3
Phalaenopsis-gene307635               1    0    0    0    0   1
Phalaenopsis-gene307636               1    0    0    0    0   1
Phalaenopsis-gene307725               1    0    0    1    0   2
Phalaenopsis-gene307730               1    0    0    0    0   1
Phalaenopsis-gene307736               0    0    1    1    0   2
Phalaenopsis-gene307759               1    0    0    0    0   1
Phalaenopsis-gene307849               1    0    1    0    1   3
Phalaenopsis-gene307883               0    0    0    0    0   0
Phalaenopsis-gene308045               1    1    1    1    0   4
Phalaenopsis-gene308726               1    1    1    1    1   5
Phalaenopsis-gene308833               1    1    1    0    0   3
Phalaenopsis-gene308894               1    1    1    1    0   4
Phalaenopsis-gene309047               1    0    0    1    0   2
Phalaenopsis-gene309048               0    0    0    1    0   1
Phalaenopsis-gene309108               1    0    0    0    0   1
Phalaenopsis-gene309109               1    0    0    1    0   2
Phalaenopsis-gene309146               0    0    1    1    0   2
Phalaenopsis-gene309156               0    1    0    0    0   1
Phalaenopsis-gene309157               1    0    0    1    0   2
Phalaenopsis-gene309194               1    0    1    0    1   3
Phalaenopsis-gene309204               0    0    0    1    0   1
Phalaenopsis-gene309668               1    1    1    1    1   5
Phalaenopsis-gene309908               1    1    1    1    1   5
Phalaenopsis-gene309938               1    0    1    1    0   3
Phalaenopsis-gene309939               0    0    1    0    0   1
Phalaenopsis-gene310109               1    0    1    1    1   4
Phalaenopsis-gene310138               1    1    1    0    0   3
Phalaenopsis-gene310139               1    0    1    0    1   3
Phalaenopsis-gene310222               1    1    1    1    1   5
Phalaenopsis-gene310223               0    0    1    1    0   2
Phalaenopsis-gene310226               1    1    1    1    1   5
Phalaenopsis-gene310444               1    0    1    1    0   3
Phalaenopsis-gene310452               1    1    1    1    1   5
Phalaenopsis-gene310813               1    1    1    1    1   5
Phalaenopsis-gene311268               1    0    0    1    0   2
Phalaenopsis-gene311269               1    0    1    0    0   2
Phalaenopsis-gene311327               1    0    0    0    0   1
Phalaenopsis-gene311328               1    0    1    1    0   3
Phalaenopsis-gene311354               0    1    1    1    0   3
Phalaenopsis-gene311355               0    0    0    1    0   1
Phalaenopsis-gene311367               1    0    1    1    0   3
Phalaenopsis-gene311372               1    1    1    1    1   5
Phalaenopsis-gene311373               0    0    0    0    0   0
Phalaenopsis-gene311672               1    1    1    1    1   5
Phalaenopsis-gene311673               1    1    1    1    1   5
Phalaenopsis-gene311674               1    1    1    1    0   4
Phalaenopsis-gene311675               1    1    1    1    1   5
Phalaenopsis-gene311683               0    0    0    0    0   0
Phalaenopsis-gene312147               1    1    1    1    1   5
Phalaenopsis-gene312198               0    0    0    1    0   1
Phalaenopsis-gene312206               1    0    0    0    0   1
Phalaenopsis-gene312601               1    0    0    0    0   1
Phalaenopsis-gene312689               0    0    1    0    0   1
Phalaenopsis-gene312690               1    1    1    1    1   5
Phalaenopsis-gene314817               1    0    0    1    0   2
Phalaenopsis-gene317767               0    0    0    1    0   1
Phalaenopsis-gene317822               0    0    1    1    0   2
Phalaenopsis-gene318135               1    1    1    1    1   5
Phalaenopsis-gene318229               1    0    1    1    0   3
Phalaenopsis-gene318335               1    0    0    0    0   1
Phalaenopsis-gene318509               1    1    1    1    1   5
Phalaenopsis-gene318510               1    1    1    1    1   5
Phalaenopsis-gene318712               1    0    0    1    0   2
Phalaenopsis-gene318756               1    1    1    1    1   5
Phalaenopsis-gene318798               1    0    1    1    0   3
Phalaenopsis-gene318821               1    0    0    1    0   2
Phalaenopsis-gene319226               0    0    0    1    0   1
Phalaenopsis-gene319227               1    1    0    0    0   2
Phalaenopsis-gene319241               1    0    0    1    0   2
Phalaenopsis-gene319242               1    1    1    0    0   3
Phalaenopsis-gene319323               1    0    0    1    0   2
Phalaenopsis-gene319656               0    0    0    1    0   1
Phalaenopsis-gene319657               1    0    1    1    0   3
Phalaenopsis-gene319671               1    0    1    1    0   3
Phalaenopsis-gene319680               1    1    1    1    1   5
Phalaenopsis-gene319681               0    1    1    1    0   3
Phalaenopsis-gene319714               1    0    1    1    0   3
Phalaenopsis-gene319720               1    0    1    1    1   4
Phalaenopsis-gene319734               1    0    0    1    0   2
Phalaenopsis-gene319802               1    1    1    1    1   5
Phalaenopsis-gene319816               1    1    0    0    0   2
Phalaenopsis-gene319817               1    0    0    1    0   2
Phalaenopsis-gene319839               1    1    1    1    0   4
Phalaenopsis-gene319845               1    1    1    1    0   4
Phalaenopsis-gene319859               1    0    0    1    0   2
Phalaenopsis-gene320074               1    0    0    0    0   1
Phalaenopsis-gene320075               1    0    1    0    0   2
Phalaenopsis-gene320146               1    0    0    1    0   2
Phalaenopsis-gene320157               0    0    0    1    0   1
Phalaenopsis-gene320158               1    1    1    1    1   5
Phalaenopsis-gene320226               1    1    1    1    0   4
Phalaenopsis-gene320746               1    1    1    1    0   4
Phalaenopsis-gene320877               0    0    1    1    0   2
Phalaenopsis-gene320884               1    0    1    1    0   3
Phalaenopsis-gene321159               1    0    0    1    0   2
Phalaenopsis-gene32119                1    0    0    0    0   1
Phalaenopsis-gene321496               1    0    1    1    0   3
Phalaenopsis-gene321937               1    0    0    1    0   2
Phalaenopsis-gene321938               1    0    0    0    0   1
Phalaenopsis-gene321993               1    1    1    1    1   5
Phalaenopsis-gene322280               1    1    1    1    1   5
Phalaenopsis-gene322284               1    0    1    1    0   3
Phalaenopsis-gene322322               1    0    0    0    0   1
Phalaenopsis-gene322326               1    0    1    1    0   3
Phalaenopsis-gene32233                1    1    1    1    1   5
Phalaenopsis-gene32234                0    0    1    0    0   1
Phalaenopsis-gene322356               1    0    0    1    0   2
Phalaenopsis-gene322442               1    0    1    0    0   2
Phalaenopsis-gene322512               1    0    0    1    0   2
Phalaenopsis-gene322516               1    0    1    0    0   2
Phalaenopsis-gene322835               1    1    1    1    1   5
Phalaenopsis-gene322849               0    0    0    0    0   0
Phalaenopsis-gene322855               1    1    1    1    1   5
Phalaenopsis-gene322856               0    1    0    1    0   2
Phalaenopsis-gene322866               1    0    1    1    0   3
Phalaenopsis-gene322868               1    1    1    1    1   5
Phalaenopsis-gene322869               0    0    1    1    0   2
Phalaenopsis-gene322913               1    0    1    1    0   3
Phalaenopsis-gene322914               1    1    1    1    1   5
Phalaenopsis-gene322916               1    0    1    1    0   3
Phalaenopsis-gene32300                0    0    1    1    0   2
Phalaenopsis-gene32301                0    0    1    1    0   2
Phalaenopsis-gene324327               1    0    1    1    0   3
Phalaenopsis-gene32449                1    1    1    1    1   5
Phalaenopsis-gene32450                1    0    0    1    0   2
Phalaenopsis-gene32454                1    1    1    1    1   5
Phalaenopsis-gene324550               1    0    1    1    0   3
Phalaenopsis-gene324608               0    1    1    1    0   3
Phalaenopsis-gene324622               1    1    1    1    1   5
Phalaenopsis-gene324633               1    1    1    1    1   5
Phalaenopsis-gene325293               1    0    0    1    0   2
Phalaenopsis-gene325295               1    1    0    1    0   3
Phalaenopsis-gene325318               1    0    0    1    0   2
Phalaenopsis-gene325560               1    0    0    0    0   1
Phalaenopsis-gene326036               1    0    1    1    1   4
Phalaenopsis-gene326396               1    0    0    1    0   2
Phalaenopsis-gene326474               1    1    1    1    1   5
Phalaenopsis-gene326488               1    0    0    1    0   2
Phalaenopsis-gene326490               0    0    1    0    0   1
Phalaenopsis-gene326637               1    0    1    1    0   3
Phalaenopsis-gene326648               1    1    1    1    1   5
Phalaenopsis-gene326651               1    0    1    1    0   3
Phalaenopsis-gene326653               1    1    1    1    1   5
Phalaenopsis-gene326901               0    0    1    0    0   1
Phalaenopsis-gene327245               1    1    1    1    1   5
Phalaenopsis-gene331600               1    1    1    1    1   5
Phalaenopsis-gene331617               1    1    0    1    0   3
Phalaenopsis-gene331618               1    0    0    0    0   1
Phalaenopsis-gene331638               0    0    1    1    0   2
Phalaenopsis-gene332160               1    0    1    1    0   3
Phalaenopsis-gene332161               1    1    1    1    1   5
Phalaenopsis-gene332786               1    1    0    1    0   3
Phalaenopsis-gene332837               1    1    1    1    1   5
Phalaenopsis-gene332865               1    0    0    1    0   2
Phalaenopsis-gene332918               1    0    0    1    0   2
Phalaenopsis-gene332919               1    1    0    0    0   2
Phalaenopsis-gene333246               1    0    0    1    0   2
Phalaenopsis-gene33372                1    0    1    1    0   3
Phalaenopsis-gene333731               1    0    1    0    1   3
Phalaenopsis-gene333750               1    0    1    1    0   3
Phalaenopsis-gene333978               0    0    0    1    0   1
Phalaenopsis-gene334323               1    0    0    0    0   1
Phalaenopsis-gene334324               1    0    0    0    0   1
Phalaenopsis-gene334801               1    1    0    0    0   2
Phalaenopsis-gene334918               0    0    0    1    0   1
Phalaenopsis-gene334919               1    1    0    1    0   3
Phalaenopsis-gene335037               0    0    1    1    0   2
Phalaenopsis-gene335038               0    1    1    1    0   3
Phalaenopsis-gene335150               1    0    0    1    0   2
Phalaenopsis-gene335151               0    0    0    1    0   1
Phalaenopsis-gene335899               1    1    1    1    1   5
Phalaenopsis-gene335911               1    0    0    1    0   2
Phalaenopsis-gene335912               0    1    0    0    0   1
Phalaenopsis-gene335915               1    1    1    1    1   5
Phalaenopsis-gene335933               1    0    1    0    0   2
Phalaenopsis-gene335940               1    1    1    1    0   4
Phalaenopsis-gene337381               1    0    1    0    0   2
Phalaenopsis-gene337737               1    1    1    1    1   5
Phalaenopsis-gene337856               1    0    1    1    0   3
Phalaenopsis-gene337857               1    0    1    1    0   3
Phalaenopsis-gene338568               1    0    1    0    0   2
Phalaenopsis-gene338583               1    0    1    0    0   2
Phalaenopsis-gene338586               1    0    0    0    0   1
Phalaenopsis-gene338626               0    1    1    1    0   3
Phalaenopsis-gene338627               1    1    1    1    1   5
Phalaenopsis-gene339330               0    1    0    1    0   2
Phalaenopsis-gene339399               1    1    0    1    0   3
Phalaenopsis-gene339626               0    0    1    0    0   1
Phalaenopsis-gene339677               1    0    1    1    0   3
Phalaenopsis-gene339731               0    0    0    0    0   0
Phalaenopsis-gene339919               1    0    1    1    0   3
Phalaenopsis-gene339943               1    0    1    1    0   3
Phalaenopsis-gene340023               1    0    0    0    0   1
Phalaenopsis-gene340026               0    0    1    1    0   2
Phalaenopsis-gene340027               1    1    1    1    0   4
Phalaenopsis-gene340035               1    0    0    1    0   2
Phalaenopsis-gene340079               0    0    1    1    0   2
Phalaenopsis-gene340087               1    1    1    1    1   5
Phalaenopsis-gene340093               1    1    1    1    1   5
Phalaenopsis-gene340228               1    0    1    0    1   3
Phalaenopsis-gene340229               1    0    1    1    0   3
Phalaenopsis-gene340248               1    0    1    1    0   3
Phalaenopsis-gene340280               1    1    0    1    0   3
Phalaenopsis-gene340282               1    0    1    1    0   3
Phalaenopsis-gene340313               1    0    0    1    0   2
Phalaenopsis-gene340327               1    0    0    0    0   1
Phalaenopsis-gene340501               1    0    0    1    0   2
Phalaenopsis-gene340586               1    0    1    0    0   2
Phalaenopsis-gene340622               1    0    1    1    0   3
Phalaenopsis-gene341100               1    1    1    1    1   5
Phalaenopsis-gene341257               1    1    1    1    1   5
Phalaenopsis-gene341430               1    1    1    1    1   5
Phalaenopsis-gene341623               1    1    1    1    1   5
Phalaenopsis-gene341640               1    1    1    1    0   4
Phalaenopsis-gene341719               0    0    0    1    0   1
Phalaenopsis-gene341993               1    1    1    1    1   5
Phalaenopsis-gene342020               1    0    1    1    0   3
Phalaenopsis-gene342226               1    1    1    1    1   5
Phalaenopsis-gene342284               1    0    0    1    0   2
Phalaenopsis-gene342313               0    0    1    1    0   2
Phalaenopsis-gene342824               0    0    1    1    0   2
Phalaenopsis-gene342885               1    0    1    1    0   3
Phalaenopsis-gene342890               1    1    1    1    1   5
Phalaenopsis-gene34304                1    1    1    1    1   5
Phalaenopsis-gene34307                0    0    0    1    0   1
Phalaenopsis-gene34313                1    0    1    1    0   3
Phalaenopsis-gene34319                1    1    1    1    1   5
Phalaenopsis-gene343280               1    1    1    1    1   5
Phalaenopsis-gene343318               1    1    1    1    1   5
Phalaenopsis-gene343345               1    0    1    0    0   2
Phalaenopsis-gene343346               0    0    0    1    0   1
Phalaenopsis-gene343397               1    0    1    1    0   3
Phalaenopsis-gene343398               1    0    0    1    0   2
Phalaenopsis-gene343520               1    0    1    1    1   4
Phalaenopsis-gene343559               0    0    1    1    0   2
Phalaenopsis-gene343561               1    0    1    1    0   3
Phalaenopsis-gene343565               1    0    0    0    0   1
Phalaenopsis-gene343586               1    0    1    1    0   3
Phalaenopsis-gene343587               1    0    1    0    0   2
Phalaenopsis-gene343860               1    1    1    1    1   5
Phalaenopsis-gene343861               1    0    0    1    0   2
Phalaenopsis-gene343867               1    0    0    1    0   2
Phalaenopsis-gene343887               1    0    1    1    0   3
Phalaenopsis-gene343888               1    0    1    1    0   3
Phalaenopsis-gene343928               1    0    1    1    0   3
Phalaenopsis-gene343929               0    0    1    1    0   2
Phalaenopsis-gene344581               1    1    0    0    0   2
Phalaenopsis-gene344827               0    0    1    1    0   2
Phalaenopsis-gene345471               1    0    0    1    0   2
Phalaenopsis-gene345472               1    0    1    1    0   3
Phalaenopsis-gene345542               0    0    1    0    0   1
Phalaenopsis-gene345543               1    0    0    1    0   2
Phalaenopsis-gene345549               1    0    1    1    0   3
Phalaenopsis-gene345938               1    0    1    1    1   4
Phalaenopsis-gene346416               1    1    1    1    1   5
Phalaenopsis-gene347198               1    1    0    1    0   3
Phalaenopsis-gene34720                1    0    1    0    0   2
Phalaenopsis-gene347298               1    1    1    1    1   5
Phalaenopsis-gene347360               0    0    1    1    0   2
Phalaenopsis-gene34748                1    0    0    1    0   2
Phalaenopsis-gene347888               1    0    0    1    0   2
Phalaenopsis-gene347901               1    0    1    1    0   3
Phalaenopsis-gene348435               1    0    0    1    0   2
Phalaenopsis-gene348467               1    0    0    1    0   2
Phalaenopsis-gene348500               1    0    0    1    0   2
Phalaenopsis-gene348547               0    0    0    1    0   1
Phalaenopsis-gene348691               0    0    1    0    1   2
Phalaenopsis-gene348713               1    0    1    0    0   2
Phalaenopsis-gene348750               1    0    1    1    0   3
Phalaenopsis-gene348791               1    1    1    1    1   5
Phalaenopsis-gene348799               1    1    1    1    1   5
Phalaenopsis-gene348800               1    1    1    0    1   4
Phalaenopsis-gene348989               1    1    1    1    1   5
Phalaenopsis-gene349077               1    1    1    1    1   5
Phalaenopsis-gene349084               1    1    1    1    1   5
Phalaenopsis-gene349217               1    0    1    0    0   2
Phalaenopsis-gene349369               1    1    1    1    1   5
Phalaenopsis-gene349370               1    0    0    0    0   1
Phalaenopsis-gene349452               1    0    0    0    0   1
Phalaenopsis-gene349470               1    0    0    0    0   1
Phalaenopsis-gene349490               1    0    0    0    0   1
Phalaenopsis-gene349491               1    0    0    1    0   2
Phalaenopsis-gene349534               0    0    0    1    0   1
Phalaenopsis-gene349535               1    0    0    0    0   1
Phalaenopsis-gene349577               1    0    0    1    0   2
Phalaenopsis-gene349587               1    0    1    1    0   3
Phalaenopsis-gene34961                0    0    0    1    0   1
Phalaenopsis-gene34962                1    1    1    0    0   3
Phalaenopsis-gene349627               1    0    0    1    0   2
Phalaenopsis-gene34963                0    0    0    0    0   0
Phalaenopsis-gene349638               1    0    0    0    0   1
Phalaenopsis-gene349671               1    1    1    1    1   5
Phalaenopsis-gene349681               1    1    0    0    0   2
Phalaenopsis-gene349809               1    0    0    0    0   1
Phalaenopsis-gene350445               1    1    1    1    1   5
Phalaenopsis-gene350456               1    0    0    0    0   1
Phalaenopsis-gene350544               0    0    1    0    0   1
Phalaenopsis-gene350692               1    0    1    0    0   2
Phalaenopsis-gene350780               1    1    1    1    1   5
Phalaenopsis-gene350932               0    0    1    1    0   2
Phalaenopsis-gene350940               1    1    1    1    1   5
Phalaenopsis-gene351001               1    1    1    1    1   5
Phalaenopsis-gene351188               1    0    0    1    0   2
Phalaenopsis-gene351200               1    1    0    1    0   3
Phalaenopsis-gene351255               0    0    1    1    0   2
Phalaenopsis-gene351268               1    0    1    0    0   2
Phalaenopsis-gene351281               0    0    1    1    0   2
Phalaenopsis-gene351328               0    0    0    1    0   1
Phalaenopsis-gene351341               0    0    0    1    0   1
Phalaenopsis-gene351354               1    0    1    1    0   3
Phalaenopsis-gene351363               1    0    0    1    0   2
Phalaenopsis-gene351364               0    0    0    1    0   1
Phalaenopsis-gene351416               1    0    1    0    0   2
Phalaenopsis-gene351438               1    0    0    0    0   1
Phalaenopsis-gene351462               1    1    1    0    0   3
Phalaenopsis-gene351464               1    0    0    1    0   2
Phalaenopsis-gene351475               1    1    1    1    1   5
Phalaenopsis-gene351488               1    0    1    1    0   3
Phalaenopsis-gene351497               0    1    0    1    0   2
Phalaenopsis-gene351601               0    0    1    0    0   1
Phalaenopsis-gene351641               0    1    1    1    0   3
Phalaenopsis-gene351856               1    0    0    1    0   2
Phalaenopsis-gene351869               1    1    1    1    1   5
Phalaenopsis-gene351874               1    1    1    0    1   4
Phalaenopsis-gene351889               0    0    0    0    0   0
Phalaenopsis-gene351959               1    0    1    0    0   2
Phalaenopsis-gene351960               1    1    1    1    1   5
Phalaenopsis-gene352006               1    0    1    1    0   3
Phalaenopsis-gene352052               1    0    1    1    0   3
Phalaenopsis-gene352098               1    1    1    1    1   5
Phalaenopsis-gene352146               1    1    1    0    0   3
Phalaenopsis-gene352147               1    1    1    1    1   5
Phalaenopsis-gene353046               1    0    1    1    0   3
Phalaenopsis-gene354255               0    1    1    1    0   3
Phalaenopsis-gene354714               1    0    1    1    0   3
Phalaenopsis-gene354758               0    1    1    1    0   3
Phalaenopsis-gene354769               1    0    0    0    0   1
Phalaenopsis-gene354859               1    0    0    0    0   1
Phalaenopsis-gene354904               1    1    1    1    1   5
Phalaenopsis-gene354906               1    1    1    1    1   5
Phalaenopsis-gene354907               1    0    0    1    0   2
Phalaenopsis-gene354919               0    0    0    1    0   1
Phalaenopsis-gene355021               1    0    0    1    0   2
Phalaenopsis-gene355127               1    0    1    1    0   3
Phalaenopsis-gene355129               1    1    1    0    0   3
Phalaenopsis-gene355130               1    0    1    1    1   4
Phalaenopsis-gene355131               0    0    1    1    0   2
Phalaenopsis-gene355222               0    0    1    0    0   1
Phalaenopsis-gene355223               0    0    0    1    0   1
Phalaenopsis-gene355225               1    0    0    0    0   1
Phalaenopsis-gene355252               1    0    1    0    0   2
Phalaenopsis-gene355253               1    0    1    0    0   2
Phalaenopsis-gene355254               1    1    1    0    0   3
Phalaenopsis-gene355284               1    0    1    1    0   3
Phalaenopsis-gene355285               0    0    1    1    0   2
Phalaenopsis-gene355287               1    0    1    0    0   2
Phalaenopsis-gene355318               1    0    1    1    0   3
Phalaenopsis-gene355319               1    0    1    1    0   3
Phalaenopsis-gene355339               1    0    1    0    0   2
Phalaenopsis-gene355341               1    0    1    0    0   2
Phalaenopsis-gene355342               1    1    1    1    1   5
Phalaenopsis-gene355372               1    0    0    1    0   2
Phalaenopsis-gene355375               1    0    0    1    0   2
Phalaenopsis-gene35539                1    1    1    1    1   5
Phalaenopsis-gene355405               1    1    1    1    1   5
Phalaenopsis-gene355442               1    1    0    1    0   3
Phalaenopsis-gene355475               1    1    1    1    1   5
Phalaenopsis-gene356194               1    0    0    1    0   2
Phalaenopsis-gene356195               1    0    0    1    0   2
Phalaenopsis-gene356214               1    1    1    1    1   5
Phalaenopsis-gene356222               0    0    0    1    0   1
Phalaenopsis-gene356223               1    0    0    0    0   1
Phalaenopsis-gene356393               0    1    1    1    0   3
Phalaenopsis-gene357780               0    1    1    1    0   3
Phalaenopsis-gene357781               0    0    0    0    0   0
Phalaenopsis-gene357989               1    1    1    1    1   5
Phalaenopsis-gene358042               1    0    1    1    0   3
Phalaenopsis-gene358043               1    0    1    0    0   2
Phalaenopsis-gene36093                0    0    0    1    0   1
Phalaenopsis-gene36094                1    0    0    0    0   1
Phalaenopsis-gene361008               1    0    1    1    0   3
Phalaenopsis-gene361476               0    0    0    1    0   1
Phalaenopsis-gene361508               1    0    1    0    0   2
Phalaenopsis-gene361575               0    0    0    1    0   1
Phalaenopsis-gene361576               0    0    0    0    0   0
Phalaenopsis-gene361605               1    1    1    1    1   5
Phalaenopsis-gene361635               0    0    0    1    0   1
Phalaenopsis-gene361636               1    0    1    0    0   2
Phalaenopsis-gene361663               1    0    0    1    0   2
Phalaenopsis-gene361664               1    1    1    1    1   5
Phalaenopsis-gene361682               1    0    0    0    0   1
Phalaenopsis-gene361695               0    1    0    1    0   2
Phalaenopsis-gene361696               1    1    1    1    1   5
Phalaenopsis-gene361722               1    0    0    1    0   2
Phalaenopsis-gene361723               0    1    0    0    0   1
Phalaenopsis-gene361751               0    0    0    1    0   1
Phalaenopsis-gene361781               0    0    0    1    0   1
Phalaenopsis-gene361782               1    1    1    1    1   5
Phalaenopsis-gene361797               0    0    0    1    0   1
Phalaenopsis-gene361810               1    1    0    0    0   2
Phalaenopsis-gene361811               1    0    1    1    0   3
Phalaenopsis-gene361838               0    0    0    1    0   1
Phalaenopsis-gene361839               0    0    0    1    0   1
Phalaenopsis-gene362905               1    0    1    0    0   2
Phalaenopsis-gene362908               1    0    1    1    0   3
Phalaenopsis-gene363225               1    0    1    0    0   2
Phalaenopsis-gene363228               1    0    0    1    0   2
Phalaenopsis-gene363233               1    0    0    1    0   2
Phalaenopsis-gene363283               0    0    1    1    0   2
Phalaenopsis-gene363284               0    0    0    1    0   1
Phalaenopsis-gene363286               1    0    0    0    0   1
Phalaenopsis-gene363290               1    1    1    1    1   5
Phalaenopsis-gene36337                1    1    1    1    0   4
Phalaenopsis-gene36338                1    0    0    1    0   2
Phalaenopsis-gene36349                1    0    0    0    0   1
Phalaenopsis-gene364323               0    0    0    0    0   0
Phalaenopsis-gene364346               1    0    0    1    0   2
Phalaenopsis-gene364348               1    0    0    1    0   2
Phalaenopsis-gene364390               0    0    0    1    0   1
Phalaenopsis-gene364423               1    1    1    1    1   5
Phalaenopsis-gene364891               1    0    0    1    0   2
Phalaenopsis-gene364930               1    0    1    1    0   3
Phalaenopsis-gene364951               0    0    0    1    0   1
Phalaenopsis-gene364952               1    1    1    1    1   5
Phalaenopsis-gene364964               1    0    1    1    0   3
Phalaenopsis-gene364970               0    0    0    1    0   1
Phalaenopsis-gene364986               1    0    0    1    0   2
Phalaenopsis-gene364999               1    1    1    1    1   5
Phalaenopsis-gene365006               1    1    1    1    1   5
Phalaenopsis-gene36545                1    1    1    0    0   3
Phalaenopsis-gene36584                1    0    0    1    0   2
Phalaenopsis-gene36593                1    0    0    0    0   1
Phalaenopsis-gene36617                0    0    1    1    0   2
Phalaenopsis-gene36642                1    0    0    1    0   2
Phalaenopsis-gene36643                1    0    1    1    0   3
Phalaenopsis-gene36646                0    0    0    1    0   1
Phalaenopsis-gene36654                1    1    1    1    0   4
Phalaenopsis-gene36663                1    0    0    1    0   2
Phalaenopsis-gene36688                1    0    1    1    0   3
Phalaenopsis-gene36715                0    0    1    1    0   2
Phalaenopsis-gene36727                0    0    0    0    0   0
Phalaenopsis-gene36736                1    0    0    1    0   2
Phalaenopsis-gene36763                1    1    1    0    0   3
Phalaenopsis-gene367767               1    0    1    0    0   2
Phalaenopsis-gene367768               0    0    0    1    0   1
Phalaenopsis-gene36788                1    0    0    1    0   2
Phalaenopsis-gene36789                1    0    1    1    0   3
Phalaenopsis-gene36792                1    0    0    1    0   2
Phalaenopsis-gene36800                1    1    1    1    1   5
Phalaenopsis-gene36809                1    0    1    1    0   3
Phalaenopsis-gene368629               0    0    1    1    0   2
Phalaenopsis-gene36863                1    0    0    1    0   2
Phalaenopsis-gene368630               1    1    1    1    1   5
Phalaenopsis-gene36864                1    1    1    1    1   5
Phalaenopsis-gene36867                0    0    0    1    0   1
Phalaenopsis-gene36875                0    0    0    1    0   1
Phalaenopsis-gene36884                0    0    1    0    0   1
Phalaenopsis-gene36939                1    0    0    1    0   2
Phalaenopsis-gene36940                1    1    1    1    1   5
Phalaenopsis-gene369417               1    1    1    1    1   5
Phalaenopsis-gene369487               1    0    1    1    0   3
Phalaenopsis-gene369557               1    0    1    1    0   3
Phalaenopsis-gene369625               1    1    1    1    0   4
Phalaenopsis-gene369829               1    1    1    1    1   5
Phalaenopsis-gene370214               1    1    0    1    0   3
Phalaenopsis-gene370851               1    0    1    1    0   3
Phalaenopsis-gene370855               1    0    1    1    1   4
Phalaenopsis-gene370867               0    0    0    0    0   0
Phalaenopsis-gene370880               1    1    1    1    0   4
Phalaenopsis-gene370902               0    0    1    1    0   2
Phalaenopsis-gene370918               1    1    1    1    1   5
Phalaenopsis-gene370934               1    0    0    1    0   2
Phalaenopsis-gene370941               1    1    1    1    1   5
Phalaenopsis-gene370974               1    1    1    1    1   5
Phalaenopsis-gene370990               1    0    0    1    0   2
Phalaenopsis-gene370994               1    0    1    1    0   3
Phalaenopsis-gene371006               1    0    0    1    0   2
Phalaenopsis-gene371019               0    1    1    0    0   2
Phalaenopsis-gene371046               1    1    1    1    1   5
Phalaenopsis-gene371405               1    0    1    1    0   3
Phalaenopsis-gene372129               1    1    1    1    1   5
Phalaenopsis-gene372165               1    1    1    1    1   5
Phalaenopsis-gene372168               1    0    0    1    0   2
Phalaenopsis-gene372210               0    0    1    1    0   2
Phalaenopsis-gene374238               1    0    1    1    0   3
Phalaenopsis-gene374340               0    0    1    1    0   2
Phalaenopsis-gene374341               1    0    1    1    0   3
Phalaenopsis-gene374402               1    0    1    0    0   2
Phalaenopsis-gene374403               1    1    1    1    1   5
Phalaenopsis-gene374612               1    1    1    1    1   5
Phalaenopsis-gene374924               0    0    0    1    0   1
Phalaenopsis-gene374989               0    0    1    1    0   2
Phalaenopsis-gene375041               1    1    1    0    0   3
Phalaenopsis-gene375140               0    0    1    1    0   2
Phalaenopsis-gene375264               1    0    0    1    0   2
Phalaenopsis-gene375442               1    0    1    0    0   2
Phalaenopsis-gene375463               0    0    1    1    0   2
Phalaenopsis-gene375657               1    1    1    1    0   4
Phalaenopsis-gene376076               0    0    1    0    0   1
Phalaenopsis-gene376099               1    1    1    1    1   5
Phalaenopsis-gene376118               0    0    1    0    0   1
Phalaenopsis-gene377318               0    0    1    1    0   2
Phalaenopsis-gene377319               1    0    0    1    0   2
Phalaenopsis-gene377336               1    0    0    1    0   2
Phalaenopsis-gene377355               1    0    1    1    0   3
Phalaenopsis-gene377789               0    0    0    1    0   1
Phalaenopsis-gene377827               1    0    1    0    1   3
Phalaenopsis-gene377828               1    1    0    0    0   2
Phalaenopsis-gene377943               1    1    1    1    1   5
Phalaenopsis-gene378063               1    1    1    1    1   5
Phalaenopsis-gene378078               1    0    1    1    0   3
Phalaenopsis-gene378106               1    1    1    1    1   5
Phalaenopsis-gene378143               0    0    1    1    0   2
Phalaenopsis-gene378840               0    0    1    1    0   2
Phalaenopsis-gene379957               1    1    1    1    1   5
Phalaenopsis-gene379958               0    0    1    0    0   1
Phalaenopsis-gene379969               1    1    1    1    1   5
Phalaenopsis-gene380466               0    0    1    0    0   1
Phalaenopsis-gene380532               1    1    1    1    1   5
Phalaenopsis-gene38097                0    0    1    0    0   1
Phalaenopsis-gene381639               1    0    1    1    0   3
Phalaenopsis-gene381640               1    1    1    1    1   5
Phalaenopsis-gene381665               1    1    0    1    0   3
Phalaenopsis-gene382077               1    0    0    1    0   2
Phalaenopsis-gene383905               1    0    1    0    0   2
Phalaenopsis-gene383945               1    0    1    1    0   3
Phalaenopsis-gene384265               0    0    1    1    0   2
Phalaenopsis-gene384298               1    0    0    1    0   2
Phalaenopsis-gene384299               0    1    0    1    0   2
Phalaenopsis-gene384554               1    1    1    1    1   5
Phalaenopsis-gene385249               1    1    1    1    1   5
Phalaenopsis-gene38580                1    0    0    0    0   1
Phalaenopsis-gene385898               0    1    1    1    0   3
Phalaenopsis-gene38592                1    0    0    1    0   2
Phalaenopsis-gene38620                1    0    0    0    1   2
Phalaenopsis-gene38650                1    0    0    0    0   1
Phalaenopsis-gene38651                1    0    0    1    0   2
Phalaenopsis-gene386754               1    0    1    0    0   2
Phalaenopsis-gene386755               1    1    1    1    1   5
Phalaenopsis-gene386756               1    0    0    1    1   3
Phalaenopsis-gene387286               1    0    0    1    0   2
Phalaenopsis-gene387287               1    0    1    1    0   3
Phalaenopsis-gene387320               0    0    1    1    0   2
Phalaenopsis-gene387968               1    0    0    1    0   2
Phalaenopsis-gene387987               0    0    1    1    1   3
Phalaenopsis-gene387989               1    1    1    1    1   5
Phalaenopsis-gene388056               1    0    0    1    0   2
Phalaenopsis-gene388143               1    0    1    1    0   3
Phalaenopsis-gene388148               1    1    1    1    1   5
Phalaenopsis-gene388595               1    0    0    1    0   2
Phalaenopsis-gene388636               1    1    1    1    1   5
Phalaenopsis-gene388683               1    0    1    1    0   3
Phalaenopsis-gene388690               1    0    1    1    0   3
Phalaenopsis-gene389231               0    0    0    1    0   1
Phalaenopsis-gene390150               1    0    0    1    0   2
Phalaenopsis-gene390162               1    0    1    1    0   3
Phalaenopsis-gene390191               1    0    0    0    0   1
Phalaenopsis-gene390222               0    0    0    0    1   1
Phalaenopsis-gene391139               1    1    1    1    1   5
Phalaenopsis-gene391151               1    0    0    1    0   2
Phalaenopsis-gene391152               1    0    1    1    0   3
Phalaenopsis-gene393759               1    0    0    0    0   1
Phalaenopsis-gene393764               1    1    1    0    0   3
Phalaenopsis-gene393767               1    1    1    1    0   4
Phalaenopsis-gene393853               1    1    0    1    0   3
Phalaenopsis-gene393858               0    0    0    0    0   0
Phalaenopsis-gene393861               1    0    1    1    0   3
Phalaenopsis-gene393909               0    0    0    0    0   0
Phalaenopsis-gene394030               1    0    1    1    0   3
Phalaenopsis-gene394064               1    0    0    1    0   2
Phalaenopsis-gene394066               1    1    1    1    1   5
Phalaenopsis-gene394098               0    0    1    1    0   2
Phalaenopsis-gene394099               1    0    0    1    0   2
Phalaenopsis-gene394101               1    1    1    1    0   4
Phalaenopsis-gene394132               1    0    1    0    0   2
Phalaenopsis-gene394165               1    1    0    1    0   3
Phalaenopsis-gene394200               1    1    1    1    1   5
Phalaenopsis-gene396946               1    1    0    1    0   3
Phalaenopsis-gene396990               1    0    0    1    0   2
Phalaenopsis-gene397035               0    0    0    1    0   1
Phalaenopsis-gene397036               0    0    0    1    0   1
Phalaenopsis-gene39715                1    0    1    1    0   3
Phalaenopsis-gene39716                1    1    0    0    0   2
Phalaenopsis-gene39742                1    0    1    0    0   2
Phalaenopsis-gene39746                1    0    0    1    0   2
Phalaenopsis-gene397612               0    0    0    0    0   0
Phalaenopsis-gene397645               1    0    0    1    0   2
Phalaenopsis-gene39781                1    0    0    1    0   2
Phalaenopsis-gene397882               0    0    1    1    0   2
Phalaenopsis-gene39812                1    0    0    0    0   1
Phalaenopsis-gene39813                0    0    0    1    0   1
Phalaenopsis-gene398146               1    1    1    1    1   5
Phalaenopsis-gene39842                1    1    1    1    1   5
Phalaenopsis-gene39872                1    0    1    1    0   3
Phalaenopsis-gene39873                1    0    1    1    0   3
Phalaenopsis-gene398784               0    0    0    0    0   0
Phalaenopsis-gene398789               0    1    1    1    0   3
Phalaenopsis-gene398831               1    1    1    1    1   5
Phalaenopsis-gene398836               1    1    1    1    1   5
Phalaenopsis-gene398876               1    1    1    1    1   5
Phalaenopsis-gene39920                1    0    1    1    0   3
Phalaenopsis-gene399388               1    0    0    1    0   2
Phalaenopsis-gene399393               0    0    0    0    0   0
Phalaenopsis-gene399425               1    0    1    1    0   3
Phalaenopsis-gene399431               1    0    1    1    0   3
Phalaenopsis-gene399460               0    0    1    1    0   2
Phalaenopsis-gene399465               0    0    1    1    0   2
Phalaenopsis-gene399466               0    0    0    1    0   1
Phalaenopsis-gene399498               0    0    1    1    0   2
Phalaenopsis-gene399536               1    0    1    1    1   4
Phalaenopsis-gene399542               0    0    1    1    0   2
Phalaenopsis-gene399578               1    0    0    1    0   2
Phalaenopsis-gene399583               1    0    1    0    0   2
Phalaenopsis-gene399584               0    1    1    1    0   3
Phalaenopsis-gene399617               1    0    0    0    0   1
Phalaenopsis-gene399622               1    0    1    1    0   3
Phalaenopsis-gene399623               1    0    1    1    0   3
Phalaenopsis-gene399657               1    0    1    1    0   3
Phalaenopsis-gene399658               1    0    1    1    1   4
Phalaenopsis-gene399701               0    0    0    0    0   0
Phalaenopsis-gene399707               1    0    0    1    0   2
Phalaenopsis-gene400626               1    1    1    1    1   5
Phalaenopsis-gene400858               1    1    1    1    1   5
Phalaenopsis-gene400859               1    0    0    1    0   2
Phalaenopsis-gene401047               1    1    1    1    1   5
Phalaenopsis-gene401048               1    0    1    1    0   3
Phalaenopsis-gene401207               1    1    1    1    1   5
Phalaenopsis-gene401208               1    1    1    1    1   5
Phalaenopsis-gene403051               1    0    0    1    0   2
Phalaenopsis-gene403052               1    1    1    1    1   5
Phalaenopsis-gene403504               0    0    0    1    0   1
Phalaenopsis-gene403505               1    0    0    0    0   1
Phalaenopsis-gene404034               1    1    1    1    1   5
Phalaenopsis-gene404570               0    0    0    1    0   1
Phalaenopsis-gene404875               0    0    0    0    0   0
Phalaenopsis-gene404876               0    0    1    0    0   1
Phalaenopsis-gene405033               1    0    1    1    0   3
Phalaenopsis-gene408352               1    0    1    0    0   2
Phalaenopsis-gene408375               1    0    0    1    1   3
Phalaenopsis-gene408683               0    0    1    1    0   2
Phalaenopsis-gene409415               1    1    1    1    1   5
Phalaenopsis-gene409443               1    0    0    1    0   2
Phalaenopsis-gene409738               0    0    1    1    0   2
Phalaenopsis-gene409740               0    0    1    1    0   2
Phalaenopsis-gene409748               1    0    0    1    0   2
Phalaenopsis-gene409768               1    0    1    1    0   3
Phalaenopsis-gene409827               1    0    1    1    0   3
Phalaenopsis-gene409872               1    0    0    0    0   1
Phalaenopsis-gene409885               0    0    0    1    0   1
Phalaenopsis-gene409886               1    0    0    1    0   2
Phalaenopsis-gene409892               1    0    1    1    0   3
Phalaenopsis-gene409920               1    1    1    1    0   4
Phalaenopsis-gene409956               1    0    0    1    0   2
Phalaenopsis-gene409957               1    0    1    1    0   3
Phalaenopsis-gene409987               1    0    1    0    0   2
Phalaenopsis-gene409993               1    0    0    1    0   2
Phalaenopsis-gene410002               1    0    1    1    0   3
Phalaenopsis-gene410011               1    1    1    1    1   5
Phalaenopsis-gene410012               1    0    1    1    0   3
Phalaenopsis-gene410153               1    0    1    1    0   3
Phalaenopsis-gene410163               1    1    0    1    0   3
Phalaenopsis-gene410211               1    0    0    0    0   1
Phalaenopsis-gene410740               1    0    1    1    0   3
Phalaenopsis-gene410794               1    1    1    1    1   5
Phalaenopsis-gene410933               1    1    1    1    1   5
Phalaenopsis-gene410938               1    0    1    1    0   3
Phalaenopsis-gene410939               1    0    1    1    0   3
Phalaenopsis-gene410976               0    1    1    1    0   3
Phalaenopsis-gene412746               0    0    1    1    0   2
Phalaenopsis-gene413304               1    1    1    1    0   4
Phalaenopsis-gene413453               1    0    1    1    0   3
Phalaenopsis-gene413487               1    0    1    1    0   3
Phalaenopsis-gene413667               1    0    0    1    0   2
Phalaenopsis-gene413668               1    1    0    1    0   3
Phalaenopsis-gene413699               1    0    0    1    0   2
Phalaenopsis-gene413700               1    0    0    1    0   2
Phalaenopsis-gene413802               0    1    0    1    0   2
Phalaenopsis-gene413867               1    0    0    0    0   1
Phalaenopsis-gene413881               1    0    1    1    0   3
Phalaenopsis-gene413915               1    0    0    1    0   2
Phalaenopsis-gene414077               1    0    0    1    0   2
Phalaenopsis-gene414078               1    1    0    1    0   3
Phalaenopsis-gene414187               0    0    0    1    0   1
Phalaenopsis-gene414213               1    1    1    1    0   4
Phalaenopsis-gene414580               1    1    1    1    1   5
Phalaenopsis-gene414728               1    1    1    1    1   5
Phalaenopsis-gene414802               0    0    1    1    0   2
Phalaenopsis-gene415458               1    1    1    1    1   5
Phalaenopsis-gene415459               1    0    1    0    0   2
Phalaenopsis-gene41642                1    1    1    1    1   5
Phalaenopsis-gene416571               1    0    1    1    0   3
Phalaenopsis-gene41829                1    1    1    1    1   5
Phalaenopsis-gene41830                1    0    1    1    0   3
Phalaenopsis-gene418444               1    1    1    1    1   5
Phalaenopsis-gene418477               1    1    1    1    1   5
Phalaenopsis-gene418773               1    1    1    1    1   5
Phalaenopsis-gene418853               0    0    0    1    0   1
Phalaenopsis-gene418995               1    0    1    0    0   2
Phalaenopsis-gene419731               1    1    1    1    1   5
Phalaenopsis-gene421983               1    1    1    1    1   5
Phalaenopsis-gene421994               1    1    1    1    1   5
Phalaenopsis-gene422004               1    0    0    1    0   2
Phalaenopsis-gene422467               1    1    0    1    0   3
Phalaenopsis-gene422545               1    1    1    1    1   5
Phalaenopsis-gene42286                1    0    0    1    0   2
Phalaenopsis-gene42287                1    0    1    0    0   2
Phalaenopsis-gene42364                0    0    0    1    1   2
Phalaenopsis-gene423728               1    1    1    0    0   3
Phalaenopsis-gene423800               1    0    0    1    0   2
Phalaenopsis-gene423805               0    0    1    1    1   3
Phalaenopsis-gene423844               0    0    0    1    0   1
Phalaenopsis-gene424271               1    1    0    1    0   3
Phalaenopsis-gene424436               1    0    0    1    0   2
Phalaenopsis-gene424441               0    1    0    1    0   2
Phalaenopsis-gene424478               0    0    1    1    0   2
Phalaenopsis-gene42492                1    0    0    1    0   2
Phalaenopsis-gene42511                1    1    1    1    1   5
Phalaenopsis-gene42512                1    0    0    1    0   2
Phalaenopsis-gene42514                0    1    1    1    0   3
Phalaenopsis-gene425237               1    0    0    0    0   1
Phalaenopsis-gene425247               1    0    0    1    0   2
Phalaenopsis-gene425256               1    0    0    1    0   2
Phalaenopsis-gene425272               1    0    0    1    0   2
Phalaenopsis-gene425273               1    1    1    1    1   5
Phalaenopsis-gene42540                0    0    0    1    0   1
Phalaenopsis-gene425510               1    1    1    1    0   4
Phalaenopsis-gene425530               0    0    1    1    0   2
Phalaenopsis-gene425664               1    0    1    1    0   3
Phalaenopsis-gene426420               1    1    1    1    1   5
Phalaenopsis-gene426566               1    1    1    1    1   5
Phalaenopsis-gene426585               1    0    1    0    0   2
Phalaenopsis-gene42737                1    1    1    1    1   5
Phalaenopsis-gene42738                0    0    1    1    0   2
Phalaenopsis-gene427452               1    1    1    1    0   4
Phalaenopsis-gene427484               1    1    1    1    1   5
Phalaenopsis-gene428419               1    1    1    1    1   5
Phalaenopsis-gene428545               1    1    1    1    1   5
Phalaenopsis-gene428546               0    0    0    1    0   1
Phalaenopsis-gene428555               1    1    1    1    1   5
Phalaenopsis-gene428556               1    0    1    0    0   2
Phalaenopsis-gene429501               1    1    1    1    1   5
Phalaenopsis-gene429889               1    1    1    1    1   5
Phalaenopsis-gene429905               1    1    1    1    0   4
Phalaenopsis-gene429910               1    0    1    1    0   3
Phalaenopsis-gene430018               1    0    0    0    0   1
Phalaenopsis-gene430371               0    0    1    1    0   2
Phalaenopsis-gene430496               1    0    0    0    0   1
Phalaenopsis-gene430499               1    0    1    0    0   2
Phalaenopsis-gene430539               1    0    1    0    0   2
Phalaenopsis-gene430589               1    1    1    1    1   5
Phalaenopsis-gene430676               1    0    0    1    0   2
Phalaenopsis-gene430679               1    0    0    0    0   1
Phalaenopsis-gene430719               1    0    1    1    0   3
Phalaenopsis-gene430765               0    0    0    0    0   0
Phalaenopsis-gene430766               1    0    0    1    0   2
Phalaenopsis-gene430768               0    0    1    0    0   1
Phalaenopsis-gene430808               1    0    0    1    0   2
Phalaenopsis-gene430843               1    0    0    1    0   2
Phalaenopsis-gene430844               0    1    0    1    0   2
Phalaenopsis-gene431140               1    0    1    1    0   3
Phalaenopsis-gene431426               1    1    1    1    1   5
Phalaenopsis-gene431427               1    1    1    1    1   5
Phalaenopsis-gene431782               0    0    1    1    0   2
Phalaenopsis-gene431798               1    0    1    1    0   3
Phalaenopsis-gene431938               0    0    0    1    0   1
Phalaenopsis-gene431950               1    0    1    1    0   3
Phalaenopsis-gene431982               1    0    1    1    0   3
Phalaenopsis-gene432334               1    1    1    1    1   5
Phalaenopsis-gene432366               1    0    0    1    0   2
Phalaenopsis-gene432798               1    0    1    1    0   3
Phalaenopsis-gene432855               1    0    1    0    0   2
Phalaenopsis-gene432909               1    0    0    1    0   2
Phalaenopsis-gene432964               1    1    1    1    1   5
Phalaenopsis-gene433094               1    0    1    1    0   3
Phalaenopsis-gene433313               1    1    1    1    1   5
Phalaenopsis-gene433547               1    0    0    1    0   2
Phalaenopsis-gene433568               0    0    0    0    1   1
Phalaenopsis-gene433587               1    0    1    0    0   2
Phalaenopsis-gene433591               1    1    1    1    0   4
Phalaenopsis-gene433687               0    0    0    1    0   1
Phalaenopsis-gene433688               1    1    0    1    0   3
Phalaenopsis-gene434121               1    1    1    1    1   5
Phalaenopsis-gene434122               1    0    1    0    0   2
Phalaenopsis-gene434181               0    0    1    0    0   1
Phalaenopsis-gene434221               1    1    1    1    0   4
Phalaenopsis-gene43474                1    1    1    1    1   5
Phalaenopsis-gene43475                1    1    1    1    1   5
Phalaenopsis-gene43484                1    1    1    1    1   5
Phalaenopsis-gene43494                0    0    0    0    0   0
Phalaenopsis-gene43558                1    1    1    1    1   5
Phalaenopsis-gene43559                1    0    0    1    0   2
Phalaenopsis-gene436277               1    0    0    1    0   2
Phalaenopsis-gene436363               1    0    0    1    0   2
Phalaenopsis-gene436364               1    1    1    1    1   5
Phalaenopsis-gene436366               1    1    1    1    1   5
Phalaenopsis-gene436481               1    1    0    1    0   3
Phalaenopsis-gene436721               0    0    0    1    0   1
Phalaenopsis-gene436844               1    1    1    1    1   5
Phalaenopsis-gene436955               1    0    0    1    0   2
Phalaenopsis-gene437330               0    0    0    1    0   1
Phalaenopsis-gene437340               1    0    0    1    0   2
Phalaenopsis-gene43738                0    0    0    0    0   0
Phalaenopsis-gene437627               1    0    0    1    1   3
Phalaenopsis-gene437725               1    0    1    1    0   3
Phalaenopsis-gene437867               1    1    1    1    1   5
Phalaenopsis-gene437897               1    1    1    1    1   5
Phalaenopsis-gene437899               1    0    0    1    0   2
Phalaenopsis-gene437900               1    1    1    1    1   5
Phalaenopsis-gene438739               1    1    1    1    1   5
Phalaenopsis-gene438740               0    1    1    1    0   3
Phalaenopsis-gene438946               0    0    1    1    0   2
Phalaenopsis-gene438997               1    1    1    1    1   5
Phalaenopsis-gene439389               1    1    0    1    0   3
Phalaenopsis-gene439418               1    1    1    1    1   5
Phalaenopsis-gene439953               0    0    1    1    0   2
Phalaenopsis-gene439955               1    0    0    1    1   3
Phalaenopsis-gene440004               1    0    0    1    0   2
Phalaenopsis-gene440006               1    0    0    1    1   3
Phalaenopsis-gene440220               1    0    1    0    0   2
Phalaenopsis-gene441761               1    1    1    1    1   5
Phalaenopsis-gene44205                1    1    1    1    1   5
Phalaenopsis-gene442367               1    1    1    1    1   5
Phalaenopsis-gene44270                1    0    0    1    0   2
Phalaenopsis-gene442765               1    0    1    1    1   4
Phalaenopsis-gene442772               1    1    1    1    1   5
Phalaenopsis-gene44281                1    0    1    1    0   3
Phalaenopsis-gene442964               1    0    1    1    0   3
Phalaenopsis-gene442973               1    1    1    1    1   5
Phalaenopsis-gene442979               1    0    0    0    0   1
Phalaenopsis-gene442987               1    1    1    1    1   5
Phalaenopsis-gene442988               1    0    0    1    0   2
Phalaenopsis-gene442995               1    1    1    1    1   5
Phalaenopsis-gene442999               1    0    0    0    0   1
Phalaenopsis-gene44301                1    1    1    1    1   5
Phalaenopsis-gene44305                1    0    1    0    0   2
Phalaenopsis-gene443137               0    0    1    1    0   2
Phalaenopsis-gene443146               0    1    0    0    0   1
Phalaenopsis-gene443162               1    0    0    0    0   1
Phalaenopsis-gene443169               0    0    1    1    0   2
Phalaenopsis-gene44327                1    0    1    0    0   2
Phalaenopsis-gene443298               1    0    1    0    0   2
Phalaenopsis-gene44366                1    0    1    0    0   2
Phalaenopsis-gene443795               0    0    1    1    0   2
Phalaenopsis-gene444138               1    1    1    1    0   4
Phalaenopsis-gene444704               1    1    1    1    1   5
Phalaenopsis-gene445889               1    0    1    1    0   3
Phalaenopsis-gene446360               1    0    1    1    0   3
Phalaenopsis-gene446737               0    0    0    1    0   1
Phalaenopsis-gene446789               1    0    1    1    0   3
Phalaenopsis-gene447109               1    1    1    1    1   5
Phalaenopsis-gene447984               1    1    0    0    0   2
Phalaenopsis-gene447985               0    1    0    1    0   2
Phalaenopsis-gene448073               1    1    1    1    1   5
Phalaenopsis-gene448126               1    0    0    1    0   2
Phalaenopsis-gene448489               1    1    1    1    1   5
Phalaenopsis-gene448492               1    0    0    0    0   1
Phalaenopsis-gene448501               1    1    1    1    1   5
Phalaenopsis-gene448779               1    0    1    0    0   2
Phalaenopsis-gene448804               1    0    1    1    0   3
Phalaenopsis-gene449151               1    0    1    1    0   3
Phalaenopsis-gene449441               1    0    1    0    0   2
Phalaenopsis-gene449445               1    0    1    0    0   2
Phalaenopsis-gene449623               1    0    0    1    0   2
Phalaenopsis-gene44972                1    1    1    0    0   3
Phalaenopsis-gene44973                0    0    0    0    0   0
Phalaenopsis-gene45010                0    0    1    1    0   2
Phalaenopsis-gene45011                1    0    1    1    1   4
Phalaenopsis-gene450137               1    0    1    1    0   3
Phalaenopsis-gene450174               1    1    1    1    1   5
Phalaenopsis-gene450190               1    1    1    1    1   5
Phalaenopsis-gene450230               1    1    1    1    1   5
Phalaenopsis-gene450231               1    1    1    1    1   5
Phalaenopsis-gene450352               1    1    1    1    1   5
Phalaenopsis-gene450369               1    1    1    1    1   5
Phalaenopsis-gene450760               1    0    1    1    0   3
Phalaenopsis-gene451779               1    0    0    1    0   2
Phalaenopsis-gene452679               1    0    1    1    0   3
Phalaenopsis-gene453893               1    1    1    1    1   5
Phalaenopsis-gene454127               1    1    1    1    1   5
Phalaenopsis-gene454521               0    0    0    0    1   1
Phalaenopsis-gene454689               1    0    1    1    0   3
Phalaenopsis-gene455581               0    0    1    0    0   1
Phalaenopsis-gene455603               1    0    1    1    0   3
Phalaenopsis-gene455631               1    0    1    1    0   3
Phalaenopsis-gene455806               1    1    0    1    0   3
Phalaenopsis-gene455809               1    0    1    1    0   3
Phalaenopsis-gene455842               0    0    1    1    0   2
Phalaenopsis-gene456542               1    1    1    1    1   5
Phalaenopsis-gene456689               1    1    1    1    1   5
Phalaenopsis-gene456690               1    1    1    1    1   5
Phalaenopsis-gene456698               1    1    1    1    1   5
Phalaenopsis-gene456705               1    1    1    1    1   5
Phalaenopsis-gene456706               1    1    1    1    1   5
Phalaenopsis-gene456846               1    1    1    1    1   5
Phalaenopsis-gene456987               1    1    1    1    1   5
Phalaenopsis-gene456988               1    0    1    0    0   2
Phalaenopsis-gene457185               1    0    1    1    0   3
Phalaenopsis-gene457186               0    0    1    0    0   1
Phalaenopsis-gene457316               1    0    0    0    0   1
Phalaenopsis-gene457317               0    0    0    1    0   1
Phalaenopsis-gene457328               1    0    0    0    0   1
Phalaenopsis-gene457338               1    0    1    1    0   3
Phalaenopsis-gene457340               1    1    1    1    1   5
Phalaenopsis-gene457341               0    0    1    1    0   2
Phalaenopsis-gene457345               1    0    1    1    0   3
Phalaenopsis-gene457369               1    1    1    1    1   5
Phalaenopsis-gene458669               1    1    1    1    1   5
Phalaenopsis-gene459091               0    1    0    1    0   2
Phalaenopsis-gene459092               0    0    0    1    0   1
Phalaenopsis-gene459355               1    1    1    1    1   5
Phalaenopsis-gene459429               1    0    1    1    0   3
Phalaenopsis-gene460169               1    0    0    0    0   1
Phalaenopsis-gene460225               0    1    0    1    0   2
Phalaenopsis-gene460461               1    1    1    1    1   5
Phalaenopsis-gene460980               1    0    1    1    0   3
Phalaenopsis-gene461345               1    0    0    1    0   2
Phalaenopsis-gene461820               1    0    1    1    0   3
Phalaenopsis-gene461855               0    0    1    1    0   2
Phalaenopsis-gene461889               0    0    0    1    0   1
Phalaenopsis-gene461925               0    1    1    1    0   3
Phalaenopsis-gene461960               0    0    1    1    0   2
Phalaenopsis-gene462915               1    0    0    1    0   2
Phalaenopsis-gene463027               1    1    1    1    0   4
Phalaenopsis-gene463254               1    0    1    1    0   3
Phalaenopsis-gene463314               1    0    0    1    0   2
Phalaenopsis-gene463510               1    0    1    0    0   2
Phalaenopsis-gene463581               1    1    1    1    1   5
Phalaenopsis-gene465393               1    0    1    1    0   3
Phalaenopsis-gene465775               1    1    1    1    1   5
Phalaenopsis-gene465803               1    1    1    1    1   5
Phalaenopsis-gene465957               1    1    0    1    0   3
Phalaenopsis-gene465959               1    0    1    1    0   3
Phalaenopsis-gene465960               1    0    0    0    0   1
Phalaenopsis-gene465983               1    0    1    1    0   3
Phalaenopsis-gene465985               1    0    0    1    0   2
Phalaenopsis-gene465986               1    1    1    1    0   4
Phalaenopsis-gene466749               1    1    1    1    1   5
Phalaenopsis-gene467119               1    0    0    1    0   2
Phalaenopsis-gene467220               1    1    1    1    1   5
Phalaenopsis-gene467221               1    0    1    0    0   2
Phalaenopsis-gene46759                1    1    1    1    1   5
Phalaenopsis-gene467791               1    1    1    1    1   5
Phalaenopsis-gene467793               1    1    1    1    1   5
Phalaenopsis-gene467794               1    0    0    0    0   1
Phalaenopsis-gene467825               1    0    1    0    0   2
Phalaenopsis-gene467827               1    1    1    1    1   5
Phalaenopsis-gene467944               1    0    1    1    0   3
Phalaenopsis-gene468207               1    1    1    1    1   5
Phalaenopsis-gene468220               1    0    1    0    0   2
Phalaenopsis-gene468251               1    0    1    1    0   3
Phalaenopsis-gene468282               1    0    1    1    0   3
Phalaenopsis-gene468283               1    1    1    1    1   5
Phalaenopsis-gene468331               1    0    0    0    0   1
Phalaenopsis-gene468335               1    0    0    1    0   2
Phalaenopsis-gene468340               0    0    1    0    0   1
Phalaenopsis-gene468341               1    0    0    0    0   1
Phalaenopsis-gene468357               1    0    1    0    0   2
Phalaenopsis-gene468360               1    0    0    1    0   2
Phalaenopsis-gene468557               1    0    0    1    0   2
Phalaenopsis-gene469222               1    0    1    1    0   3
Phalaenopsis-gene469226               1    1    1    1    1   5
Phalaenopsis-gene470101               0    0    1    1    0   2
Phalaenopsis-gene470103               1    1    1    1    1   5
Phalaenopsis-gene470124               1    0    0    1    0   2
Phalaenopsis-gene470125               1    0    0    1    0   2
Phalaenopsis-gene470466               1    1    1    1    1   5
Phalaenopsis-gene470635               1    0    0    0    0   1
Phalaenopsis-gene470636               1    0    0    0    0   1
Phalaenopsis-gene470915               0    0    1    1    0   2
Phalaenopsis-gene471031               1    0    1    0    0   2
Phalaenopsis-gene471142               1    1    1    1    1   5
Phalaenopsis-gene471150               1    0    1    0    0   2
Phalaenopsis-gene471563               0    0    0    0    0   0
Phalaenopsis-gene471564               1    0    1    0    0   2
Phalaenopsis-gene471565               1    1    1    1    1   5
Phalaenopsis-gene471566               0    0    0    1    0   1
Phalaenopsis-gene471570               0    0    0    1    0   1
Phalaenopsis-gene471596               1    0    0    1    0   2
Phalaenopsis-gene471599               1    0    1    0    0   2
Phalaenopsis-gene471624               0    1    0    1    0   2
Phalaenopsis-gene471897               1    1    1    1    0   4
Phalaenopsis-gene471939               0    0    1    0    1   2
Phalaenopsis-gene471981               1    0    0    1    0   2
Phalaenopsis-gene473552               0    0    0    1    0   1
Phalaenopsis-gene4740                 1    0    1    1    0   3
Phalaenopsis-gene47421                0    0    1    0    0   1
Phalaenopsis-gene475186               1    0    1    0    0   2
Phalaenopsis-gene475190               0    0    0    1    0   1
Phalaenopsis-gene475220               1    1    1    1    1   5
Phalaenopsis-gene475222               1    0    0    0    0   1
Phalaenopsis-gene475283               1    1    1    1    1   5
Phalaenopsis-gene475790               1    1    1    1    1   5
Phalaenopsis-gene476340               0    1    1    1    0   3
Phalaenopsis-gene47635                1    1    0    0    0   2
Phalaenopsis-gene47636                1    1    1    1    1   5
Phalaenopsis-gene47648                1    1    1    1    1   5
Phalaenopsis-gene477574               1    0    1    0    0   2
Phalaenopsis-gene477891               1    1    1    1    1   5
Phalaenopsis-gene477927               1    1    1    1    0   4
Phalaenopsis-gene477964               1    0    0    0    0   1
Phalaenopsis-gene477968               1    0    0    1    0   2
Phalaenopsis-gene478000               1    0    1    0    0   2
Phalaenopsis-gene478083               1    1    1    1    1   5
Phalaenopsis-gene478162               1    0    1    0    0   2
Phalaenopsis-gene478577               0    0    1    1    0   2
Phalaenopsis-gene478585               1    0    1    1    0   3
Phalaenopsis-gene478613               1    1    1    1    1   5
Phalaenopsis-gene478621               1    0    1    1    0   3
Phalaenopsis-gene478622               1    0    1    0    0   2
Phalaenopsis-gene478854               1    1    1    1    1   5
Phalaenopsis-gene479514               1    0    0    0    0   1
Phalaenopsis-gene479628               1    1    1    1    1   5
Phalaenopsis-gene479629               1    0    0    0    0   1
Phalaenopsis-gene479652               0    0    1    1    0   2
Phalaenopsis-gene479688               1    1    1    1    1   5
Phalaenopsis-gene479775               1    1    1    1    1   5
Phalaenopsis-gene479776               1    0    1    0    1   3
Phalaenopsis-gene479782               1    0    1    1    0   3
Phalaenopsis-gene479818               1    1    1    1    1   5
Phalaenopsis-gene479819               1    0    1    1    0   3
Phalaenopsis-gene479825               1    0    0    1    0   2
Phalaenopsis-gene479849               1    1    1    1    0   4
Phalaenopsis-gene479855               1    1    1    1    1   5
Phalaenopsis-gene480603               1    0    0    1    0   2
Phalaenopsis-gene480607               1    0    0    0    0   1
Phalaenopsis-gene480635               1    0    1    1    0   3
Phalaenopsis-gene480642               1    0    1    1    0   3
Phalaenopsis-gene480644               1    0    0    1    0   2
Phalaenopsis-gene481030               1    0    1    1    0   3
Phalaenopsis-gene481094               1    0    0    0    0   1
Phalaenopsis-gene481565               1    1    1    1    1   5
Phalaenopsis-gene481566               1    0    1    1    0   3
Phalaenopsis-gene482062               1    1    1    1    1   5
Phalaenopsis-gene482147               0    0    0    0    0   0
Phalaenopsis-gene482170               1    0    1    1    0   3
Phalaenopsis-gene482177               1    1    1    1    1   5
Phalaenopsis-gene482178               1    0    1    1    0   3
Phalaenopsis-gene482602               1    1    1    1    1   5
Phalaenopsis-gene482772               0    0    1    0    0   1
Phalaenopsis-gene482773               1    0    1    1    0   3
Phalaenopsis-gene482812               0    0    1    1    0   2
Phalaenopsis-gene48282                1    0    1    1    0   3
Phalaenopsis-gene482853               0    1    1    0    0   2
Phalaenopsis-gene482854               1    0    1    0    0   2
Phalaenopsis-gene482891               1    1    1    1    0   4
Phalaenopsis-gene483853               1    0    1    1    0   3
Phalaenopsis-gene483854               1    0    1    1    0   3
Phalaenopsis-gene483910               1    1    1    1    1   5
Phalaenopsis-gene485452               1    1    1    1    1   5
Phalaenopsis-gene485465               1    0    0    0    0   1
Phalaenopsis-gene485629               1    0    0    1    0   2
Phalaenopsis-gene485703               0    1    0    1    0   2
Phalaenopsis-gene485710               1    0    1    1    0   3
Phalaenopsis-gene485716               0    0    0    1    0   1
Phalaenopsis-gene485875               1    0    1    1    0   3
Phalaenopsis-gene485881               1    0    0    1    0   2
Phalaenopsis-gene485950               1    0    0    0    1   2
Phalaenopsis-gene485963               1    0    0    1    0   2
Phalaenopsis-gene486031               1    1    1    1    1   5
Phalaenopsis-gene486032               1    0    1    1    0   3
Phalaenopsis-gene486044               0    0    1    1    0   2
Phalaenopsis-gene486045               1    0    0    1    0   2
Phalaenopsis-gene486200               1    0    1    1    0   3
Phalaenopsis-gene486206               1    0    0    1    0   2
Phalaenopsis-gene486277               1    0    1    1    0   3
Phalaenopsis-gene486288               1    0    0    1    0   2
Phalaenopsis-gene486290               0    0    0    1    1   2
Phalaenopsis-gene486719               0    1    1    1    0   3
Phalaenopsis-gene486720               1    0    0    1    0   2
Phalaenopsis-gene486805               1    1    1    1    1   5
Phalaenopsis-gene486844               1    0    1    0    0   2
Phalaenopsis-gene486903               1    1    1    1    1   5
Phalaenopsis-gene486904               1    1    1    1    1   5
Phalaenopsis-gene486913               1    1    1    1    1   5
Phalaenopsis-gene486919               1    1    0    0    0   2
Phalaenopsis-gene487526               1    1    1    1    1   5
Phalaenopsis-gene487544               1    1    1    1    1   5
Phalaenopsis-gene487546               1    1    1    1    1   5
Phalaenopsis-gene487547               1    1    1    1    1   5
Phalaenopsis-gene487549               1    1    1    1    1   5
Phalaenopsis-gene487567               0    0    1    1    0   2
Phalaenopsis-gene487591               1    0    1    1    0   3
Phalaenopsis-gene487754               1    1    1    1    1   5
Phalaenopsis-gene487884               1    0    0    1    0   2
Phalaenopsis-gene487961               1    0    0    1    0   2
Phalaenopsis-gene487971               1    0    0    1    0   2
Phalaenopsis-gene488014               1    0    0    0    0   1
Phalaenopsis-gene488016               1    0    0    1    0   2
Phalaenopsis-gene488024               0    0    0    1    0   1
Phalaenopsis-gene488048               1    0    1    1    0   3
Phalaenopsis-gene488074               0    0    1    1    0   2
Phalaenopsis-gene488090               1    0    1    1    0   3
Phalaenopsis-gene488100               1    0    0    1    0   2
Phalaenopsis-gene488133               1    1    1    1    1   5
Phalaenopsis-gene488153               1    0    1    0    0   2
Phalaenopsis-gene488175               1    0    1    1    0   3
Phalaenopsis-gene488195               0    0    0    0    1   1
Phalaenopsis-gene488231               1    0    0    1    0   2
Phalaenopsis-gene488232               1    1    1    1    1   5
Phalaenopsis-gene488233               1    0    0    0    0   1
Phalaenopsis-gene488250               1    0    0    1    0   2
Phalaenopsis-gene488251               1    1    1    1    1   5
Phalaenopsis-gene488278               1    1    1    1    1   5
Phalaenopsis-gene488296               0    1    1    1    1   4
Phalaenopsis-gene488297               1    0    1    1    0   3
Phalaenopsis-gene488330               1    1    1    1    1   5
Phalaenopsis-gene488331               0    0    1    1    0   2
Phalaenopsis-gene488558               0    0    1    0    0   1
Phalaenopsis-gene489275               1    0    0    0    0   1
Phalaenopsis-gene490318               1    1    1    0    0   3
Phalaenopsis-gene49038                1    1    1    1    1   5
Phalaenopsis-gene490622               0    0    0    1    0   1
Phalaenopsis-gene491488               0    0    0    1    0   1
Phalaenopsis-gene491521               1    0    0    1    0   2
Phalaenopsis-gene491522               1    1    1    1    1   5
Phalaenopsis-gene491998               1    0    0    1    0   2
Phalaenopsis-gene492063               0    0    1    0    0   1
Phalaenopsis-gene492385               1    1    1    1    1   5
Phalaenopsis-gene492818               1    0    1    1    0   3
Phalaenopsis-gene492819               1    1    1    1    1   5
Phalaenopsis-gene492936               1    0    0    1    0   2
Phalaenopsis-gene492937               1    1    1    1    1   5
Phalaenopsis-gene494487               1    0    1    1    0   3
Phalaenopsis-gene494488               1    0    1    1    0   3
Phalaenopsis-gene494567               1    0    1    1    0   3
Phalaenopsis-gene494568               1    1    1    1    1   5
Phalaenopsis-gene495342               1    0    1    1    0   3
Phalaenopsis-gene495396               1    0    1    1    0   3
Phalaenopsis-gene495397               1    1    1    1    0   4
Phalaenopsis-gene495401               1    1    1    1    1   5
Phalaenopsis-gene495447               1    0    1    1    0   3
Phalaenopsis-gene495456               1    1    1    1    1   5
Phalaenopsis-gene497106               1    1    1    1    1   5
Phalaenopsis-gene499408               1    0    0    0    0   1
Phalaenopsis-gene499414               1    0    1    1    0   3
Phalaenopsis-gene499441               1    0    1    0    0   2
Phalaenopsis-gene499447               1    0    1    1    0   3
Phalaenopsis-gene499478               1    1    0    0    0   2
Phalaenopsis-gene499798               1    1    1    1    1   5
Phalaenopsis-gene499799               0    0    0    1    0   1
Phalaenopsis-gene500235               1    0    1    1    0   3
Phalaenopsis-gene500239               1    1    0    1    0   3
Phalaenopsis-gene500243               0    0    0    1    0   1
Phalaenopsis-gene500725               0    0    1    1    0   2
Phalaenopsis-gene501155               1    0    1    0    0   2
Phalaenopsis-gene501183               1    0    1    1    0   3
Phalaenopsis-gene501295               1    1    1    1    1   5
Phalaenopsis-gene501306               0    0    1    1    0   2
Phalaenopsis-gene502159               1    0    1    0    0   2
Phalaenopsis-gene502171               0    0    1    0    0   1
Phalaenopsis-gene502196               1    1    1    1    1   5
Phalaenopsis-gene502208               1    0    0    1    0   2
Phalaenopsis-gene502948               1    0    1    0    0   2
Phalaenopsis-gene502949               1    0    0    0    0   1
Phalaenopsis-gene502965               0    0    1    0    0   1
Phalaenopsis-gene503416               1    0    0    1    0   2
Phalaenopsis-gene503417               1    1    1    1    0   4
Phalaenopsis-gene503873               1    0    0    1    0   2
Phalaenopsis-gene503876               0    0    0    1    0   1
Phalaenopsis-gene503901               1    0    0    0    0   1
Phalaenopsis-gene503904               1    1    0    1    0   3
Phalaenopsis-gene503967               1    1    1    1    1   5
Phalaenopsis-gene504013               0    0    1    0    0   1
Phalaenopsis-gene504175               1    0    1    1    0   3
Phalaenopsis-gene504188               0    0    0    1    0   1
Phalaenopsis-gene504189               1    0    0    0    0   1
Phalaenopsis-gene504303               1    1    1    1    1   5
Phalaenopsis-gene504347               1    1    1    1    1   5
Phalaenopsis-gene504348               1    1    1    1    1   5
Phalaenopsis-gene504568               1    1    1    1    0   4
Phalaenopsis-gene504662               1    0    1    0    1   3
Phalaenopsis-gene50491                1    1    1    1    1   5
Phalaenopsis-gene505491               1    0    0    0    0   1
Phalaenopsis-gene506303               0    0    0    1    0   1
Phalaenopsis-gene507990               1    1    0    1    0   3
Phalaenopsis-gene508010               0    0    1    0    0   1
Phalaenopsis-gene508011               1    1    1    1    1   5
Phalaenopsis-gene508042               1    0    1    1    0   3
Phalaenopsis-gene508051               0    0    1    1    0   2
Phalaenopsis-gene508547               1    0    0    1    0   2
Phalaenopsis-gene508761               0    0    1    1    0   2
Phalaenopsis-gene508762               1    0    1    1    0   3
Phalaenopsis-gene50917                0    0    1    1    0   2
Phalaenopsis-gene509244               1    1    1    1    1   5
Phalaenopsis-gene509668               1    1    1    1    1   5
Phalaenopsis-gene509669               0    0    0    1    0   1
Phalaenopsis-gene510867               1    1    1    1    1   5
Phalaenopsis-gene510875               1    1    1    1    1   5
Phalaenopsis-gene51135                1    1    1    1    1   5
Phalaenopsis-gene511853               1    1    1    1    1   5
Phalaenopsis-gene511875               1    1    1    1    1   5
Phalaenopsis-gene512088               1    0    1    1    0   3
Phalaenopsis-gene512124               0    0    0    1    0   1
Phalaenopsis-gene512186               1    0    0    1    0   2
Phalaenopsis-gene512187               1    1    1    1    1   5
Phalaenopsis-gene512896               1    1    0    0    0   2
Phalaenopsis-gene512906               0    0    0    1    0   1
Phalaenopsis-gene5132                 1    1    1    1    1   5
Phalaenopsis-gene5133                 1    1    1    1    1   5
Phalaenopsis-gene51340                1    0    0    1    0   2
Phalaenopsis-gene513415               1    0    0    1    0   2
Phalaenopsis-gene513416               0    0    1    1    0   2
Phalaenopsis-gene513644               0    0    0    0    0   0
Phalaenopsis-gene513694               1    1    1    1    1   5
Phalaenopsis-gene513735               1    0    0    1    0   2
Phalaenopsis-gene513780               1    0    0    0    0   1
Phalaenopsis-gene513826               1    0    1    1    0   3
Phalaenopsis-gene513874               1    0    1    1    0   3
Phalaenopsis-gene513917               0    0    1    1    0   2
Phalaenopsis-gene513971               0    0    1    0    0   1
Phalaenopsis-gene514052               1    0    1    0    0   2
Phalaenopsis-gene514439               1    0    0    1    0   2
Phalaenopsis-gene51448                1    0    1    1    0   3
Phalaenopsis-gene514712               1    0    1    1    0   3
Phalaenopsis-gene514714               0    0    0    1    0   1
Phalaenopsis-gene514735               1    1    1    1    1   5
Phalaenopsis-gene514747               0    0    1    1    0   2
Phalaenopsis-gene514748               0    0    1    1    0   2
Phalaenopsis-gene514751               1    1    1    1    1   5
Phalaenopsis-gene514797               1    1    1    1    1   5
Phalaenopsis-gene515684               0    0    1    1    0   2
Phalaenopsis-gene515873               0    0    0    1    0   1
Phalaenopsis-gene516155               0    0    1    1    0   2
Phalaenopsis-gene516442               1    0    1    0    0   2
Phalaenopsis-gene516533               1    0    1    0    0   2
Phalaenopsis-gene516534               1    0    0    1    0   2
Phalaenopsis-gene516541               1    1    1    1    1   5
Phalaenopsis-gene516560               1    0    0    0    0   1
Phalaenopsis-gene516561               0    0    1    1    0   2
Phalaenopsis-gene516588               1    0    1    1    0   3
Phalaenopsis-gene516589               1    0    1    0    1   3
Phalaenopsis-gene516613               0    1    0    1    0   2
Phalaenopsis-gene516614               0    0    1    0    0   1
Phalaenopsis-gene516640               1    0    0    0    0   1
Phalaenopsis-gene516641               1    1    0    0    0   2
Phalaenopsis-gene517042               1    1    1    1    1   5
Phalaenopsis-gene517045               1    1    1    1    1   5
Phalaenopsis-gene517061               0    0    1    1    0   2
Phalaenopsis-gene517074               0    0    0    0    0   0
Phalaenopsis-gene517924               1    0    1    1    0   3
Phalaenopsis-gene517957               1    0    1    1    0   3
Phalaenopsis-gene518597               1    1    0    1    0   3
Phalaenopsis-gene518605               1    0    1    1    0   3
Phalaenopsis-gene518606               1    0    1    1    0   3
Phalaenopsis-gene518619               1    0    1    0    0   2
Phalaenopsis-gene518786               0    0    0    1    0   1
Phalaenopsis-gene518787               1    0    0    0    0   1
Phalaenopsis-gene520225               1    0    1    1    0   3
Phalaenopsis-gene520233               1    1    1    1    0   4
Phalaenopsis-gene520559               1    1    1    1    1   5
Phalaenopsis-gene520560               1    0    1    1    0   3
Phalaenopsis-gene520600               1    0    1    0    0   2
Phalaenopsis-gene520710               1    1    0    1    1   4
Phalaenopsis-gene520727               1    1    1    1    1   5
Phalaenopsis-gene520878               1    0    0    1    0   2
Phalaenopsis-gene520893               1    1    1    1    1   5
Phalaenopsis-gene521229               1    1    0    1    0   3
Phalaenopsis-gene521230               1    0    0    1    0   2
Phalaenopsis-gene522070               0    0    1    1    0   2
Phalaenopsis-gene522071               1    0    1    1    0   3
Phalaenopsis-gene522089               1    0    0    1    0   2
Phalaenopsis-gene522091               0    0    1    1    0   2
Phalaenopsis-gene522102               1    0    1    1    0   3
Phalaenopsis-gene522121               1    0    0    1    0   2
Phalaenopsis-gene522139               1    0    0    0    0   1
Phalaenopsis-gene522140               1    1    1    1    1   5
Phalaenopsis-gene522141               1    0    1    1    0   3
Phalaenopsis-gene522152               1    1    1    1    1   5
Phalaenopsis-gene522153               1    0    0    1    0   2
Phalaenopsis-gene522170               1    0    1    0    0   2
Phalaenopsis-gene522189               1    0    1    0    0   2
Phalaenopsis-gene522191               1    0    0    1    0   2
Phalaenopsis-gene522202               1    1    1    1    1   5
Phalaenopsis-gene522203               0    0    0    0    0   0
Phalaenopsis-gene522219               1    0    0    0    0   1
Phalaenopsis-gene522237               0    0    0    1    0   1
Phalaenopsis-gene522238               1    0    1    1    0   3
Phalaenopsis-gene522239               0    0    1    1    0   2
Phalaenopsis-gene522240               0    1    0    1    0   2
Phalaenopsis-gene522251               0    0    0    0    0   0
Phalaenopsis-gene522323               1    0    0    0    0   1
Phalaenopsis-gene522324               1    0    0    0    0   1
Phalaenopsis-gene522339               0    0    1    1    0   2
Phalaenopsis-gene522411               1    0    0    1    0   2
Phalaenopsis-gene522412               1    0    1    0    0   2
Phalaenopsis-gene522513               1    1    1    0    0   3
Phalaenopsis-gene522542               0    0    1    0    0   1
Phalaenopsis-gene522833               1    1    0    1    0   3
Phalaenopsis-gene523020               1    0    0    0    0   1
Phalaenopsis-gene523051               1    1    1    1    1   5
Phalaenopsis-gene523070               0    0    0    0    0   0
Phalaenopsis-gene523121               1    0    0    1    0   2
Phalaenopsis-gene523435               1    1    0    1    0   3
Phalaenopsis-gene523442               1    0    0    0    0   1
Phalaenopsis-gene523486               0    0    0    1    0   1
Phalaenopsis-gene524024               0    1    0    1    0   2
Phalaenopsis-gene524866               1    0    1    0    0   2
Phalaenopsis-gene525840               1    0    1    1    0   3
Phalaenopsis-gene526112               1    0    1    1    0   3
Phalaenopsis-gene526371               1    0    0    1    0   2
Phalaenopsis-gene52661                1    0    1    1    0   3
Phalaenopsis-gene526697               0    0    1    1    0   2
Phalaenopsis-gene527935               1    1    1    1    1   5
Phalaenopsis-gene528182               0    0    0    0    0   0
Phalaenopsis-gene528718               1    0    0    0    1   2
Phalaenopsis-gene528834               0    0    0    1    0   1
Phalaenopsis-gene528835               1    0    1    1    0   3
Phalaenopsis-gene528853               1    0    1    1    0   3
Phalaenopsis-gene529201               1    0    0    1    0   2
Phalaenopsis-gene529202               1    0    1    1    0   3
Phalaenopsis-gene529886               1    0    1    1    0   3
Phalaenopsis-gene529930               0    1    1    0    0   2
Phalaenopsis-gene531075               0    0    0    1    0   1
Phalaenopsis-gene531079               1    0    0    1    0   2
Phalaenopsis-gene531082               1    1    1    1    1   5
Phalaenopsis-gene531089               1    1    1    1    1   5
Phalaenopsis-gene531090               1    0    0    1    0   2
Phalaenopsis-gene531670               1    1    1    1    1   5
Phalaenopsis-gene531671               0    0    0    0    0   0
Phalaenopsis-gene53261                1    1    1    1    1   5
Phalaenopsis-gene532611               1    1    1    1    1   5
Phalaenopsis-gene53269                0    0    1    1    0   2
Phalaenopsis-gene53271                1    0    1    0    0   2
Phalaenopsis-gene532788               1    0    0    1    0   2
Phalaenopsis-gene53299                1    1    1    1    1   5
Phalaenopsis-gene533101               1    1    1    1    1   5
Phalaenopsis-gene533104               1    0    1    1    0   3
Phalaenopsis-gene533105               1    0    0    1    0   2
Phalaenopsis-gene533110               1    0    0    1    0   2
Phalaenopsis-gene533116               1    0    1    1    0   3
Phalaenopsis-gene533156               1    0    1    1    0   3
Phalaenopsis-gene533160               1    0    1    1    1   4
Phalaenopsis-gene533265               1    0    1    1    0   3
Phalaenopsis-gene533280               1    1    1    1    1   5
Phalaenopsis-gene533533               1    1    1    1    1   5
Phalaenopsis-gene533696               1    0    0    0    1   2
Phalaenopsis-gene533697               1    1    1    1    1   5
Phalaenopsis-gene533703               1    1    1    1    1   5
Phalaenopsis-gene534056               1    0    0    1    0   2
Phalaenopsis-gene5358                 1    0    1    1    0   3
Phalaenopsis-gene5359                 1    0    0    0    0   1
Phalaenopsis-gene536700               1    1    1    1    0   4
Phalaenopsis-gene536712               1    0    1    1    0   3
Phalaenopsis-gene537692               0    0    1    1    0   2
Phalaenopsis-gene537693               1    1    1    1    1   5
Phalaenopsis-gene537746               1    1    1    1    1   5
Phalaenopsis-gene537961               1    0    1    1    0   3
Phalaenopsis-gene537962               1    1    1    1    1   5
Phalaenopsis-gene538021               1    0    0    1    0   2
Phalaenopsis-gene538077               1    1    1    1    1   5
Phalaenopsis-gene538078               1    1    1    1    1   5
Phalaenopsis-gene538081               1    0    1    1    0   3
Phalaenopsis-gene538227               1    0    0    1    0   2
Phalaenopsis-gene538259               1    0    1    1    0   3
Phalaenopsis-gene538401               1    1    0    0    0   2
Phalaenopsis-gene538527               1    0    0    0    0   1
Phalaenopsis-gene5390                 1    0    1    1    0   3
Phalaenopsis-gene539020               1    0    1    1    0   3
Phalaenopsis-gene5391                 0    0    1    0    0   1
Phalaenopsis-gene53917                0    0    0    1    0   1
Phalaenopsis-gene539382               1    0    0    1    0   2
Phalaenopsis-gene539383               1    1    1    1    1   5
Phalaenopsis-gene539384               1    1    1    0    0   3
Phalaenopsis-gene539385               0    0    1    1    0   2
Phalaenopsis-gene539387               1    1    1    1    1   5
Phalaenopsis-gene539543               1    1    1    1    1   5
Phalaenopsis-gene539552               1    1    1    1    1   5
Phalaenopsis-gene539887               1    1    1    1    1   5
Phalaenopsis-gene539888               1    1    0    0    0   2
Phalaenopsis-gene54075                1    0    1    1    0   3
Phalaenopsis-gene54224                0    0    1    1    0   2
Phalaenopsis-gene54227                1    0    1    1    0   3
Phalaenopsis-gene54230                1    0    1    1    0   3
Phalaenopsis-gene543274               1    0    1    1    0   3
Phalaenopsis-gene543336               1    0    0    1    0   2
Phalaenopsis-gene543357               0    0    1    1    0   2
Phalaenopsis-gene543358               0    0    0    1    0   1
Phalaenopsis-gene543421               0    0    0    1    0   1
Phalaenopsis-gene543450               1    0    0    0    0   1
Phalaenopsis-gene543502               1    0    1    1    0   3
Phalaenopsis-gene543744               1    0    0    1    0   2
Phalaenopsis-gene543765               1    1    1    1    1   5
Phalaenopsis-gene543775               1    0    0    0    0   1
Phalaenopsis-gene543928               0    0    1    1    0   2
Phalaenopsis-gene543993               1    0    1    0    0   2
Phalaenopsis-gene544050               0    0    1    1    0   2
Phalaenopsis-gene544121               1    0    1    0    0   2
Phalaenopsis-gene544398               1    0    1    1    0   3
Phalaenopsis-gene544399               1    1    0    0    0   2
Phalaenopsis-gene544434               0    0    0    1    0   1
Phalaenopsis-gene544717               1    1    1    1    1   5
Phalaenopsis-gene544718               1    1    1    1    1   5
Phalaenopsis-gene545529               1    1    1    1    1   5
Phalaenopsis-gene545594               1    0    0    1    0   2
Phalaenopsis-gene545641               1    1    1    1    1   5
Phalaenopsis-gene545652               0    0    1    1    0   2
Phalaenopsis-gene545663               0    0    1    1    0   2
Phalaenopsis-gene545916               1    0    0    1    0   2
Phalaenopsis-gene546251               1    0    1    1    0   3
Phalaenopsis-gene546518               1    0    0    1    0   2
Phalaenopsis-gene546583               1    1    1    1    1   5
Phalaenopsis-gene546742               0    0    0    1    0   1
Phalaenopsis-gene547090               1    0    1    1    0   3
Phalaenopsis-gene547095               1    1    1    1    1   5
Phalaenopsis-gene547096               1    1    0    1    1   4
Phalaenopsis-gene547111               1    1    1    1    1   5
Phalaenopsis-gene547132               1    0    0    1    0   2
Phalaenopsis-gene547207               0    1    1    0    0   2
Phalaenopsis-gene547208               1    1    1    1    1   5
Phalaenopsis-gene547310               0    0    0    1    0   1
Phalaenopsis-gene547410               1    0    1    1    0   3
Phalaenopsis-gene548150               1    0    1    1    0   3
Phalaenopsis-gene548151               1    0    1    1    0   3
Phalaenopsis-gene548183               1    0    0    1    0   2
Phalaenopsis-gene548223               1    0    0    1    0   2
Phalaenopsis-gene548230               1    1    1    1    0   4
Phalaenopsis-gene548248               1    1    1    1    1   5
Phalaenopsis-gene548689               1    0    0    1    0   2
Phalaenopsis-gene548713               0    0    1    1    0   2
Phalaenopsis-gene548810               1    1    1    1    1   5
Phalaenopsis-gene549121               1    1    1    1    1   5
Phalaenopsis-gene549162               1    0    1    0    0   2
Phalaenopsis-gene549206               1    1    1    1    1   5
Phalaenopsis-gene549237               1    1    1    1    1   5
Phalaenopsis-gene549243               1    0    1    1    0   3
Phalaenopsis-gene549406               0    0    0    0    0   0
Phalaenopsis-gene549412               1    0    0    1    1   3
Phalaenopsis-gene550118               0    0    1    1    0   2
Phalaenopsis-gene550129               1    0    1    0    0   2
Phalaenopsis-gene550131               1    1    1    1    1   5
Phalaenopsis-gene550133               1    0    1    1    0   3
Phalaenopsis-gene550167               1    0    0    1    0   2
Phalaenopsis-gene550364               1    1    1    1    1   5
Phalaenopsis-gene550368               1    1    1    1    1   5
Phalaenopsis-gene550838               1    0    1    1    0   3
Phalaenopsis-gene550861               0    0    1    1    0   2
Phalaenopsis-gene550863               1    1    1    1    1   5
Phalaenopsis-gene551021               0    0    1    1    0   2
Phalaenopsis-gene551042               0    1    1    0    0   2
Phalaenopsis-gene551222               1    1    0    0    0   2
Phalaenopsis-gene551265               1    0    1    1    0   3
Phalaenopsis-gene551478               1    1    1    1    1   5
Phalaenopsis-gene551507               1    1    1    1    1   5
Phalaenopsis-gene554742               0    0    1    0    0   1
Phalaenopsis-gene555628               1    0    1    1    0   3
Phalaenopsis-gene555704               1    0    0    1    0   2
Phalaenopsis-gene556193               1    1    1    1    1   5
Phalaenopsis-gene556601               0    1    1    1    0   3
Phalaenopsis-gene556602               1    1    1    1    1   5
Phalaenopsis-gene556810               1    0    1    1    0   3
Phalaenopsis-gene556816               1    0    1    1    0   3
Phalaenopsis-gene557480               1    0    1    1    0   3
Phalaenopsis-gene557571               1    0    1    1    0   3
Phalaenopsis-gene557608               1    0    0    1    1   3
Phalaenopsis-gene557609               0    0    1    1    0   2
Phalaenopsis-gene557665               1    1    1    1    1   5
Phalaenopsis-gene557673               1    0    0    1    0   2
Phalaenopsis-gene557718               0    1    0    1    0   2
Phalaenopsis-gene557758               1    1    0    1    0   3
Phalaenopsis-gene557794               1    1    0    1    0   3
Phalaenopsis-gene557795               1    1    1    1    1   5
Phalaenopsis-gene558045               0    0    0    1    0   1
Phalaenopsis-gene558056               1    1    0    1    0   3
Phalaenopsis-gene558109               0    0    0    1    0   1
Phalaenopsis-gene558172               1    0    0    1    0   2
Phalaenopsis-gene558179               0    0    1    1    0   2
Phalaenopsis-gene558237               1    1    1    0    0   3
Phalaenopsis-gene558248               0    0    0    1    0   1
Phalaenopsis-gene558275               1    0    0    0    0   1
Phalaenopsis-gene558278               1    0    0    1    0   2
Phalaenopsis-gene559186               0    0    0    1    1   2
Phalaenopsis-gene559212               1    0    0    1    0   2
Phalaenopsis-gene559247               0    0    0    1    0   1
Phalaenopsis-gene559281               1    0    1    1    1   4
Phalaenopsis-gene559282               1    0    0    1    0   2
Phalaenopsis-gene559310               1    0    1    0    0   2
Phalaenopsis-gene559396               0    1    1    1    0   3
Phalaenopsis-gene559400               1    0    1    1    0   3
Phalaenopsis-gene559852               1    0    0    1    0   2
Phalaenopsis-gene559853               1    0    0    0    0   1
Phalaenopsis-gene559862               1    1    1    1    1   5
Phalaenopsis-gene560448               0    0    0    0    0   0
Phalaenopsis-gene560615               0    1    0    1    0   2
Phalaenopsis-gene560616               1    0    1    0    0   2
Phalaenopsis-gene560624               0    1    0    0    0   1
Phalaenopsis-gene560638               1    1    0    0    0   2
Phalaenopsis-gene560639               1    0    1    0    1   3
Phalaenopsis-gene560685               1    0    0    1    0   2
Phalaenopsis-gene560686               1    1    1    1    1   5
Phalaenopsis-gene560989               1    0    0    1    0   2
Phalaenopsis-gene560996               1    0    1    1    0   3
Phalaenopsis-gene560998               1    1    0    0    0   2
Phalaenopsis-gene561812               0    0    0    0    0   0
Phalaenopsis-gene561927               1    1    1    1    1   5
Phalaenopsis-gene561928               1    1    1    1    1   5
Phalaenopsis-gene561937               1    1    1    1    1   5
Phalaenopsis-gene562241               1    0    0    0    0   1
Phalaenopsis-gene56250                1    1    1    1    1   5
Phalaenopsis-gene56263                1    1    1    1    1   5
Phalaenopsis-gene562657               0    0    1    1    0   2
Phalaenopsis-gene562658               1    1    1    1    1   5
Phalaenopsis-gene562665               1    1    1    1    1   5
Phalaenopsis-gene56273                1    0    1    1    0   3
Phalaenopsis-gene562931               1    1    1    1    1   5
Phalaenopsis-gene562932               0    0    0    0    0   0
Phalaenopsis-gene563026               1    0    0    1    0   2
Phalaenopsis-gene563052               0    0    1    0    0   1
Phalaenopsis-gene563075               1    0    0    0    0   1
Phalaenopsis-gene563076               1    0    1    0    0   2
Phalaenopsis-gene563088               1    1    1    1    0   4
Phalaenopsis-gene563089               1    1    1    1    1   5
Phalaenopsis-gene563094               0    0    0    1    0   1
Phalaenopsis-gene563120               1    0    0    0    0   1
Phalaenopsis-gene563150               1    0    1    0    0   2
Phalaenopsis-gene563168               1    0    0    1    0   2
Phalaenopsis-gene563169               1    1    1    1    1   5
Phalaenopsis-gene563181               1    0    1    0    0   2
Phalaenopsis-gene563182               0    0    1    0    0   1
Phalaenopsis-gene563213               0    0    1    0    0   1
Phalaenopsis-gene563238               1    1    1    1    1   5
Phalaenopsis-gene563243               1    0    1    1    0   3
Phalaenopsis-gene563261               1    0    0    1    0   2
Phalaenopsis-gene563262               1    0    1    1    0   3
Phalaenopsis-gene563274               1    0    1    1    0   3
Phalaenopsis-gene563275               0    0    1    1    0   2
Phalaenopsis-gene563888               1    0    1    0    0   2
Phalaenopsis-gene563902               0    0    1    0    0   1
Phalaenopsis-gene563903               0    0    1    0    0   1
Phalaenopsis-gene56453                1    0    0    1    1   3
Phalaenopsis-gene56457                1    0    1    1    0   3
Phalaenopsis-gene56458                1    0    0    1    0   2
Phalaenopsis-gene564684               1    1    0    1    0   3
Phalaenopsis-gene564700               1    0    0    1    0   2
Phalaenopsis-gene564808               1    0    0    1    0   2
Phalaenopsis-gene56483                0    0    1    1    0   2
Phalaenopsis-gene56487                0    1    0    0    0   1
Phalaenopsis-gene56488                1    0    0    1    1   3
Phalaenopsis-gene56512                1    1    1    1    1   5
Phalaenopsis-gene56513                1    1    1    1    1   5
Phalaenopsis-gene56517                0    1    0    1    0   2
Phalaenopsis-gene56518                1    1    1    1    1   5
Phalaenopsis-gene56541                1    0    0    1    0   2
Phalaenopsis-gene56545                1    0    0    1    0   2
Phalaenopsis-gene56546                1    0    0    0    0   1
Phalaenopsis-gene565472               1    1    1    1    1   5
Phalaenopsis-gene565509               1    0    1    1    0   3
Phalaenopsis-gene56568                1    1    1    1    1   5
Phalaenopsis-gene56569                1    1    1    1    1   5
Phalaenopsis-gene56573                1    1    0    1    0   3
Phalaenopsis-gene565865               1    1    1    1    1   5
Phalaenopsis-gene566002               1    0    0    1    0   2
Phalaenopsis-gene566004               0    0    1    1    0   2
Phalaenopsis-gene566022               0    0    0    1    0   1
Phalaenopsis-gene566067               1    0    1    1    0   3
Phalaenopsis-gene56633                1    0    0    1    0   2
Phalaenopsis-gene566524               1    1    1    1    1   5
Phalaenopsis-gene566574               1    0    1    1    0   3
Phalaenopsis-gene567695               1    1    0    1    0   3
Phalaenopsis-gene567710               1    1    1    0    0   3
Phalaenopsis-gene567711               1    0    0    1    0   2
Phalaenopsis-gene567718               0    0    0    1    0   1
Phalaenopsis-gene567805               0    0    0    1    0   1
Phalaenopsis-gene567820               1    0    1    0    0   2
Phalaenopsis-gene567828               1    0    0    1    0   2
Phalaenopsis-gene567855               0    0    0    1    0   1
Phalaenopsis-gene568303               1    1    1    1    0   4
Phalaenopsis-gene570002               0    0    1    0    1   2
Phalaenopsis-gene570085               1    1    1    1    1   5
Phalaenopsis-gene570088               0    0    1    1    0   2
Phalaenopsis-gene570092               1    0    1    1    0   3
Phalaenopsis-gene570108               1    0    0    1    0   2
Phalaenopsis-gene570115               1    0    1    0    0   2
Phalaenopsis-gene570134               0    0    0    1    0   1
Phalaenopsis-gene570141               1    1    1    0    0   3
Phalaenopsis-gene570847               1    1    1    1    1   5
Phalaenopsis-gene570893               1    0    0    1    0   2
Phalaenopsis-gene570932               1    0    0    0    0   1
Phalaenopsis-gene570933               1    0    0    0    0   1
Phalaenopsis-gene571161               1    1    1    1    1   5
Phalaenopsis-gene571259               1    1    1    1    1   5
Phalaenopsis-gene571268               1    0    0    1    0   2
Phalaenopsis-gene571301               0    1    0    1    0   2
Phalaenopsis-gene571302               1    0    0    1    0   2
Phalaenopsis-gene571719               1    1    1    1    1   5
Phalaenopsis-gene571721               1    0    0    1    0   2
Phalaenopsis-gene572311               1    0    0    1    0   2
Phalaenopsis-gene572974               1    1    1    1    1   5
Phalaenopsis-gene573007               1    0    1    1    0   3
Phalaenopsis-gene573050               0    0    0    1    0   1
Phalaenopsis-gene573134               1    1    0    1    0   3
Phalaenopsis-gene573427               1    0    1    0    0   2
Phalaenopsis-gene573448               0    0    1    0    0   1
Phalaenopsis-gene573449               1    1    1    1    1   5
Phalaenopsis-gene57388                0    0    0    1    0   1
Phalaenopsis-gene57393                1    0    1    1    0   3
Phalaenopsis-gene57422                1    0    1    1    0   3
Phalaenopsis-gene574731               1    1    1    1    1   5
Phalaenopsis-gene575470               1    0    1    1    0   3
Phalaenopsis-gene575516               1    1    0    1    0   3
Phalaenopsis-gene575538               1    0    1    1    1   4
Phalaenopsis-gene575601               0    1    0    0    1   2
Phalaenopsis-gene575608               1    0    1    0    0   2
Phalaenopsis-gene575612               1    0    1    0    0   2
Phalaenopsis-gene575728               1    0    0    1    0   2
Phalaenopsis-gene575735               1    1    1    1    1   5
Phalaenopsis-gene575736               1    0    0    1    0   2
Phalaenopsis-gene576203               1    0    1    1    0   3
Phalaenopsis-gene576473               1    1    1    1    1   5
Phalaenopsis-gene578097               0    0    1    1    0   2
Phalaenopsis-gene578770               1    0    0    1    0   2
Phalaenopsis-gene578795               1    0    0    0    0   1
Phalaenopsis-gene57916                1    0    0    0    0   1
Phalaenopsis-gene57926                1    1    1    1    1   5
Phalaenopsis-gene579480               0    1    1    0    0   2
Phalaenopsis-gene57952                1    0    0    1    0   2
Phalaenopsis-gene57961                1    0    1    1    0   3
Phalaenopsis-gene57962                1    0    1    1    0   3
Phalaenopsis-gene57989                0    0    0    1    0   1
Phalaenopsis-gene57991                0    0    0    1    0   1
Phalaenopsis-gene57992                1    1    1    1    1   5
Phalaenopsis-gene580063               1    1    1    0    0   3
Phalaenopsis-gene580113               1    1    1    1    1   5
Phalaenopsis-gene58042                1    0    1    1    0   3
Phalaenopsis-gene580891               1    0    0    1    0   2
Phalaenopsis-gene582550               1    0    0    1    1   3
Phalaenopsis-gene585486               1    1    1    1    1   5
Phalaenopsis-gene585510               1    0    1    0    0   2
Phalaenopsis-gene585579               0    0    0    0    0   0
Phalaenopsis-gene585960               1    0    1    1    0   3
Phalaenopsis-gene587481               1    1    1    1    1   5
Phalaenopsis-gene587734               1    1    1    1    1   5
Phalaenopsis-gene588144               1    1    1    1    1   5
Phalaenopsis-gene588145               1    0    1    0    0   2
Phalaenopsis-gene588153               1    0    0    0    0   1
Phalaenopsis-gene588155               1    0    0    1    0   2
Phalaenopsis-gene588211               0    0    0    0    0   0
Phalaenopsis-gene58996                0    1    1    1    0   3
Phalaenopsis-gene58997                1    1    1    1    1   5
Phalaenopsis-gene59002                1    1    1    1    0   4
Phalaenopsis-gene59009                0    0    1    1    0   2
Phalaenopsis-gene590530               1    1    1    1    1   5
Phalaenopsis-gene590548               1    0    1    1    1   4
Phalaenopsis-gene590599               0    0    1    1    0   2
Phalaenopsis-gene590611               0    0    1    1    0   2
Phalaenopsis-gene590952               1    0    1    0    0   2
Phalaenopsis-gene591700               1    0    0    1    0   2
Phalaenopsis-gene592038               0    1    0    0    0   1
Phalaenopsis-gene592050               1    0    0    1    0   2
Phalaenopsis-gene592051               1    0    1    1    0   3
Phalaenopsis-gene592233               1    1    1    1    1   5
Phalaenopsis-gene59248                1    0    0    1    0   2
Phalaenopsis-gene592684               1    0    1    1    0   3
Phalaenopsis-gene592851               1    0    1    0    0   2
Phalaenopsis-gene592978               1    0    0    0    0   1
Phalaenopsis-gene592982               1    1    1    1    1   5
Phalaenopsis-gene593002               1    0    0    1    0   2
Phalaenopsis-gene593023               1    0    1    0    0   2
Phalaenopsis-gene593031               1    1    1    1    0   4
Phalaenopsis-gene593066               0    0    1    1    0   2
Phalaenopsis-gene593074               1    0    1    1    0   3
Phalaenopsis-gene593105               1    1    1    1    1   5
Phalaenopsis-gene593107               1    0    1    1    0   3
Phalaenopsis-gene593115               1    1    1    1    1   5
Phalaenopsis-gene59341                1    1    1    1    1   5
Phalaenopsis-gene59352                1    0    0    1    0   2
Phalaenopsis-gene59383                0    1    1    0    0   2
Phalaenopsis-gene59386                1    0    0    0    0   1
Phalaenopsis-gene593971               1    0    1    0    0   2
Phalaenopsis-gene593972               1    1    1    1    0   4
Phalaenopsis-gene594019               0    0    1    1    0   2
Phalaenopsis-gene594289               1    0    1    0    0   2
Phalaenopsis-gene594290               0    0    0    0    0   0
Phalaenopsis-gene594291               1    1    1    1    1   5
Phalaenopsis-gene594293               1    1    1    1    1   5
Phalaenopsis-gene594440               1    1    1    1    1   5
Phalaenopsis-gene594441               0    0    1    0    0   1
Phalaenopsis-gene594442               1    1    1    1    1   5
Phalaenopsis-gene594467               1    1    1    1    1   5
Phalaenopsis-gene594468               1    1    1    1    1   5
Phalaenopsis-gene594469               1    1    0    1    0   3
Phalaenopsis-gene59467                0    0    0    0    0   0
Phalaenopsis-gene59468                1    0    0    0    0   1
Phalaenopsis-gene594724               1    0    0    0    0   1
Phalaenopsis-gene594752               0    0    0    1    0   1
Phalaenopsis-gene594753               1    0    0    1    0   2
Phalaenopsis-gene594779               1    0    0    0    0   1
Phalaenopsis-gene594782               1    0    0    1    0   2
Phalaenopsis-gene59481                1    1    1    1    1   5
Phalaenopsis-gene594963               0    0    1    0    0   1
Phalaenopsis-gene595066               1    0    0    0    0   1
Phalaenopsis-gene595079               1    0    0    1    0   2
Phalaenopsis-gene595129               1    0    0    1    0   2
Phalaenopsis-gene595130               1    1    1    1    1   5
Phalaenopsis-gene595142               1    0    0    1    0   2
Phalaenopsis-gene595203               1    0    0    1    0   2
Phalaenopsis-gene595868               1    1    0    0    1   3
Phalaenopsis-gene595871               1    0    1    1    0   3
Phalaenopsis-gene595894               1    0    1    1    0   3
Phalaenopsis-gene595895               0    0    0    1    0   1
Phalaenopsis-gene595897               1    0    0    0    0   1
Phalaenopsis-gene595921               1    0    0    1    0   2
Phalaenopsis-gene595922               1    0    1    1    0   3
Phalaenopsis-gene595957               1    0    0    1    0   2
Phalaenopsis-gene595981               0    1    1    1    0   3
Phalaenopsis-gene595982               1    0    1    0    0   2
Phalaenopsis-gene595986               0    0    1    1    1   3
Phalaenopsis-gene596025               0    0    0    1    0   1
Phalaenopsis-gene596030               1    1    1    1    1   5
Phalaenopsis-gene596065               0    0    0    0    0   0
Phalaenopsis-gene596501               1    0    1    1    0   3
Phalaenopsis-gene596507               1    1    1    1    1   5
Phalaenopsis-gene596593               1    0    1    1    0   3
Phalaenopsis-gene598119               1    0    1    0    0   2
Phalaenopsis-gene598665               1    0    1    1    0   3
Phalaenopsis-gene598666               1    1    1    1    1   5
Phalaenopsis-gene598900               1    1    1    1    1   5
Phalaenopsis-gene598901               1    1    1    1    1   5
Phalaenopsis-gene598937               1    1    1    1    1   5
Phalaenopsis-gene598938               0    0    1    1    0   2
Phalaenopsis-gene600772               1    0    0    0    0   1
Phalaenopsis-gene60085                0    0    1    1    0   2
Phalaenopsis-gene600939               1    0    0    1    0   2
Phalaenopsis-gene602453               1    0    1    1    0   3
Phalaenopsis-gene60465                1    1    1    1    1   5
Phalaenopsis-gene604909               1    0    1    1    0   3
Phalaenopsis-gene604910               1    0    0    1    0   2
Phalaenopsis-gene605253               1    0    1    0    0   2
Phalaenopsis-gene605642               1    1    1    1    1   5
Phalaenopsis-gene605746               1    0    0    1    0   2
Phalaenopsis-gene605748               1    0    0    1    0   2
Phalaenopsis-gene60614                1    1    1    1    1   5
Phalaenopsis-gene60615                1    0    0    1    0   2
Phalaenopsis-gene60639                1    1    1    1    1   5
Phalaenopsis-gene606576               1    1    1    1    1   5
Phalaenopsis-gene607069               1    0    0    1    0   2
Phalaenopsis-gene607110               1    0    0    1    0   2
Phalaenopsis-gene607638               1    0    1    1    0   3
Phalaenopsis-gene60765                1    0    1    1    0   3
Phalaenopsis-gene60766                1    1    1    1    1   5
Phalaenopsis-gene60782                1    0    1    1    0   3
Phalaenopsis-gene60783                1    1    1    1    1   5
Phalaenopsis-gene60785                1    0    0    1    0   2
Phalaenopsis-gene60817                1    0    0    1    0   2
Phalaenopsis-gene60818                1    0    1    1    0   3
Phalaenopsis-gene60820                1    0    1    1    0   3
Phalaenopsis-gene609348               1    0    0    0    0   1
Phalaenopsis-gene609407               1    0    1    0    0   2
Phalaenopsis-gene609408               0    0    0    0    0   0
Phalaenopsis-gene609497               1    1    1    1    1   5
Phalaenopsis-gene609498               1    1    1    1    1   5
Phalaenopsis-gene61087                1    0    0    1    0   2
Phalaenopsis-gene61137                1    1    1    1    1   5
Phalaenopsis-gene611533               1    1    1    1    1   5
Phalaenopsis-gene611534               0    0    1    0    0   1
Phalaenopsis-gene611566               1    0    0    0    0   1
Phalaenopsis-gene611598               1    0    0    1    0   2
Phalaenopsis-gene6121                 1    1    1    1    1   5
Phalaenopsis-gene612747               1    1    0    0    0   2
Phalaenopsis-gene61281                1    0    0    0    0   1
Phalaenopsis-gene61304                1    0    1    1    0   3
Phalaenopsis-gene614031               0    1    1    1    0   3
Phalaenopsis-gene614320               1    1    1    1    0   4
Phalaenopsis-gene614447               1    0    1    1    0   3
Phalaenopsis-gene614508               1    1    1    1    1   5
Phalaenopsis-gene614523               1    0    0    1    0   2
Phalaenopsis-gene614580               1    1    1    1    1   5
Phalaenopsis-gene614784               1    1    1    1    1   5
Phalaenopsis-gene615235               1    1    1    1    1   5
Phalaenopsis-gene615236               1    1    1    1    1   5
Phalaenopsis-gene615298               1    0    1    1    0   3
Phalaenopsis-gene615745               1    0    0    1    0   2
Phalaenopsis-gene615815               1    0    0    1    0   2
Phalaenopsis-gene615871               0    0    1    0    0   1
Phalaenopsis-gene615872               0    1    1    1    0   3
Phalaenopsis-gene615891               0    0    0    0    0   0
Phalaenopsis-gene615900               0    0    0    1    0   1
Phalaenopsis-gene615956               1    0    1    1    0   3
Phalaenopsis-gene615973               1    1    1    1    1   5
Phalaenopsis-gene615994               1    1    1    1    1   5
Phalaenopsis-gene616013               1    0    0    1    1   3
Phalaenopsis-gene616022               1    0    1    0    0   2
Phalaenopsis-gene616285               1    0    1    1    0   3
Phalaenopsis-gene617836               1    1    1    1    1   5
Phalaenopsis-gene617850               0    0    1    1    0   2
Phalaenopsis-gene617861               1    0    1    0    0   2
Phalaenopsis-gene617862               1    0    0    1    0   2
Phalaenopsis-gene620171               1    1    1    1    1   5
Phalaenopsis-gene620412               1    0    0    1    0   2
Phalaenopsis-gene620421               1    1    1    1    1   5
Phalaenopsis-gene621104               1    0    0    0    0   1
Phalaenopsis-gene621284               1    0    1    1    0   3
Phalaenopsis-gene621333               1    0    0    0    0   1
Phalaenopsis-gene621374               1    0    0    1    0   2
Phalaenopsis-gene621375               1    1    1    1    1   5
Phalaenopsis-gene621667               1    0    1    0    0   2
Phalaenopsis-gene621668               1    1    1    1    1   5
Phalaenopsis-gene623129               1    1    1    1    1   5
Phalaenopsis-gene62334                1    1    1    1    1   5
Phalaenopsis-gene623557               0    0    0    1    0   1
Phalaenopsis-gene623707               1    0    1    0    0   2
Phalaenopsis-gene623759               0    0    1    0    0   1
Phalaenopsis-gene623793               1    0    1    1    0   3
Phalaenopsis-gene624141               1    0    0    0    0   1
Phalaenopsis-gene624174               0    0    0    1    0   1
Phalaenopsis-gene624192               1    0    0    1    0   2
Phalaenopsis-gene624684               1    0    1    1    0   3
Phalaenopsis-gene624696               1    1    1    1    1   5
Phalaenopsis-gene624704               1    1    1    1    1   5
Phalaenopsis-gene625497               1    1    0    1    0   3
Phalaenopsis-gene625499               1    0    0    0    0   1
Phalaenopsis-gene625500               1    0    0    0    0   1
Phalaenopsis-gene62566                1    0    0    0    0   1
Phalaenopsis-gene626111               1    1    1    1    1   5
Phalaenopsis-gene626908               1    1    1    1    1   5
Phalaenopsis-gene626984               1    0    0    0    0   1
Phalaenopsis-gene627063               1    0    0    1    0   2
Phalaenopsis-gene627733               0    0    1    1    0   2
Phalaenopsis-gene62836                1    0    1    0    0   2
Phalaenopsis-gene629182               1    0    1    1    0   3
Phalaenopsis-gene629232               1    1    1    1    1   5
Phalaenopsis-gene629265               1    0    0    0    0   1
Phalaenopsis-gene629663               1    0    0    1    0   2
Phalaenopsis-gene6297                 1    1    1    1    1   5
Phalaenopsis-gene629747               1    1    0    0    0   2
Phalaenopsis-gene629933               1    1    1    1    1   5
Phalaenopsis-gene630168               1    0    1    0    0   2
Phalaenopsis-gene630169               1    0    0    0    0   1
Phalaenopsis-gene630222               1    0    0    1    0   2
Phalaenopsis-gene630557               1    0    1    1    0   3
Phalaenopsis-gene630586               1    1    1    1    1   5
Phalaenopsis-gene630587               1    1    1    1    1   5
Phalaenopsis-gene630588               1    1    1    1    1   5
Phalaenopsis-gene630759               1    0    0    1    0   2
Phalaenopsis-gene630769               1    1    1    1    0   4
Phalaenopsis-gene630796               1    0    1    1    0   3
Phalaenopsis-gene630830               1    0    0    0    0   1
Phalaenopsis-gene631080               0    1    1    1    0   3
Phalaenopsis-gene631480               1    0    0    1    0   2
Phalaenopsis-gene631940               1    1    1    1    1   5
Phalaenopsis-gene631941               1    1    1    1    0   4
Phalaenopsis-gene632323               0    0    0    1    0   1
Phalaenopsis-gene632324               0    1    1    0    0   2
Phalaenopsis-gene632413               0    1    1    1    0   3
Phalaenopsis-gene632414               0    0    0    1    0   1
Phalaenopsis-gene632503               0    0    1    0    0   1
Phalaenopsis-gene632504               1    0    1    0    0   2
Phalaenopsis-gene632594               1    0    0    0    0   1
Phalaenopsis-gene632595               0    0    1    1    0   2
Phalaenopsis-gene632995               1    1    1    1    1   5
Phalaenopsis-gene633046               1    1    1    1    0   4
Phalaenopsis-gene633373               1    0    0    1    0   2
Phalaenopsis-gene633385               1    0    1    0    0   2
Phalaenopsis-gene634141               1    1    1    1    1   5
Phalaenopsis-gene634142               1    0    1    1    0   3
Phalaenopsis-gene635243               1    1    1    1    1   5
Phalaenopsis-gene635244               0    0    1    1    0   2
Phalaenopsis-gene635277               1    1    1    1    1   5
Phalaenopsis-gene635278               0    0    1    1    0   2
Phalaenopsis-gene635867               1    0    1    1    0   3
Phalaenopsis-gene636936               0    1    1    0    0   2
Phalaenopsis-gene637044               0    0    1    1    0   2
Phalaenopsis-gene638071               1    0    1    0    0   2
Phalaenopsis-gene638073               1    1    1    1    1   5
Phalaenopsis-gene638117               1    0    1    0    0   2
Phalaenopsis-gene638577               1    1    1    1    1   5
Phalaenopsis-gene638578               1    0    0    1    0   2
Phalaenopsis-gene638644               1    0    1    1    0   3
Phalaenopsis-gene638669               1    0    1    0    0   2
Phalaenopsis-gene638695               1    0    1    1    0   3
Phalaenopsis-gene63879                1    0    1    0    0   2
Phalaenopsis-gene639000               1    0    0    0    0   1
Phalaenopsis-gene640196               1    1    0    1    0   3
Phalaenopsis-gene640222               1    0    0    1    0   2
Phalaenopsis-gene640223               1    1    1    1    0   4
Phalaenopsis-gene640457               1    1    1    1    1   5
Phalaenopsis-gene640502               1    0    1    1    0   3
Phalaenopsis-gene640993               0    0    1    1    1   3
Phalaenopsis-gene641199               1    0    1    1    0   3
Phalaenopsis-gene641203               1    1    1    1    1   5
Phalaenopsis-gene641220               1    1    1    1    1   5
Phalaenopsis-gene641221               1    1    1    1    1   5
Phalaenopsis-gene641664               1    0    0    0    0   1
Phalaenopsis-gene643250               1    1    1    1    0   4
Phalaenopsis-gene643306               1    0    0    1    0   2
Phalaenopsis-gene643366               1    0    0    1    0   2
Phalaenopsis-gene643706               0    1    0    1    0   2
Phalaenopsis-gene643707               1    0    0    1    0   2
Phalaenopsis-gene643715               1    0    0    0    0   1
Phalaenopsis-gene643716               0    1    0    1    0   2
Phalaenopsis-gene643747               1    0    0    0    0   1
Phalaenopsis-gene643756               0    0    0    1    0   1
Phalaenopsis-gene643757               1    0    0    1    0   2
Phalaenopsis-gene644045               0    0    0    1    1   2
Phalaenopsis-gene644046               1    1    1    1    1   5
Phalaenopsis-gene644049               0    0    1    1    0   2
Phalaenopsis-gene644314               1    1    1    1    1   5
Phalaenopsis-gene644317               1    0    0    0    0   1
Phalaenopsis-gene644320               0    0    1    1    0   2
Phalaenopsis-gene644656               1    0    0    0    0   1
Phalaenopsis-gene644706               1    0    1    1    0   3
Phalaenopsis-gene644777               1    1    0    1    0   3
Phalaenopsis-gene644784               0    0    0    0    0   0
Phalaenopsis-gene645275               1    1    1    1    1   5
Phalaenopsis-gene645276               0    0    1    1    0   2
Phalaenopsis-gene645440               1    0    0    1    0   2
Phalaenopsis-gene645446               1    1    1    1    1   5
Phalaenopsis-gene645456               1    0    0    0    0   1
Phalaenopsis-gene645676               1    0    0    0    0   1
Phalaenopsis-gene645778               0    1    0    0    0   1
Phalaenopsis-gene645849               1    0    0    0    0   1
Phalaenopsis-gene645952               1    0    1    1    0   3
Phalaenopsis-gene645965               0    0    0    1    0   1
Phalaenopsis-gene646195               1    0    0    0    0   1
Phalaenopsis-gene646336               0    0    1    0    0   1
Phalaenopsis-gene646447               1    1    1    1    1   5
Phalaenopsis-gene646471               1    1    0    0    0   2
Phalaenopsis-gene646908               0    0    1    1    0   2
Phalaenopsis-gene646912               1    1    1    1    0   4
Phalaenopsis-gene647005               1    0    1    1    0   3
Phalaenopsis-gene647233               1    0    1    1    0   3
Phalaenopsis-gene647262               1    0    1    0    0   2
Phalaenopsis-gene647263               1    0    1    1    0   3
Phalaenopsis-gene647287               1    1    1    1    1   5
Phalaenopsis-gene647393               0    0    0    1    0   1
Phalaenopsis-gene647394               1    0    1    0    0   2
Phalaenopsis-gene647626               1    1    1    1    1   5
Phalaenopsis-gene648731               0    0    0    1    0   1
Phalaenopsis-gene648732               1    0    0    1    0   2
Phalaenopsis-gene648745               0    0    1    0    0   1
Phalaenopsis-gene648770               0    0    1    1    0   2
Phalaenopsis-gene648864               1    0    1    1    0   3
Phalaenopsis-gene648907               1    0    0    1    0   2
Phalaenopsis-gene64906                1    1    1    1    1   5
Phalaenopsis-gene649328               1    1    0    0    0   2
Phalaenopsis-gene64933                1    1    1    0    0   3
Phalaenopsis-gene649338               0    1    1    1    0   3
Phalaenopsis-gene649383               0    0    1    1    0   2
Phalaenopsis-gene650391               1    0    0    1    0   2
Phalaenopsis-gene650702               0    0    1    0    0   1
Phalaenopsis-gene650747               0    0    0    1    0   1
Phalaenopsis-gene650768               1    0    0    1    0   2
Phalaenopsis-gene651011               0    0    1    1    0   2
Phalaenopsis-gene651052               1    0    1    1    0   3
Phalaenopsis-gene651068               1    0    1    1    0   3
Phalaenopsis-gene651190               1    0    1    1    0   3
Phalaenopsis-gene651482               1    1    1    1    1   5
Phalaenopsis-gene651573               1    0    1    0    0   2
Phalaenopsis-gene651998               1    1    1    1    1   5
Phalaenopsis-gene651999               1    0    0    1    0   2
Phalaenopsis-gene652189               1    0    0    1    0   2
Phalaenopsis-gene65306                0    0    1    1    0   2
Phalaenopsis-gene65307                1    1    1    1    1   5
Phalaenopsis-gene653106               0    0    0    1    1   2
Phalaenopsis-gene65369                0    0    0    0    0   0
Phalaenopsis-gene65402                1    0    0    1    0   2
Phalaenopsis-gene654299               1    0    1    1    0   3
Phalaenopsis-gene65436                1    0    0    0    0   1
Phalaenopsis-gene654371               1    0    1    1    0   3
Phalaenopsis-gene65462                1    0    0    0    0   1
Phalaenopsis-gene65463                0    0    0    1    0   1
Phalaenopsis-gene655410               1    0    1    1    0   3
Phalaenopsis-gene655804               0    0    0    1    0   1
Phalaenopsis-gene655805               1    0    1    1    0   3
Phalaenopsis-gene655814               1    0    1    1    0   3
Phalaenopsis-gene656127               1    1    1    1    1   5
Phalaenopsis-gene656128               1    1    1    1    1   5
Phalaenopsis-gene656286               1    0    0    1    1   3
Phalaenopsis-gene657750               0    0    0    1    0   1
Phalaenopsis-gene657817               0    0    0    1    0   1
Phalaenopsis-gene657923               1    0    1    1    0   3
Phalaenopsis-gene658040               1    1    1    1    0   4
Phalaenopsis-gene658077               1    0    1    1    0   3
Phalaenopsis-gene658078               1    0    0    0    0   1
Phalaenopsis-gene658079               1    1    1    1    1   5
Phalaenopsis-gene658757               1    0    1    1    0   3
Phalaenopsis-gene658760               1    1    1    1    0   4
Phalaenopsis-gene658761               1    0    1    0    0   2
Phalaenopsis-gene658811               1    1    1    1    0   4
Phalaenopsis-gene658814               1    1    1    1    1   5
Phalaenopsis-gene658815               1    0    1    1    0   3
Phalaenopsis-gene658960               0    0    0    1    0   1
Phalaenopsis-gene659048               1    0    0    1    0   2
Phalaenopsis-gene659049               1    0    1    1    1   4
Phalaenopsis-gene659061               1    0    0    1    0   2
Phalaenopsis-gene659062               1    0    0    1    0   2
Phalaenopsis-gene659063               1    0    0    0    0   1
Phalaenopsis-gene659147               1    0    0    1    0   2
Phalaenopsis-gene659148               1    1    1    1    1   5
Phalaenopsis-gene659392               1    0    1    1    0   3
Phalaenopsis-gene659576               1    1    1    1    1   5
Phalaenopsis-gene66009                1    1    1    1    1   5
Phalaenopsis-gene662745               1    1    1    1    1   5
Phalaenopsis-gene662772               1    0    1    1    0   3
Phalaenopsis-gene662800               1    0    1    1    0   3
Phalaenopsis-gene662842               1    0    0    1    0   2
Phalaenopsis-gene662843               1    0    0    1    0   2
Phalaenopsis-gene662845               0    0    1    0    0   1
Phalaenopsis-gene662850               1    1    1    1    1   5
Phalaenopsis-gene662856               0    0    0    1    0   1
Phalaenopsis-gene662892               1    0    1    1    0   3
Phalaenopsis-gene662900               0    1    0    1    0   2
Phalaenopsis-gene66323                1    1    1    1    1   5
Phalaenopsis-gene663244               1    1    1    1    1   5
Phalaenopsis-gene663395               1    0    1    1    0   3
Phalaenopsis-gene663422               1    1    1    1    1   5
Phalaenopsis-gene663423               1    0    1    1    0   3
Phalaenopsis-gene663454               1    0    0    1    0   2
Phalaenopsis-gene663820               1    1    1    1    1   5
Phalaenopsis-gene663830               1    0    1    1    0   3
Phalaenopsis-gene664811               1    0    0    0    0   1
Phalaenopsis-gene664955               1    0    1    1    0   3
Phalaenopsis-gene665414               0    1    0    1    0   2
Phalaenopsis-gene66636                1    0    0    1    0   2
Phalaenopsis-gene668435               1    1    1    1    1   5
Phalaenopsis-gene668715               1    0    0    0    0   1
Phalaenopsis-gene668783               1    1    1    1    1   5
Phalaenopsis-gene668784               1    1    1    1    1   5
Phalaenopsis-gene668789               1    0    0    1    0   2
Phalaenopsis-gene669931               1    0    0    1    0   2
Phalaenopsis-gene670469               0    0    1    0    0   1
Phalaenopsis-gene670477               1    1    1    1    1   5
Phalaenopsis-gene670478               0    1    1    1    0   3
Phalaenopsis-gene670522               1    1    1    1    0   4
Phalaenopsis-gene67283                1    1    1    1    1   5
Phalaenopsis-gene673407               0    0    0    1    0   1
Phalaenopsis-gene67367                1    0    0    1    0   2
Phalaenopsis-gene67369                1    1    1    1    1   5
Phalaenopsis-gene673973               1    0    0    1    0   2
Phalaenopsis-gene67399                1    0    1    1    0   3
Phalaenopsis-gene674011               1    1    1    1    1   5
Phalaenopsis-gene674012               0    0    0    1    0   1
Phalaenopsis-gene674721               1    1    1    1    1   5
Phalaenopsis-gene674914               0    0    0    1    0   1
Phalaenopsis-gene674932               1    0    1    0    0   2
Phalaenopsis-gene676009               1    1    1    1    1   5
Phalaenopsis-gene676132               1    0    0    1    0   2
Phalaenopsis-gene676133               1    0    1    1    0   3
Phalaenopsis-gene676183               0    1    0    1    0   2
Phalaenopsis-gene676184               1    0    0    0    0   1
Phalaenopsis-gene676207               1    0    1    0    0   2
Phalaenopsis-gene676230               0    0    0    1    0   1
Phalaenopsis-gene676267               1    0    0    0    0   1
Phalaenopsis-gene676283               0    1    1    1    0   3
Phalaenopsis-gene676287               0    0    0    1    0   1
Phalaenopsis-gene676356               1    0    0    1    0   2
Phalaenopsis-gene676429               1    1    1    1    1   5
Phalaenopsis-gene676434               1    0    0    0    0   1
Phalaenopsis-gene676607               1    0    1    1    0   3
Phalaenopsis-gene676707               1    0    1    0    0   2
Phalaenopsis-gene676710               1    0    1    1    0   3
Phalaenopsis-gene676767               1    1    1    1    1   5
Phalaenopsis-gene676902               0    0    1    1    0   2
Phalaenopsis-gene676918               0    0    0    0    0   0
Phalaenopsis-gene676919               1    0    1    1    0   3
Phalaenopsis-gene676928               1    1    1    1    1   5
Phalaenopsis-gene677000               1    0    1    0    0   2
Phalaenopsis-gene677001               1    0    1    1    0   3
Phalaenopsis-gene677027               0    0    0    1    0   1
Phalaenopsis-gene677062               1    0    0    0    0   1
Phalaenopsis-gene677107               1    0    0    0    0   1
Phalaenopsis-gene677151               1    0    0    1    0   2
Phalaenopsis-gene677326               1    0    0    1    0   2
Phalaenopsis-gene67734                1    1    1    1    1   5
Phalaenopsis-gene677343               1    0    0    0    0   1
Phalaenopsis-gene677351               0    1    0    0    0   1
Phalaenopsis-gene677352               1    0    0    1    0   2
Phalaenopsis-gene677397               0    0    1    0    0   1
Phalaenopsis-gene677402               1    1    1    1    1   5
Phalaenopsis-gene677418               1    0    0    1    0   2
Phalaenopsis-gene677419               1    1    1    1    1   5
Phalaenopsis-gene677427               1    0    0    1    0   2
Phalaenopsis-gene678211               1    1    1    1    1   5
Phalaenopsis-gene678212               1    1    1    1    1   5
Phalaenopsis-gene678710               1    0    0    1    0   2
Phalaenopsis-gene679277               1    1    1    1    1   5
Phalaenopsis-gene679560               1    1    1    1    1   5
Phalaenopsis-gene679844               1    0    1    0    0   2
Phalaenopsis-gene680671               1    0    0    1    0   2
Phalaenopsis-gene680674               1    0    0    1    0   2
Phalaenopsis-gene680731               1    0    0    0    0   1
Phalaenopsis-gene680781               0    0    0    0    0   0
Phalaenopsis-gene680782               1    1    0    1    0   3
Phalaenopsis-gene680784               1    0    0    1    0   2
Phalaenopsis-gene681231               1    1    1    1    1   5
Phalaenopsis-gene681232               1    0    0    1    0   2
Phalaenopsis-gene681423               1    0    1    0    0   2
Phalaenopsis-gene681424               1    0    0    1    0   2
Phalaenopsis-gene681588               0    0    1    1    0   2
Phalaenopsis-gene681598               1    0    0    1    0   2
Phalaenopsis-gene681599               1    0    1    0    0   2
Phalaenopsis-gene682022               0    1    0    1    0   2
Phalaenopsis-gene682050               1    0    1    0    0   2
Phalaenopsis-gene682102               1    0    0    1    0   2
Phalaenopsis-gene682125               1    1    1    1    0   4
Phalaenopsis-gene682126               1    0    1    0    0   2
Phalaenopsis-gene682161               1    1    1    1    1   5
Phalaenopsis-gene682162               1    1    1    1    1   5
Phalaenopsis-gene68396                0    0    1    1    0   2
Phalaenopsis-gene685544               0    0    0    1    0   1
Phalaenopsis-gene685630               1    0    0    1    0   2
Phalaenopsis-gene685677               0    0    1    0    0   1
Phalaenopsis-gene685725               1    0    1    0    0   2
Phalaenopsis-gene68575                1    0    0    0    0   1
Phalaenopsis-gene685752               1    0    0    0    1   2
Phalaenopsis-gene68576                1    0    1    1    0   3
Phalaenopsis-gene685803               1    0    0    1    0   2
Phalaenopsis-gene687015               1    1    1    1    1   5
Phalaenopsis-gene687025               1    0    1    1    0   3
Phalaenopsis-gene687046               1    0    1    1    0   3
Phalaenopsis-gene687160               0    0    1    1    0   2
Phalaenopsis-gene687161               0    0    1    1    0   2
Phalaenopsis-gene687761               1    1    1    1    1   5
Phalaenopsis-gene688280               1    0    0    0    0   1
Phalaenopsis-gene688281               1    0    0    1    0   2
Phalaenopsis-gene688316               1    1    1    1    1   5
Phalaenopsis-gene688317               0    0    0    1    0   1
Phalaenopsis-gene688356               0    0    0    0    0   0
Phalaenopsis-gene688357               1    0    0    0    0   1
Phalaenopsis-gene689115               1    0    1    1    0   3
Phalaenopsis-gene689116               1    0    0    0    0   1
Phalaenopsis-gene689370               1    0    1    1    0   3
Phalaenopsis-gene689371               1    1    1    1    1   5
Phalaenopsis-gene689628               1    0    0    1    0   2
Phalaenopsis-gene689629               1    0    0    1    0   2
Phalaenopsis-gene689638               0    1    1    1    0   3
Phalaenopsis-gene689685               0    0    0    1    0   1
Phalaenopsis-gene689688               1    0    0    0    0   1
Phalaenopsis-gene68970                1    0    1    1    0   3
Phalaenopsis-gene689723               0    0    0    1    0   1
Phalaenopsis-gene689733               0    0    0    0    0   0
Phalaenopsis-gene689736               1    0    0    1    0   2
Phalaenopsis-gene689762               0    0    0    0    0   0
Phalaenopsis-gene689772               0    0    1    0    0   1
Phalaenopsis-gene690064               0    0    1    1    0   2
Phalaenopsis-gene690079               0    0    0    0    0   0
Phalaenopsis-gene690120               1    0    1    1    0   3
Phalaenopsis-gene690121               0    0    0    1    0   1
Phalaenopsis-gene690146               1    0    0    1    0   2
Phalaenopsis-gene690147               1    0    1    1    0   3
Phalaenopsis-gene690186               0    0    0    0    0   0
Phalaenopsis-gene690187               1    0    0    1    0   2
Phalaenopsis-gene690213               1    0    0    1    0   2
Phalaenopsis-gene690214               1    0    1    0    0   2
Phalaenopsis-gene690246               1    0    0    1    0   2
Phalaenopsis-gene690257               1    1    1    1    1   5
Phalaenopsis-gene690273               0    0    1    0    1   2
Phalaenopsis-gene690379               1    0    0    1    0   2
Phalaenopsis-gene691112               1    0    0    1    0   2
Phalaenopsis-gene691113               1    0    0    0    0   1
Phalaenopsis-gene691196               1    0    1    1    0   3
Phalaenopsis-gene692235               1    1    1    1    1   5
Phalaenopsis-gene692406               0    1    0    1    0   2
Phalaenopsis-gene692428               1    1    0    0    0   2
Phalaenopsis-gene692429               1    0    1    1    0   3
Phalaenopsis-gene692430               0    0    1    1    0   2
Phalaenopsis-gene692448               1    0    1    0    0   2
Phalaenopsis-gene692449               0    0    1    1    0   2
Phalaenopsis-gene692476               1    0    0    0    0   1
Phalaenopsis-gene692477               1    0    1    1    0   3
Phalaenopsis-gene692500               0    0    0    1    0   1
Phalaenopsis-gene692542               1    0    0    1    0   2
Phalaenopsis-gene692593               1    0    1    0    0   2
Phalaenopsis-gene692594               1    0    0    1    0   2
Phalaenopsis-gene693082               1    0    1    1    0   3
Phalaenopsis-gene693122               1    1    1    1    1   5
Phalaenopsis-gene693213               1    1    1    1    0   4
Phalaenopsis-gene693332               1    0    1    1    0   3
Phalaenopsis-gene693472               1    0    0    1    0   2
Phalaenopsis-gene693490               0    0    0    0    0   0
Phalaenopsis-gene693498               0    1    1    1    0   3
Phalaenopsis-gene694119               1    1    1    1    1   5
Phalaenopsis-gene694419               1    1    1    1    1   5
Phalaenopsis-gene694665               1    0    0    1    0   2
Phalaenopsis-gene694682               0    0    0    1    0   1
Phalaenopsis-gene694683               1    0    1    0    0   2
Phalaenopsis-gene694757               1    1    1    1    1   5
Phalaenopsis-gene696572               1    1    1    1    1   5
Phalaenopsis-gene696579               1    0    0    1    0   2
Phalaenopsis-gene697725               0    0    0    0    0   0
Phalaenopsis-gene697980               1    1    1    1    1   5
Phalaenopsis-gene698049               1    0    0    0    0   1
Phalaenopsis-gene698050               1    0    1    1    0   3
Phalaenopsis-gene698117               0    1    0    0    0   1
Phalaenopsis-gene698118               1    1    1    1    1   5
Phalaenopsis-gene698185               0    0    1    1    0   2
Phalaenopsis-gene698186               1    0    1    1    0   3
Phalaenopsis-gene698187               1    0    0    1    0   2
Phalaenopsis-gene698345               1    0    1    1    0   3
Phalaenopsis-gene698346               1    0    0    1    0   2
Phalaenopsis-gene698882               1    0    0    0    0   1
Phalaenopsis-gene698883               1    0    0    0    0   1
Phalaenopsis-gene698932               0    0    0    1    0   1
Phalaenopsis-gene698982               1    1    0    1    0   3
Phalaenopsis-gene700252               1    0    0    1    0   2
Phalaenopsis-gene700405               1    1    1    1    1   5
Phalaenopsis-gene70099                0    0    1    1    0   2
Phalaenopsis-gene701491               1    0    0    0    0   1
Phalaenopsis-gene701493               0    0    1    1    0   2
Phalaenopsis-gene702524               1    1    1    1    1   5
Phalaenopsis-gene702928               1    0    1    0    1   3
Phalaenopsis-gene70413                1    0    1    1    0   3
Phalaenopsis-gene70414                1    1    1    1    1   5
Phalaenopsis-gene70421                1    0    0    0    0   1
Phalaenopsis-gene70443                1    1    1    1    1   5
Phalaenopsis-gene704583               1    0    0    1    0   2
Phalaenopsis-gene704588               1    1    1    1    1   5
Phalaenopsis-gene70466                1    0    1    1    0   3
Phalaenopsis-gene704892               1    1    1    1    1   5
Phalaenopsis-gene704902               0    0    1    1    0   2
Phalaenopsis-gene704927               1    0    0    0    0   1
Phalaenopsis-gene704935               1    1    1    1    1   5
Phalaenopsis-gene704936               1    0    1    1    0   3
Phalaenopsis-gene705249               0    0    1    0    0   1
Phalaenopsis-gene705282               1    0    1    1    1   4
Phalaenopsis-gene706368               1    0    1    1    0   3
Phalaenopsis-gene706377               1    1    1    1    1   5
Phalaenopsis-gene706499               1    0    0    0    0   1
Phalaenopsis-gene706543               0    0    0    1    0   1
Phalaenopsis-gene706562               0    0    0    0    0   0
Phalaenopsis-gene706703               1    1    1    1    1   5
Phalaenopsis-gene706710               1    0    0    0    0   1
Phalaenopsis-gene706711               1    1    1    1    1   5
Phalaenopsis-gene706738               0    1    1    1    0   3
Phalaenopsis-gene706766               0    0    0    1    0   1
Phalaenopsis-gene706831               1    0    1    0    0   2
Phalaenopsis-gene707875               1    0    1    1    0   3
Phalaenopsis-gene708028               1    1    1    1    1   5
Phalaenopsis-gene708033               0    0    1    0    0   1
Phalaenopsis-gene708083               1    0    0    1    0   2
Phalaenopsis-gene708201               1    1    1    0    0   3
Phalaenopsis-gene708202               1    0    1    1    0   3
Phalaenopsis-gene709200               1    0    0    1    0   2
Phalaenopsis-gene709201               1    1    1    1    1   5
Phalaenopsis-gene709244               0    0    0    1    0   1
Phalaenopsis-gene709245               1    0    0    0    0   1
Phalaenopsis-gene709320               1    0    1    1    0   3
Phalaenopsis-gene709321               1    1    1    0    0   3
Phalaenopsis-gene709392               0    1    1    1    0   3
Phalaenopsis-gene709393               1    0    1    1    0   3
Phalaenopsis-gene709470               1    0    1    1    0   3
Phalaenopsis-gene709471               1    1    1    1    1   5
Phalaenopsis-gene709549               1    0    1    1    0   3
Phalaenopsis-gene709550               1    1    1    1    1   5
Phalaenopsis-gene709599               1    1    0    1    0   3
Phalaenopsis-gene710030               1    0    1    1    0   3
Phalaenopsis-gene71028                1    0    1    0    0   2
Phalaenopsis-gene711040               1    0    0    0    0   1
Phalaenopsis-gene712                  0    0    1    1    0   2
Phalaenopsis-gene712179               1    0    1    0    0   2
Phalaenopsis-gene712398               0    0    0    1    0   1
Phalaenopsis-gene713926               1    1    1    1    1   5
Phalaenopsis-gene714279               1    1    1    1    0   4
Phalaenopsis-gene714284               1    1    1    1    1   5
Phalaenopsis-gene714302               1    0    1    1    0   3
Phalaenopsis-gene714303               0    0    0    0    0   0
Phalaenopsis-gene714311               1    1    1    1    1   5
Phalaenopsis-gene715472               1    0    0    1    0   2
Phalaenopsis-gene715498               1    1    1    1    1   5
Phalaenopsis-gene715528               1    0    1    1    0   3
Phalaenopsis-gene715529               1    0    1    1    0   3
Phalaenopsis-gene715557               1    0    0    0    0   1
Phalaenopsis-gene715991               1    0    0    0    0   1
Phalaenopsis-gene716149               1    0    0    1    0   2
Phalaenopsis-gene716150               0    0    1    0    0   1
Phalaenopsis-gene716242               1    0    1    1    0   3
Phalaenopsis-gene716243               1    0    1    1    0   3
Phalaenopsis-gene716257               1    1    0    1    0   3
Phalaenopsis-gene716324               1    1    1    1    0   4
Phalaenopsis-gene716345               1    1    1    1    1   5
Phalaenopsis-gene716346               0    0    1    1    0   2
Phalaenopsis-gene716379               1    0    1    0    0   2
Phalaenopsis-gene716508               0    0    0    0    0   0
Phalaenopsis-gene716711               1    0    0    1    0   2
Phalaenopsis-gene716715               0    0    1    1    0   2
Phalaenopsis-gene716746               1    1    1    1    1   5
Phalaenopsis-gene716750               1    0    1    1    0   3
Phalaenopsis-gene717827               1    0    0    0    0   1
Phalaenopsis-gene717831               0    0    0    0    0   0
Phalaenopsis-gene717905               0    0    1    1    0   2
Phalaenopsis-gene717909               1    1    1    1    1   5
Phalaenopsis-gene718632               1    1    1    1    0   4
Phalaenopsis-gene718633               0    1    0    1    0   2
Phalaenopsis-gene718693               0    0    1    1    0   2
Phalaenopsis-gene718794               1    0    1    1    0   3
Phalaenopsis-gene718820               1    0    0    1    0   2
Phalaenopsis-gene718828               1    1    1    1    0   4
Phalaenopsis-gene71896                0    0    1    1    0   2
Phalaenopsis-gene719220               1    0    1    1    0   3
Phalaenopsis-gene720014               1    1    1    1    1   5
Phalaenopsis-gene720237               1    0    1    0    0   2
Phalaenopsis-gene720870               1    0    0    1    0   2
Phalaenopsis-gene720875               1    0    1    1    0   3
Phalaenopsis-gene722598               1    1    1    1    1   5
Phalaenopsis-gene722616               1    1    1    1    1   5
Phalaenopsis-gene722633               1    0    0    1    0   2
Phalaenopsis-gene722720               1    0    1    0    0   2
Phalaenopsis-gene72336                1    1    0    0    0   2
Phalaenopsis-gene723850               1    1    1    1    1   5
Phalaenopsis-gene723880               1    0    0    0    0   1
Phalaenopsis-gene723910               0    0    0    1    0   1
Phalaenopsis-gene723911               1    0    0    1    0   2
Phalaenopsis-gene723988               1    1    1    1    1   5
Phalaenopsis-gene724105               1    0    1    1    0   3
Phalaenopsis-gene72439                1    0    0    1    0   2
Phalaenopsis-gene724612               0    0    1    0    0   1
Phalaenopsis-gene724676               1    0    0    0    0   1
Phalaenopsis-gene724697               0    0    0    1    0   1
Phalaenopsis-gene724698               1    0    0    1    0   2
Phalaenopsis-gene725112               1    1    1    1    1   5
Phalaenopsis-gene725497               1    0    1    1    0   3
Phalaenopsis-gene725502               1    1    1    1    1   5
Phalaenopsis-gene725511               0    0    0    1    0   1
Phalaenopsis-gene726136               1    1    1    1    1   5
Phalaenopsis-gene726166               0    0    1    0    0   1
Phalaenopsis-gene726173               1    1    1    1    1   5
Phalaenopsis-gene726176               1    1    1    1    1   5
Phalaenopsis-gene726580               1    1    1    1    1   5
Phalaenopsis-gene726612               1    1    1    1    1   5
Phalaenopsis-gene726613               1    1    1    1    1   5
Phalaenopsis-gene726665               1    0    0    1    0   2
Phalaenopsis-gene726666               0    0    0    1    0   1
Phalaenopsis-gene726693               1    0    0    1    0   2
Phalaenopsis-gene726718               1    0    0    0    0   1
Phalaenopsis-gene726781               1    1    1    1    1   5
Phalaenopsis-gene727382               1    0    1    1    0   3
Phalaenopsis-gene727663               1    0    1    1    0   3
Phalaenopsis-gene727782               1    1    0    1    0   3
Phalaenopsis-gene727783               1    1    1    1    1   5
Phalaenopsis-gene728095               1    1    0    1    0   3
Phalaenopsis-gene728100               1    1    1    1    1   5
Phalaenopsis-gene728101               1    0    0    1    1   3
Phalaenopsis-gene728113               0    1    1    1    1   4
Phalaenopsis-gene728193               1    0    0    1    0   2
Phalaenopsis-gene728194               1    0    1    0    0   2
Phalaenopsis-gene728231               1    0    0    1    0   2
Phalaenopsis-gene728232               1    1    1    1    1   5
Phalaenopsis-gene729408               1    0    1    0    0   2
Phalaenopsis-gene729410               1    0    1    1    0   3
Phalaenopsis-gene729429               1    1    1    1    1   5
Phalaenopsis-gene729431               0    0    1    1    0   2
Phalaenopsis-gene729550               1    1    1    1    1   5
Phalaenopsis-gene729570               1    0    0    0    0   1
Phalaenopsis-gene729574               0    1    0    1    0   2
Phalaenopsis-gene729578               1    0    1    0    0   2
Phalaenopsis-gene729619               1    1    1    1    1   5
Phalaenopsis-gene730526               1    0    0    1    0   2
Phalaenopsis-gene730578               0    0    1    1    0   2
Phalaenopsis-gene731511               1    1    1    0    0   3
Phalaenopsis-gene731537               1    1    1    1    1   5
Phalaenopsis-gene731538               1    0    1    1    0   3
Phalaenopsis-gene731567               1    1    1    1    1   5
Phalaenopsis-gene731568               1    1    1    1    1   5
Phalaenopsis-gene732173               1    1    1    1    1   5
Phalaenopsis-gene732321               0    0    1    1    0   2
Phalaenopsis-gene732439               1    1    1    1    1   5
Phalaenopsis-gene733025               1    0    0    1    0   2
Phalaenopsis-gene733034               1    1    1    1    1   5
Phalaenopsis-gene733035               0    0    1    0    0   1
Phalaenopsis-gene733158               0    0    1    0    0   1
Phalaenopsis-gene733161               1    1    1    1    1   5
Phalaenopsis-gene733605               0    0    1    1    0   2
Phalaenopsis-gene733627               1    0    1    1    0   3
Phalaenopsis-gene733628               1    1    1    1    1   5
Phalaenopsis-gene734295               0    1    1    1    0   3
Phalaenopsis-gene734350               1    1    1    1    1   5
Phalaenopsis-gene734351               1    0    0    0    0   1
Phalaenopsis-gene734404               0    0    1    1    0   2
Phalaenopsis-gene734405               1    0    0    1    0   2
Phalaenopsis-gene734407               1    1    1    1    1   5
Phalaenopsis-gene734459               1    0    1    0    0   2
Phalaenopsis-gene734460               1    0    0    1    0   2
Phalaenopsis-gene735133               1    0    1    1    0   3
Phalaenopsis-gene735279               1    0    1    1    0   3
Phalaenopsis-gene735338               1    0    1    1    0   3
Phalaenopsis-gene735613               1    0    1    1    0   3
Phalaenopsis-gene735614               1    0    0    0    0   1
Phalaenopsis-gene735689               1    0    0    1    0   2
Phalaenopsis-gene735766               1    0    1    0    0   2
Phalaenopsis-gene735843               1    1    0    0    0   2
Phalaenopsis-gene73656                1    0    0    0    0   1
Phalaenopsis-gene737102               1    0    1    1    0   3
Phalaenopsis-gene737169               1    0    0    1    0   2
Phalaenopsis-gene739242               1    0    0    1    0   2
Phalaenopsis-gene739243               1    1    1    1    1   5
Phalaenopsis-gene73938                1    0    0    0    0   1
Phalaenopsis-gene739533               1    1    1    1    1   5
Phalaenopsis-gene739534               1    1    1    1    1   5
Phalaenopsis-gene739566               1    1    1    1    1   5
Phalaenopsis-gene73983                1    0    1    1    0   3
Phalaenopsis-gene740418               1    1    1    1    1   5
Phalaenopsis-gene740487               1    0    0    0    0   1
Phalaenopsis-gene740815               1    1    1    1    1   5
Phalaenopsis-gene741116               1    1    1    1    1   5
Phalaenopsis-gene741143               1    1    0    0    0   2
Phalaenopsis-gene741144               1    1    1    1    1   5
Phalaenopsis-gene741158               1    1    1    1    1   5
Phalaenopsis-gene741164               1    1    1    1    1   5
Phalaenopsis-gene742038               1    1    0    1    0   3
Phalaenopsis-gene742039               1    0    0    1    0   2
Phalaenopsis-gene742123               1    1    1    1    1   5
Phalaenopsis-gene742413               1    0    0    0    0   1
Phalaenopsis-gene742485               1    0    1    1    0   3
Phalaenopsis-gene742555               1    0    1    1    0   3
Phalaenopsis-gene742556               1    1    1    1    1   5
Phalaenopsis-gene742578               0    0    1    1    0   2
Phalaenopsis-gene742624               1    0    0    1    0   2
Phalaenopsis-gene742625               1    1    1    1    1   5
Phalaenopsis-gene742670               1    0    1    1    0   3
Phalaenopsis-gene742671               1    1    1    1    1   5
Phalaenopsis-gene742690               1    1    1    1    1   5
Phalaenopsis-gene742794               1    1    1    1    1   5
Phalaenopsis-gene742867               1    1    1    1    1   5
Phalaenopsis-gene744092               0    0    0    0    0   0
Phalaenopsis-gene744200               0    0    1    1    0   2
Phalaenopsis-gene744230               1    0    0    0    0   1
Phalaenopsis-gene744261               1    0    0    1    0   2
Phalaenopsis-gene744262               1    0    1    1    0   3
Phalaenopsis-gene744485               1    0    1    1    0   3
Phalaenopsis-gene744904               1    1    1    1    1   5
Phalaenopsis-gene745979               1    0    0    1    0   2
Phalaenopsis-gene746008               1    0    1    1    0   3
Phalaenopsis-gene746053               0    0    0    1    0   1
Phalaenopsis-gene746111               1    0    0    1    0   2
Phalaenopsis-gene746164               1    0    0    1    0   2
Phalaenopsis-gene746217               1    0    0    1    0   2
Phalaenopsis-gene746308               0    0    0    0    0   0
Phalaenopsis-gene746309               1    1    1    1    1   5
Phalaenopsis-gene746316               0    0    0    1    0   1
Phalaenopsis-gene746369               1    1    1    1    1   5
Phalaenopsis-gene746370               0    0    1    0    0   1
Phalaenopsis-gene746393               1    0    0    1    0   2
Phalaenopsis-gene746416               1    0    0    1    0   2
Phalaenopsis-gene746793               1    0    1    1    0   3
Phalaenopsis-gene74681                1    1    1    1    1   5
Phalaenopsis-gene747347               0    0    1    1    0   2
Phalaenopsis-gene747349               1    1    1    1    1   5
Phalaenopsis-gene747389               1    1    1    1    0   4
Phalaenopsis-gene747874               1    1    1    1    1   5
Phalaenopsis-gene747875               1    0    0    0    0   1
Phalaenopsis-gene747889               1    0    0    1    0   2
Phalaenopsis-gene747892               1    0    1    1    0   3
Phalaenopsis-gene747911               1    1    1    1    0   4
Phalaenopsis-gene74802                1    0    1    0    0   2
Phalaenopsis-gene74833                1    0    0    1    0   2
Phalaenopsis-gene74864                1    0    1    1    0   3
Phalaenopsis-gene74943                1    1    1    1    1   5
Phalaenopsis-gene74944                0    0    1    0    0   1
Phalaenopsis-gene749561               0    0    0    1    0   1
Phalaenopsis-gene750625               0    0    0    1    0   1
Phalaenopsis-gene750718               0    0    0    0    0   0
Phalaenopsis-gene751076               0    0    1    0    0   1
Phalaenopsis-gene751346               1    0    1    0    0   2
Phalaenopsis-gene751367               1    0    0    0    0   1
Phalaenopsis-gene751415               0    0    0    0    0   0
Phalaenopsis-gene751425               1    0    1    1    0   3
Phalaenopsis-gene751429               1    0    0    1    0   2
Phalaenopsis-gene751430               1    0    0    1    0   2
Phalaenopsis-gene751678               0    0    0    1    0   1
Phalaenopsis-gene751979               1    1    1    1    1   5
Phalaenopsis-gene751987               1    1    1    1    1   5
Phalaenopsis-gene75260                1    0    1    1    0   3
Phalaenopsis-gene753414               1    0    0    1    0   2
Phalaenopsis-gene753719               1    1    1    1    1   5
Phalaenopsis-gene753732               1    0    0    1    0   2
Phalaenopsis-gene753736               1    0    0    1    0   2
Phalaenopsis-gene753737               0    0    1    0    0   1
Phalaenopsis-gene753808               1    0    1    0    0   2
Phalaenopsis-gene753813               1    1    1    1    1   5
Phalaenopsis-gene753814               1    1    1    1    1   5
Phalaenopsis-gene753968               1    1    1    1    1   5
Phalaenopsis-gene75397                1    1    1    1    1   5
Phalaenopsis-gene754294               1    1    1    1    1   5
Phalaenopsis-gene754646               1    0    1    1    0   3
Phalaenopsis-gene754700               1    0    0    1    0   2
Phalaenopsis-gene754701               1    0    0    1    0   2
Phalaenopsis-gene754702               1    1    1    1    1   5
Phalaenopsis-gene755036               0    1    1    1    0   3
Phalaenopsis-gene755043               1    1    1    0    0   3
Phalaenopsis-gene75512                1    0    1    0    0   2
Phalaenopsis-gene755142               1    1    1    1    1   5
Phalaenopsis-gene756339               1    0    1    1    0   3
Phalaenopsis-gene757033               1    0    1    1    0   3
Phalaenopsis-gene757034               1    0    1    1    0   3
Phalaenopsis-gene757044               1    1    0    1    0   3
Phalaenopsis-gene757045               0    1    0    0    0   1
Phalaenopsis-gene75762                1    1    1    1    1   5
Phalaenopsis-gene75767                1    1    1    1    1   5
Phalaenopsis-gene757741               1    1    1    1    1   5
Phalaenopsis-gene757852               1    1    1    1    1   5
Phalaenopsis-gene757874               1    1    1    1    1   5
Phalaenopsis-gene758015               0    0    0    1    0   1
Phalaenopsis-gene758298               1    1    1    1    0   4
Phalaenopsis-gene759072               1    1    1    0    0   3
Phalaenopsis-gene759086               1    1    1    1    1   5
Phalaenopsis-gene760136               1    1    1    1    1   5
Phalaenopsis-gene760137               1    0    1    0    0   2
Phalaenopsis-gene760143               1    1    1    1    1   5
Phalaenopsis-gene76038                1    0    0    1    0   2
Phalaenopsis-gene76045                1    0    0    1    0   2
Phalaenopsis-gene760805               1    1    1    1    1   5
Phalaenopsis-gene761594               1    0    0    1    0   2
Phalaenopsis-gene761595               1    0    1    0    0   2
Phalaenopsis-gene761636               1    0    1    0    0   2
Phalaenopsis-gene761793               1    1    1    1    1   5
Phalaenopsis-gene761794               1    0    0    1    0   2
Phalaenopsis-gene761837               0    1    0    0    0   1
Phalaenopsis-gene76187                0    0    1    1    0   2
Phalaenopsis-gene761872               1    0    0    1    0   2
Phalaenopsis-gene761873               0    0    0    0    0   0
Phalaenopsis-gene762705               1    1    1    1    1   5
Phalaenopsis-gene763196               1    1    1    1    1   5
Phalaenopsis-gene763237               0    0    1    0    0   1
Phalaenopsis-gene763263               1    1    1    1    1   5
Phalaenopsis-gene763264               1    0    1    0    0   2
Phalaenopsis-gene763266               1    1    1    1    1   5
Phalaenopsis-gene763660               1    0    1    1    0   3
Phalaenopsis-gene763664               1    0    1    1    0   3
Phalaenopsis-gene763675               0    0    0    0    0   0
Phalaenopsis-gene763716               0    0    1    0    0   1
Phalaenopsis-gene763725               1    0    1    1    0   3
Phalaenopsis-gene763727               1    1    1    1    1   5
Phalaenopsis-gene764197               1    0    0    0    0   1
Phalaenopsis-gene764278               1    1    0    1    0   3
Phalaenopsis-gene764440               1    0    0    1    0   2
Phalaenopsis-gene764812               1    1    1    1    1   5
Phalaenopsis-gene764820               1    1    1    1    1   5
Phalaenopsis-gene764828               1    1    1    1    1   5
Phalaenopsis-gene765870               1    0    1    1    0   3
Phalaenopsis-gene766021               1    0    1    0    0   2
Phalaenopsis-gene766026               1    1    1    1    1   5
Phalaenopsis-gene766031               1    1    1    1    1   5
Phalaenopsis-gene766032               1    0    1    1    0   3
Phalaenopsis-gene766064               1    0    1    0    0   2
Phalaenopsis-gene766075               1    1    1    1    1   5
Phalaenopsis-gene766592               1    1    1    1    1   5
Phalaenopsis-gene767331               1    1    1    1    1   5
Phalaenopsis-gene767787               1    0    0    1    0   2
Phalaenopsis-gene767984               1    1    1    1    1   5
Phalaenopsis-gene768592               1    1    1    1    1   5
Phalaenopsis-gene769418               1    0    1    1    0   3
Phalaenopsis-gene77036                1    0    0    1    0   2
Phalaenopsis-gene770601               1    0    1    1    0   3
Phalaenopsis-gene770602               1    1    1    1    1   5
Phalaenopsis-gene770633               1    1    1    1    1   5
Phalaenopsis-gene770636               1    0    1    0    0   2
Phalaenopsis-gene770899               1    0    1    0    0   2
Phalaenopsis-gene770920               1    1    1    1    1   5
Phalaenopsis-gene771225               1    0    1    0    0   2
Phalaenopsis-gene771250               1    0    0    0    0   1
Phalaenopsis-gene771276               0    0    0    0    0   0
Phalaenopsis-gene771748               1    1    1    0    0   3
Phalaenopsis-gene772366               0    0    1    0    0   1
Phalaenopsis-gene772428               1    1    1    1    1   5
Phalaenopsis-gene772700               1    1    1    1    1   5
Phalaenopsis-gene772738               1    0    1    1    0   3
Phalaenopsis-gene772747               1    1    1    1    1   5
Phalaenopsis-gene773160               1    1    1    1    1   5
Phalaenopsis-gene773851               1    0    0    1    0   2
Phalaenopsis-gene774228               1    0    0    1    0   2
Phalaenopsis-gene774234               1    0    0    1    0   2
Phalaenopsis-gene774237               0    1    1    1    0   3
Phalaenopsis-gene774263               1    1    1    1    1   5
Phalaenopsis-gene774264               1    1    1    1    1   5
Phalaenopsis-gene774304               1    0    0    1    0   2
Phalaenopsis-gene774330               1    0    1    1    0   3
Phalaenopsis-gene774337               0    1    0    1    0   2
Phalaenopsis-gene774358               1    1    1    1    1   5
Phalaenopsis-gene774366               1    0    1    1    0   3
Phalaenopsis-gene774384               1    1    0    0    0   2
Phalaenopsis-gene774392               1    0    0    0    0   1
Phalaenopsis-gene774865               0    0    1    1    0   2
Phalaenopsis-gene775395               1    0    0    1    0   2
Phalaenopsis-gene775415               0    0    0    0    0   0
Phalaenopsis-gene775434               0    0    0    1    0   1
Phalaenopsis-gene775435               1    0    0    1    0   2
Phalaenopsis-gene775454               0    0    1    1    0   2
Phalaenopsis-gene775493               1    1    1    1    1   5
Phalaenopsis-gene775495               1    0    1    1    0   3
Phalaenopsis-gene775521               1    0    1    1    0   3
Phalaenopsis-gene776190               1    0    0    1    0   2
Phalaenopsis-gene776192               0    1    0    1    0   2
Phalaenopsis-gene776684               1    1    0    0    0   2
Phalaenopsis-gene776725               0    1    1    1    0   3
Phalaenopsis-gene77810                1    0    0    1    0   2
Phalaenopsis-gene77881                1    0    1    1    0   3
Phalaenopsis-gene779653               1    1    1    1    1   5
Phalaenopsis-gene779658               1    0    0    1    0   2
Phalaenopsis-gene779730               0    0    1    1    0   2
Phalaenopsis-gene779756               0    0    1    1    0   2
Phalaenopsis-gene779785               1    1    1    1    1   5
Phalaenopsis-gene780198               1    1    1    1    1   5
Phalaenopsis-gene780235               1    0    1    0    0   2
Phalaenopsis-gene780241               1    0    0    0    0   1
Phalaenopsis-gene780725               1    1    1    1    1   5
Phalaenopsis-gene781189               1    0    0    1    0   2
Phalaenopsis-gene781201               1    0    1    1    0   3
Phalaenopsis-gene781222               1    1    1    1    0   4
Phalaenopsis-gene781228               1    1    1    1    1   5
Phalaenopsis-gene781229               1    1    1    1    1   5
Phalaenopsis-gene781235               1    0    1    1    0   3
Phalaenopsis-gene781236               1    0    0    0    0   1
Phalaenopsis-gene781264               1    1    1    1    1   5
Phalaenopsis-gene781265               1    0    0    0    1   2
Phalaenopsis-gene781298               1    0    1    1    0   3
Phalaenopsis-gene781303               1    1    1    1    1   5
Phalaenopsis-gene781311               1    0    0    1    0   2
Phalaenopsis-gene782100               0    1    1    1    0   3
Phalaenopsis-gene782861               1    1    1    1    1   5
Phalaenopsis-gene782944               1    1    1    1    1   5
Phalaenopsis-gene783380               1    1    1    1    1   5
Phalaenopsis-gene783381               1    0    1    0    0   2
Phalaenopsis-gene783383               1    1    1    1    1   5
Phalaenopsis-gene783702               1    1    1    1    1   5
Phalaenopsis-gene784051               0    0    0    1    0   1
Phalaenopsis-gene784186               1    1    1    1    1   5
Phalaenopsis-gene784200               1    1    1    1    1   5
Phalaenopsis-gene784203               0    0    0    1    0   1
Phalaenopsis-gene784850               1    0    1    0    0   2
Phalaenopsis-gene78511                0    0    0    1    0   1
Phalaenopsis-gene78512                0    1    0    1    0   2
Phalaenopsis-gene78531                0    0    0    1    0   1
Phalaenopsis-gene785372               0    0    1    1    0   2
Phalaenopsis-gene785405               0    0    1    1    0   2
Phalaenopsis-gene785540               0    0    1    1    0   2
Phalaenopsis-gene785541               0    0    0    1    0   1
Phalaenopsis-gene785686               1    1    1    1    1   5
Phalaenopsis-gene785913               1    1    1    1    1   5
Phalaenopsis-gene786136               0    1    1    1    0   3
Phalaenopsis-gene786150               1    1    1    1    0   4
Phalaenopsis-gene786154               1    1    1    1    1   5
Phalaenopsis-gene786423               1    1    1    1    1   5
Phalaenopsis-gene786429               1    1    1    1    1   5
Phalaenopsis-gene786440               0    1    1    0    0   2
Phalaenopsis-gene786504               1    1    1    1    1   5
Phalaenopsis-gene786508               1    0    0    1    0   2
Phalaenopsis-gene786941               1    1    1    0    0   3
Phalaenopsis-gene787082               1    0    1    1    0   3
Phalaenopsis-gene787083               1    1    1    1    1   5
Phalaenopsis-gene787114               1    1    1    1    0   4
Phalaenopsis-gene787115               1    0    0    0    1   2
Phalaenopsis-gene787144               1    0    1    1    0   3
Phalaenopsis-gene787145               1    0    0    1    0   2
Phalaenopsis-gene787174               1    0    1    1    0   3
Phalaenopsis-gene788737               0    0    0    0    0   0
Phalaenopsis-gene788979               1    1    1    1    1   5
Phalaenopsis-gene789484               0    0    1    1    0   2
Phalaenopsis-gene789564               1    0    0    1    0   2
Phalaenopsis-gene789583               1    1    1    1    1   5
Phalaenopsis-gene789589               0    0    1    1    0   2
Phalaenopsis-gene789692               1    0    0    1    0   2
Phalaenopsis-gene789738               1    0    1    1    0   3
Phalaenopsis-gene789783               0    0    1    1    0   2
Phalaenopsis-gene789784               1    0    0    1    0   2
Phalaenopsis-gene789829               1    0    1    1    0   3
Phalaenopsis-gene790109               1    1    0    0    0   2
Phalaenopsis-gene79017                0    0    1    1    0   2
Phalaenopsis-gene79019                1    0    1    1    0   3
Phalaenopsis-gene792363               0    0    0    0    0   0
Phalaenopsis-gene792464               1    1    1    1    1   5
Phalaenopsis-gene792504               1    0    0    1    0   2
Phalaenopsis-gene792604               0    0    1    0    0   1
Phalaenopsis-gene792622               0    0    1    0    0   1
Phalaenopsis-gene792654               1    0    0    1    0   2
Phalaenopsis-gene793732               1    0    1    1    0   3
Phalaenopsis-gene793733               1    1    1    1    1   5
Phalaenopsis-gene793734               0    0    1    1    0   2
Phalaenopsis-gene793735               1    1    1    1    0   4
Phalaenopsis-gene793773               1    0    1    1    0   3
Phalaenopsis-gene793774               1    0    1    0    0   2
Phalaenopsis-gene793775               0    0    1    0    0   1
Phalaenopsis-gene793796               0    0    0    1    0   1
Phalaenopsis-gene793800               1    1    1    1    1   5
Phalaenopsis-gene794959               0    0    1    0    0   1
Phalaenopsis-gene794961               1    1    1    1    0   4
Phalaenopsis-gene794999               1    1    1    1    1   5
Phalaenopsis-gene795000               1    1    1    1    0   4
Phalaenopsis-gene795006               1    1    1    1    1   5
Phalaenopsis-gene795127               1    1    1    1    1   5
Phalaenopsis-gene796225               1    1    1    1    1   5
Phalaenopsis-gene796316               1    0    1    0    0   2
Phalaenopsis-gene797176               1    0    1    1    0   3
Phalaenopsis-gene797204               0    0    1    1    0   2
Phalaenopsis-gene7993                 1    0    0    0    0   1
Phalaenopsis-gene799678               1    0    0    1    0   2
Phalaenopsis-gene799679               1    1    0    0    0   2
Phalaenopsis-gene799832               1    1    1    1    0   4
Phalaenopsis-gene800003               1    1    1    1    1   5
Phalaenopsis-gene800007               1    1    1    1    1   5
Phalaenopsis-gene800008               1    1    1    1    1   5
Phalaenopsis-gene800582               1    0    0    0    0   1
Phalaenopsis-gene801810               1    0    1    1    0   3
Phalaenopsis-gene801811               1    1    1    1    1   5
Phalaenopsis-gene801842               0    0    1    1    0   2
Phalaenopsis-gene801847               1    0    0    1    0   2
Phalaenopsis-gene803658               1    1    1    1    1   5
Phalaenopsis-gene803696               1    0    1    1    0   3
Phalaenopsis-gene804123               1    1    0    0    0   2
Phalaenopsis-gene804161               1    1    1    1    1   5
Phalaenopsis-gene804162               0    0    0    1    0   1
Phalaenopsis-gene804198               1    0    0    1    0   2
Phalaenopsis-gene80454                1    1    0    1    0   3
Phalaenopsis-gene80455                0    0    1    0    0   1
Phalaenopsis-gene805035               1    0    1    0    0   2
Phalaenopsis-gene805648               1    0    0    1    0   2
Phalaenopsis-gene805649               1    0    0    0    0   1
Phalaenopsis-gene806003               1    0    1    0    0   2
Phalaenopsis-gene806004               0    0    1    1    0   2
Phalaenopsis-gene806903               1    0    1    1    0   3
Phalaenopsis-gene806946               0    0    1    1    0   2
Phalaenopsis-gene807125               1    1    1    1    1   5
Phalaenopsis-gene807141               1    1    1    1    1   5
Phalaenopsis-gene807142               1    1    1    1    1   5
Phalaenopsis-gene807316               1    1    1    1    1   5
Phalaenopsis-gene808545               1    0    1    0    0   2
Phalaenopsis-gene808555               1    1    0    0    0   2
Phalaenopsis-gene808717               0    0    1    1    0   2
Phalaenopsis-gene809639               1    1    1    1    1   5
Phalaenopsis-gene810389               1    0    0    1    0   2
Phalaenopsis-gene810457               0    0    0    0    0   0
Phalaenopsis-gene811361               1    0    0    1    0   2
Phalaenopsis-gene811369               1    1    1    1    1   5
Phalaenopsis-gene811370               1    0    0    1    0   2
Phalaenopsis-gene811537               0    0    0    1    0   1
Phalaenopsis-gene811538               1    0    1    1    1   4
Phalaenopsis-gene81193                1    1    1    1    1   5
Phalaenopsis-gene812153               0    0    1    1    0   2
Phalaenopsis-gene812155               1    1    1    1    1   5
Phalaenopsis-gene812207               1    0    0    1    0   2
Phalaenopsis-gene812481               1    0    0    0    0   1
Phalaenopsis-gene812547               1    0    1    0    0   2
Phalaenopsis-gene813030               0    0    0    0    0   0
Phalaenopsis-gene814276               1    0    1    1    0   3
Phalaenopsis-gene814284               0    0    0    1    0   1
Phalaenopsis-gene814419               1    1    1    1    1   5
Phalaenopsis-gene814643               1    0    1    1    0   3
Phalaenopsis-gene814644               0    0    0    0    0   0
Phalaenopsis-gene814708               1    0    0    1    0   2
Phalaenopsis-gene814745               1    0    0    1    0   2
Phalaenopsis-gene814781               1    0    0    1    0   2
Phalaenopsis-gene815110               1    0    0    1    0   2
Phalaenopsis-gene815119               0    0    0    1    0   1
Phalaenopsis-gene815260               1    0    0    1    0   2
Phalaenopsis-gene815261               1    1    1    1    0   4
Phalaenopsis-gene815664               1    1    1    1    1   5
Phalaenopsis-gene815719               0    0    0    1    0   1
Phalaenopsis-gene816283               1    0    1    1    0   3
Phalaenopsis-gene816821               1    1    1    1    1   5
Phalaenopsis-gene816825               1    1    1    1    1   5
Phalaenopsis-gene816836               1    0    1    0    0   2
Phalaenopsis-gene816837               1    0    1    1    0   3
Phalaenopsis-gene81707                1    1    1    1    1   5
Phalaenopsis-gene81725                1    0    1    0    0   2
Phalaenopsis-gene817567               1    0    1    0    0   2
Phalaenopsis-gene817664               1    1    1    1    1   5
Phalaenopsis-gene817665               1    0    1    0    0   2
Phalaenopsis-gene817845               1    1    1    1    1   5
Phalaenopsis-gene818236               0    0    1    1    0   2
Phalaenopsis-gene819271               1    1    0    0    0   2
Phalaenopsis-gene819273               1    1    0    1    0   3
Phalaenopsis-gene819274               1    1    1    1    1   5
Phalaenopsis-gene819477               1    1    1    1    1   5
Phalaenopsis-gene819480               0    0    1    1    0   2
Phalaenopsis-gene820037               1    1    1    1    1   5
Phalaenopsis-gene820063               1    1    1    1    0   4
Phalaenopsis-gene820353               1    0    0    1    0   2
Phalaenopsis-gene820354               1    1    0    0    0   2
Phalaenopsis-gene820710               1    0    1    1    0   3
Phalaenopsis-gene82096                1    0    0    1    0   2
Phalaenopsis-gene822589               0    0    0    0    0   0
Phalaenopsis-gene822590               1    1    1    1    1   5
Phalaenopsis-gene823709               0    0    1    1    0   2
Phalaenopsis-gene823712               1    0    1    1    0   3
Phalaenopsis-gene823759               1    0    1    1    0   3
Phalaenopsis-gene823781               1    0    1    1    0   3
Phalaenopsis-gene823853               1    1    1    1    0   4
Phalaenopsis-gene824644               0    0    0    1    0   1
Phalaenopsis-gene824883               1    0    1    0    0   2
Phalaenopsis-gene824885               1    0    1    0    0   2
Phalaenopsis-gene826070               1    1    1    1    1   5
Phalaenopsis-gene826071               1    1    0    1    0   3
Phalaenopsis-gene826101               1    0    1    0    0   2
Phalaenopsis-gene826156               0    0    0    1    0   1
Phalaenopsis-gene8262                 1    0    0    1    0   2
Phalaenopsis-gene826926               1    0    1    1    0   3
Phalaenopsis-gene8272                 0    0    1    1    0   2
Phalaenopsis-gene827224               1    0    0    1    0   2
Phalaenopsis-gene827225               1    1    1    1    1   5
Phalaenopsis-gene827244               1    0    0    1    0   2
Phalaenopsis-gene827439               1    1    1    1    1   5
Phalaenopsis-gene827467               1    1    1    1    1   5
Phalaenopsis-gene828527               1    0    0    1    0   2
Phalaenopsis-gene830491               1    1    1    1    1   5
Phalaenopsis-gene8305                 1    0    0    1    0   2
Phalaenopsis-gene830991               1    0    1    1    0   3
Phalaenopsis-gene831075               0    0    1    0    0   1
Phalaenopsis-gene831805               0    0    0    0    0   0
Phalaenopsis-gene831823               0    0    0    1    0   1
Phalaenopsis-gene832742               1    1    1    1    1   5
Phalaenopsis-gene833000               1    0    0    1    0   2
Phalaenopsis-gene833686               1    0    1    0    0   2
Phalaenopsis-gene833687               1    0    1    0    0   2
Phalaenopsis-gene833688               1    0    1    1    0   3
Phalaenopsis-gene833739               1    0    1    1    0   3
Phalaenopsis-gene833740               1    0    1    0    0   2
Phalaenopsis-gene833741               1    1    1    1    1   5
Phalaenopsis-gene834126               1    1    1    1    1   5
Phalaenopsis-gene83458                1    1    1    1    1   5
Phalaenopsis-gene83519                1    0    1    1    0   3
Phalaenopsis-gene83520                1    0    0    1    0   2
Phalaenopsis-gene835966               1    1    1    1    1   5
Phalaenopsis-gene835968               1    1    1    1    1   5
Phalaenopsis-gene836667               1    0    1    1    0   3
Phalaenopsis-gene836672               0    0    0    0    0   0
Phalaenopsis-gene836700               1    0    0    0    0   1
Phalaenopsis-gene836705               0    0    1    0    0   1
Phalaenopsis-gene836706               1    1    1    1    1   5
Phalaenopsis-gene837146               1    0    0    1    0   2
Phalaenopsis-gene837147               1    0    0    0    0   1
Phalaenopsis-gene837175               0    0    0    1    0   1
Phalaenopsis-gene837176               0    0    0    1    0   1
Phalaenopsis-gene837219               1    1    1    1    1   5
Phalaenopsis-gene837384               1    1    0    1    0   3
Phalaenopsis-gene838009               1    1    1    1    1   5
Phalaenopsis-gene838018               1    0    1    0    0   2
Phalaenopsis-gene838034               1    0    0    1    0   2
Phalaenopsis-gene838043               0    0    0    1    0   1
Phalaenopsis-gene83820                1    0    0    1    0   2
Phalaenopsis-gene838357               0    1    1    0    0   2
Phalaenopsis-gene838852               1    0    1    1    0   3
Phalaenopsis-gene839522               1    0    0    0    0   1
Phalaenopsis-gene839523               1    0    0    1    0   2
Phalaenopsis-gene839533               1    1    1    1    1   5
Phalaenopsis-gene839540               1    0    1    1    0   3
Phalaenopsis-gene839560               0    0    0    0    0   0
Phalaenopsis-gene839561               1    1    1    1    1   5
Phalaenopsis-gene839567               1    1    1    1    1   5
Phalaenopsis-gene839583               1    0    1    1    0   3
Phalaenopsis-gene839593               0    0    0    1    0   1
Phalaenopsis-gene839598               1    1    1    1    0   4
Phalaenopsis-gene839600               1    0    1    1    0   3
Phalaenopsis-gene839601               1    0    1    0    0   2
Phalaenopsis-gene839607               1    0    1    1    0   3
Phalaenopsis-gene839620               1    0    0    0    0   1
Phalaenopsis-gene839621               1    1    1    1    1   5
Phalaenopsis-gene839625               1    1    1    1    0   4
Phalaenopsis-gene839642               1    1    1    1    1   5
Phalaenopsis-gene839652               1    1    1    1    1   5
Phalaenopsis-gene839659               0    0    1    1    0   2
Phalaenopsis-gene839665               1    0    1    0    0   2
Phalaenopsis-gene839666               1    1    1    1    1   5
Phalaenopsis-gene839679               1    0    0    1    0   2
Phalaenopsis-gene839683               1    0    0    1    0   2
Ophrys-gene693205                     1    1    1    1    1   5
Ophrys-gene693245                     0    0    0    0    0   0
Ophrys-gene693260                     1    0    0    1    0   2
Ophrys-gene693267                     1    1    1    1    1   5
Ophrys-gene693537                     1    0    0    0    0   1
Ophrys-gene693542                     1    0    1    1    0   3
Ophrys-gene694366                     1    0    1    1    0   3
Ophrys-gene694605                     1    1    1    1    1   5
Ophrys-gene694834                     0    0    0    1    0   1
Ophrys-gene695748                     1    0    0    1    0   2
Ophrys-gene695797                     0    0    0    1    0   1
Ophrys-gene695815                     1    1    1    1    1   5
Ophrys-gene695852                     1    0    0    0    0   1
Ophrys-gene695908                     0    1    0    1    0   2
Ophrys-gene696056                     1    1    0    1    0   3
Ophrys-gene696117                     0    1    0    1    0   2
Ophrys-gene696144                     1    1    1    1    1   5
Ophrys-gene696165                     1    1    1    1    1   5
Ophrys-gene696833                     0    0    0    1    0   1
Ophrys-gene696834                     1    1    1    1    1   5
Ophrys-gene697157                     1    1    1    1    1   5
Ophrys-gene697158                     1    1    1    1    1   5
Ophrys-gene697180                     1    1    1    1    1   5
Ophrys-gene697181                     0    0    0    1    0   1
Ophrys-gene697215                     1    0    0    0    0   1
Ophrys-gene697238                     1    1    1    1    1   5
Ophrys-gene697239                     1    1    1    1    1   5
Ophrys-gene697296                     1    1    1    1    1   5
Ophrys-gene697330                     1    1    1    1    1   5
Ophrys-gene697331                     1    1    1    1    1   5
Ophrys-gene697353                     1    0    0    1    0   2
Ophrys-gene697354                     0    0    0    1    0   1
Ophrys-gene697387                     1    1    1    1    1   5
Ophrys-gene697410                     1    1    1    1    1   5
Ophrys-gene697411                     1    1    0    1    0   3
Ophrys-gene697447                     1    1    1    1    1   5
Ophrys-gene697470                     1    0    0    1    0   2
Ophrys-gene697471                     1    1    1    1    1   5
Ophrys-gene697524                     1    0    0    0    0   1
Ophrys-gene697557                     1    1    1    1    1   5
Ophrys-gene697558                     1    1    1    1    1   5
Ophrys-gene697580                     1    1    0    1    0   3
Ophrys-gene697581                     1    0    0    1    0   2
Ophrys-gene697616                     1    0    0    1    0   2
Ophrys-gene697639                     1    1    1    1    1   5
Ophrys-gene697640                     0    0    0    1    1   2
Ophrys-gene697723                     1    1    1    1    1   5
Ophrys-gene697725                     0    0    0    0    0   0
Ophrys-gene697748                     1    1    1    1    1   5
Ophrys-gene697749                     1    1    1    1    1   5
Ophrys-gene697780                     1    1    1    1    1   5
Ophrys-gene697803                     1    1    1    1    1   5
Ophrys-gene697804                     1    0    0    1    0   2
Ophrys-gene697893                     1    1    1    1    1   5
Ophrys-gene697916                     1    0    0    1    0   2
Ophrys-gene697917                     1    0    0    1    0   2
Ophrys-gene697947                     0    0    0    1    0   1
Ophrys-gene697970                     0    0    0    1    0   1
Ophrys-gene697971                     1    1    1    1    1   5
Ophrys-gene698261                     1    0    1    1    0   3
Ophrys-gene698632                     1    0    0    1    0   2
Ophrys-gene698739                     1    0    0    0    1   2
Ophrys-gene698767                     1    0    1    0    0   2
Ophrys-gene698842                     1    1    1    1    1   5
Ophrys-gene699130                     1    1    1    1    1   5
Ophrys-gene699131                     1    0    0    1    0   2
Ophrys-gene699520                     1    1    1    1    1   5
Ophrys-gene699522                     1    1    1    1    1   5
Ophrys-gene699852                     1    1    1    1    1   5
Ophrys-gene699853                     1    1    1    1    1   5
Ophrys-gene700022                     0    0    1    1    0   2
Ophrys-gene700023                     0    0    0    1    0   1
Ophrys-gene700559                     1    0    0    1    0   2
Ophrys-gene700568                     1    0    0    1    0   2
Ophrys-gene700913                     1    0    1    1    0   3
Ophrys-gene700916                     1    1    1    1    1   5
Ophrys-gene700920                     1    0    0    0    0   1
Ophrys-gene700921                     1    0    1    0    0   2
Ophrys-gene700937                     0    0    1    1    0   2
Ophrys-gene700994                     1    1    1    1    1   5
Ophrys-gene700999                     1    1    1    1    1   5
Ophrys-gene701001                     0    0    0    0    0   0
Ophrys-gene701002                     1    0    0    1    0   2
Ophrys-gene700978                     1    0    0    1    0   2
Ophrys-gene700979                     1    0    1    1    0   3
Ophrys-gene700982                     0    0    1    1    0   2
Ophrys-gene701038                     1    0    0    1    0   2
Ophrys-gene701011                     0    0    1    1    0   2
Ophrys-gene701016                     1    1    1    1    1   5
Ophrys-gene701566                     0    0    0    0    0   0
Ophrys-gene701567                     1    0    0    1    0   2
Ophrys-gene701563                     1    0    1    1    0   3
Ophrys-gene702033                     1    1    1    1    1   5
Ophrys-gene702087                     1    0    0    1    0   2
Ophrys-gene702092                     0    0    1    1    0   2
Ophrys-gene702175                     1    1    1    1    1   5
Ophrys-gene702181                     1    1    1    1    1   5
Ophrys-gene702182                     0    0    1    0    0   1
Ophrys-gene702665                     1    0    0    0    0   1
Ophrys-gene703118                     1    0    0    1    0   2
Ophrys-gene703149                     0    0    0    1    0   1
Ophrys-gene703202                     1    1    1    1    1   5
Ophrys-gene703210                     1    1    1    1    1   5
Ophrys-gene703211                     0    0    0    1    0   1
Ophrys-gene703284                     1    1    1    1    1   5
Ophrys-gene703533                     1    1    1    1    1   5
Ophrys-gene703607                     1    1    1    1    1   5
Ophrys-gene703608                     1    0    0    1    0   2
Ophrys-gene703663                     1    1    0    1    0   3
Ophrys-gene703664                     0    0    0    1    0   1
Ophrys-gene703995                     1    1    1    1    1   5
Ophrys-gene704567                     1    1    0    0    0   2
Ophrys-gene704568                     1    1    1    1    1   5
Ophrys-gene704940                     1    1    1    1    1   5
Ophrys-gene704972                     1    1    1    1    1   5
Ophrys-gene706130                     0    0    0    1    0   1
Ophrys-gene706131                     1    1    1    1    1   5
Ophrys-gene706384                     1    1    1    1    1   5
Ophrys-gene706397                     1    1    1    1    1   5
Ophrys-gene707135                     1    1    1    1    1   5
Ophrys-gene707331                     0    0    0    0    0   0
Ophrys-gene707463                     0    1    0    1    0   2
Ophrys-gene707464                     1    0    0    0    0   1
Ophrys-gene707697                     1    1    1    1    1   5
Ophrys-gene707698                     1    1    1    1    1   5
Ophrys-gene708093                     1    0    0    1    0   2
Ophrys-gene708081                     1    0    0    0    0   1
Ophrys-gene708643                     1    0    1    1    0   3
Ophrys-gene708698                     1    0    0    1    0   2
Ophrys-gene708974                     1    1    1    1    1   5
Ophrys-gene708968                     1    0    1    1    0   3
Ophrys-gene709104                     1    1    1    1    1   5
Ophrys-gene709160                     1    1    1    1    1   5
Ophrys-gene709234                     1    1    1    1    1   5
Ophrys-gene709235                     1    1    1    1    1   5
Ophrys-gene709238                     1    0    1    1    0   3
Ophrys-gene709312                     1    0    0    0    0   1
Ophrys-gene709658                     1    0    0    1    0   2
Ophrys-gene709660                     1    0    1    1    0   3
Ophrys-gene709661                     1    1    1    1    1   5
Ophrys-gene709665                     1    0    0    1    0   2
Ophrys-gene709671                     1    1    1    1    1   5
Ophrys-gene709642                     1    1    1    1    1   5
Ophrys-gene709654                     1    1    1    1    1   5
Ophrys-gene710465                     1    0    1    1    0   3
Ophrys-gene710546                     1    0    1    1    0   3
Ophrys-gene710711                     1    1    1    1    1   5
Ophrys-gene710698                     1    0    1    1    0   3
Ophrys-gene710977                     1    0    0    1    0   2
Ophrys-gene710979                     1    1    1    1    1   5
Ophrys-gene712146                     1    0    0    1    0   2
Ophrys-gene712229                     1    1    0    0    0   2
Ophrys-gene713854                     1    1    1    1    1   5
Ophrys-gene714340                     1    0    0    1    0   2
Ophrys-gene714328                     1    0    0    0    0   1
Ophrys-gene714331                     1    1    1    1    1   5
Ophrys-gene714804                     0    0    0    0    0   0
Ophrys-gene715208                     1    1    0    0    0   2
Ophrys-gene715314                     1    0    0    0    0   1
Ophrys-gene715388                     1    1    1    1    1   5
Ophrys-gene715390                     0    0    0    1    0   1
Ophrys-gene715393                     1    0    0    1    0   2
Ophrys-gene715364                     1    0    0    1    0   2
Ophrys-gene715600                     1    1    1    1    1   5
Ophrys-gene715601                     1    1    1    1    1   5
Ophrys-gene715824                     1    0    0    1    0   2
Ophrys-gene716044                     1    0    0    1    0   2
Ophrys-gene716045                     0    0    0    0    0   0
Ophrys-gene716423                     1    1    1    1    1   5
Ophrys-gene716405                     0    1    0    1    0   2
Ophrys-gene716438                     1    1    1    1    1   5
Ophrys-gene716443                     1    1    1    1    1   5
Ophrys-gene717190                     1    1    1    1    1   5
Ophrys-gene717191                     1    0    1    0    0   2
Ophrys-gene717192                     1    1    1    1    1   5
Ophrys-gene717181                     1    1    0    1    0   3
Ophrys-gene717185                     1    0    1    1    0   3
Ophrys-gene717300                     1    1    1    1    1   5
Ophrys-gene717312                     1    0    0    0    0   1
Ophrys-gene717353                     0    0    0    1    0   1
Ophrys-gene717365                     1    0    0    1    0   2
Ophrys-gene717366                     1    0    0    1    0   2
Ophrys-gene717413                     0    0    0    1    0   1
Ophrys-gene717401                     1    0    0    1    0   2
Ophrys-gene718047                     1    1    1    1    1   5
Ophrys-gene718188                     0    1    0    1    0   2
Ophrys-gene718189                     1    1    1    1    1   5
Ophrys-gene718190                     1    1    1    1    1   5
Ophrys-gene720195                     0    0    0    1    0   1
Ophrys-gene720196                     1    0    0    1    0   2
Ophrys-gene720222                     1    0    0    1    0   2
Ophrys-gene720223                     1    0    0    1    0   2
Ophrys-gene720975                     0    0    1    0    0   1
Ophrys-gene721693                     1    1    1    1    1   5
Ophrys-gene721854                     1    1    1    1    1   5
Ophrys-gene721855                     1    0    1    1    0   3
Ophrys-gene722695                     1    0    0    1    1   3
Ophrys-gene722698                     0    0    0    0    0   0
Ophrys-gene722667                     0    0    0    0    0   0
Ophrys-gene722668                     0    0    1    1    0   2
Ophrys-gene722775                     1    0    0    0    0   1
Ophrys-gene722784                     1    1    1    1    1   5
Ophrys-gene722785                     1    0    0    1    0   2
Ophrys-gene722765                     1    1    0    1    0   3
Ophrys-gene722766                     1    1    1    0    0   3
Ophrys-gene722883                     1    1    1    1    1   5
Ophrys-gene722884                     0    0    1    1    0   2
Ophrys-gene722886                     1    0    0    1    0   2
Ophrys-gene722887                     1    0    0    0    0   1
Ophrys-gene724436                     1    1    1    1    1   5
Ophrys-gene724542                     1    1    1    1    1   5
Ophrys-gene724544                     1    1    1    1    1   5
Ophrys-gene724546                     1    0    1    0    0   2
Ophrys-gene726152                     0    1    0    0    0   1
Ophrys-gene726162                     0    0    0    1    0   1
Ophrys-gene726163                     1    1    1    1    1   5
Ophrys-gene726200                     0    0    0    0    0   0
Ophrys-gene726201                     0    1    0    1    0   2
Ophrys-gene726342                     1    1    0    1    0   3
Ophrys-gene726453                     1    0    0    0    0   1
Ophrys-gene726463                     1    0    0    1    0   2
Ophrys-gene726733                     1    1    1    1    1   5
Ophrys-gene727264                     1    0    0    1    0   2
Ophrys-gene727265                     1    0    0    1    0   2
Ophrys-gene727251                     1    0    0    1    0   2
Ophrys-gene727252                     1    1    1    1    1   5
Ophrys-gene727257                     1    0    0    1    0   2
Ophrys-gene727258                     1    0    0    1    0   2
Ophrys-gene727292                     1    1    1    1    1   5
Ophrys-gene727799                     1    1    1    1    1   5
Ophrys-gene727851                     1    1    1    1    1   5
Ophrys-gene727855                     1    1    1    1    1   5
Ophrys-gene727850                     1    1    1    1    1   5
Ophrys-gene727897                     1    1    1    1    1   5
Ophrys-gene727893                     1    1    1    1    1   5
Ophrys-gene727950                     1    1    1    1    1   5
Ophrys-gene729696                     1    0    0    1    0   2
Ophrys-gene729702                     1    1    1    1    1   5
Ophrys-gene729695                     0    0    1    1    0   2
Ophrys-gene729904                     1    0    0    1    0   2
Ophrys-gene730043                     1    0    0    0    0   1
Ophrys-gene730157                     1    0    0    1    0   2
Ophrys-gene730210                     0    0    0    1    0   1
Ophrys-gene730262                     1    0    0    1    0   2
Ophrys-gene730263                     0    0    0    1    0   1
Ophrys-gene730289                     1    0    0    0    0   1
Ophrys-gene730637                     1    0    1    1    0   3
Ophrys-gene730670                     1    1    1    1    1   5
Ophrys-gene730975                     1    1    1    1    1   5
Ophrys-gene731401                     1    1    1    1    1   5
Ophrys-gene731655                     1    1    0    1    0   3
Ophrys-gene732058                     1    0    0    1    0   2
Ophrys-gene735541                     1    1    1    1    1   5
Ophrys-gene735553                     1    1    1    1    1   5
Ophrys-gene735817                     1    1    1    1    1   5
Ophrys-gene736625                     1    1    1    1    1   5
Ophrys-gene736626                     0    0    1    1    0   2
Ophrys-gene737949                     1    1    1    1    1   5
Ophrys-gene737950                     1    1    1    1    1   5
Ophrys-gene737956                     1    0    0    1    0   2
Ophrys-gene737957                     1    1    1    1    1   5
Ophrys-gene738995                     1    0    0    1    0   2
Ophrys-gene738996                     1    0    0    1    0   2
Ophrys-gene739300                     1    0    0    1    0   2
Ophrys-gene739660                     0    0    0    1    0   1
Ophrys-gene739646                     1    1    1    1    1   5
Ophrys-gene739654                     1    1    1    1    1   5
Ophrys-gene739694                     0    0    1    1    0   2
Ophrys-gene739680                     1    1    1    1    1   5
Ophrys-gene739882                     1    1    1    1    0   4
Ophrys-gene742033                     1    1    1    1    1   5
Ophrys-gene742057                     0    0    1    1    0   2
Ophrys-gene742061                     1    1    1    1    1   5
Ophrys-gene746530                     1    1    1    1    1   5
Ophrys-gene746806                     1    1    1    1    1   5
Ophrys-gene747141                     1    1    0    0    1   3
Ophrys-gene747137                     1    0    0    1    0   2
Ophrys-gene747175                     1    0    0    1    0   2
Ophrys-gene747171                     1    1    1    1    1   5
Ophrys-gene747672                     1    0    0    0    1   2
Ophrys-gene747682                     0    1    0    1    0   2
Ophrys-gene747671                     1    0    0    0    0   1
Ophrys-gene748105                     1    1    1    1    1   5
Ophrys-gene748140                     1    1    1    1    1   5
Ophrys-gene748212                     1    0    1    1    0   3
Ophrys-gene748904                     1    0    0    0    0   1
Ophrys-gene750133                     0    0    0    1    0   1
Ophrys-gene750159                     1    1    1    1    1   5
Ophrys-gene753183                     1    0    0    1    0   2
Ophrys-gene753289                     1    0    0    1    0   2
Ophrys-gene753297                     1    0    1    1    0   3
Ophrys-gene753428                     1    0    0    1    0   2
Ophrys-gene753530                     1    0    1    1    0   3
Ophrys-gene754877                     0    0    0    1    0   1
Ophrys-gene754878                     0    0    1    1    0   2
Ophrys-gene755142                     1    1    1    1    1   5
Ophrys-gene755151                     1    0    1    1    0   3
Ophrys-gene757438                     1    0    0    1    0   2
Ophrys-gene757439                     1    0    0    1    0   2
Ophrys-gene757498                     1    0    0    1    0   2
Ophrys-gene757499                     1    0    0    0    0   1
Ophrys-gene757558                     1    0    0    1    0   2
Ophrys-gene757733                     1    0    0    1    0   2
Ophrys-gene757734                     1    0    0    1    0   2
Ophrys-gene758065                     1    1    1    1    1   5
Ophrys-gene758217                     1    1    0    0    0   2
Ophrys-gene758572                     0    0    0    0    0   0
Ophrys-gene758667                     1    0    0    1    0   2
Ophrys-gene758852                     1    0    0    1    0   2
Ophrys-gene759076                     1    1    0    1    0   3
Ophrys-gene759357                     1    0    0    1    0   2
Ophrys-gene759492                     0    0    0    1    0   1
Ophrys-gene760393                     1    0    1    0    1   3
Ophrys-gene760394                     1    1    1    1    1   5
Ophrys-gene760436                     1    0    0    0    0   1
Ophrys-gene761532                     1    0    0    0    0   1
Ophrys-gene761533                     0    0    1    1    0   2
Ophrys-gene761569                     0    0    0    1    0   1
Ophrys-gene761570                     1    1    1    1    1   5
Ophrys-gene762733                     1    0    0    1    0   2
Ophrys-gene762855                     1    0    0    1    0   2
Ophrys-gene762861                     1    1    1    1    1   5
Ophrys-gene762866                     1    1    1    1    1   5
Ophrys-gene762867                     1    0    0    0    0   1
Ophrys-gene762983                     1    0    0    0    0   1
Ophrys-gene762984                     1    0    0    1    0   2
Ophrys-gene762988                     1    1    1    1    1   5
Ophrys-gene762989                     1    0    1    1    0   3
Ophrys-gene762976                     1    1    1    1    0   4
Ophrys-gene762977                     0    0    0    0    0   0
Ophrys-gene763321                     1    1    1    0    0   3
Ophrys-gene763322                     1    0    1    0    0   2
Ophrys-gene763360                     1    0    1    1    0   3
Ophrys-gene763361                     0    0    0    0    0   0
Ophrys-gene763466                     0    1    0    1    0   2
Ophrys-gene763581                     1    1    1    1    1   5
Ophrys-gene764814                     1    1    1    1    1   5
Ophrys-gene765010                     1    1    1    1    1   5
Ophrys-gene766653                     1    1    1    1    1   5
Ophrys-gene768849                     1    1    1    1    1   5
Ophrys-gene768891                     1    1    1    1    1   5
Ophrys-gene768892                     0    0    0    1    0   1
Ophrys-gene768946                     1    1    1    1    1   5
Ophrys-gene769421                     1    1    1    1    1   5
Ophrys-gene436368                     1    1    1    1    1   5
Ophrys-gene436174                     1    0    0    0    0   1
Ophrys-gene332730                     0    1    0    1    0   2
Ophrys-gene203602                     0    1    0    1    0   2
Ophrys-gene117163                     1    1    1    1    1   5
Ophrys-gene634136                     1    0    0    1    0   2
Ophrys-gene231607                     1    0    0    1    0   2
Ophrys-gene644205                     1    1    1    1    1   5
Ophrys-gene528710                     1    1    1    1    1   5
Ophrys-gene275025                     1    0    0    0    0   1
Ophrys-gene136269                     1    0    0    1    0   2
Ophrys-gene121376                     0    0    0    1    0   1
Ophrys-gene51459                      1    1    1    1    1   5
Ophrys-gene11523                      1    1    1    1    1   5
Ophrys-gene429182                     1    0    0    1    0   2
Ophrys-gene76259                      1    1    1    1    1   5
Ophrys-gene655006                     1    1    1    1    1   5
Ophrys-gene467389                     1    0    0    0    0   1
Ophrys-gene392619                     1    0    0    0    0   1
Ophrys-gene355138                     1    0    0    0    0   1
Ophrys-gene322283                     0    0    0    0    0   0
Ophrys-gene225946                     1    1    1    1    1   5
Ophrys-gene178710                     0    0    0    0    0   0
Ophrys-gene67770                      1    1    1    1    1   5
Ophrys-gene430979                     1    1    1    1    1   5
Ophrys-gene433745                     1    1    1    1    1   5
Ophrys-gene355066                     1    1    1    1    1   5
Ophrys-gene231677                     1    0    0    1    0   2
Ophrys-gene602942                     1    1    1    1    1   5
Ophrys-gene724543                     0    0    0    1    1   2
Ophrys-gene715772                     1    1    1    1    1   5
Ophrys-gene708699                     1    0    0    1    0   2
Ophrys-gene693197                     1    0    0    1    0   2
Ophrys-gene532852                     1    0    0    1    0   2
Ophrys-gene422291                     1    0    0    0    0   1
Ophrys-gene419281                     1    1    1    1    1   5
Ophrys-gene354992                     0    0    0    0    0   0
Ophrys-gene274857                     1    1    1    1    1   5
Ophrys-gene177730                     1    1    0    0    0   2
Ophrys-gene142506                     1    1    1    1    1   5
Ophrys-gene11808                      1    0    0    1    0   2
Ophrys-gene7125                       1    0    1    0    0   2
Ophrys-gene1574                       1    1    1    1    1   5
Ophrys-gene1316                       1    1    1    1    1   5
Ophrys-gene449813                     1    1    1    1    1   5
Ophrys-gene221640                     1    1    1    1    1   5
Ophrys-gene536629                     0    0    1    1    0   2
Ophrys-gene414151                     0    1    0    1    0   2
Ophrys-gene190405                     0    0    0    1    0   1
Ophrys-gene137682                     1    0    1    1    0   3
Ophrys-gene419339                     1    1    1    1    1   5
Ophrys-gene332762                     1    0    0    1    0   2
Ophrys-gene306828                     1    1    0    0    0   2
Ophrys-gene297221                     1    1    1    1    1   5
Ophrys-gene177729                     1    0    0    0    0   1
Ophrys-gene579840                     1    0    0    1    0   2
Ophrys-gene296684                     1    0    0    1    0   2
Ophrys-gene274856                     1    1    1    1    1   5
Ophrys-gene190238                     0    0    0    1    0   1
Ophrys-gene136268                     1    0    0    1    0   2
Ophrys-gene46894                      1    1    1    1    1   5
Ophrys-gene23526                      1    1    1    1    1   5
Ophrys-gene570501                     1    1    1    1    1   5
Ophrys-gene440971                     0    1    0    1    0   2
Ophrys-gene313763                     1    0    0    0    0   1
Ophrys-gene271473                     0    0    0    1    0   1
Ophrys-gene65169                      1    1    1    1    1   5
Ophrys-gene378356                     1    1    1    1    1   5
Ophrys-gene378311                     0    0    0    0    0   0
Ophrys-gene137823                     0    0    1    1    0   2
Ophrys-gene483951                     1    0    1    1    0   3
Ophrys-gene185106                     0    0    0    1    0   1
Ophrys-gene339408                     1    1    1    1    1   5
Ophrys-gene288588                     0    0    1    0    0   1
Ophrys-gene205706                     1    1    1    1    1   5
Ophrys-gene288424                     1    0    1    0    0   2
Ophrys-gene629887                     1    0    0    1    0   2
Ophrys-gene490470                     1    0    0    1    0   2
Ophrys-gene440396                     1    1    1    1    1   5
Ophrys-gene341048                     1    1    1    1    1   5
Ophrys-gene337423                     1    1    1    1    1   5
Ophrys-gene251048                     1    0    0    1    0   2
Ophrys-gene231659                     1    0    0    0    0   1
Ophrys-gene180183                     1    0    0    1    0   2
Ophrys-gene167687                     1    1    1    1    1   5
Ophrys-gene143572                     1    1    1    1    1   5
Ophrys-gene137650                     1    1    0    1    0   3
Ophrys-gene107286                     1    0    0    1    0   2
Ophrys-gene2040                       1    0    0    0    0   1
Ophrys-gene60672                      1    0    1    1    0   3
Ophrys-gene178619                     0    0    0    0    0   0
Ophrys-gene258609                     0    0    0    1    0   1
Ophrys-gene284331                     1    1    1    1    1   5
Ophrys-gene303750                     0    0    1    0    0   1
Ophrys-gene312980                     1    1    0    0    0   2
Ophrys-gene314059                     1    0    0    1    0   2
Ophrys-gene336279                     1    0    0    1    0   2
Ophrys-gene377170                     1    0    0    1    0   2
Ophrys-gene419222                     1    1    1    1    1   5
Ophrys-gene419253                     1    0    0    0    0   1
Ophrys-gene441053                     0    0    0    1    0   1
Ophrys-gene461890                     1    1    1    1    1   5
Ophrys-gene504480                     1    1    1    1    1   5
Ophrys-gene504466                     1    1    1    1    1   5
Ophrys-gene580010                     1    1    1    1    1   5
Ophrys-gene597822                     1    0    0    0    0   1
Ophrys-gene597946                     1    1    1    1    1   5
Ophrys-gene717182                     1    1    1    1    1   5
Ophrys-gene727892                     1    0    0    1    0   2
Ophrys-gene739885                     1    1    1    1    1   5
Ophrys-gene748211                     0    0    0    0    1   1
Ophrys-gene378437                     1    1    1    1    1   5
Ophrys-gene676972                     1    0    1    1    1   4
Ophrys-gene736655                     0    0    1    1    1   3
Ophrys-gene582451                     1    1    1    1    1   5
Ophrys-gene579923                     1    1    1    1    1   5
Ophrys-gene449814                     1    0    0    1    0   2
Ophrys-gene409832                     0    0    0    1    0   1
Ophrys-gene353521                     1    1    1    1    1   5
Ophrys-gene340145                     0    0    0    1    0   1
Ophrys-gene38261                      0    0    1    1    0   2
Ophrys-gene108033                     1    1    1    1    1   5
Ophrys-gene132442                     1    1    1    1    1   5
Ophrys-gene159908                     1    1    1    1    1   5
Ophrys-gene214739                     1    0    0    1    0   2
Ophrys-gene253321                     1    1    1    1    1   5
Ophrys-gene278911                     1    0    1    0    0   2
Ophrys-gene300512                     1    1    1    1    1   5
Ophrys-gene314226                     1    1    0    1    0   3
Ophrys-gene337496                     1    1    1    1    1   5
Ophrys-gene404895                     1    0    1    1    0   3
Ophrys-gene404979                     1    1    1    1    1   5
Ophrys-gene419163                     1    0    0    1    0   2
Ophrys-gene458119                     1    0    0    0    0   1
Ophrys-gene740786                     1    1    1    1    1   5
Phalaenopsis-gene839684               1    0    1    1    0   3
Phalaenopsis-gene839698               1    0    0    1    0   2
Phalaenopsis-gene839699               0    0    0    0    0   0
Phalaenopsis-gene839709               1    1    1    1    1   5
Phalaenopsis-gene839716               0    0    1    1    0   2
Phalaenopsis-gene839717               1    0    1    0    0   2
Phalaenopsis-gene839736               0    0    0    1    0   1
Phalaenopsis-gene839743               1    0    1    1    0   3
Phalaenopsis-gene839756               0    1    1    0    0   2
Phalaenopsis-gene839766               1    0    0    1    0   2
Phalaenopsis-gene839771               1    0    1    1    0   3
Phalaenopsis-gene839773               1    0    1    0    0   2
Phalaenopsis-gene839774               1    0    0    0    0   1
Phalaenopsis-gene839793               1    1    1    1    1   5
Phalaenopsis-gene839798               1    0    1    1    0   3
Phalaenopsis-gene839800               1    0    1    1    0   3
Phalaenopsis-gene839808               0    0    0    1    0   1
Phalaenopsis-gene839809               0    0    1    1    0   2
Phalaenopsis-gene839819               0    0    1    0    0   1
Phalaenopsis-gene839824               1    1    1    1    1   5
Phalaenopsis-gene839826               1    0    1    0    0   2
Phalaenopsis-gene839827               0    0    0    1    0   1
Phalaenopsis-gene839833               1    0    1    0    0   2
Phalaenopsis-gene839846               1    0    0    1    0   2
Phalaenopsis-gene839847               0    0    1    1    0   2
Phalaenopsis-gene839851               1    1    1    1    0   4
Phalaenopsis-gene839972               1    0    0    1    0   2
Phalaenopsis-gene839991               1    0    0    1    0   2
Phalaenopsis-gene839992               1    0    0    1    0   2
Phalaenopsis-gene840082               0    0    0    0    0   0
Phalaenopsis-gene840083               1    1    1    1    0   4
Phalaenopsis-gene84016                1    0    0    1    1   3
Phalaenopsis-gene840429               1    0    0    1    0   2
Phalaenopsis-gene841134               1    1    0    1    0   3
Phalaenopsis-gene841135               1    0    0    1    0   2
Phalaenopsis-gene841140               1    1    1    0    1   4
Phalaenopsis-gene841516               1    0    1    1    0   3
Phalaenopsis-gene841520               1    0    1    1    0   3
Phalaenopsis-gene841527               1    0    1    1    0   3
Phalaenopsis-gene841834               1    1    1    1    1   5
Phalaenopsis-gene84232                1    1    1    1    1   5
Phalaenopsis-gene84246                0    1    1    1    0   3
Phalaenopsis-gene842832               0    1    1    1    0   3
Phalaenopsis-gene842834               1    0    0    1    0   2
Phalaenopsis-gene842872               1    0    1    1    0   3
Phalaenopsis-gene842873               1    0    1    0    0   2
Phalaenopsis-gene842874               1    0    1    1    0   3
Phalaenopsis-gene842876               0    1    0    1    0   2
Phalaenopsis-gene842915               1    0    0    1    0   2
Phalaenopsis-gene842916               1    1    1    0    0   3
Phalaenopsis-gene842917               1    0    1    1    0   3
Phalaenopsis-gene842919               1    0    0    1    0   2
Phalaenopsis-gene843490               1    0    1    0    0   2
Phalaenopsis-gene843519               1    0    0    0    0   1
Phalaenopsis-gene843955               1    1    1    1    1   5
Phalaenopsis-gene843958               1    1    1    1    1   5
Phalaenopsis-gene843959               1    1    1    1    1   5
Phalaenopsis-gene843973               1    1    1    1    1   5
Phalaenopsis-gene843987               1    1    1    1    1   5
Phalaenopsis-gene843990               1    1    1    1    0   4
Phalaenopsis-gene843991               1    1    1    1    1   5
Phalaenopsis-gene844005               1    1    1    1    1   5
Phalaenopsis-gene844832               1    1    1    1    1   5
Phalaenopsis-gene844984               1    1    1    1    1   5
Phalaenopsis-gene845369               1    1    1    1    1   5
Phalaenopsis-gene845719               1    0    0    1    0   2
Phalaenopsis-gene845732               1    0    0    1    0   2
Phalaenopsis-gene845733               0    0    0    1    0   1
Phalaenopsis-gene845734               1    0    0    1    0   2
Phalaenopsis-gene845735               1    0    1    1    0   3
Phalaenopsis-gene845754               0    0    0    1    0   1
Phalaenopsis-gene845767               1    0    0    0    0   1
Phalaenopsis-gene845768               1    1    1    1    1   5
Phalaenopsis-gene845770               1    0    1    1    0   3
Phalaenopsis-gene845789               0    0    1    1    0   2
Phalaenopsis-gene845790               1    0    0    1    0   2
Phalaenopsis-gene845803               0    0    1    0    0   1
Phalaenopsis-gene845805               0    0    1    1    0   2
Phalaenopsis-gene845806               0    1    0    0    0   1
Phalaenopsis-gene845825               1    1    1    1    1   5
Phalaenopsis-gene845838               1    0    1    1    0   3
Phalaenopsis-gene845839               1    0    1    1    0   3
Phalaenopsis-gene845841               1    1    1    1    1   5
Phalaenopsis-gene846095               0    0    0    1    0   1
Phalaenopsis-gene846394               1    0    0    0    1   2
Phalaenopsis-gene846608               0    0    1    0    0   1
Phalaenopsis-gene846646               1    0    0    1    0   2
Phalaenopsis-gene847265               1    1    1    1    1   5
Phalaenopsis-gene84761                1    0    1    1    1   4
Phalaenopsis-gene84762                1    1    1    1    1   5
Phalaenopsis-gene848026               1    0    0    1    0   2
Phalaenopsis-gene848075               1    1    1    1    1   5
Phalaenopsis-gene84820                1    0    0    1    0   2
Phalaenopsis-gene84830                1    1    1    1    1   5
Phalaenopsis-gene84849                1    1    1    1    1   5
Phalaenopsis-gene84851                1    1    1    1    1   5
Phalaenopsis-gene84852                1    1    1    1    1   5
Phalaenopsis-gene848546               1    0    0    1    0   2
Phalaenopsis-gene848830               1    0    0    0    0   1
Phalaenopsis-gene849104               1    0    1    1    0   3
Phalaenopsis-gene84922                1    0    0    0    0   1
Phalaenopsis-gene849223               1    0    0    0    0   1
Phalaenopsis-gene849771               1    0    1    1    0   3
Phalaenopsis-gene850709               1    1    1    1    1   5
Phalaenopsis-gene850851               1    1    1    1    1   5
Phalaenopsis-gene850941               1    1    1    1    1   5
Phalaenopsis-gene851031               1    0    1    0    0   2
Phalaenopsis-gene851476               0    0    0    1    0   1
Phalaenopsis-gene851496               0    0    0    1    0   1
Phalaenopsis-gene851537               0    0    0    1    0   1
Phalaenopsis-gene851597               1    0    1    1    0   3
Phalaenopsis-gene851609               1    0    0    1    0   2
Phalaenopsis-gene851697               1    1    1    1    1   5
Phalaenopsis-gene851698               1    1    1    1    0   4
Phalaenopsis-gene851709               0    0    0    1    0   1
Phalaenopsis-gene851710               1    0    0    1    0   2
Phalaenopsis-gene852460               1    1    1    1    1   5
Phalaenopsis-gene852461               1    0    0    1    0   2
Phalaenopsis-gene852464               1    1    1    1    1   5
Phalaenopsis-gene852620               1    1    1    1    1   5
Phalaenopsis-gene852750               1    1    1    1    1   5
Phalaenopsis-gene852751               1    1    1    1    1   5
Phalaenopsis-gene853548               1    1    1    1    0   4
Phalaenopsis-gene854753               1    0    0    1    0   2
Phalaenopsis-gene855377               1    0    0    0    1   2
Phalaenopsis-gene855392               1    0    0    1    0   2
Phalaenopsis-gene855901               0    0    1    1    0   2
Phalaenopsis-gene856113               1    0    0    1    0   2
Phalaenopsis-gene856130               0    0    1    1    0   2
Phalaenopsis-gene856712               1    1    1    1    1   5
Phalaenopsis-gene856723               1    1    1    1    1   5
Phalaenopsis-gene856724               1    0    1    0    0   2
Phalaenopsis-gene856729               1    0    0    0    0   1
Phalaenopsis-gene856828               1    1    0    1    0   3
Phalaenopsis-gene856859               0    0    1    0    0   1
Phalaenopsis-gene857239               1    0    0    1    0   2
Phalaenopsis-gene857603               0    0    1    0    0   1
Phalaenopsis-gene859278               1    1    0    1    0   3
Phalaenopsis-gene859294               1    0    0    1    0   2
Phalaenopsis-gene859309               1    1    1    1    1   5
Phalaenopsis-gene859321               1    0    0    1    0   2
Phalaenopsis-gene859402               1    1    1    1    1   5
Phalaenopsis-gene859404               1    0    0    0    0   1
Phalaenopsis-gene859405               1    0    1    1    0   3
Phalaenopsis-gene859408               1    0    1    1    0   3
Phalaenopsis-gene859512               1    0    0    1    0   2
Phalaenopsis-gene859548               0    0    0    1    0   1
Phalaenopsis-gene859559               0    0    0    1    0   1
Phalaenopsis-gene859606               1    1    1    1    0   4
Phalaenopsis-gene859633               1    0    0    1    0   2
Phalaenopsis-gene860001               1    1    1    1    1   5
Phalaenopsis-gene860151               0    1    0    1    0   2
Phalaenopsis-gene860193               1    0    1    1    0   3
Phalaenopsis-gene860237               1    0    1    0    0   2
Phalaenopsis-gene860412               1    0    1    1    0   3
Phalaenopsis-gene860680               1    1    1    1    1   5
Phalaenopsis-gene860967               1    1    1    1    1   5
Phalaenopsis-gene860968               1    0    0    1    0   2
Phalaenopsis-gene862157               1    0    1    1    0   3
Phalaenopsis-gene862193               1    0    0    1    0   2
Phalaenopsis-gene862229               0    0    0    1    0   1
Phalaenopsis-gene862349               1    0    1    1    0   3
Phalaenopsis-gene862360               1    1    1    1    1   5
Phalaenopsis-gene864730               0    0    0    1    0   1
Phalaenopsis-gene865567               1    1    1    1    1   5
Phalaenopsis-gene865588               1    0    1    1    0   3
Phalaenopsis-gene866427               1    1    1    1    1   5
Phalaenopsis-gene866834               1    1    1    1    1   5
Phalaenopsis-gene866835               1    1    1    1    1   5
Phalaenopsis-gene867432               0    0    0    0    0   0
Phalaenopsis-gene867439               1    1    1    1    1   5
Phalaenopsis-gene867441               1    0    1    1    0   3
Phalaenopsis-gene867448               1    0    0    0    0   1
Phalaenopsis-gene867449               1    1    1    1    1   5
Phalaenopsis-gene867480               0    0    0    1    0   1
Phalaenopsis-gene868264               1    1    1    1    0   4
Phalaenopsis-gene868295               0    0    0    0    0   0
Phalaenopsis-gene868326               1    0    0    1    0   2
Phalaenopsis-gene868327               1    0    1    0    0   2
Phalaenopsis-gene871015               0    0    1    1    0   2
Phalaenopsis-gene871569               1    0    0    1    0   2
Phalaenopsis-gene871573               1    0    0    0    0   1
Phalaenopsis-gene871578               0    0    1    1    0   2
Phalaenopsis-gene871597               1    0    0    1    0   2
Phalaenopsis-gene872369               1    0    1    1    0   3
Phalaenopsis-gene872947               1    1    1    1    1   5
Phalaenopsis-gene873130               1    1    1    1    1   5
Phalaenopsis-gene873607               0    0    0    1    0   1
Phalaenopsis-gene873679               1    0    0    1    0   2
Phalaenopsis-gene874704               1    1    1    1    1   5
Phalaenopsis-gene875206               1    0    0    0    0   1
Phalaenopsis-gene875311               1    1    1    1    1   5
Phalaenopsis-gene875312               1    0    0    1    1   3
Phalaenopsis-gene875484               1    0    0    1    0   2
Phalaenopsis-gene876510               0    1    0    0    0   1
Phalaenopsis-gene876513               1    0    0    1    0   2
Phalaenopsis-gene876676               1    0    1    0    0   2
Phalaenopsis-gene876679               0    0    0    1    0   1
Phalaenopsis-gene876725               1    0    1    0    0   2
Phalaenopsis-gene877579               1    0    1    0    0   2
Phalaenopsis-gene878078               1    0    0    1    0   2
Phalaenopsis-gene878386               1    0    0    1    0   2
Phalaenopsis-gene878723               1    1    1    1    1   5
Phalaenopsis-gene879640               1    0    1    1    0   3
Phalaenopsis-gene879648               1    0    0    0    0   1
Phalaenopsis-gene879793               1    1    1    1    1   5
Phalaenopsis-gene880829               0    0    1    0    0   1
Phalaenopsis-gene880854               1    1    1    1    1   5
Phalaenopsis-gene881616               0    0    1    0    0   1
Phalaenopsis-gene881804               1    1    1    1    1   5
Phalaenopsis-gene881824               1    0    1    0    0   2
Phalaenopsis-gene881852               1    0    0    1    0   2
Phalaenopsis-gene881905               1    0    0    1    0   2
Phalaenopsis-gene881906               0    0    0    1    0   1
Phalaenopsis-gene882087               1    0    1    0    0   2
Phalaenopsis-gene882088               1    0    1    0    0   2
Phalaenopsis-gene882145               1    0    1    1    0   3
Phalaenopsis-gene882304               0    0    0    0    0   0
Phalaenopsis-gene882351               1    0    0    1    0   2
Phalaenopsis-gene882559               1    1    0    0    0   2
Phalaenopsis-gene882602               1    0    1    0    0   2
Phalaenopsis-gene883018               1    0    1    1    0   3
Phalaenopsis-gene883019               1    1    1    1    1   5
Phalaenopsis-gene883828               1    1    1    1    1   5
Phalaenopsis-gene883829               1    1    1    1    1   5
Phalaenopsis-gene884497               0    0    1    1    0   2
Phalaenopsis-gene884729               1    1    1    1    1   5
Phalaenopsis-gene884766               1    0    1    1    1   4
Phalaenopsis-gene884773               0    0    1    0    0   1
Phalaenopsis-gene88502                1    0    1    1    0   3
Phalaenopsis-gene885061               0    0    1    0    0   1
Phalaenopsis-gene88531                1    1    0    1    0   3
Phalaenopsis-gene88532                1    1    0    1    0   3
Phalaenopsis-gene885718               1    1    0    0    0   2
Phalaenopsis-gene887106               1    1    0    1    0   3
Phalaenopsis-gene887114               0    0    0    0    0   0
Phalaenopsis-gene887115               1    1    1    1    1   5
Phalaenopsis-gene887152               1    0    0    0    0   1
Phalaenopsis-gene887155               1    1    1    1    1   5
Phalaenopsis-gene887191               1    1    1    1    1   5
Phalaenopsis-gene887200               0    0    1    1    1   3
Phalaenopsis-gene887245               1    0    0    1    0   2
Phalaenopsis-gene887292               0    0    0    1    0   1
Phalaenopsis-gene887293               1    0    1    1    1   4
Phalaenopsis-gene887465               1    1    1    1    1   5
Phalaenopsis-gene887466               1    1    1    1    1   5
Phalaenopsis-gene887534               1    1    1    1    1   5
Phalaenopsis-gene887535               1    0    1    1    0   3
Phalaenopsis-gene887540               1    1    1    1    1   5
Phalaenopsis-gene888190               0    0    1    0    0   1
Phalaenopsis-gene888191               0    0    1    1    0   2
Phalaenopsis-gene888195               1    0    0    0    1   2
Phalaenopsis-gene888196               1    1    1    1    0   4
Phalaenopsis-gene888204               1    0    1    1    0   3
Phalaenopsis-gene888214               1    0    1    1    0   3
Phalaenopsis-gene888218               1    0    0    1    0   2
Phalaenopsis-gene888243               1    0    1    1    0   3
Phalaenopsis-gene888270               1    1    1    1    1   5
Phalaenopsis-gene888271               1    0    1    0    0   2
Phalaenopsis-gene888381               1    1    1    1    1   5
Phalaenopsis-gene888945               1    1    1    1    1   5
Phalaenopsis-gene888952               1    0    0    1    0   2
Phalaenopsis-gene889537               1    1    1    1    1   5
Phalaenopsis-gene889540               1    0    1    1    0   3
Phalaenopsis-gene88999                1    1    1    1    1   5
Phalaenopsis-gene89021                0    0    1    1    0   2
Phalaenopsis-gene89033                0    0    1    1    0   2
Phalaenopsis-gene89052                0    0    1    0    0   1
Phalaenopsis-gene89064                0    0    1    1    0   2
Phalaenopsis-gene890856               1    1    1    1    1   5
Phalaenopsis-gene890860               1    1    1    1    1   5
Phalaenopsis-gene8909                 1    0    0    1    0   2
Phalaenopsis-gene89135                1    1    1    1    1   5
Phalaenopsis-gene892022               1    1    1    1    1   5
Phalaenopsis-gene892465               1    0    0    1    0   2
Phalaenopsis-gene892471               1    1    0    1    0   3
Phalaenopsis-gene89250                0    0    0    0    0   0
Phalaenopsis-gene89261                1    1    1    1    1   5
Phalaenopsis-gene89273                0    0    0    0    0   0
Phalaenopsis-gene89327                0    1    1    1    0   3
Phalaenopsis-gene893294               1    1    1    1    1   5
Phalaenopsis-gene893467               1    0    0    0    0   1
Phalaenopsis-gene893499               1    0    1    0    0   2
Phalaenopsis-gene893542               1    0    1    1    0   3
Phalaenopsis-gene893819               1    0    1    1    0   3
Phalaenopsis-gene89382                0    0    1    1    0   2
Phalaenopsis-gene893822               1    0    1    0    1   3
Phalaenopsis-gene893823               1    0    0    0    0   1
Phalaenopsis-gene893851               1    0    0    0    0   1
Phalaenopsis-gene893978               1    1    1    1    1   5
Phalaenopsis-gene89430                0    0    1    0    0   1
Phalaenopsis-gene894340               1    1    1    0    0   3
Phalaenopsis-gene89484                1    0    1    1    0   3
Phalaenopsis-gene89485                1    0    0    1    0   2
Phalaenopsis-gene895164               1    0    1    0    0   2
Phalaenopsis-gene895174               0    0    1    0    0   1
Phalaenopsis-gene895175               1    0    1    0    0   2
Phalaenopsis-gene895179               1    1    1    1    1   5
Phalaenopsis-gene895194               0    0    0    1    0   1
Phalaenopsis-gene895204               0    0    1    0    0   1
Phalaenopsis-gene895209               1    0    1    1    0   3
Phalaenopsis-gene89523                1    0    1    1    0   3
Phalaenopsis-gene895279               1    0    1    0    0   2
Phalaenopsis-gene896659               0    0    0    1    0   1
Phalaenopsis-gene896660               0    0    1    0    0   1
Phalaenopsis-gene897243               0    1    1    1    0   3
Phalaenopsis-gene897244               1    0    1    0    0   2
Phalaenopsis-gene897282               1    0    1    1    0   3
Phalaenopsis-gene897285               1    1    1    1    0   4
Phalaenopsis-gene897313               0    0    0    1    0   1
Phalaenopsis-gene897314               1    0    0    0    0   1
Phalaenopsis-gene897330               1    0    1    1    0   3
Phalaenopsis-gene897333               1    1    1    1    1   5
Phalaenopsis-gene897370               1    0    0    1    0   2
Phalaenopsis-gene897413               1    0    0    0    0   1
Phalaenopsis-gene897414               0    0    1    0    0   1
Phalaenopsis-gene897529               0    0    0    1    0   1
Phalaenopsis-gene897530               1    1    1    1    1   5
Phalaenopsis-gene89805                0    0    1    1    0   2
Phalaenopsis-gene898187               0    0    0    1    0   1
Phalaenopsis-gene898223               1    0    1    0    0   2
Phalaenopsis-gene89832                1    0    0    1    0   2
Phalaenopsis-gene898907               1    0    0    0    0   1
Phalaenopsis-gene898910               1    0    1    0    1   3
Phalaenopsis-gene898911               1    0    0    1    0   2
Phalaenopsis-gene898912               0    0    1    1    0   2
Phalaenopsis-gene898914               1    0    1    1    0   3
Phalaenopsis-gene898921               1    0    0    1    0   2
Phalaenopsis-gene898922               1    1    1    1    1   5
Phalaenopsis-gene899231               0    0    0    0    0   0
Phalaenopsis-gene899239               1    0    0    1    0   2
Phalaenopsis-gene899264               1    0    0    1    0   2
Phalaenopsis-gene8997                 1    0    0    0    0   1
Phalaenopsis-gene899785               1    0    0    1    0   2
Phalaenopsis-gene9                    1    0    1    0    0   2
Phalaenopsis-gene901203               1    1    1    1    1   5
Phalaenopsis-gene901204               1    1    1    1    1   5
Phalaenopsis-gene901209               1    0    1    0    0   2
Phalaenopsis-gene90124                1    0    1    1    0   3
Phalaenopsis-gene90145                1    0    1    1    0   3
Phalaenopsis-gene901631               1    1    1    1    1   5
Phalaenopsis-gene901638               1    0    0    1    0   2
Phalaenopsis-gene901939               1    1    1    1    1   5
Phalaenopsis-gene901940               1    0    0    1    0   2
Phalaenopsis-gene901946               1    1    1    1    1   5
Phalaenopsis-gene902296               1    0    1    1    0   3
Phalaenopsis-gene902322               1    0    0    0    0   1
Phalaenopsis-gene902323               0    0    1    1    0   2
Phalaenopsis-gene903001               0    0    1    1    0   2
Phalaenopsis-gene903025               1    0    1    1    0   3
Phalaenopsis-gene903265               1    0    0    1    0   2
Phalaenopsis-gene903266               1    1    1    1    1   5
Phalaenopsis-gene90367                1    1    1    1    1   5
Phalaenopsis-gene903703               1    0    1    0    0   2
Phalaenopsis-gene903708               1    0    0    0    0   1
Phalaenopsis-gene903720               1    0    0    0    0   1
Phalaenopsis-gene903751               1    0    0    1    0   2
Phalaenopsis-gene903755               1    0    0    0    0   1
Phalaenopsis-gene903762               1    1    0    1    0   3
Phalaenopsis-gene90388                1    0    0    1    0   2
Phalaenopsis-gene904183               1    1    1    1    1   5
Phalaenopsis-gene904192               0    0    0    1    0   1
Phalaenopsis-gene904198               1    0    1    1    0   3
Phalaenopsis-gene9042                 1    0    0    0    0   1
Phalaenopsis-gene904230               1    0    1    1    0   3
Phalaenopsis-gene904239               1    1    1    1    1   5
Phalaenopsis-gene904245               1    0    0    0    0   1
Phalaenopsis-gene904275               0    0    0    1    0   1
Phalaenopsis-gene904422               1    0    0    1    0   2
Phalaenopsis-gene904439               1    0    1    1    0   3
Phalaenopsis-gene904440               1    1    1    1    1   5
Phalaenopsis-gene904441               1    1    1    1    1   5
Phalaenopsis-gene904622               1    0    0    1    0   2
Phalaenopsis-gene904826               1    1    1    1    1   5
Phalaenopsis-gene904827               1    1    1    1    1   5
Phalaenopsis-gene905235               0    0    0    1    0   1
Phalaenopsis-gene905255               1    1    1    1    0   4
Phalaenopsis-gene905276               1    1    1    1    1   5
Phalaenopsis-gene905653               1    1    1    1    1   5
Phalaenopsis-gene906740               1    0    0    1    0   2
Phalaenopsis-gene906773               1    0    0    0    0   1
Phalaenopsis-gene906841               1    0    1    0    0   2
Phalaenopsis-gene906862               1    0    0    1    0   2
Phalaenopsis-gene907002               1    0    0    1    0   2
Phalaenopsis-gene907003               1    0    1    1    0   3
Phalaenopsis-gene907062               1    0    0    0    0   1
Phalaenopsis-gene907560               1    0    1    0    0   2
Phalaenopsis-gene907561               1    0    1    1    0   3
Phalaenopsis-gene907578               1    0    1    1    0   3
Phalaenopsis-gene907580               0    1    1    1    1   4
Phalaenopsis-gene907581               1    0    0    1    0   2
Phalaenopsis-gene907601               0    1    0    1    0   2
Phalaenopsis-gene907602               1    1    1    1    1   5
Phalaenopsis-gene907604               1    0    1    0    0   2
Phalaenopsis-gene907606               1    0    1    0    0   2
Phalaenopsis-gene907846               1    1    1    1    1   5
Phalaenopsis-gene907882               1    0    0    1    0   2
Phalaenopsis-gene907912               1    1    1    1    1   5
Phalaenopsis-gene907939               1    1    1    1    1   5
Phalaenopsis-gene908004               0    0    0    1    0   1
Phalaenopsis-gene908029               0    0    1    1    0   2
Phalaenopsis-gene908031               1    1    1    1    1   5
Phalaenopsis-gene909754               1    0    1    1    0   3
Phalaenopsis-gene909755               1    1    1    1    1   5
Phalaenopsis-gene9180                 1    0    0    1    0   2
Phalaenopsis-gene9187                 1    0    1    0    0   2
Phalaenopsis-gene9199                 1    1    1    1    1   5
Phalaenopsis-gene9200                 1    0    1    1    0   3
Phalaenopsis-gene9202                 1    0    1    1    0   3
Phalaenopsis-gene92999                1    1    1    1    1   5
Phalaenopsis-gene93130                1    1    1    1    1   5
Phalaenopsis-gene93131                1    0    0    0    0   1
Phalaenopsis-gene93246                1    0    0    1    1   3
Phalaenopsis-gene93368                1    1    1    1    1   5
Phalaenopsis-gene9343                 1    0    1    1    0   3
Phalaenopsis-gene93684                1    1    0    1    0   3
Phalaenopsis-gene93722                1    0    1    0    0   2
Phalaenopsis-gene9376                 0    0    1    0    0   1
Phalaenopsis-gene9377                 0    1    1    0    0   2
Phalaenopsis-gene93998                1    1    1    1    1   5
Phalaenopsis-gene93999                0    0    0    1    0   1
Phalaenopsis-gene9457                 1    1    1    1    1   5
Phalaenopsis-gene9458                 1    0    1    1    0   3
Phalaenopsis-gene9508                 1    0    0    0    0   1
Phalaenopsis-gene9538                 0    0    1    0    0   1
Phalaenopsis-gene95389                1    0    1    1    0   3
Phalaenopsis-gene9539                 1    0    1    1    0   3
Phalaenopsis-gene95390                1    0    1    1    0   3
Phalaenopsis-gene95401                0    0    0    1    0   1
Phalaenopsis-gene95483                1    0    0    1    0   2
Phalaenopsis-gene95555                0    1    0    1    0   2
Phalaenopsis-gene95591                1    0    1    0    0   2
Phalaenopsis-gene95628                0    0    1    0    0   1
Phalaenopsis-gene95948                1    1    1    1    1   5
Phalaenopsis-gene96061                1    0    0    0    0   1
Phalaenopsis-gene96072                0    0    1    0    0   1
Phalaenopsis-gene96080                0    0    1    0    0   1
Phalaenopsis-gene96102                1    0    0    0    0   1
Phalaenopsis-gene96798                1    1    1    1    1   5
Phalaenopsis-gene96799                1    0    1    1    0   3
Phalaenopsis-gene96808                1    1    1    1    1   5
Phalaenopsis-gene96886                0    0    0    1    0   1
Phalaenopsis-gene96893                1    1    1    1    1   5
Phalaenopsis-gene96968                1    0    0    0    0   1
Phalaenopsis-gene96971                0    0    0    0    0   0
Phalaenopsis-gene96972                1    0    0    0    0   1
Phalaenopsis-gene97053                1    1    1    1    1   5
Phalaenopsis-gene97054                0    0    1    1    0   2
Phalaenopsis-gene97057                1    0    0    1    0   2
Phalaenopsis-gene97058                0    0    1    1    0   2
Phalaenopsis-gene97139                1    1    1    1    1   5
Phalaenopsis-gene97143                1    0    1    0    0   2
Phalaenopsis-gene97831                1    0    0    0    0   1
Phalaenopsis-gene97855                0    0    0    1    0   1
Phalaenopsis-gene97879                0    0    0    1    0   1
Phalaenopsis-gene97900                0    0    0    0    0   0
Phalaenopsis-gene98818                1    0    1    1    0   3
Phalaenopsis-gene98819                1    1    1    1    0   4
Phalaenopsis-gene99444                1    0    1    1    0   3
Phalaenopsis-gene99472                1    1    1    1    0   4
Phalaenopsis-gene99476                1    1    1    1    1   5
Phalaenopsis-gene99477                1    0    1    0    0   2
Phalaenopsis-gene99481                1    0    0    1    0   2
Phalaenopsis-gene99825                0    0    0    1    0   1
Phalaenopsis-gene99908                1    0    1    1    0   3
Phalaenopsis-gene99971                1    0    0    1    0   2
Phalaenopsis-gene432583               1    1    1    1    0   4
```

**Suppl. Table 3.** Distribution of the 20,000 nuclear baits across the five Orchidaceae subfamilies. For each bait (rows), 1/0 indicates recovery or absence in Apostasioideae (A; n = 16,070), Cypripedioideae (C; n = 8,390), Epidendroideae (E; n = 12,546), Orchidoideae (O; n = 15,903) and Vanilloideae (V; n = 7,108); the N_groups column reports the number of subfamilies in which the bait was recovered. The 6,682 baits with N_groups = 5 represent the set shared across all major clades.

| **Sample** | **Specie** | **Number raw reads (PE)** | **Number trimmed reads (PE)** | **% trimmed reads (PE)** | **Number mapped reads** | **% nuclear mapped reads** | **Number of nuclear baits with coverage >3** | **% plastid mapped reads** | **Number of platid baits with coverage >3** | **Number of nuclear SNPs** | **Number of species-specific nuclear SNPs** | **Number of SNPs differing from the reference species** | **Number of plastid SNPs** | **Number of species-specific plastid SNPs** |
| --- | --- | --- | --- | --- | --- | --- | --- | --- | --- | --- | --- | --- | --- | --- |
| **Catasetum_albovirens_1** | *C. albovirens* | Reference sequencing statistics reported in Pinheiro-Millet et al (in prep.) | | | | 91.03 | 8765 | 4.09 | 372 | 2255 | 143 | - | 182 | 29 |
| **Catasetum_aratrum_1** | *C. atratum* |  |  |  |  | 80.60 | 10466 | 3.39 | 375 | 2278 | 155 | - | 168 | 40 |
| **Catasetum arietinum_6119** | *C. arietinum* |  |  |  |  | 79.88 | 7491 | 4.73 | 300 | 2303 | 183 | - | 111 | 22 |
| **Catasetum_ariquemense_1** | *C. ariquemense* |  |  |  |  | 74.07 | 12428 | 5.34 | 371 | 1767 | 151 | - | 123 | 6 |
| **Catasetum_boyi_1** | *C. boyi* |  |  |  |  | 84.83 | 11661 | 5.05 | 366 | 1898 | 138 | - | 153 | 9 |
| **C._cernuum_1** | *C. cernuum* |  |  |  |  | 48.29 | 8279 | 5.35 | 359 | 2408 | 180 | - | 131 | 23 |
| **C. denticulatum_8** | *C. denticulatum* |  |  |  |  | 57.26 | 11590 | 3.52 | 373 | 2058 | 177 | - | 119 | 42 |
| **C._fimbriatum_1** | *C. fimbriatum* |  |  |  |  | 88.11 | 12407 | 1.38 | 373 | 1490 | 159 | - | 189 | 52 |
| **C._fuchsi_1** | *C. fuchsii* |  |  |  |  | 66.14 | 10943 | 4.15 | 356 | 2274 | 539 | - | 156 | 48 |
| **C. gardneri_1** | *C. gardneri* |  |  |  |  | 74.59 | 10807 | 4.70 | 372 | 2108 | 641 | - | 147 | 79 |
| **C. hookeri_6030** | *C. hookeri* |  |  |  |  | 57.10 | 4570 | 0.32 | 149 | 2448 | 694 | - | 161 | 61 |
| **C._juruenense_1** | *C. juruenense* |  |  |  |  | 63.18 | 11412 | 2.57 | 373 | 2083 | 485 | - | 126 | 48 |
| **C. lanciferum_6429** | *C. lanciferum* |  |  |  |  | 70.58 | 7524 | 0.09 | 199 | 2431 | 95 | - | 135 | 33 |
| **C._matogrossense_1** | *C. matogrossense* |  |  |  |  | 64.48 | 10600 | 2.62 | 313 | 1878 | 195 | - | 156 | 75 |
| **C._osakadianum_2** | *C. osakadianum* |  |  |  |  | 35.28 | 9270 | 39.10 | 296 | 2122 | 191 | - | 199 | 65 |
| **C._osculatum_1** | *C. osculatum* |  |  |  |  | 78.19 | 11725 | 4.07 | 375 | 2278 | 190 | - | 117 | 43 |
| **C._parguazense_1** | *C. parguazense* |  |  |  |  | 87.68 | 8116 | 4.82 | 353 | 2260 | 165 | - | 140 | 66 |
| **C._pileatum_1** | *C. pileatum* |  |  |  |  | 59.72 | 10795 | 2.85 | 342 | 1920 | 122 | - | 137 | 54 |
| **C._planiceps_1** | *C. planiceps* |  |  |  |  | 98.58 | 3538 | 1.42 | 347 | 2424 | 152 | - | 153 | 59 |
| **C. pulchrum_7648** | *C. pulchrum* |  |  |  |  | 71.34 | 12917 | 3.29 | 377 | 1601 | 176 | - | 143 | 72 |
| **C. tigrinum_2** | *C. tigrinum* |  |  |  |  | 93.78 | 11446 | 5.51 | 385 | 2369 | 164 | - | 116 | 39 |
| **C. vinaceum_5641** | *C. vinaceum* |  |  |  |  | 40.53 | 8240 | 9.73 | 217 | 2208 | 124 | - | 83 | 78 |
| **P4** | *C. albovirens* | 56560929 | 54246371 | 95.91 | 36742510 | 67.73 | 11846 | - | - | 2564 | 143 | 12 | - | - |
| **P64** | *C. albovirens* | 20213606 | 19335317 | 95.65 | 3795851 | 19.63 | 8621 | - | - | 2115 | 143 | 18 | - | - |
| **P14** | *C. atratum* | 18926345 | 18055929 | 95.4 | 6715422 | 37.19 | 4750 | - | - | 1964 | 155 | 20 | - | - |
| **P48** | *C. atratum* | 2783314 | 2532984 | 91.01 | 965175 | 38.10 | 8050 | - | - | 1434 | 155 | 14 | - | - |
| **P60** | *C. atratum* | 3424477 | 3279175 | 95.76 | 2220566 | 67.72 | 7431 | - | - | 2039 | 155 | 22 | - | - |
| **P52** | *C. arietinum* | 61586 | 59455 | 96.54 | 40893 | 68.78 | 514 | - | - | 1826 | 183 | 15 | - | - |
| **P65** | *C. ariquemense* | 12525205 | 12091858 | 96.54 | 8822561 | 72.96 | 9947 | - | - | 2013 | 151 | 18 | - | - |
| **P70** | *C. ariquemense* | 17803812 | 17210861 | 96.67 | 16838870 | 97.84 | 10157 | - | - | 2234 | 151 | 22 | - | - |
| **P69** | *C. boyi* | 4927135 | 4734776 | 96.1 | 3176004 | 67.08 | 8102 | - | - | 1302 | 138 | 16 | - | - |
| **P10** | *C. cernuum* | 5271367 | 5035219 | 95.52 | 3923603 | 77.92 | 3358 | - | - | 2098 | 180 | 26 | - | - |
| **P16** | *C. denticulatum* | 3427297 | 3301628 | 96.33 | 2443735 | 74.02 | 7646 | - | - | 1374 | 177 | 32 | - | - |
| **P21** | *C. denticulatum* | 13870545 | 13119142 | 94.58 | 5365124 | 40.90 | 9353 | - | - | 2397 | 177 | 40 | - | - |
| **P24** | *C. denticulatum* | 12710291 | 12193517 | 95.93 | 1425657 | 11.69 | 7608 | - | - | 1971 | 177 | 28 | - | - |
| **P25** | *C. denticulatum* | 9599569 | 9201284 | 95.85 | 4876229 | 53.00 | 9095 | - | - | 1398 | 177 | 22 | - | - |
| **P26** | *C. denticulatum* | 16875164 | 16221499 | 96.13 | 6531950 | 40.27 | 9212 | - | - | 1280 | 177 | 20 | - | - |
| **P3** | *C. denticulatum* | 48019677 | 45924971 | 95.64 | 29651033 | 64.56 | 11093 | - | - | 1960 | 177 | 30 | - | - |
| **P38** | *C. denticulatum* | 8356507 | 8090864 | 96.82 | 6022143 | 74.43 | 9482 | - | - | 1738 | 177 | 25 | - | - |
| **P41** | *C. denticulatum* | 2749420 | 2649514 | 96.37 | 2445928 | 92.32 | 7737 | - | - | 1449 | 177 | 22 | - | - |
| **P42** | *C. denticulatum* | 6271273 | 6030102 | 96.15 | 4152134 | 68.86 | 8553 | - | - | 1848 | 177 | 29 | - | - |
| **P7** | *C. denticulatum* | 21952813 | 21252560 | 96.81 | 20512669 | 96.52 | 10461 | - | - | 2412 | 177 | 38 | - | - |
| **P8** | *C. denticulatum* | 1730447 | 1645496 | 95.09 | 580067 | 35.25 | 6917 | - | - | 1597 | 177 | 23 | - | - |
| **P9** | *C. denticulatum* | 3899507 | 3768420 | 96.64 | 2461833 | 65.33 | 7926 | - | - | 1973 | 177 | 27 | - | - |
| **P50** | *C. fimbriatum* | 2764338 | 2626795 | 95.02 | 1238039 | 47.13 | 5625 | - | - | 2007 | 159 | 15 | - | - |
| **P66** | *C. fuchsii* | 13778447 | 13282771 | 96.4 | 8969376 | 67.53 | 9826 | - | - | 1279 | 539 | 58 | - | - |
| **P68** | *C. gardneri* | 10077354 | 9690126 | 96.16 | 7190658 | 74.21 | 9750 | - | - | 1287 | 641 | 18 | - | - |
| **P47** | *C. hookeri* | 5410678 | 5200584 | 96.12 | 4216619 | 81.08 | 8856 | - | - | 2268 | 694 | 20 | - | - |
| **P15** | *C. juruenense* | 9912087 | 9470347 | 95.54 | 7995769 | 84.43 | 9398 | - | - | 1273 | 485 | 55 | - | - |
| **P56** | *C. juruenense* | 3994025 | 3893748 | 97.49 | 3499128 | 89.87 | 8779 | - | - | 1622 | 485 | 60 | - | - |
| **P17** | *C. lanciferum* | 8773618 | 8535240 | 97.28 | 5179638 | 60.69 | 8867 | - | - | 1798 | 95 | 7 | - | - |
| **P19** | *C. lanciferum* | 8971978 | 8646647 | 96.37 | 4041747 | 46.74 | 8620 | - | - | 1464 | 95 | 10 | - | - |
| **P2** | *C. lanciferum* | 99644760 | 95079823 | 95.42 | 45483753 | 47.84 | 11593 | - | - | 1754 | 95 | 12 | - | - |
| **P20** | *C. lanciferum* | 7295309 | 7067199 | 96.87 | 6467192 | 91.51 | 9177 | - | - | 1757 | 95 | 8 | - | - |
| **P22** | *C. lanciferum* | 9974187 | 9464759 | 94.89 | 8016262 | 84.70 | 9493 | - | - | 2027 | 95 | 9 | - | - |
| **P27** | *C. lanciferum* | 5247804 | 5029126 | 95.83 | 3571529 | 71.02 | 8697 | - | - | 2292 | 95 | 11 | - | - |
| **P30** | *C. lanciferum* | 4968567 | 4844661 | 97.51 | 3736291 | 77.12 | 7829 | - | - | 1292 | 95 | 6 | - | - |
| **P37** | *C. lanciferum* | 9409875 | 8961315 | 95.23 | 5931553 | 66.19 | 8430 | - | - | 1672 | 95 | 12 | - | - |
| **P40** | *C. lanciferum* | 919659 | 876503 | 95.31 | 661727 | 75.50 | 7756 | - | - | 2014 | 95 | 9 | - | - |
| **P5** | *C. lanciferum* | 113983970 | 110027630 | 96.53 | 80665163 | 73.31 | 12349 | - | - | 2294 | 95 | 6 | - | - |
| **P58** | *C. matogrossense* | 9702794 | 9423691 | 97.12 | 9086803 | 96.43 | 9465 | - | - | 1506 | 195 | 20 | - | - |
| **P53** | *C. osakadianum* | 19957289 | 19265462 | 96.53 | 11894099 | 61.74 | 9637 | - | - | 1318 | 191 | 18 | - | - |
| **P59** | *C. osculatum* | 15799735 | 15125064 | 95.73 | 8595006 | 56.83 | 9837 | - | - | 1764 | 190 | 22 | - | - |
| **P1** | *C. parguazense* | 66038704 | 63402641 | 96.01 | 32000506 | 50.47 | 11072 | - | - | 2430 | 165 | 36 | - | - |
| **P11** | *C. parguazense* | 3931648 | 3806034 | 96.81 | 2841740 | 74.66 | 7049 | - | - | 1456 | 165 | 28 | - | - |
| **P12** | *C. parguazense* | 6709650 | 6440535 | 95.99 | 2779759 | 43.16 | 8239 | - | - | 1407 | 165 | 30 | - | - |
| **P28** | *C. parguazense* | 9935056 | 9477605 | 95.4 | 3263679 | 34.44 | 8650 | - | - | 1818 | 165 | 33 | - | - |
| **P31** | *C. parguazense* | 14010247 | 13366566 | 95.41 | 6136305 | 45.91 | 9186 | - | - | 1422 | 165 | 24 | - | - |
| **P39** | *C. parguazense* | 3453974 | 3279883 | 94.96 | 1906657 | 58.13 | 7457 | - | - | 1559 | 165 | 29 | - | - |
| **P44** | *C. parguazense* | 10692850 | 10250780 | 95.87 | 5331235 | 52.01 | 9351 | - | - | 1787 | 165 | 35 | - | - |
| **P6** | *C. parguazense* | 113104345 | 108527268 | 95.95 | 48557869 | 44.74 | 12001 | - | - | 2117 | 165 | 40 | - | - |
| **P45** | *C. pileatum* | 4768178 | 4571143 | 95.87 | 3553744 | 77.74 | 8137 | - | - | 1600 | 122 | 16 | - | - |
| **P23** | *C. planiceps* | 3375354 | 3120170 | 92.44 | 1251910 | 40.12 | 5828 | - | - | 1400 | 152 | 20 | - | - |
| **P46** | *C. planiceps* | 6995894 | 6758810 | 96.61 | 4449300 | 65.83 | 8899 | - | - | 1784 | 152 | 23 | - | - |
| **P57** | *C. pulchrum* | 5423093 | 5291099 | 97.57 | 4862966 | 91.91 | 8448 | - | - | 1386 | 176 | 18 | - | - |
| **P63** | *C. pulchrum* | 21241525 | 20121652 | 94.73 | 2639598 | 13.12 | 7534 | - | - | 1566 | 176 | 21 | - | - |
| **P51** | *C. tigrinum* | 24360136 | 23505302 | 96.49 | 16188436 | 68.87 | 10160 | - | - | 1575 | 164 | 19 | - | - |
| **P61** | *C. vinaceum* | 11137053 | 10760862 | 96.62 | 8151616 | 75.75 | 9223 | - | - | 1887 | 124 | 17 | - | - |

**Suppl. Table 4.** Sequencing statistics, SNP recovery, and pollinarium–reference divergence for *Catasetum*. The table reported sequencing and mapping statistics for both reference species and 56 pollinaria samples. For each pollinarium, we provided raw and trimmed read counts, mapping efficiency, nuclear bait recovery, total nuclear SNPs, the subset of species-specific diagnostic SNPs used for assignment, and the number of SNPs differing from the corresponding reference species. Reference-level capture statistics (nuclear and plastid mapped reads and baits recovered) were reported for each species and were repeated across conspecific pollinarium to facilitate direct comparison between intraspecific divergence and species-level genomic representation. The species-specific nuclear and plastid SNPs were defined from the reference dataset. In contrast, pollinarium-specific SNP differences represented private or low-frequency variants that were not present in the sequenced reference individuals.

| **Species** | **No. accessions** | **Species-specific SNPs (N)** | **Sites different (N)** | **% of intraspecific SNPs variation** |
| --- | --- | --- | --- | --- |
| *C. pulchrum* | 4 | 176 | 29 | 16,6 |
| *C. denticulatum* | 7 | 177 | 26 | 14,6 |
| *C. tigrinum* | 2 | 164 | 12 | 7,3 |
| *C. hookeri* | 2 | 694 | 37 | 5,3 |
| *C. lanciferum* | 3 | 95 | 4 | 4,7 |
| *C. gardneri* | 2 | 641 | 0 | 0 |

**Suppl. Table 5.** Intraspecific polymorphism of species-specific diagnostic SNPs.
